# Cobalt‐Catalyzed Asymmetric Construction of Boron Stereocenters With C–N Axial Chirality

**DOI:** 10.1002/advs.77047

**Published:** 2026-08-10

**Authors:** Subramani Kumaran, Harihara Subramanian Ravi Sankar, Pritam Maity, J. Richard Premkumar, Abir Das, Basker Sundararaju

**Affiliations:** ^1^ Department of Chemistry Indian Institute of Technology Kanpur Kanpur Uttar Pradesh India; ^2^ PG & Research Department of Chemistry Bishop Heber College Tiruchirappalli Tamil Nadu India

**Keywords:** asymmetric catalysis, atropisomers, boron, cobalt

## Abstract

The efficient construction of boron stereogenic centers alongside axial chirality remains a significant synthetic challenge, particularly under one‐pot conditions. Herein, we report a cobalt/Salox‐catalyzed enantioselective annulation strategy that enables the simultaneous formation of B‐stereogenic frameworks and a C─N chiral axis via C─H and N─H annulation of arylamides with dialkynylborons. This protocol achieves asymmetric desymmetrization at boron along with effective diastereocontrol on the C─N axis under mild conditions. A wide range of aryl amides and alkynes are well tolerated, delivering structurally diverse products in good yields with consistently high diastereo‐ and enantioselectivities. Mechanistic studies, including control experiments and isotope labelling, provide insight into the reaction pathway and the origin of stereocontrol. The resulting chiral boron‐containing compounds exhibit notable fluorescence, and their photophysical properties suggest potential utility in optoelectronic applications.

## Graphical abstract

A cobalt/Salox‐catalyzed one‐pot annulation enables enantioselective formation of *B*‐stereogenic centers and C─N axial chirality via C─H/N─H annulation, affording diverse fluorescent products with high stereocontrol and optoelectronic potential.

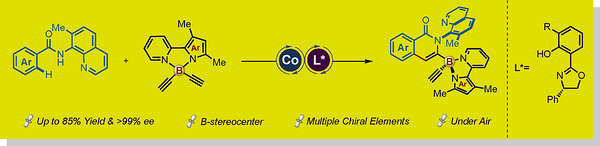

## 1 Introduction

**FIGURE 1:**
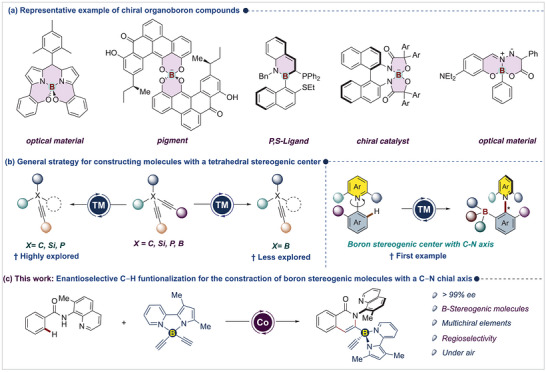
Construction of tetrahedral boron‐stereogenic compounds.

Over the past few decades, carbon‐centered stereogenicity has played a pivotal role in the fields of natural products, material science, and biological systems [1, 2, 3, 4, 5, 6, 7, 8]. Although stereogenic carbon atoms have long dominated asymmetric synthesis, increasing attention has recently shifted toward stereogenic centers based on tetracoordinate main‐group elements such as sulfur [9], phosphorus [10], and silicon [11]. These elements have attracted significant attention due to their unique structural features and their wide applications in agrochemicals, materials science, and organic electronic materials such as OLEDs. Among the main group elements, boron occupies a distinct position. In most cases, organoboron compounds exist as tricoordinate species characterized by an electron‐deficient boron center with a vacant *p*‐orbital [12]. However, upon coordination with a Lewis base, these tricoordinate boron compounds can accept an electron pair to form tetracoordinate boron complexes [13, 14, 15, 16]. The formation of such tetracoordinate boron species enables the generation of stereogenic boron centers, which has recently emerged as an intriguing area of research in modern synthetic chemistry. In this context, B‐stereogenic compounds have attracted considerable attention because of their potential applications in optical materials [17, 18, 19], asymmetric catalysis [20, 21], and natural products (Figure 1a) [22]. Nevertheless, the ligands coordinated to a tetracoordinate boron center are often labile, which can lead to rapid ligand exchange and consequently result in configurational instability [23, 24]. Therefore, the development of efficient enantioselective strategies for the construction of structurally diverse *B*‐stereogenic molecules remains an important objective in synthetic chemistry [25, 26, 27, 28, 29, 30, 31, 32, 33]. Among the available strategies, enantioselective desymmetrization reactions have emerged as powerful methods for generating complex chiral frameworks in a step‐ and atom‐economical fashion [34, 35, 36].

**SCHEME 1:**
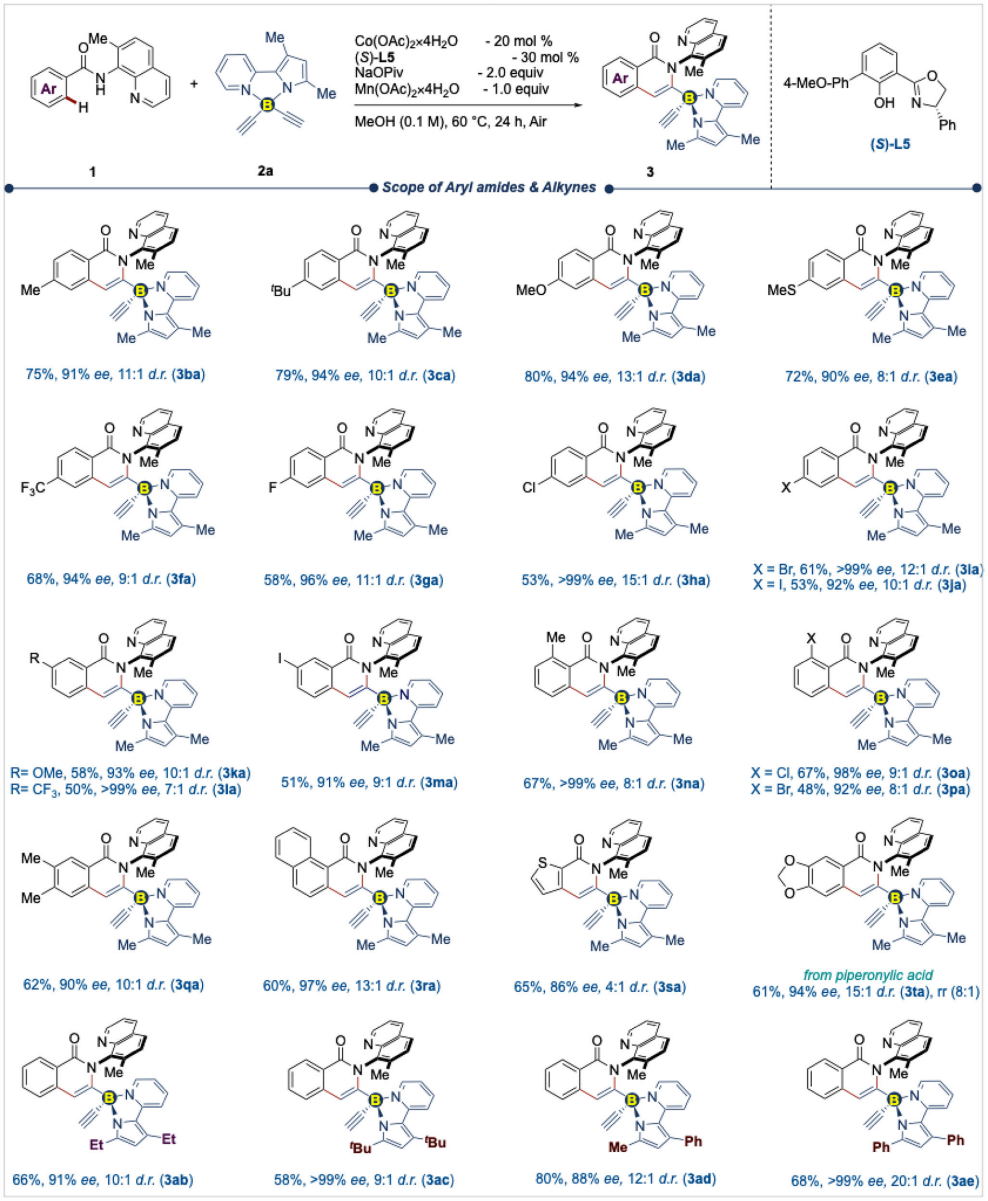
Scope of Aryl Amides and Alkynes.

Owing to their wide availability and distinctive reactivity, alkynes have proven to be versatile building blocks in such transformations. In particular, transition metal‐catalyzed desymmetrization of alkynes has become an attractive strategy for the generation of tetra‐substituted stereogenic centers (Figure 1b) [37, 38, 39]. In this context, in 2008, Tanaka and co‐workers reported the enantioselective synthesis of *P*‐stereogenic alkynylphosphine oxides through a [2 + 2 + 2] cycloaddition between symmetrical dialkynylphosphine oxide and 1,6‐diyne, using a cationic rhodium(I) catalyst, affording products with excellent enantiopurity [40]. Subsequently, in 2013, the group of Zhou demonstrated the construction of carbon‐centered stereogenic centers through a copper‐catalyzed azide‐alkyne cycloaddition reaction, providing the desired products in high yields with excellent stereoselectivity [41]. In addition, Nozaki and co‐workers achieved the enantioselective construction of *Si*‐stereogenic centers through a rhodium‐catalyzed desymmetrization strategy, delivering the products in excellent yields and enantiomeric excess [42]. Despite these advances in the enantioselective synthesis of stereogenic centers involving other main‐group elements such as carbon, silicon, phosphorus, and the asymmetric construction of B‐stereogenic compounds remains comparatively underexplored (Scheme 1b). More recently, He and co‐workers reported the first enantioselective synthesis of B‐stereogenic molecules through desymmetrization of dialkynylborons substrates via a copper‐catalyzed azide‐alkyne cycloaddition reaction, achieving excellent enantiocontrol [43]. Alongside these developments, transition metal‐catalyzed C─H bond functionalization has emerged as a powerful and atom‐economical strategy for constructing complex molecular architectures, including boron‐stereogenic frameworks. In this regard, Li and co‐workers recently reported a Cp^*^Rh(III)‐catalyzed enantioselective C─H annulation reaction that enabled the simultaneous formation of a B‐stereogenic center and C─B axial chirality with excellent yields and enantiopurity [44]. However, the specially designed Binol‐derived Cp‐type ligand required for Rh‐catalyzed C─H bond annulation is expensive, and modification further on the Cp‐framework is synthetically demanding. In recent years, enantioselective C─H bond functionalization catalyzed by earth‐abundant 3d transition metals has emerged as a sustainable alternative to fast depleting their noble counterpart from 4d and 5d metals [45, 46, 47, 48, 49]. Following the pioneering work of Daugulis [50], Co(II)‐catalyzed C─H bond functionalization assisted by a bidentate directing group has been extensively investigated [46, 47, 48, 49, 50]. Furthermore, the combination of cobalt(II) salts with enantiopure Salox ligand as chiral sources has enabled remarkable progress in asymmetric catalysis [51, 52, 53]. Building on the pioneering studies of Shi, Niu and Ackermann, further advanced by our group and others, this strategy has enabled the synthesis of a wide‐range of complex chiral molecular architectures featuring central [54, 55, 56, 57, 58, 59, 60, 61, 62, 63, 64, 65, 66, 67], axial [68, 69, 70, 71, 72], planar [73, 74, 75, 76] and inherent chirality [77, 78], as well as the system containing multiple stereogenic elements [79, 80, 81, 82, 83, 84, 85, 86, 87]. Nevertheless, to the best of our knowledge, the asymmetric synthesis of B‐stereogenic molecules bearing a C─N chiral axis through earth‐abundant transition metal‐catalyzed enantioselective C─H bond functionalization has not yet been reported (Figure 1b). This challenge likely arises from the relatively low reactivity of electron‐deficient alkyne substrates and the challenge in maintaining high chemoselectivity while avoiding double annulation, which could lead to the formation of racemic boron products. Herein, we report a novel strategy for the asymmetric synthesis of B‐stereogenic molecules bearing a C─N chiral axis through a cobalt/Salox catalyzed enantioselective C─H and N─H bond annulation between amides and dialkynylboron substrates. This transformation proceeds with excellent regioselectivity to deliver a highly regioisomeric product in good yields, along with excellent enantio‐ and diastereoselectivity (up to > 99% ee, > 20:1 dr) (Scheme 1c) [88, 89].

## 2 Results and Discussion

With the aim of constructing *B*‐stereogenic molecules bearing a C─N chiral axis through the desymmetrization of terminal alkynes, we initiated our preliminary investigation using *N*‐(7‐methylquinolin‐8‐yl)benzamide **1a** as the limiting reagent and 5,5‐diethynyl‐1,3‐dimethyl‐5H‐5l4,6l4‐pyrrolo[1',2':3,4][1,3,2]diazaborolo[1,5‐*a*]pyridine **2a** as the alkyne partner. Under the conditions of 20 mol% Co(OAc)_2_∙4H_2_O as the catalyst, 30 mol% (*S*)‐**L1** as the chiral ligand, 2.0 equiv of NaOPiv as the base and 1.0 equiv of Mn(OAc)_2_∙4H_2_O as the oxidant in MeOH at 60°C for 24 h, the desired B‐stereogenic product **3aa** was isolated in 48% yield with 73% enantiomeric excess and a 1.4:1 d.r. as a single regioisomer (Table 1, entry 1). To improve the yield and stereoselectivity, a series of derivatized Salox ligands ((*S*)‐**L2** to (*S*)‐**L5**) were subsequently evaluated. The results revealed that the presence of an ortho substituent on the Salox ligand is crucial for achieving high levels of enantio‐ and diastereoselectivity (entries 2 & 3, 9% & 3% ee, 1.2:1 & 1.3:1 dr). Notably, introducing an isobutyl group at the ortho position of the phenyl ring ((*S*)‐**L4**) significantly enhanced the enantioselectivity and moderately enhanced the diastereoselectivity (entry 4, 90% ee, 5.3:1 dr). Further optimization revealed that incorporation of a 4‐OMe‐Ph substituent on the Salox ligand ((*S*)‐**L5**) delivered **3aa** in improved yield (84%) with excellent enantio‐ and diastereoselectivity (entry 5, 98% ee, 20:1 dr). Subsequently, a range of solvents was screened (entries 6–9); however, none of them outperformed MeOH in delivering the desired product **3aa** (entry 5, 84% yield, 98% ee, 20:1 dr). Further investigations were conducted to elucidate the role of each reaction parameter. Control experiments revealed that the absence of cobalt catalyst, Salox ligand, base, or oxidant completely suppressed the reaction, highlighting the essential role of each parameter in the formation of the B‐stereogenic product (entries 11–14). To demonstrate the practicality of the developed protocol, a 1.0 mmol scale reaction was performed under the optimized conditions, affording the desired product in 66% yield with 94% enantiomeric excess and a 20:1 d.r. (entry 15). The structure of **3aa** was confirmed through X‐ray crystallographic analysis, and its absolute configuration was assigned as (*R*_a_,*S*)‐configuration.

**TABLE 1:** Reaction Optimization^a^ ^.^

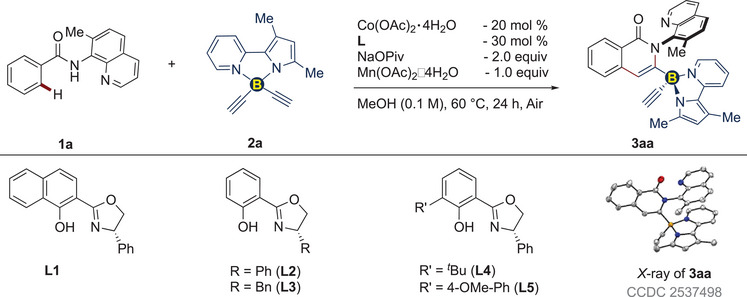						
Entry	L	Base	Solvent	Yield (%)^b^	*ee* (%)^c^	*dr* ^d^
1	**(*S*)‐L1**	NaOPiv	MeOH	48	73	1.4:1
2	**(*S*)‐L2**	NaOPiv	MeOH	76	09	1.2:1
3	**(*S*)‐L3**	NaOPiv	MeOH	58	03	1.3:1
4	**(*S*)‐L4**	NaOPiv	MeOH	68	90	5.3:1
5	**(*S*)‐L5**	NaOPiv	MeOH	84	98	20:1
6	**(*S*)‐L5**	NaOPiv	EtOH	70	96	21:1
7	**(*S*)‐L5**	NaOPiv	iPrOH	69	97	24:1
8	**(*S*)‐L5**	NaOPiv	*^t^* BuOH	45	96	21:1
9	**(*S*)‐L5**	NaOPiv	CH_3_CN	71	96	12:1
11	**(*S*)‐L5**	NaOPiv	MeOH	n.r.	n.d.^e^	n.d.
12		NaOPiv	MeOH	42	n.d.^f^	4:1
13	**(*S*)‐L5**		MeOH	19	74^g^	6.4:1
14	**(*S*)‐L5**	NaOPiv	MeOH	10	72^h^	4:1
15	**(*S*)‐L5**	NaOPiv	MeOH	66	94^i^	20:1

^a^ All reactions were carried out under air unless otherwise stated using **1a** (0.1 mmol), **2a** (0.15 mmol),Co(OAc)_2_·4H_2_O (20 mol%), Salox (*S*)‐**L** (30 mol%), NaOPiv (0.20 mmol) and Mn(OAc)_2_·4H_2_O (0.10 mmol) in MeOH (1.0 mL) at 60°C for 24 h.

^b^ Isolated yield.

^c^ The e.e. values were determined by chiral HPLC analysis.

^d^ d.r. calculated using ^1^H NMR from the crude mixture.

^e^ without Co(OAc)_2_·4H_2_O.

^f^ without (*S*)‐**L5**.

^g^ without NaOPiv.

^h^ without [Mn].

^i^ Performed on a 1.0 mmol scale. n.r. = no reaction, n.d. = not determined.

**FIGURE 2:**
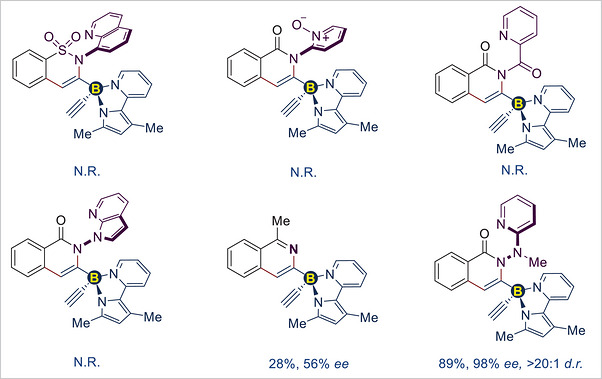
Evaluation of other bidentate directing groups.

Under the optimized conditions, several other bidentate directing groups such as Arylsulfonamide, pyridine *N*‐oxide, Picolinamide, aza‐indole, arylhydrazone derived from pyridyl hydrazine and acetophenone, *N*‐methylhydrazinyl pyridine were evaluated (Figure 2). Among the directing groups investigated, the arylhydrazone afforded the expected product with moderate yield and enantioselectivity, whereas *N*‐methylhydrazinyl pyridine emerged as the optimal substrate, delivering the product in exceptional yield (89%) and with excellent stereoselectivity (98% *ee*, >20:1 *dr*) (See the Supporting Information) [85].

With the optimized reaction conditions in hand, we explored the substrate scope of aryl amides, as shown in Scheme 1. A broad range of *para*‐, *meta*‐, and ortho‐substituted *N*‐(7‐methylquinolin‐8‐yl)benzamides were well tolerated, affording the corresponding B‐stereogenic products in good to excellent yields with high stereoselectivity. Benzamides bearing various electron‐donating and electron‐withdrawing groups at the para‐position reacted smoothly, providing the desired products (**3ba**–**3ja**) in 53%–80% yield, with enantiomeric excesses ranging from 90% to > 99% and diastereomeric ratios of 5:1 to 15:1. Importantly, functional groups such as Cl (**1** **h**), Br (**1i**), and I (**1j**) remained intact under the reaction conditions, offering useful handles for further transformations. The scope was further extended to *meta*‐substituted benzamides. Substrates containing ─OMe, ─CF_3_, and ─I groups at the *meta*‐position underwent efficient annulation to furnish the desired boron‐stereogenic products (**3ka**–**3ma**) in 50%–58% yield, with excellent enantioselectivity (91→99% ee) and diastereoselectivity (5:1–10:1 dr). Sterically hindered ortho‐substituted benzamides (**1n**–**1p**) were also compatible, delivering the corresponding products in moderate yields (48%–67%) with outstanding enantioselectivity (up to > 99% ee). Similarly, substrates such as naphthyl‐ and dimethyl‐substituted benzamides afforded products **3qa** and **3ra** in good yields (62% and 60%, respectively) with excellent stereocontrol (92%–99% ee, 10:1–13:1 dr). Notably, a heteroaromatic benzamide bearing a thiophene moiety (**1s**) was well tolerated and provided the desired product in 65% yield with high enantioselectivity (86% ee, 4:1 dr). A benzamide substrate incorporating a bioactive moiety also reacted efficiently, affording product **3ta** in 61% yield with excellent stereocontrol (94% ee, 8:1 dr). Encouraged by these results, we next investigated the scope of sterically and electronically diverse tetracoordinated boron species. Boron alkynes bearing an electron‐donating ─Et group on the pyrrole ring underwent smooth annulation to give product **3ab** in good yield (66%) with high enantio‐ and diastereoselectivity (91% ee, 10:1 d.r.). Furthermore, sterically demanding substituents such as –*^t^* Bu (**2c**) –Me & Ph (**2d**), and –Ph (**2e**) at the 2‐ and 4‐positions of the pyrrole ring were well tolerated, affording the desired products (**3ac**‐**3ae**) in good yields (66%–80%) without loss of enantioselectivity (91→99% ee).

**SCHEME 2:**
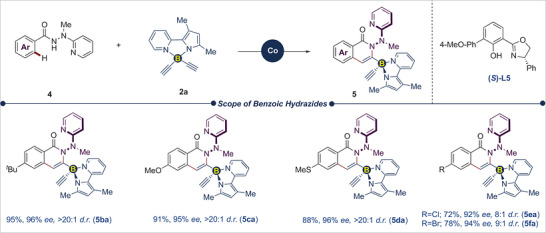
Scope of Benzoic Hydrazides. **4** (0.1 mmol), **2a** (0.15 mmol), Co(OAc)_2_·4H_2_O (20 mol%) (*S*)‐**L5** (30 mol%), Mn(OAc)_2_·4H_2_O (0.10 mmol) in CH_3_CN (1.0 mL) at 60°C for 16 h.

To demonstrate the broad applicability of the strategy, the protocol was subsequently extended to various benzoic hydrazide substrates (Scheme 2). A variety of electronically biased benzoic hydrazides, including ─^t^Bu, ─OMe, ─SMe, ─Cl, ─Br, were successfully coupled in this transformation, delivering the desired annulated product (**5ba**‐**5fa**) with yields ranging from 72% to 95% and stereoselectivity ranging from 92% to 96%.

**FIGURE 3:**
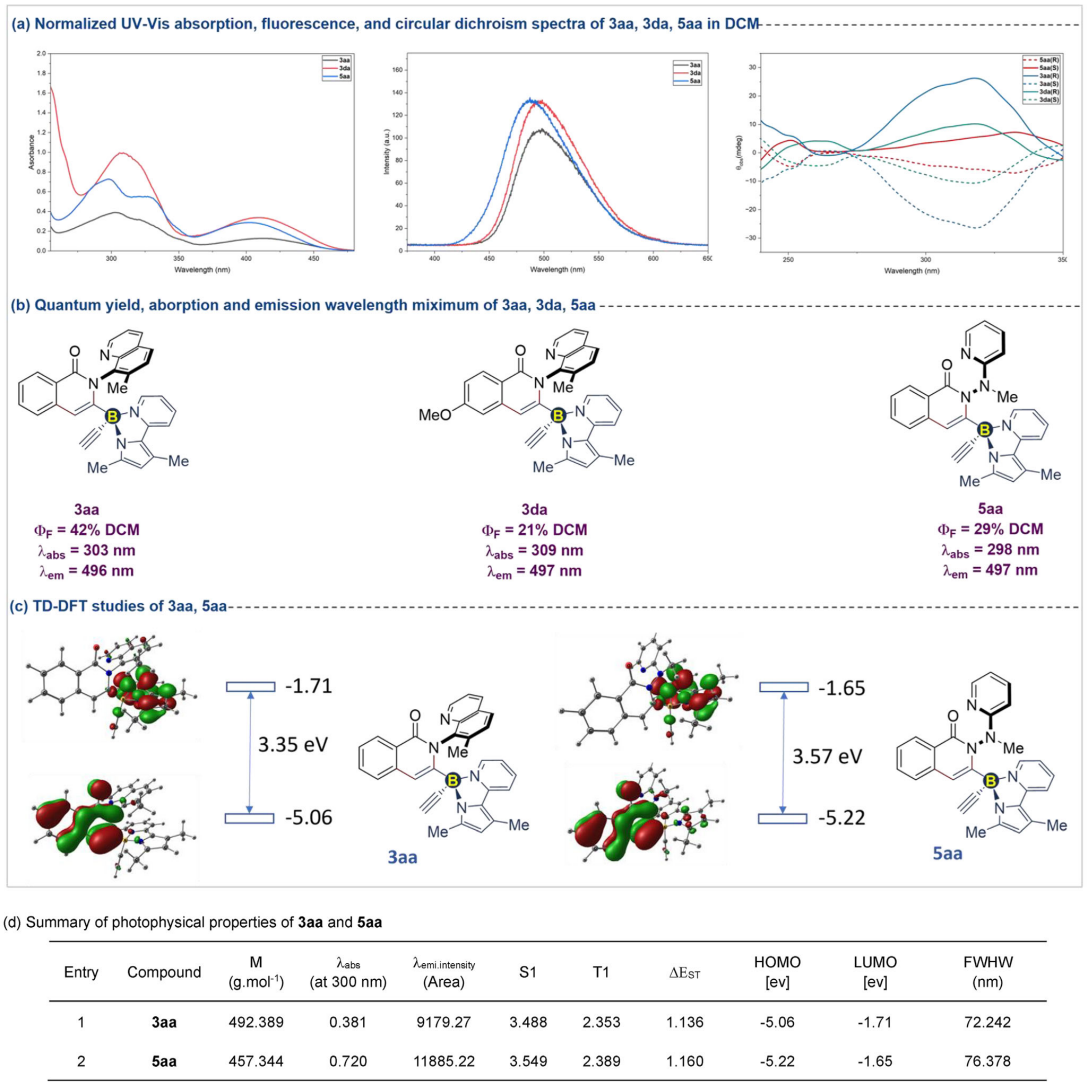
Photophysical properties and TD‐DFT studies of representative chiral compounds containing a *B*‐stereogenic center.

**SCHEME 3:**
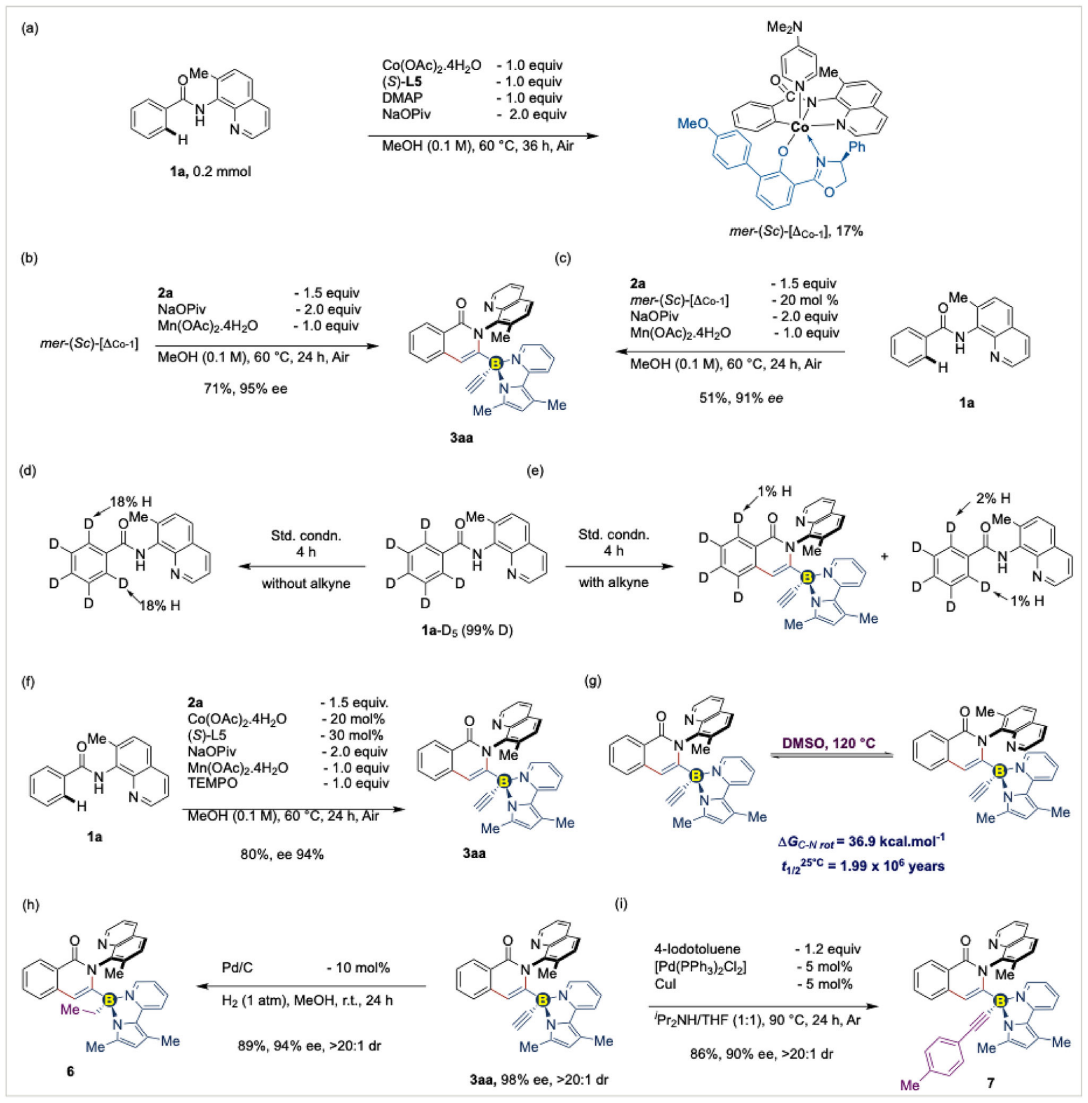
Mechanistic investigations. (a) Isolation of Cobaltacycle. (b)Stoichiometric experiment with cobaltacycle and alkyne 2a. (c) Standard reaction using Cobaltacycle as catalyst. (d)and (e) H/D exchange experiments without and with alkyne. (f) Experiment with TEMPO. (g) Determination of epimerization barriers along the C─N axis. (h, i) Post‐synthetic modifications.

Subsequently, representative tetracoordinate boron stereogenic compounds (**3aa**, **3** **da**, and **5aa**) were selected to evaluate their photophysical properties (Figure 3). Compounds **3aa** and **3** **da** exhibited strong absorption bands in the range of 298–303 nm, whereas compound **5aa** showed a slightly shifted absorption maximum at 309 nm in dichloromethane solution (Figure 3a) (2 × 10^−5^ M in DCM). Under identical conditions, all compounds displayed greenish‐yellow fluorescence, with emission maxima at 498 nm (**3aa**), 496 nm (**3** **da**) and 487 nm (**5aa**) (Figure 3a) (2 × 10^−5^ M in DCM). In addition, circular dichroism (CD) measurements revealed perfect mirror image Cotton effects for each enantiomeric pair, unequivocally confirming their enantiomeric relationship and opposite chiroptical properties (Figure 3a) (2 × 10^−5^ M in DCM). Moreover, quantum yield measurements revealed good‐to‐excellent efficiencies, ranging from 21% to 42% across the series (Figure 3b). Circularly polarized luminescence (CPL) measurements revealed mirror‐image emission profiles for each enantiomeric pair, with emission maxima at 573 nm for **3aa**, 557 nm for **3** **da**, and 545 nm for **5aa** (2 × 10^−5^ M in DCM). The corresponding luminescence dissymmetry factors (glum) were determined as 0.78 × 10^−3^ (**3aa**), 1.0 × 10^−3^ (**3** **da**), and 0.8 × 10^−3^ (**5aa**), respectively (See the Supporting Information). Additionally, TD‐DFT studies were carried out to elucidate the electronic properties of representative tetracoordinate boron compounds (**3aa** & **5aa**). The computed HOMO–LUMO energy gaps were found to be in the range from 3.35 to 3.57 eV (Figure 3c, see the Supporting Information). These results indicate that B‐stereogenic compounds bearing a C─N chiral axis possess promising photophysical characteristics (Scheme 3d), highlighting their potential for applications in chiroptical materials and devices.

To gain insight into the reaction pathway, a series of mechanistic studies were conducted to elucidate the underlying mechanism of the desymmetrization of boron alkynes, as outlined in Scheme 3. First, we sought to isolate the key cobaltacycle intermediate under the standard conditions. Accordingly, benzamide **1a** was treated with Co(OAc)_2_.4H_2_O, (*S*)‐**L5**, NaOPiv, Mn(OAc)_2_.4H_2_O and DMAP in MeOH under air for 36 h. Gratifyingly, the major diastereoisomer of the in situ formed octahedral cobaltacycle, denoted as *mer*‐[(*S*_L_)‐ΔCo], was successfully isolated in 17% yield (Scheme 3a). Subsequently, a stoichiometric reaction between *mer*‐[(*S*_L_)‐ΔCo] and dialkylboron **2a** afforded the desired product **3aa** in 71% yield with 95% enantiopurity (Scheme 3b). Furthermore, when *mer*‐[(*S*_L_)‐ΔCo] was employed as the catalyst under standard conditions, the expected product **3aa** was obtained in 51% yield and 91% ee (Scheme 3c). These results clearly support the involvement of the cobaltacycle *mer*‐[(*S*_L_)‐Δ_Co_] as the key intermediate in the catalytic cycle. To further probe the C─H activation step, D/H scrambling experiments were performed using [D5]‐**1a** in the absence of alkyne **2a** under standard reaction conditions. The experiment revealed 18% H incorporation at both *ortho* positions, indicating that the C─H activation step is reversible (Scheme 3d). In contrast, no D/H exchange was observed in either the recovered benzamide substrate or the annulated product when the reaction was conducted in the presence of alkynes **2a**, suggesting that alkyne insertion is significantly faster than the reverse C─H activation step (Scheme 3e) [90]. To examine the possibility of a radical pathway, the reaction was carried out in the presence of a radical scavenger, TEMPO. No significant decrease in the yield of the desired product was observed, indicating that a radical pathway is unlikely to be involved in this transformation (Scheme 3f). Furthermore, the conformational stability of the C─N chiral axis and *B*‐stereogenic center in compound **3aa** was evaluated. Epimerization studies revealed a rotational barrier ΔG*_C‐N_* of 36.90 kcal/mol, corresponding to a half‐life (t_1/2_) of 1.991 × 10^6^ years at room temperature (25°C). Similarly, the *B*‐stereogenic center exhibited a racemization barrier (ΔG*_B_*) of > 30 kcal mol^−1^ at room temperature (Scheme 3g). These findings highlight the exceptional configurational stability of both stereogenic elements in **3aa**. To further demonstrate the synthetic utility of product **3aa**, several derivatization reactions were performed (Scheme 3h,i). Enantiospecific hydrogenation of the alkyne moiety in the presence of Pd/C afforded the corresponding product **6** in 89% yield while fully preserving its stereochemical integrity (94% ee, > 20:1 dr). Moreover, Sonogashira cross‐coupling reaction between **3aa** and 4‐iodotoluene proceeded smoothly to furnish the desired product **7** without any significant loss of enantio‐ and diastereoselectivity (90% ee, > 20:1 dr).

### 2.1 DFT Studies

**FIGURE 4:**
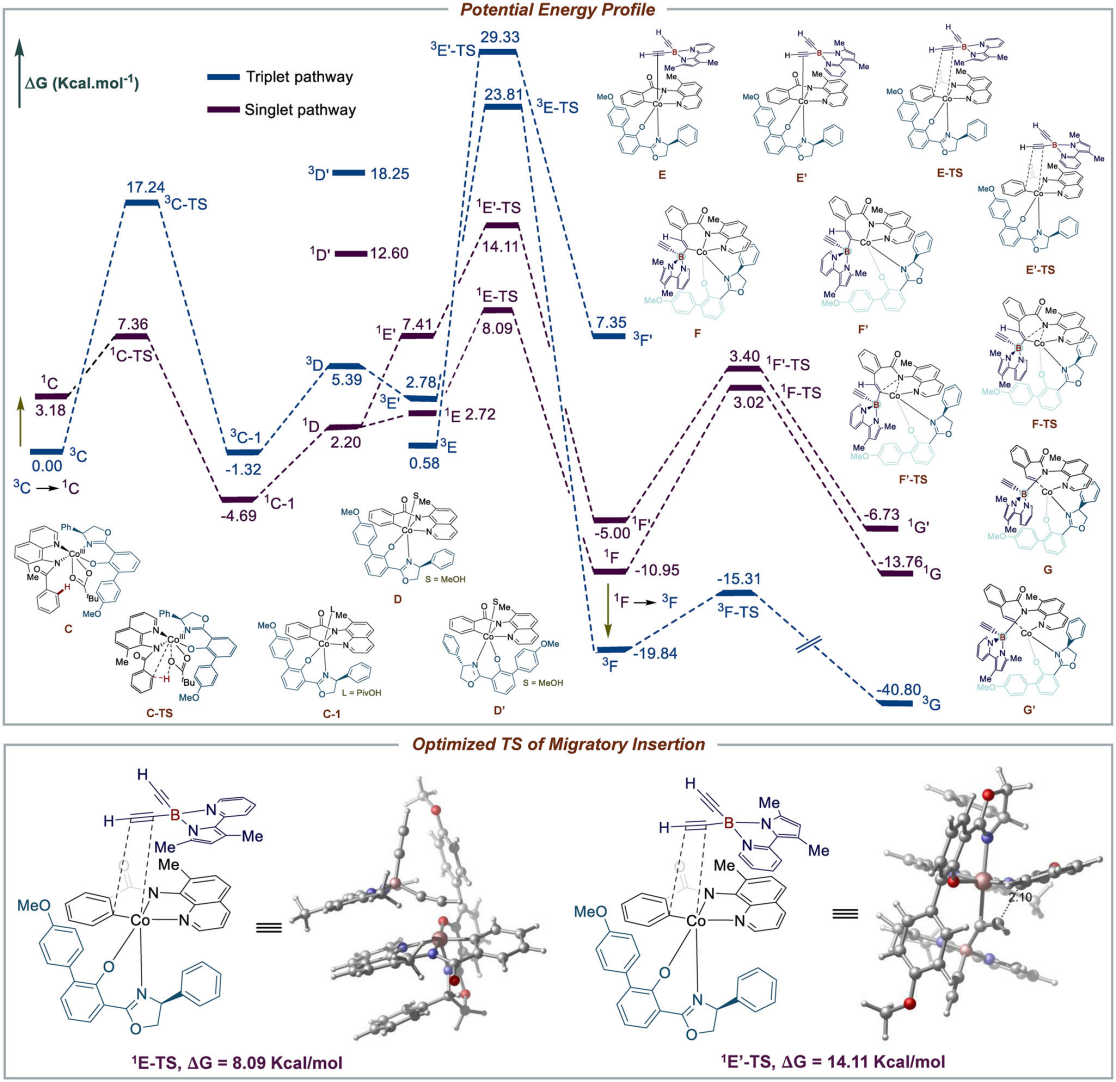
Free Energy Profile and optimized TS.

Density functional theory calculations were performed to gain mechanistic insight into the stereoselective desymmetrization of dialkynylborons **2a**, leading to **3aa** (Figure 4). The Gaussian 16 software package was used with its implemented M06 functional, the 6−31G(d) basis set for all main group elements, and the LANL2DZ (ECP) basis set for Co metal. Single‐point calculations were conducted at the M06‐L/6−311++G(d,p)‐SDD(ECP) level of theory. The reaction begins with the formation of intermediate **C‐1** from intermediate **C** via the **C‐TS**, following a concerted metalation and deprotonation pathway. The computed free energy barriers indicate that the singlet pathway is energetically preferred, as **^1^C‐**TS is 9.88 kcal/mol lower in energy than the analogous triplet transition state. Following ligand exchange with PivOH, intermediate **D** is generated. Evaluation of the **D**(Δ)‐ and **D’**(Λ)‐cobaltacycle isomers in both singlet and triplet spin states revealed a clear energetic preference for the singlet configuration. Among these, the Δ‐isomer was identified as the thermodynamically preferred species, exhibiting a ΔG of 10.4 kcal/mol relative to the Λ‐isomer. Subsequently, coordination of dialkynylboron **2a** to the cobalt center occurs via displacement of the coordinated MeOH solvent, leading to the formation of intermediate **E**. Comparison of the relative energies of the singlet and triplet spin states reveals that the triplet state is 2.14 kcal/mol lower in energy than the corresponding singlet state. The orientation of the heterocyclic substituent on dialkynylboron **2a** dictates the stereochemical outcome at the boron center. In both spin states, the (*S*)‐configured pathway proceeds through a lower‐energy transition state (**E‐TS**) than the corresponding (*R*)‐configured pathway (**E′‐TS**) (Figure 4). The singlet pathway is further favored by 15.72 kcal/mol, indicating that the migratory insertion step is responsible for the observed enantioselectivity. According to the calculated free energy profile, the migratory insertion step constitutes the rate‐determining step of the catalytic cycle, proceeding through **1E′‐TS**, which exhibits the highest activation barrier of 14.11 kcal/mol. The calculations further indicate that the reductive elimination step proceeds through a spin crossover from the singlet to the triplet spin state via the sequence **^1^F→^3^F→^3^F‐TS**. The subsequent reductive elimination intermediate afforded the intermediate **^3^G**, which is calculated to be −40.80 kcal/mol lower in free energy.

**SCHEME 4:**
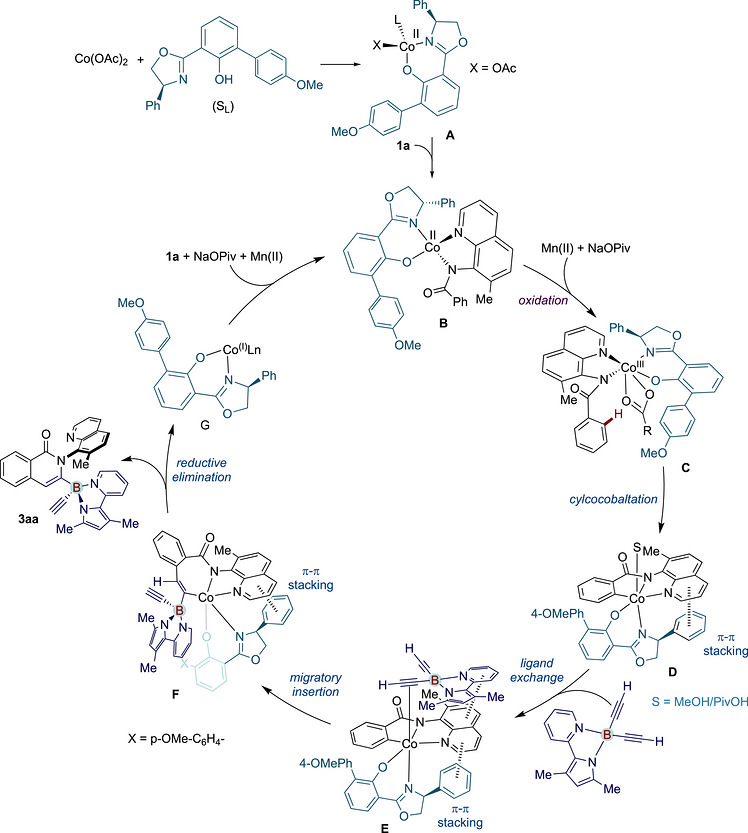
Plausible catalytic cycle.

Based on the mechanistic studies, DFT calculations, and previous literature reports [50, 51, 52, 53, 54, 55, 56, 57, 58, 59, 60, 61, 62, 63, 64, 65, 66, 67, 68, 69, 70, 71, 72, 73, 74, 75, 76, 77, 78, 79, 80, 81, 82, 83, 84, 85, 86, 87, 91, 92, 93, 94, 95], a plausible catalytic cycle for the construction of *B*‐stereogenic molecules is proposed, as depicted in Scheme 4. Initially, cobalt(II) acetate undergoes ligand exchange with the Salox ligand (*S*)‐**L5** and benzamide **1a**, generating the corresponding Co(II) intermediate **B**. Subsequently, the intermediate **B** undergoes oxidation in the presence of the oxidant and sodium pivalate to form the corresponding octahedral Co(III) pivalate intermediate **C**. Intermediate **C** further undergoes an enantio‐determining carboxylate‐assisted C─H activation to furnish the diastereoselective cobaltacycle **D** *mer*‐[(*S*_L_)‐ΔCo]. The resulting cobaltacycle **D** subsequently coordinates with the boron‐tethered alkyne **2**, leading to intermediate **E**.

## 3 Conclusion

In summary, we have developed an efficient and versatile method for the synthesis of *B*‐stereogenic molecules featuring a C─N chiral axis through a cobalt/Salox catalytic system, enabled by enantioselective C─H and N─H bond annulation between benzamides and dialkynylborons. This step‐economical approach provides access to a broad range of boron‐stereogenic chiral scaffolds under mild reaction conditions. Importantly, the protocol also furnishes optically active terminal alkynes, which serve as valuable building blocks for further synthetic applications. Epimerization studies revealed a high diastereomerization barrier (36.9 kcal/mol) and an exceptionally long half‐life, demonstrating the remarkable configurational stability of the products. In addition, these tetracoordinate boron stereogenic compounds exhibit bright fluorescence, highlighting their potential utility in optoelectronic materials. We anticipate that this methodology will expand the synthetic toolbox for boron‐stereogenic molecules and inspire the development of new asymmetric strategies for constructing other heteroatom‐based stereocenters.

## Author Contributions

S.K., H.S.R.S., A.D., and B.S. conceived the concept. S.K., H.S.R.S., P.M., and A.D. performed all the reactions and analyzed the products. J.R.P designed and performed all the computational studies and analyzed the results. S.K. and A.D. performed X‐ray diffraction analysis and analyzed the structure. The manuscript was written through the contribution of all authors.

## Conflicts of Interest

The authors declare no conflicts of interest.

## Supporting information




**Supporting File**: advs77047‐sup‐0001‐SuppMat.pdf.

## Data Availability

CCDC 2537498 (3aa) contains the supplementary crystallographic data for this paper. These data can be obtained free of charge from the Cambridge Crystallographic Data Centre via www.Ccdc.Cam.Ac.uk/Datarequest/cif. The supporting information also provides additional information.
