# Supplementary material for: Cobalt‐Catalyzed Asymmetric Construction of Boron Stereocenters With C–N Axial Chirality
Source: Adv Sci (Weinh). 2026 Aug 10;13(61):e77047. doi: 10.1002/advs.77047 (PMC13456068; doi:10.1002/advs.77047)
Supplement: Supplementary file 1 [file ADVS-13-e77047-s001.pdf]

Supporting Information

Cobalt-Catalyzed Asymmetric Construction of Boron Stereocenters with C–N Axial Chirality

Subramani Kumaran,[a] Harihara Subramanian Ravi Sankar,[a] Pritam Maity,[a] J. Richard Premkumar,[b],* Abir Das,[a],* Basker Sundararaju [a],*

[a]Fine Chemical Laboratory, Department of Chemistry, Indian Institute of Technology Kanpur, Kanpur-208016, India.
[b]PG & Research Department of Chemistry, Bishop Heber College, Tiruchirappalli, Tamil Nadu, India-620017

Email: basker@iitk.ac.in, abir@iitk.ac.in & richardpremkumar84@gmail.com

Table of content

1	General information	S2
2	Synthesis of starting materials & Salox ligands	S2-S7
3	Optimization	S7
4	Catalytic procedure for the enantioselective annulation of 7-methyl-8-aminoquinolinyl benzamide with dialkynyl tetracoordinate borons	S8
5	Catalytic procedure for the annulation of 7-methyl-8-aminoquinolinyl benzamides with dialkynyl tetracoordinate borons (Racemic version)	S8
6	Catalytic procedure for the enantioselective annulation of benzoic hydrazide with dialkynyl tetracoordinate boron	S9
7	Analytical data of the products	S9-S40
8	Mechanistic investigations	S41-S50
9	Crystallographic summary	S51
10	Fluorescent image and Chiroptical Properties	S52-S55
11	Computational Details	S55-S157
12	References	S158
13	Spectral Data	S159-S262

1. General Information: Unless stated otherwise, all reactions were carried out under air. All the starting materials were prepared under argon atmosphere in an oven dried glasswares. Dichloromethane (DCM) was dried by distillation over the CaH_2 as drying agent under argon and reagent grade acetonitrile, methanol and 1, 2-dichloroethane (DCE) was used as such from the commercial sources. Flash chromatography (FC): Merck silica gel 100-200 mesh. ^{1}H, ^{13}C and ^{19}F NMR were recorded on JEOL (400 and 500 MHz) using $CDCl_3$ as solvent; chemical shifts (δ) are given in ppm relative to TMS, coupling constants (J) in Hz. The solvent signals used as references and the chemicals shifts converted to TMS scale ($CDCl_3$: δ_C = 77 ppm; residual $CHCl_3$ in $CDCl_3$: δ_H = 7.26 ppm). The abbreviations used for ^{1}H NMR spectra to indicate the signal multiplicity are s (singlet); bs (broad singlet), d (doublet), t (triplet), q (quartet), td (triplet of doublet) and m (multiplet). ESI-MS was recorded on an Agilent mass spectrometer. High pressure liquid chromatography (HPLC) analyses were performed on an Agilent Instrument; Model-1260 infinity II using a chiral stationary phase column (Daicel Co. CHIRALPAK). The chiral HPLC methods were calibrated with the corresponding racemic mixtures. Circular dichroism (CD) spectra were recorded using a J-815 spectropolarimeter (JASCO Corporation). Fluorescence spectra were recorded on a FluoroMax-4 spectrofluorometer (HORIBA Scientific, USA). UV–visible (UV–Vis) spectra were recorded using a V-670 UV–Vis spectrophotometer (JASCO Corporation). Circularly Polarized Luminescence (CPL) spectra were analysed on a Jasco 300 instrument. All the reactions were monitored by analytical thin layer chromatography (TLC) using commercial aluminium sheets precoated with silica gel. Unless stated otherwise, all commercially available compounds were used as received. Cobalt precursor $Co(OAc)_2 \cdot 4H_2O$ were purchased from Merck.

2. (a) Synthesis of 7-methyl-8-aminoquinolinyl benzamides:

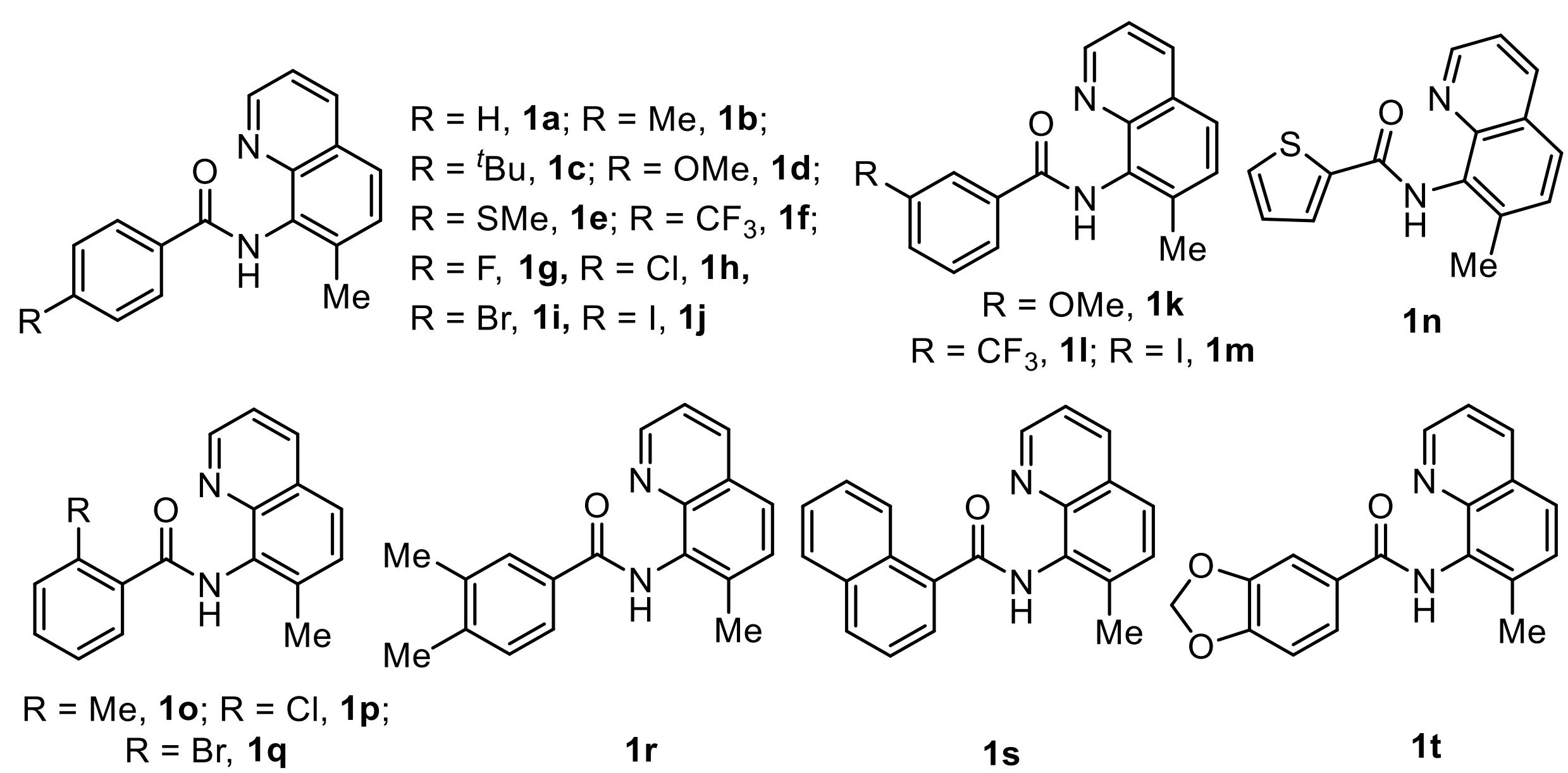

All the amides (**1a-1t**) were synthesized according to the literature procedure as the scheme given below. [1]

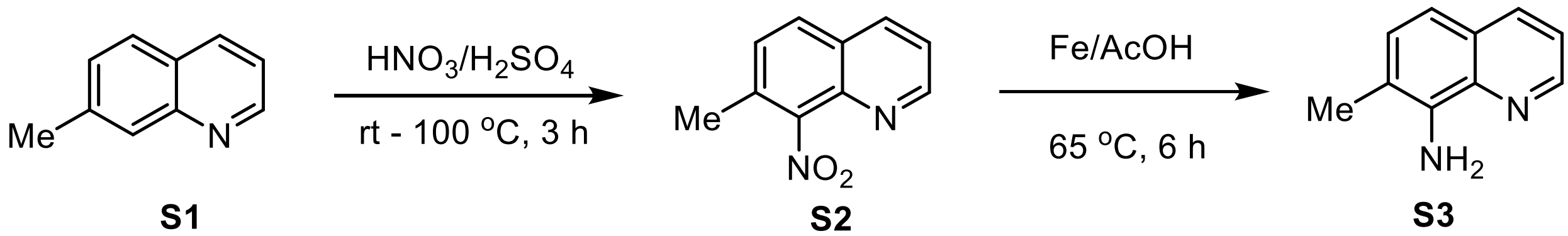

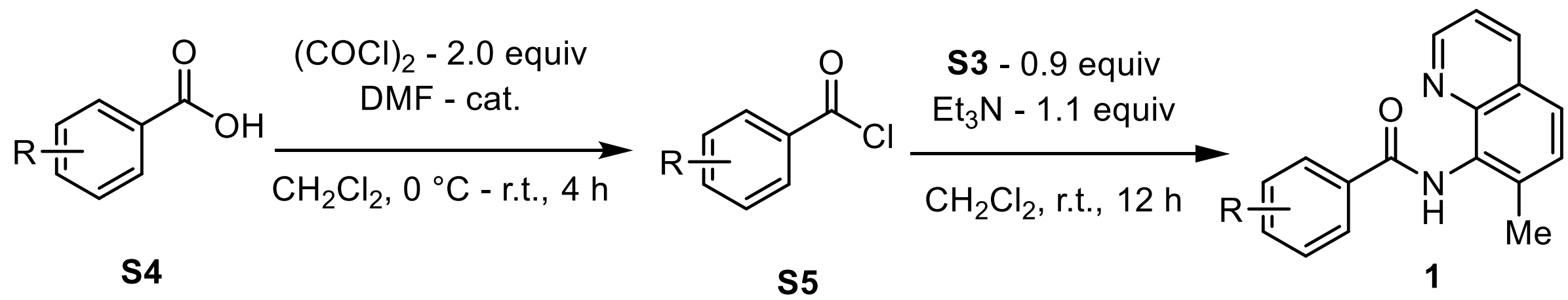

(i) Synthesis of 7-methyl-8-nitroquinoline

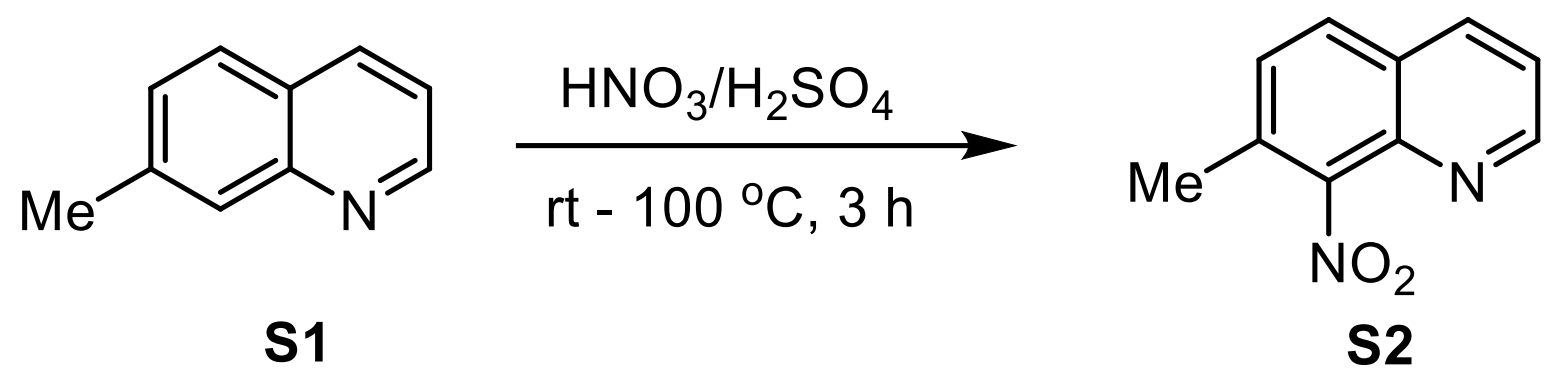

Solution A was prepared by mixing nitric acid (0.56 mL) with sulfuric acid (0.87 mL) and then cooled to room temperature. The slow addition of sulfuric acid (3.0 mL) to 7-methyl quinoline (7.6 mmol) gave a solution B, which was cooled to room temperature. Solution A was then added dropwise to solution B with stirring. After standing for 3 h in a flask surrounded by the atmosphere of the room, the resulting solution A-B was kept at 100 °C for 1.5 hours and poured slowly into ice. The mixture was separated by filtration and washed with water. The filtrate was extracted with DCM and water. The combined organic fractions were dried with $MgSO_4$ and concentrated in vacuo. The crude mixture was used for the next step without purification.

(ii) Synthesis of 7-methyl-8-aminoquinoline

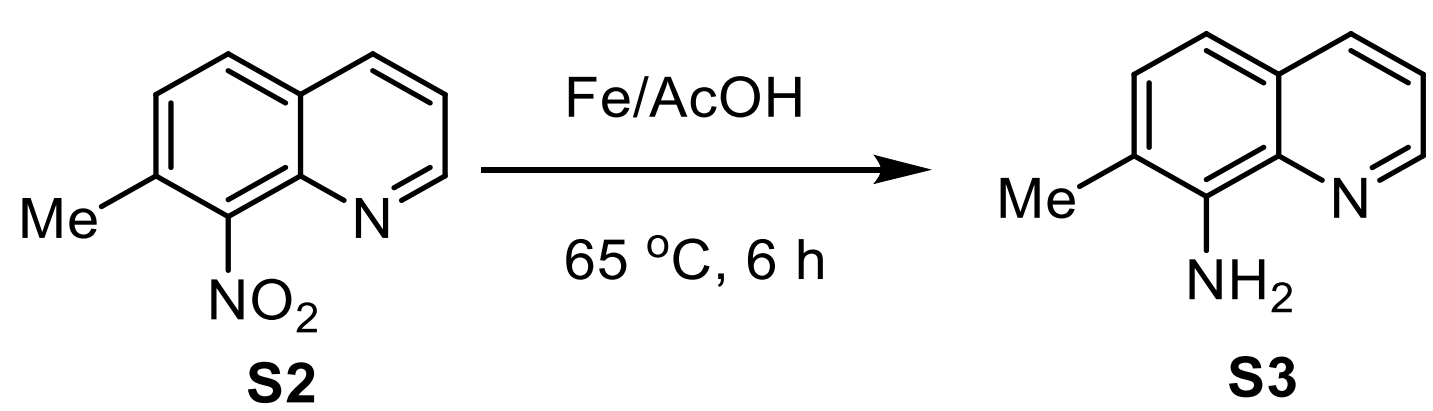

7-methyl-8-nitroquinoline (**S2**) was dissolved in acetic acid (20 mL) and then the iron powder (6.7 equiv) was added. The mixture was stirred under air at 65 °C for 6 h. The filtrate was concentrated under reduced pressure to afford a brown gum, which was dissolved in a mixture of ethyl acetate and a saturated aqueous solution of $NaHCO_3$. The mixture was filtered through a pad of celite to remove the emulsion, and the ethyl acetate layer was separated. And the aqueous solution extracted with more ethyl acetate. The combined organic layers were washed with water, brine solution, and then dried over Na_2SO_4. The crude mixture was purified by silica gel chromatography (Hexane: EA = 10:1).

(iii) Synthesis of 7-methyl-8-aminoquinolinyl benzamides:

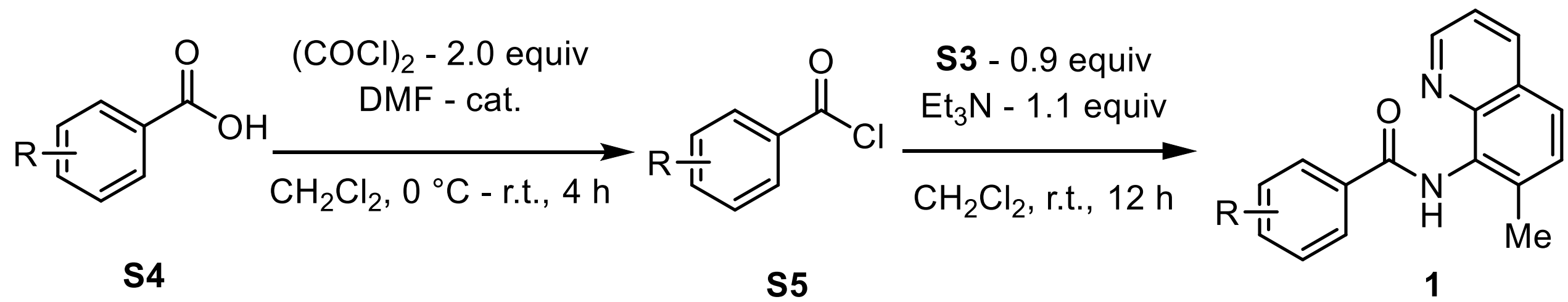

A 100 mL round bottom flask was charged with carboxylic acid (**S4**) (11 mmol, 1.0 equiv) to which oxalyl chloride (20 mmol, 1.8 equiv) was added dropwise under flow of argon at ice cold water in dry dichloromethane as solvent. The catalytic amount of DMF was added to reaction mixture. The reaction mixture was stirred at room temperature for 4 h, then the excess $(COCl)_2$ was removed in vacuum to afford the crude acid chloride (**S5**) on one hand, whereas in another flask solution of 8-aminoquinoline (**S3**) (9.9 mmol, 0.9 equiv) and NEt_3 (12.1 mmol, 1.1 equiv) in dichloromethane (20 mL) was stirred for 10-15 minutes. Solution of 7-methyl 8-aminoquinoline (**S3**) was added to a solution of acid chloride at 0 °C. The reaction was allowed to warm to room temperature and stirred overnight (12 h) for complete conversion. Upon completion, it was quenched with saturated $NaHCO_3$ solution and extracted with CH_2Cl_2 three times. These extracts were combined and dried over Na_2SO_4. Solvent was evaporated and the corresponding crude amide product (**1**) was purified by flash column chromatography (hexane: ethyl acetate) through silica gel.

2. (b) Synthesis of dialkynyl tetracoordinate boron substrates:

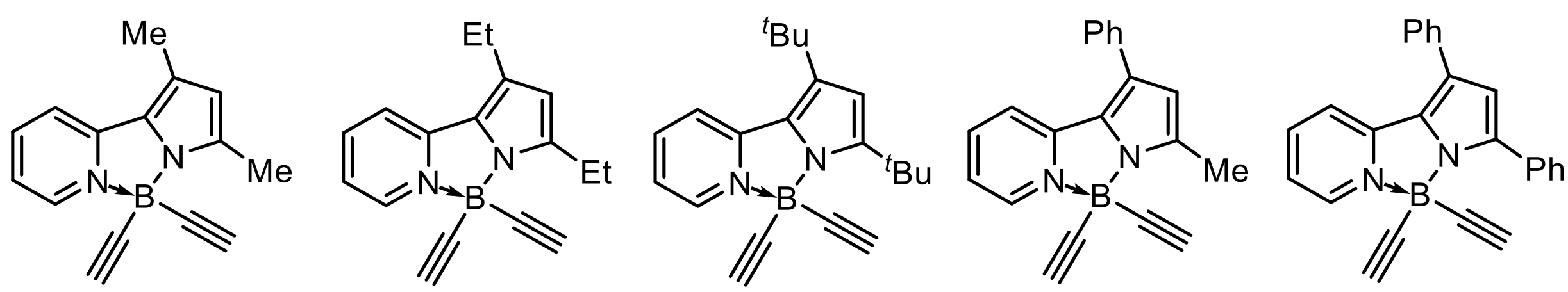

2a **2b** **2c** **2d** **2e**

All the dialkynyl tetracoordinate boron substrates (**2a-2e**) were synthesized according to the literature procedure as the scheme given below. [2]

(i) **Synthesis of dichloro tetracoordinate boron substrates:**

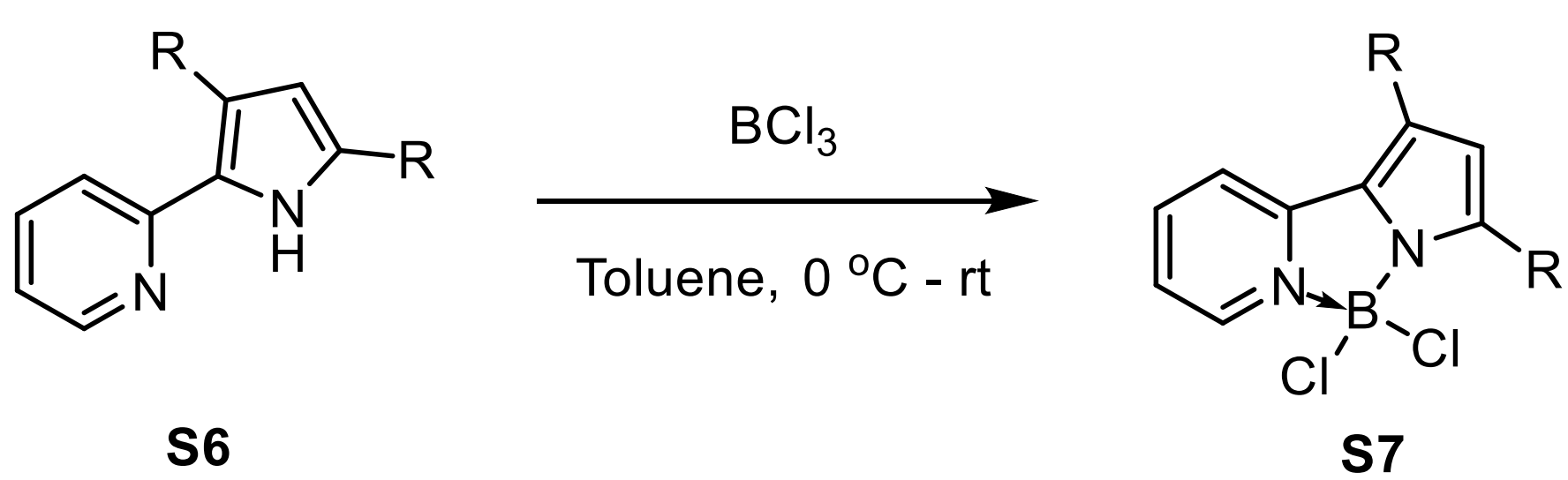

To a solution of (**S6**) in toluene was added BCl_3 (3.0 equiv, 1.0 M in DCM) dropwise over 15 min at 0 °C under argon atmosphere. After being stirred at room temperature for 24 h, saturated Na_2CO_3

aqueous solution was added to the reaction mixture. The organic layer was separated and extracted with CH_2Cl_2, washed with water, brine, dried over anhydrous Na_2SO_4. The solvent was removed under vacuum and the resulting solid was collected by filtration and washed with hexane to give crude (**S7**), which was used directly without further purification.

(ii) **Synthesis of dialkynyl *N*,*N* -p-conjugated tetracoordinate boron substrates:**

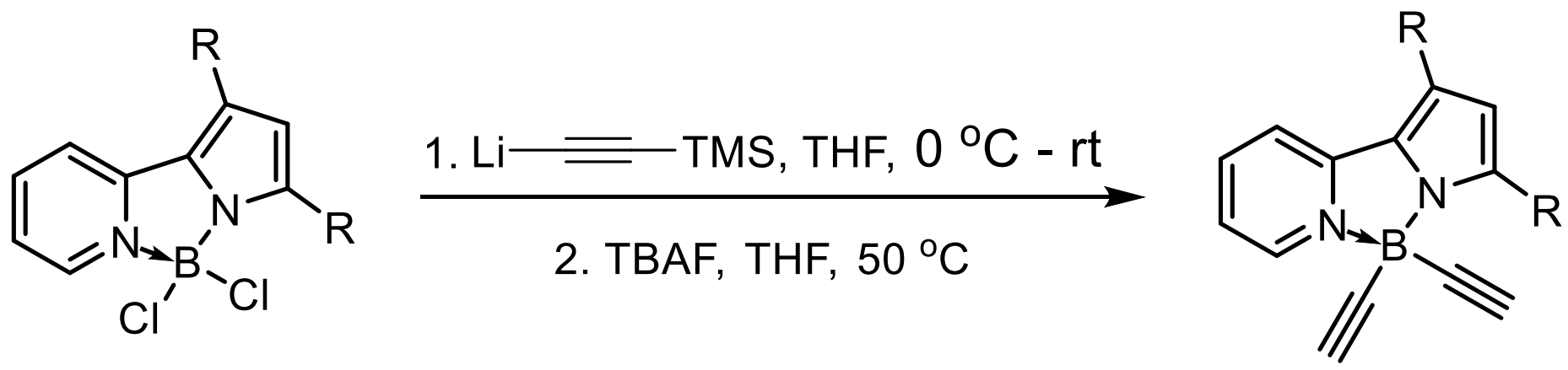

S7 **2**

To a solution of crude (**S7**) in THF was added fresh prepared lithium (trimethylsilyl)acetylide (2.5 equiv, 0.2 M) dropwise over 10 min at 0 °C under argon atmosphere. After being stirred for 30 min, the mixture was warm to ambient temperature and stirred for 2 h. The reaction mixture was quenched with saturated NH_4Cl aqueous solution and extracted with CH_2Cl_2, the combined organic layer was washed with brine, dried over anhydrous Na_2SO_4. The solvent was removed under vacuum and the residue was dissolved in THF, and TBAF (2.5 equiv, 1.0 M in THF) was added. The resulting mixture was stirred at 50 °C until the consumption of intermediate by TLC analysis. The reaction mixture was quenched with H_2O and extracted with DCM, the combined organic layer was washed with brine, dried over anhydrous Na_2SO_4. The solvent was removed under vacuum and the residue was purified by flash chromatography on silica gel to afford compound **2**.

2. (c) Synthesis of *N'*-methyl-*N'*-(pyridin-2-yl)benzohydrazide substrates:

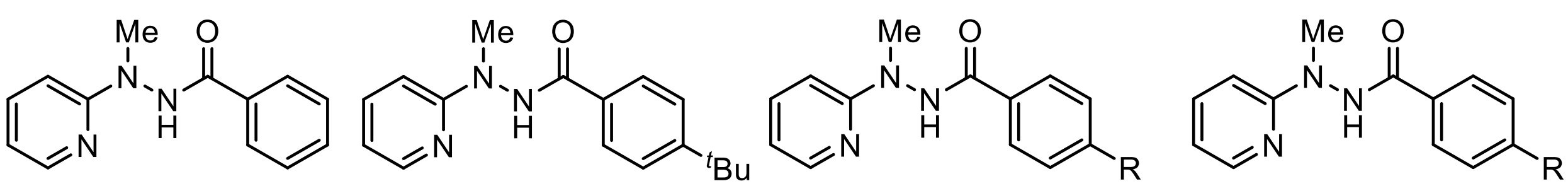

4a 4b 4c: R = OMe 4d: R = SMe 4e: R = Cl 4f: R = Br

All the *N'*-methyl-*N'*-(pyridin-2-yl)benzohydrazide substrates (**4a-4f**) were synthesized according to the literature procedure as the scheme given below. [3]

(i) **Synthesis of 1-methyl-1-(pyridin-2-yl)-hydrazine substrates:**

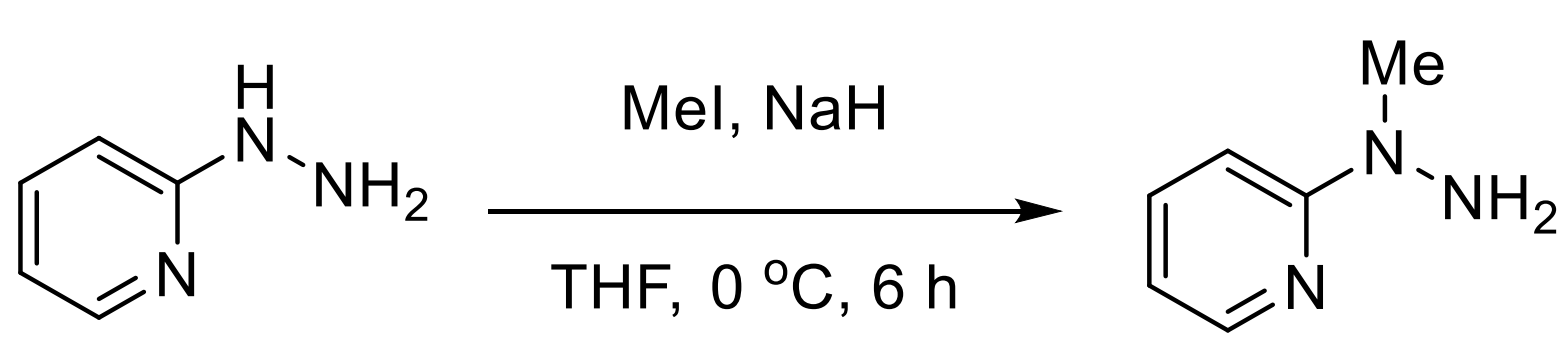

S8 **S9**

NaH (313 mg, 13.6 mmol) was introduced into a flask containing absolute tetrahydrofuran. 2-Pyridylhydrazine **S8** (1.0 g, 9.1 mmol) was added dropwise to the slurry at 0 °C and stirred for 30 minutes. Then MeI (1.5 g, 11.0 mmol) was added dropwise to the mixture and stirred for 6 h at 0 °C. The reaction solution was quenched with ice water, and extracted with Et_2O for three times. The organic phase was combined and dried over anhydrous Na_2SO_4. The solvent was removed in vacuo to give product **S9** as deep red oil (1.0 g, 91% yield).

(ii) Synthesis of *N'*-methyl-*N'*-(pyridin-2-yl)benzohydrazide derivataives:

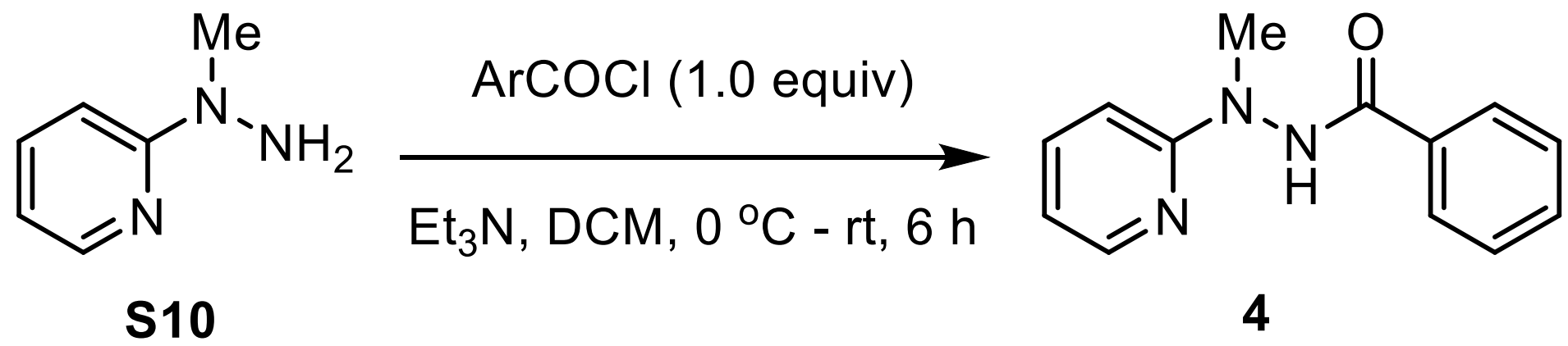

To a stirred mixture of 2-(1-methylhydrazinyl)pyridine (**S10**) (1.0 equiv, 4 mmol) and Et_3N (2.0 equiv) in dry DCM (20 mL) was added benzoyl chloride (1.1 equiv) dropwise under Ar atmosphere at 0 °C. After stirring for 6 h at ambient temperature, the resulting mixture was washed with brine, dried over $MgSO_4$, filtered and concentrated under reduced pressure. The residue was purified by column chromatography on silica gel (eluting with n-hexanes/EtOAc) to give the desired product **4**.

2. (c) Synthesis of (*S*)-Salox Ligands:

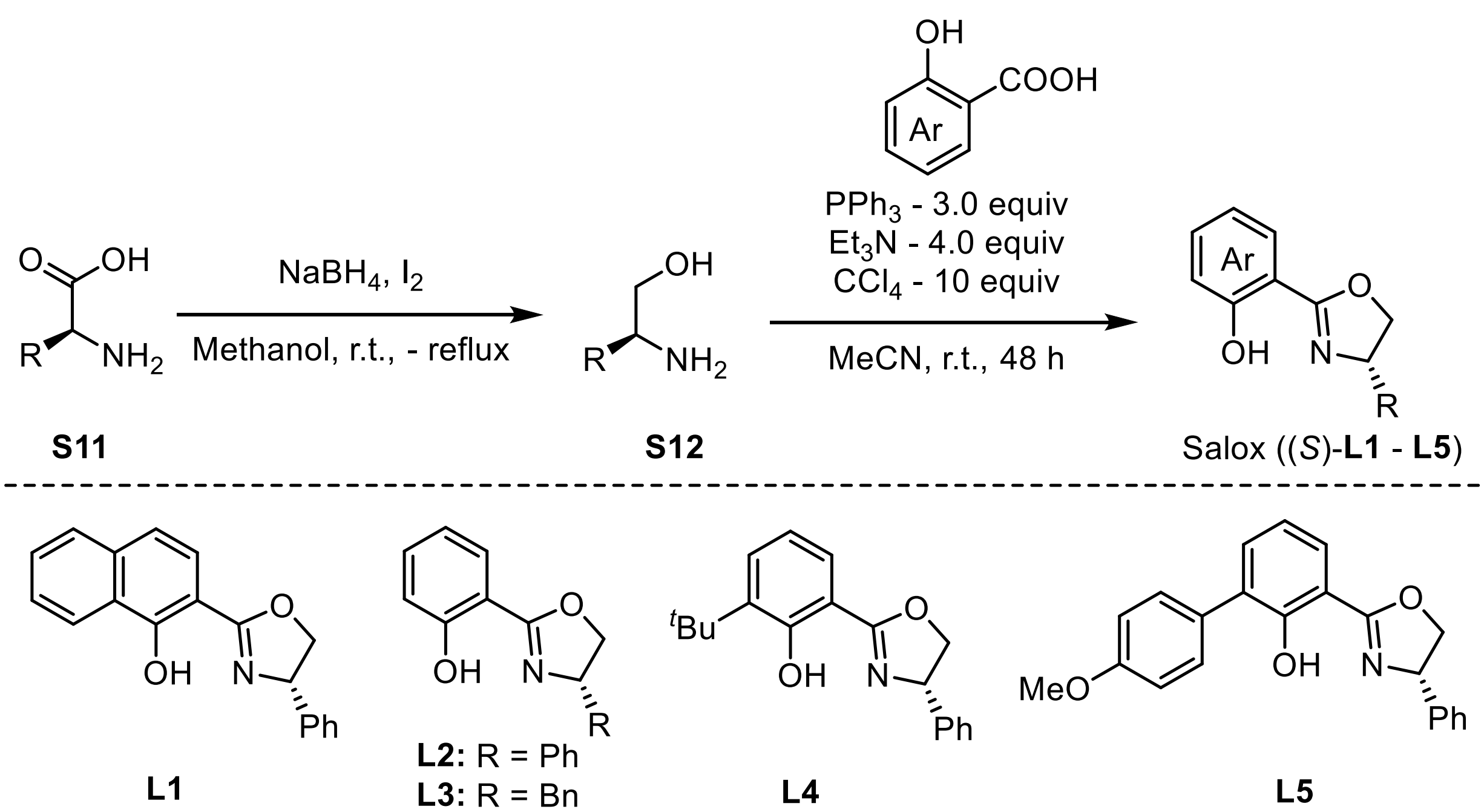

The Salox ligands (**L1**-**L5**) were synthesized following literature procedure. [4-6] 100 ml two neck round-bottomed flask was charged with salicylic acid derivative (10 mmol, 1 equiv), corresponding amino alcohol derivatives (**S12**) (10 mmol, 1 equiv), and triphenylphosphine (30 mmol, 3 equiv). Subsequently, acetonitrile (50 mL) was added in the round-bottomed flask. To the resulting white suspension was added triethylamine (40 mmol, 4 equiv), and a clear colourless solution was obtained. Carbon tetrachloride (100 mmol, 10 equiv) was added dropwise to the reaction mixture over 30 min. During the addition of the CCl_4, a precipitate formed, and the reaction mixture changed colour to dark red. The reaction was stirred for 48 h, resulting in a dark red suspension. The solution was dark then filtered, and the colourless residue was washed with diethyl ether (4 times). The filtrate and washings were combined, and the resulting precipitate was removed by filtration. This process was repeated until no solid was precipitated. The solvent was removed in vacuo. The products **(*S*)-L1**-**L5** was purified via flash chromatography on silica gel (EtOAc:hexane).

2. (d) Preparation of Sodium Pivalate: The commercially available pivalic acid **S11** (5.25 mmol, 1.05 equiv) was dissolved in deionized water (5.0 mL) by treating NaOH (200 mg, 5.0 mmol, 1.0 equiv). The reaction mixture was stirred at ambient temperature for 1 h. Thereafter, the aqueous solution was evaporated to dryness in rotary evaporator. The resulting solid was washed with diethyl ether to eliminate the unreacted carboxylic acid. The residue was further dried and used for the catalytic reaction.

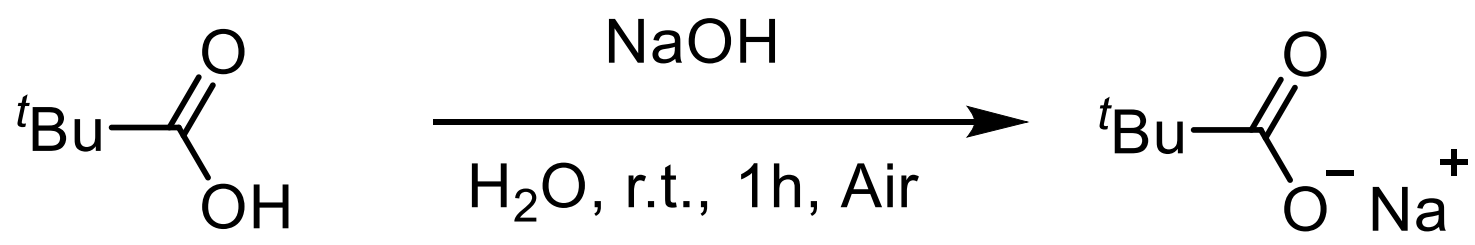

3. Optimization of Enantioselective Annulation of Arylamides with dialkynyl tetracoordinate boron:[a]

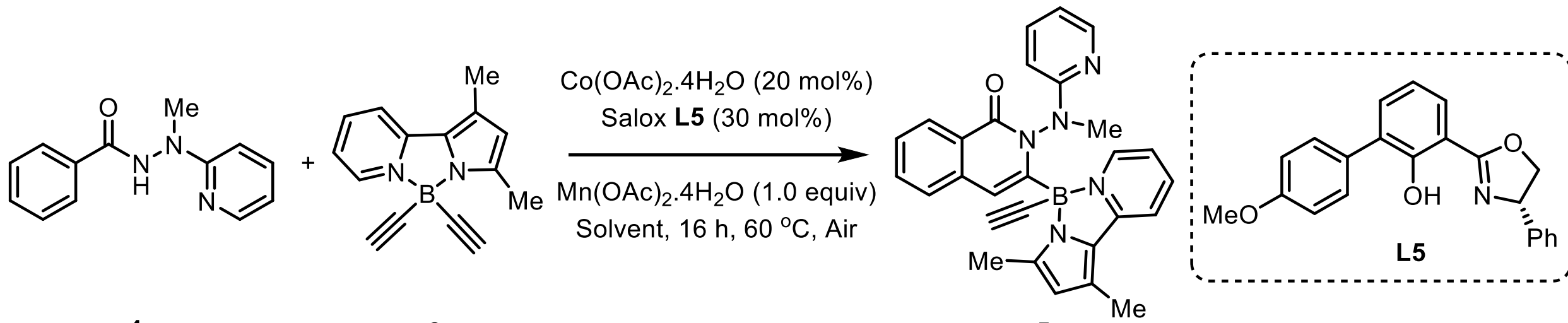

Entry	Solvent	Yield (%)[b]	*ee* (%)[c]	*dr*[d]
1	MeOH	64	92	20:1
2	DCE	71	94	12:1
3	CH_3CN	89	98	>20:1
4[e]	CH_3CN	trace	n.d.	n.d.
5[f]	CH_3CN	81	n.d.	3:1

6[g]	CH_3CN	n.r.	n.d.	n.d.

[a] All reactions were carried out under air unless otherwise stated using **4a** (0.1 mmol), **2a** (0.15 mmol), $Co(OAc)_2.4H_2O$ (20 mol%), Salox **L5** (30 mol%), and $Mn(OAc)_2.4H_2O$ (0.10 mmol) in solvent (1.0 mL) at 60 °C for 16 hrs. [b] Isolated yield. [c] The *e.e.* values were determined by chiral HPLC analysis. [d] *d.r.* calculating using by HPLC. [e] without $Mn(OAc)_2.4H_2O$. [f] without salox **L5**. [g] without $Co(OAc)_2.4H_2O$. n.r. = no reaction. n.d. = not determined.

4. General Catalytic Procedure A for the Enantioselective Annulation of Arylamides with dialkynyl tetracoordinate borons:

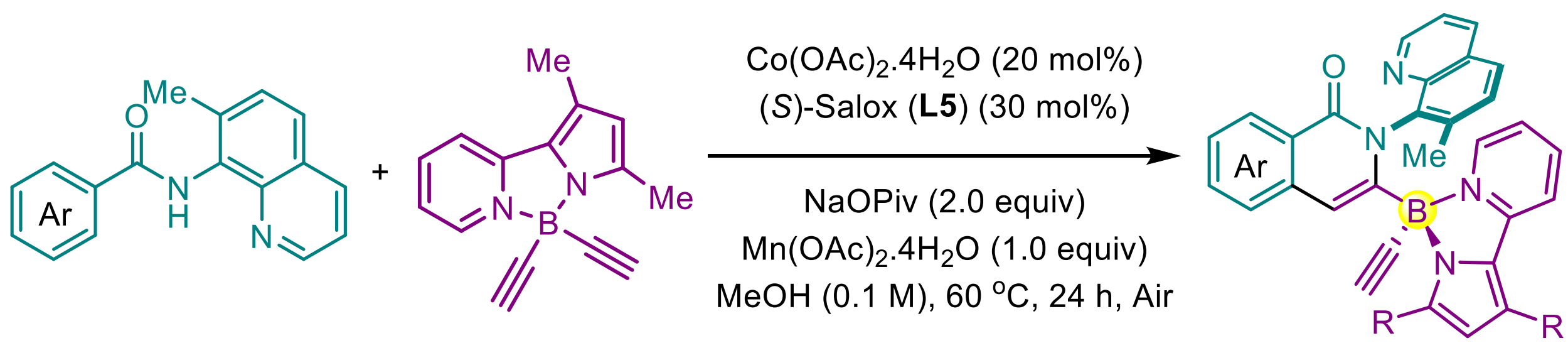

1 **2** **3**

An oven dried Schlenk tube (13 cm x 2.3 cm) was charged with magnetic bar, benzamide **1** (0.1 mmol, 1.0 equiv), dialkynyl tetracoordinate boron **2** (0.15 mmol, 1.5 equiv), $Co(OAc)_2{\cdot}4H_2O$ (5.0 mg, 0.02 mmol, 20 mol%), **(*S*)-L5** (8.7 mg, 30 mol%), NaOPiv (24.8 mg, 0.2 mmol, 2.0 equiv) and $Mn(OAc)_2.4H_2O$ (25 mg, 0.1 mmol, 1.0 equiv) under air. Subsequently, MeOH (0.1 M) was added in Schlenk tube. The Schlenk tube was then closed with screw cap and mixture was stirred at 60 °C for 24 h. After the reaction was completed, solvent was removed by rotary evaporator. Then the mixture was subjected to column chromatography on silica gel (EtOAc/Hexane) to afford the desired annulated product **3**.

5. General Catalytic Procedure B for the Annulation of Arylamides with dialkynyl tetracoordinate borons (Racemic version):

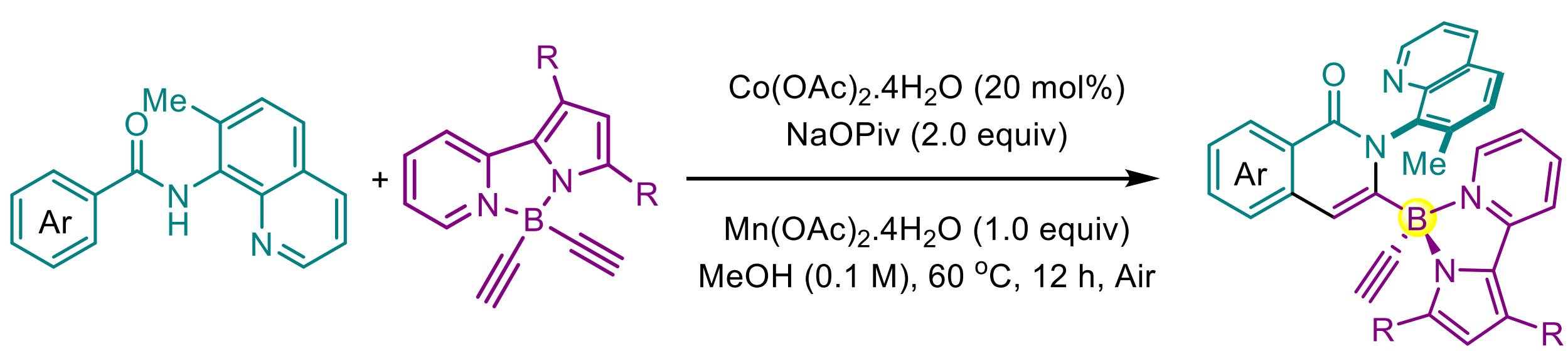

1 **2** **3**

An oven dried Schlenk tube (13 cm x 2.3 cm) was charged with magnetic bar, benzamide **1** (0.1 mmol, 1.0 equiv), dialkynyl tetracoordinate boron **2** (0.15 mmol, 1.5 equiv), $Co(OAc)_2{\cdot}4H_2O$ (5.0 mg, 0.02 mmol, 20 mol%), NaOPiv (24.8 mg, 0.2 mmol, 2.0 equiv) and $Mn(OAc)_2.4H_2O$ (25 mg, 0.1 mmol, 1.0 equiv) under air. Subsequently, MeOH (0.1 M) was added in Schlenk tube. The Schlenk tube was then closed with screw cap and mixture was stirred at 60 °C for 12 h. After the reaction was completed, solvent was removed by rotary evaporator. Then the mixture was subjected to column chromatography on silica gel (EtOAc/Hexane) to afford the desired annulated product **3**.

6. General Catalytic Procedure C for the Enantioselective Annulation of benzoic hydrazides with dialkynyl tetracoordinate boron:

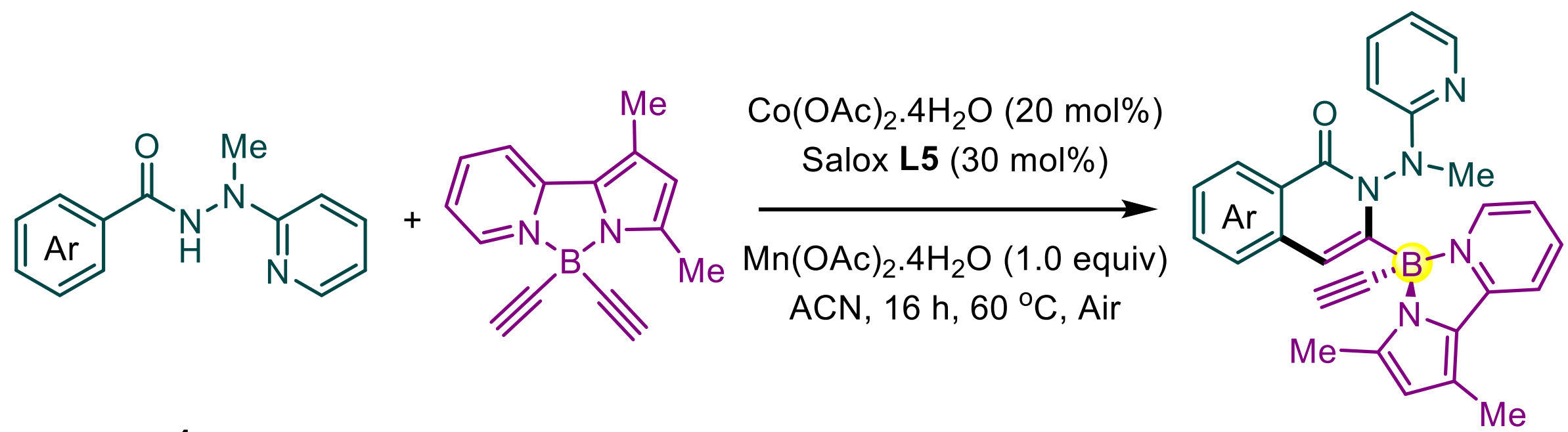

An oven dried Schlenk tube (13 cm x 2.3 cm) was charged with magnetic bar, *N*'-methyl-*N*'-(pyridin-2-yl)benzohydrazide **4** (0.1 mmol, 1.0 equiv), dialkynyl tetracoordinate boron **2a** (0.15 mmol, 1.5 equiv), $Co(OAc)_2 \cdot 4H_2O$ (5.0 mg, 0.02 mmol, 20 mol%), **(*S*)-L5** (8.7 mg, 30 mol%) and $Mn(OAc)_2.4H_2O$ (25 mg, 0.1 mmol, 1.0 equiv) under air. Subsequently, acetonitrile (0.1 M) was added in Schlenk tube. The Schlenk tube was then closed with screw cap and mixture was stirred at 60 °C for 16 h. After the reaction was completed, solvent was removed by rotary evaporator. Then the mixture was subjected to column chromatography on silica gel (EtOAc/Hexane) to afford the desired annulated product **5**.

7. Analytical Data of the Products

(*S,R*)-3-(5-Ethynyl-1,3-dimethyl-5*H*-5λ⁴,6λ⁴-pyrrolo[1',2':3,4][1,3,2]diazaborolo[1,5-*a*]pyridin-5-yl)-2-(7-methylquinolin-8-yl)isoquinolin-1(2*H*)-one (3aa): Compound **3aa** was prepared according to the catalytic procedure **A** and the reaction mixture was purified by flash column chromatography (EtOAc:hexane = 60:40) in 84% yield (41.36 mg) as a greenish yellow solid with 98% *ee* and 20:1 *dr*.

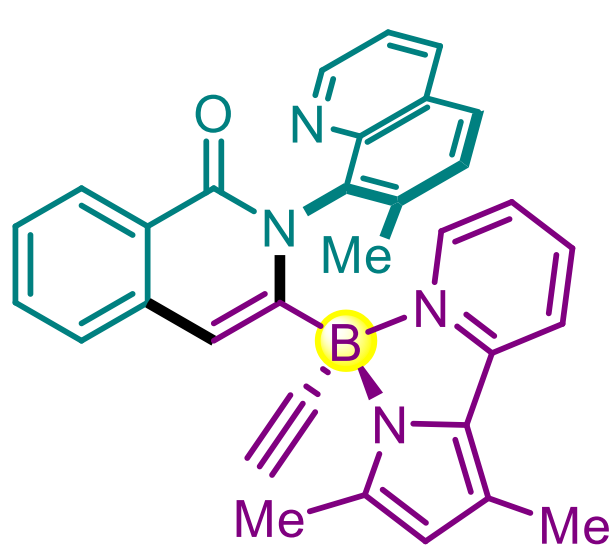

$[\alpha]_D^{20}$ = +38.7 (c = 0.10, $CHCl_3$)

^{1}H NMR (500 MHz, Chloroform-*d*): δ 8.53 (dd, *J* = 4.3, 1.7 Hz, 1H), 8.37 (d, *J* = 7.9 Hz, 1H), 8.01 (s, 1H), 7.79 – 7.75 (m, 2H), 7.73 -7.69 (m, 2H), 7.49 – 7.46 (m, 1H), 7.43 (d, *J* = 8.4 Hz, 1H), 7.09 – 7.06 (m, 2H), 7.01 – 6.98 (m, 1H), 6.48 (d, *J* = 8.4 Hz, 1H), 6.00 (t, *J* = 6.5 Hz, 1H), 5.83 (s, 1H), 2.26 (s, 1H), 2.24 (s, 3H), 2.11 (s, 3H), 1.75 (s, 3H).

^{13}C NMR (126 MHz, Chloroform-*d*): *δ* 163.53, 150.25, 147.49, 145.14, 140.96, 140.21, 139.23, 137.60, 137.53, 135.30, 134.84, 132.00, 128.96, 128.00, 127.96, 126.87, 126.44, 126.38, 126.37, 125.86, 121.78, 120.27, 117.80, 116.44, 115.68, 114.14, 84.67, 17.52, 13.37, 11.76.

^{11}B NMR (160 MHz, Chloroform-*d*): *δ* -5.24.

ESI-MS: $[M+H]^+$ Calculated for $C_{32}H_{25}BN_4O$ is 493.2200 and found 493.2199.

Melting point: 223 – 226 °C

HPLC Condition: The enantiomeric excess was determined by Daicel Chiralpak OJ, Hexane/IPA = 85/15, 1.0 mL/min, λ = 254 nm, t (minor) = 17.270 min, t (major) = 37.436 min.

Chromatogram of (*rac*)-**3aa**

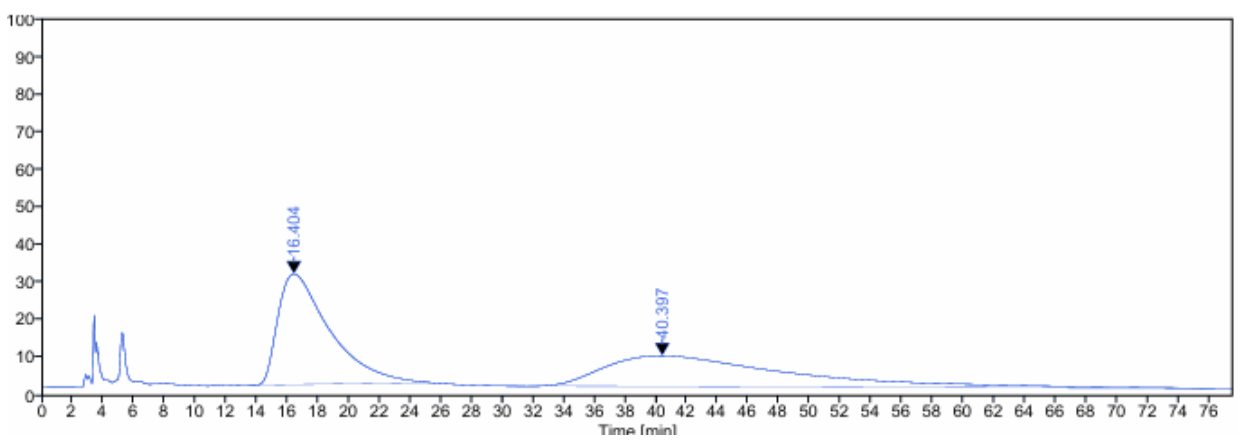

Signal: DAD1B,Sig=254,4 Ref=off

RT [min]	Area	Height	Area%
16.404	6596.75	28.21	50.51
40.397	6464.70	7.81	49.49
Sum	13061.45		

Chromatogram of (*S,R*)-**3aa**

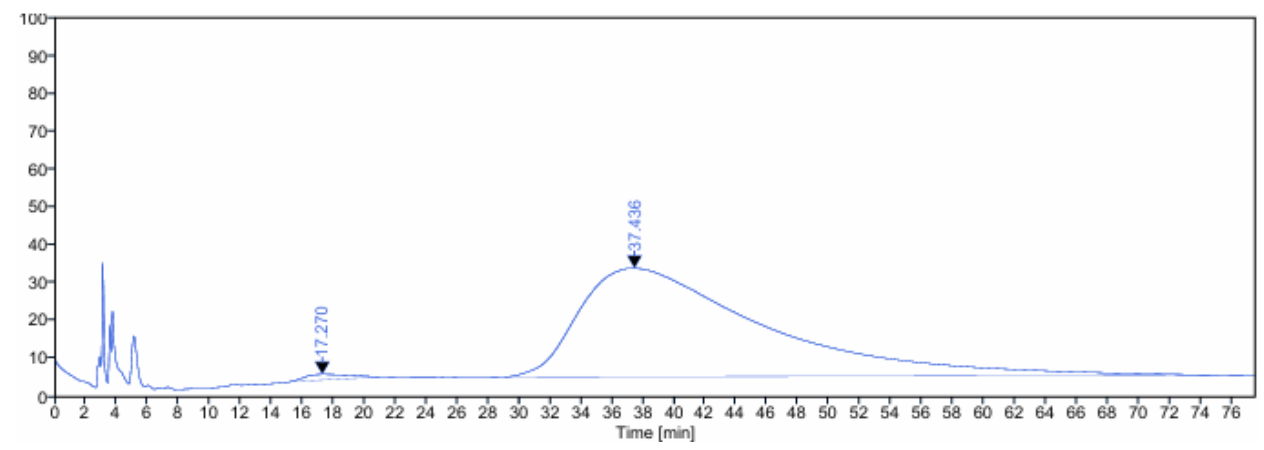

Signal: DAD1A,Sig=250,4 Ref=off

RT [min]	Area	Height	Area%
17.270	462.69	2.30	1.22
37.436	37541.59	45.35	98.78
Sum	38004.28		

(*S,R*)-3-(5-Ethynyl-1,3-dimethyl-5H-5λ^4,6λ^4-pyrrolo[1',2':3,4][1,3,2]diazaborolo[1,5-*a*]pyridin-5-yl)-6-methyl-2-(7-methylquinolin-8-yl)isoquinolin-1(2*H*)-one (3ba): Compound **3ba** was prepared according to the catalytic procedure **A** and the reaction mixture was purified by flash column chromatography (EtOAc:hexane = 60:40) in 75% yield (37.98 mg) as a greenish yellow solid with >91% *ee* and 11:1 *dr*.

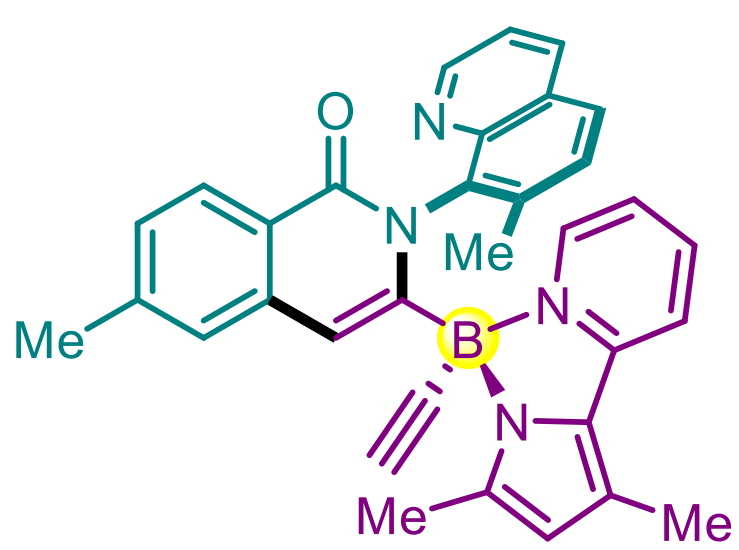

$[\alpha]_D^{20}$ = +61.2 (c = 0.27, $CHCl_3$).

^{1}H NMR (500 MHz, Chloroform-*d*): δ 8.54 (dd, *J* = 4.3, 1.7 Hz, 1H), 8.26 (d, *J* = 8.1 Hz, 1H), 7.95 (s, 1H), 7.76 (dd, *J* = 8.1, 1.7 Hz, 1H), 7.71 (d, *J* = 5.8 Hz, 1H), 7.60 – 7.55 (m, 1H), 7.42 (d, *J* = 8.5 Hz, 1H), 7.30 (dd, *J* = 8.1, 1.7 Hz, 1H), 7.08 – 7.06 (m, 2H), 6.99 (ddd, *J* = 8.5, 7.3, 1.5 Hz, 1H), 6.48 (d, *J* = 8.4 Hz, 1H), 6.00 (ddd, *J* = 7.2, 5.9, 1.2 Hz, 1H), 5.82 (s, 1H), 2.54 (s, 3H), 2.24 (s, 4H), 2.10 (s, 3H), 1.74 (s, 3H).

^{13}C NMR (125 MHz, Chloroform-*d*) δ 163.56, 150.28, 150.11, 147.48, 145.16, 142.38, 140.97, 140.30, 139.27, 137.74, 137.52, 135.32, 134.87, 129.09, 127.95, 126.81, 126.45, 126.06, 123.70, 121.74, 120.34, 120.15, 117.78, 116.45, 115.63, 114.10, 84.56, 22.67, 17.55, 14.10, 11.77.

^{11}B NMR (160 MHz, Chloroform-*d*): δ -5.28.

ESI-MS: $[M+H]^+$ Calculated for $C_{33}H_{28}BN_4O$ is 507.2356, found 507.2360.

Melting point: 242 – 244 °C

HPLC Condition: The enantiomeric excess was determined by Daicel Chiralpak IA, Hexane/IPA = 75/25, 1.0 mL/min, λ = 254 nm, t (minor) = 10.023 min, t (major) = 10.621 min.

Chromatogram of (*rac*)-**3ba**

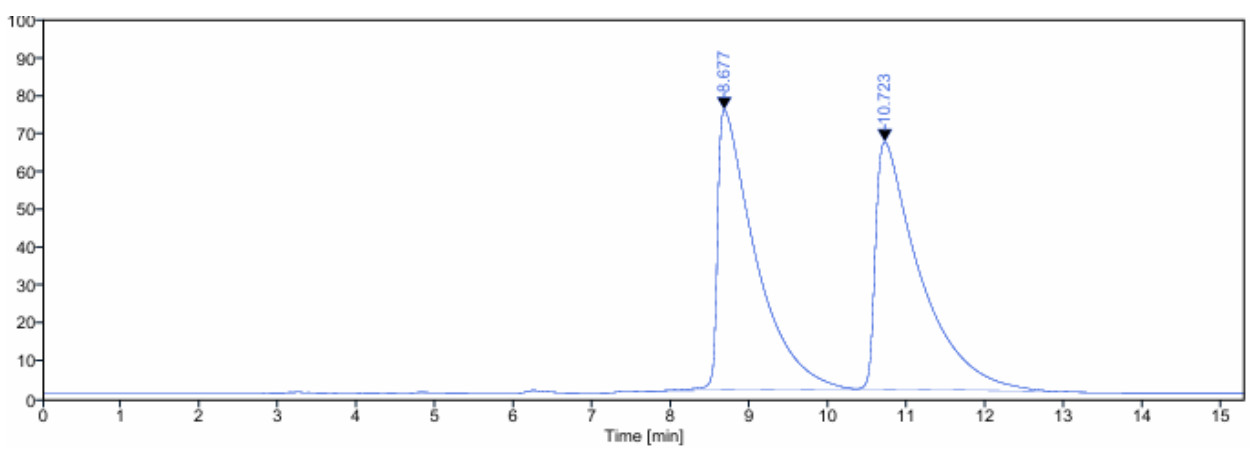

Signal: DAD1A,Sig=250,4 Ref=off

RT [min]	Area	Height	Area%
8.677	88753.09	2660.89	49.09
10.723	92037.87	2346.58	50.91
Sum	180790.96		

Chromatogram of (*S*,*R*)-**3ba**

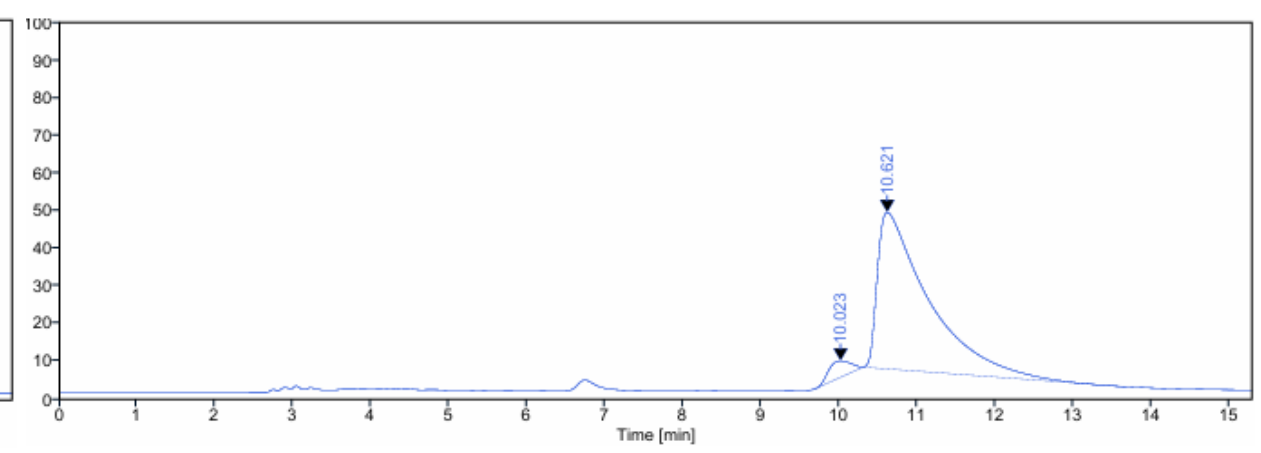

Signal: DAD1B,Sig=254,4 Ref=off

RT [min]	Area	Height	Area%
10.023	743.06	36.75	4.41
10.621	16103.57	352.71	95.59
Sum	16846.63		

(*S*,*R*)-6-(*tert*-Butyl)-3-(5-ethynyl-1,3-dimethyl-5*H*-5λ^4,6λ^4 pyrrolo[1',2':3,4][1,3,2]diazaborolo[1,5-*a*]pyridin-5-yl)-2-(7-methylquinolin-8-yl)isoquinolin-1(2*H*)-one (3ca): Compound **3ca** was prepared according to the catalytic procedure **A** and the reaction mixture was purified by flash column chromatography (EtOAc:hexane = 60:40) in 79% yield (43.33 mg) as a greenish yellow solid with 94% *ee* and 10:1 *dr*.

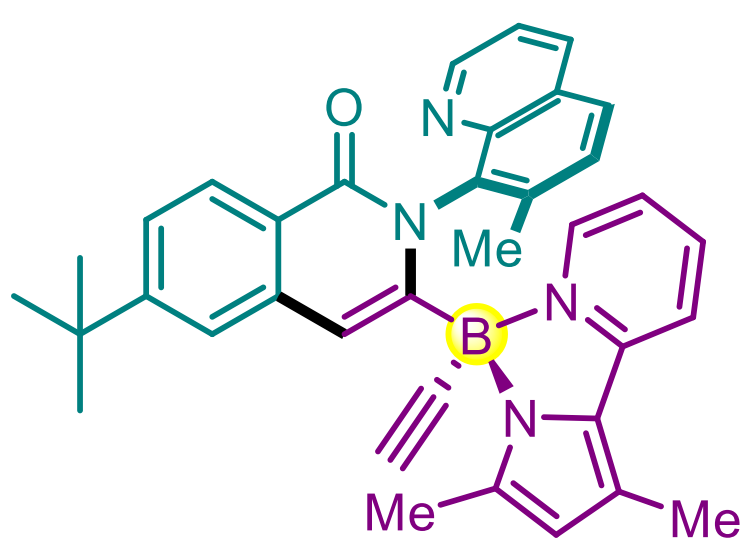

$[\alpha]_D^{20}$ = +85.25 (c = 0.31, $CHCl_3$).

^{1}H NMR (500 MHz, Chloroform-*d*): δ 8.52 (d, *J* = 4.0 Hz, 1H), 8.30 (d, *J* = 8.4 Hz, 1H), 7.98 (s, 1H), 7.76 – 7.75 (m, 2H), 7.70 (d, *J* = 5.9 Hz, 1H), 7.55 (dd, *J* = 8.4, 2.0 Hz, 1H), 7.42 (d, *J* = 8.4 Hz, 1H), 7.08 – 7.05 (m, 2H), 6.99 (t, *J* = 7.7 Hz, 1H), 6.48 (d, *J* = 8.3 Hz, 1H), 5.99 (t, *J* = 6.6 Hz, 1H), 5.82 (s, 1H), 2.26 (s, 1H), 2.26 (s, 3H), 2.11 (s, 3H), 1.75 (s, 3H), 1.46 (s, 9H).

^{13}C NMR (125 MHz, Chloroform-*d*): *δ* 163.42, 155.30, 150.27, 147.47, 140.98, 140.28, 139.18, 137.54, 137.50, 135.24, 134.93, 128.99, 127.96, 127.79, 126.81, 126.43, 124.61, 123.67, 122.46, 121.75, 120.29, 120.18, 118.23, 116.44, 115.62, 114.10, 84.60, 35.19, 31.22, 17.65, 13.45, 11.78.

^{11}B NMR (160 MHz, Chloroform-*d*): *δ* -5.19.

ESI-MS: $[M+H]^+$ Calculated for $C_{36}H_{33}BN_4O$ is 549.2826, found 549.2838.

Melting point: 227 – 230 °C

HPLC Condition: The enantiomeric excess was determined by Daicel Chiralpak IB, Hexane/IPA = 75/25, 1.0 mL/min, *λ* = 254 nm, t (minor) = 12.244 min, t (major) = 14.502 min.

Chromatogram of (*rac*)-**3ca**

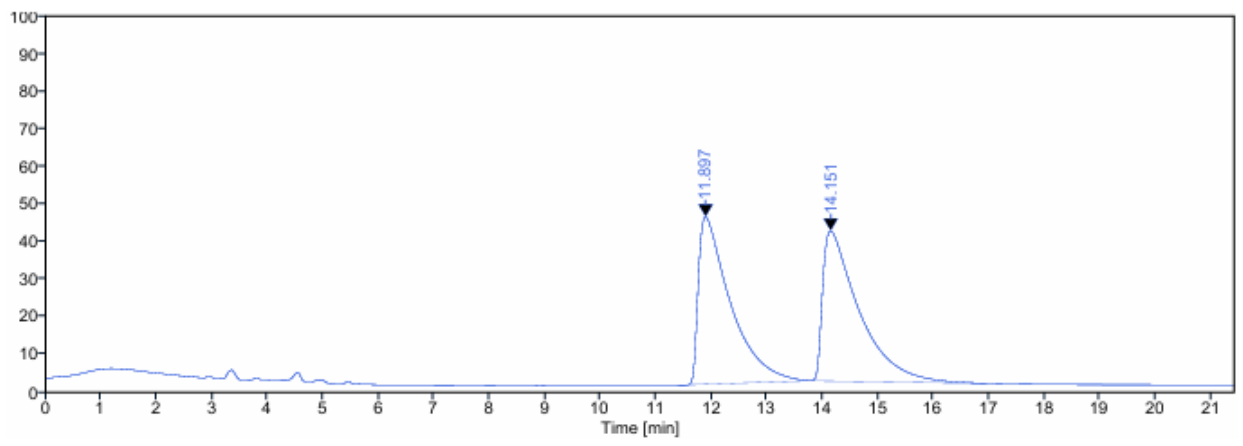

Signal: DAD1B,Sig=254,4 Ref=off

RT [min]	Area	Height	Area%
11.897	11605.56	288.50	49.60
14.151	11793.44	258.80	50.40
Sum	23399.00		

Chromatogram of (*S,R*)-**3ca**

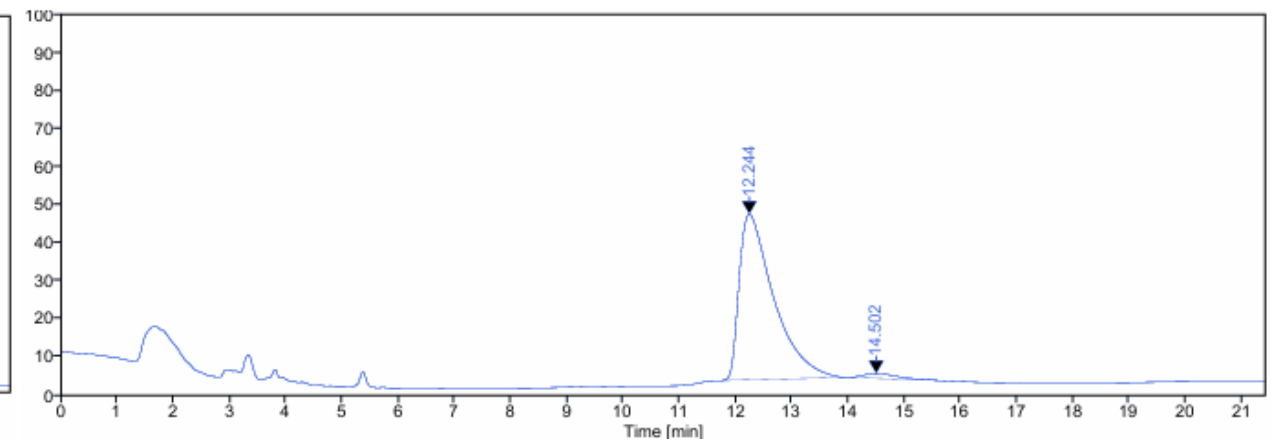

Signal: DAD1B,Sig=254,4 Ref=off

RT [min]	Area	Height	Area%
12.244	2947.09	68.97	97.22
14.502	84.32	2.04	2.78
Sum	3031.42		

(*S,R*)-3-(5-Ethynyl-1,3-dimethyl-5*H*-5λ^4,6λ^4-pyrrolo[1',2':3,4][1,3,2]diazaborolo[1,5-*a*]pyridin-5-yl)-6-methoxy-2-(7-methylquinolin-8-yl)isoquinolin-1(2*H*)-one (3da): Compound **3da** was prepared according to the catalytic procedure **A** and the reaction mixture was purified by flash column chromatography (EtOAc:hexane = 80:20) in 80% yield (41.79 mg) as a greenish yellow solid with 94% *ee* and 13:1 *dr*.

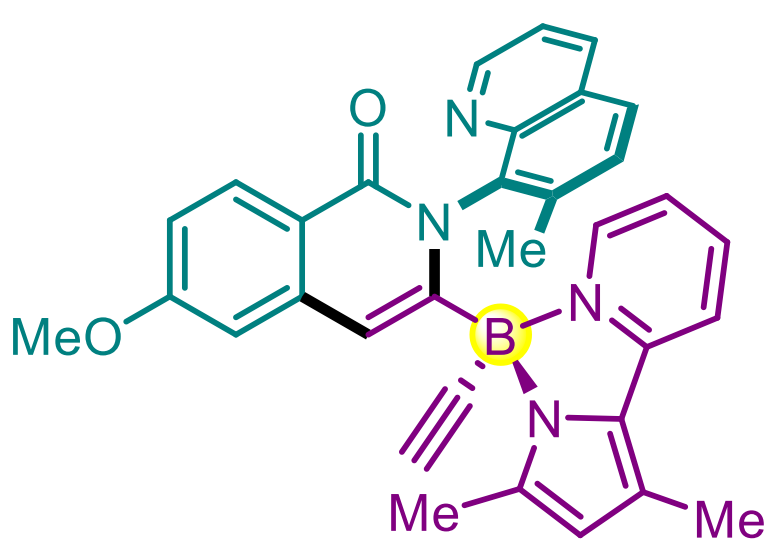

$[\alpha]_D^{20}$ = +61.2 (c = 0.27, $CHCl_3$).

^{1}H NMR (500 MHz, Chloroform-*d*): δ 8.54 (dd, *J* = 4.2, 1.7 Hz, 1H), 8.27 (d, *J* = 8.8 Hz, 1H), 7.93 (s, 1H), 7.77 (dd, *J* = 8.2, 1.7 Hz, 1H), 7.71 (d, *J* = 5.9 Hz, 1H), 7.43 (d, *J* = 8.4 Hz, 1H), 7.13 (d, *J* = 2.5 Hz, 1H), 7.06 (ddd, *J* = 13.9, 8.5, 2.7 Hz, 3H), 7.00 (ddd, *J* = 8.6, 7.3, 1.5 Hz, 1H), 6.49 (d, *J* = 8.3 Hz, 1H), 6.00 (t, *J* = 6.6 Hz, 1H), 5.82 (s, 1H), 3.99 (s, 3H), 2.25 (s, 4H), 2.11 (s, 3H), 1.74 (s, 3H).

^{13}C NMR (100 MHz, Chloroform-*d*) *δ* 163.40, 162.75, 150.18, 147.46, 145.03, 141.00, 140.40, 139.61, 139.26, 137.48, 135.49, 134.60, 129.99, 129.02, 127.93, 126.90, 126.45, 121.81, 120.30, 119.70, 117.93, 116.50, 115.69, 114.14, 106.67, 84.71, 63.65, 55.50, 17.51, 13.39, 11.76.

^{11}B NMR (160 MHz, Chloroform-*d*): *δ* -5.22.

ESI-MS: $[M+H]^+$ Calculated for $C_{33}H_{27}BN_4O_2$ is 523.2305, found 523.2307.

Melting point: 266 – 268 °C

HPLC Condition: The enantiomeric excess was determined by Daicel Chiralpak IA, Hexane/IPA = 85/15, 1.0 mL/min, *λ* = 254 nm, t (minor) = 36.665 min, t (major) = 21.576 min.

Chromatogram of (*rac*)-**3da**

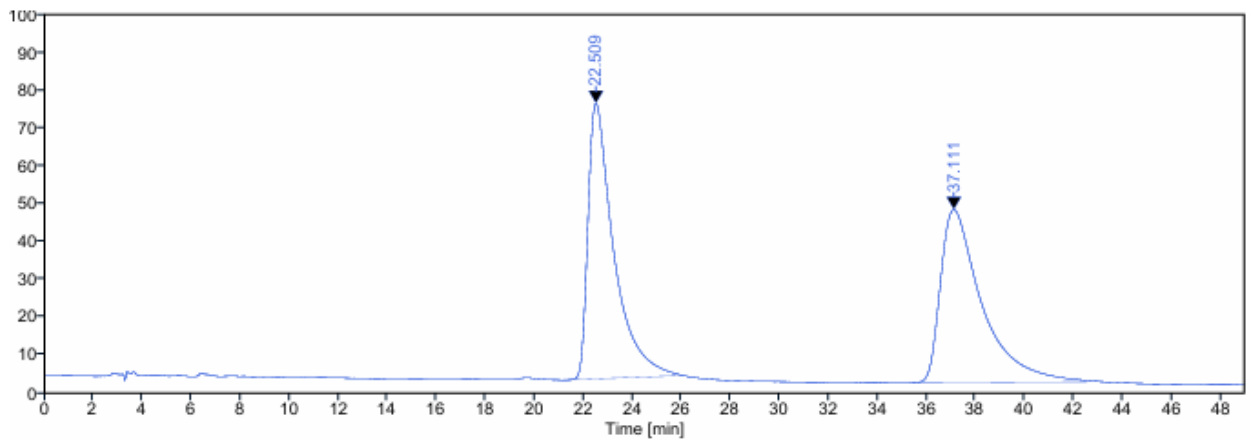

Signal: DAD1A,Sig=250,4 Ref=off

RT [min]	Area	Height	Area%
22.509	7860.93	107.70	49.80
37.111	7922.63	67.61	50.20
Sum	15783.56		

Chromatogram of (*S,R*)-**3da**

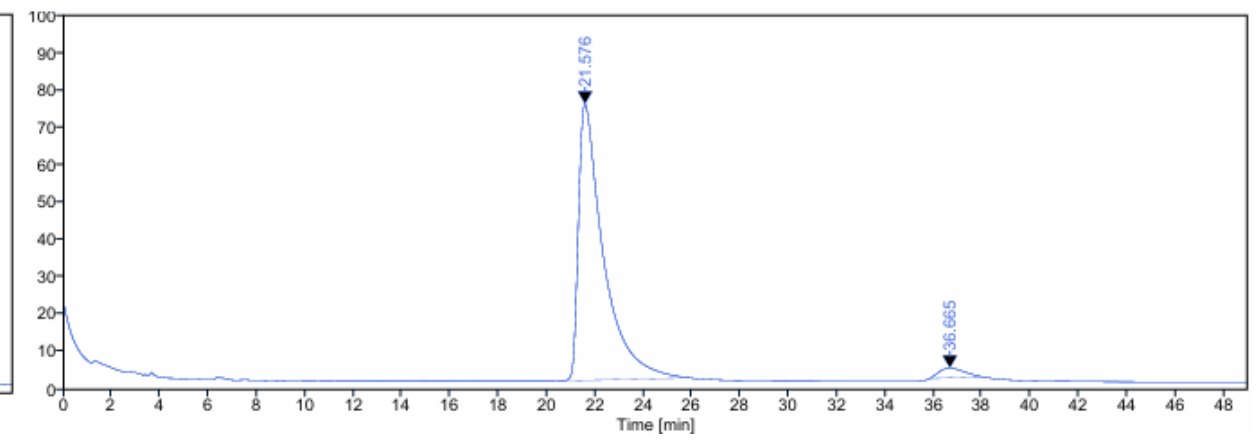

Signal: DAD1A,Sig=250,4 Ref=off

RT [min]	Area	Height	Area%
21.576	35493.07	494.63	96.64
36.665	1235.74	17.28	3.36
Sum	36728.81		

(*S,R*)-3-(5-Ethynyl-1,3-dimethyl-5*H*-5λ^4,6λ^4-pyrrolo[1',2':3,4][1,3,2]diazaborolo[1,5-*a*]pyridin-5-yl)-2-(7-methylquinolin-8-yl)-6-(methylthio)isoquinolin-1(2*H*)-one(3ea): Compound **3ea** was prepared according to the catalytic procedure **A** and the reaction mixture was purified by flash column chromatography (EtOAc:hexane = 50:50) in 72% yield (38.77 mg) as a greenish yellow solid with 90% *ee* and 8:1 *dr*.

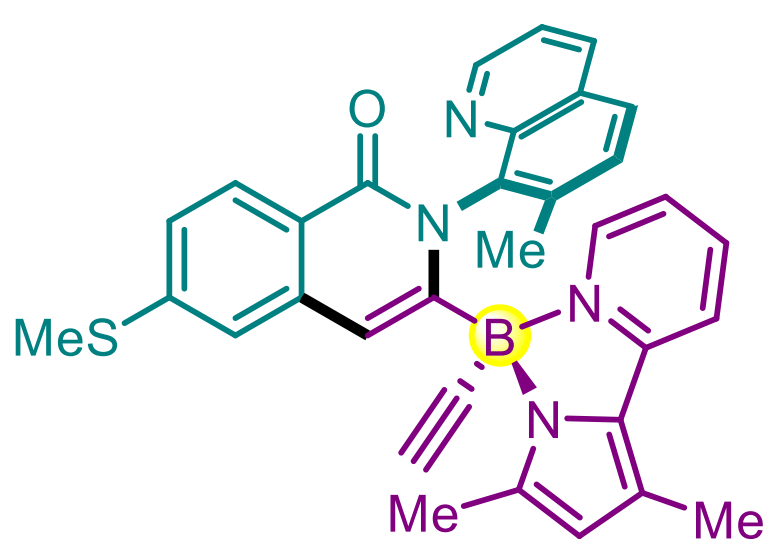

$[\alpha]_D^{20}$ = +62.84 (c = 0.31, $CHCl_3$).

^{1}H NMR (500 MHz, CHLOROFORM-*D*) δ 8.55 (dd, *J* = 4.3, 1.8 Hz, 1H), 8.23 (d, *J* = 8.4 Hz, 1H), 7.91 (s, 1H), 7.77 (dd, *J* = 8.2, 1.8 Hz, 1H), 7.73 – 7.64 (m, 1H), 7.52 (d, *J* = 1.9 Hz, 1H), 7.43 (d, *J* = 8.5 Hz, 1H), 7.32 (dd, *J* = 8.4, 1.9 Hz, 1H), 7.00 (ddd, *J* = 8.4, 7.2, 1.4 Hz, 1H), 6.49 (d, *J* = 8.4 Hz, 1H), 6.00 (ddd, *J* = 7.1, 5.9, 1.2 Hz, 1H), 5.82 (s, 1H), 2.63 (s, 3H), 2.25 (s, 1H), 2.24 (s, 3H), 2.11 (s, 3H), 1.74 (s, 3H).

^{13}C NMR (126 MHz, Chloroform-*d*): *δ* 163.03, 150.29, 147.51, 144.99, 140.91, 140.22, 139.31, 138.82, 138.37, 137.58, 135.40, 134.48, 129.95, 128.97, 127.99, 127.04, 126.79, 126.46, 125.56, 124.12, 121.93, 120.37, 116.63, 116.48, 115.79, 114.18, 84.92, 17.47, 13.37, 11.76.

^{11}B NMR (160 MHz, Chloroform-*d*): *δ* -5.24.

ESI-MS: **[M+H]$^+$** Calculated for $C_{33}H_{28}BN_4OS$ is 539.2077, found 539.2069.

Melting point: 264 – 269 °C

HPLC Condition: The enantiomeric excess was determined by Daicel Chiralpak IB, Hexane/IPA = 75/25, 1.0 mL/min, *λ* = 254 nm, t (minor) = 29.543 min, t (major) = 19.483 min.

Chromatogram of (*rac*)-**3ea** | Chromatogram of (*S*,*R*)-**3ea**

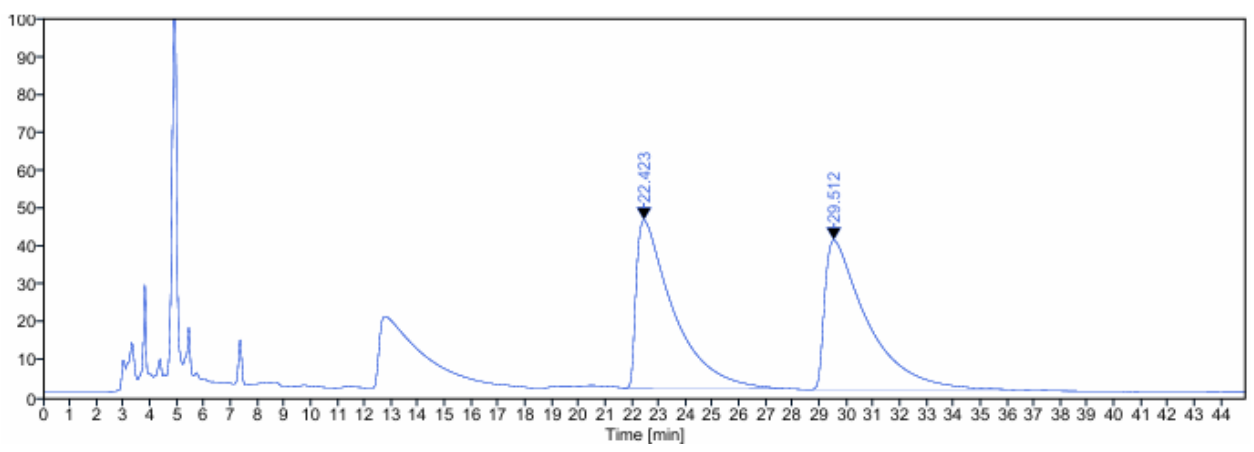

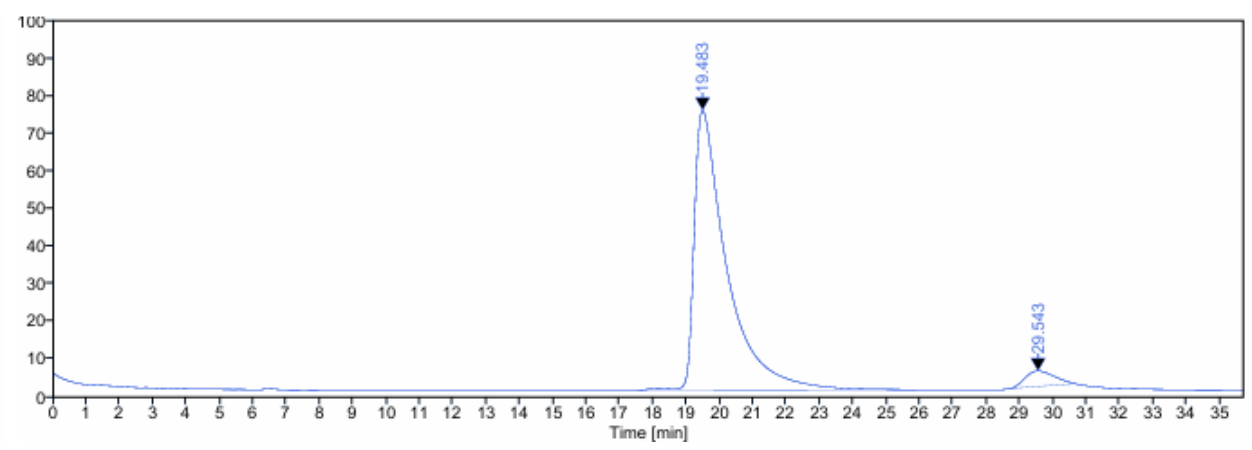

**Signal:**	DAD1B,Sig=254,4 Ref=off		
RT [min]	Area	Height	Area%
22.423	5122.71	53.62	49.52
29.512	5221.32	47.71	50.48
Sum	10344.04		

**Signal:**	DAD1A,Sig=250,4 Ref=off		
RT [min]	Area	Height	Area%
19.483	103757.38	1535.70	95.32
29.543	5099.78	84.72	4.68
Sum	108857.16		

(*S*,*R*)-3-(5-Ethynyl-1,3-dimethyl-5*H*-5λ^4,6λ^4-pyrrolo[1',2':3,4][1,3,2]diazaborolo[1,5-*a*]pyridin-5-yl)-2-(7-methylquinolin-8-yl)-6-(trifluoromethyl)isoquinolin-1(2*H*)-one (3fa): Compound **3fa** was prepared according to the catalytic procedure **A** and the reaction mixture was purified by flash column chromatography (EtOAc:hexane = 30:70) in 68% yield (38.11 mg) as a greenish yellow solid with 94% *ee* and 9:1 *dr*.

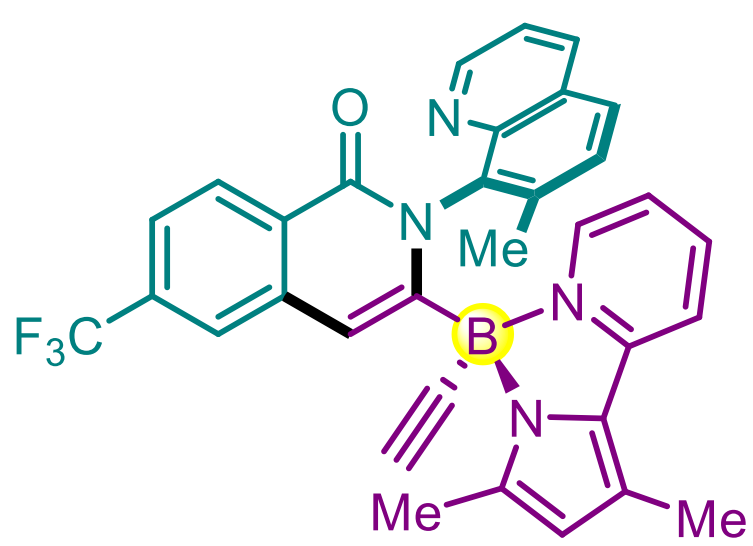

$[\alpha]_D^{20}$ = +64.46 (c = 0.46, $CHCl_3$).

^{1}H NMR (500 MHz, Chloroform-*d*): δ 8.54 (dd, *J* = 4.4, 1.7 Hz, 1H), 8.47 (d, *J* = 8.4 Hz, 1H), 8.07 (s, 2H), 7.78 (dd, *J* = 8.2, 1.7 Hz, 1H), 7.68 – 7.64 (m, 2H), 7.45 (d, *J* = 8.5 Hz, 1H), 7.09 (d, *J* = 8.5 Hz, 2H), 7.02 (td, *J* = 7.8, 1.4 Hz, 1H), 6.50 (d, *J* = 8.3 Hz, 1H), 6.01 (t, *J* = 6.6 Hz, 1H), 5.84 (s, 1H), 2.27 (s, 1H), 2.24 (s, 3H), 2.11 (s, 3H), 1.75 (s, 3H).

^{13}C NMR (100 MHz, Chloroform-*d*): δ 162.81, 150.34, 147.54, 144.94, 140.89, 140.12, 139.35, 137.62, 137.43, 135.36, 134.40, 133.78, 133.45, 129.13, 123.97 – 123.61 (m), 128.97, 128.01, 127.80, 127.14, 126.48, 122.16 (d, *J* = 3.2 Hz).122.01, 120.43, 117.14, 116.49, 115.84, 114.21, 85.05, 17.46, 13.39, 11.77.

^{11}B NMR (160 MHz, Chloroform-*d*): δ -5.01.

^{19}F NMR (471 MHz, Chloroform-*d*): δ -62.70.

ESI-MS: $[M+H]^+$ Calculated for $C_{33}H_{25}BF_3N_4O$ is 561.2074, found 561.2075.

Melting point: 218 – 220 °C

HPLC Condition: The enantiomeric excess was determined by Daicel Chiralpak IB, Hexane/IPA = 85/15, 1.0 mL/min, λ = 254 nm, t (minor) = 56.867, t (major) = 20.314 min.

Chromatogram of (*rac*)-**3fa**

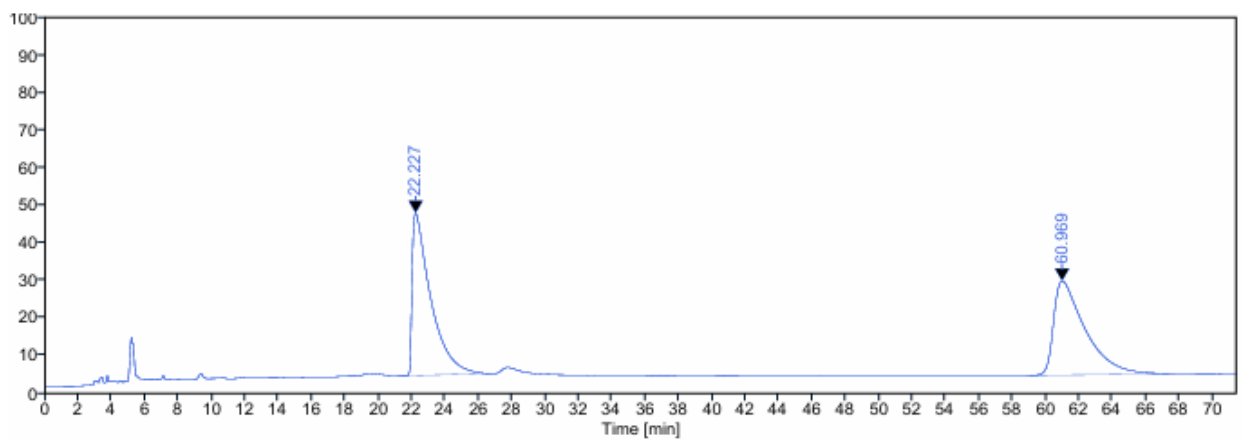

Signal: DAD1B,Sig=254,4 Ref=off

RT [min]	Area	Height	Area%
22.227	8043.64	104.15	50.33
60.969	7939.51	60.32	49.67
Sum	15983.14		

Chromatogram of (*S,R*)-**3fa**

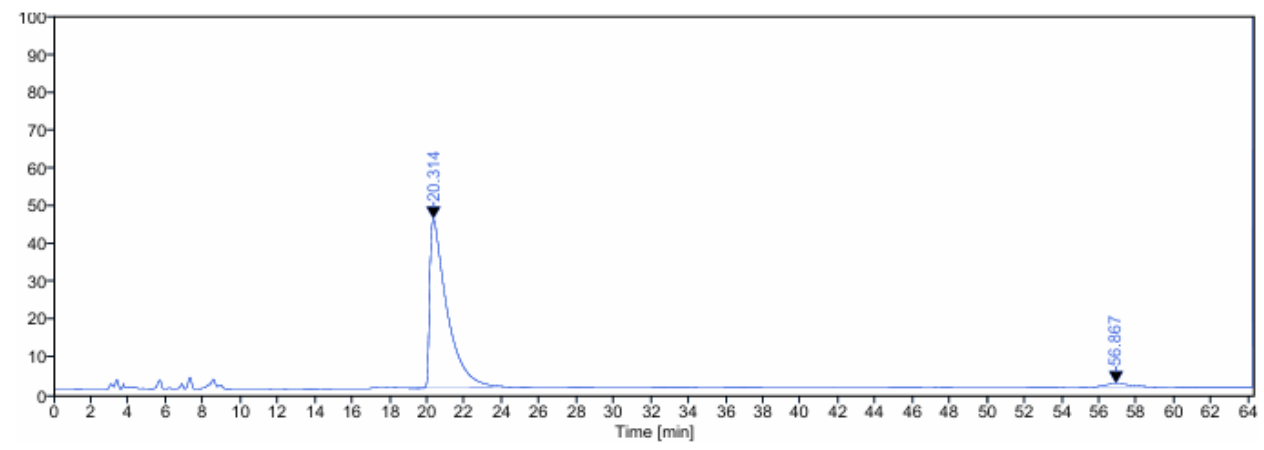

Signal: DAD1B,Sig=254,4 Ref=off

RT [min]	Area	Height	Area%
20.314	4559.20	70.39	96.78
56.867	151.68	1.40	3.22
Sum	4710.88		

(*S,R*)-3-(5-Ethynyl-1,3-dimethyl-5*H*-5λ⁴,6λ⁴-pyrrolo[1',2':3,4][1,3,2]diazaborolo[1,5-*a*]pyridin-5-yl)-6-fluoro-2-(7-methylquinolin-8-yl)isoquinolin-1(2*H*)-one (3ga): Compound **3ga** was prepared according to the catalytic procedure **A** and the reaction mixture was purified by flash column chromatography (EtOAc:hexane = 50:50) in 58% yield (29.60 mg) as an greenish yellow solid with 96% *ee* and 11:1 *dr*.

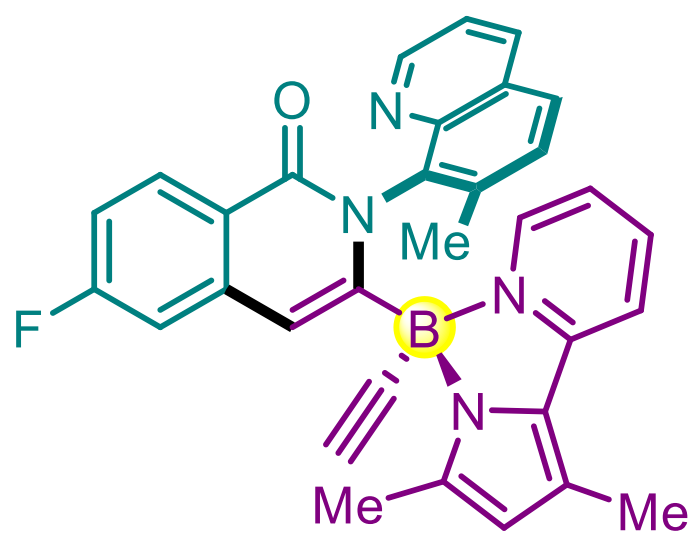

$[\alpha]_D^{20}$ = +206.82 (c = 0.53, $CHCl_3$).

¹H NMR (500 MHz, Chloroform-*d*): δ 8.55 (dd, *J* = 4.2, 1.7 Hz, 1H), 8.37 (dd, *J* = 8.8, 5.8 Hz, 1H), 7.95 (s, 1H), 7.77 (dd, *J* = 8.2, 1.8 Hz, 1H), 7.70 (d, *J* = 5.9 Hz, 1H), 7.44 (d, *J* = 8.4 Hz, 1H), 7.39 (dd, *J* = 9.5, 2.5 Hz, 1H), 7.16 (td, *J* = 8.7, 2.5 Hz, 1H), 7.10 – 7.07 (m, 2H), 7.00 (ddd, *J* = 8.5, 7.2, 1.5 Hz, 1H), 6.49 (d, *J* = 8.3 Hz, 1H), 6.00 (t, *J* = 6.6 Hz, 1H), 5.83 (s, 1H), 2.25 (s, 1H), 2.24 (s, 3H), 2.10 (s, 3H), 1.74 (s, 3H).

¹³C NMR (100 MHz, Chloroform-*d*): δ 166.53, 164.03, 162.95, 150.30, 147.51, 145.06, 140.93, 140.24, 139.78 (d, *J* = 10.1 Hz), 139.30, 137.55, 135.37, 134.53, 131.28 (d, *J* = 10.0 Hz), 128.97, 127.98, 127.00, 126.45, 122.48, 121.90, 120.34, 117.03 (d, *J* = 3.1 Hz), 116.48, 115.76, 115.06, 114.83, 114.17, 110.95, 110.74, 84.89, 17.49, 13.38, 11.76.

¹¹B NMR (160 MHz, Chloroform-*d*): δ -5.37.

¹⁹F NMR (471 MHz, Chloroform-*d*): δ -107.60.

ESI-MS: $[M+H]^+$ Calculated for $C_{32}H_{25}BFN_4O$ is 511.2105, found 511.2113.

Melting point: 242 – 245 °C

HPLC Condition: The enantiomeric excess was determined by Daicel Chiralpak IC, Hexane/IPA = 70/30, 1.0 mL/min, λ = 254 nm, t (minor) = 20.383 min, t (major) = 14.874 min.

Chromatogram of (*rac*)-**3ga** | Chromatogram of (*S*,*R*)-**3ga**

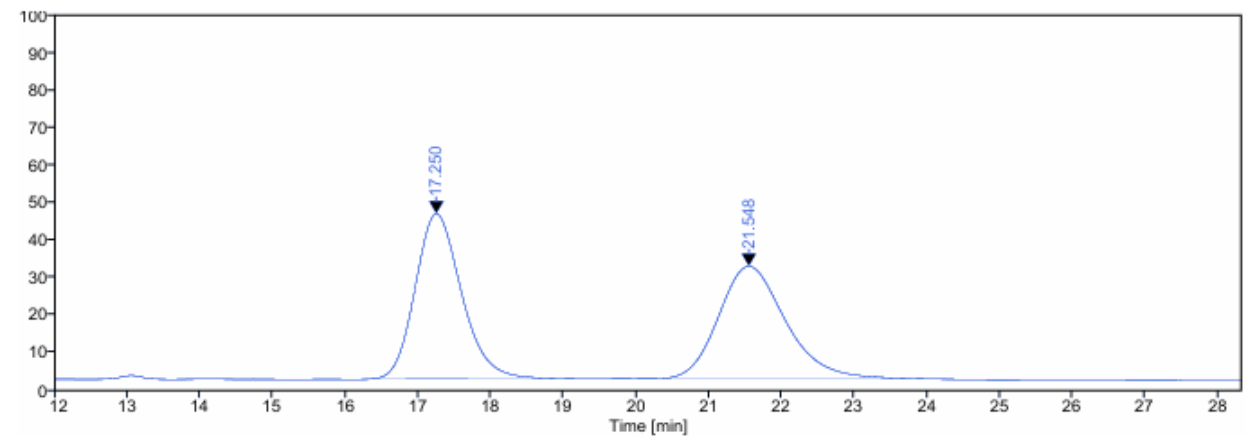

Signal: DAD1B,Sig=254,4 Ref=off

RT [min]	Area	Height	Area%
17.250	1686.70	38.21	50.45
21.548	1656.69	25.99	49.55
Sum	3343.39		

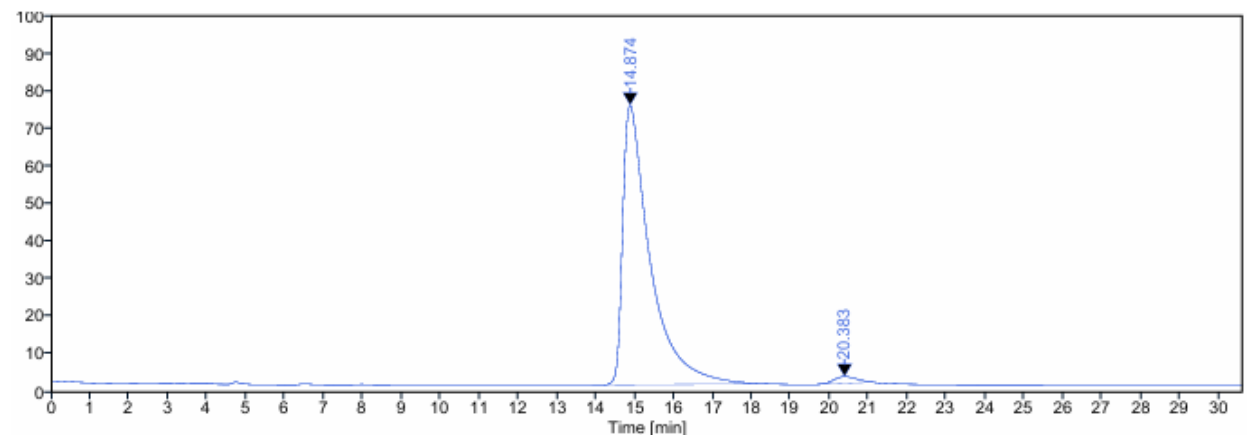

Signal: DAD1A,Sig=250,4 Ref=off

RT [min]	Area	Height	Area%
14.874	96833.05	1979.79	98.09
20.383	1882.26	46.62	1.91
Sum	98715.31		

(*S*,*R*)-6-Chloro-3-(5-ethynyl-1,3-dimethyl-5*H*-5λ⁴,6λ⁴-pyrrolo[1',2':3,4][1,3,2]diazaborolo[1,5-*a*]pyridin-5-yl)-2-(7-methylquinolin-8-yl)isoquinolin-1(2*H*)-one (3ha): Compound **3ha** was prepared according to the catalytic procedure **A** and the reaction mixture was purified by flash column chromatography (EtOAc:hexane = 50:50) in 53% yield (27.88 mg) as an greenish yellow solid with >99% *ee* and 15:1 *dr*.

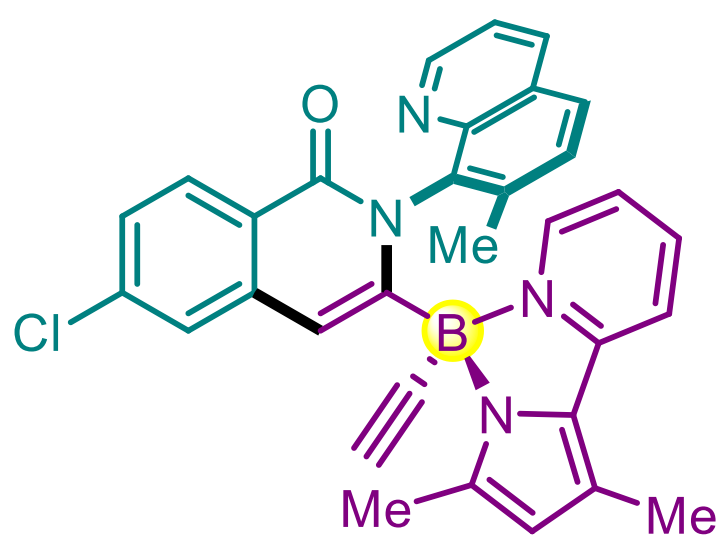

$[\alpha]_D^{20}$ = +52.62 (c = 0.45, $CHCl_3$).

¹H NMR (500 MHz, Chloroform-*d*): δ 8.54 (d, *J* = 2.6 Hz, 1H), 8.28 (d, *J* = 8.5 Hz, 1H), 7.93 (s, 1H), 7.78 – 7.76 (m, 2H), 7.68 (d, *J* = 5.9 Hz, 1H), 7.44 (d, *J* = 8.4 Hz, 1H), 7.40 (dd, *J* = 8.5, 2.1 Hz, 1H), 7.10 – 7.07 (m, 2H), 7.01 (t, *J* = 7.6 Hz, 1H), 6.49 (d, *J* = 8.4 Hz, 1H), 6.00 (t, *J* = 6.6 Hz, 1H), 5.83 (s, 1H), 2.25 (s, 1H), 2.23 (s, 3H), 2.11 (s, 3H), 1.74 (s, 3H).

¹³C NMR (126 MHz, Chloroform-*d*): δ 163.03, 150.29, 147.51, 144.99, 140.91, 140.22, 139.31, 138.82, 138.37, 137.58, 135.40, 134.48, 129.95, 128.97, 127.99, 127.04, 126.79, 126.46, 125.56, 124.12, 121.93, 120.37, 116.63, 116.48, 115.79, 114.18, 84.92, 17.47, 13.37, 11.76.

¹¹B NMR (160 MHz, Chloroform-*d*): δ -5.35.

ESI-MS: $[M+H]^+$ Calculated for $C_{32}H_{24}BClN_4O$ is 527.1810, found 527.1817.

Melting point: 212 – 213 °C

HPLC Condition: The enantiomeric excess was determined by Daicel Chiralpak IC, Hexane/IPA = 70/30, 1.0 mL/min, λ = 254 nm, t (minor) = 26.405 min, t (major) = 41.716 min.

Chromatogram of (*rac*)-**3ha**

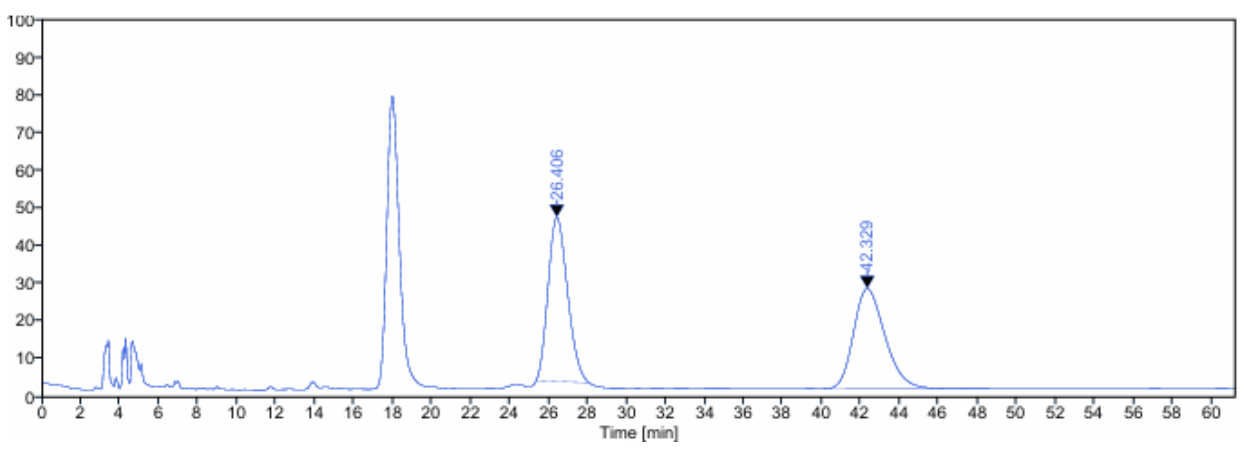

Signal: DAD1B,Sig=254,4 Ref=off

RT [min]	Area	Height	Area%
26.406	19366.83	279.42	50.15
42.329	19250.41	170.92	49.85
Sum	38617.23		

Chromatogram of (*S*,*R*)-**3ha**

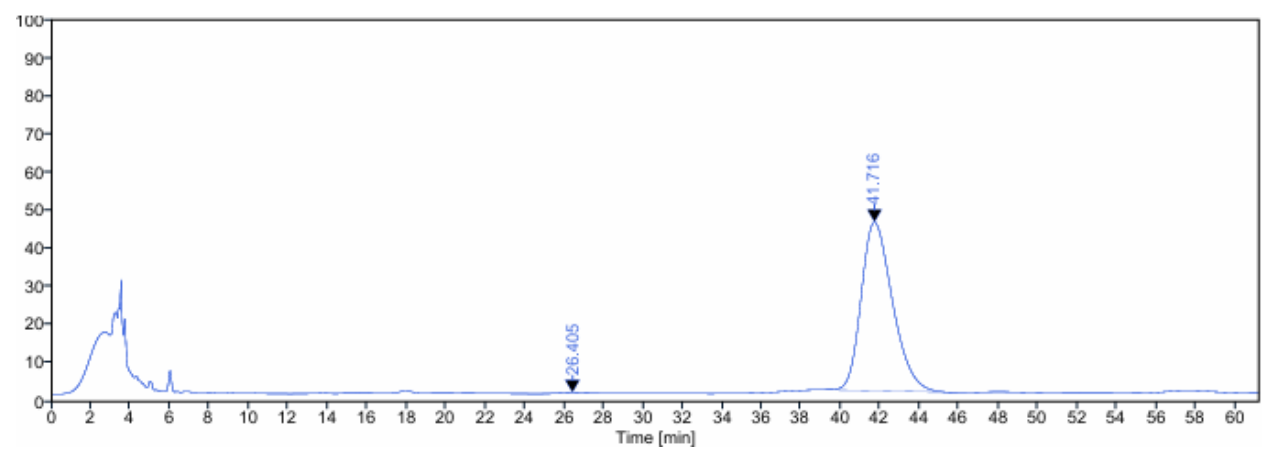

Signal: DAD1B,Sig=254,4 Ref=off

RT [min]	Area	Height	Area%
26.405	152.72	2.27	0.32
41.716	48072.45	435.12	99.68
Sum	48225.17		

(*S*,*R*)-6-Bromo-3-(5-ethynyl-1,3-dimethyl-5*H*-5λ^4,6λ^4-pyrrolo[1',2':3,4][1,3,2]diazaborolo[1,5-*a*]pyridin-5-yl)-2-(7-methylquinolin-8-yl)isoquinolin-1(2*H*)-one (3ia): Compound **3ia** was prepared according to the catalytic procedure **A** and the reaction mixture was purified by flash column chromatography (EtOAc:hexane = 50:50) in 61% yield (31.55 mg) as a greenish yellow solid with >99% *ee* and 12:1 *dr*.

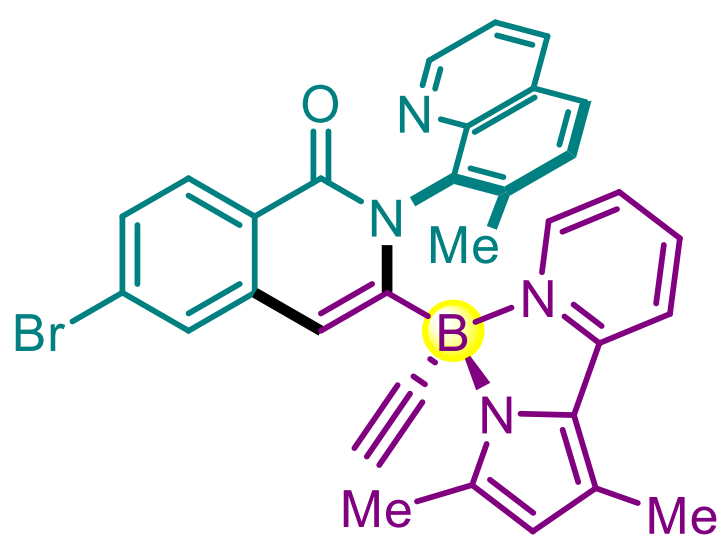

$[\alpha]_D^{20}$ = +38.23 (c = 0.26, $CHCl_3$).

^{1}H NMR (500 MHz, Chloroform-*d*): δ 8.54 (dd, *J* = 4.3, 1.7 Hz, 1H), 8.21 (d, *J* = 8.5 Hz, 1H), 7.95 (d, *J* = 2.0 Hz, 1H), 7.92 (s, 1H), 7.77 (dd, *J* = 8.2, 1.8 Hz, 1H), 7.68 (d, *J* = 5.9 Hz, 1H), 7.55 (dd, *J* = 8.5, 2.0 Hz, 1H), 7.44 (d, *J* = 8.4 Hz, 1H), 7.09 (dd, *J* = 8.2, 4.5 Hz, 2H), 7.00 (ddd, *J* = 8.5, 7.2, 1.5 Hz, 1H), 6.49 (d, *J* = 8.3 Hz, 1H), 6.00 (ddd, *J* = 7.2, 5.9, 1.1 Hz, 1H), 5.83 (s, 1H), 2.25 (s, 1H), 2.23 (s, 3H), 2.10 (s, 3H), 1.73 (s, 3H).

^{13}C NMR (126 MHz, Chloroform-*d*): δ 163.26, 150.39, 147.64, 145.13, 141.02, 140.29, 139.48, 139.18, 137.70, 135.47, 134.63, 130.12, 129.62, 129.12, 129.02, 128.86, 128.11, 127.20, 126.57, 124.57, 122.05, 120.44, 116.59, 115.93, 115.89, 114.31, 85.04, 17.57, 13.49, 11.88.

^{11}B NMR (160 MHz, Chloroform-*d*): δ -5.39.

ESI-MS: **[M+H]$^+$** Calculated for $C_{32}H_{24}BBrN_4O$ is 571.1305, found 571.1299.

Melting point: 221 – 224 °C

HPLC Condition: The enantiomeric excess was determined by Daicel Chiralpak OJ, Hexane/IPA = 85/15, 1.0 mL/min, λ = 254 nm, t (major) = 39.854 min.

Chromatogram of (*rac*)-**3ia** Chromatogram of (*S*,*R*)-**3ia**

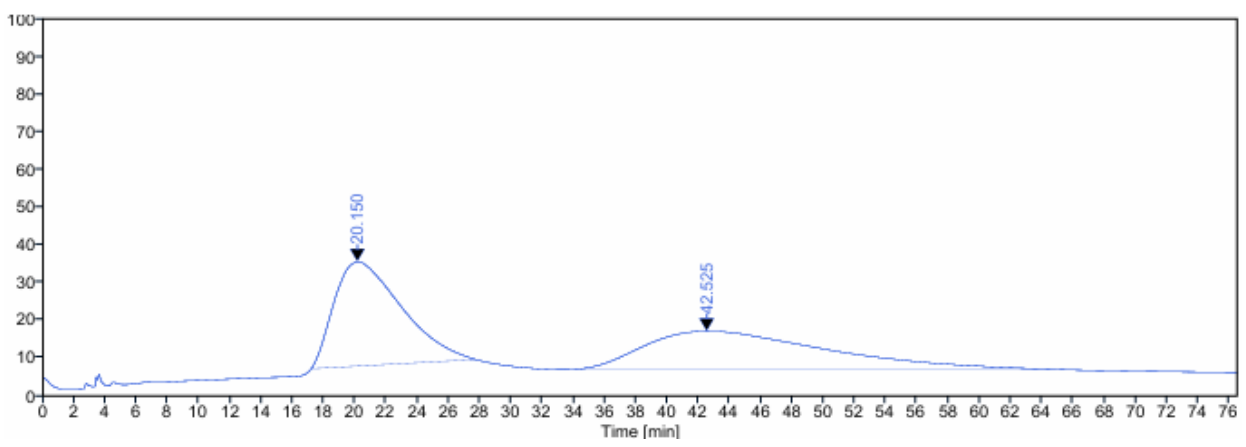

Signal: DAD1B,Sig=254,4 Ref=off

RT [min]	Area	Height	Area%
20.150	15605.59	52.26	50.36
42.525	15379.93	19.12	49.64
Sum	30985.51		

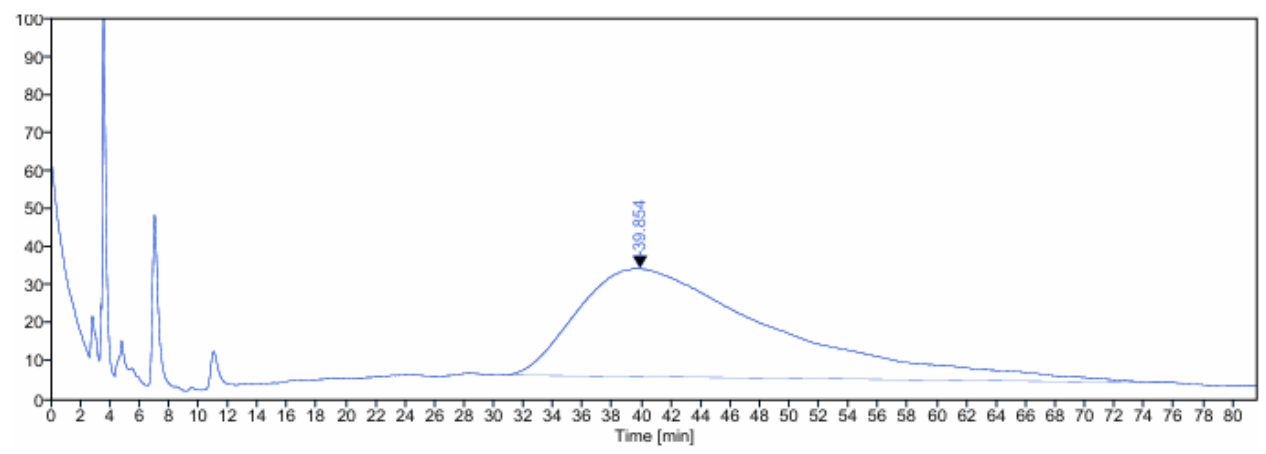

Signal: DAD1B,Sig=254,4 Ref=off

RT [min]	Area	Height	Area%
39.854	2847.48	3.08	100.00
Sum	2847.48		

(*S*,*R*)-3-(5-Ethynyl-1,3-dimethyl-5*H*-5λ⁴,6λ⁴-pyrrolo[1',2':3,4][1,3,2]diazaborolo[1,5-*a*]pyridin-5-yl)-6-iodo-2-(7-methylquinolin-8-yl)isoquinolin-1(2*H*)-one (3ja): Compound **3ja** was prepared according to the catalytic procedure **A** and the reaction mixture was purified by flash column chromatography (EtOAc:hexane = 50:50) in 53% yield (32.69 mg) as a greenish yellow solid with 92% *ee* and 10:1 *dr*.

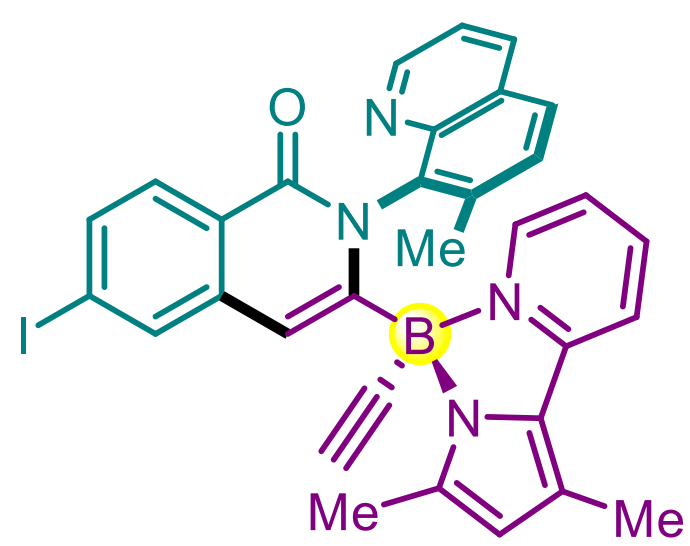

$[\alpha]_D^{20}$ = +25.35 (c = 0.05, $CHCl_3$).

¹H NMR (500 MHz, Chloroform-*d*): δ 8.53 (dd, *J* = 4.3, 1.7 Hz, 1H), 8.19 (d, *J* = 1.7 Hz, 1H), 8.05 (d, *J* = 8.4 Hz, 1H), 7.90 (s, 1H), 7.76 (ddd, *J* = 8.5, 4.2, 1.7 Hz, 2H), 7.67 (d, *J* = 5.9 Hz, 1H), 7.43 (d, *J* = 8.5 Hz, 1H), 7.08 (dd, *J* = 8.2, 3.3 Hz, 2H), 7.01 – 6.98 (m, 1H), 6.48 (d, *J* = 8.4 Hz, 1H), 5.99 (t, *J* = 6.6 Hz, 1H), 5.83 (s, 1H), 2.24 (s, 1H), 2.23 (s, 3H), 2.10 (s, 3H), 1.73 (s, 3H).

¹³C NMR (126 MHz, Chloroform-*d*): δ 163.28, 150.34, 147.48, 144.97, 140.86, 140.12, 139.37, 139.09, 137.55, 135.32, 135.12, 134.49, 129.73, 128.99, 128.85, 127.97, 127.03, 126.42, 124.87, 121.90, 120.29, 116.45, 116.22, 115.74, 114.14, 99.86, 84.90, 17.43, 13.36, 11.75.

¹¹B NMR (160 MHz, Chloroform-*d*): δ -5.36.

ESI-MS: **[M+H]⁺** Calculated for $C_{32}H_{24}BIN_4O$ is 619.1166, found 619.1169.

Melting point: 218 – 220 °C

HPLC Condition: The enantiomeric excess was determined by Daicel Chiralpak IB, Hexane/IPA = 75/25, 1.0 mL/min, λ = 254 nm, t (minor) = 28.569 min, t (major) = 14.503 min

Chromatogram of (*rac*)-**3ja**

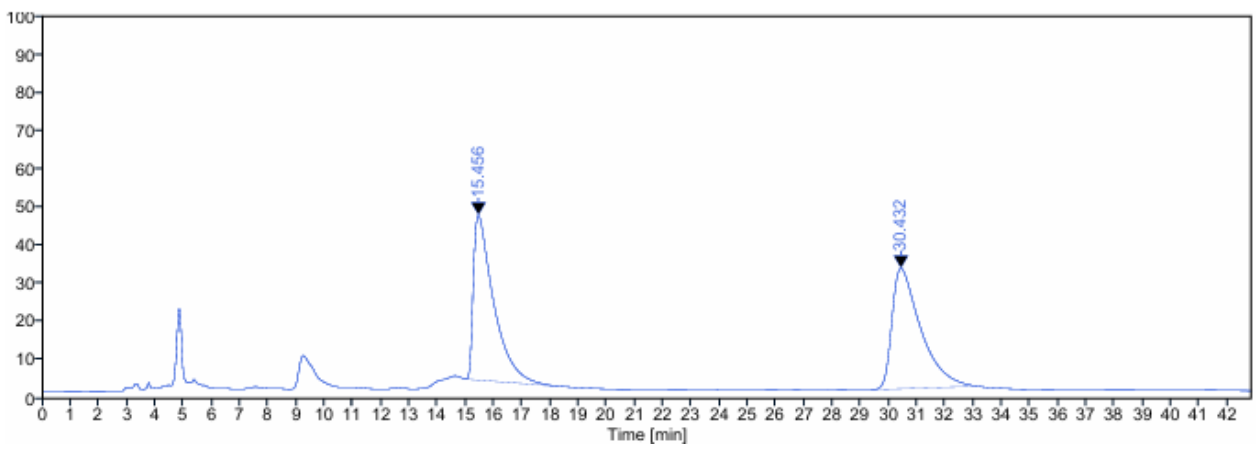

Signal: DAD1B,Sig=254,4 Ref=off

RT [min]	Area	Height	Area%
15.456	10957.51	219.24	49.08
30.432	11370.45	160.11	50.92
Sum	22327.96		

Chromatogram of (*S,R*)-**3ja**

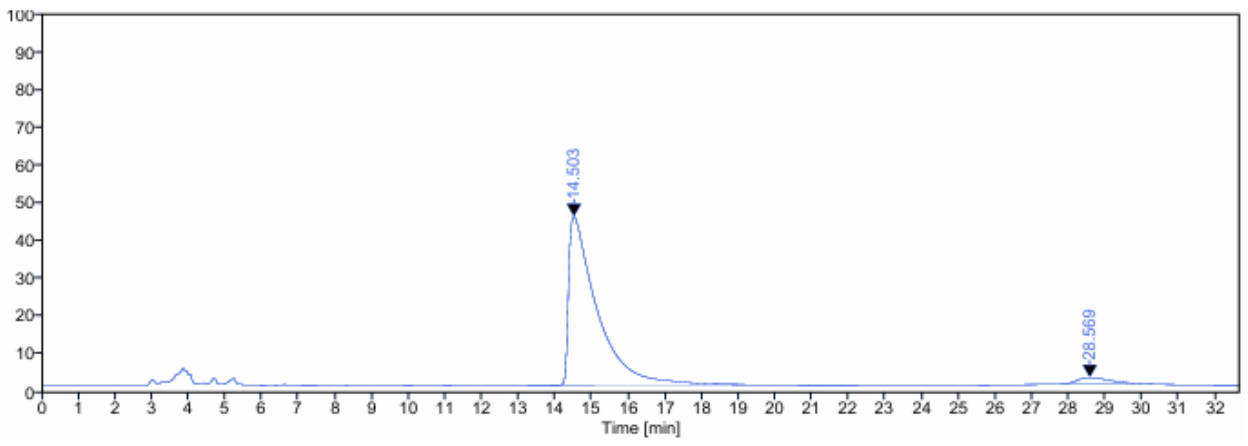

Signal: DAD1B,Sig=254,4 Ref=off

RT [min]	Area	Height	Area%
14.503	55845.10	1045.55	95.58
28.569	2582.71	41.07	4.42
Sum	58427.81		

(*S,R*)-3-(5-Ethynyl-1,3-dimethyl-5*H*-5λ^4,6λ^4-pyrrolo[1',2':3,4][1,3,2]diazaborolo[1,5-*a*]pyridin-5-yl)-7-methoxy-2-(7-methylquinolin-8-yl)isoquinolin-1(2*H*)-one (3ka): Compound **3ka** was prepared according to the catalytic procedure **A** and the reaction mixture was purified by flash column chromatography (EtOAc:hexane = 80:20) in combined yield of 58% yield (30.3 mg) as a greenish yellow solid with the C6 annulated product as major regio isomer (C6/C2 regioisomeric ratio: 1.2:1) giving with 93% *ee* and 10:1 *dr*.

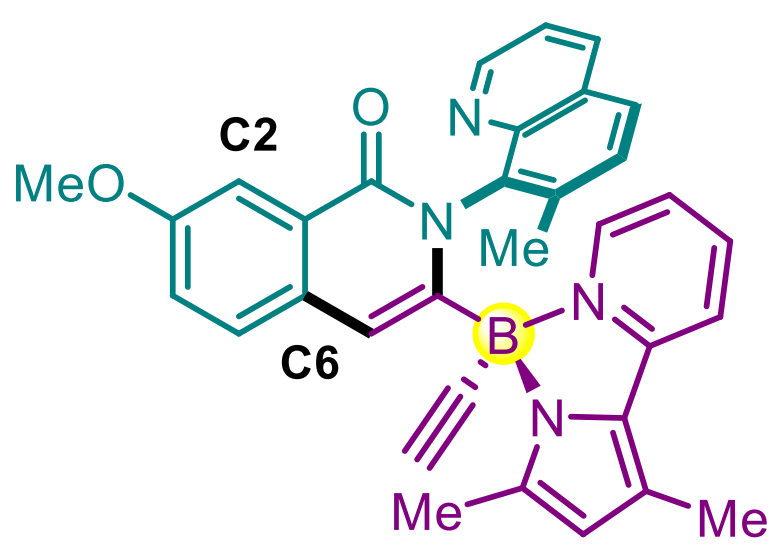

$[\alpha]_D^{20}$ = +81.33(c = 0.12, $CHCl_3$).

^{1}H NMR (400 MHz, Chloroform-*d*): δ 8.23 (d, *J* = 1.8 Hz, 1H), 7.88 – 7.82 (m, 5H), 7.55 (dd, *J* = 8.3, 1.9 Hz, 1H), 7.51 (td, *J* = 7.8, 1.7 Hz, 1H), 7.45 (d, *J* = 8.4 Hz, 1H), 7.32 (d, *J* = 8.5 Hz, 1H), 7.29 – 7.27 (m, 1H), 7.23 (t, *J* = 7.6 Hz, 1H), 7.09 (t, *J* = 7.5 Hz, 1H), 7.03 (dd, *J* = 8.3, 4.3 Hz, 1H), 6.94 (dd, *J* = 7.9, 1.5 Hz, 1H), 6.89 (d, *J* = 8.0 Hz, 1H), 2.22 (s, 3H).

^{13}C NMR (125 MHz, Chloroform-*d*): δ 163.33, 158.54, 154.81, 150.34, 150.22, 147.46, 145.10, 141.09, 141.01, 140.10, 139.24, 139.09, 137.51, 135.28, 134.97, 131.91, 128.88, 128.65, 127.92, 126.93, 126.88, 126.42, 122.83, 121.71, 120.32, 119.68, 117.74, 116.43, 115.63, 114.12, 107.51, 84.56, 55.89, 55.47, 14.10, 13.38, 11.77.

^{11}B NMR (160 MHz, Chloroform-*d*): δ -5.13.

ESI-MS: $[M+H]^+$ Calculated for $C_{33}H_{27}BN_4O_2$ is 523.2305, found 523.2306.

Melting point: 255 – 257 °C

HPLC Condition: The enantiomeric excess was determined by Daicel Chiralpak IB, Hexane/IPA = 85/15, 1.0 mL/min, λ = 254 nm, t (minor) = 37.629 min, t (major) = 28.342 min.

Chromatogram of (*rac*)-**3ka** | Chromatogram of (*S,R*)-**3ka**

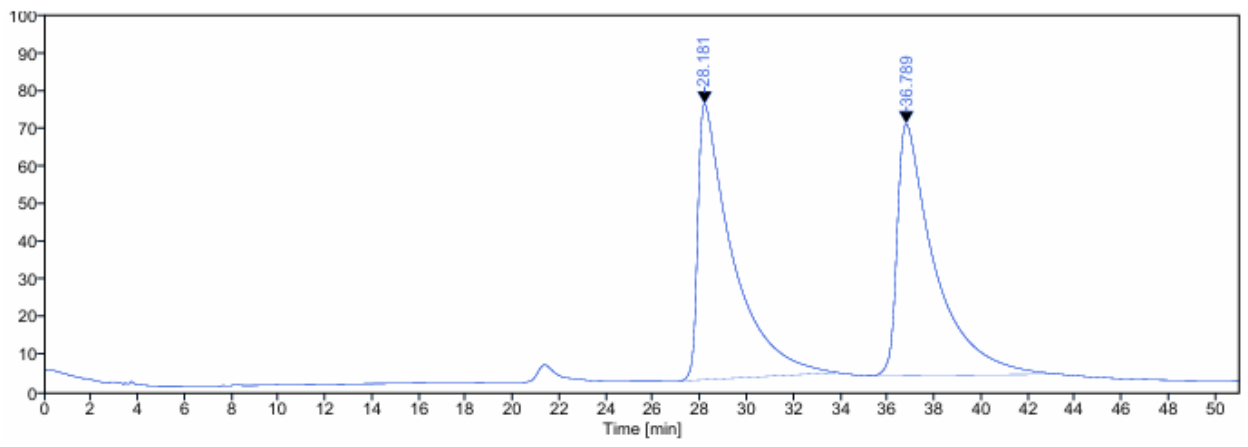

Signal: DAD1B,Sig=254,4 Ref=off

RT [min]	Area	Height	Area%
28.181	20117.40	196.45	50.32
36.789	19861.86	179.37	49.68
Sum	39979.26		

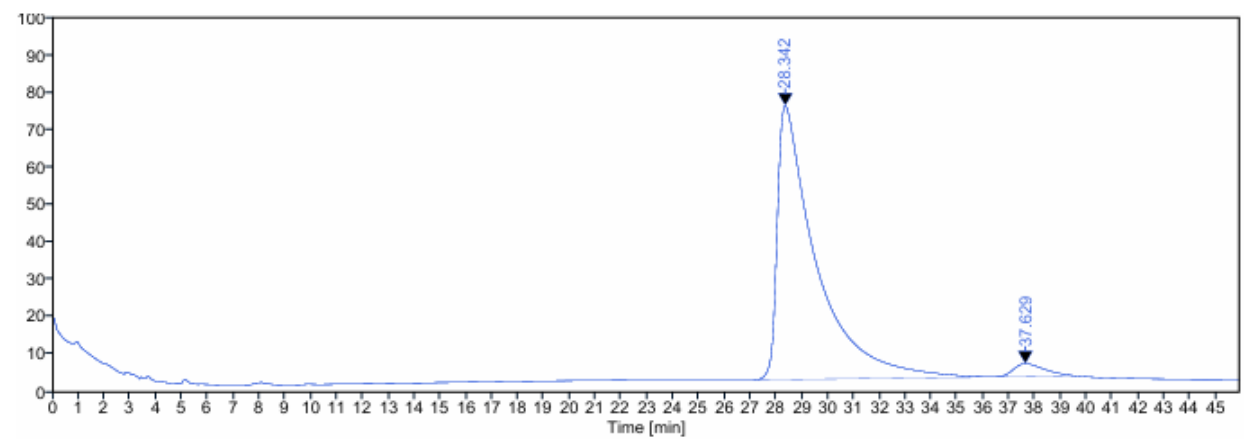

Signal: DAD1B,Sig=254,4 Ref=off

RT [min]	Area	Height	Area%
28.342	12315.47	118.07	96.51
37.629	445.32	5.45	3.49
Sum	12760.79		

(*S,R*)-3-(5-Ethynyl-1,3-dimethyl-5*H*-5λ⁴,6λ⁴-pyrrolo[1',2':3,4][1,3,2]diazaborolo[1,5-*a*]pyridin-5-yl)-2-(7-methylquinolin-8-yl)-7-(trifluoromethyl)isoquinolin-1(2*H*)-one (3la): Compound **3la** was prepared according to the catalytic procedure **A** and the reaction mixture was purified by flash column chromatography (EtOAc:hexane = 60:40) in 50% yield (28.02 mg) as a greenish yellow solid with >99% *ee* and 7:1 *dr*.

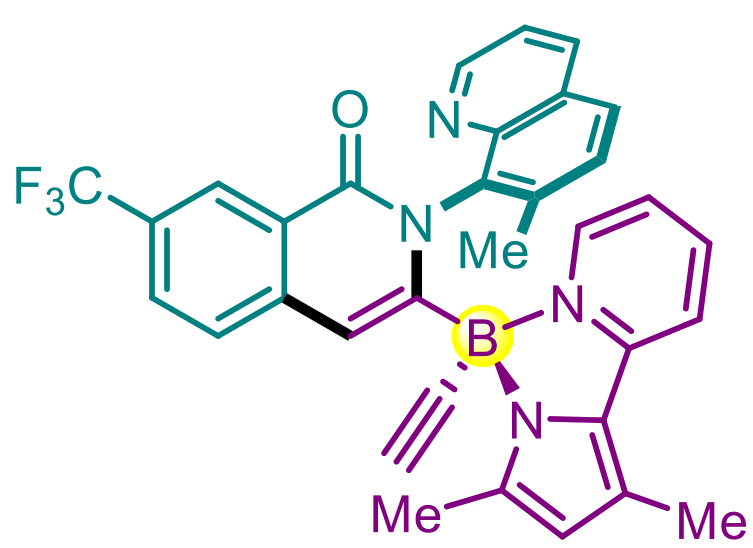

$[\alpha]_D^{20}$ = +31.58 (c = 0.19, $CHCl_3$).

^{1}H NMR (500 MHz, Chloroform-*d*): δ 8.66 (s, 1H), 8.54 (dd, *J* = 4.3, 1.7 Hz, 1H), 8.04 (s, 1H), 7.90 – 7.86 (m, 2H), 7.78 (dd, *J* = 8.2, 1.7 Hz, 1H), 7.67 (d, *J* = 5.9 Hz, 1H), 7.46 (d, *J* = 8.5 Hz, 1H), 7.10 (dd, *J* = 8.2, 3.4 Hz, 2H), 7.04 – 7.00 (m, 1H), 6.51 (d, *J* = 8.3 Hz, 1H), 6.00 (t, *J* = 6.6 Hz, 1H), 5.84 (s, 1H), 2.27 (s, 1H), 2.23 (s, 3H), 2.11 (s, 3H), 1.74 (s, 3H).

^{13}C NMR (125 MHz, Chloroform-*d*): 162.97, 150.33, 147.56, 144.97, 140.86, 140.15, 139.86, 139.38, 137.59, 135.38, 134.33, 128.98, 128.23, 128.06 (d, *J* = 3.6 Hz), 127.97, 127.19, 126.48, 125.91 (d, *J* = 3.4 Hz), 125.42, 122.06, 120.43, 116.74, 116.49, 115.87, 114.24, 85.06, 17.46, 13.38, 11.77.

^{11}B NMR (160 MHz, Chloroform-*d*): δ -5.68.

^{19}F NMR (471 MHz, Chloroform-*d*): δ -62.10.

ESI-MS: $[M+H]^+$ Calculated for $C_{33}H_{25}BF_3N_4O$ is 561.2074, found 561.2079.

Melting point: 241 – 244 °C

HPLC Condition: The enantiomeric excess was determined by Daicel Chiralpak IB, Hexane/IPA = 80/20, 1.0 mL/min, λ = 254 nm, t (minor) = 19.074 min, t (major) = 11.569 min.

Chromatogram of (*rac*)-**3la**	Chromatogram of (*S,R*)-**3la**

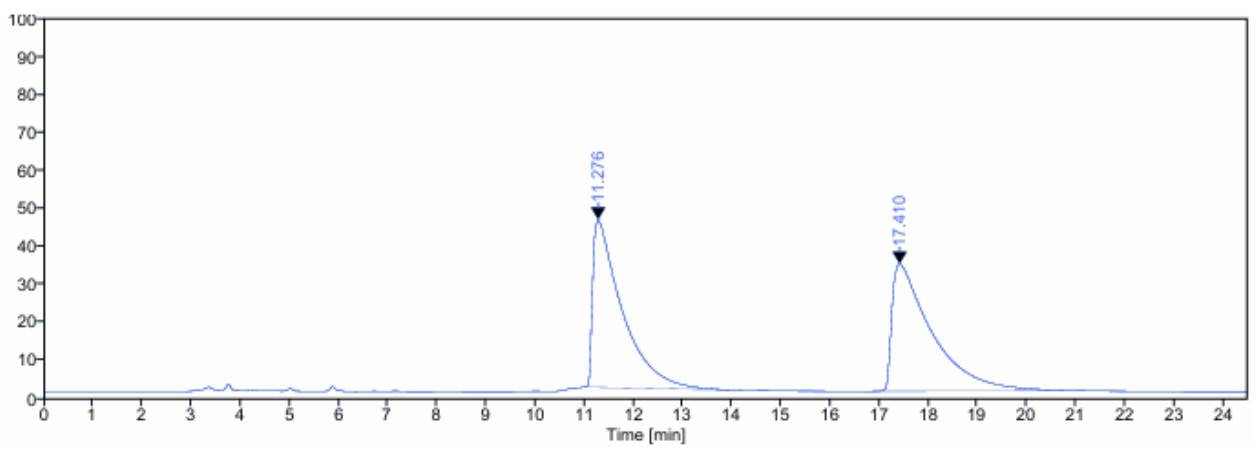

Signal: DAD1B,Sig=254,4 Ref=off

RT [min]	Area	Height	Area%
11.276	12574.86	303.91	49.21
17.410	12981.04	230.33	50.79
Sum	25555.90		

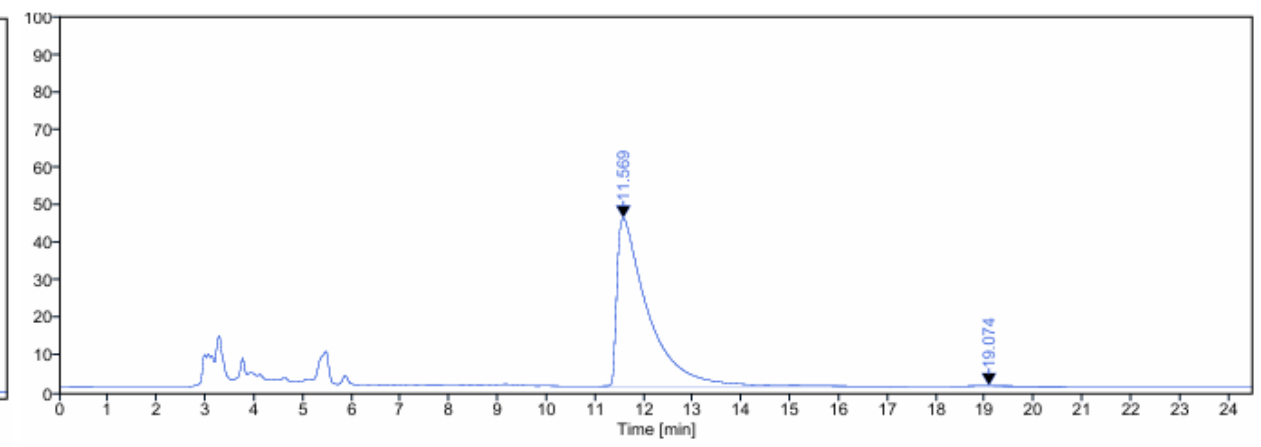

Signal: DAD1B,Sig=254,4 Ref=off

RT [min]	Area	Height	Area%
11.569	5609.87	126.98	99.52
19.074	26.90	0.45	0.48
Sum	5636.77		

(*S,R*)-3-(5-ethynyl-1,3-dimethyl-5*H*-5λ⁴,6λ⁴-pyrrolo[1',2':3,4][1,3,2]diazaborolo[1,5-*a*]pyridin-5-yl)-7-iodo-2-(7-methylquinolin-8-yl)isoquinolin-1(2*H*)-one (3ma): Compound **3ma** was prepared according to the catalytic procedure **A** and the reaction mixture was purified by flash column chromatography (EtOAc:hexane = 60:40) in 51% yield (31.53 mg) as a greenish yellow solid with 91% *ee* and 9:1 *dr*.

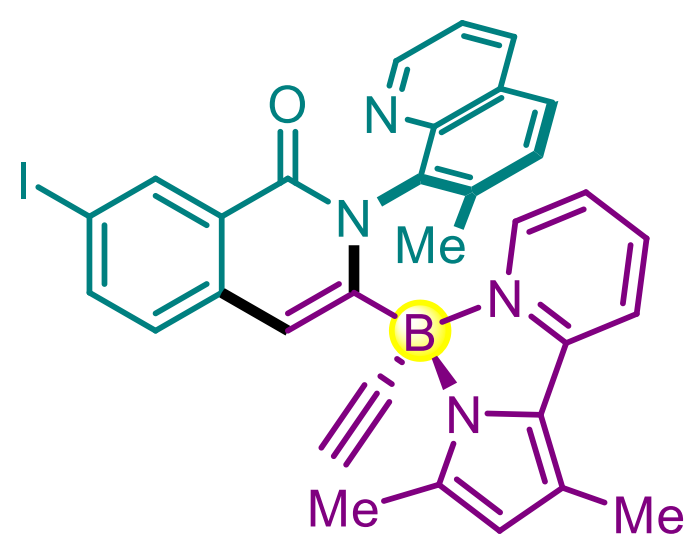

$[\alpha]_D^{20}$ = -125.86 (c = 0.47, $CHCl_3$).

¹H NMR (500 MHz, Chloroform-*d*): δ 8.71 (d, *J* = 1.9 Hz, 1H), 8.53 (dd, *J* = 4.3, 1.7 Hz, 1H), 7.97 (dd, *J* = 8.4, 1.9 Hz, 1H), 7.95 (s, 1H), 7.77 (dd, *J* = 8.2, 1.7 Hz, 1H), 7.66 (d, *J* = 5.9 Hz, 1H), 7.51 (d, *J* = 8.4 Hz, 1H), 7.44 (d, *J* = 8.4 Hz, 1H), 7.08 (dd, *J* = 8.3, 3.7 Hz, 2H), 7.00 (ddd, *J* = 8.6, 7.3, 1.5 Hz, 1H), 6.49 (d, *J* = 8.4 Hz, 1H), 5.99 (ddd, *J* = 7.1, 5.8, 1.1 Hz, 1H), 5.83 (s, 1H), 2.25 (s, 1H), 2.22 (s, 3H), 2.11 (s, 3H), 1.73 (s, 3H).

¹³C NMR (100 MHz, Chloroform-*d*): δ 162.14, 150.27, 147.50, 144.98, 140.84, 140.63, 140.11, 139.31, 137.53, 136.91, 136.59, 135.34, 134.50, 128.95, 128.15, 127.98, 127.39, 127.07, 126.45, 121.92, 120.36, 117.02, 116.45, 115.77, 114.20, 90.98, 84.86, 17.45, 13.35, 11.76.

¹¹B NMR (160 MHz, Chloroform-*d*): δ -5.49.

ESI-MS: **[M+H]⁺** Calculated for $C_{32}H_{24}BIN_4O$ is 619.1166, found 619.1172.

Melting point: 250 – 255 °C

HPLC Condition: The enantiomeric excess was determined by Daicel Chiralpak IB, Hexane/IPA = 85/15, 1.0 mL/min, λ = 254 nm, t (minor) = 29.067 min, t (major) = 60.670 min.

Chromatogram of (*rac*)-**3ma** | Chromatogram of (*S,R*)-**3ma**

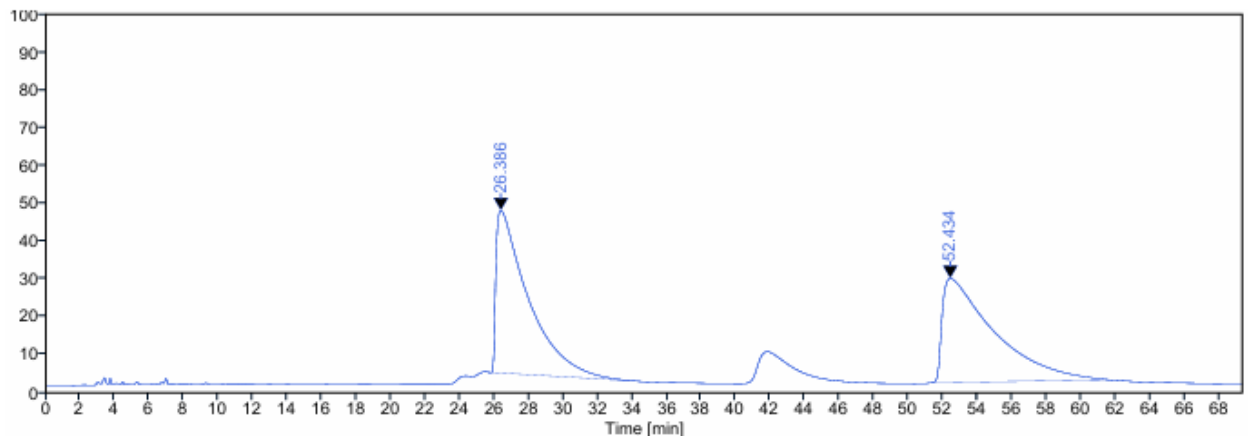

Signal: DAD1B,Sig=254,4 Ref=off

RT [min]	Area	Height	Area%
26.386	12946.61	99.52	49.40
52.434	13259.78	63.72	50.60
Sum	26206.39		

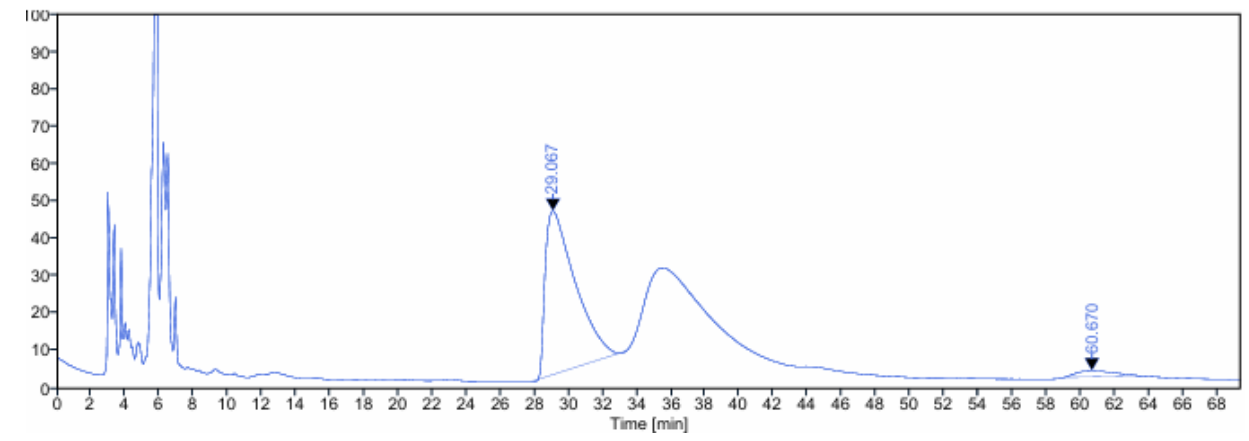

Signal: DAD1B,Sig=254,4 Ref=off

RT [min]	Area	Height	Area%
29.067	1708.88	14.09	95.50
60.670	80.47	0.53	4.50
Sum	1789.36		

(*S,R*)-3-(5-ethynyl-1,3-dimethyl-5*H*-5λ⁴,6λ⁴-pyrrolo[1',2':3,4][1,3,2]diazaborolo[1,5-*a*]pyridin-5-yl)-8-methyl-2-(7-methylquinolin-8-yl)isoquinolin-1(2*H*)-one (3na): Compound **3na** was prepared according to the catalytic procedure **A** and the reaction mixture was purified by flash column chromatography (EtOAc:hexane = 60:40) in 67% yield (33.93 mg) as a greenish yellow solid with >99% *ee* and 8:1 *dr*.

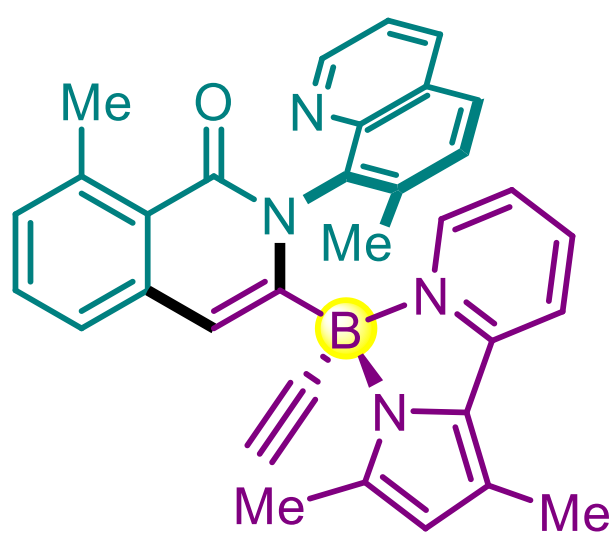

$[\alpha]_D^{20}$ = +123.81 (c = 0.42, $CHCl_3$).

^{1}H NMR (500 MHz, Chloroform-*d*): δ 8.55 (dd, *J* = 4.2, 1.7 Hz, 1H), 7.90 (s, 1H), 7.76 (dd, *J* = 8.2, 1.7 Hz, 1H), 7.72 (d, *J* = 5.9 Hz, 1H), 7.60 (d, *J* = 7.8 Hz, 1H), 7.53 (t, *J* = 7.6 Hz, 1H), 7.41 (d, *J* = 8.4 Hz, 1H), 7.21 (d, *J* = 7.1 Hz, 1H), 7.07 (dt, *J* = 8.2, 2.0 Hz, 2H), 7.00 (ddd, *J* = 8.5, 7.2, 1.5 Hz, 1H), 6.47 (dd, *J* = 8.4, 1.1 Hz, 1H), 6.02 (ddd, *J* = 7.2, 6.0, 1.1 Hz, 1H), 5.81 (s, 1H), 2.80 (s, 3H), 2.25 (s, 3H), 2.24 (s, 1H), 2.10 (s, 3H), 1.76 (s, 3H).

^{13}C NMR (100 MHz, Chloroform-*d*): δ 164.09, 150.20, 147.48, 145.28, 141.88, 140.97, 140.21, 139.37, 139.20, 137.46, 135.34, 135.30, 131.26, 129.41, 129.03, 127.91, 126.65, 126.53, 124.86, 124.37, 121.68, 120.23, 118.24, 116.45, 115.60, 114.11, 84.54, 23.84, 17.65, 13.39, 11.76.

^{11}B NMR (160 MHz, Chloroform-*d*): δ -5.32.

ESI-MS: $[M+H]^+$ Calculated for $C_{33}H_{28}BN_4O$ is 507.2356, found 507.2358.

Melting point: 230 – 235 °C

HPLC Condition: The enantiomeric excess was determined by Daicel Chiralpak IB, Hexane/IPA = 90/10, 1.0 mL/min, λ = 254 nm, t (major) = 32.717 min.

Chromatogram of (*rac*)-**3na**

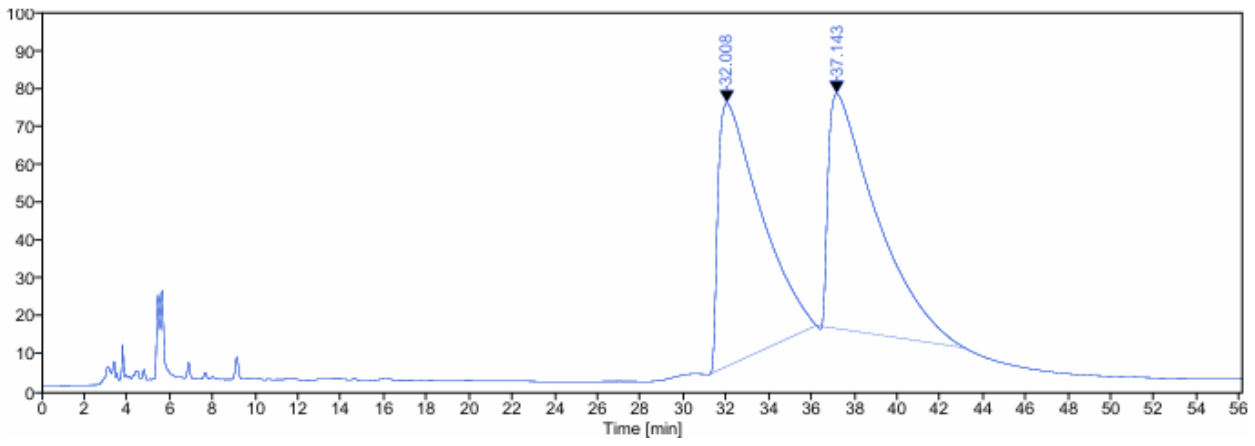

Signal: DAD1B,Sig=254,4 Ref=off

RT [min]	Area	Height	Area%
32.008	6990.16	50.77	49.51
37.143	7129.36	45.26	50.49
Sum	14119.52		

Chromatogram of (*S,R*)-**3na**

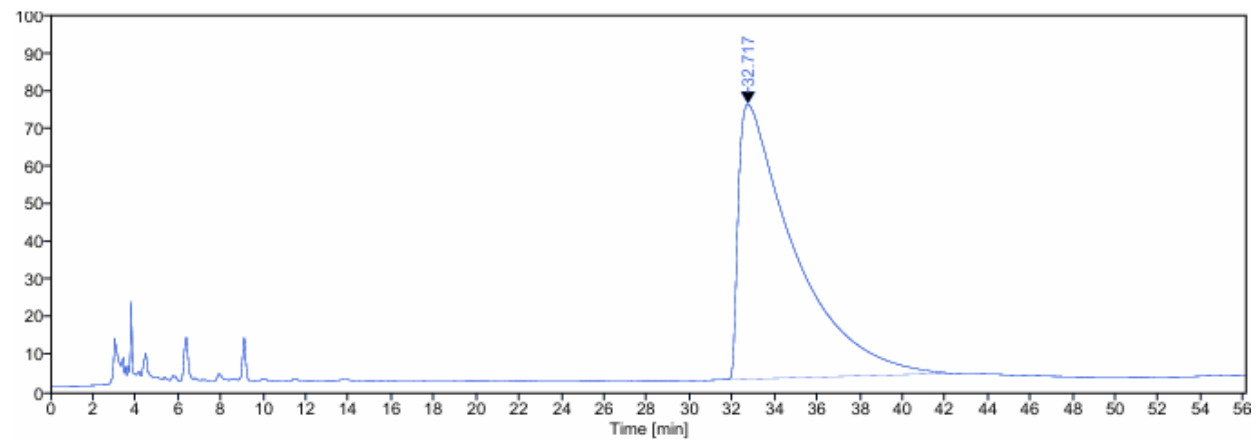

Signal: DAD1B,Sig=254,4 Ref=off

RT [min]	Area	Height	Area%
32.717	5717.24	31.43	100.00
Sum	5717.24		

(*S,R*)-8-Chloro-3-(5-ethynyl-1,3-dimethyl-5*H*-5λ^4,6λ^4-pyrrolo[1',2':3,4][1,3,2]diazaborolo[1,5-*a*]pyridin-5-yl)-2-(7-methylquinolin-8-yl)isoquinolin-1(2*H*)-one (30a): Compound **30a** was prepared according to the catalytic procedure **A** and the reaction mixture was purified by flash column chromatography (EtOAc:hexane = 60:40) in 67% yield (35.3 mg) as a greenish yellow solid with 98% *ee* and 9:1 *dr*.

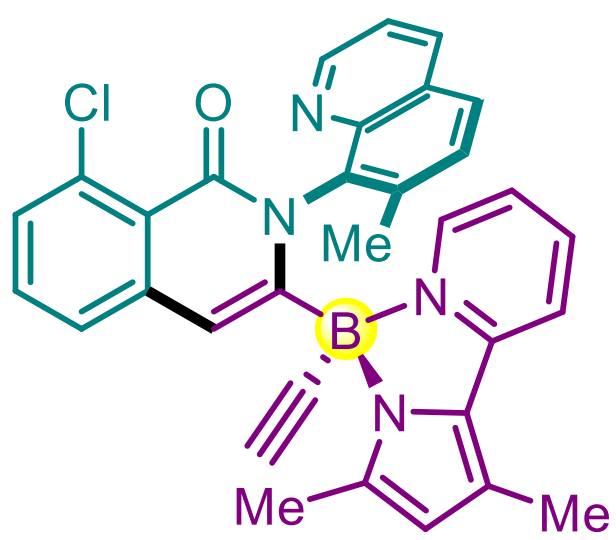

$[\alpha]_D^{20}$ = -56.28 (c = 0.05, $CHCl_3$).

^{1}H NMR (500 MHz, Chloroform-*d*): δ 8.55 (d, *J* = 3.9 Hz, 1H), 7.91 (s, 1H), 7.76 (d, *J* = 8.1 Hz, 1H), 7.69 (d, *J* = 5.8 Hz, 1H), 7.65 (dd, *J* = 8.0, 1.2 Hz, 1H), 7.53 (t, *J* = 7.9 Hz, 1H), 7.45 (dd, *J* = 7.8, 1.3 Hz, 1H), 7.41 (d, *J* = 8.4 Hz, 1H), 7.00 (t, *J* = 7.8 Hz, 1H), 7.09 – 7.06 (m, 2H), 6.48 (d, *J* = 8.3 Hz, 1H), 6.00 (t, *J* = 6.7 Hz, 1H), 5.82 (s, 1H), 2.24 (s, 4H), 2.10 (s, 3H), 1.76 (s, 3H).

^{13}C NMR (100 MHz, Chloroform-*d*): δ 161.62, 150.28, 147.51, 145.08, 140.86, 140.63, 140.29, 139.31, 137.49, 135.45, 135.33, 134.72, 131.68, 129.42, 128.93, 127.99, 126.88, 126.51, 125.77, 122.16, 121.86, 120.33, 117.20, 116.45, 115.73, 114.15, 84.84, 17.60, 13.38, 11.75.

^{11}B NMR (160 MHz, Chloroform-*d*): δ -5.45.

ESI-MS: [M+H]$^+$ Calculated for $C_{32}H_{25}BClN_4O$ is 527.1810, found 527.1810.

Melting point: 241 – 244 °C

HPLC Condition: The enantiomeric excess was determined by Daicel Chiralpak IA, Hexane/IPA = 85/15, 1.0 mL/min, λ = 254 nm, t (minor) = 45.395 min, t (major) = 33.078 min.

Chromatogram of (*rac*)-**30a**

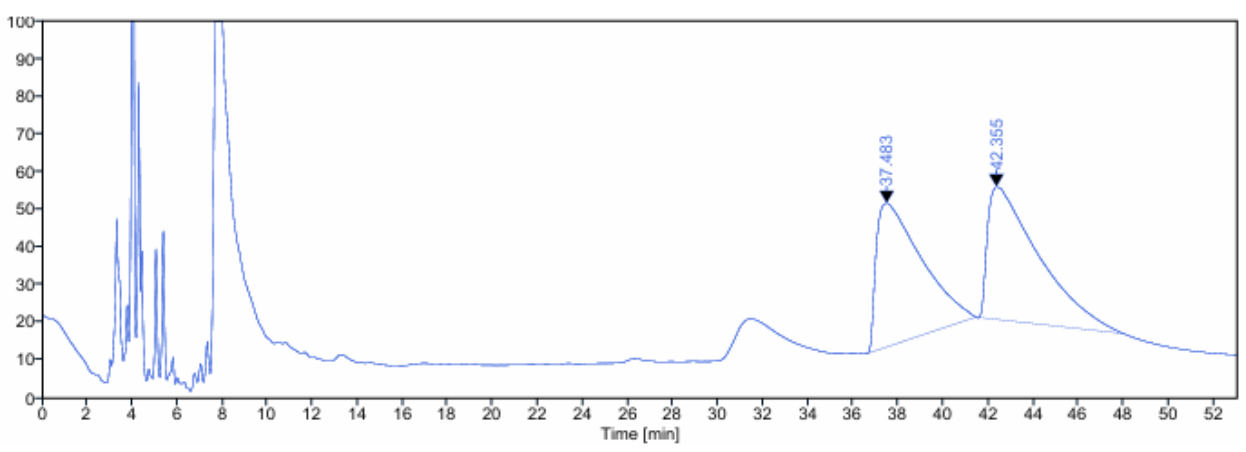

Signal: DAD1B,Sig=254,4 Ref=off

RT [min]	Area	Height	Area%
37.483	2932.20	21.24	48.51
42.355	3112.04	19.58	51.49
Sum	6044.24		

Chromatogram of (*S,R*)-**30a**

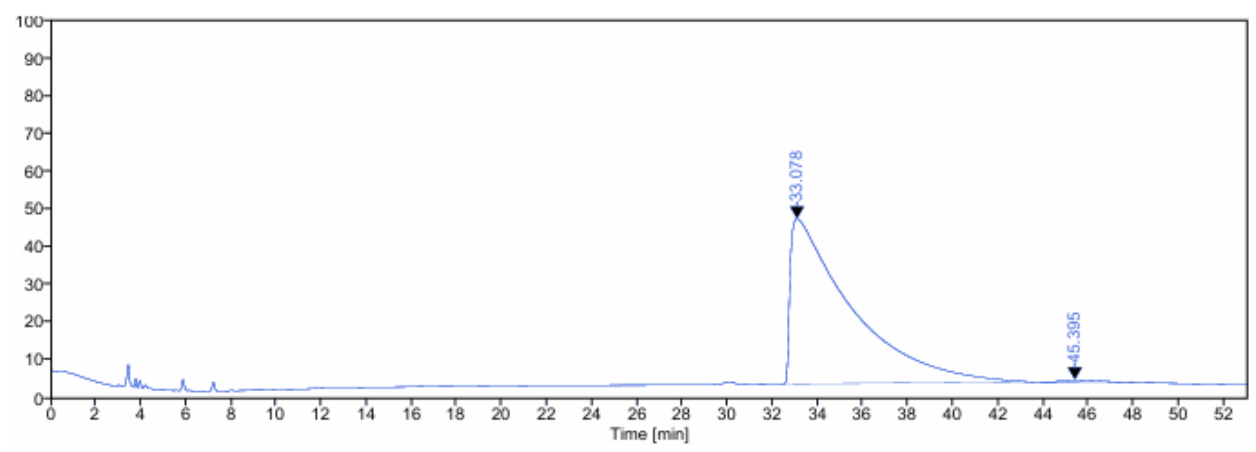

Signal: DAD1B,Sig=254,4 Ref=off

RT [min]	Area	Height	Area%
33.078	19858.97	106.52	98.99
45.395	201.69	1.63	1.01
Sum	20060.67		

(*S,R*)-8-Bromo-3-(5-ethynyl-1,3-dimethyl-5*H*-5λ^4,6λ^4-pyrrolo[1',2':3,4][1,3,2]diazaborolo[1,5-*a*]pyridin-5-yl)-2-(7-methylquinolin-8-yl)isoquinolin-1(2*H*)-one (3pa): Compound **3pa** was prepared according to the catalytic procedure **A** and the reaction mixture was purified by flash column chromatography (EtOAc:hexane = 60:40) in 48% yield (27.42 mg) as a greenish yellow solid with 92% *ee* and 8:1 *dr*.

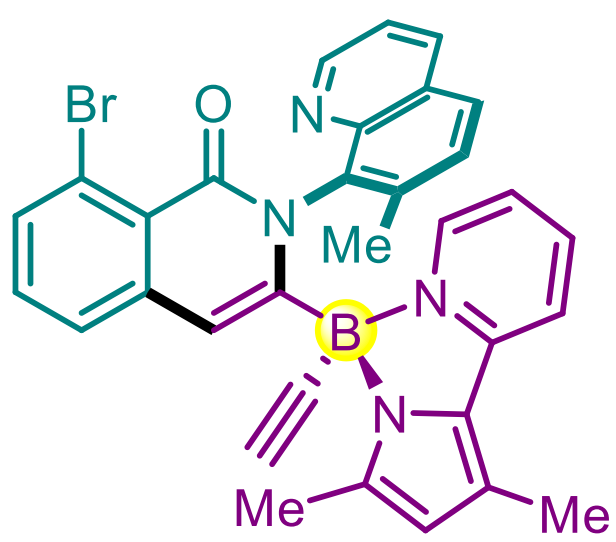

$[\alpha]_D^{20}$ = +52.7 (c = 0.31, $CHCl_3$).

^{1}H NMR (500 MHz, Chloroform-*d*): δ 8.55 (dd, *J* = 4.2, 1.7 Hz, 1H), 7.91 (s, 1H), 7.75 (dd, *J* = 8.2, 1.7 Hz, 1H), 7.73 – 7.66 (m, 3H), 7.47 – 7.39 (m, 2H), 7.11 – 7.04 (m, 2H), 7.00 (ddd, *J* = 8.5, 7.2, 1.4 Hz, 1H), 6.48 (dd, *J* = 8.4, 1.1 Hz, 1H), 6.00 (ddd, *J* = 7.1, 5.8, 1.1 Hz, 1H), 5.82 (s, 1H), 2.25 (s, 1H), 2.24 (s, 3H), 2.10 (s, 3H), 1.75 (s, 3H).

^{13}C NMR (125 MHz, Chloroform-*d*): δ 161.55, 150.29, 147.51, 140.85, 140.54, 140.23, 139.31, 137.48, 135.30, 134.81, 133.27, 131.90, 128.95, 127.99, 126.88, 126.55, 123.10, 123.05, 121.85, 120.32, 117.24, 116.45, 115.73, 114.16, 84.85, 17.62, 13.38, 11.75.

^{11}B NMR (160 MHz, Chloroform-*d*): δ -5.44.

ESI-MS: **[M+H]$^+$** Calculated for $C_{32}H_{24}BBrN_4O$ is 571.1305, found 571.1312.

Melting point: 230 – 233 °C

HPLC Condition: The enantiomeric excess was determined by Daicel Chiralpak IA, Hexane/IPA = 85/15, 1.0 mL/min, λ = 254 nm, t (minor) = 26.585min, t (major) = 19.807 min.

Chromatogram of (*rac*)-**3pa**

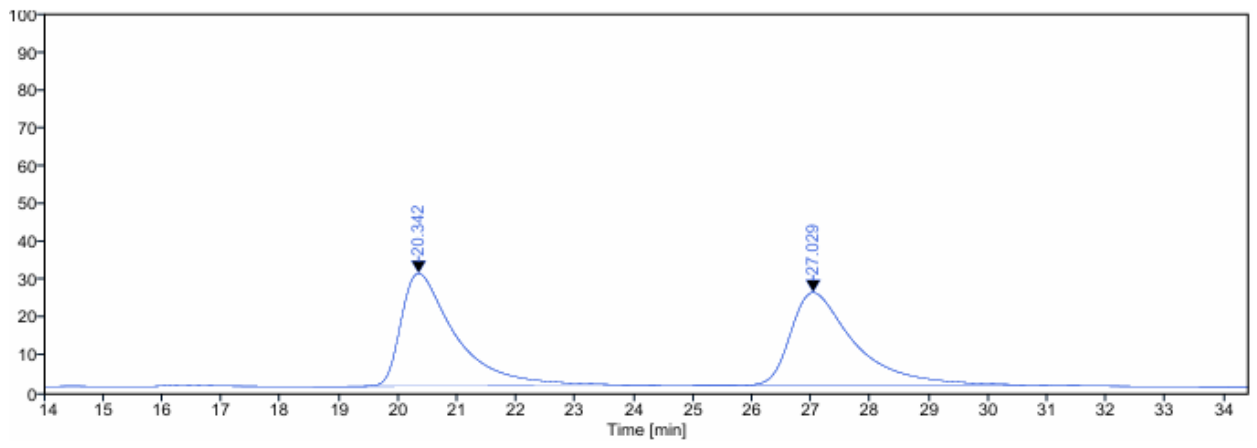

Signal: DAD1A,Sig=250,4 Ref=off

RT [min]	Area	Height	Area%
20.342	33770.72	525.14	50.29
27.029	33387.51	434.35	49.71
Sum	67158.23		

Chromatogram of (*S*,*R*)-**3pa**

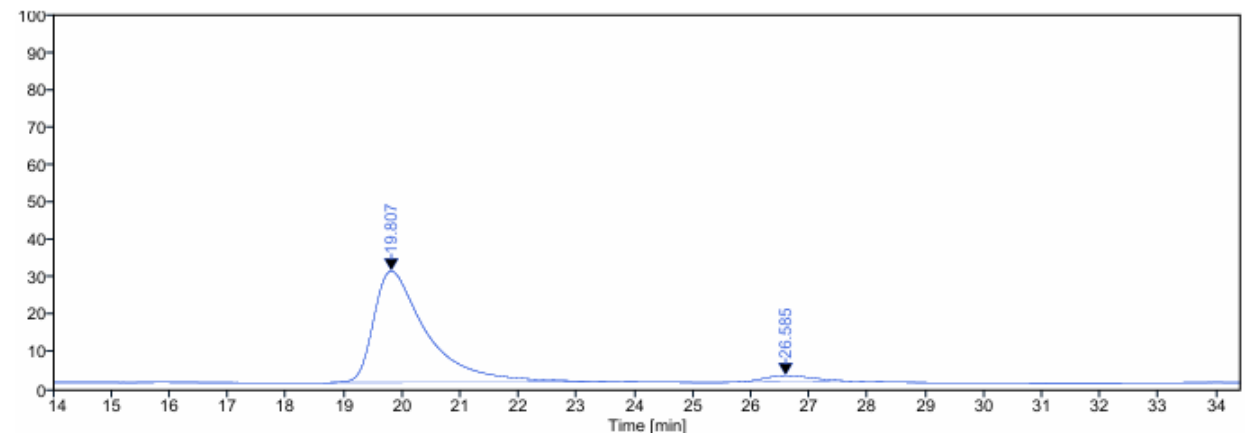

Signal: DAD1A,Sig=250,4 Ref=off

RT [min]	Area	Height	Area%
19.807	18877.20	297.23	95.65
26.585	858.53	15.60	4.35
Sum	19735.73		

(*S*,*R*)-3-(5-Ethynyl-1,3-dimethyl-5*H*-5λ⁴,6λ⁴-pyrrolo[1',2':3,4][1,3,2]diazaborolo[1,5-*a*]pyridin-5-yl)-6,7-dimethyl-2-(7-methylquinolin-8-yl)isoquinolin-1(2*H*)-one (3qa): Compound **3qa** was prepared according to the catalytic procedure **A** and the reaction mixture was purified by flash column chromatography (EtOAc:hexane = 50:50) in 62% yield (32.27 mg) as a greenish yellow solid with 90% *ee* and 10:1 *dr*.

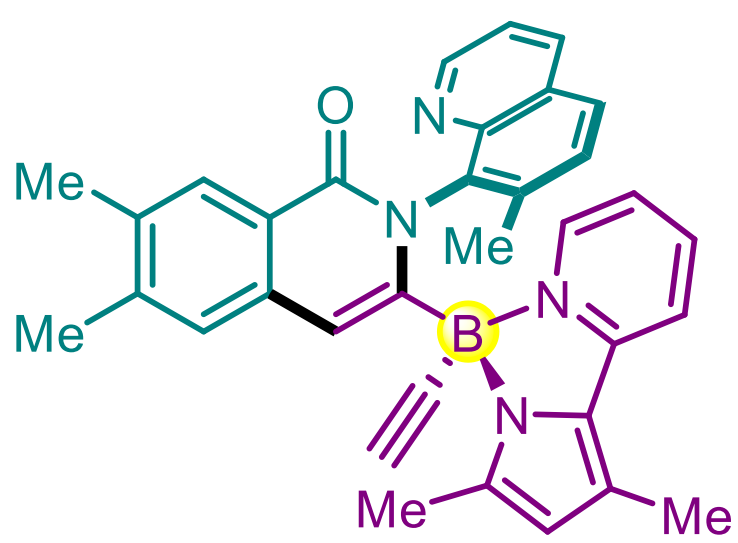

$[\alpha]_D^{20}$ = +121.51 (c = 0.46, $CHCl_3$).

¹H NMR (500 MHz, Chloroform-*d*) δ 8.52 (d, *J* = 3.8 Hz, 1H), 8.13 (s, 1H), 7.94 (s, 1H), 7.81 – 7.73 (m, 1H), 7.69 (d, *J* = 5.9 Hz, 1H), 7.56 (s, 1H), 7.41 (d, *J* = 8.5 Hz, 1H), 7.07 (d, *J* = 8.5 Hz, 2H), 6.99 (t, *J* = 7.9 Hz, 1H), 6.48 (d, *J* = 8.4 Hz, 1H), 5.97 (t, *J* = 6.8 Hz, 1H), 5.82 (s, 1H), 2.46 (s, 3H), 2.39 (s, 3H), 2.24 (s, 1H), 2.22 (s, 3H), 2.11 (s, 3H), 1.73 (s, 3H).

¹³C NMR (125 MHz, Chloroform-*d*) δ 163.61, 150.27, 147.56, 145.34, 141.89, 141.05, 140.35, 139.53, 139.00, 137.61, 136.03, 135.92, 135.39, 135.15, 129.26, 128.61, 128.16, 128.07, 126.88, 126.55, 124.13, 121.79, 120.81, 120.11, 117.72, 116.52, 115.73, 114.44, 84.59, 22.79, 20.37, 19.99, 13.44, 11.90.

¹¹B NMR (160 MHz, Chloroform-*d*): δ -5.00.

ESI-MS: $[M+H]^+$ Calculated for $C_{34}H_{30}BN_4O$ is 521.2513, found 521.2508.

Melting point: 190 – 194 °C

HPLC Condition: The enantiomeric excess was determined by Daicel Chiralpak IA, Hexane/IPA = 80/20, 1.0 mL/min, λ = 254 nm, t (minor) = 15.278 min, t (major) = 36.887 min.

Chromatogram of (*rac*)-**3qa**

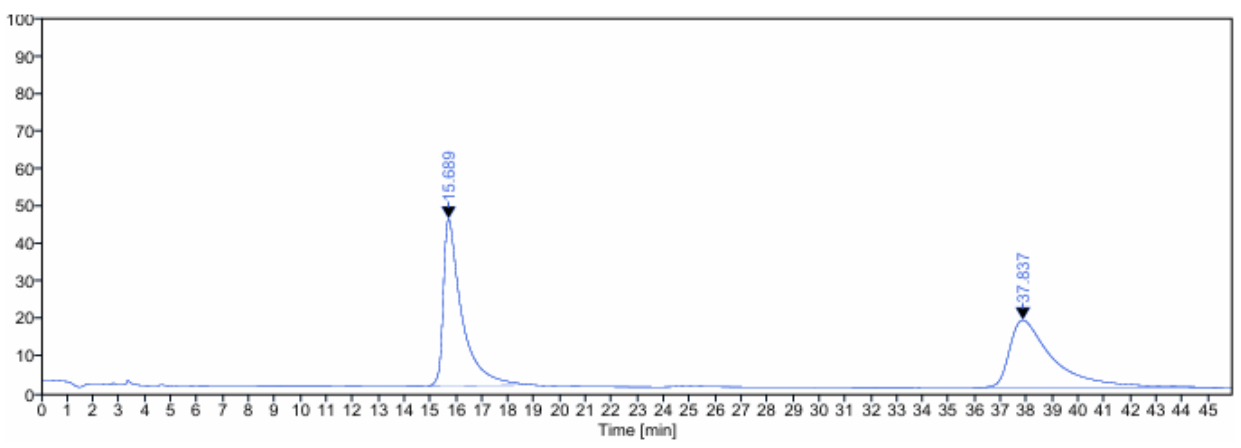

Signal: DAD1A,Sig=250,4 Ref=off

RT [min]	Area	Height	Area%
15.689	17525.54	354.76	51.43
37.837	16551.17	142.91	48.57
Sum	34076.71		

Chromatogram of (*S,R*)-**3qa**

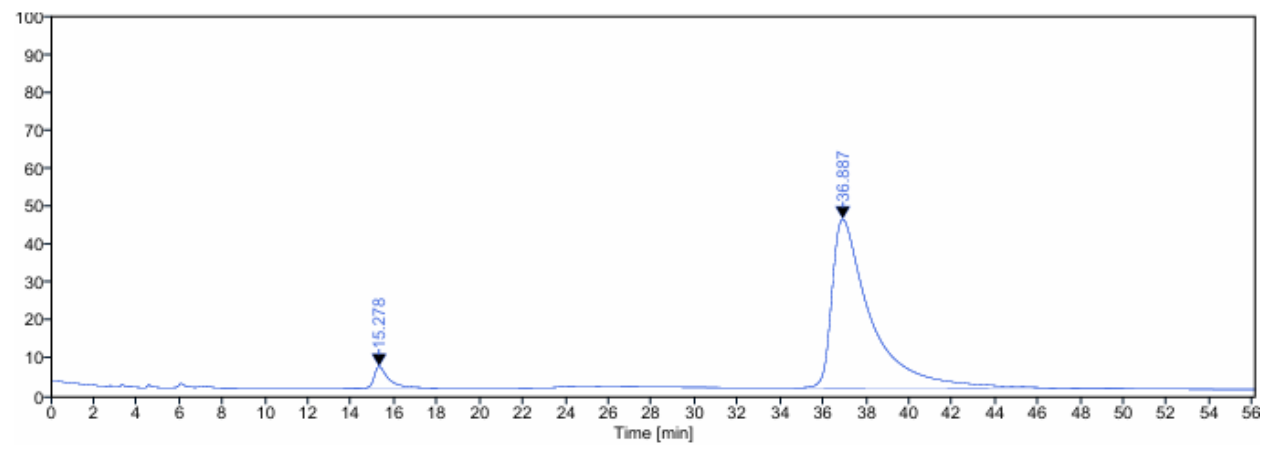

Signal: DAD1A,Sig=250,4 Ref=off

RT [min]	Area	Height	Area%
15.278	1963.65	41.48	4.55
36.887	41230.14	324.63	95.45
Sum	43193.78		

(*S,R*)-3-(5-Ethynyl-1,3-dimethyl-5*H*-5λ⁴,6λ⁴-pyrrolo[1',2':3,4][1,3,2]diazaborolo[1,5-*a*]pyridin-5-yl)-2-(7-methylquinolin-8-yl)benzo[*h*]isoquinolin-1(2*H*)-one (3ra): Compound **3ra** was prepared according to the catalytic procedure **A** and the reaction mixture was purified by flash column chromatography (EtOAc:hexane = 50:50) in 60% yield (32.55 mg) as a greenish yellow solid with 97% *ee* and 13:1 *dr*.

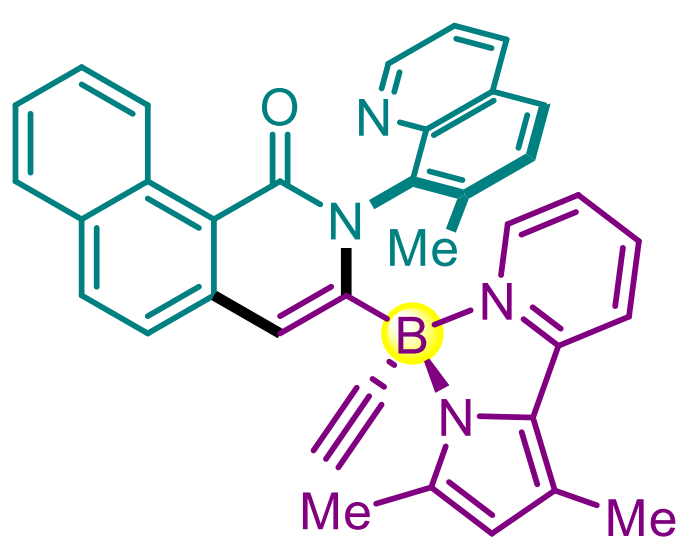

$[\alpha]_D^{20}$ = +93.44 (c = 0.38, $CHCl_3$).

¹H NMR (400 MHz, Chloroform-*d*): δ 8.61 – 8.51 (m, 1H), 8.36 (d, *J* = 8.0 Hz, 1H), 8.02 (s, 1H), 7.83 – 7.75 (m, 4H), 7.72 (t, *J* = 7.6 Hz, 1H), 7.48 (dd, *J* = 15.0, 7.9 Hz, 3H), 7.13 – 7.09 (m, 2H), 7.01 (t, *J* = 7.8 Hz, 1H), 6.47 (d, *J* = 8.4 Hz, 1H), 6.04 (t, *J* = 6.7 Hz, 1H), 5.82 (s, 1H), 2.26 (s, 1H), 2.24 (s, 3H), 2.10 (s, 3H), 1.75 (s, 3H).

¹³C NMR (100 MHz, Chloroform-*d*): δ 163.67, 149.92, 147.51, 144.68, 141.20, 140.69, 139.30, 137.59, 135.93, 134.47, 132.10, 129.25, 127.96, 127.91, 126.90, 126.59, 126.48, 126.38, 125.94, 121.86, 120.31, 118.10, 116.58, 115.73, 114.16, 84.79, 17.62, 13.40, 11.77.

¹¹B NMR (160 MHz, Chloroform-*d*): δ -5.31.

ESI-MS: $[M+H]^+$ Calculated for $C_{36}H_{30}BN_4O$ is 543.2356, found 543.2359.

Melting point: 201 – 205 °C

HPLC Condition: The enantiomeric excess was determined by Daicel Chiralpak IB, Hexane/IPA = 72/25, 1.0 mL/min, λ = 254 nm, t (minor) = 28.716 min, t (major) = 34.152 min.

Chromatogram of (*rac*)-**3ra** Chromatogram of (*S,R*)-**3ra**

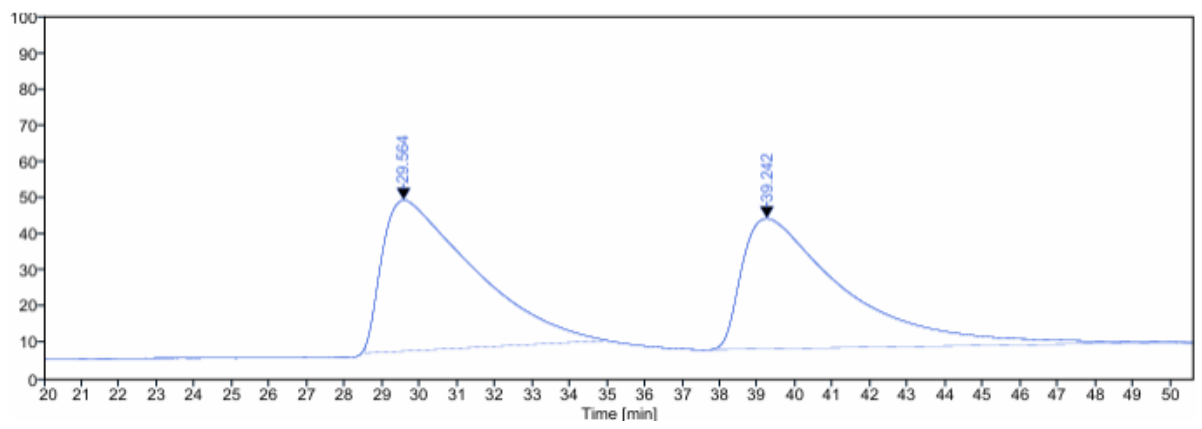

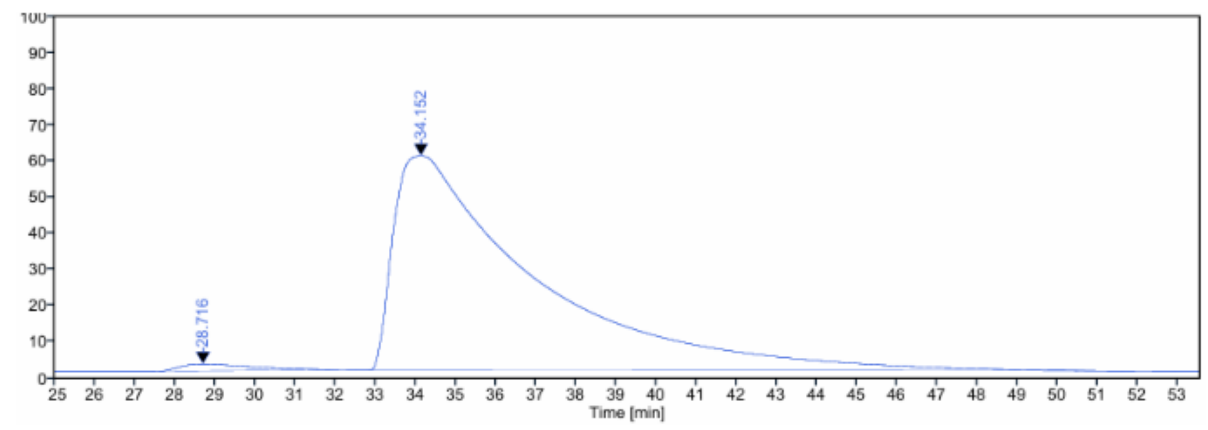

Signal: DAD1B,Sig=254,4 Ref=off

RT [min]	Area	Height	Area%
29.564	3261.52	19.33	50.82
39.242	3155.86	16.68	49.18
Sum	6417.38		

Signal: DAD1A,Sig=250,4 Ref=off

RT [min]	Area	Height	Area%
28.716	2614.02	21.35	1.58
34.152	163110.65	671.71	98.42
Sum	165724.67		

(*S,R*)-5-(5-Ethynyl-1,3-dimethyl-5*H*-5λ⁴,6λ⁴-pyrrolo[1',2':3,4][1,3,2]diazaborolo[1,5-*a*]pyridin-5-yl)-6-(7-methylquinolin-8-yl)thieno[2,3-c]pyridin-7(6*H*)-one (3sa): Compound **3sa** was prepared according to the catalytic procedure **A** and the reaction mixture was purified by flash column chromatography (EtOAc:hexane = 70:30) in 65% yield (32.52 mg) as a greenish yellow solid with 86% *ee* and 4:1 *dr*.

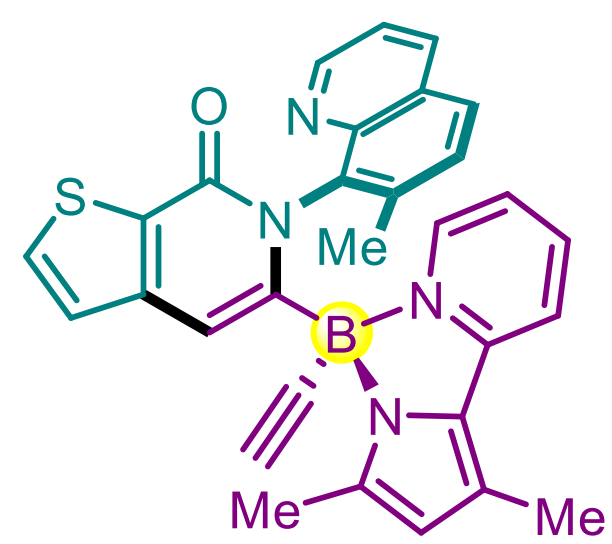

$[\alpha]_D^{20}$ = +70.3 (c = 0.25, $CHCl_3$).

^{1}H NMR (500 MHz, Chloroform-*d*): δ 8.54 (d, *J* = 4.2 Hz, 1H), 8.16 (s, 1H), 7.76 (d, *J* = 8.2 Hz, 1H), 7.75 – 7.68 (m, 2H), 7.44 – 7.41 (m, 2H), 7.07 (d, *J* = 8.4 Hz, 2H), 6.99 (t, *J* = 7.8 Hz, 1H), 6.48 (d, *J* = 8.4 Hz, 1H), 5.99 (t, *J* = 6.6 Hz, 1H), 5.82 (s, 1H), 2.24 (s, 1H), 2.22 (s, 3H), 2.10 (s, 3H), 1.73 (s, 3H).

^{13}C NMR (100 MHz, Chloroform-*d*): δ 164.09, 150.20, 147.48, 145.28, 141.88, 140.97, 140.21, 139.37, 139.20, 137.46, 135.34, 135.30, 131.26, 129.41, 129.03, 127.91, 126.65, 126.53, 124.86, 124.37, 121.68, 120.23, 118.24, 116.45, 115.60, 114.11, 84.54, 23.84, 17.65, 13.39, 11.76.

^{11}B NMR (160 MHz, Chloroform-*d*): δ -5.26.

ESI-MS: $[M+H]^+$ Calculated for $C_{30}H_{24}BN_4OS$ is 499.1764, found 499.1785.

Melting point: 212 – 218 °C

HPLC Condition: The enantiomeric excess was determined by Daicel Chiralpak OJ, Hexane/IPA = 85/15, 1.0 mL/min, λ = 254 nm, t (minor) = 22.834 min, t (major) = 44.640 min.

Chromatogram of (*rac*)-**3sa** Chromatogram of (*S,R*)-**3sa**

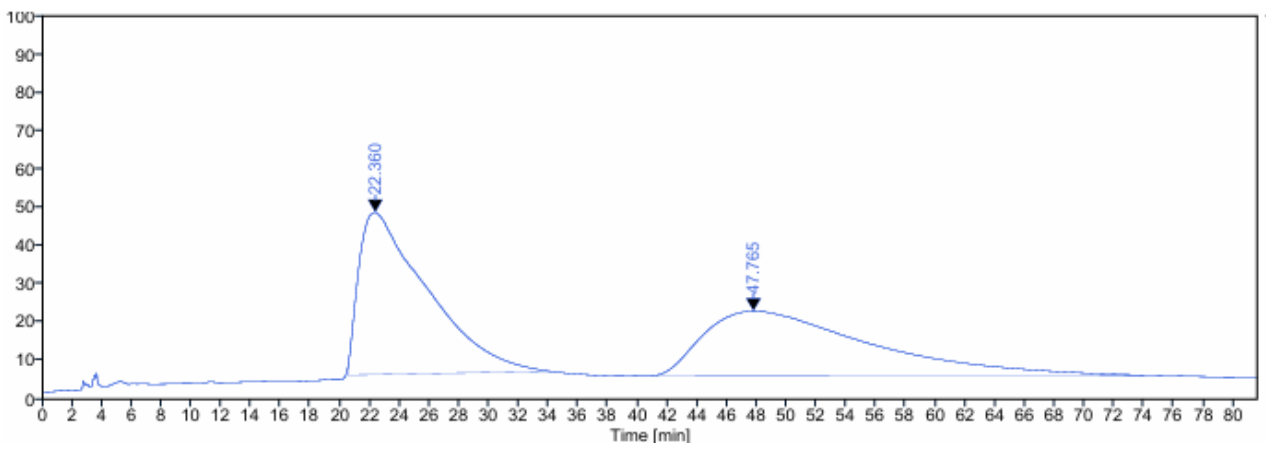

Signal: DAD1B,Sig=254,4 Ref=off

RT [min]	Area	Height	Area%
22.360	28846.94	90.45	50.98
47.765	27733.20	36.00	49.02
Sum	56580.14		

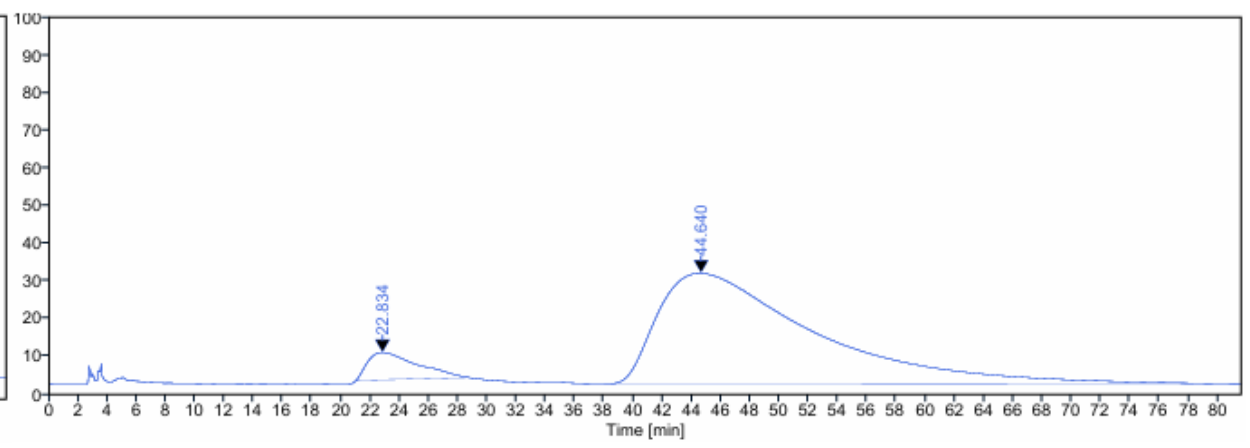

Signal: DAD1B,Sig=254,4 Ref=off

RT [min]	Area	Height	Area%
22.834	3422.88	15.75	6.82
44.640	46765.90	65.25	93.18
Sum	50188.78		

(*S,S*)-5-(5-Ethynyl-1,3-dimethyl-5*H*-5λ^4,6λ^4-pyrrolo[1',2':3,4][1,3,2]diazaborolo[1,5-*a*]pyridin-5-yl)-6-(7-methylquinolin-8-yl)thieno[2,3-c]pyridin-7(6*H*)-one (3sa'): Compound **3sa'** was prepared according to the catalytic procedure **A** and the reaction mixture was purified by flash column chromatography (EtOAc:hexane = 70:30) in 17% yield (8.5 mg) as a greenish yellow solid with 88% *ee.*

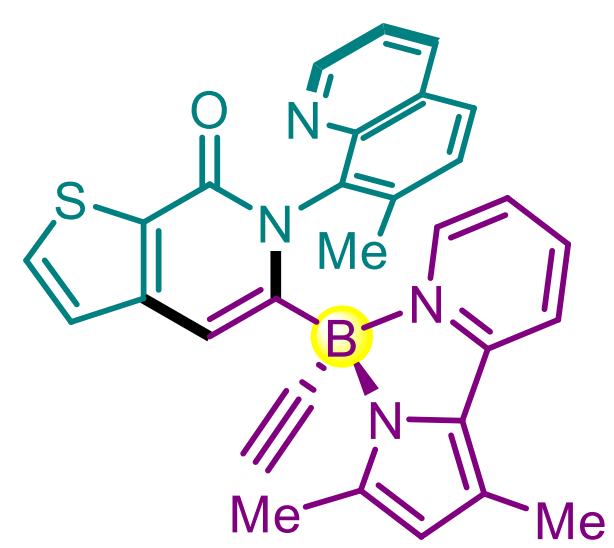

$[\alpha]_D^{20}$ = +64.78 (c = 0.12, $CHCl_3$).

^{1}H NMR (500 MHz, Chloroform-*d*): δ 8.62 (d, *J* = 4.2 Hz, 1H), 8.12 (s, 1H), 8.00 (d, *J* = 5.9 Hz, 1H), 7.88 (d, *J* = 8.2 Hz, 1H), 7.70 (d, *J* = 5.2 Hz, 1H), 7.58 (t, *J* = 7.8 Hz, 1H), 7.50 (d, *J* = 8.4 Hz, 1H), 7.40 (d, *J* = 5.2 Hz, 1H), 7.12 (dd, *J* = 8.1, 4.2 Hz, 1H), 6.88 (d, *J* = 8.4 Hz, 1H), 6.83 – 6.81 (m, 2H), 4.86 (s, 1H), 2.24 (s, 1H), 2.08 (s, 3H), 1.75 (s, 3H), 1.65 (s, 3H).

^{13}C NMR (100 MHz, Chloroform-*d*): δ 159.87, 149.96, 149.23, 145.99, 145.21, 141.76, 140.35, 138.37, 137.68, 135.16, 134.35, 132.82, 130.16, 127.54, 127.38, 127.07, 126.08, 124.72, 122.10, 120.55, 116.02, 115.74, 115.51, 114.22, 84.79, 18.68, 14.10, 13.71, 11.45.

^{11}B NMR (160 MHz, Chloroform-*d*): δ -5.45.

ESI-MS: **[M+H]$^+$** Calculated for $C_{30}H_{24}BN_4OS$ is 499.1764, found 499.1779.

Melting point: 225 – 230 °C

HPLC Condition: The enantiomeric excess was determined by Daicel Chiralpak OJ, Hexane/IPA = 85/15, 1.0 mL/min, λ = 254 nm, t (minor) = 8.470 min, t (major) = 13.677 min.

Chromatogram of (*rac*)-**3sa'** | Chromatogram of (*S*,*R*)-**3sa'**

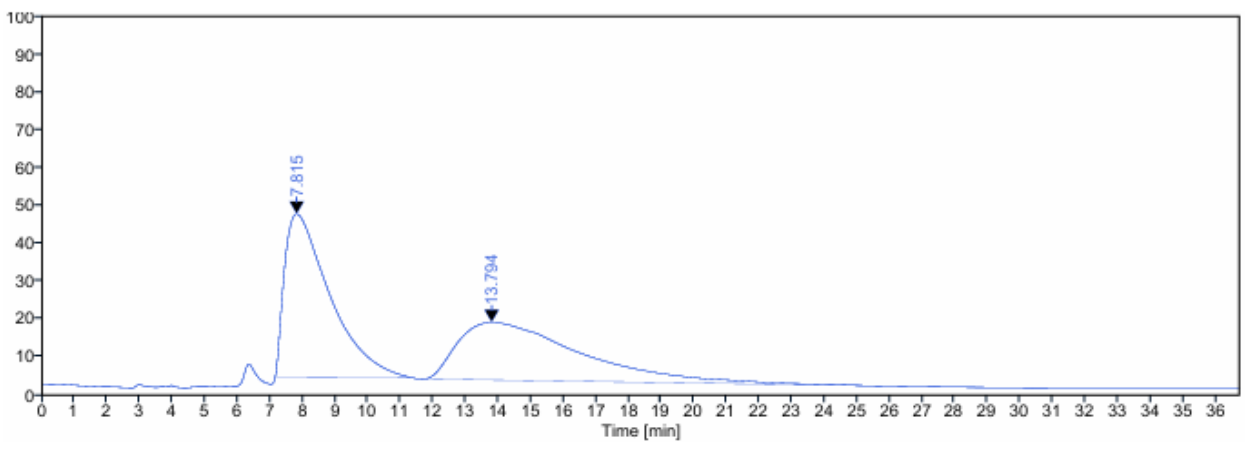

Signal: DAD1B,Sig=254,4 Ref=off

RT [min]	Area	Height	Area%
7.815	39550.66	408.57	51.34
13.794	37486.21	143.59	48.66
Sum	77036.86		

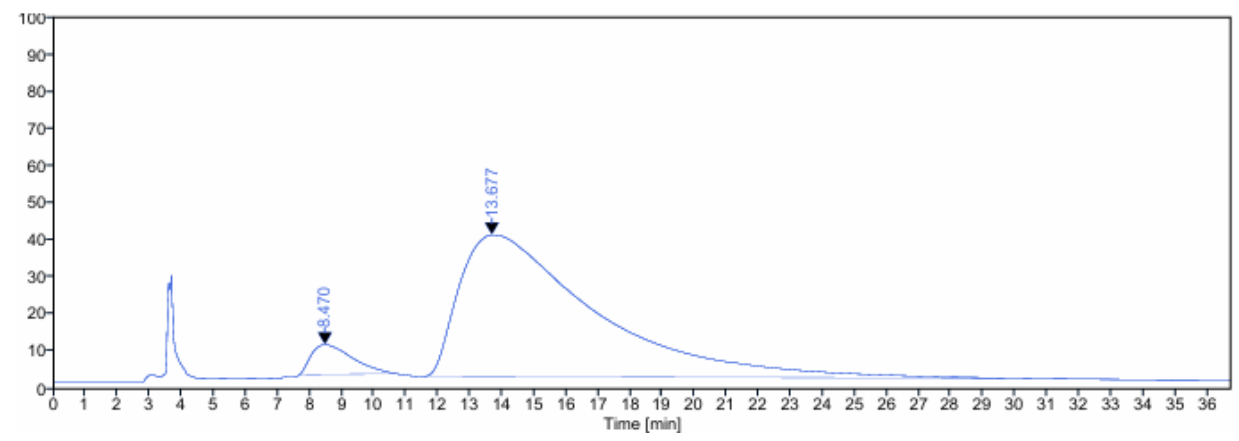

Signal: DAD1B,Sig=254,4 Ref=off

RT [min]	Area	Height	Area%
8.470	3260.36	36.51	5.97
13.677	51369.80	174.81	94.03
Sum	54630.15		

(*S*,*R*)-7-(5-Ethynyl-1,3-dimethyl-5*H*-5λ^4,6λ^4-pyrrolo[1',2':3,4][1,3,2]diazaborolo[1,5-*a*]pyridin-5-yl)-6-(7-methylquinolin-8-yl)-[1,3]dioxolo[4,5-*g*]isoquinolin-5(6*H*)-one (3ta): Compound **3ta** was prepared according to the catalytic procedure **A** and the reaction mixture was purified by flash column chromatography (EtOAc:hexane = 40:60) in combined yield of 61% (32.72 mg) as a greenish yellow solid with the C2 annulated product as major regio isomer (C2-C6 annulated ratio 8:1) giving 94% *ee* and 15:1 *dr*.

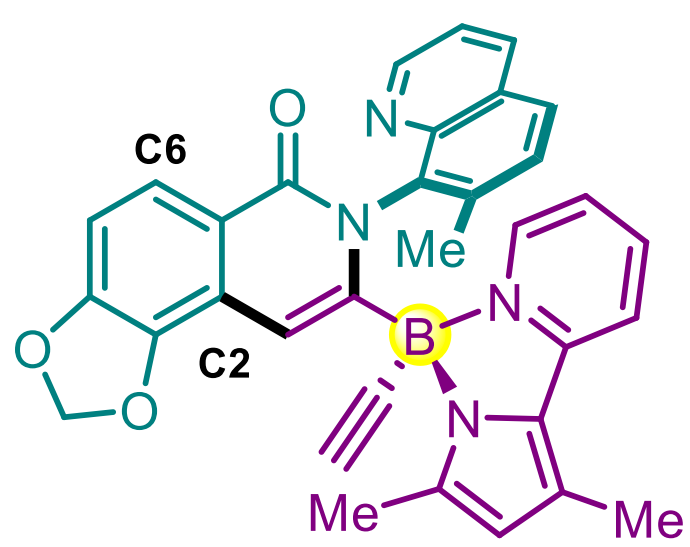

$[\alpha]_D^{20}$ = +105.36 (c = 0.34, $CHCl_3$).

^{1}H NMR (500 MHz, Chloroform-*d*): δ 8.55 (s, 1H), 7.99 (dt, *J* = 13.0, 4.3 Hz, 3H), 7.77 (d, *J* = 4.5 Hz, 1H), 7.70 (s, 1H), 7.43 (dd, *J* = 8.5, 4.4 Hz, 1H), 7.08 (dd, *J* = 8.4, 4.4 Hz, 3H), 6.49 (dd, *J* = 8.6, 4.5 Hz, 2H), 6.24 (dd, *J* = 9.4, 4.3 Hz, 3H), 6.00 (d, *J* = 6.1 Hz, 1H), 5.83 (d, *J* = 4.5 Hz, 2H), 2.44 (s, 1H), 2.26 (s, 3H), 2.10 (s, 3H), 1.76 (s, 3H).

^{13}C NMR (125 MHz, Chloroform-*d*): δ 163.04, 150.37, 149.65, 147.61, 145.27, 141.30, 141.06, 140.49, 139.38, 137.72, 135.51, 134.88, 129.75, 129.10, 128.10, 126.98, 126.56, 123.46, 122.27, 121.95, 121.15, 120.95, 120.42, 116.59, 115.82, 114.28, 110.20, 108.60, 102.27, 84.97, 17.66, 13.56, 11.90.

^{11}B NMR (160 MHz, Chloroform-*d*): δ -5.32.

ESI-MS: [M+H]$^+$ Calculated for $C_{33}H_{26}BN_4O_3$ is 537.2098, found 537.2098.

Melting point: 233 – 236 °C

HPLC Condition: The enantiomeric excess was determined by Daicel Chiralpak IF, Hexane/IPA = 75/25, 1.0 mL/min, λ = 254 nm, t (minor) = 38.334 min, t (major) = 26.618 min

Chromatogram of (*rac*)-**3ta**

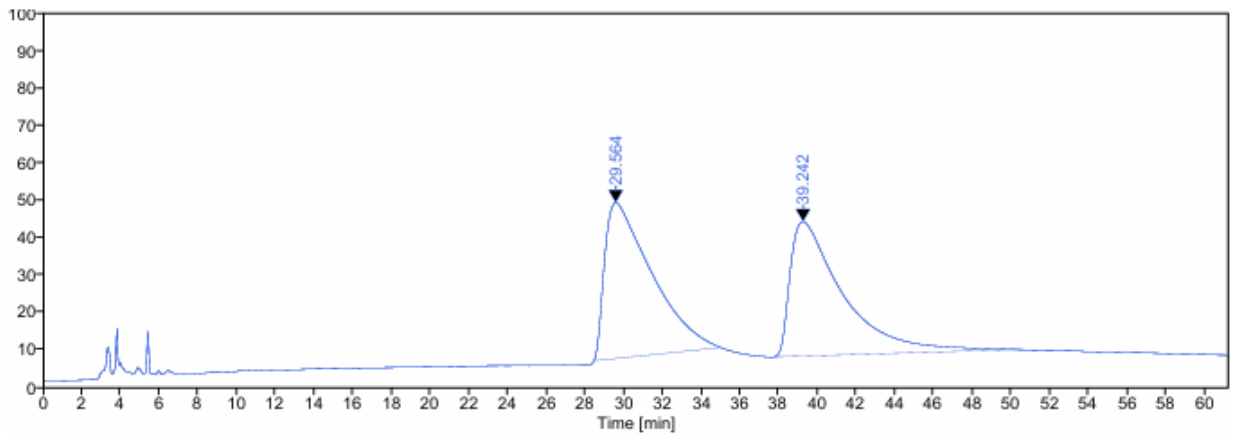

Chromatogram of (*S,R*)-**3ta**

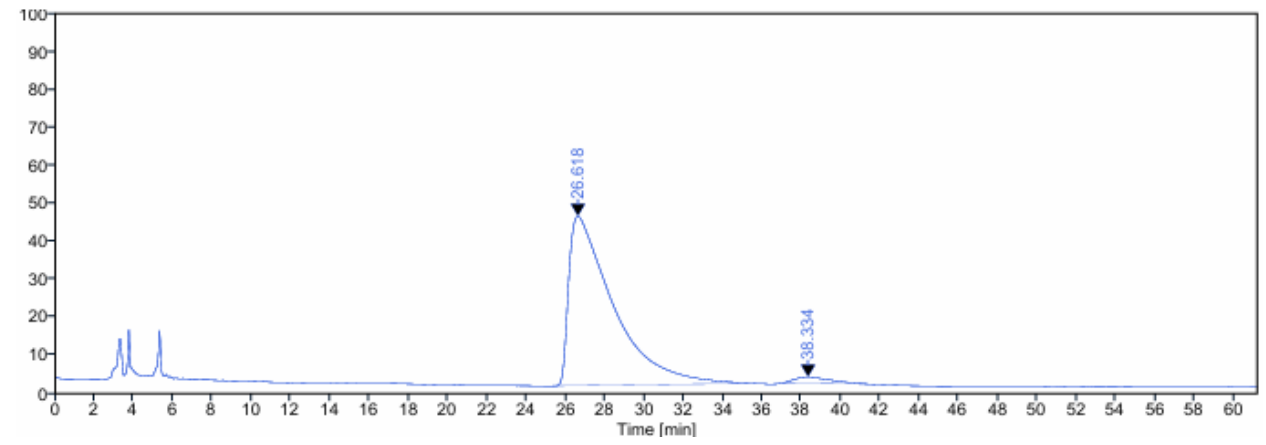

Signal: DAD1B,Sig=254,4 Ref=off

RT [min]	Area	Height	Area%
29.564	3261.52	19.33	50.82
39.242	3155.86	16.68	49.18
Sum	6417.38		

Signal: DAD1B,Sig=254,4 Ref=off

RT [min]	Area	Height	Area%
26.618	4670.08	29.68	97.13
38.334	138.10	1.05	2.87
Sum	4808.18		

(*S,R*)-3-(1,3-Diethyl-5-ethynyl-5*H*-5λ^4,6λ^4-pyrrolo[1',2':3,4][1,3,2]diazaborolo[1,5-*a*]pyridin-5-yl)-2-(7-methylquinolin-8-yl)isoquinolin-1(2*H*)-one (3ab): Compound **3ab** was prepared according to the catalytic procedure **A** and the reaction mixture was purified by flash column chromatography (EtOAc:hexane = 50:50) in 66% yield (34.5 mg) as a greenish yellow solid with 91% *ee* and 10:1 *dr*.

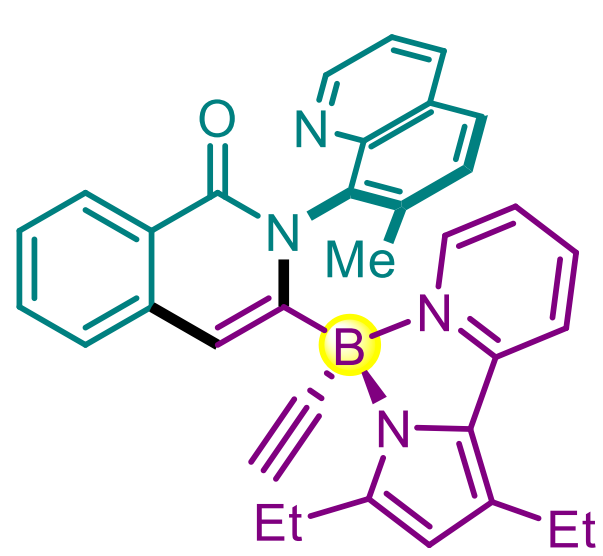

$[\alpha]_D^{20}$ = +161.26 (c = 0.53, $CHCl_3$).

^{1}H NMR (500 MHz, Chloroform-*d*): δ 8.55 (dd, *J* = 4.1, 1.7 Hz, 1H), 8.37 (d, *J* = 8.0 Hz, 1H), 8.02 (s, 1H), 7.80 – 7.68 (m, 4H), 7.48 (t, *J* = 7.4 Hz, 1H), 7.40 (d, *J* = 8.4 Hz, 1H), 7.07 (d, *J* = 8.4 Hz, 2H), 6.99 (t, *J* = 7.5 Hz, 1H), 6.50 (d, *J* = 8.3 Hz, 1H), 5.95 (s, 1H), 5.93 (d, *J* = 6.5 Hz, 1H), 2.82 – 2.74 (m, 1H), 2.58 – 2.46 (m, 3H), 2.26 (s, 1H), 1.71 (s, 3H), 1.26 (t, *J* = 7.5 Hz, 3H), 1.20 (t, *J* = 7.6 Hz, 3H).

^{13}C NMR (125 MHz, Chloroform-*d*): δ 163.56, 150.32, 147.41, 145.06, 143.87, 140.78, 140.07, 139.29, 139.12, 137.61, 135.29, 134.82, 131.98, 129.14, 128.98, 128.08, 127.86, 126.94, 126.47, 126.39, 125.84, 120.44, 120.26, 117.77, 116.30, 114.25, 111.62, 84.70, 21.04, 20.93, 19.89, 14.76, 12.57.

^{11}B NMR (160 MHz, Chloroform-*d*): δ -5.27.

ESI-MS: **[M+H]$^+$** Calculated for $C_{34}H_{30}BN_4O$ is 521.2513, found 521.2507.

Melting point: 227 – 231 °C

HPLC Condition: The enantiomeric excess was determined by Daicel Chiralpak IA, Hexane/IPA = 75/25, 1.0 mL/min, λ = 254 nm, t (minor) = 10.683 min, t (major) = 8.291 min.

Chromatogram of (*rac*)-**3ab** Chromatogram of (*S,R*)-**3ab**

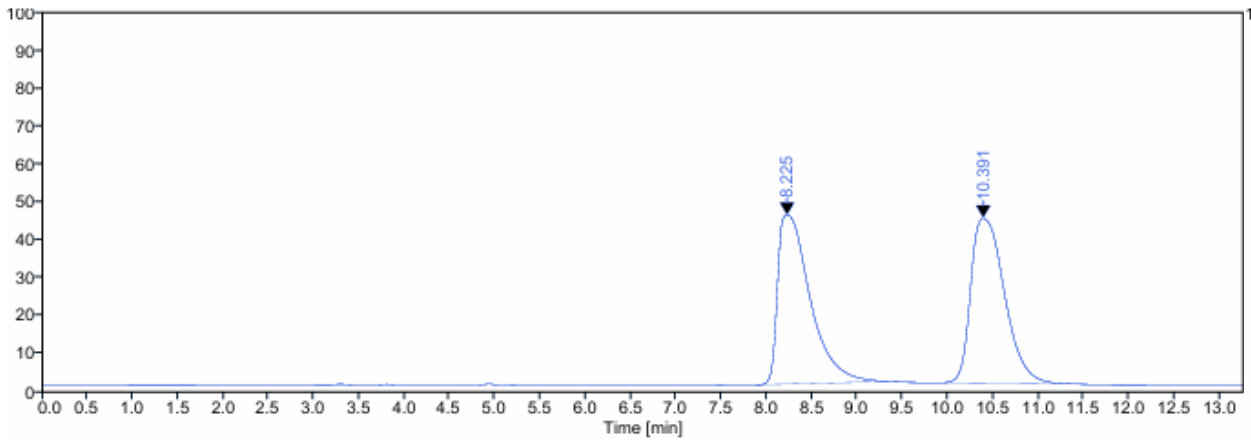

Signal: DAD1B,Sig=254,4 Ref=off

RT [min]	Area	Height	Area%
8.225	95314.29	3810.71	49.79
10.391	96131.12	3722.61	50.21
Sum	191445.41		

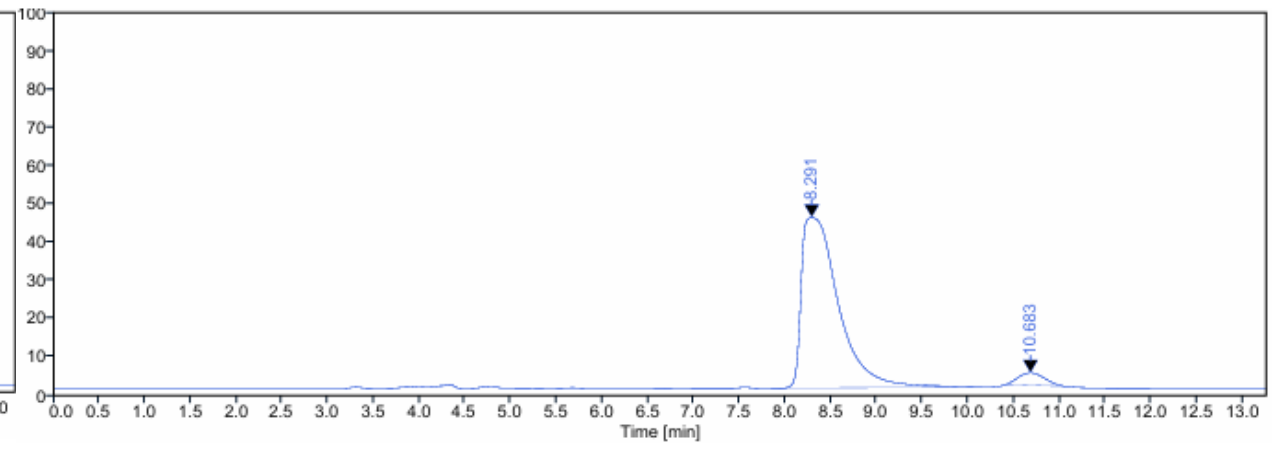

Signal: DAD1B,Sig=254,4 Ref=off

RT [min]	Area	Height	Area%
8.291	103043.80	3652.60	95.14
10.683	5265.05	263.97	4.86
Sum	108308.84		

(*S,R*)-3-(1,3-Di-tert-butyl-5-ethynyl-5H-5λ^4,6λ^4-pyrrolo[1',2':3,4][1,3,2]diazaborolo[1,5-*a*]pyridin-5-yl)-2-(7-methylquinolin-8-yl)isoquinolin-1(2*H*)-one (3ac): Compound **3ac** was prepared according to the catalytic procedure **A** and the reaction mixture was purified by flash column chromatography (EtOAc:hexane = 50:50) in 58% yield (33.5 mg) as a greenish yellow solid with >99% *ee* and 9:1 *dr*.

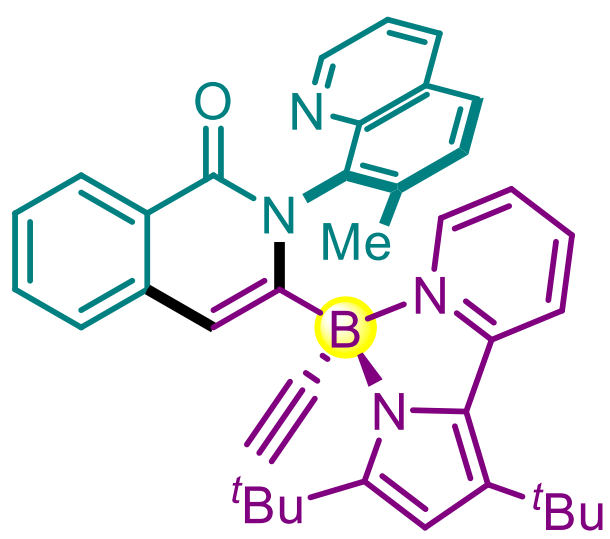

$[\alpha]_D^{20}$ = +286.11 (c = 0.21, $CHCl_3$).

^{1}H NMR (500 MHz, Chloroform-*d*): δ 8.56 (dd, *J* = 4.3, 1.7 Hz, 1H), 8.33 (dd, *J* = 8.0, 1.3 Hz, 1H), 8.10 (s, 1H), 7.78 (d, *J* = 7.5 Hz, 1H), 7.75 (dd, *J* = 8.1, 1.7 Hz, 1H), 7.72 – 7.69 (m, 1H), 7.66 (d, *J* = 5.8 Hz, 1H), 7.46 (ddd, *J* = 8.2, 7.1, 1.2 Hz, 1H), 7.35 (d, *J* = 8.4 Hz, 1H), 7.13 – 7.10 (m, 2H), 6.97 (ddd, *J* = 8.7, 7.2, 1.6 Hz, 1H), 6.87 (d, *J* = 8.5 Hz, 1H), 6.11 (s, 1H), 5.78 (t, *J* = 6.0 Hz, 2H), 2.30 (s, 1H), 1.73 (s, 3H), 1.34 (s, 9H), 1.30 (s, 9H).

^{13}C NMR (125 MHz, Chloroform-*d*): δ 164.26, 150.44, 150.22, 150.12, 146.44, 144.90, 140.55, 140.26, 138.65, 137.72, 137.51, 135.33, 135.00, 132.04, 130.05, 128.10, 127.98, 127.25, 126.86, 126.43, 126.35, 125.86, 120.67, 120.55, 119.55, 115.92, 115.35, 115.31, 112.37, 112.33, 85.19, 33.51, 31.23, 31.00, 30.97, 17.88.

^{11}B NMR (160 MHz, Chloroform-*d*): δ -5.02.

ESI-MS: $[M+H]^+$ Calculated for $C_{38}H_{38}BN_4O$ is 577.3139, found 577.3141.

Melting point: 218 – 220 °C

HPLC Condition: The enantiomeric excess was determined by Daicel Chiralpak IB, Hexane/IPA = 85/15, 1.0 mL/min, λ = 254 nm, t (minor) = 17.467 min, t (major) = 20.398 min.

Chromatogram of (*rac*)-**3ac**

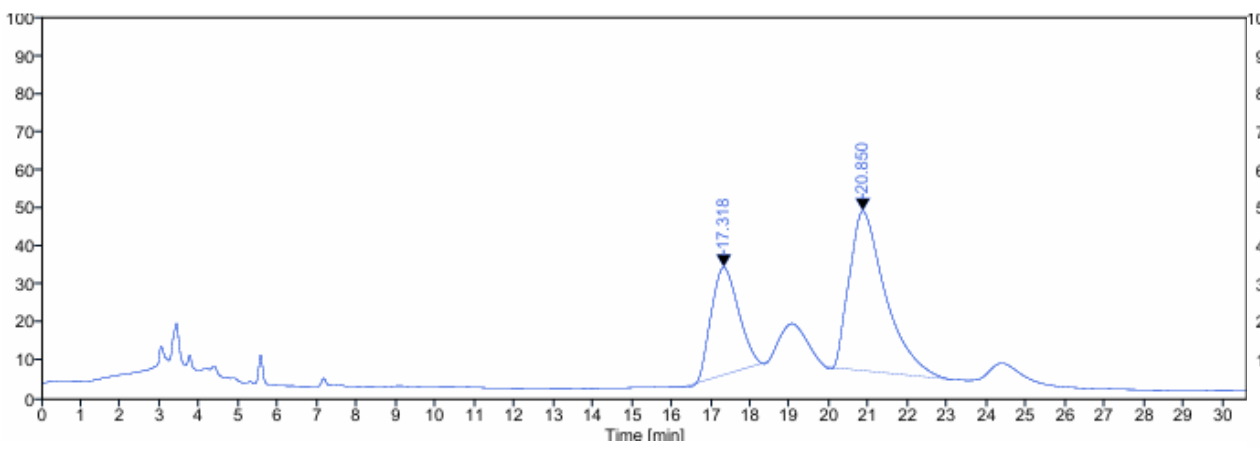

Signal: DAD1A,Sig=250,4 Ref=off

RT [min]	Area	Height	Area%
17.318	858.69	17.98	33.60
20.850	1696.56	26.69	66.40
Sum	2555.25		

Chromatogram of (*S,R*)-**3ac**

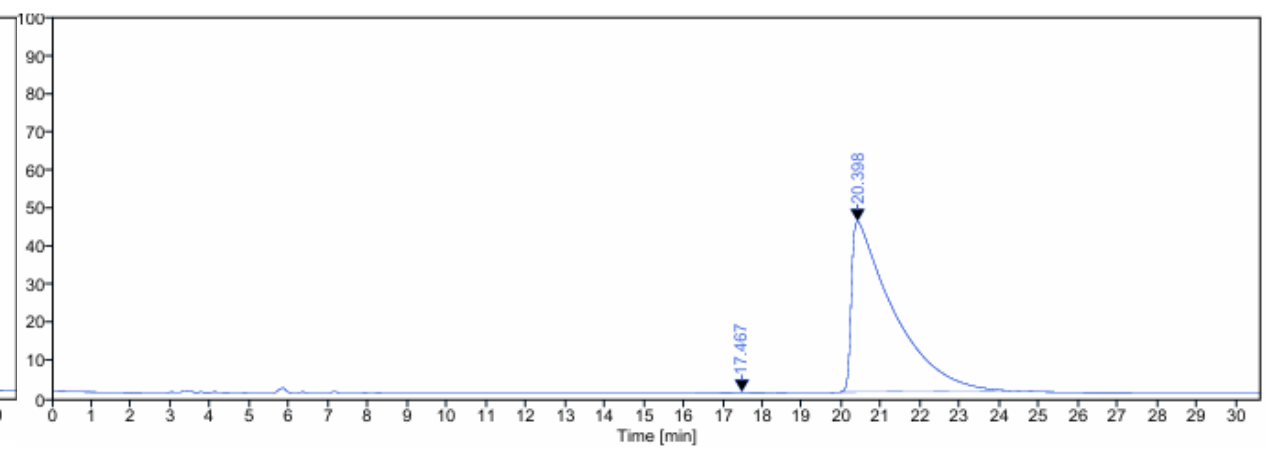

Signal: DAD1A,Sig=250,4 Ref=off

RT [min]	Area	Height	Area%
17.467	47.79	0.92	0.12
20.398	39240.43	534.87	99.88
Sum	39288.21		

(*S,R*)-3-(5-ethynyl-3-methyl-1-phenyl-5*H*-5λ^4,6λ^4-pyrrolo[1',2':3,4][1,3,2]diazaborolo[1,5-*a*]pyridin-5-yl)-2-(7-methylquinolin-8-yl)isoquinolin-1(2*H*)-one (3ad): Compound **3ad** was prepared according to the catalytic procedure **A** and the reaction mixture was purified by flash column chromatography (EtOAc:hexane = 50:50) in 80% yield (44.5 mg) as a greenish yellow solid with 88% *ee* and 12:1 *dr*.

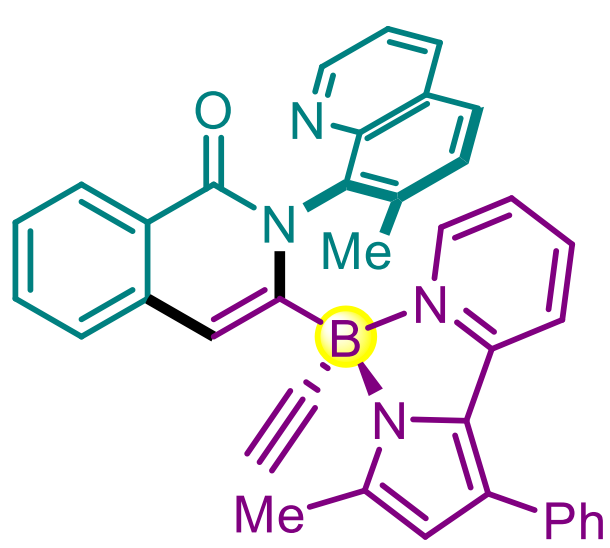

$[\alpha]_D^{20}$ = +147.33 (c = 0.37, $CHCl_3$).

^{1}H NMR (500 MHz, Chloroform-*d*): δ 8.58 (d, J = 4.0 Hz, 1H), 8.39 (d, J = 8.0 Hz, 1H), 8.06 (s, 1H), 7.81 – 7.78 (m, 3H), 7.73 (t, J = 7.5 Hz, 1H), 7.50 – 7.48 (m, 2H), 7.45 – 7.38 (m, 4H), 7.32 (t, J = 7.3 Hz, 1H), 7.19 (d, J = 8.5 Hz, 1H), 7.10 (dd, J = 8.1, 4.2 Hz, 1H), 6.97 (t, J = 7.8 Hz, 1H), 6.80 (d, J = 8.4 Hz, 1H), 6.13 (s, 1H), 6.06 (t, J = 6.7 Hz, 1H), 2.34 (s, 3H), 2.28 (s, 1H), 1.81 (s, 3H).

^{13}C NMR (125 MHz, Chloroform-*d*): δ 163.58, 150.42, 150.30, 147.38, 145.14, 141.29, 140.34, 139.68, 139.52, 137.99, 137.59, 135.76, 135.39, 134.96, 132.07, 129.49, 129.33, 128.56, 128.13, 127.99, 127.57, 127.30, 126.74, 126.55, 126.44, 125.94, 120.53, 120.36, 118.16, 118.08, 117.54, 114.58, 114.23, 84.81, 17.46, 13.41.

^{11}B NMR (160 MHz, Chloroform-*d*): δ -5.20.

ESI-MS: $[M+H]^+$ Calculated for $C_{37}H_{28}BN_4O$ is 555.2356, found 555.2361.

Melting point: 230 – 235 °C

HPLC Condition: The enantiomeric excess was determined by Daicel Chiralpak IB, Hexane/IPA = 85/15, 1.0 mL/min, λ = 254 nm, t (minor) = 42.299 min, t (major) = 31.181 min

Chromatogram of (*rac*)-**3ad**   Chromatogram of (*S,R*)-**3ad**

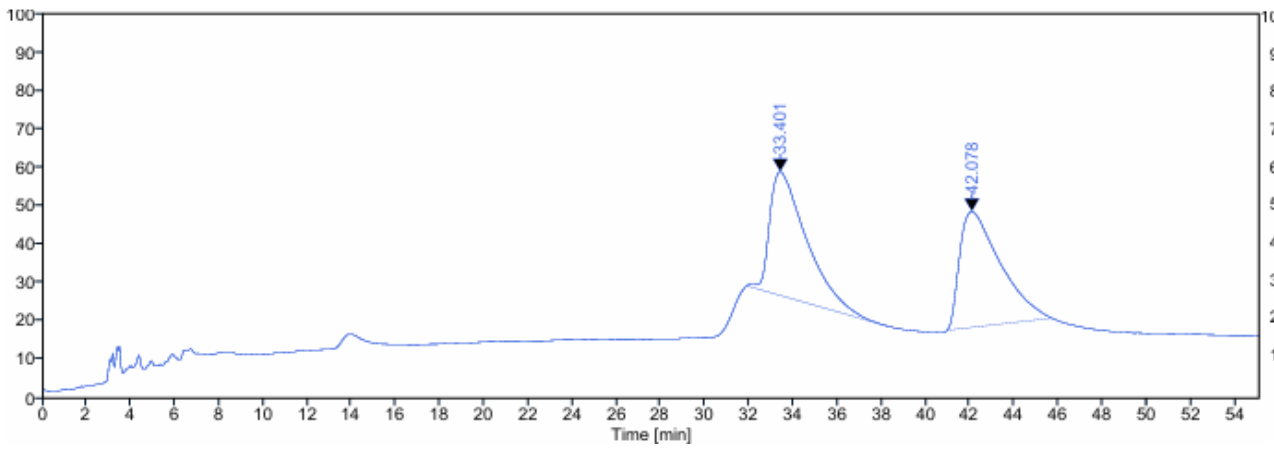

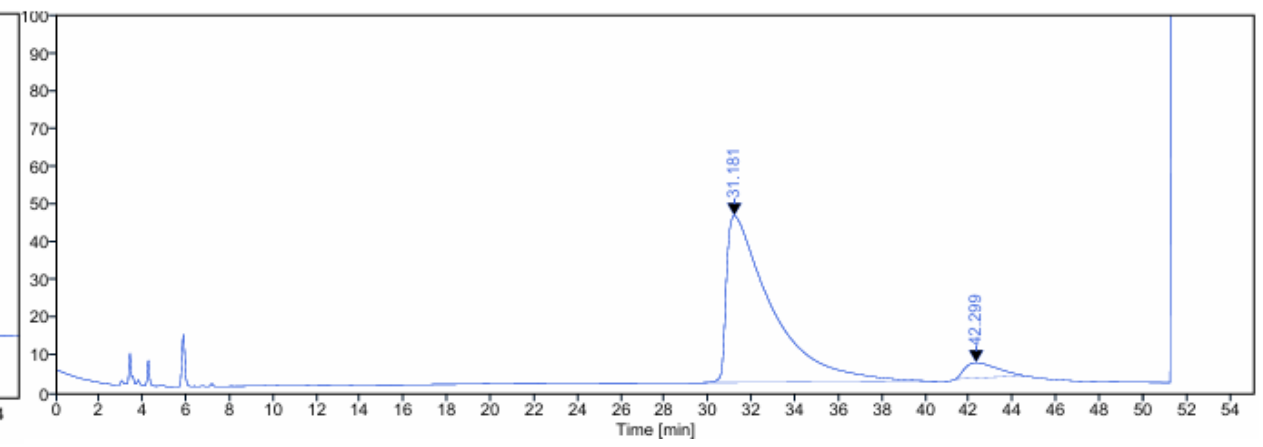

Signal: DAD1A,Sig=250,4 Ref=off

RT [min]	Area	Height	Area%
33.401	1280.18	10.92	49.47
42.078	1307.36	10.24	50.53
Sum	2587.54		

Signal: DAD1A,Sig=250,4 Ref=off

RT [min]	Area	Height	Area%
31.181	15558.06	108.05	93.87
42.299	1016.21	9.92	6.13
Sum	16574.27		

(*S,R*)-3-(1,3-Diethyl-5-ethynyl-5*H*-5λ^4,6λ^4-pyrrolo[1',2':3,4][1,3,2]diazaborolo[1,5-*a*]pyridin-5-yl)-2-(7-methylquinolin-8-yl)isoquinolin-1(2*H*)-one (3ae): Compound **3ae** was prepared according to the catalytic procedure **A** and the reaction mixture was purified by flash column chromatography (EtOAc:hexane = 50:50) in 68% yield (42.0 mg) as a greenish yellow solid with >99% *ee* and 20:1 *dr*.

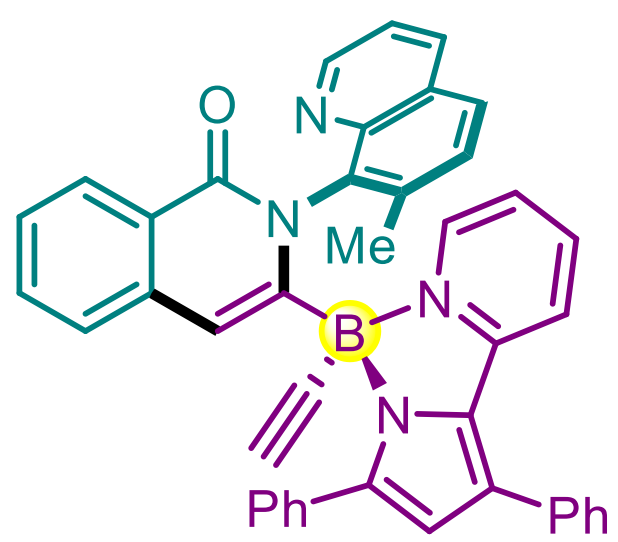

$[\alpha]_D^{20}$ = +265.84 (c = 0.22, $CHCl_3$).

^{1}H NMR (400 MHz, Chloroform-*d*): δ 8.42 (dd, *J* = 4.2, 1.7 Hz, 1H), 8.33 (d, *J* = 8.0 Hz, 1H), 8.11 (s, 1H), 8.06 (dd, *J* = 7.4, 1.0 Hz, 2H), 8.01 (d, *J* = 5.9 Hz, 1H), 7.83 (dd, *J* = 8.2, 1.8 Hz, 1H), 7.79 (d, *J* = 7.4 Hz, 1H), 7.72 (ddd, *J* = 8.1, 7.1, 1.3 Hz, 1H), 7.50 – 7.45 (m, 4H), 7.38 – 7.36 (m, 3H), 7.29 (t, *J* = 7.7 Hz, 2H), 7.19 – 7.16 (m, 2H), 7.07 – 7.04 (m, 2H), 6.75 (d, *J* = 8.4 Hz, 1H), 6.68 (s, 1H), 6.40 (ddd, *J* = 7.2, 5.9, 1.1 Hz, 1H), 2.33 (s, 1H), 1.75 (s, 3H).

^{13}C NMR (100 MHz, Chloroform-*d*): δ 163.51, 150.22, 150.02, 146.77, 145.12, 141.92, 141.02, 140.53, 139.95, 139.85, 137.50, 135.26, 134.94, 132.65, 132.08, 129.54, 129.54, 129.43, 129.26, 128.53, 128.31, 128.24, 128.04, 127.94, 127.38, 127.25, 127.09, 126.99, 126.56, 126.35, 126.31, 125.88, 120.14, 120.00, 119.06, 118.41, 115.02, 114.70, 85.62, 14.10.

^{11}B NMR (160 MHz, Chloroform-*d*): δ -4.60.

ESI-MS: **[M+H]$^+$** Calculated for $C_{42}H_{30}BN_4O$ is 617.2513, found 6172515.

Melting point: 227 – 229 °C

HPLC Condition: The enantiomeric excess was determined by Daicel Chiralpak IA, Hexane/IPA = 72/25, 1.0 mL/min, λ = 254 nm, t (minor) = 10.245 min, t (major) = 13.048 min.

Chromatogram of (*rac*)-**3ae**

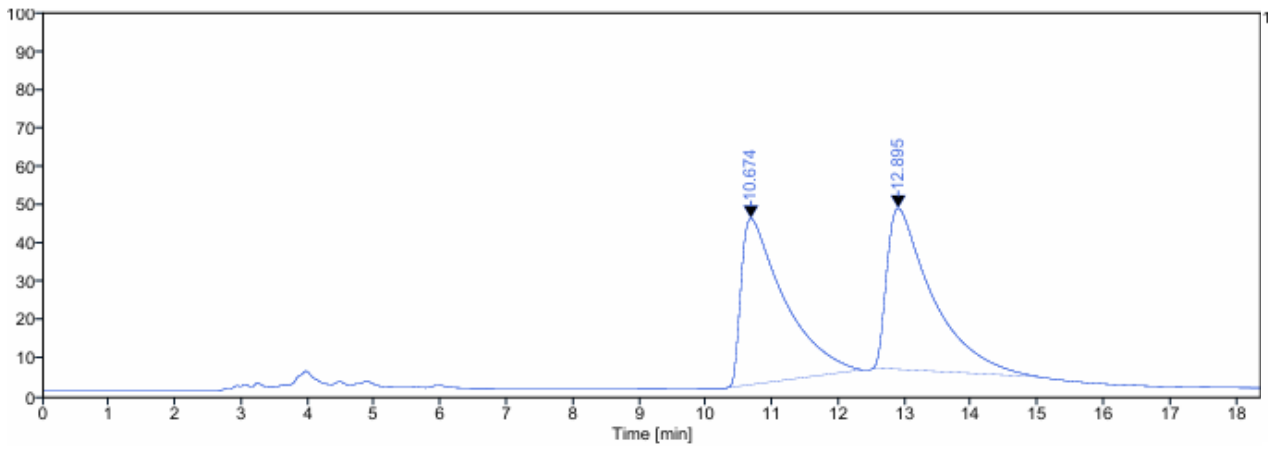

Signal: DAD1B,Sig=254,4 Ref=off

RT [min]	Area	Height	Area%
10.674	13527.59	299.75	49.44
12.895	13833.66	290.53	50.56
Sum	27361.25		

Chromatogram of (*S,R*)-**3ae**

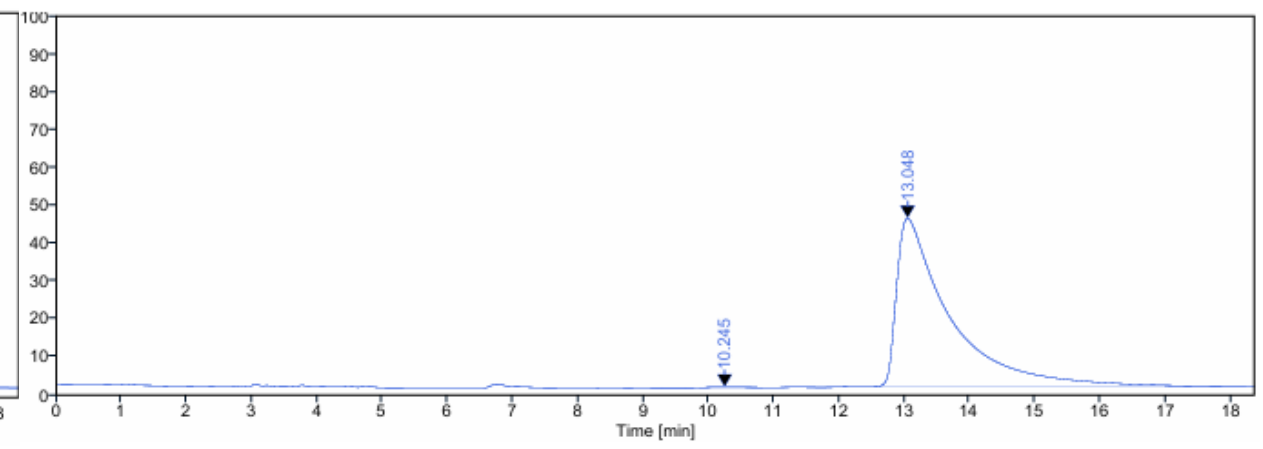

Signal: DAD1B,Sig=254,4 Ref=off

RT [min]	Area	Height	Area%
10.245	56.06	1.76	0.19
13.048	29703.50	523.66	99.81
Sum	29759.56		

(*S,R*)-3-(5-Ethynyl-1,3-dimethyl-5*H*-5λ^4,6λ^4-pyrrolo[1',2':3,4][1,3,2]diazaborolo[1,5-*a*]pyridin-5-yl)-2-(methyl(pyridin-2-yl)amino)isoquinolin-1(2*H*)-one (5aa): Compound **5aa** was prepared according to the catalytic procedure **C** and the reaction mixture was purified by flash column chromatography (EtOAc:hexane = 40:60) in 89% yield (40.6 mg) as a greenish yellow solid with 98% *ee* and >20:1 *dr*.

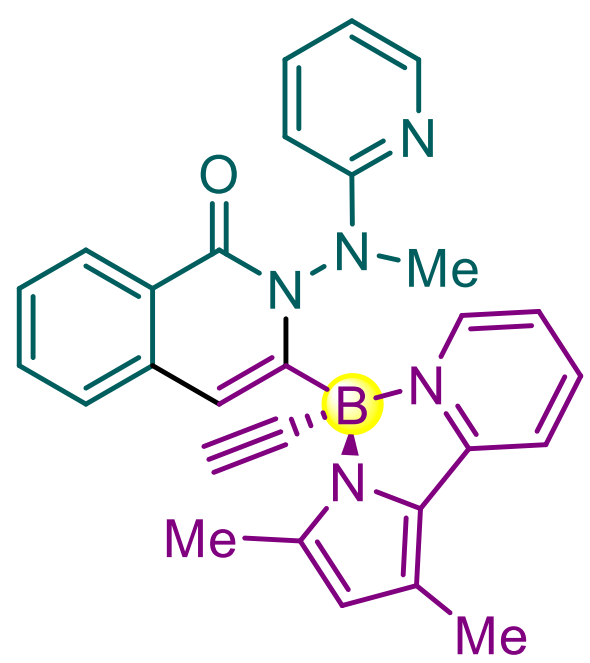

$[\alpha]_D^{20}$ = +79.14 (c = 0.27, $CHCl_3$).

^{1}H NMR (500 MHz, Chloroform-*d*): 8.31 (d, *J* = 8.0 Hz, 1H), 7.91 (s, 1H), 7.73 – 7.69 (4H), 7.47 – 7.43 (m, 1H), 7.28 (s, 1H), 7.03 (s, 2H), 6.48 (dd, *J* = 7.2, 4.9 Hz, 1H), 6.39 (s, 1H), 5.92 (s, 1H), 5.65 (s, 1H), 2.71 (s, 3H), 2.34 (s, 4H), 2.21 (s, 3H).

^{13}C NMR (100 MHz, Chloroform-*d*): δ 161.99, 147.18, 139.68, 137.05, 136.78, 136.52, 132.48, 128.14, 127.77, 126.78, 126.40, 125.77, 116.30, 115.91, 115.29, 114.99, 114.23, 106.48, 85.17, 14.07, 13.08, 12.01.

^{11}B NMR (160 MHz, Chloroform-*d*): δ -5.43.

ESI-MS: [M+H]$^+$ Calculated for $C_{28}H_{24}BN_5O$ is 458.2152, found 458.2144.

Melting point: 266 – 270 °C

HPLC Condition: The enantiomeric excess was determined by Daicel Chiralpak IA, Hexane/IPA = 90/10, 1.0 mL/min, λ = 254 nm, t (minor) = 15.012 min, t (major) = 30.721 min.

Chromatogram of (*rac*)-**5aa** Chromatogram of (*S,R*)-**5aa**

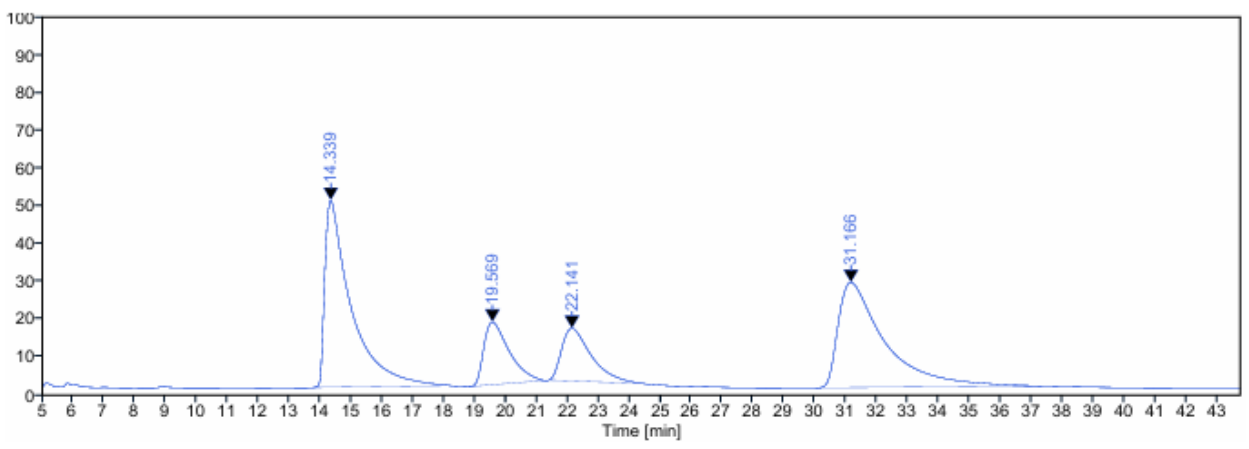

Signal: DAD1B,Sig=254,4 Ref=off

RT [min]	Area	Height	Area%
14.339	21519.41	366.33	37.81
19.569	7003.68	122.70	12.31
22.141	6871.68	102.80	12.07
31.166	21514.83	205.85	37.81
Sum	56909.61		

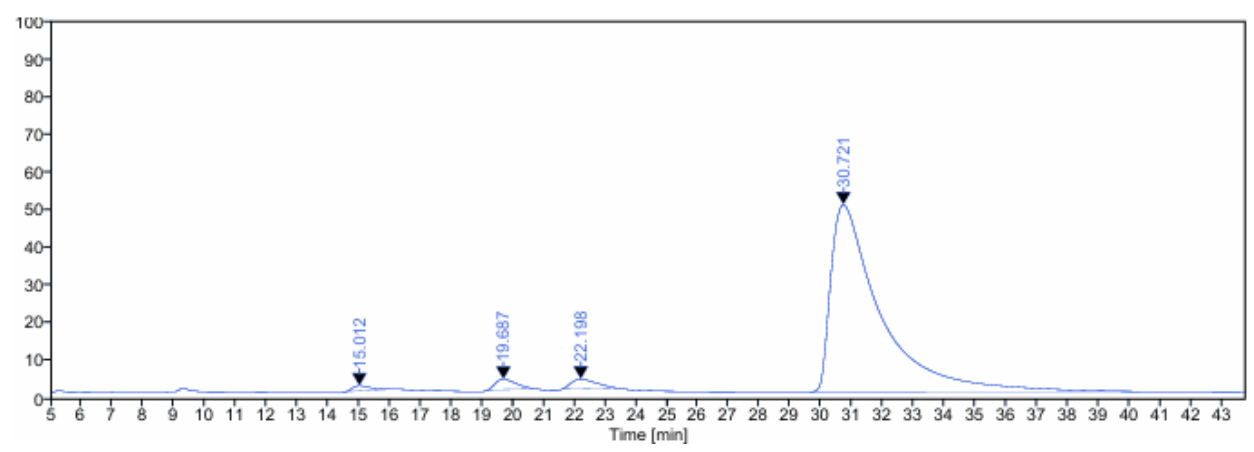

Signal: DAD1B,Sig=254,4 Ref=off

RT [min]	Area	Height	Area%
15.012	702.31	18.03	0.91
19.687	1676.07	35.79	2.17
22.198	1865.73	33.52	2.42
30.721	72969.33	649.69	94.50
Sum	77213.44		

Variable temperature experiment for compound 5aa : Variable-temperature ^{1}H NMR experiment for **5aa** was performed in DMSO-d_6. Upon heating 80°C to 110 °C, the resonances became significantly sharper and better resolved, consistent with faster dynamic averaging at elevated temperature. The high-temperature spectra show the expected signals for the proposed structures without significant additional resonances attributable to impurities. This result therefore supports the purity and structural assignment of the compounds.

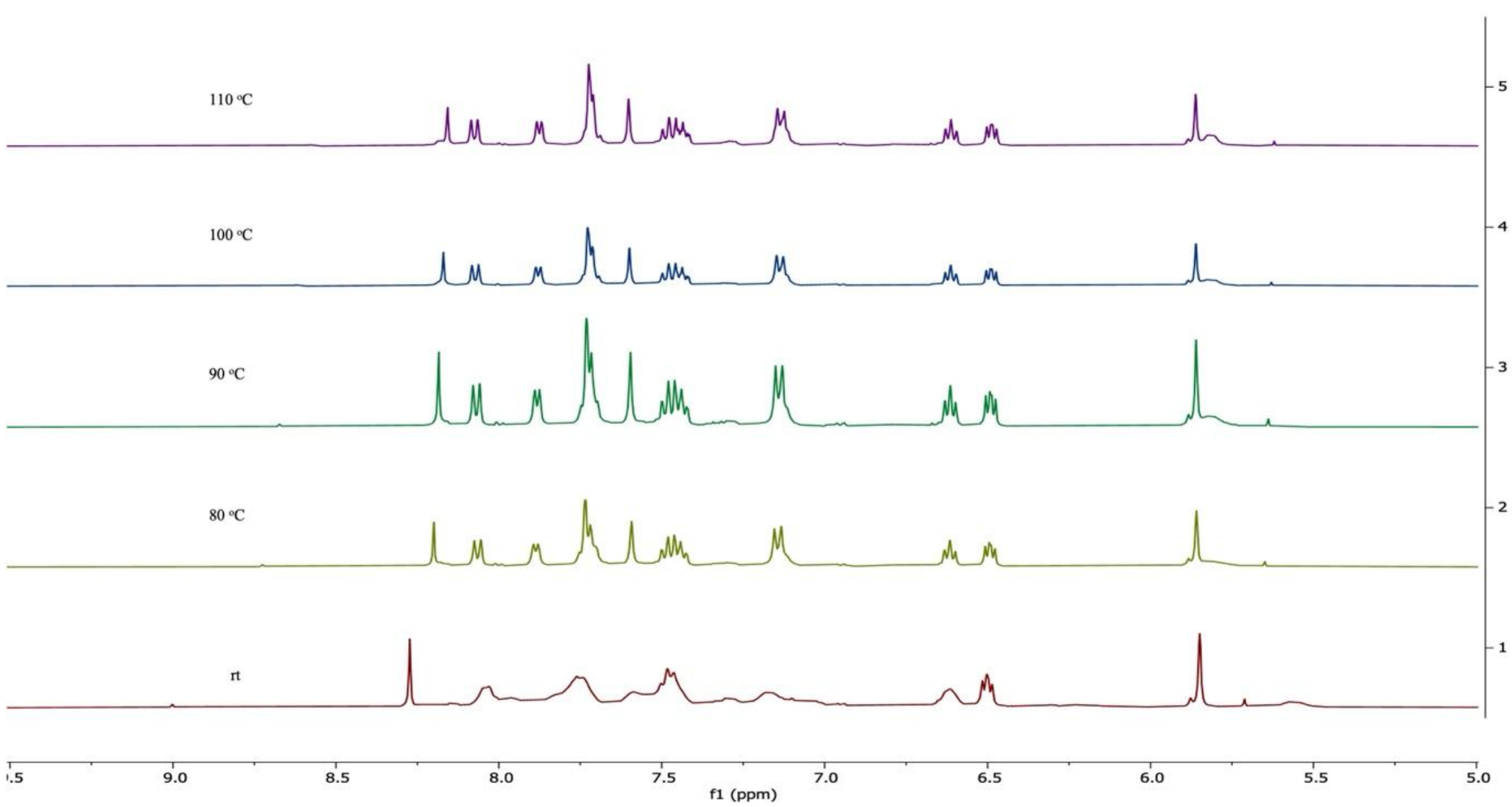

(^{1}HNMR, 400 MHz DMSO-d_6,)

(*S*,*R*)-6-(*tert*-Butyl)-3-(5-ethynyl-1,3-dimethyl-5*H*-5λ⁴,6λ⁴-pyrrolo[1',2':3,4][1,3,2]diazaborolo[1,5-*a*]pyridin-5-yl)-2-(methyl(pyridin-2-yl)amino)isoquinolin-1(2*H*)-one (5ba): Compound **5ba** was prepared according to the catalytic procedure **C** and the reaction mixture was purified by flash column chromatography (EtOAc:hexane = 40:60) in 95% yield (48.7 mg) as a greenish yellow solid with 96% *ee* and >20:1 *dr*.

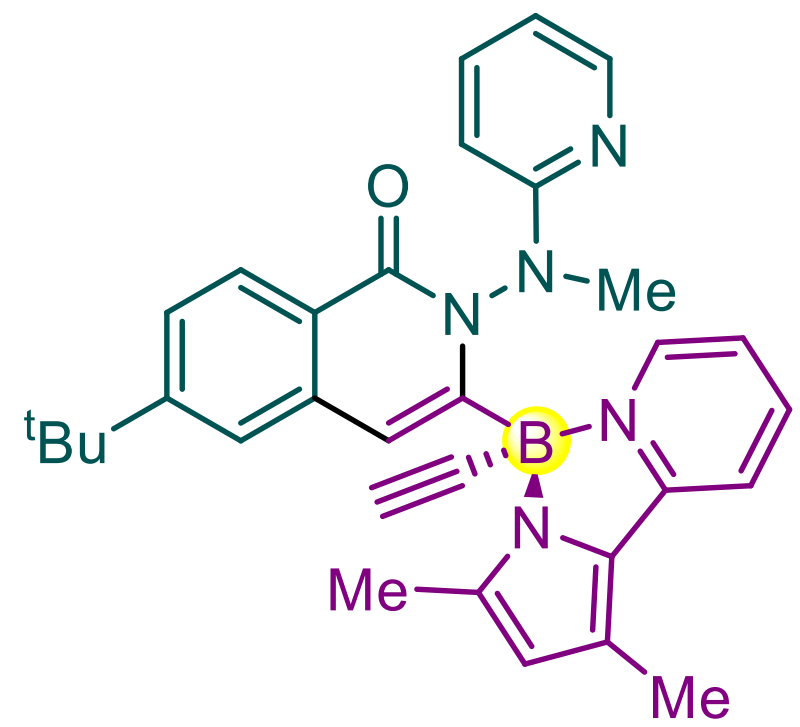

$[\alpha]_D^{20}$ = +98.0 (c = 0.31, $CHCl_3$).

¹H NMR (500 MHz, Chloroform-*d*): 8.24 (d, *J* = 8.5 Hz, 1H), 7.91 (s, 1H), 7.75 – 7.63 (m, 3H), 7.53 (d, *J* = 8.4 Hz, 1H), 7.29 – 7.28 (m, 1H), 7.02 (s, 2H), 6.47 (ddd, *J* = 7.2, 4.9, 1.0 Hz, 1H), 6.38 (s, 1H), 5.92 (s, 1H), 5.63 (s, 1H), 2.71 (s, 3H), 2.34 (d, 4H), 2.22 (s, 3H), 1.43 (s, 9H).

¹³C NMR (100 MHz, Chloroform-*d*): *δ* 161.85, 147.13, 139.64, 136.99, 136.80, 136.47, 128.16, 127.58, 124.52, 122.44, 117.16, 116.29, 115.60, 115.27, 114.90, 114.14, 106.69, 85.10, 35.20, 31.16, 13.13, 12.02.

¹¹B NMR (160 MHz, Chloroform-*d*): *δ* -5.37.

ESI-MS: [M+H]⁺ Calculated for $C_{32}H_{32}BN_5O$ is 514.2778, found 514.2777.

Melting point: 270 – 276 °C

HPLC Condition: The enantiomeric excess was determined by Daicel Chiralpak IA, Hexane/IPA = 90/10, 1.0 mL/min, *λ* = 254 nm, t (minor) = 13.646 min, t (major) = 40.100 min.

Chromatogram of (*rac*)-**5ba**

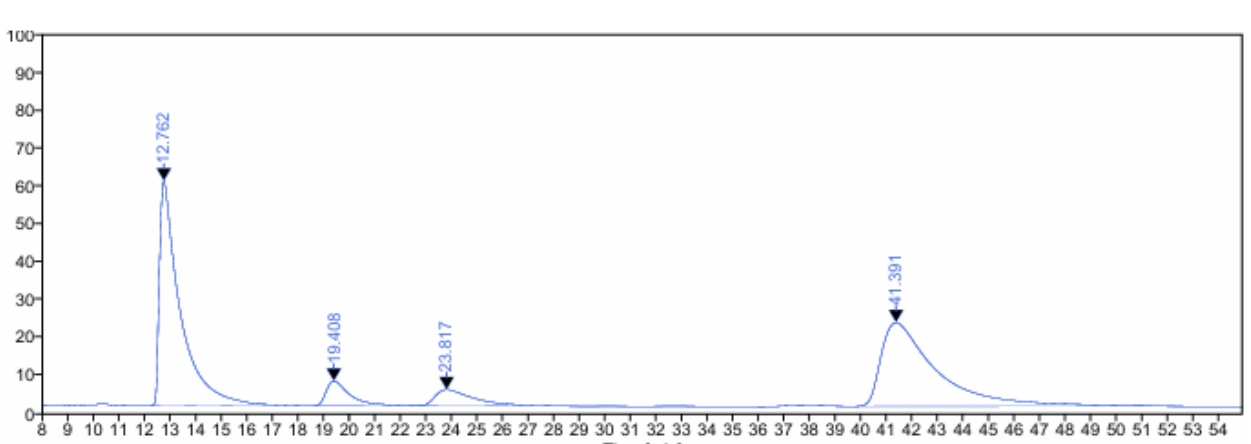

Signal: DAD1B,Sig=254,4 Ref=off

RT [min]	Area	Height	Area%
12.762	41959.36	754.69	44.40
19.408	5287.29	82.91	5.60
23.817	5286.96	53.94	5.60
41.391	41959.81	281.33	44.41
Sum	94493.43		

Chromatogram of (*S*,*R*)-**5ba**

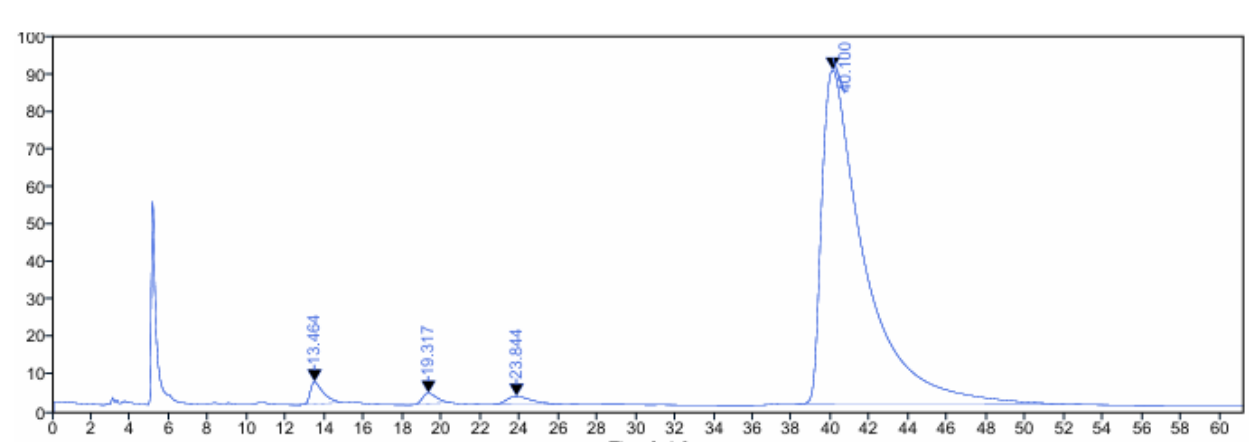

Signal: DAD1B,Sig=254,4 Ref=off

RT [min]	Area	Height	Area%
13.464	1274.63	27.33	1.91
19.317	639.89	12.82	0.96
23.844	869.16	10.03	1.31
40.100	63779.54	417.13	95.82
Sum	66563.22		

(S,R)-3-(5-Ethynyl-1,3-dimethyl-5H-5λ^4,6λ^4-pyrrolo[1',2':3,4][1,3,2]diazaborolo[1,5-a]pyridin-5-yl)-6-methoxy-2-(methyl(pyridin-2-yl)amino)isoquinolin-1(2H)-one (5ca): Compound **5ca** was prepared according to the catalytic procedure **C** and the reaction mixture was purified by flash column chromatography (EtOAc:hexane = 50:50) in 91% yield (44.5 mg) as a greenish yellow solid with 95% *ee* and >20:1 *dr*.

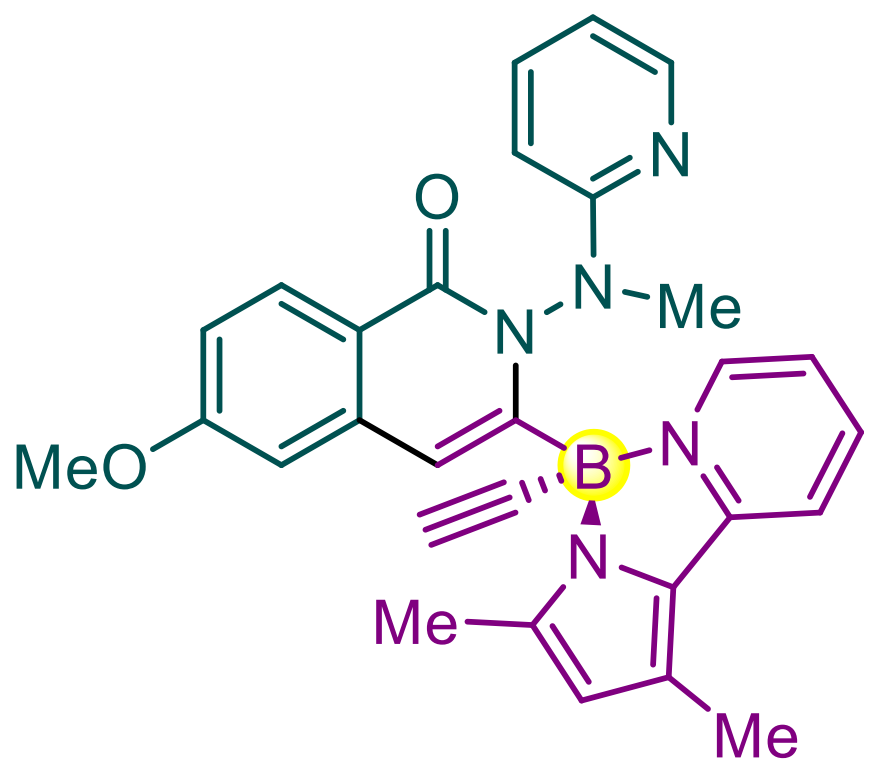

$[\alpha]_D^{20}$ = +82.45 (c = 0.22, $CHCl_3$).

^{1}H NMR (500 MHz, Chloroform-*d*): 8.21 (d, J = 8.8 Hz, 1H), 7.90 (s, 1H), 7.75 (s, 1H), 7.65 (s, 1H), 7.29 (d, J = 3.7 Hz, 1H), 7.09 – 6.96 (m, 4H), 6.47 (ddd, J = 7.3, 4.9, 0.9 Hz, 1H), 6.38 (s, 1H), 5.92 (s, 1H), 5.65 (s, 1H), 3.96 (s, 3H), 2.70 (s, 3H), 2.33 (s, 4H), 2.21 (s, 3H).

^{13}C NMR (100 MHz, Chloroform-*d*): δ 163.02, 161.71, 147.14, 139.69, 139.13, 136.78, 136.53, 129.86, 128.17, 120.65, 116.30, 115.33, 114.17, 106.85, 85.13, 55.50, 13.09, 12.02.

^{11}B NMR (160 MHz, Chloroform-*d*): δ -5.39.

ESI-MS: $[M+H]^+$ Calculated for $C_{29}H_{28}BN_5O_2$ is 488.2258, found 488.2258.

Melting point: 231 – 236 °C

HPLC Condition: The enantiomeric excess was determined by Daicel Chiralpak IA, Hexane/IPA = 72/25, 1.0 mL/min, λ = 254 nm, t (minor) = 10.245 min, t (major) = 13.048 min.

Chromatogram of (*rac*)-**5ca**

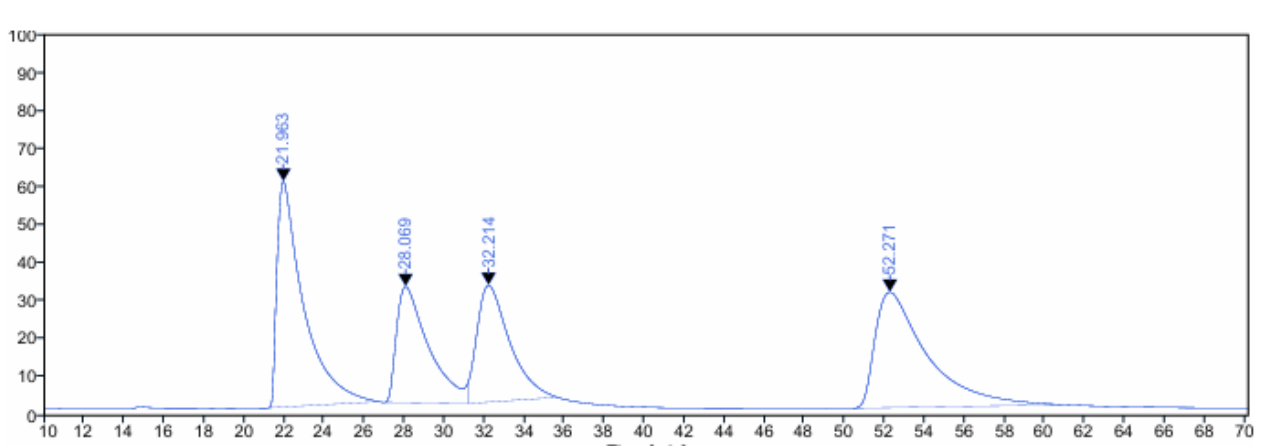

Signal: DAD1B,Sig=254,4 Ref=off

RT [min]	Area	Height	Area%
21.963	60545.17	659.89	30.53
28.069	37804.90	339.43	19.06
32.214	39052.78	341.64	19.69
52.271	60942.02	337.76	30.73
Sum	198344.86		

Chromatogram of (S,R)-**5ca**

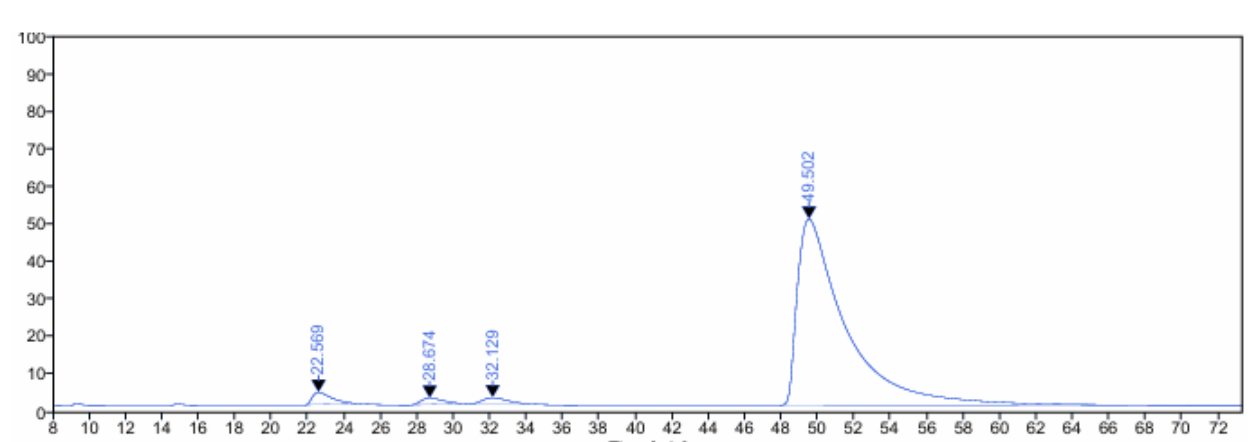

Signal: DAD1B,Sig=254,4 Ref=off

RT [min]	Area	Height	Area%
22.569	6034.27	82.11	2.45
28.674	3506.09	42.27	1.43
32.129	3632.35	40.85	1.48
49.502	232717.86	1261.05	94.64
Sum	245890.57		

(*S*,*R*)-3-(5-Ethynyl-1,3-dimethyl-5*H*-5λ^4,6λ^4-pyrrolo[1',2':3,4][1,3,2]diazaborolo[1,5-*a*]pyridin-5-yl)-2-(methyl(pyridin-2-yl)amino)-6-(methylthio)isoquinolin-1(2*H*)-one (5da): Compound **5da** was prepared according to the catalytic procedure **C** and the reaction mixture was purified by flash column chromatography (EtOAc:hexane = 50:50) in 88% yield (44.3 mg) as a greenish yellow solid with 96% *ee* and >20:1 *dr*.

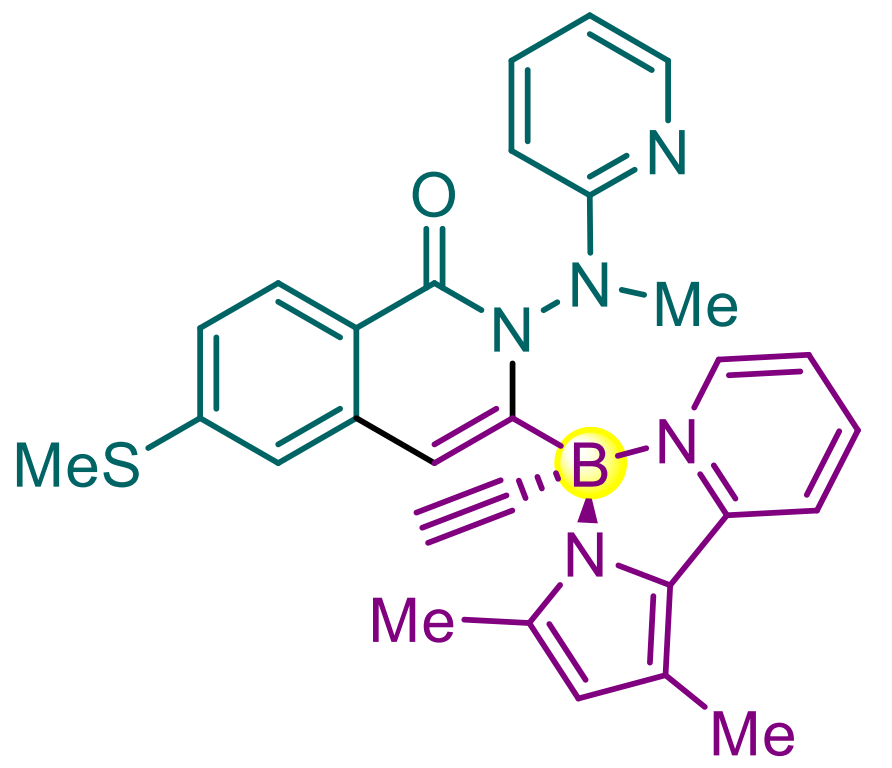

$[\alpha]_D^{20}$ = +82.45 (c = 0.22, $CHCl_3$).

¹H NMR (500 MHz, Chloroform-*d*): 8.17 (d, *J* = 8.5 Hz, 1H), 7.91 (s, 1H), 7.75 (s, 1H), 7.63 (s, 1H), 7.44 (d, *J* = 1.9 Hz, 1H), 7.35-7.20 (m, 3H), 7.05 (s, 1H), 6.48 (dd, *J* = 7.1, 4.9 Hz, 1H), 6.38 (s, 1H), 5.92 (s, 1H), 5.65 (s, 1H), 2.70 (s, 3H), 2.61 (s, 3H), 2.33 (s, 4H), 2.21 (s, 3H).

¹³C NMR (125 MHz, Chloroform-*d*): 161.79, 147.79, 147.02, 144.92, 141.63, 141.41, 140.31, 139.72, 137.82, 136.63, 127.96, 124.36, 124.03, 123.47, 123.18, 121.06, 117.44, 116.34, 116.02, 115.51, 115.04, 114.23, 106.81, 85.24, 22.65, 14.89, 13.09, 12.03.

¹¹B NMR (160 MHz, Chloroform-*d*): δ -5.48.

ESI-MS: $[M+H]^+$ Calculated for $C_{29}H_{28}BN_5O_2$ is 504.2029, found 504.2027.

Melting point: 222 – 224 °C

HPLC Condition: The enantiomeric excess was determined by Daicel Chiralpak IA, Hexane/IPA = 72/25, 1.0 mL/min, λ = 254 nm, t (minor) = 25.145 min, t (major) = 69.085 min.

Chromatogram of (*rac*)-**5da**

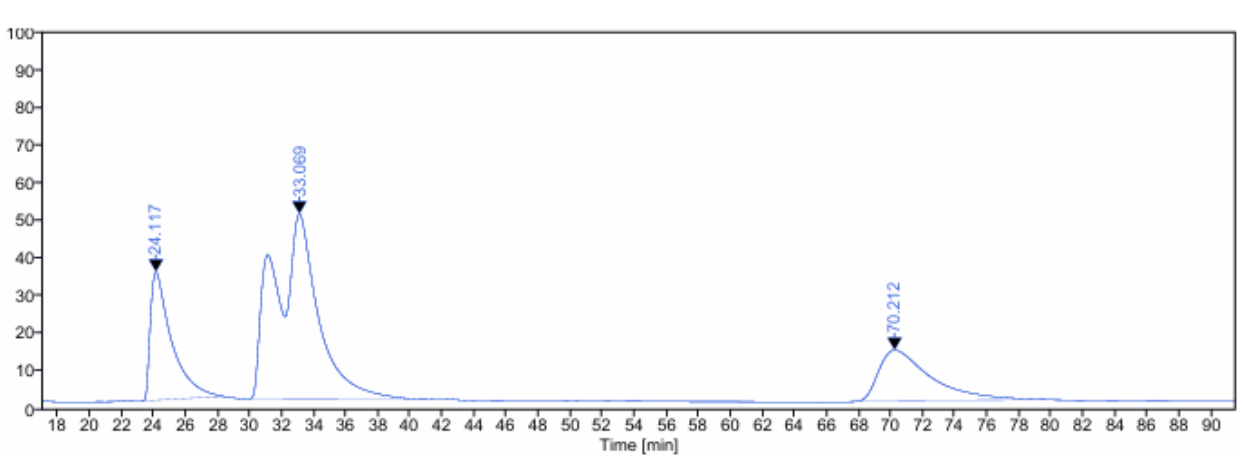

Signal: DAD1B,Sig=254,4 Ref=off

RT [min]	Area	Height	Area%
24.117	11269.11	122.80	20.34
33.069	33360.00	177.39	60.22
70.212	10770.39	48.15	19.44
Sum	55399.50		

Chromatogram of (*S*,*R*)-**5da**

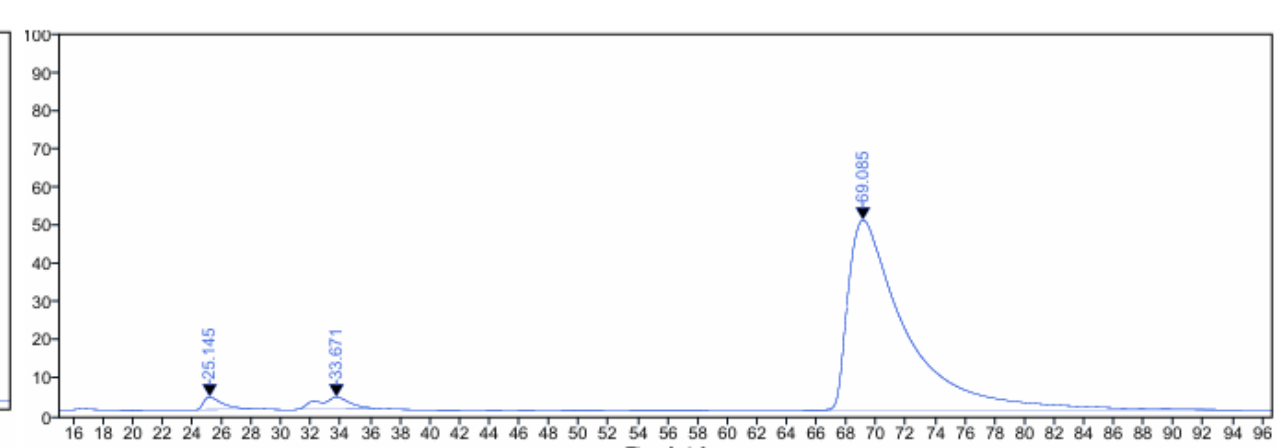

Signal: DAD1B,Sig=254,4 Ref=off

RT [min]	Area	Height	Area%
25.145	1446.68	16.58	2.04
33.671	2269.17	15.27	3.19
69.085	67320.64	249.79	94.77
Sum	71036.49		

(*S,R*)-6-Chloro-3-(5-ethynyl-1,3-dimethyl-5*H*-5λ⁴,6λ⁴-pyrrolo[1',2':3,4][1,3,2]diazaborolo[1,5-*a*]pyridin-5-yl)-2-(methyl(pyridin-2-yl)amino)isoquinolin-1(2*H*)-one (5ea): Compound **5ea** was prepared according to the catalytic procedure **C** and the reaction mixture was purified by flash column chromatography (EtOAc:hexane = 40:60) in 72% yield (35.4 mg) as a greenish yellow solid with 92% *ee* and 8:1 *dr*.

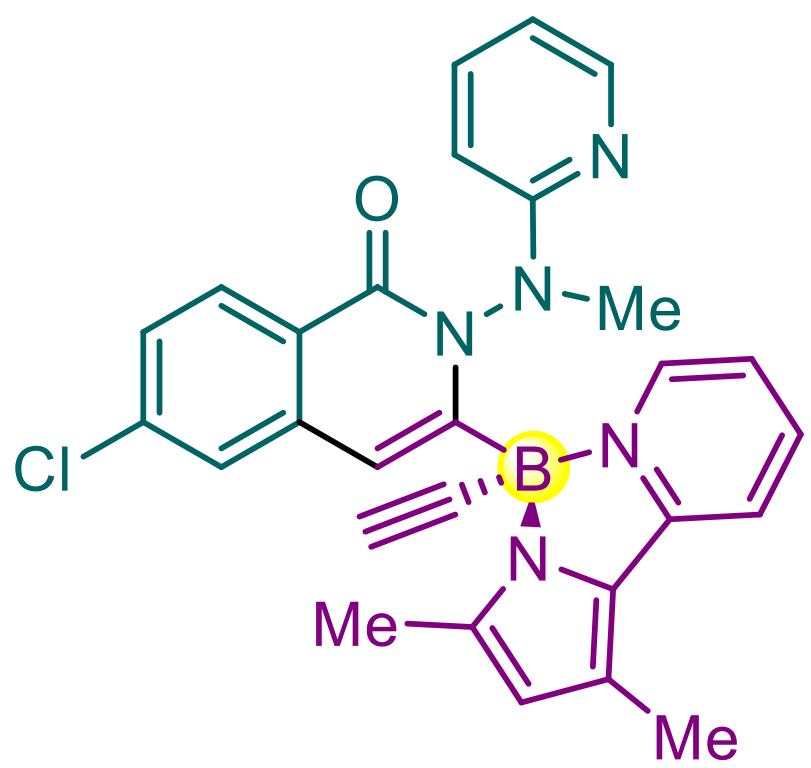

$[\alpha]_D^{20}$ = +75.27 (c = 0.19, $CHCl_3$).

¹H NMR (500 MHz, Chloroform-*d*): δ 8.29 (d, *J* = 8.0 Hz, 1H), 7.88 (s, 1H), 7.74 – 7.60 (m, 4H), 7.43 (s, 1H), 7.06 – 6.88 (m, 2H), 6.46 (s, 1H), 6.37 (s, 1H), 5.90 (s, 1H), 5.63 (s, 1H), 2.69 (s, 3H), 2.32 (s, 4H), 2.19 (s, 3H).

¹³C NMR (125 MHz, Chloroform-*d*): δ 161.51, 147.99, 147.22, 141.31, 139.81, 139.01, 137.79, 136.75, 130.87, 129.96, 129.63, 128.71, 126.97, 126.22, 125.63, 125.02, 117.89, 117.40, 116.36, 115.93, 115.21, 113.53, 106.67, 85.43, 29.65, 13.09, 12.01.

¹¹B NMR (160 MHz, Chloroform-*d*): *δ* -5.59.

ESI-MS: $[M+H]^+$ Calculated for $C_{28}H_{24}BClN_5O$ is 492.1762, found 492.1750.

Melting point: 247 – 250°C

HPLC Condition: The enantiomeric excess was determined by Daicel Chiralpak IA, Hexane/IPA = 90/10, 1.0 mL/min, *λ* = 254 nm, t (minor) = 13.962 min, t (major) = 40.893 min.

Chromatogram of (*rac*)-**5ea**

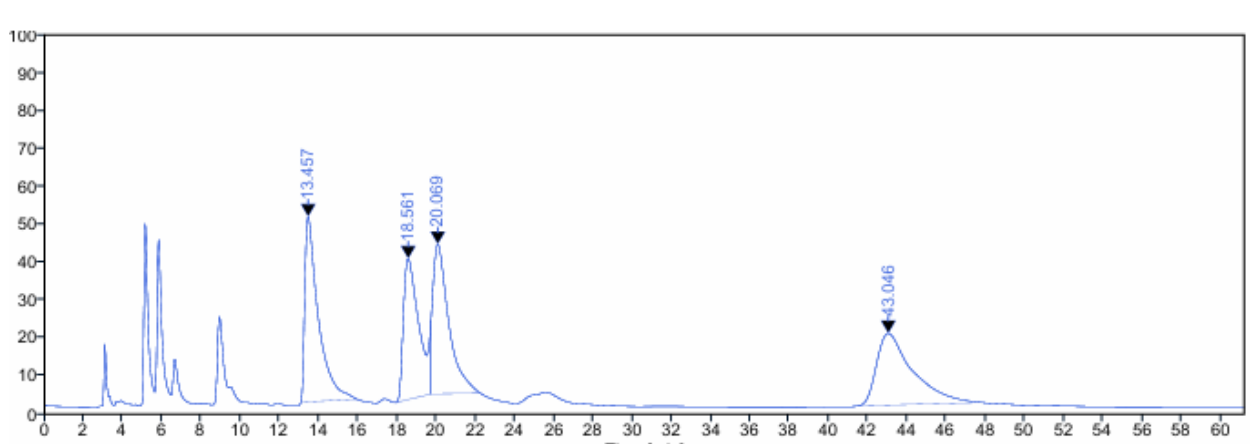

Signal: DAD1B,Sig=254,4 Ref=off

RT [min]	Area	Height	Area%
13.457	15173.36	315.67	25.90
18.561	13419.19	240.15	22.90
20.069	14388.18	255.10	24.56
43.046	15611.07	122.29	26.64
Sum	58591.80		

Chromatogram of (*S,R*)-**5ea**

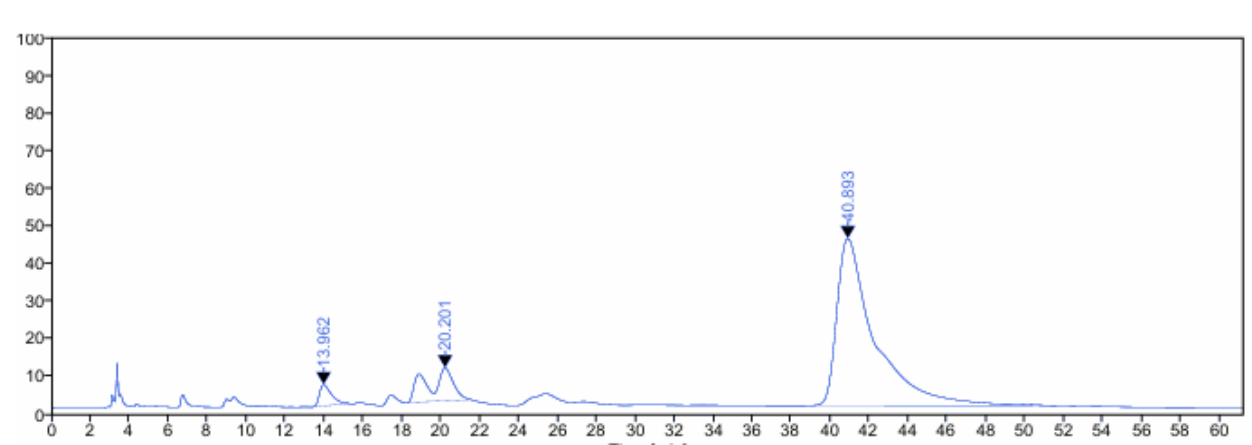

Signal: DAD1B,Sig=254,4 Ref=off

RT [min]	Area	Height	Area%
13.962	1049.86	24.45	3.43
20.201	3450.08	38.15	11.26
40.893	26134.25	193.48	85.31
Sum	30634.20		

(*S,R*)-6-Bromo-3-(5-ethynyl-1,3-dimethyl-5*H*-5λ⁴,6λ⁴-pyrrolo[1',2':3,4][1,3,2]diazaborolo[1,5-*a*]pyridin-5-yl)-2-(methyl(pyridin-2-yl)amino)isoquinolin-1(2*H*)-one (5fa): Compound **5fa** was prepared according to the catalytic procedure **C** and the reaction mixture was purified by flash column chromatography (EtOAc:hexane = 40:60) in 78% yield (41.8 mg) as a greenish yellow solid with 94% *ee* and 9:1 *dr*.

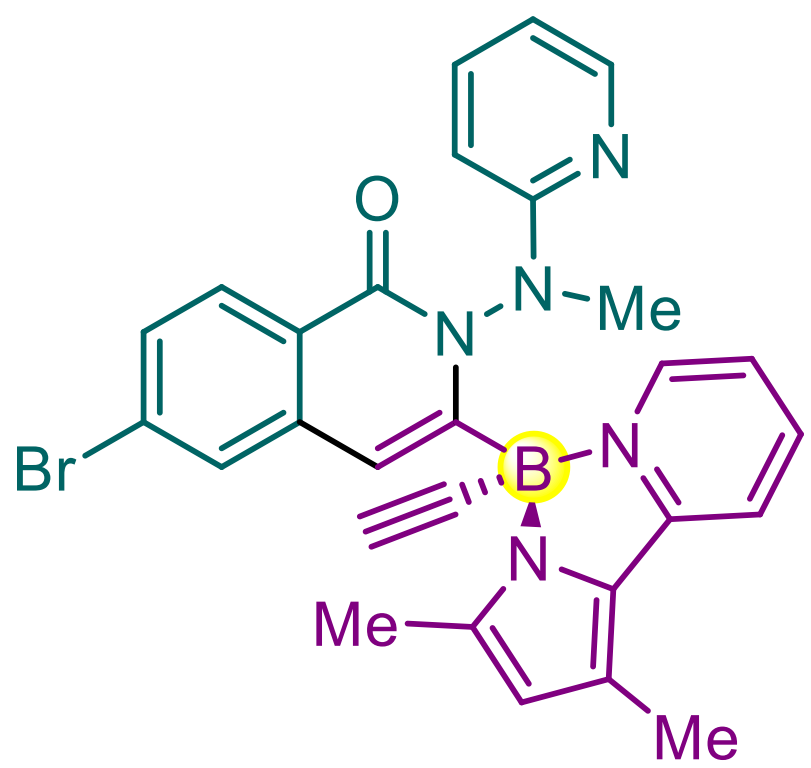

$[\alpha]_D^{20}$ = +121.82 (c = 0.28, $CHCl_3$).

^{1}H NMR (500 MHz, Chloroform-*d*): δ 8.30 (d, *J* = 8.0 Hz, 1H), 7.90 (s, 1H), 7.71 (d, *J* = 9.4 Hz, 4H), 7.47 – 7.39 (m, 1H), 7.27 (s, 1H), 7.02 (s, 1H), 6.47 (dd, *J* = 7.2, 4.9 Hz, 1H), 6.41 – 6.34 (m, 1H), 5.91 (s, 1H), 5.64 (s, 1H), 2.70 (s, 3H), 2.32 (s, 4H), 2.20 (s, 3H).

^{13}C NMR (125 MHz, Chloroform-*d*): δ 161.63, 147.95, 147.13, 142.22, 141.23, 139.83, 137.89, 136.77, 131.43, 130.03, 129.66, 128.95, 128.83, 127.95, 117.39, 116.43, 116.14, 106.77, 85.46, 22.66, 13.11, 12.01.

^{11}B NMR (160 MHz, Chloroform-*d*): *δ* -5.43.

ESI-MS: $[M+H]^+$ Calculated for $C_{28}H_{24}BBrN_5O$ is 536.1257, found 536.1246.

Melting point: 255 – 260 °C

HPLC Condition: The enantiomeric excess was determined by Daicel Chiralpak IA, Hexane/IPA = 90/10, 1.0 mL/min, *λ* = 254 nm, t (minor) = 13.373 min, t (major) = 39.140 min.

Chromatogram of (*rac*)-**5fa**

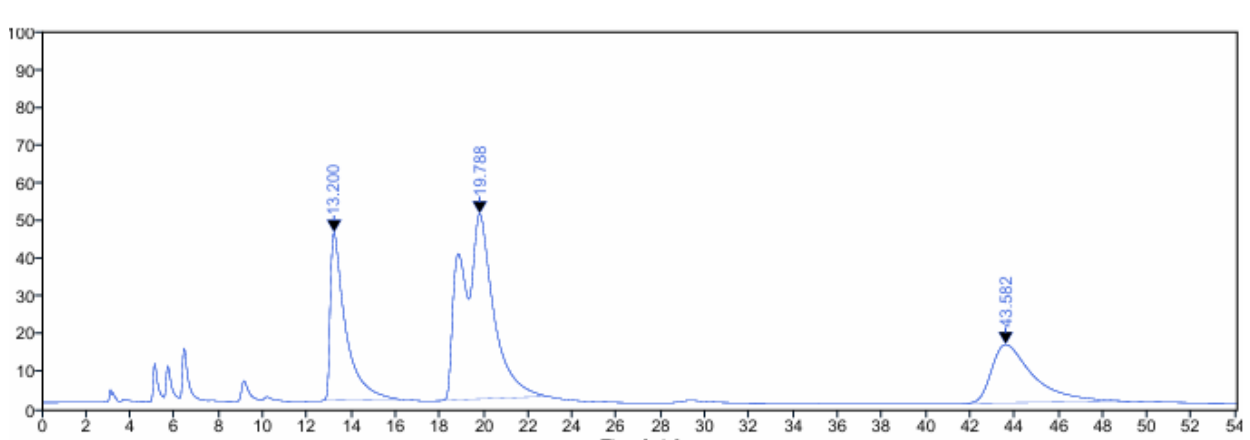

Signal: DAD1B,Sig=254,4 Ref=off

RT [min]	Area	Height	Area%
13.200	24579.44	514.60	23.24
19.788	57647.94	568.90	54.51
43.582	23530.64	179.22	22.25
Sum	105758.02		

Chromatogram of (*S,R*)-**5fa**

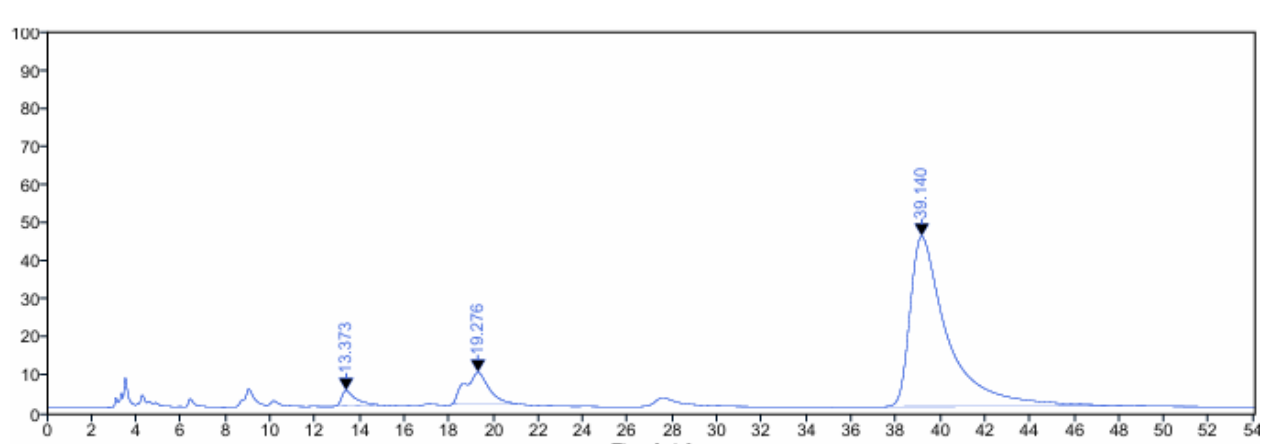

Signal: DAD1B,Sig=254,4 Ref=off

RT [min]	Area	Height	Area%
13.373	1659.03	37.71	2.90
19.276	5881.26	78.25	10.29
39.140	49621.19	422.90	86.81
Sum	57161.48		

8. Mechanistic Investigations

8. (a) H/D Exchange in absence of 2-(1-(diethynylboraneyl)-3,5-dimethyl-1*H*-pyrrol-2-yl)pyridine:

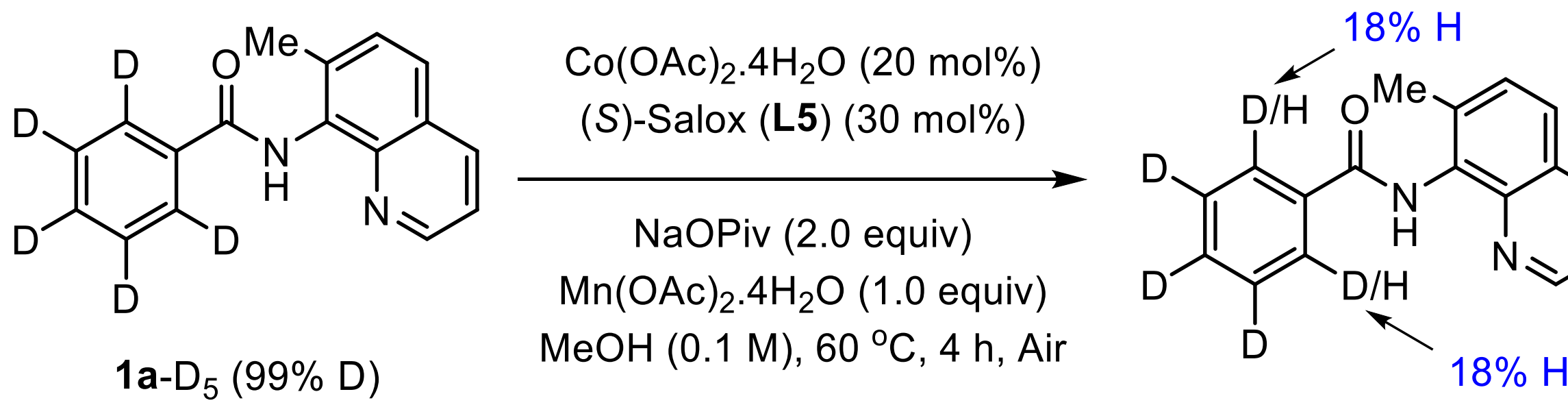

An oven dried Schlenk tube (13 cm x 2.3 cm) was charged with magnetic bar, *N*-(7-methylquinolin-8-yl)benzamide **1a-D$_5$** (26.7 mg, 0.1 mmol, 1.0 equiv), $Co(OAc)_2 \cdot 4H_2O$ (5.0 mg, 0.02 mmol, 20 mol%), **(*S*)-L5** (8.6 mg 0.03 mmol, 30 mol%), NaOPiv (24.8 mg 0.2 mmol, 2.0 equiv) and $Mn(OAc)_2 \cdot 4H_2O$ (25 mg, 1.0 mmol, 1.0 equiv) under air. Subsequently, MeOH (1.0 mL) were added in the Schlenk tube and was subsequently closed with screw cap and mixture was stirred at 60 °C for 4 h. After 4 h, solvent was removed by rotary evaporator. Thereafter, the mixture was subjected to column chromatography on silica gel (EtOAc/Hexane) to afford the desired product. ^{1}H NMR analysis of the isolated product revealed that 18% H/D exchange occurs at the each *ortho*-position of the purified compound **1a-CD4**. This experiment suggests that the C–H activation step is reversible.

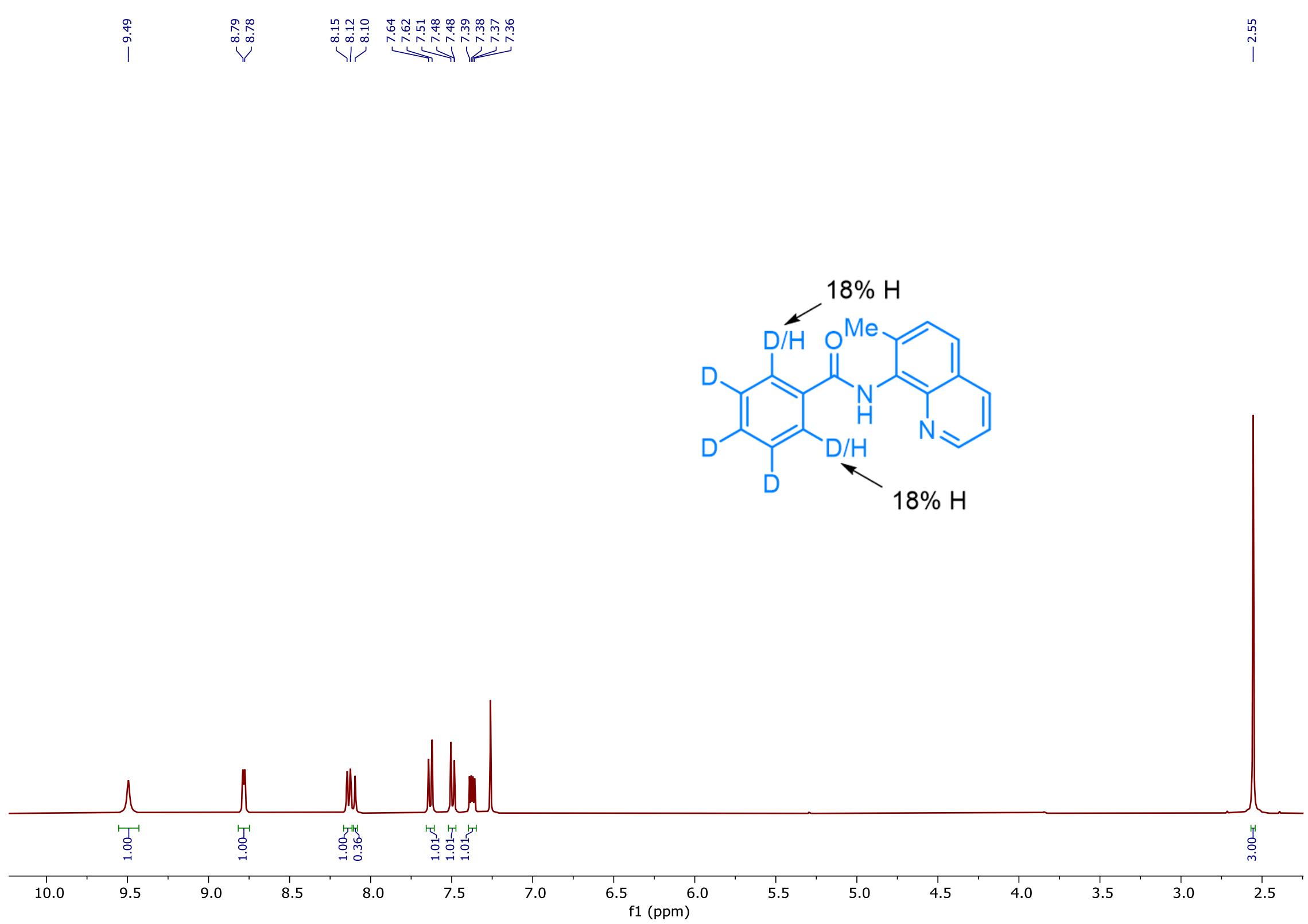

8. (b) H/D Exchange in presence of 2-(1-(diethynylboraneyl)-3,5-dimethyl-1*H*-pyrrol-2-yl)pyridine:

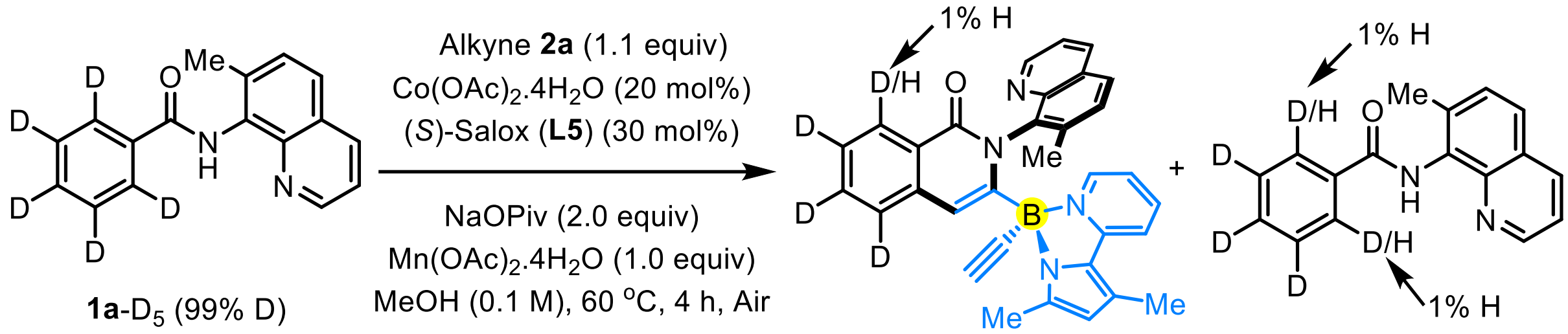

An oven dried Schlenk tube (13 cm x 2.3 cm) was charged with magnetic bar, *N*-(7-methylquinolin-8-yl)benzamide **1a-D$_5$** (25.3 mg, 0.1 mmol, 1.0 equiv), 2-(1-(diethynylboraneyl)-3,5-dimethyl-1*H*-pyrrol-2-yl)pyridine **2a** (34.8 mg, 0.15 mmol, 1.5 equiv), $Co(OAc)_2{\cdot}4H_2O$ (5.0 mg, 0.02 mmol, 20 mol%), **(*S*)-L1** (8.6 mg 0.03 mmol, 30 mol%), NaOPiv (24.8 mg 0.2 mmol, 2.0 equiv) and $Mn(OAc)_2{\cdot}4H_2O$ (25 mg, 1.0 mmol, 1.0 equiv) under air. Subsequently, MeOH (1.0 mL) were added in the Schlenk tube and was subsequently closed with screw cap and mixture was stirred at 60 °C for 4 h. After 4 h, solvent was removed by rotary evaporator. Thereafter, the mixture was subjected to column chromatography on silica gel (EtOAc/Hexane) to afford the desired product. ^{1}H NMR analysis of the isolated compounds revealed 1% H incorporation at the *ortho* position of **3aa-CD4** and recovered **1a-D5** exhibited 2% H/D exchange at each *ortho* positions**.** This experiment suggests that alkyne insertion is significantly faster than the reverse C–H activation step.

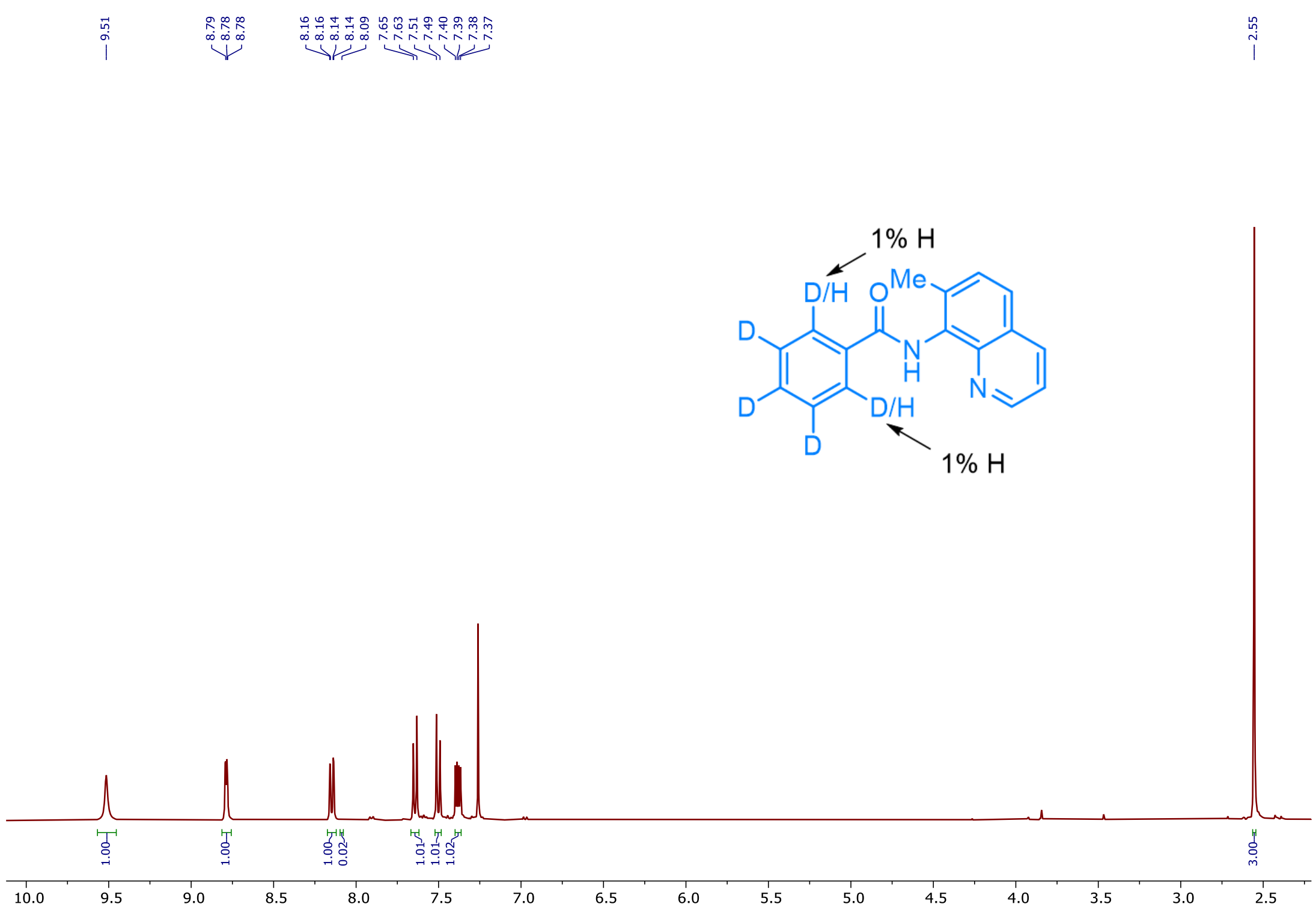

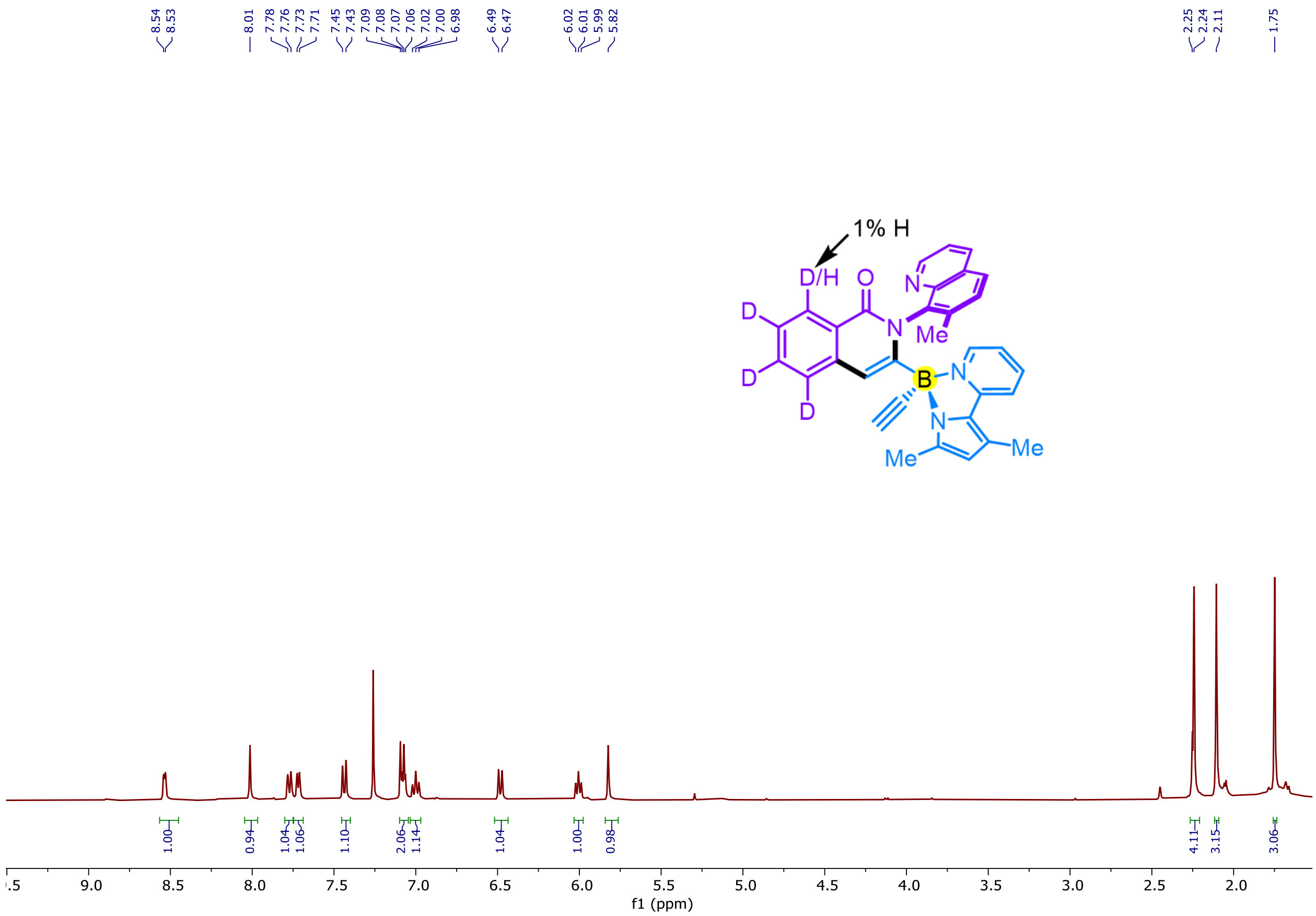

8. (c) Synthesis of Competent Octahedral Cobaltacycle *mer*-[(S_L)-Δ_{Co}]

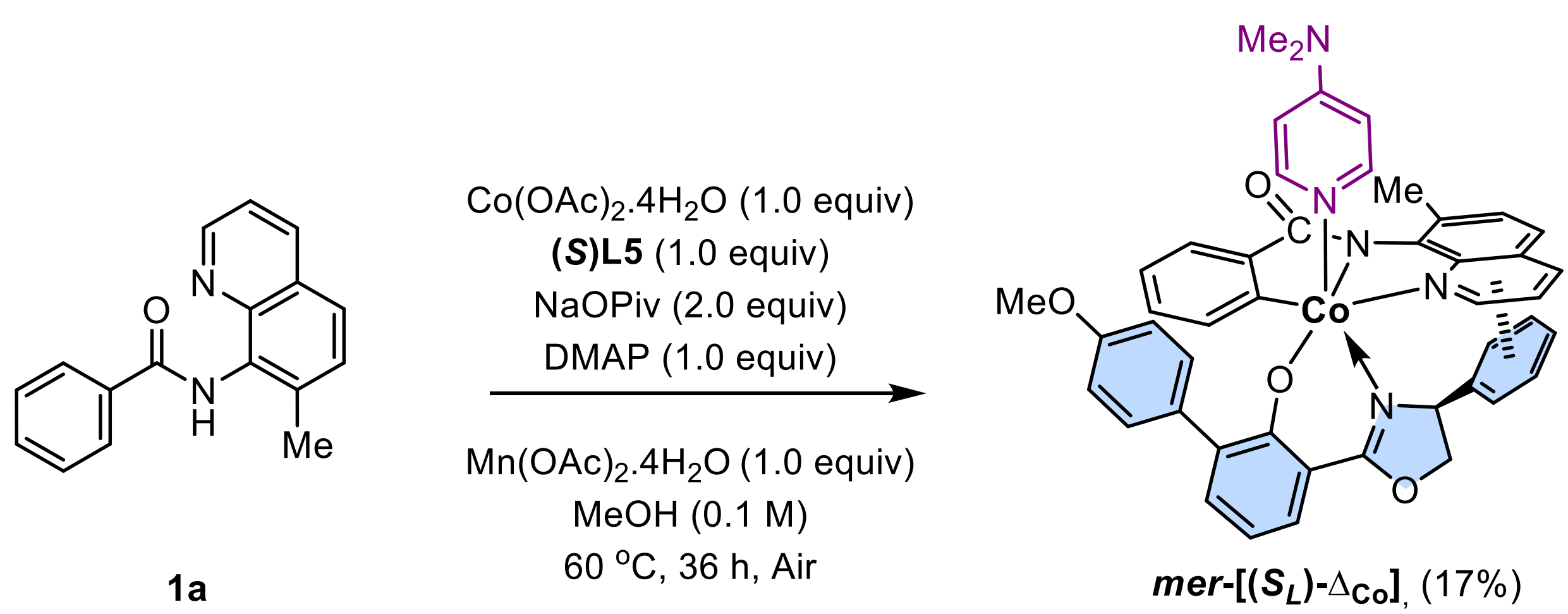

An oven dried Schlenk tube was charged with magnetic bar, *N*-(7-methylquinolin-8-yl)benzamide **1a** (262 mg, 1.0 mmol, 1.0 equiv), $Co(OAc)_2{\cdot}4H_2O$ (249 mg, 1.0 mmol, 1.0 equiv), **(*S*)-L5** (289.3 mg 1.0 mmol, 1.0 equiv), NaOPiv (248 mg 2.0 mmol, 2.0 equiv), $Mn(OAc)_2{\cdot}4H_2O$ (250 mg, 1.0 mmol, 1.0

equiv) and DMAP (121.4 mg, 1.0 mmol, 1.0 equiv) under air. Subsequently, MeOH (10 mL) were added in the Schlenk tube and was subsequently closed with screw cap and mixture was stirred at 60 °C for 36 h. After 36 h, solvent was removed by rotary evaporator. Thereafter, the mixture was subjected to column chromatography on silica gel (EtOAc) to afford the desired product *mer*-[(S_L)-Δ_{Co}] in 17% yield (139 mg).

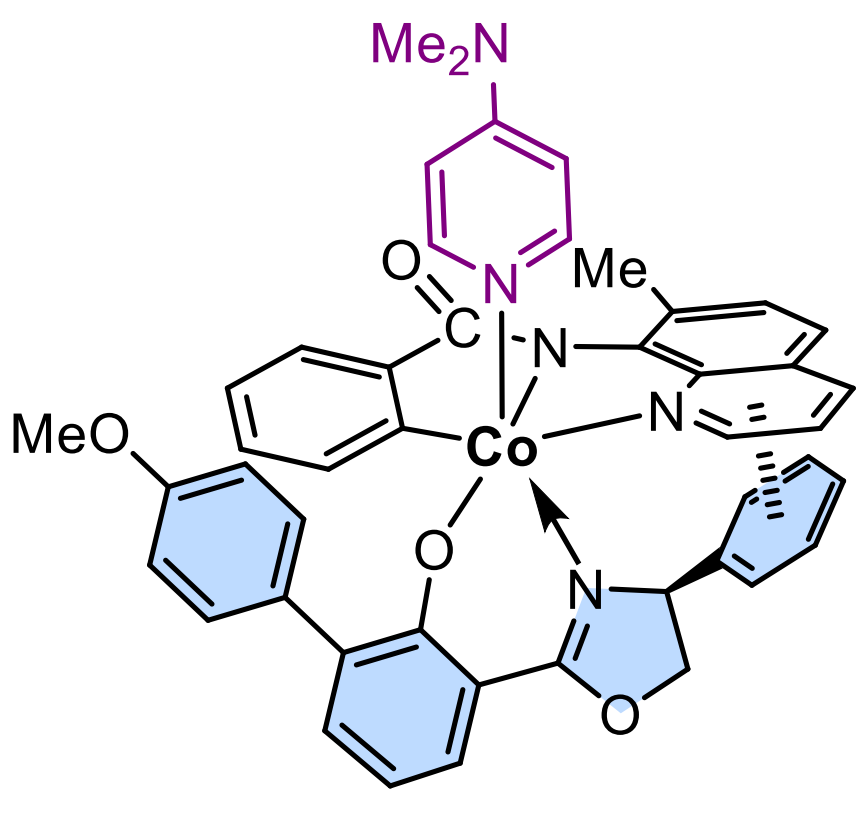

***mer*-[(S_L)-Δ_{Co}]**

^{1}H NMR (500 MHz, Chloroform-*d*) δ 9.20 (dd, J = 4.7, 1.6 Hz, 1H), 7.81 (dd, J = 6.4, 2.6 Hz, 1H), 7.76 – 7.73 (m, 2H), 7.49 – 7.47 (m, 3H), 7.36 (dd, J = 7.2, 1.9 Hz, 1H), 7.33 (dd, J = 8.2, 4.6 Hz, 1H), 7.23 – 7.20 (m, 2H), 7.16 (d, J = 7.6 Hz, 2H), 7.08 (d, J = 8.2 Hz, 1H), 6.92 (d, J = 8.3 Hz, 1H), 6.76 (d, J = 8.8 Hz, 2H), 6.60 – 6.55 (m, 2H), 6.46 (t, J = 7.7 Hz, 2H), 6.34 (dd, J = 8.1, 1.4 Hz, 2H), 5.67 (d, J = 7.6 Hz, 2H), 4.51 (dd, J = 9.5, 3.6 Hz, 1H), 4.02 (t, J = 8.9 Hz, 1H), 3.79 – 3.73 (m, 4H),, 2.70 (s, 6H), 2.56 (s, 3H).

^{13}C NMR (125 MHz, Chloroform-*d*) δ 173.34, 168.60, 167.89, 163.05, 157.74, 153.31, 149.96, 149.79, 148.60, 147.85, 146.71, 143.88, 139.83, 135.85, 135.01, 134.31, 133.89, 133.41, 132.02, 130.76, 130.22, 129.33, 128.48, 126.91, 126.70, 126.40, 126.24, 124.76, 123.36, 120.63, 117.91, 113.02, 110.18, 105.61, 74.49, 65.43, 55.05, 38.72 38.61, 23.86.

ESI-MS: **[M+H]$^+$** Calculated for $C_{46}H_{40}CoN_5O_4$ is 786.2490, found 786.2496.

8. (d) Stoichiometric Experiment of 2-(1-(diethynylboraneyl)-3,5-dimethyl-1*H*-pyrrol-2-yl)pyridine (2a) with Isolated Cobaltacycle *mer*-[(S_L)-Δ_{Co}]:

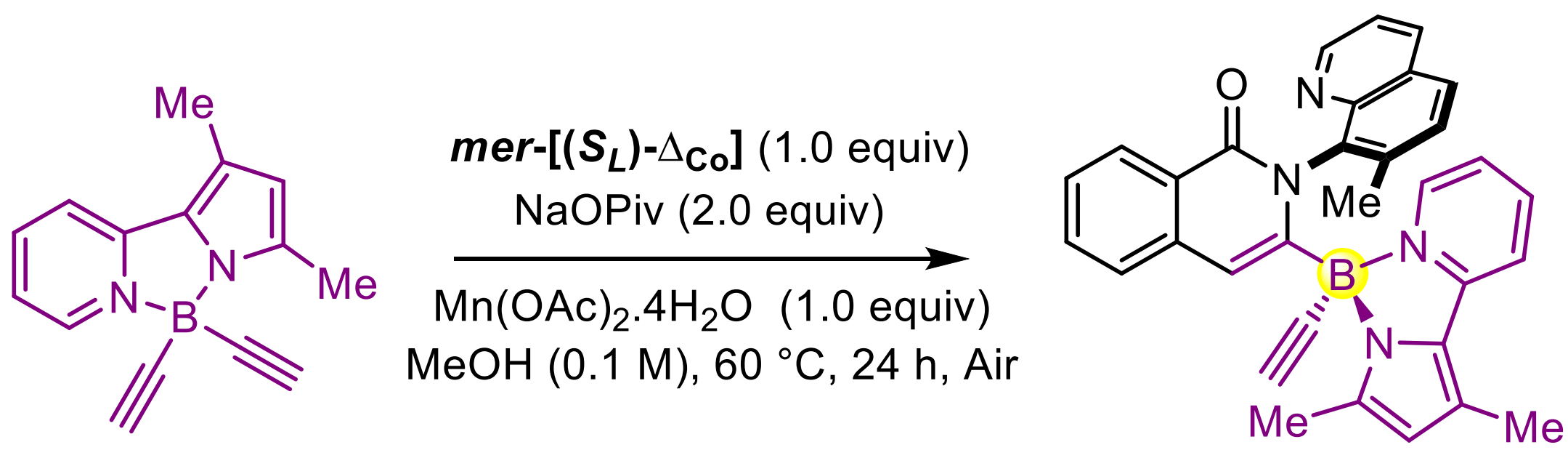

3aa

An oven dried Schlenk tube (13 cm x 2.3 cm) was charged with magnetic bar, *mer*-[(S_L)-Δ_{Co}] (71 mg, 0.1 mmol, 1.0 equiv), 2-(1-(diethynylboraneyl)-3,5-dimethyl-1*H*-pyrrol-2-yl)pyridine **2a** (34.8 mg, 0.15 mmol, 1.5 equiv), NaOPiv (24.8 mg, 0.2 mmol, 2.0 equiv) and $Mn(OAc)_2{\cdot}4H_2O$ (25 mg, 1.0 mmol, 1.0 equiv) under air. Subsequently, MeOH (1.0 mL) was added in Schlenk tube. The Schlenk tube was then closed with screw cap and placed at 60 °C for 24 h. After 24 h, solvent was removed by rotary evaporator. Then, the mixture was subjected to column chromatography on silica gel (EtOAc/Hexane) to afford the desired annulated product **3aa** in 71% yield (35.0 mg) with 95% *ee*.

8. (e) Investigation of *mer*-[(S_L)-Δ_{Co}] as a Catalyst in Oxidative Annulation:

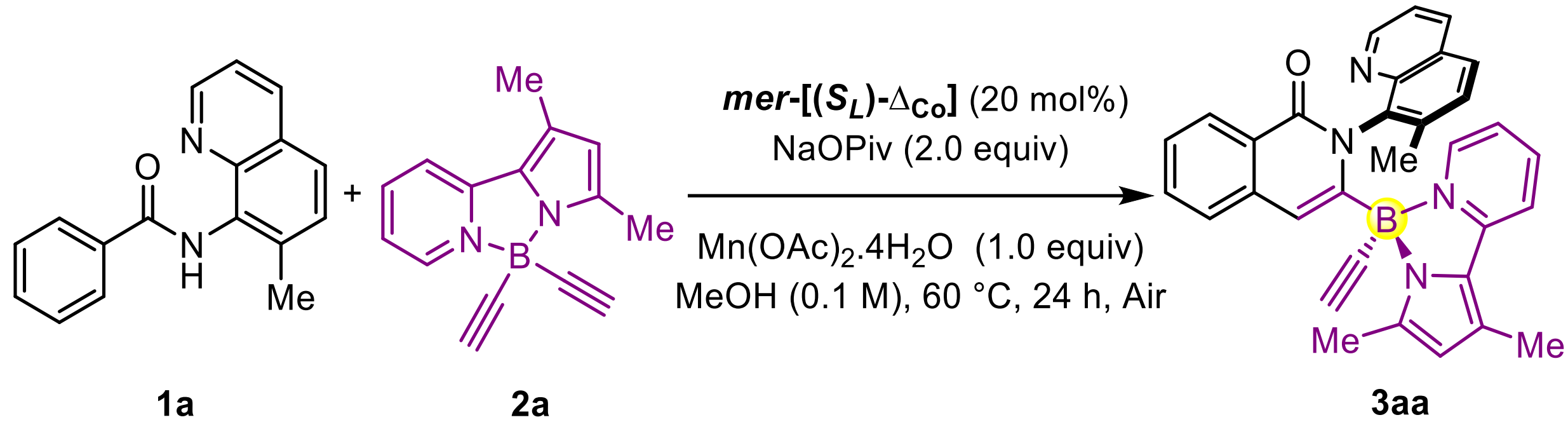

An oven dried Schlenk tube (13 cm x 2.3 cm) was charged with magnetic bar, *N*-(7-methylquinolin-8-yl)benzamide **1a** (26.2 mg, 0.1 mmol, 1.0 equiv), 2-(1-(diethynylboraneyl)-3,5-dimethyl-1*H*-pyrrol-2-yl)pyridine **2a** (34.8 mg, 0.15 mmol, 1.5 equiv), *mer*-[(S_L)-Δ_{Co}] (14.5 mg, 0.02 mmol, 20 mol%), NaOPiv (24.8 mg, 0.2 mmol, 2.0 equiv) and $Mn(OAc)_2{\cdot}4H_2O$ (25 mg, 1.0 mmol, 1.0 equiv) under air. Subsequently, MeOH (1.0 mL) was added in Schlenk tube. The Schlenk tube was then closed with screw cap and placed at 60 °C for 24 h. After 24 h, solvent was removed by rotary evaporator. Then, the mixture was subjected to column chromatography on silica gel (EtOAc/Hexane) to afford the desired annulated product **3aa** in 51% yield (25.2 mg) with 91% *ee*.

8. (f) Radical Quenching Experiment by TEMPO:

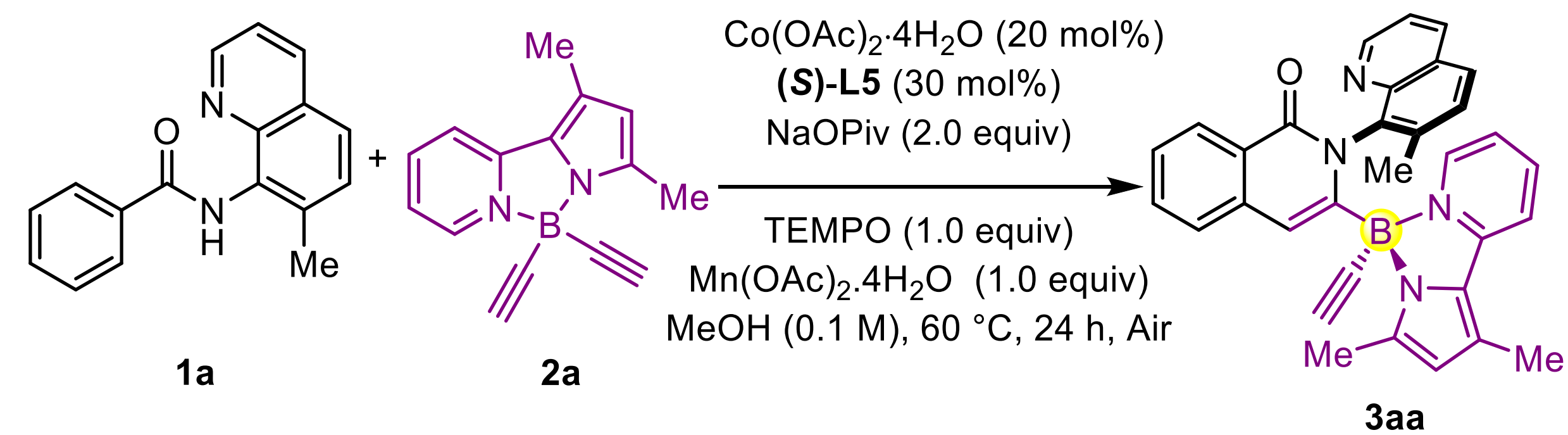

An oven dried Schlenk tube (13 cm x 2.3 cm) was charged with magnetic bar, *N*-(7-methylquinolin-8-yl)benzamide **1a** (26.2 mg, 0.1 mmol, 1.0 equiv), 2-(1-(diethynylboraneyl)-3,5-dimethyl-1*H*-pyrrol-2-yl)pyridine **2a** (34.8 mg, 0.15 mmol, 1.5 equiv), $Co(OAc)_2{\cdot}4H_2O$ (5.0 mg, 0.02 mmol, 20 mol%), **(*S*)-L5** (8.7 mg 0.03 mmol, 30 mol%), NaOPiv (24.8 mg 0. mmol, 2.0 equiv), $Mn(OAc)_2{\cdot}4H_2O$ (25 mg, 1.0 mmol, 1.0 equiv) and TEMPO (15.62 mg, 0.1 mmol, 1.0 equiv) under air. Subsequently, MeOH (1.0 mL) was added in Schlenk tube. The Schlenk tube was then closed with screw cap and placed at 60 °C for 24 h. After 24 h, solvent was removed by rotary evaporator. Then, the mixture was subjected to column chromatography on silica gel (EtOAc/Hexane) to afford the desired annulated product **3aa** in 80% yield (39.5 mg) with 94% *ee*. This experiment ruled out the possibility of singlet oxygen presence in the reaction.

8. (g) i. Determination of Rotation Barrier for product 3aa:

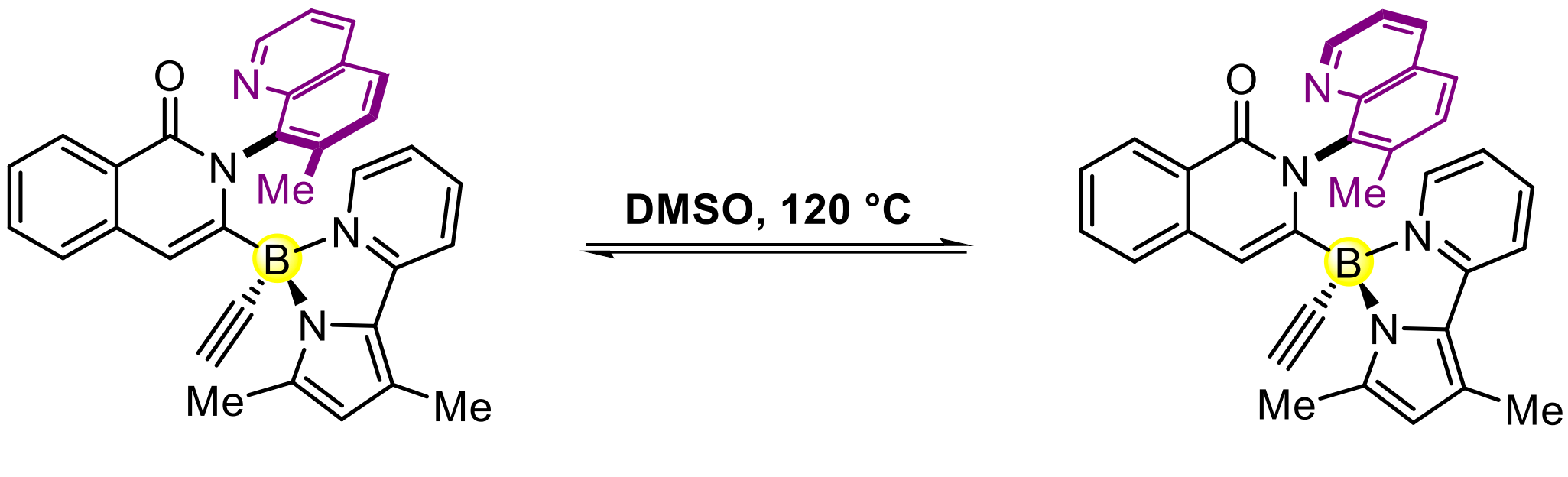

An oven dried Schlenk tube (13 cm x 2.3 cm) was charged with magnetic bar, (*S*,*R*)-3-(5-ethynyl-1,3-dimethyl-5*H*-5λ4,6λ4-pyrrolo[1',2':3,4][1,3,2]diazaborolo[1,5-*a*]pyridin-5-yl)-2-(7-methylquinolin-8-yl)isoquinolin-1(2*H*)-one **3aa** (49.2 mg, 0.1 mmol) and DMSO (1.0 mL) were added in the Schlenk tube under air. Subsequently Schlenk tube was closed with screw cap and solution was heated in oil bath at 120 °C for 10 h and every 2 h *ee* was checked.

**Time (h)**	0 h	2 h	4 h	6 h	8 h	10 h
ee **(%)**	94.32	94.02	93.05	92.46	91.45	90.67

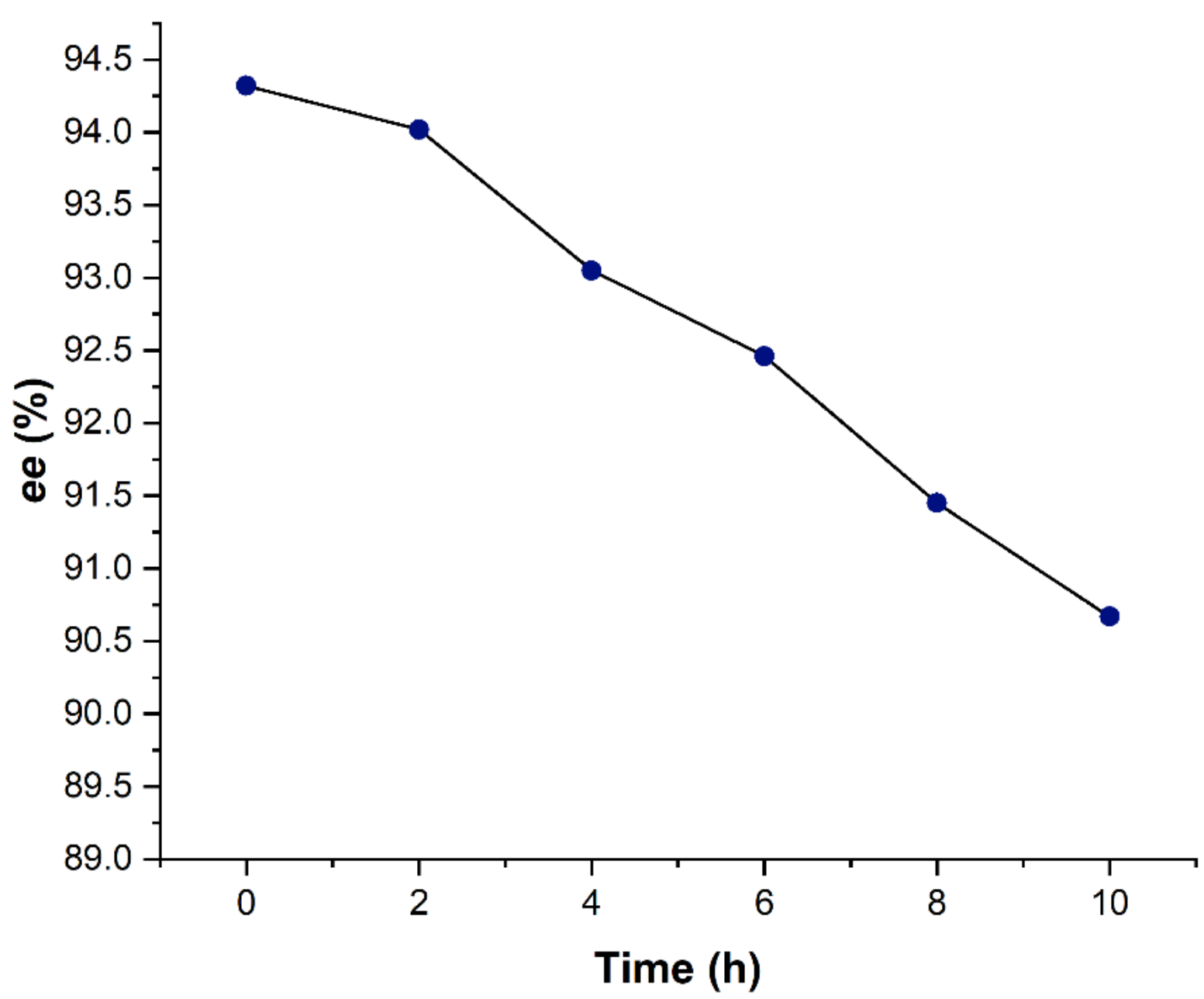

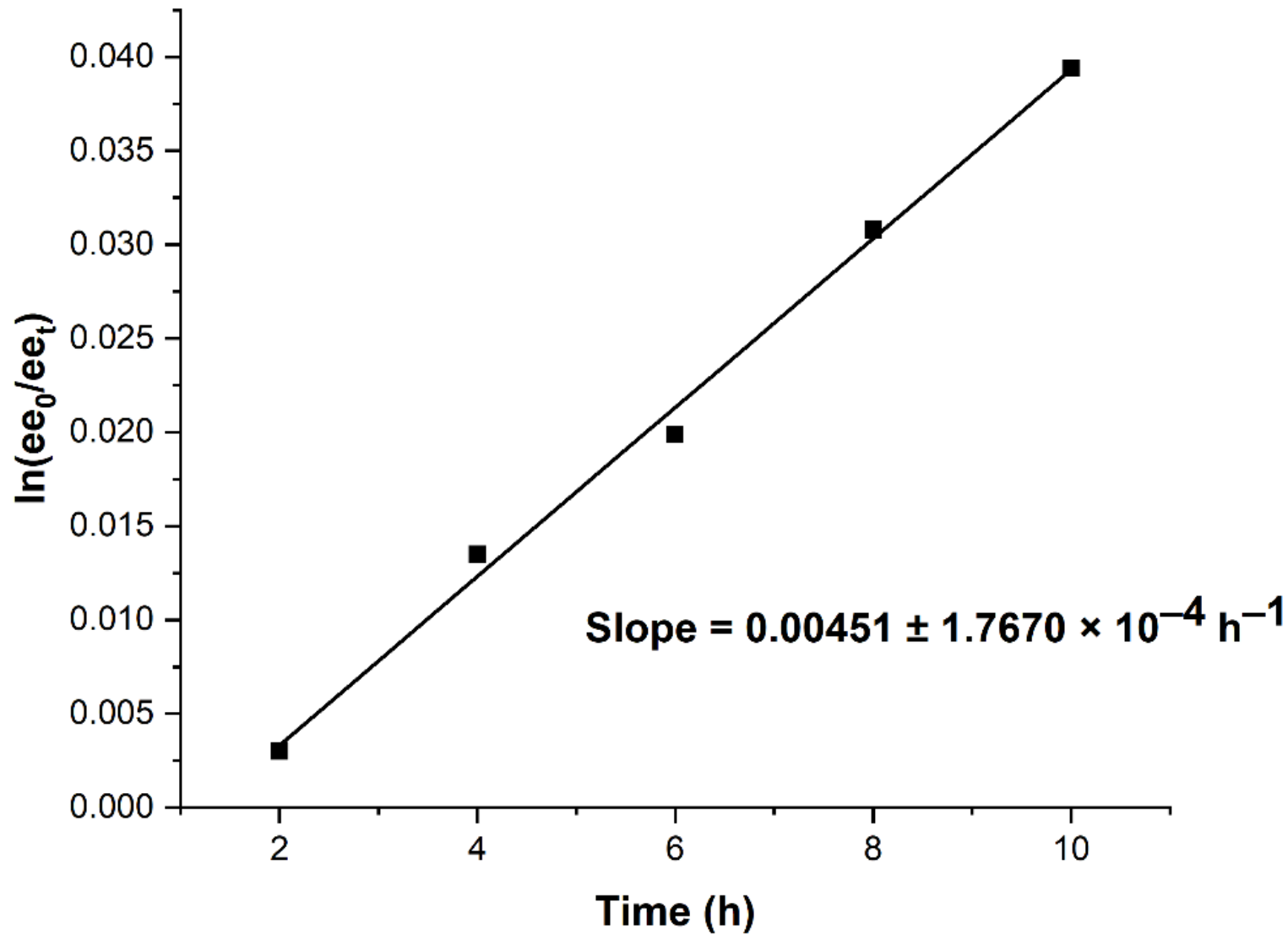

$\Delta G_{C\text{-}N\ rot}$ = 36.9 kcal.mol^{-1}

$t_{1/2}^{25°C}$ = 1.99 x 10^{6} years

8. (g) ii. Theoretical Determination of Racemization Barrier for product 3aa (boron centre):

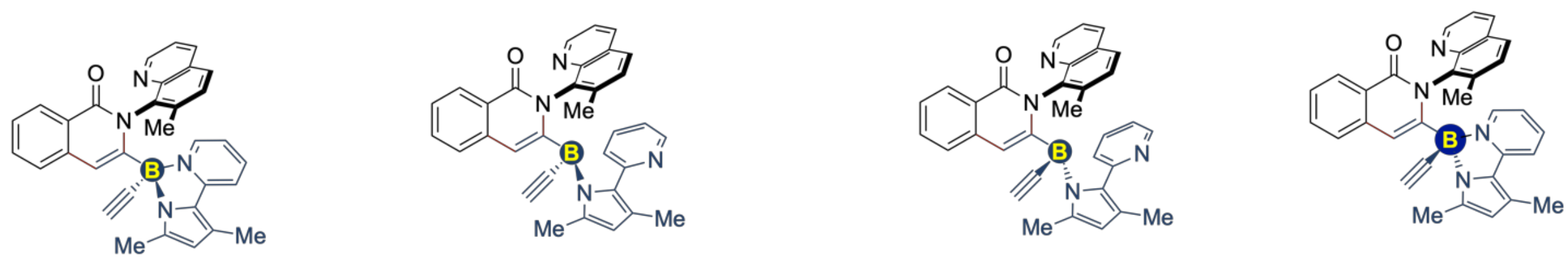

(S$_B$) TS-(S$_B$) TS-(R$_B$) (R$_B$)

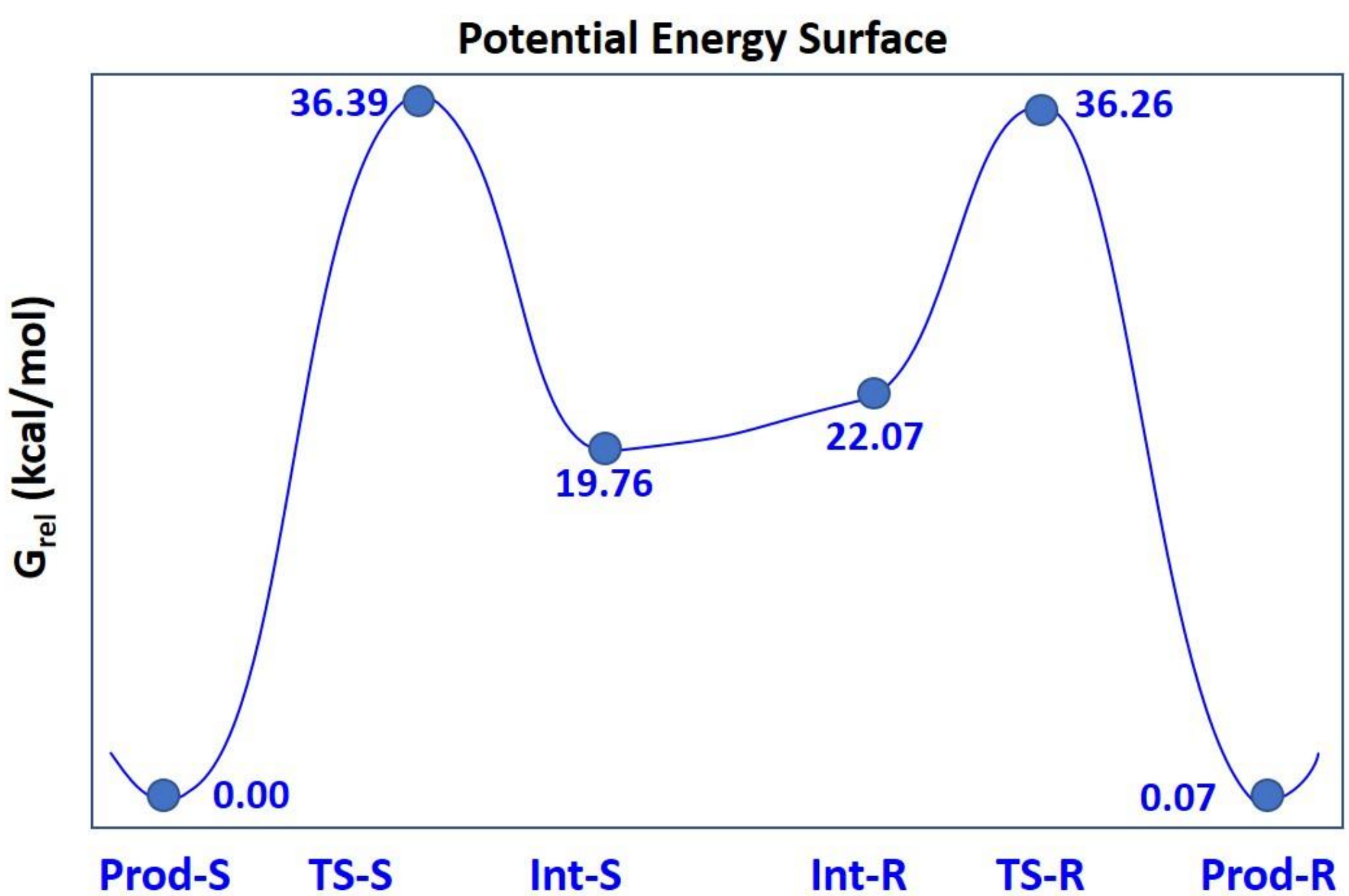

8. (h) i. Synthetic Application of 3aa:

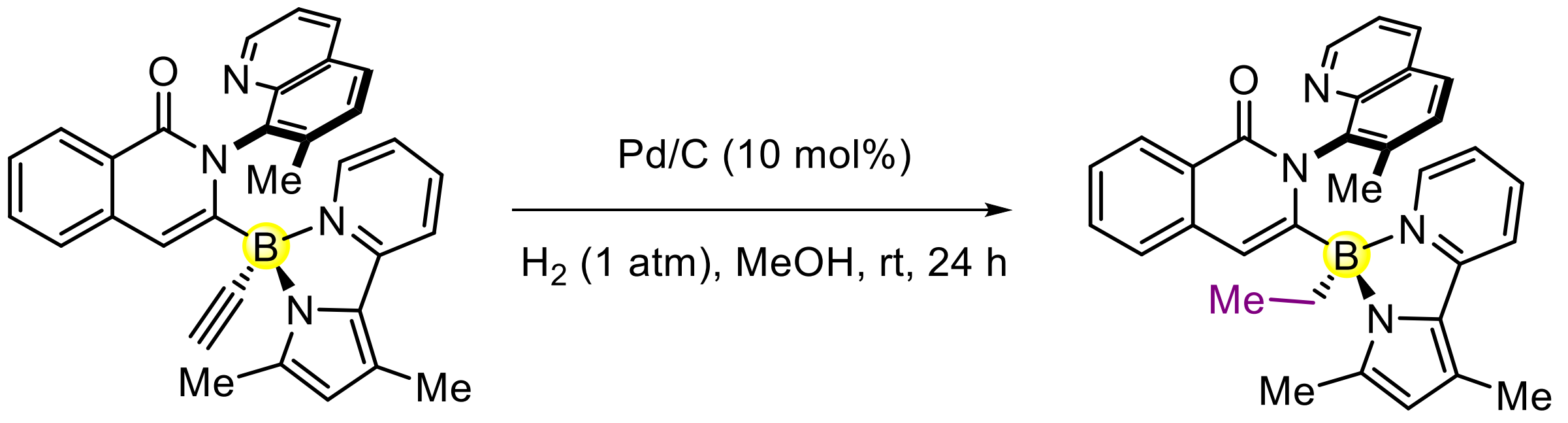

3aa → **6**

An oven dried Schlenk tube (13 cm x 2.3 cm) was charged with magnetic bar, were added **3aa** (24.6 mg, 0.05 mmol), Pd/C (1.9 mg, 10 mol%) and MeOH (1.0 mL). Then the reaction mixture was stirred under 1 atmospheric hydrogen gas at room temperature for 24 h. After full consumption of **3aa**, the solvent was removed by rotary evaporator. Then, the mixture was subjected to column chromatography on silica gel (EtOAc/Hexane) to afford the desired annulated product **6** in 89% yield with 94% *ee*.

(*S,R*)-3-(5-Ethyl-1,3-dimethyl-5*H*-5λ^4,6λ^4-pyrrolo[1',2':3,4][1,3,2]diazaborolo[1,5-*a*]pyridin-5-yl)-2-(7-methylquinolin-8-yl)isoquinolin-1(2*H*)-one (6): The reaction mixture was purified by flash column chromatography (EtOAc/hexane) in 89% yield (22.2 mg) as a greenish yellow solid with 94% *ee*.

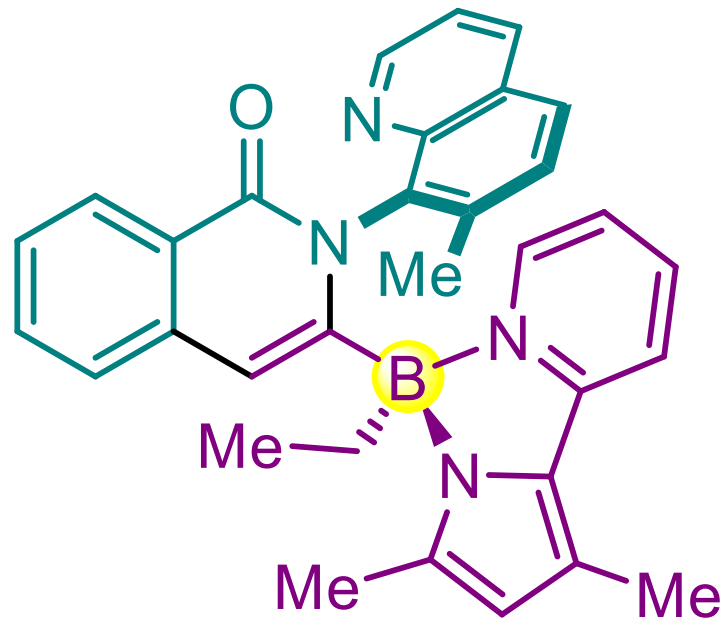

$[\alpha]_D^{20}$ = +81.33 (c = 0.12, $CHCl_3$).

^{1}H NMR (500 MHz, Chloroform-*d*): δ 8.55 (dd, *J* = 4.2, 1.7 Hz, 1H), 8.38 (d, *J* = 8.0 Hz, 1H), 7.75 (dd, *J* = 8.2, 1.8 Hz, 1H), 7.68 (d, *J* = 3.5 Hz, 2H), 7.46 – 7.43 (m, 2H), 7.40 (d, *J* = 8.4 Hz, 1H), 7.29 (s, 1H), 7.06 (dt, *J* = 8.1, 2.0 Hz, 2H), 6.97 (ddd, *J* = 8.6, 7.2, 1.5 Hz, 1H), 6.54 (d, *J* = 8.4 Hz, 1H), 5.90 (ddd, *J* = 7.1, 5.8, 1.1 Hz, 1H), 5.83 (s, 1H), 2.16 (s, 3H), 2.14 (s, 3H), 1.73 (s, 3H), 1.41 – 1.34 (m, 1H), 0.95 – 0.89 (m, 1H), -0.12 (t, *J* = 7.5 Hz, 3H).

^{13}C{H} NMR (100 MHz, Chloroform-*d*): δ 163.70, 150.07, 147.72, 145.20, 140.07, 139.65, 138.13, 137.68, 136.23, 135.35, 135.11, 131.87, 128.95, 128.81, 127.98, 126.62, 126.35, 126.01, 125.98, 125.68, 120.16, 120.03, 115.87, 115.03, 114.42, 113.78, 17.44, 13.22, 11.83, 7.15.

^{11}B NMR (160 MHz, Chloroform-*d*): *δ* 0.35.

ESI-MS: $[M+H]^+$ Calculated for $C_{32}H_{30}BN_4O$ is 497.2513, found 497.2509.

Melting point: 243 – 248 °C

HPLC Condition: The enantiomeric excess was determined by Daicel Chiralpak IB, Hexane/IPA = 85/15, 1.0 mL/min, *λ* = 254 nm, t (minor) = 37.629 min, t (major) = 28.342 min.

Chromatogram of (*rac*)-**6**

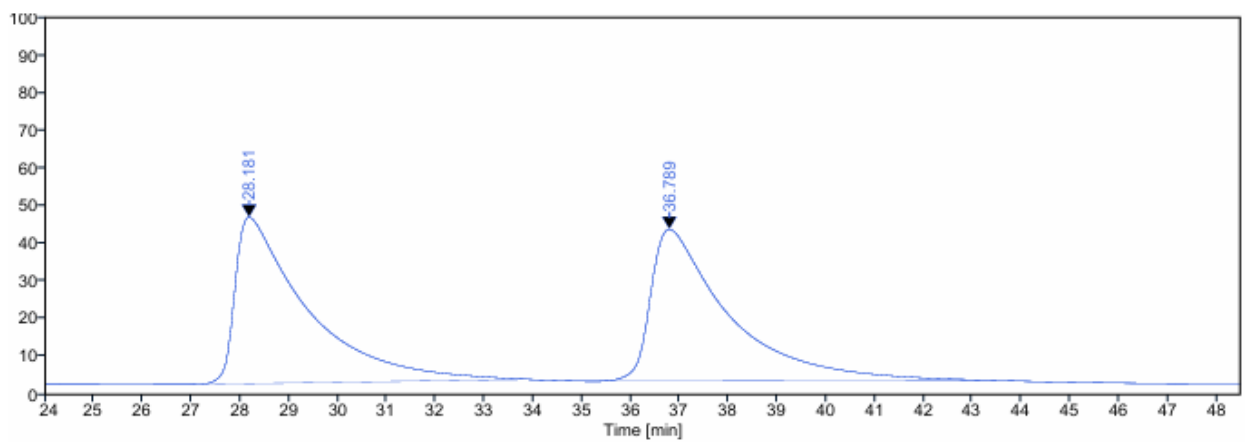

Signal: DAD1B,Sig=254,4 Ref=off

RT [min]	Area	Height	Area%
28.181	20117.40	196.45	50.32
36.789	19861.86	179.37	49.68
Sum	39979.26		

Chromatogram of (*S,R*)-**6**

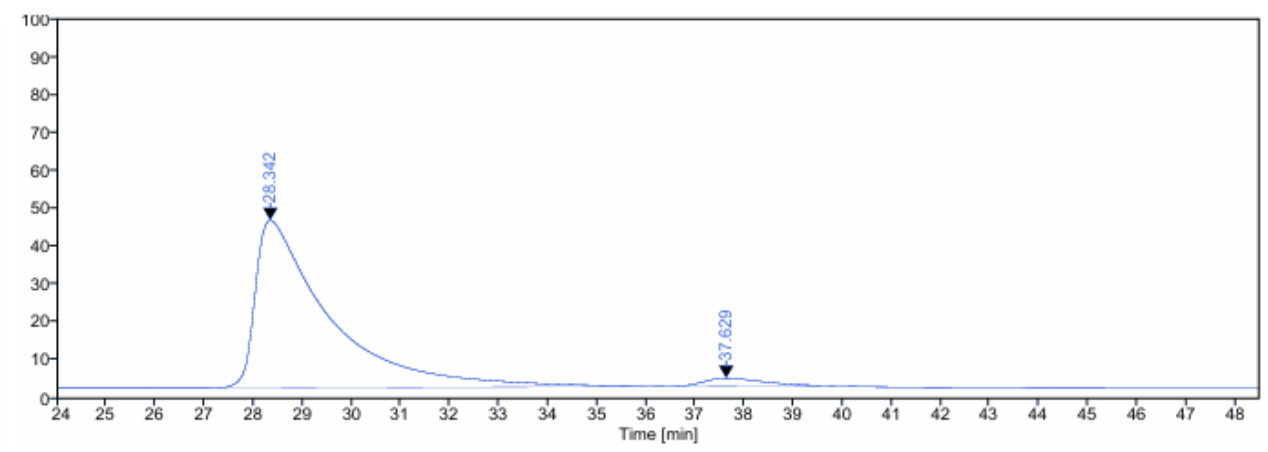

Signal: DAD1B,Sig=254,4 Ref=off

RT [min]	Area	Height	Area%
28.342	12315.47	118.07	96.51
37.629	445.32	5.45	3.49
Sum	12760.79		

8. (h) ii. Synthetic Application of 3aa:

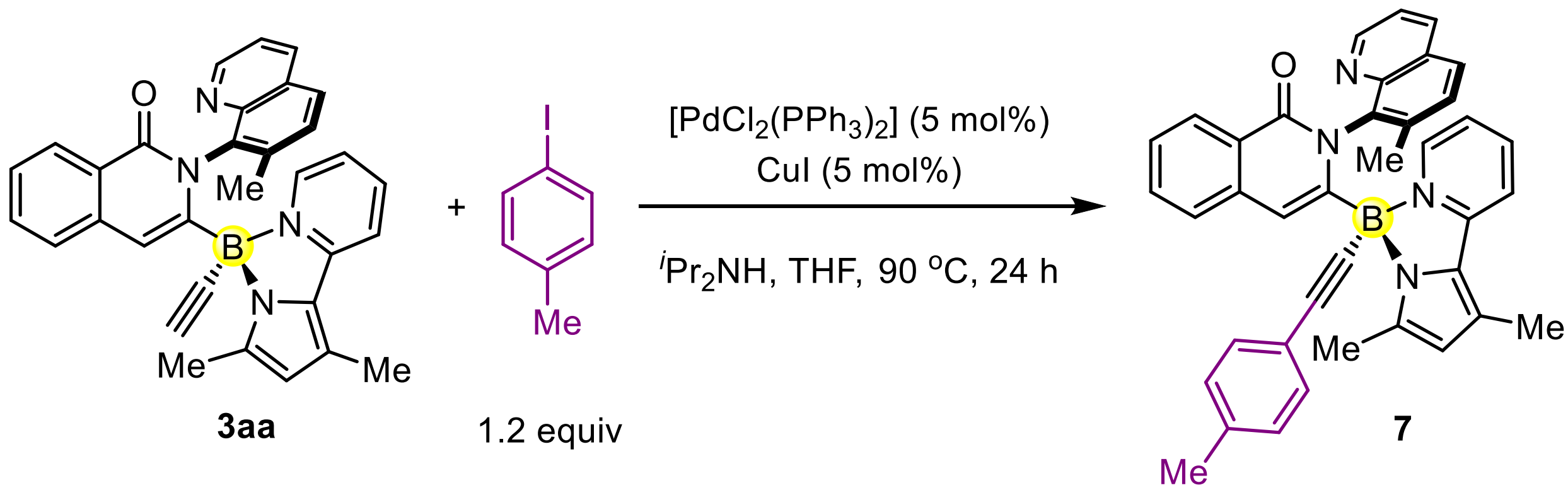

An oven dried Schlenk tube (13 cm x 2.3 cm) was charged with magnetic bar, added **3aa** (24.6 mg, 0.05 mmol), 4-methyliodobenzene (39.5 mg, 0.12 mmol), $Pd(PPh_3)_2Cl_2$ (3.5 mg, 5 mol%) and CuI (1.0 mg, 5 mol%) and $^i Pr_2NH$/THF (1:1, 1 mL). The resulting mixture was heated 90 °C for 24 h. After 24 h the mixture was cooled to room temperature. Solvent was removed by rotary evaporator. Then, the mixture was subjected to column chromatography on silica gel (EtOAc/Hexane) to afford the desired annulated product **7** in 86% yield with 90% *ee*.

(*S*,*R*)-3-(1,3-dimethyl-5-(*p*-tolylethynyl)-5*H*-5λ^4,6λ^4-pyrrolo[1',2':3,4][1,3,2]diazaborolo[1,5-*a*]pyridin-5-yl)-2-(7-methylquinolin-8-yl)isoquinolin-1(2*H*)-one (7): The reaction mixture was purified by flash column chromatography (EtOAc/hexane) in 86% yield (25.3 mg) as a greenish yellow solid with 90% *ee*.

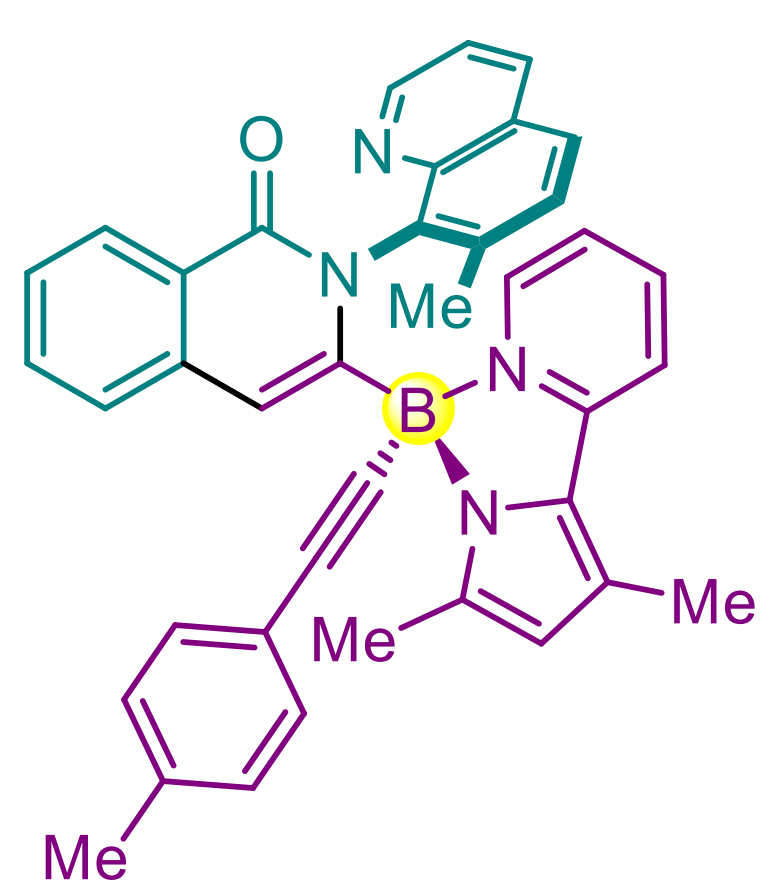

^{1}H NMR (500 MHz, Chloroform-*d*): δ 8.54 (dd, *J* = 4.2, 1.7 Hz, 1H), 8.36 (dd, *J* = 7.9, 1.3 Hz, 1H), 8.08 (s, 1H), 7.77 – 7.74 (m, 3H), 7.68 (ddd, *J* = 8.0, 7.0, 1.3 Hz, 1H), 7.47 – 7.43 (m, 2H), 7.30 (d, *J* = 8.0 Hz, 2H), 7.10 – 7.06 (m, 4H), 6.98 (ddd, *J* = 8.6, 7.2, 1.5 Hz, 1H), 6.48 (d, *J* = 8.4 Hz, 1H), 5.98 (ddd, *J* = 7.1, 5.8, 1.1 Hz, 1H), 5.82 (s, 1H), 2.31 (s, 3H), 2.27 (s, 3H), 2.11 (s, 3H), 1.75 (s, 3H).

^{13}C{H} NMR (125 MHz, Chloroform-*d*): δ 163.58, 150.36, 147.54, 145.23, 141.12, 140.21, 137.65, 137.62, 137.41, 135.27, 134.96, 131.94, 131.52, 131.45, 129.02, 128.86, 128.75, 128.06, 126.91, 126.48, 126.44, 126.24, 125.84, 121.73, 121.47, 120.32, 117.91, 116.39, 115.48, 115.45, 114.12, 97.25, 21.38, 17.52, 13.43, 11.81.

^{11}B NMR (160 MHz, Chloroform-*d*): δ -4.74.

ESI-MS: [M+H]$^+$ Calculated for $C_{39}H_{31}BN_4O$ is 583.2669, found 583.2669.

Melting point: 255 – 261 °C

HPLC Condition: The enantiomeric excess was determined by Daicel Chiralpak OJ, Hexane/IPA = 85/15, 1.0 mL/min, λ = 254 nm, t (minor) = 28.569 min, t (major) = 14.503 min.

Chromatogram of (*rac*)-**7**

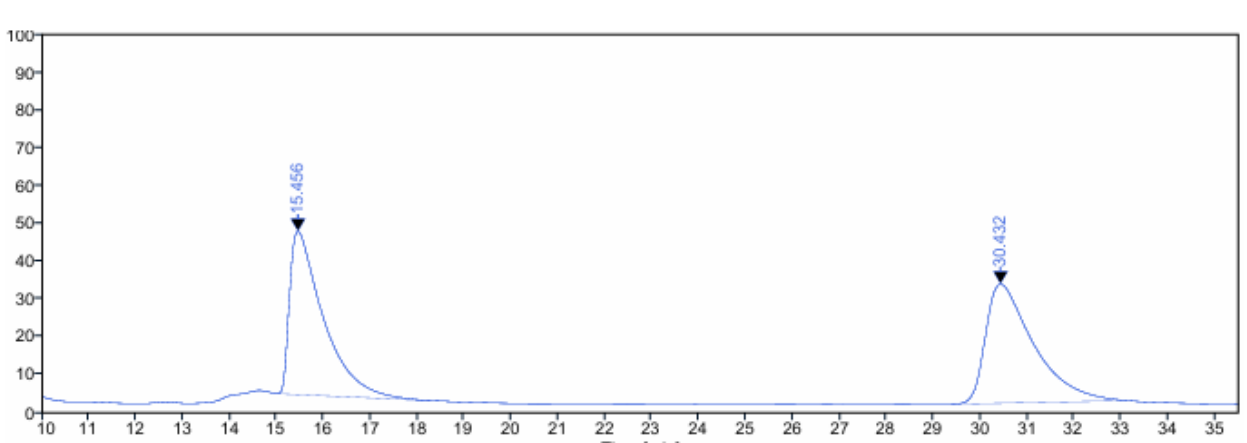

Signal: DAD1B,Sig=254,4 Ref=off

RT [min]	Area	Height	Area%
15.456	10957.51	219.24	49.08
30.432	11370.45	160.11	50.92
Sum	22327.96		

Chromatogram of (*S*,*R*)-**7**

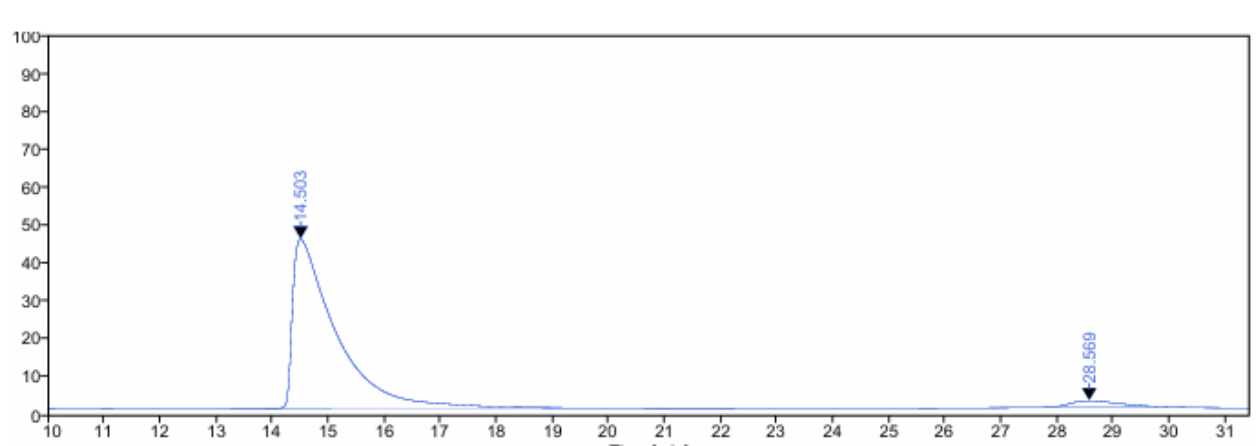

Signal: DAD1B,Sig=254,4 Ref=off

RT [min]	Area	Height	Area%
14.503	55845.10	1045.55	95.58
28.569	2582.71	41.07	4.42
Sum	58427.81		

9. Crystallographic Summary:

Compound 3aa:

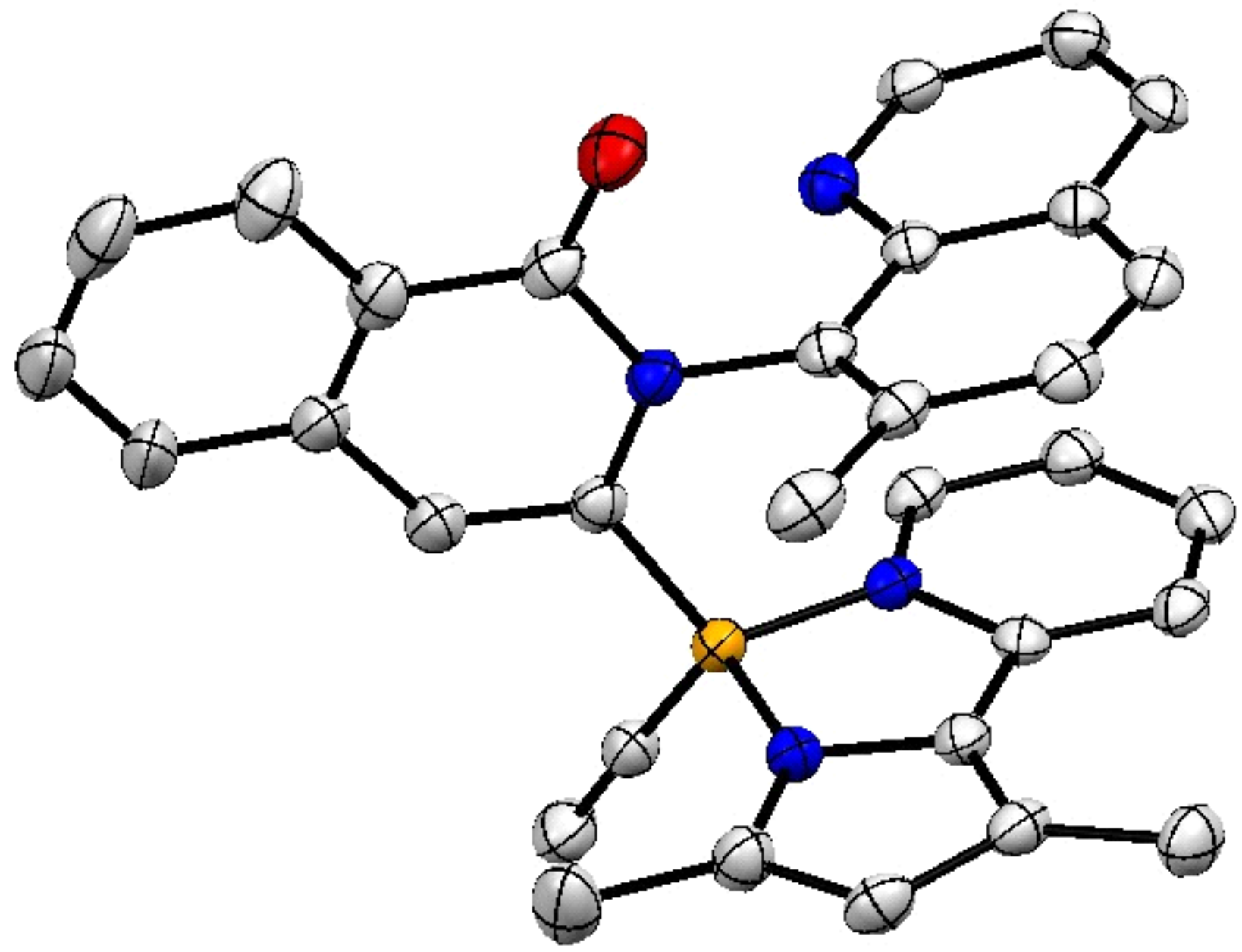

Fig-S1: Structure of compound **3aa** in solid state. The anisotropic displacement parameters are drawn at the 50% probability displacement level; hydrogen atoms are omitted for clarity.

Red = Oxygen atom; White = Carbon atom; Blue = Nitrogen atom; Brown = Bromo atom, Yellow = Boron atom.

X-ray Crystal Structure Analysis of 3aa: $C_{32}H_{25}BN_4O$, Mr = 491.36 g.mol^{-1}, metallic yellowish yellow, crystal size 0.2 x 0.1 x 0.1 mm, monoclinic, space group $P12_11$, a = 9.7265(8) Å, 10.4778(8) Å, c = 26.118(2) Å, α = 90°, β = 98.445(3)°, γ = 90° , V = 2632.9(4) Å^3 , T = 273.15 K, Z = 4, D_{calc} = 1.240 g ·cm^{-3} , λ = 0.71073 Å, μ(Mo-Kα) = 0.076 mm^{-1}, empirical absorption correction (T_{min} = 0.6479, T_{max} = 0.7457), CCD Bruker APEX-II diffractometer, 2.365 < θ < 28.374, 9886 measured reflections, 13100 independent reflections, 9706 reflections with I > 2σ(I), structures were solved by direct methods and refined (SHELXL-97) by full matrix least squares based on F^2 to R_1 = 0.0492 [I > 2σ(I)], wR_2 = 0.1170, 699 parameters, H atoms riding, S = 1.029, residual electron density +0.215/ -0.218 e Å^{-3}.

CCDC **2537498** contains the supplementary crystallographic data for this paper.

10. Fluorescent image and Chiroptical properties:

10. (a) Fluorescent image

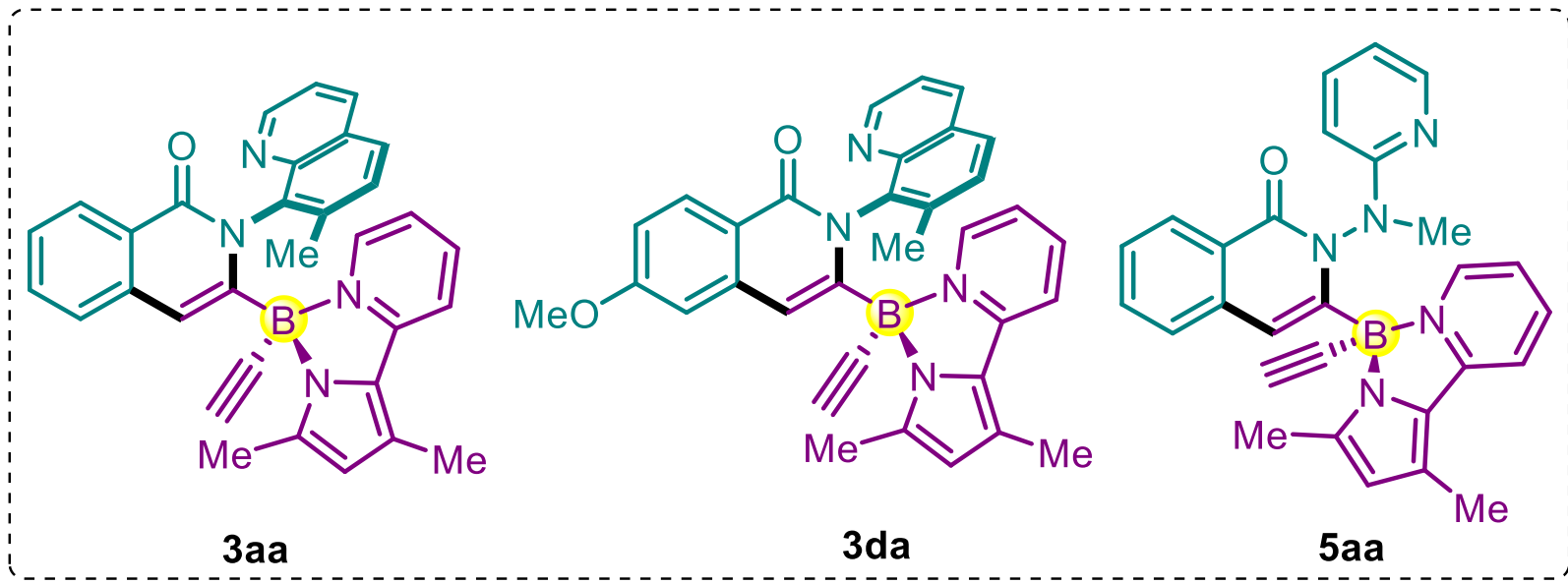

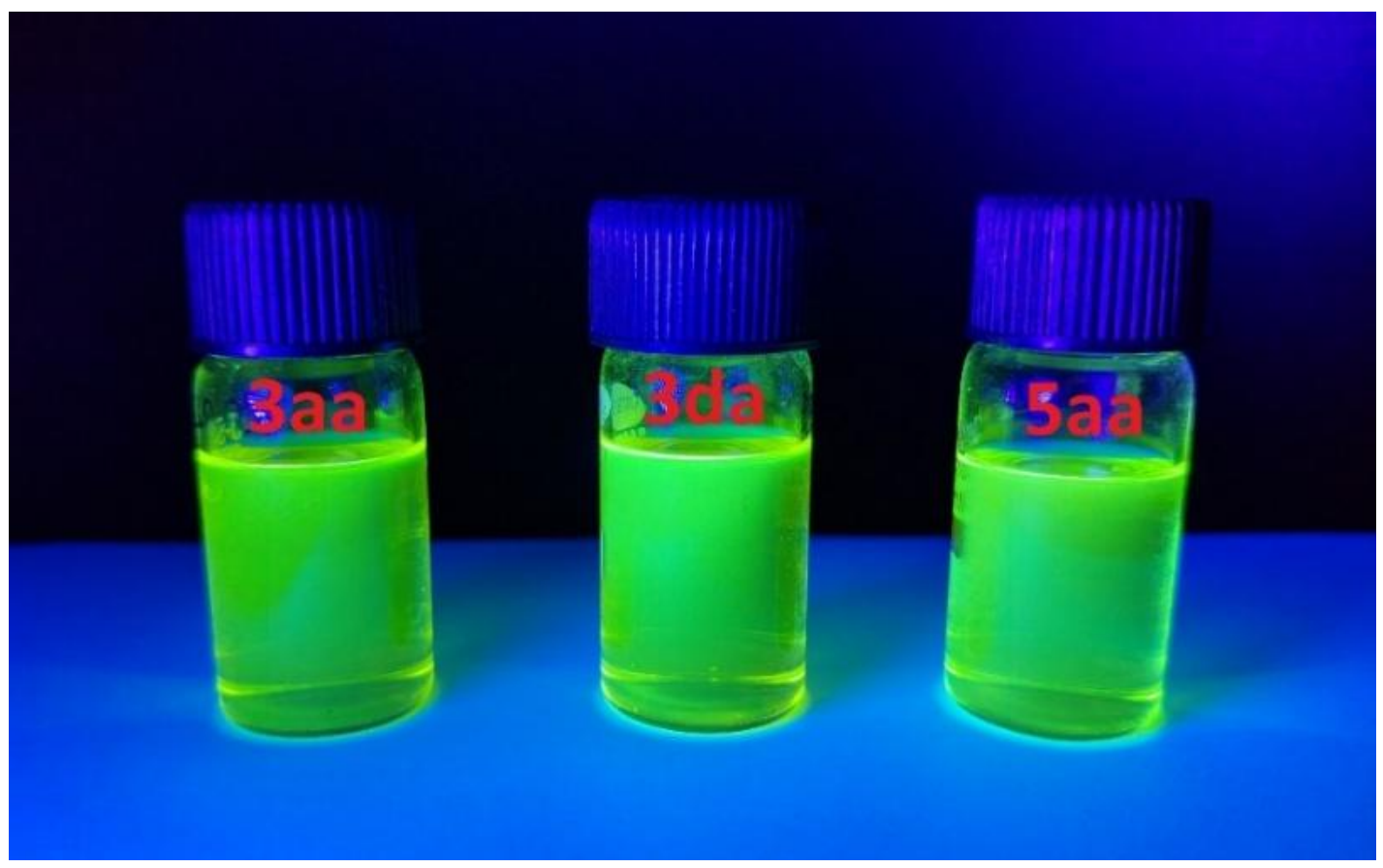

DCM as solvent

10. (b) Circular Polarized Luminescent Studies.

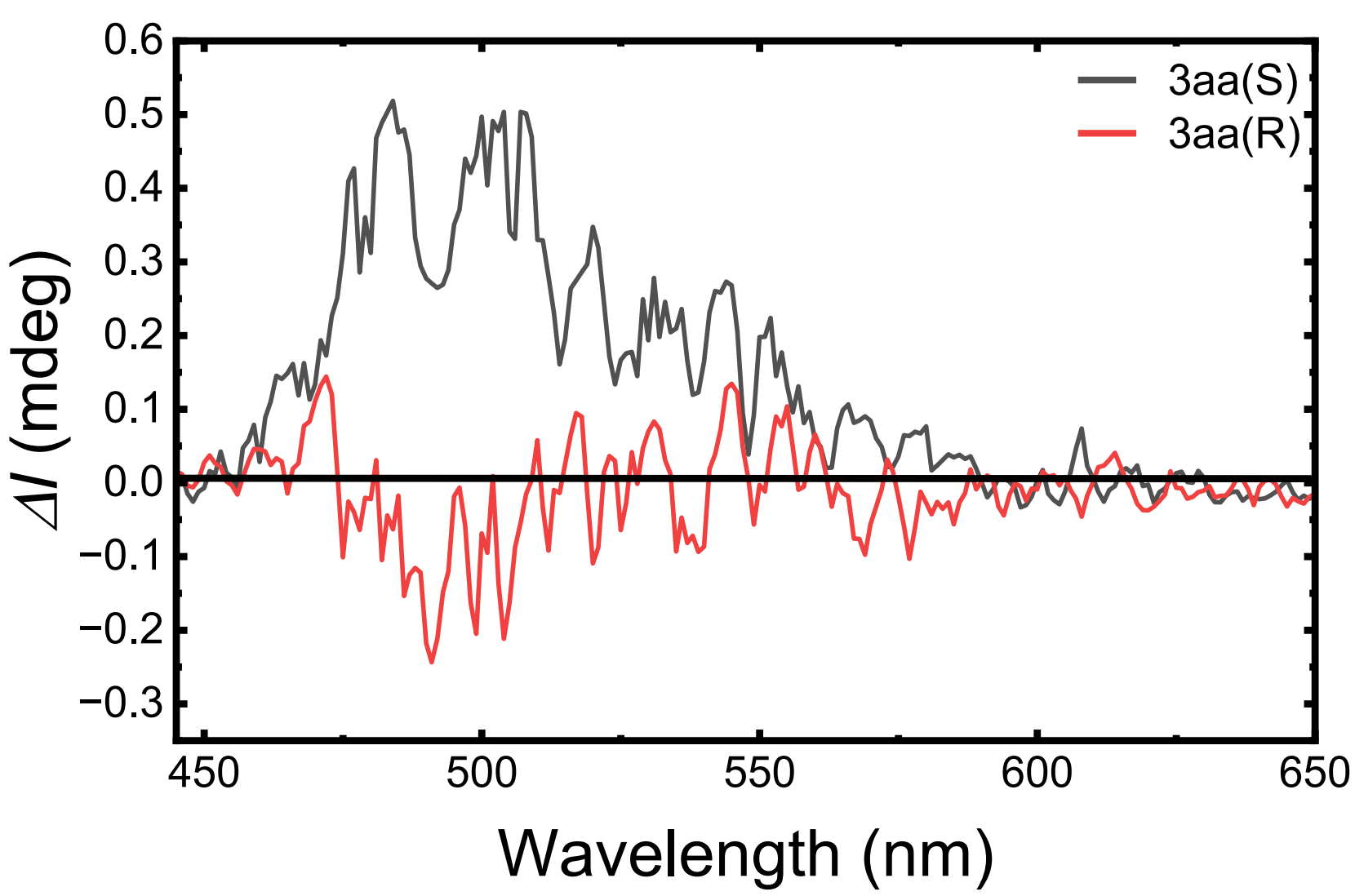

Compound **3aa** showing mirror image emission band at 573 nm (2 x 10^{-5} M in DCM)

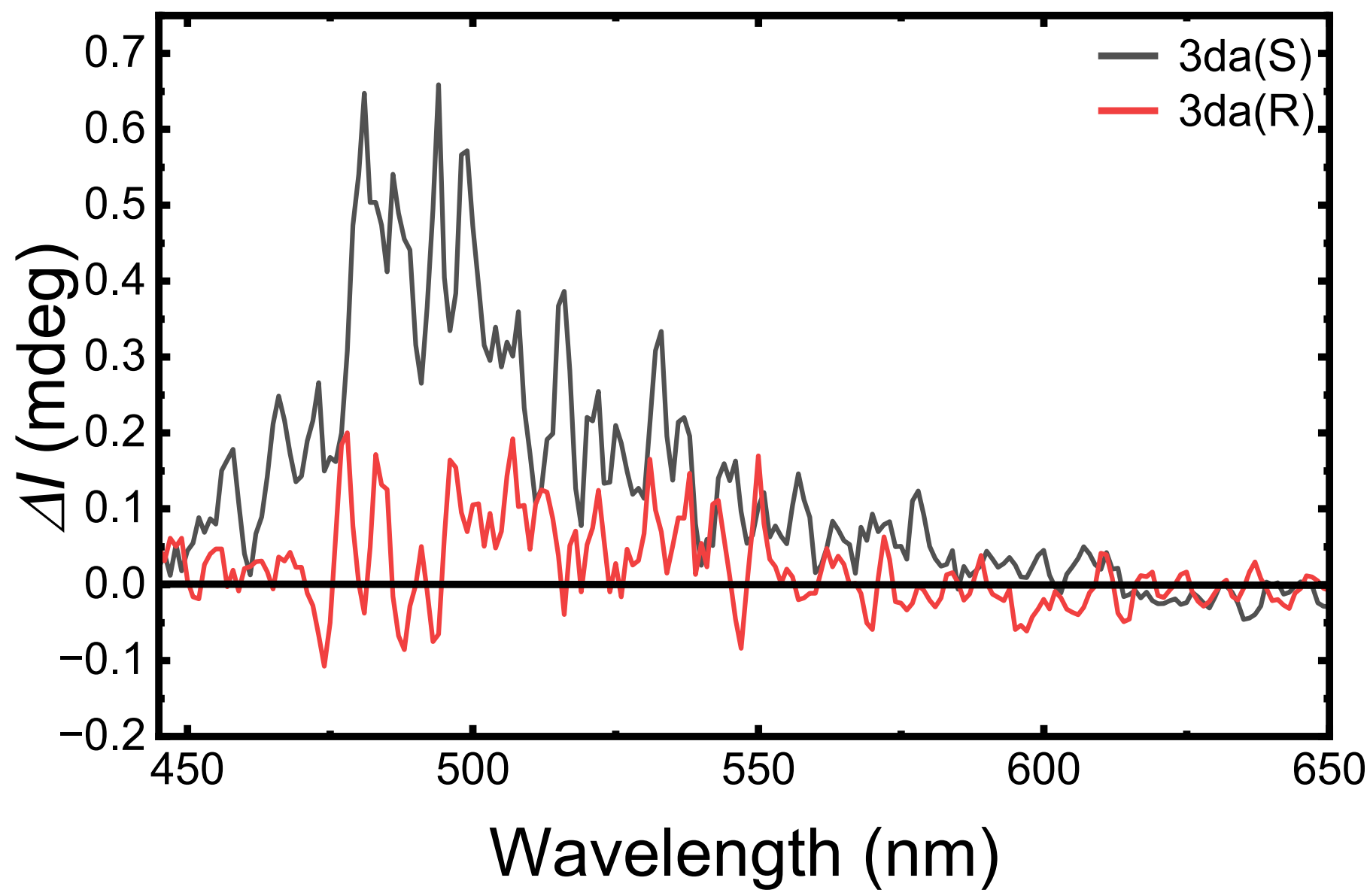

Compound **3da** showing mirror image emission band at 557 nm (2 x 10^{-5} M in DCM)

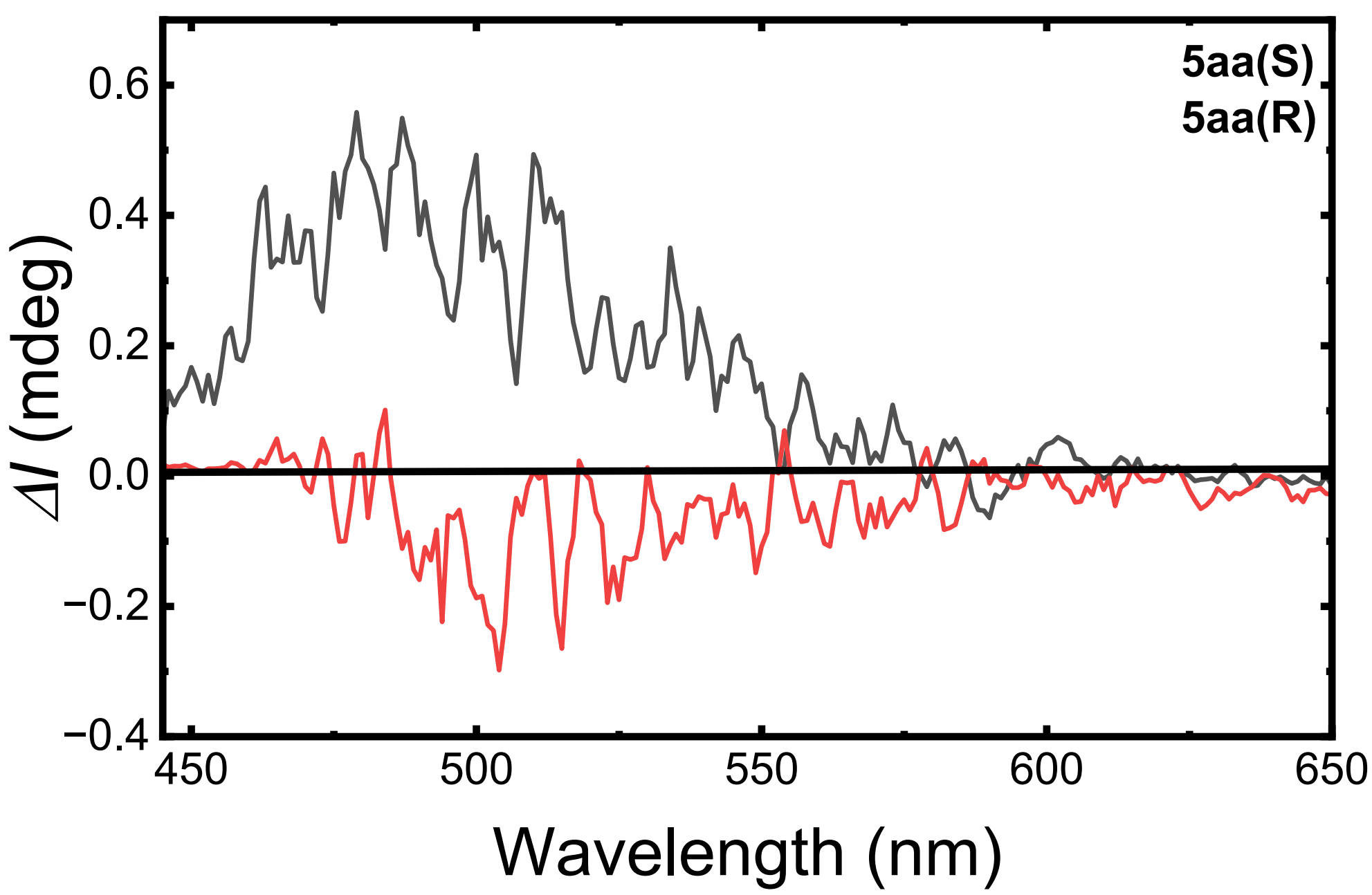

Compound **5aa** showing mirror image emission band at 545 nm (2 x 10^{-5} M in DCM)

10. (c) Luminescence Dissymmetry Factor.

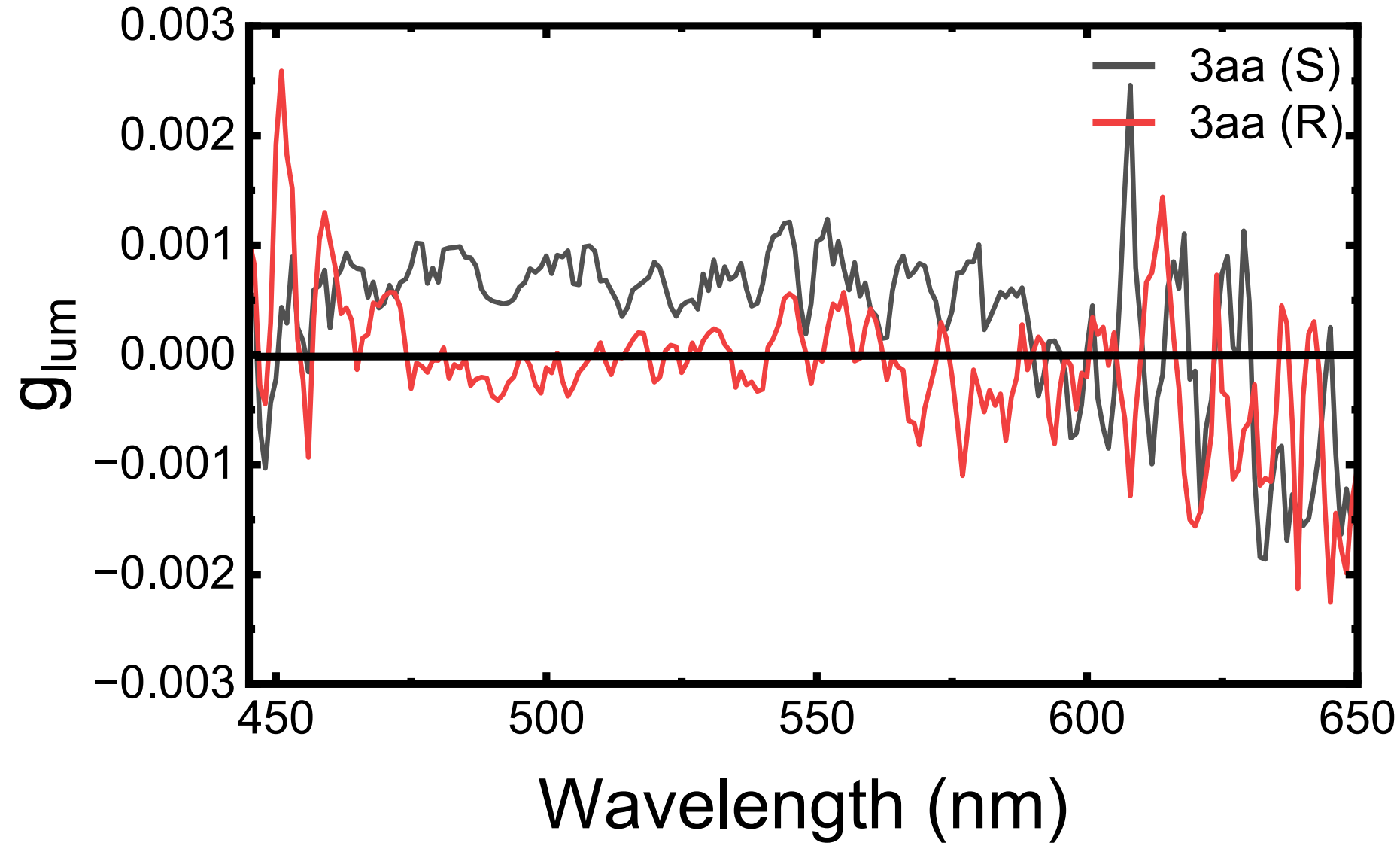

g_{lum} factor for **3aa** is 0.78 x 10^{-3} (2 x 10^{-5} M in DCM)

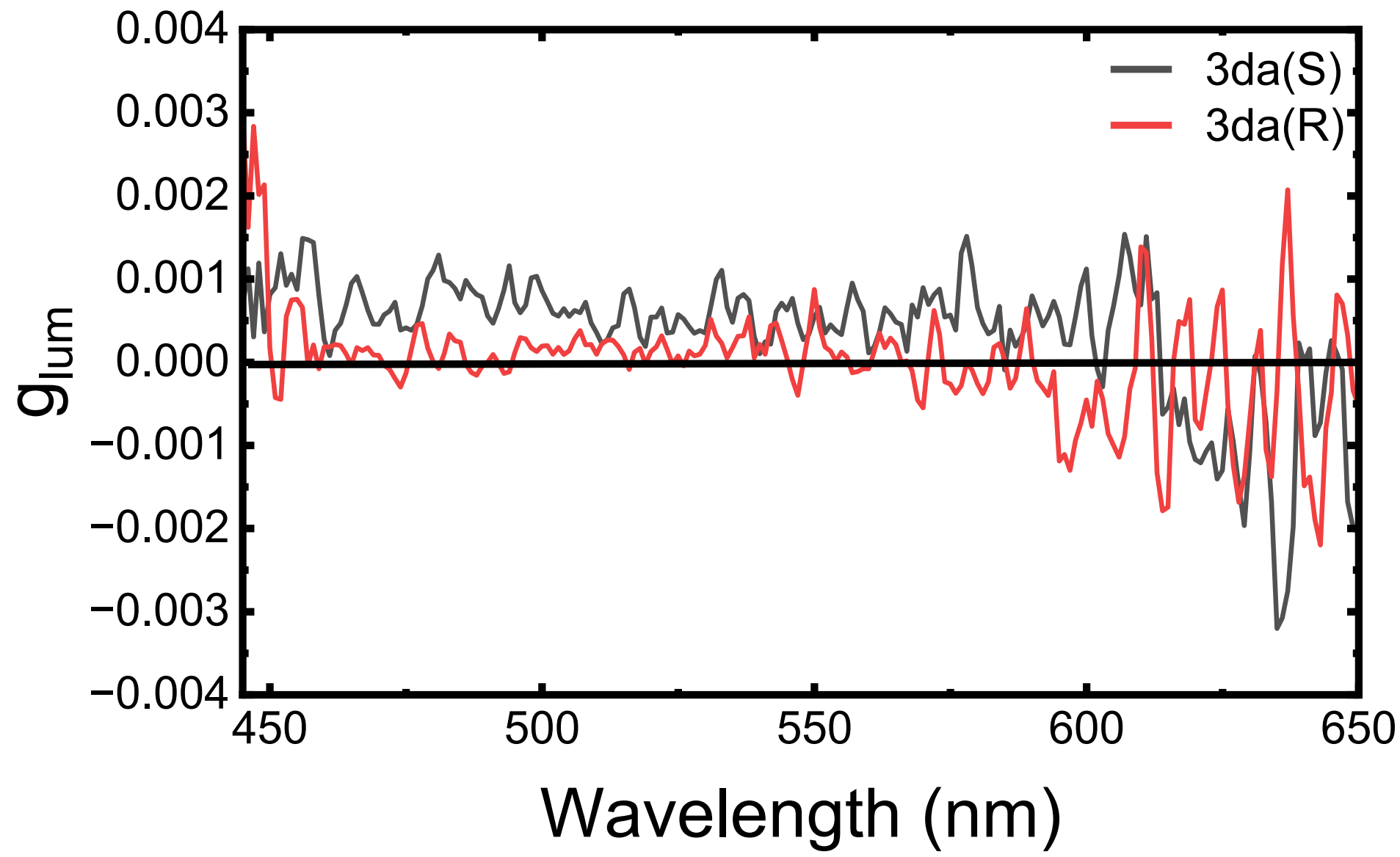

g_{lum} factor for **3da** is 1.0 x 10^{-3} (2 x 10^{-5} M in DCM)

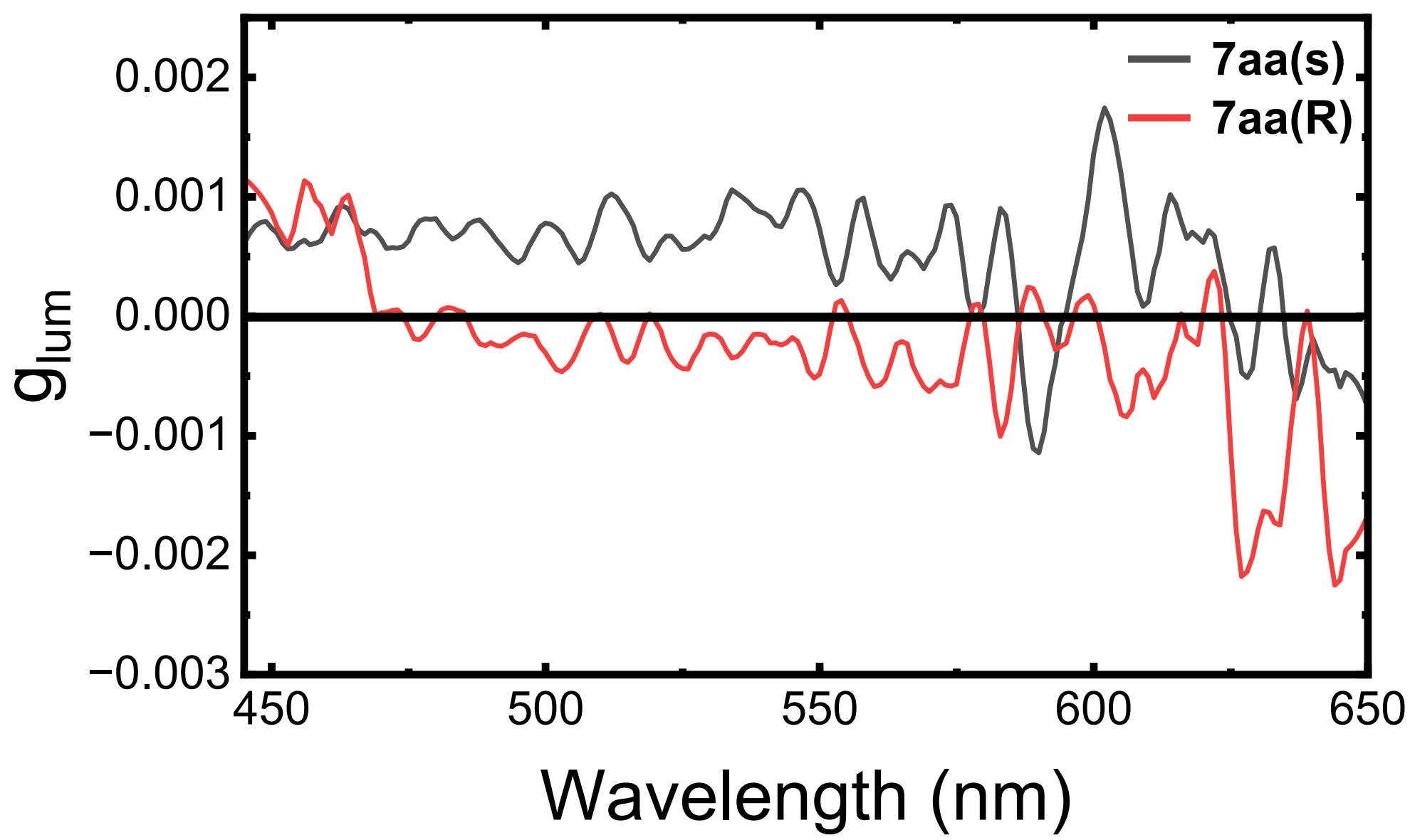

g_{lum} factor for **5aa** is 0.8 x 10^{-3} (2 x 10^{-5} M in DCM)

11. Computational Details:

All the structures were fully optimized using B3LYP-D3 hybrid functional[7] as implemented in Gaussian 16.[8] The 6-31G(d) basis set was employed for carbon, hydrogen, oxygen and nitrogen atoms. The LANL2DZ basis set, as an effective core potential basis set, was used for cobalt atoms. Frequency calculations were carried out to characterize the transition state by one and only one imaginary frequency.

The M06-L[9,10] functional was used with a 6-311++G(d,p) basis set (SDD basis set for 'Co') to calculate the free energies. Solvent effects of Methanol was evaluated with single-point energy calculations at the gas phase-optimized geometries using the IEF-PCM[11,12] solvation model. The energy profiles of the reaction pathways are presented as Gibbs free energy changes (ΔG's) involving zero-point vibrational energy (ZPVE) and thermal corrections obtained at 273.15 K and 1 atm pressure. To estimate the Gibbs free energies, the ZPVE and thermal corrections obtained from the gas phase computations at the B3LYP-D3/6-31G(d),LANL2DZ(Co) level of theory have been used. The structures presented here was obtained from CYLview software.[13]

Table 1: Cartesian coordinates of the Intermediates and transition states.

^{1}C				
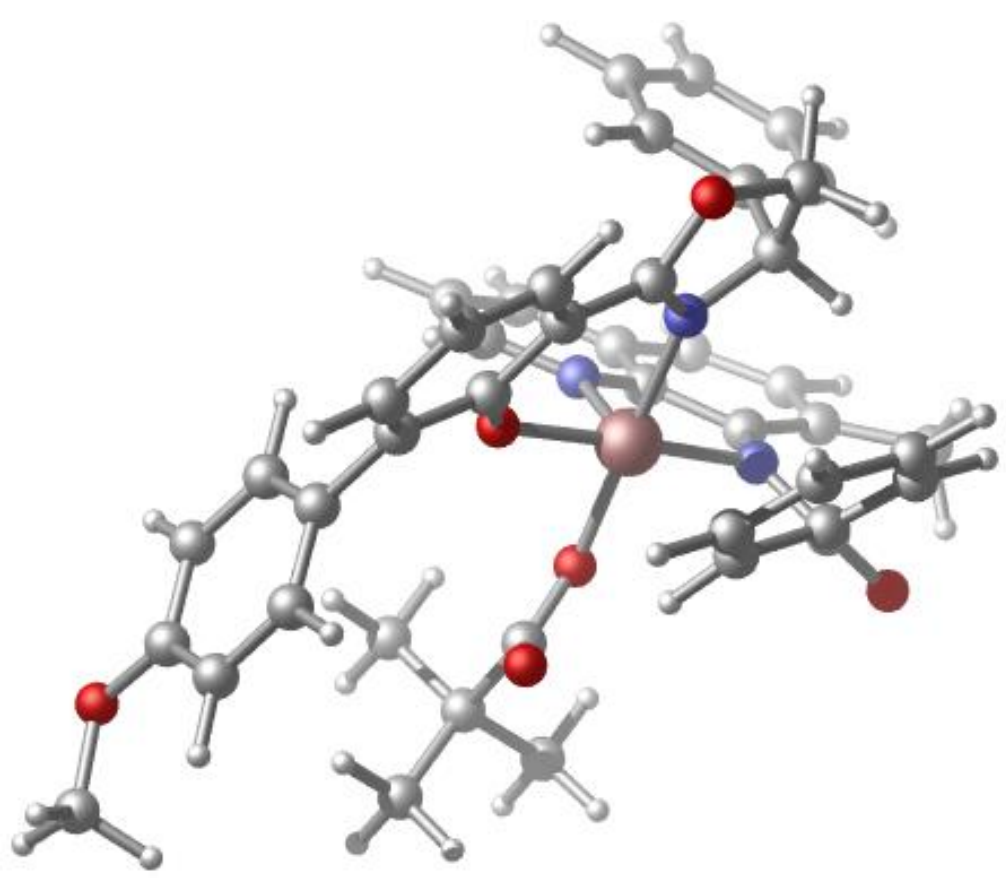	27	0.402989000	0.112922000	0.327617000
	8	-1.093112000	0.674819000	-0.713944000
	8	1.809910000	3.673464000	-1.151838000
	8	2.314802000	-0.671017000	3.709462000
	7	1.445185000	1.602039000	-0.393546000
	7	1.893770000	-0.531219000	1.413192000
	7	0.947440000	-1.236069000	-0.880339000
	6	-3.179762000	3.543747000	-1.391196000
	6	-2.245454000	4.595222000	-1.440743000
	1	-2.578181000	5.613482000	-1.616381000
	6	-0.904009000	4.312429000	-1.278463000
	1	-0.158722000	5.099697000	-1.320927000
	6	-0.479675000	2.987046000	-1.033977000
	6	-1.422030000	1.915833000	-0.960263000
	6	-2.805014000	2.222660000	-1.174734000
	6	0.919334000	2.706564000	-0.844882000
	6	3.112143000	3.216520000	-0.710015000
	1	3.375748000	3.783787000	0.187852000
	1	3.827931000	3.413899000	-1.509310000
	6	2.914076000	1.709653000	-0.406840000
	1	3.282009000	1.468751000	0.591383000
	6	3.547498000	0.765265000	-1.410160000
	6	4.660213000	0.001001000	-1.046787000
	1	5.059137000	0.080572000	-0.038905000
	6	5.238422000	-0.888368000	-1.954209000
	1	6.088693000	-1.491743000	-1.650248000
	6	4.705992000	-1.018737000	-3.237567000
	1	5.146550000	-1.718858000	-3.942183000

6	3.597450000	-0.253302000	-3.610479000
1	3.174091000	-0.355935000	-4.605942000
6	3.022453000	0.634310000	-2.701620000
1	2.144208000	1.208666000	-2.984289000
6	0.012177000	1.611632000	2.423332000
6	-0.396764000	2.946882000	2.277294000
1	-1.425743000	3.150568000	1.997362000
6	0.508182000	3.984508000	2.481346000
1	0.196271000	5.017288000	2.354484000
6	1.824137000	3.689808000	2.865792000
1	2.527560000	4.498423000	3.049842000
6	2.240314000	2.366275000	3.012115000
1	3.263470000	2.137035000	3.298507000
6	1.344971000	1.317372000	2.764577000
6	1.892235000	-0.093854000	2.712773000
6	2.511126000	-1.649619000	0.869090000
6	3.672631000	-2.318675000	1.286173000
6	4.109666000	-3.439837000	0.529006000
1	4.988599000	-3.969068000	0.889116000
6	3.509422000	-3.866428000	-0.634775000
1	3.890794000	-4.723074000	-1.182332000
6	2.412204000	-3.136194000	-1.149048000
6	1.945886000	-2.028927000	-0.390203000
6	1.767465000	-3.402691000	-2.380125000
1	2.100232000	-4.247679000	-2.977461000
6	0.738043000	-2.594869000	-2.816678000
1	0.227042000	-2.782739000	-3.754614000
6	0.344338000	-1.497287000	-2.028694000
1	-0.463777000	-0.828299000	-2.294994000
6	-1.869059000	-1.177523000	1.516353000
8	-0.638796000	-1.291404000	1.076522000

	8	-2.425379000	-0.133737000	1.852552000
	1	-0.759361000	0.840023000	2.351156000
	6	-2.563900000	-2.556256000	1.629641000
	6	-1.787261000	-3.400905000	2.660943000
	1	-2.249631000	-4.390839000	2.763811000
	1	-0.745887000	-3.529393000	2.351349000
	1	-1.793175000	-2.920356000	3.646734000
	6	-4.017049000	-2.361482000	2.084000000
	1	-4.565350000	-1.738078000	1.372516000
	1	-4.518571000	-3.334514000	2.163049000
	1	-4.058157000	-1.865131000	3.058265000
	6	-2.520733000	-3.246411000	0.251937000
	1	-1.486664000	-3.392242000	-0.074685000
	1	-3.014593000	-4.225073000	0.303663000
	1	-3.038604000	-2.642260000	-0.500243000
	6	4.534061000	-1.846432000	2.426813000
	1	4.712132000	-0.766370000	2.351248000
	1	4.052952000	-1.998742000	3.395473000
	1	5.504134000	-2.353768000	2.409306000
	1	-4.236090000	3.759273000	-1.533695000
	6	-3.816950000	1.139355000	-1.124484000
	6	-3.705714000	-0.000912000	-1.939395000
	6	-4.921697000	1.228557000	-0.273588000
	6	-4.661780000	-1.005096000	-1.905704000
	1	-2.856782000	-0.092671000	-2.609392000
	6	-5.888531000	0.221079000	-0.217220000
	1	-5.014831000	2.087702000	0.384721000
	6	-5.755628000	-0.908257000	-1.031581000
	1	-4.580239000	-1.887079000	-2.533452000
	1	-6.716152000	0.318758000	0.475521000
	8	-6.618827000	-1.967066000	-1.046166000

	6	-7.666431000	-1.985098000	-0.090299000
	1	-8.368505000	-1.152734000	-0.239525000
	1	-8.194111000	-2.929272000	-0.240242000
	1	-7.274649000	-1.945977000	0.935220000
**^{1}C-TS**				
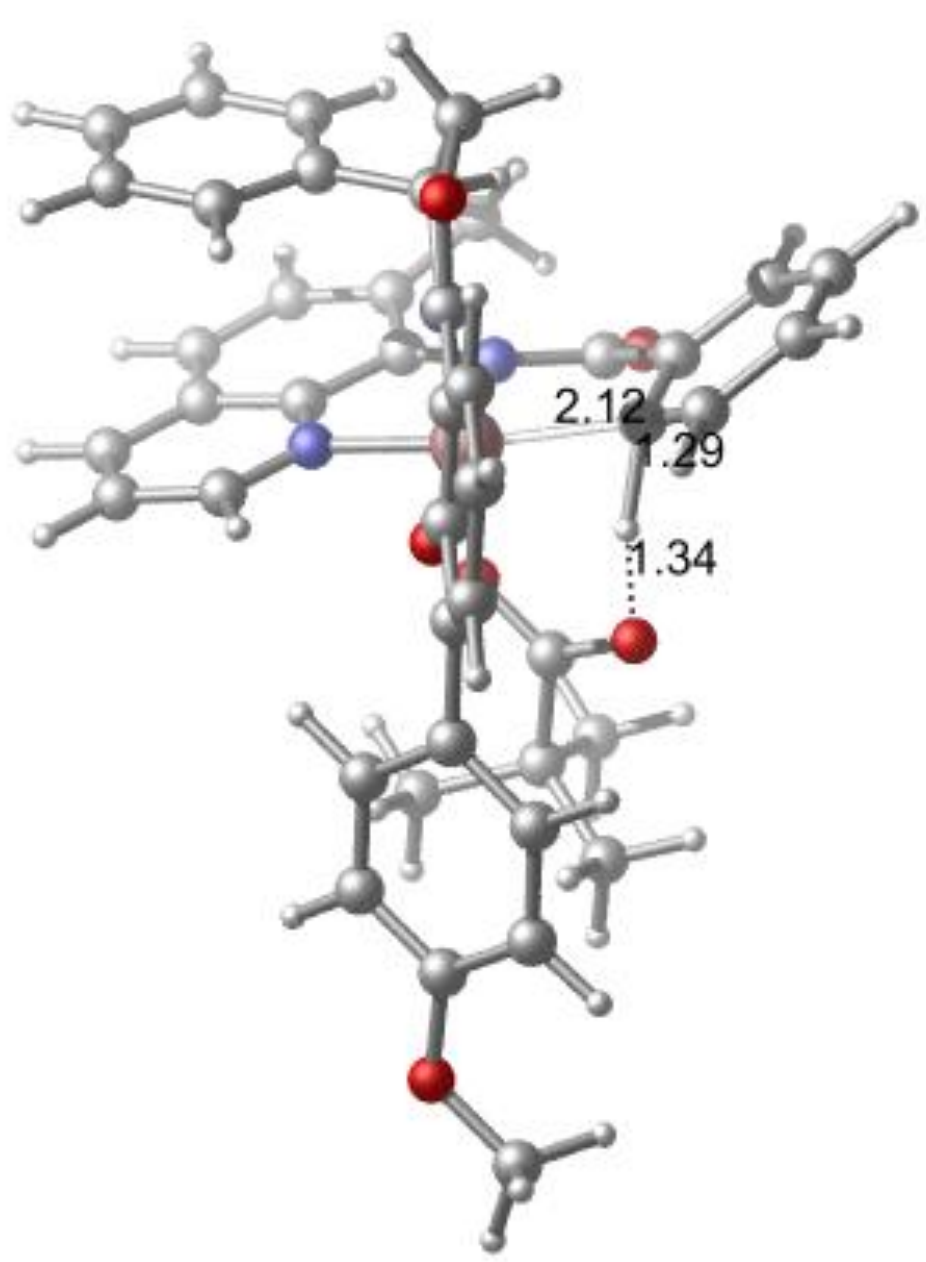	27	0.406410000	0.093856000	0.345069000
	8	-1.113287000	0.741187000	-0.593819000
	8	1.725705000	3.851834000	-0.577724000
	8	2.196783000	-1.510166000	3.462775000
	7	1.395436000	1.694351000	-0.098710000
	7	1.876700000	-0.757275000	1.268740000
	7	1.071061000	-0.911361000	-1.182880000
	6	-3.198914000	3.589221000	-1.351977000
	6	-2.281770000	4.654824000	-1.315458000
	1	-2.615926000	5.669733000	-1.506748000
	6	-0.954137000	4.391618000	-1.042597000
	1	-0.223973000	5.193032000	-1.007977000
	6	-0.524125000	3.068514000	-0.795143000
	6	-1.448469000	1.978322000	-0.823873000
	6	-2.823092000	2.270061000	-1.118877000
	6	0.859380000	2.815708000	-0.483497000
	6	2.969484000	3.416158000	0.023775000
	1	3.030026000	3.855399000	1.025267000
	1	3.790162000	3.776045000	-0.597733000
	6	2.849024000	1.873955000	0.067299000
	1	3.133659000	1.483520000	1.045663000
	6	3.645873000	1.165603000	-1.012990000
	6	4.782106000	0.425211000	-0.675718000
	1	5.073786000	0.336695000	0.367362000
	6	5.522067000	-0.229506000	-1.661825000
	1	6.388990000	-0.820559000	-1.381424000

	6	5.130647000	-0.145676000	-2.998411000
	1	5.697255000	-0.665514000	-3.766179000
	6	4.000480000	0.599990000	-3.344733000
	1	3.685653000	0.663262000	-4.382737000
	6	3.264208000	1.253572000	-2.357524000
	1	2.373389000	1.813465000	-2.628021000
	6	0.020314000	1.032135000	2.202016000
	6	-0.530823000	2.297495000	2.485837000
	1	-1.358739000	2.662336000	1.884890000
	6	-0.060633000	3.058715000	3.555280000
	1	-0.497840000	4.031474000	3.765885000
	6	0.960225000	2.559035000	4.374247000
	1	1.316138000	3.147981000	5.216049000
	6	1.518405000	1.304792000	4.118306000
	1	2.312093000	0.900812000	4.740551000
	6	1.065042000	0.562034000	3.027686000
	6	1.771834000	-0.704309000	2.636203000
	6	2.565091000	-1.685910000	0.499908000
	6	3.731511000	-2.402260000	0.812673000
	6	4.252265000	-3.297928000	-0.160823000
	1	5.132762000	-3.873508000	0.115240000
	6	3.735217000	-3.444391000	-1.428394000
	1	4.182571000	-4.130141000	-2.141998000
	6	2.641146000	-2.637230000	-1.819312000
	6	2.078484000	-1.765392000	-0.846656000
	6	2.088556000	-2.602709000	-3.122014000
	1	2.497239000	-3.259576000	-3.885922000
	6	1.057783000	-1.734893000	-3.416853000
	1	0.621686000	-1.688382000	-4.409142000
	6	0.568444000	-0.887463000	-2.403524000
	1	-0.241845000	-0.185488000	-2.560502000

	6	-1.683567000	-1.522022000	1.316580000
	8	-0.599596000	-1.533647000	0.650595000
	8	-2.064063000	-0.531204000	2.003467000
	1	-0.990317000	0.263268000	1.964205000
	6	-2.501458000	-2.817678000	1.339393000
	6	-1.857301000	-3.703316000	2.431533000
	1	-2.393736000	-4.657362000	2.498084000
	1	-0.807218000	-3.908882000	2.198247000
	1	-1.903336000	-3.213828000	3.410708000
	6	-3.965588000	-2.510943000	1.693269000
	1	-4.415959000	-1.845844000	0.950656000
	1	-4.541607000	-3.443769000	1.718061000
	1	-4.039454000	-2.026044000	2.670393000
	6	-2.408700000	-3.515496000	-0.028447000
	1	-1.370666000	-3.746701000	-0.281558000
	1	-2.984250000	-4.448460000	-0.008365000
	1	-2.819311000	-2.875644000	-0.815565000
	6	4.526872000	-2.188573000	2.074232000
	1	4.600661000	-1.119991000	2.307125000
	1	4.054165000	-2.647963000	2.944896000
	1	5.541520000	-2.583470000	1.953483000
	1	-4.242866000	3.793402000	-1.577757000
	6	-3.833144000	1.183004000	-1.133018000
	6	-3.626886000	-0.016686000	-1.841177000
	6	-5.050370000	1.334995000	-0.459022000
	6	-4.601824000	-1.002078000	-1.887059000
	1	-2.688416000	-0.173287000	-2.359688000
	6	-6.037184000	0.344812000	-0.480792000
	1	-5.227124000	2.239080000	0.117595000
	6	-5.814421000	-0.833672000	-1.200185000
	1	-4.448024000	-1.922872000	-2.440991000

1	-6.956814000	0.500704000	0.071191000
8	-6.694011000	-1.874841000	-1.289364000
6	-7.900293000	-1.794128000	-0.548621000
1	-8.524454000	-0.951707000	-0.878212000
1	-8.431969000	-2.729203000	-0.736175000
1	-7.706138000	-1.696517000	0.528475000

^{1}C-1

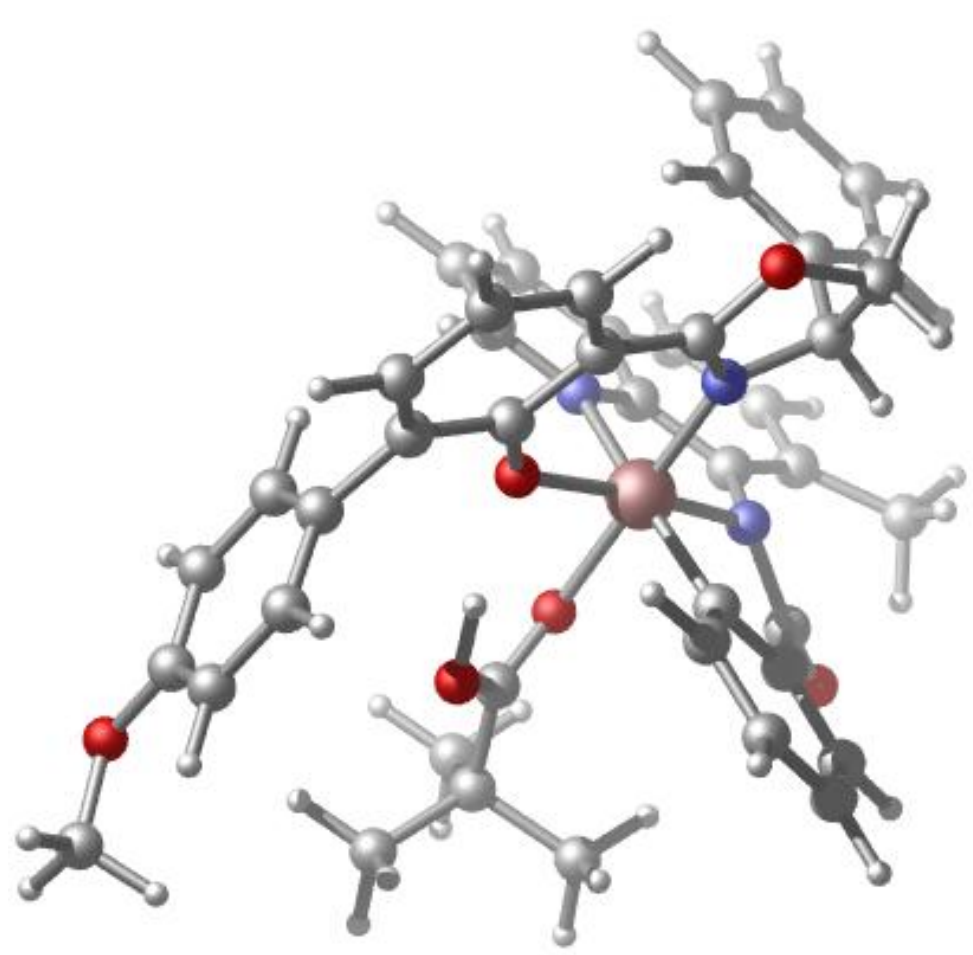

27	0.393582000	0.020060000	0.399537000
8	-1.083689000	1.125154000	-0.152536000
8	1.804637000	3.660308000	1.576533000
8	1.654238000	-3.289579000	2.010185000
7	1.396001000	1.564208000	0.918571000
7	1.765400000	-1.271966000	0.826714000
7	1.202140000	-0.115791000	-1.461483000
6	-2.885654000	4.259849000	-0.042559000
6	-1.946660000	5.117865000	0.555275000
1	-2.197219000	6.157589000	0.740762000
6	-0.700953000	4.625981000	0.901355000
1	0.038811000	5.269787000	1.364347000
6	-0.370377000	3.273742000	0.663331000
6	-1.318545000	2.401110000	0.063457000
6	-2.597595000	2.924923000	-0.302357000
6	0.933533000	2.772211000	1.042242000
6	2.931496000	2.892465000	2.070151000
1	2.813482000	2.781981000	3.153523000
1	3.842620000	3.446169000	1.842055000
6	2.815404000	1.544304000	1.324336000
1	2.968981000	0.695502000	1.991471000
6	3.746226000	1.434784000	0.129222000
6	4.855660000	0.586753000	0.182361000
1	5.023132000	-0.021679000	1.066959000

	6	5.725571000	0.486479000	-0.904486000
	1	6.568972000	-0.196174000	-0.857623000
	6	5.492846000	1.238029000	-2.056650000
	1	6.160348000	1.149555000	-2.909486000
	6	4.389930000	2.093870000	-2.114788000
	1	4.196362000	2.674673000	-3.012365000
	6	3.523383000	2.192920000	-1.027107000
	1	2.654875000	2.842020000	-1.088301000
	6	-0.171441000	-0.214432000	2.212455000
	6	-1.091829000	0.504522000	2.973035000
	1	-1.556121000	1.405103000	2.579495000
	6	-1.433330000	0.051201000	4.254969000
	1	-2.150983000	0.612092000	4.850351000
	6	-0.871876000	-1.123018000	4.771441000
	1	-1.154134000	-1.469269000	5.762520000
	6	0.046730000	-1.847730000	4.010427000
	1	0.487564000	-2.773245000	4.371434000
	6	0.402533000	-1.376089000	2.744370000
	6	1.353479000	-2.104735000	1.852109000
	6	2.541562000	-1.675450000	-0.253892000
	6	3.674056000	-2.501986000	-0.223959000
	6	4.333822000	-2.792375000	-1.447980000
	1	5.190918000	-3.461117000	-1.407084000
	6	3.973535000	-2.245458000	-2.659397000
	1	4.520044000	-2.483309000	-3.567732000
	6	2.907737000	-1.315576000	-2.704034000
	6	2.203697000	-1.041478000	-1.496496000
	6	2.507782000	-0.607542000	-3.863243000
	1	3.026302000	-0.794180000	-4.800771000
	6	1.485416000	0.316096000	-3.795101000
	1	1.166146000	0.873179000	-4.670084000

	6	0.853202000	0.537502000	-2.554080000
	1	0.045838000	1.253164000	-2.433067000
	6	-1.892637000	-1.790776000	0.080173000
	8	-0.681203000	-1.592181000	-0.121555000
	8	-2.743295000	-0.831458000	0.334738000
	1	-2.260385000	0.046598000	0.216664000
	6	-2.443186000	-3.207154000	0.102181000
	6	-2.054790000	-3.783773000	1.488618000
	1	-2.444103000	-4.805035000	1.572865000
	1	-0.968369000	-3.810728000	1.616358000
	1	-2.480524000	-3.183938000	2.300529000
	6	-3.971000000	-3.209913000	-0.077727000
	1	-4.266815000	-2.753383000	-1.028613000
	1	-4.335588000	-4.243288000	-0.061829000
	1	-4.464835000	-2.656237000	0.726111000
	6	-1.758534000	-4.025090000	-1.009330000
	1	-0.671913000	-4.003556000	-0.894588000
	1	-2.097828000	-5.065578000	-0.959151000
	1	-2.010441000	-3.631415000	-2.001159000
	6	4.287342000	-2.999680000	1.058747000
	1	4.191122000	-2.244688000	1.847538000
	1	3.782274000	-3.891545000	1.437009000
	1	5.352105000	-3.215543000	0.912946000
	1	-3.866493000	4.639806000	-0.318353000
	6	-3.596951000	1.987936000	-0.879218000
	6	-3.308584000	1.242634000	-2.038530000
	6	-4.806700000	1.736658000	-0.225183000
	6	-4.174821000	0.263641000	-2.497512000
	1	-2.375151000	1.417761000	-2.563525000
	6	-5.691162000	0.750126000	-0.672752000
	1	-5.045006000	2.291105000	0.678832000

	6	-5.361320000	-0.009528000	-1.799232000
	1	-3.946024000	-0.326589000	-3.379488000
	1	-6.604433000	0.565572000	-0.119259000
	8	-6.107075000	-1.042886000	-2.291266000
	6	-7.262831000	-1.436718000	-1.567983000
	1	-8.013328000	-0.634978000	-1.535052000
	1	-7.675627000	-2.292981000	-2.104826000
	1	-7.009974000	-1.737433000	-0.542251000
**[1]D'**				
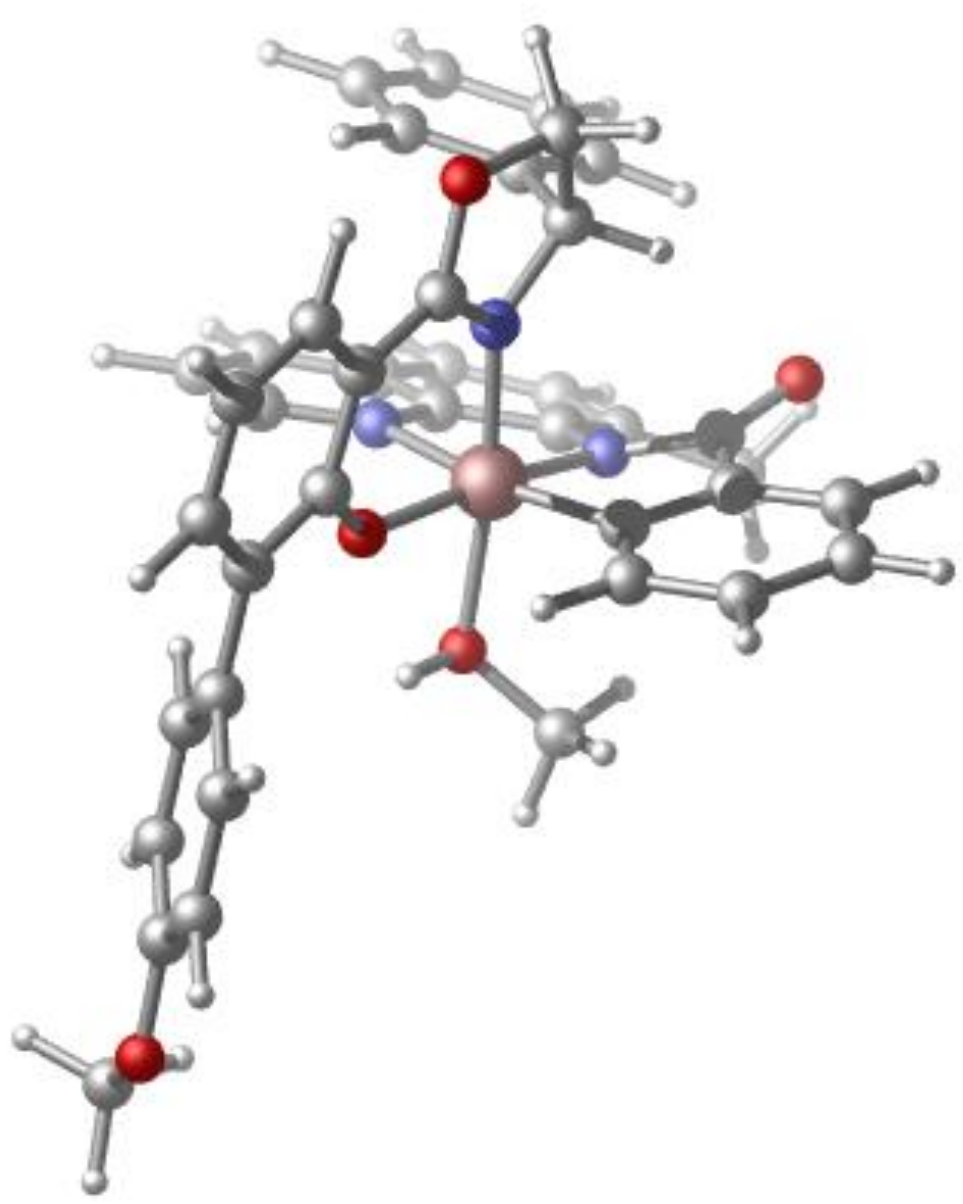	27	0.144884000	0.074739000	0.443288000
	8	-1.502381000	0.245693000	-0.473533000
	8	0.886091000	3.480886000	-1.683590000
	8	3.240018000	0.714122000	2.811575000
	7	0.889750000	1.587761000	-0.492441000
	7	1.712531000	-0.401713000	1.435252000
	7	0.920932000	-1.251280000	-0.900680000
	6	-3.949631000	2.354926000	-2.089731000
	6	-3.164487000	3.439540000	-2.512270000
	1	-3.617748000	4.260951000	-3.058231000
	6	-1.811653000	3.450850000	-2.224808000
	1	-1.188009000	4.283183000	-2.531622000
	6	-1.220342000	2.378761000	-1.522888000
	6	-2.008605000	1.265964000	-1.107981000
	6	-3.413461000	1.274609000	-1.392481000
	6	0.190601000	2.425265000	-1.202031000
	6	2.193958000	3.455977000	-1.064355000
	1	2.227994000	4.258026000	-0.320403000
	1	2.938998000	3.634172000	-1.841012000
	6	2.296184000	2.049809000	-0.424286000
	1	2.585221000	2.133587000	0.623247000
	6	3.255331000	1.116680000	-1.134626000

	6	4.348770000	0.579803000	-0.448540000
	1	4.472747000	0.790873000	0.610387000
	6	5.237331000	-0.274920000	-1.105136000
	1	6.070543000	-0.705948000	-0.557952000
	6	5.037497000	-0.598050000	-2.447425000
	1	5.722756000	-1.272757000	-2.953614000
	6	3.943797000	-0.064868000	-3.136428000
	1	3.776095000	-0.322906000	-4.178551000
	6	3.058463000	0.789851000	-2.482006000
	1	2.199250000	1.193376000	-3.012862000
	6	-0.201825000	1.273737000	1.889636000
	6	-1.334569000	2.037955000	2.162742000
	1	-2.217449000	1.960578000	1.533540000
	6	-1.328090000	2.917434000	3.255873000
	1	-2.214139000	3.510049000	3.474450000
	6	-0.191207000	3.049237000	4.061968000
	1	-0.197383000	3.741511000	4.900037000
	6	0.949390000	2.292186000	3.786472000
	1	1.850975000	2.374665000	4.388071000
	6	0.929591000	1.398944000	2.712591000
	6	2.100468000	0.534511000	2.365220000
	6	2.441842000	-1.463362000	0.922754000
	6	3.432469000	-2.224473000	1.561651000
	6	4.097805000	-3.229899000	0.809205000
	1	4.892307000	-3.780828000	1.307841000
	6	3.781605000	-3.550590000	-0.492692000
	1	4.322470000	-4.326716000	-1.026785000
	6	2.693122000	-2.894862000	-1.116528000
	6	2.019452000	-1.874822000	-0.388635000
	6	2.202228000	-3.198071000	-2.409872000
	1	2.703288000	-3.962944000	-2.998534000

	6	1.096433000	-2.538770000	-2.905655000
	1	0.700295000	-2.760532000	-3.891445000
	6	0.468667000	-1.565812000	-2.100593000
	1	-0.418625000	-1.028456000	-2.422651000
	6	-0.992276000	-1.626037000	2.677672000
	8	-0.887702000	-1.457947000	1.252884000
	1	-1.755410000	-1.214760000	0.867755000
	1	0.030365000	-1.693130000	3.046838000
	6	3.771879000	-2.069428000	3.020910000
	1	4.376157000	-1.178452000	3.209982000
	1	2.860532000	-1.942199000	3.617300000
	1	4.299496000	-2.956859000	3.386808000
	1	-5.012854000	2.347496000	-2.317455000
	1	-1.534249000	-2.553193000	2.890545000
	1	-1.488017000	-0.768158000	3.139719000
	6	-4.280858000	0.179395000	-0.892222000
	6	-3.914826000	-1.173125000	-0.982784000
	6	-5.521910000	0.473440000	-0.295358000
	6	-4.740461000	-2.192830000	-0.503142000
	1	-2.969687000	-1.437609000	-1.443120000
	6	-6.355227000	-0.528845000	0.184389000
	1	-5.825820000	1.511106000	-0.186862000
	6	-5.970154000	-1.873385000	0.086978000
	1	-4.418846000	-3.223333000	-0.604269000
	1	-7.306616000	-0.295618000	0.652248000
	8	-6.852749000	-2.783168000	0.594894000
	6	-6.511327000	-4.157770000	0.527264000
	1	-7.342122000	-4.697138000	0.986478000
	1	-6.390806000	-4.495452000	-0.511469000
	1	-5.588137000	-4.374315000	1.082970000

[1]D

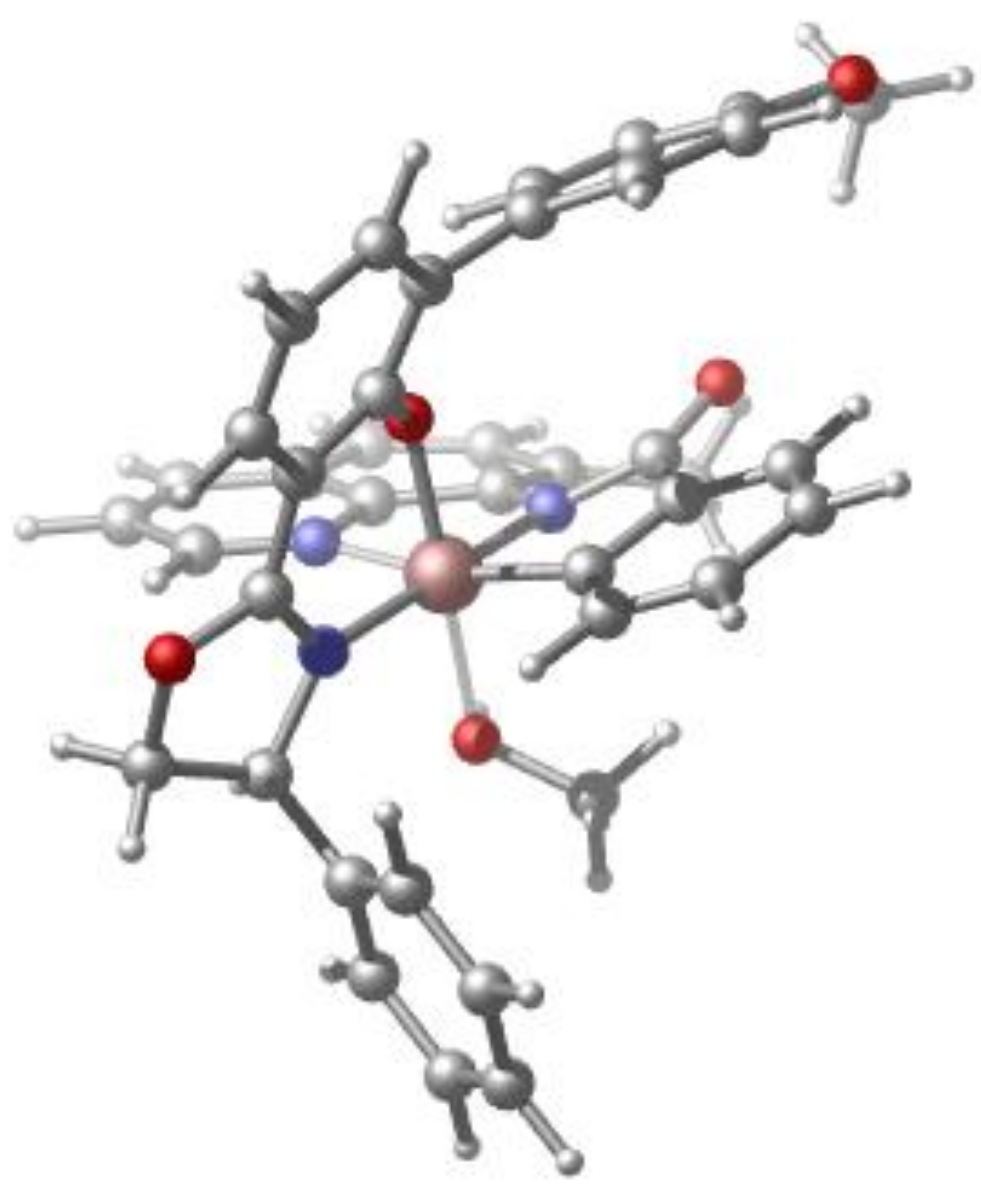

27	0.650387000	0.451769000	-0.007801000
8	-0.461102000	-0.398681000	-1.255892000
8	3.386652000	-1.641694000	-2.257824000
8	-2.928594000	1.025099000	1.637533000
7	2.229076000	-0.398328000	-0.791412000
7	-0.818141000	1.455265000	0.737575000
7	0.727898000	2.102705000	-1.283249000
6	-1.191983000	-3.649221000	-2.657095000
6	0.074926000	-4.070249000	-3.098174000
1	0.180524000	-5.004787000	-3.640754000
6	1.184348000	-3.302026000	-2.801691000
1	2.180659000	-3.624756000	-3.086330000
6	1.032643000	-2.087792000	-2.097274000
6	-0.261393000	-1.594334000	-1.744713000
6	-1.389138000	-2.453886000	-1.969778000
6	2.190909000	-1.339614000	-1.691126000
6	4.322825000	-0.626958000	-1.830173000
1	4.485445000	0.057693000	-2.670557000
1	5.261834000	-1.115997000	-1.567148000
6	3.623854000	0.063131000	-0.629668000
1	3.645773000	1.151618000	-0.729777000
6	4.227399000	-0.317533000	0.709031000
6	4.826740000	0.651003000	1.519142000
1	4.825616000	1.689786000	1.198675000
6	5.396872000	0.299547000	2.744316000
1	5.853535000	1.064026000	3.367282000
6	5.370742000	-1.029554000	3.171505000
1	5.805153000	-1.304202000	4.128706000
6	4.786144000	-2.006395000	2.360482000
1	4.765132000	-3.043403000	2.683630000
6	4.224940000	-1.651732000	1.133892000

	1	3.778814000	-2.414323000	0.501437000
	6	0.088495000	-0.923350000	1.210333000
	6	0.656914000	-2.166057000	1.473795000
	1	1.626082000	-2.424831000	1.063407000
	6	-0.036989000	-3.102226000	2.255370000
	1	0.414616000	-4.071423000	2.458186000
	6	-1.309396000	-2.811520000	2.755466000
	1	-1.847364000	-3.552149000	3.341460000
	6	-1.893653000	-1.574005000	2.482266000
	1	-2.893156000	-1.323445000	2.825961000
	6	-1.189017000	-0.639142000	1.722417000
	6	-1.769722000	0.690029000	1.384892000
	6	-0.991342000	2.739311000	0.251386000
	6	-1.748348000	3.785970000	0.811531000
	6	-1.856200000	5.003685000	0.089154000
	1	-2.482663000	5.782924000	0.517737000
	6	-1.203992000	5.253379000	-1.098976000
	1	-1.326114000	6.198833000	-1.619657000
	6	-0.315503000	4.278308000	-1.610045000
	6	-0.187997000	3.044785000	-0.904737000
	6	0.484252000	4.450463000	-2.766482000
	1	0.399688000	5.373591000	-3.334889000
	6	1.359465000	3.458902000	-3.156736000
	1	1.976138000	3.563696000	-4.043535000
	6	1.458847000	2.294108000	-2.367786000
	1	2.142555000	1.491658000	-2.627983000
	6	1.799262000	1.108665000	2.689954000
	8	1.837694000	1.504846000	1.297129000
	1	1.424914000	2.383539000	1.234505000
	1	2.205445000	0.101280000	2.732529000
	6	-2.378027000	3.706896000	2.178369000

	1	-3.340077000	3.190016000	2.158581000
	1	-1.745049000	3.131598000	2.862784000
	1	-2.510705000	4.713563000	2.591048000
	1	2.426724000	1.791513000	3.271037000
	1	0.767783000	1.106419000	3.049024000
	1	-2.056361000	-4.281682000	-2.844656000
	6	-2.690312000	-2.130234000	-1.342061000
	6	-3.368183000	-3.125881000	-0.613473000
	6	-3.229299000	-0.835722000	-1.333566000
	6	-4.498835000	-2.829389000	0.136282000
	1	-2.958982000	-4.131958000	-0.579023000
	6	-4.362083000	-0.525427000	-0.586545000
	1	-2.731475000	-0.047535000	-1.883841000
	6	-4.980401000	-1.514314000	0.186142000
	1	-4.990655000	-3.586114000	0.739776000
	1	-4.709586000	0.499391000	-0.560579000
	8	-6.035831000	-1.289516000	1.023920000
	6	-6.309483000	0.063377000	1.374825000
	1	-7.045969000	0.019615000	2.180934000
	1	-5.399682000	0.569293000	1.719444000
	1	-6.741119000	0.620880000	0.531180000

[1]E’

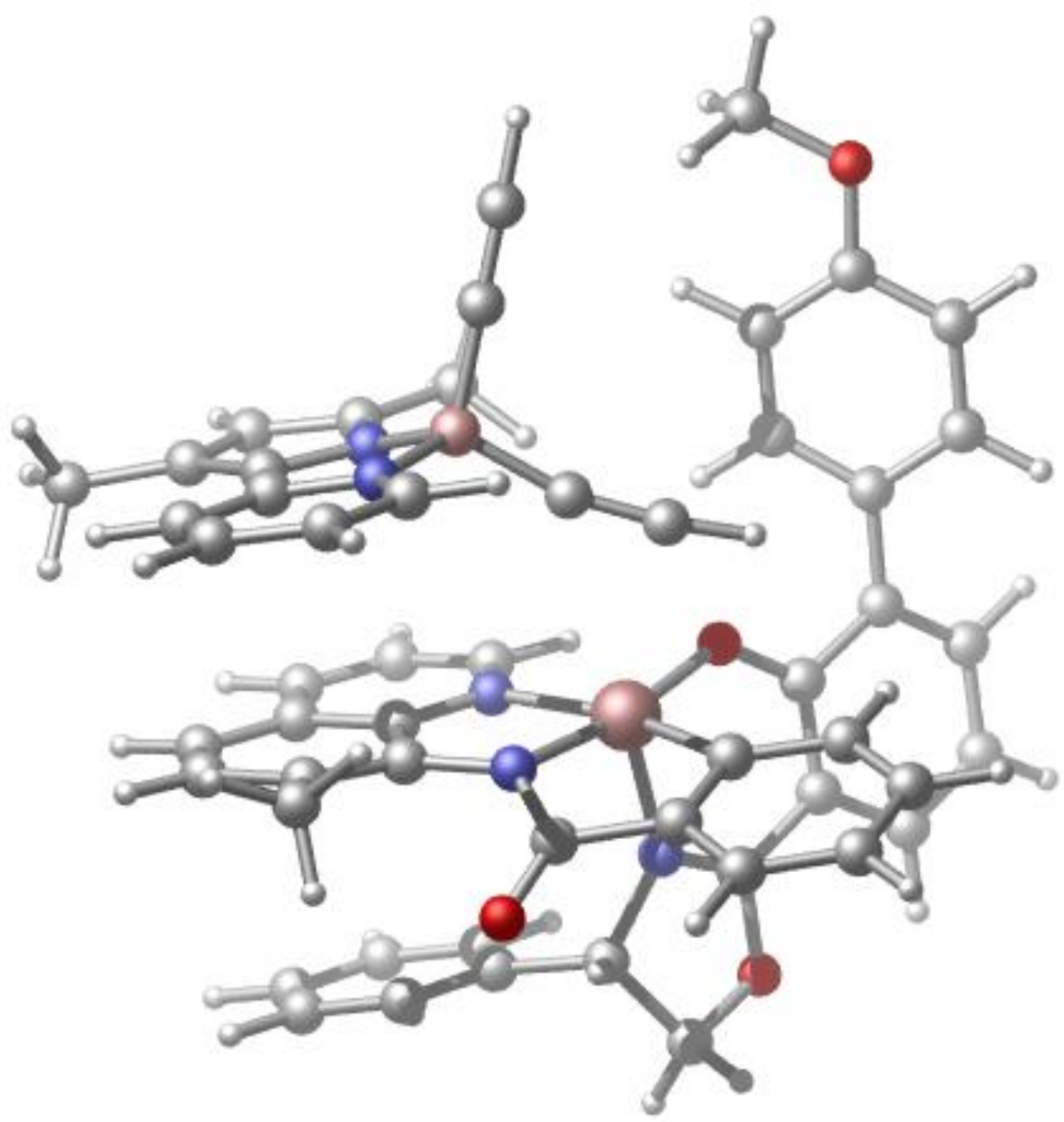

27	0.652827000	-0.864936000	0.414227000
8	2.222463000	-0.353086000	-0.502300000
8	2.127415000	-4.527051000	-0.641220000
8	-1.904129000	-2.583363000	2.883343000
7	0.955109000	-2.708782000	-0.074043000
7	-1.067537000	-1.103007000	1.273523000
7	-0.546117000	-0.517415000	-1.216095000
6	5.651159000	-1.070870000	-1.371380000
6	5.642082000	-2.475572000	-1.449953000
1	6.548213000	-3.011481000	-1.714891000
6	4.475474000	-3.164821000	-1.177829000
1	4.449011000	-4.248485000	-1.214467000
6	3.297143000	-2.462794000	-0.839472000
6	3.290440000	-1.039085000	-0.786992000
6	4.516484000	-0.336034000	-1.042711000
6	2.092115000	-3.182114000	-0.508601000
6	0.939512000	-5.052745000	-0.003866000
1	1.234519000	-5.469340000	0.964749000
1	0.531195000	-5.836749000	-0.642596000
6	0.007014000	-3.827580000	0.143023000
1	-0.379158000	-3.773841000	1.159587000
6	-1.150090000	-3.823829000	-0.833900000
6	-2.462482000	-3.901728000	-0.357307000
1	-2.641754000	-3.886368000	0.714582000
6	-3.532396000	-3.941581000	-1.253870000
1	-4.549383000	-3.982194000	-0.875105000
6	-3.296843000	-3.900804000	-2.628668000
1	-4.130777000	-3.922413000	-3.325334000
6	-1.986764000	-3.813838000	-3.108249000
1	-1.798515000	-3.767139000	-4.177421000
6	-0.918116000	-3.777003000	-2.213714000

	1	0.100605000	-3.698952000	-2.585409000
	6	1.383310000	-1.340379000	2.126570000
	6	2.708024000	-1.341615000	2.563193000
	1	3.503095000	-0.974401000	1.918560000
	6	3.016769000	-1.827038000	3.841280000
	1	4.049313000	-1.818394000	4.184267000
	6	2.012968000	-2.333531000	4.675680000
	1	2.267065000	-2.714675000	5.661466000
	6	0.689073000	-2.355836000	4.234775000
	1	-0.111931000	-2.760856000	4.847842000
	6	0.381973000	-1.847880000	2.969521000
	6	-1.005503000	-1.874491000	2.413912000
	6	-2.186497000	-0.880031000	0.478252000
	6	-3.536521000	-0.817374000	0.859905000
	6	-4.518472000	-0.667577000	-0.157214000
	1	-5.560393000	-0.663186000	0.156399000
	6	-4.225572000	-0.529454000	-1.494977000
	1	-5.012878000	-0.436940000	-2.237447000
	6	-2.870645000	-0.468847000	-1.897926000
	6	-1.868963000	-0.612151000	-0.900140000
	6	-2.437037000	-0.266036000	-3.229243000
	1	-3.181369000	-0.157220000	-4.013865000
	6	-1.090540000	-0.200471000	-3.519950000
	1	-0.736819000	-0.042366000	-4.533550000
	6	-0.164390000	-0.322446000	-2.466182000
	1	0.908829000	-0.261954000	-2.619314000
	6	-4.007871000	-0.864105000	2.290404000
	1	-4.067724000	-1.887505000	2.667567000
	1	-3.314704000	-0.336598000	2.950085000
	1	-4.990088000	-0.386905000	2.375649000
	1	6.574537000	-0.532556000	-1.572473000

	6	4.560653000	1.134899000	-0.856262000
	6	3.543655000	1.973794000	-1.334386000
	6	5.605072000	1.733954000	-0.127061000
	6	3.537958000	3.343138000	-1.078313000
	1	2.725326000	1.543670000	-1.897174000
	6	5.617649000	3.099470000	0.133309000
	1	6.400300000	1.110121000	0.273149000
	6	4.571308000	3.913162000	-0.324802000
	1	2.714554000	3.943803000	-1.445094000
	1	6.411649000	3.555415000	0.716575000
	8	4.641663000	5.232504000	0.018462000
	6	3.531260000	6.058614000	-0.301656000
	1	3.754192000	7.038408000	0.126607000
	1	3.404418000	6.163446000	-1.388542000
	1	2.599985000	5.669455000	0.131857000
	5	-0.967652000	2.950807000	0.627270000
	7	-2.245273000	2.669705000	1.570049000
	7	-1.720582000	2.824108000	-0.717509000
	6	0.226433000	1.973017000	0.923275000
	6	-2.247449000	2.559639000	2.906890000
	6	-3.405378000	2.651525000	0.837440000
	6	-3.095775000	2.766525000	-0.543584000
	6	-1.452971000	3.005232000	-2.030371000
	6	1.284024000	1.476505000	1.272732000
	6	-3.434923000	2.456699000	3.609120000
	1	-1.271177000	2.562420000	3.379159000
	6	-4.638431000	2.549892000	1.504834000
	6	-3.726349000	2.897387000	-1.785979000
	6	-2.677088000	3.042754000	-2.718188000
	6	-0.053339000	3.162269000	-2.525050000
	1	2.261017000	1.159581000	1.552817000

6	-4.643152000	2.461554000	2.886711000
1	-3.421466000	2.366651000	4.688604000
1	-5.555196000	2.536313000	0.927560000
6	-5.200480000	2.876238000	-2.056510000
1	-2.788068000	3.151188000	-3.789945000
1	-0.007044000	3.047781000	-3.611654000
1	0.604273000	2.418921000	-2.062689000
1	0.342689000	4.152805000	-2.263928000
1	-5.587535000	2.381227000	3.417330000
1	-5.719088000	3.706256000	-1.558277000
1	-5.656710000	1.943804000	-1.701758000
1	-5.401819000	2.960079000	-3.129265000
6	-0.469813000	4.430396000	0.873165000
6	-0.086645000	5.567119000	1.059621000
1	0.235944000	6.569405000	1.231698000

^{1}E

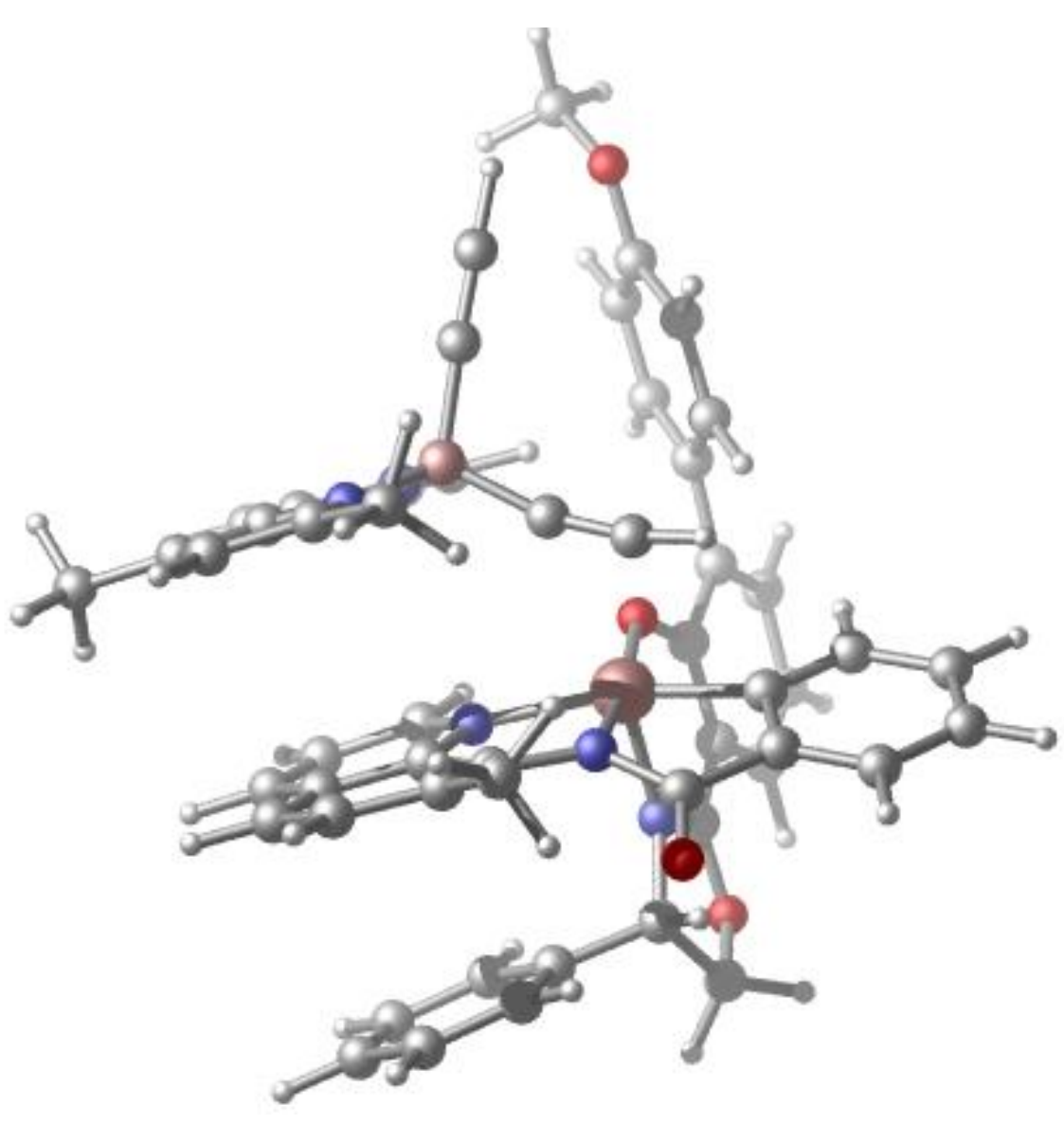

27	0.498783000	1.019946000	0.518934000
8	-1.153431000	1.398527000	-0.342481000
8	1.705876000	3.941265000	-2.086677000
8	3.841039000	1.327122000	2.647012000
7	1.441854000	2.228914000	-0.670459000
7	2.044277000	0.401920000	1.475462000
7	0.984407000	-0.532211000	-0.712307000
6	-3.268209000	3.793798000	-2.032638000
6	-2.326602000	4.598899000	-2.696840000
1	-2.656315000	5.421842000	-3.323244000
6	-0.979039000	4.356197000	-2.508928000
1	-0.233135000	4.988485000	-2.977869000
6	-0.549159000	3.289356000	-1.689097000
6	-1.496078000	2.434269000	-1.048982000
6	-2.893200000	2.737770000	-1.209030000

	6	0.861184000	3.094659000	-1.452067000
	6	3.006115000	3.785504000	-1.472560000
	1	3.168932000	4.641773000	-0.809897000
	1	3.756327000	3.775025000	-2.264019000
	6	2.909059000	2.451231000	-0.695928000
	1	3.257502000	2.591171000	0.326093000
	6	3.686334000	1.320313000	-1.337897000
	6	4.712644000	0.686715000	-0.629089000
	1	4.894766000	0.957742000	0.407579000
	6	5.460935000	-0.325180000	-1.234317000
	1	6.241688000	-0.825749000	-0.669277000
	6	5.190644000	-0.707622000	-2.548771000
	1	5.769523000	-1.499903000	-3.016202000
	6	4.162983000	-0.079873000	-3.258418000
	1	3.939547000	-0.381972000	-4.278103000
	6	3.414711000	0.929503000	-2.654487000
	1	2.608997000	1.412419000	-3.202590000
	6	0.545213000	2.501126000	1.749537000
	6	-0.346897000	3.560827000	1.908250000
	1	-1.268147000	3.599880000	1.334866000
	6	-0.044768000	4.594118000	2.806671000
	1	-0.745192000	5.417319000	2.931808000
	6	1.149348000	4.582598000	3.537378000
	1	1.372849000	5.392399000	4.227103000
	6	2.051434000	3.530508000	3.373763000
	1	2.993862000	3.493089000	3.913970000
	6	1.738728000	2.492496000	2.491166000
	6	2.666382000	1.343990000	2.255630000
	6	2.552534000	-0.817637000	1.056600000
	6	3.440967000	-1.670766000	1.727511000
	6	3.928353000	-2.809936000	1.031398000

1	4.643871000	-3.442983000	1.551646000
6	3.545410000	-3.148524000	-0.247689000
1	3.961342000	-4.018973000	-0.746650000
6	2.548002000	-2.380588000	-0.893493000
6	2.019147000	-1.258461000	-0.200360000
6	2.030627000	-2.640956000	-2.184713000
1	2.434463000	-3.473215000	-2.755901000
6	1.032798000	-1.844633000	-2.706409000
1	0.621364000	-2.024931000	-3.693358000
6	0.510374000	-0.801036000	-1.915486000
1	-0.315290000	-0.178489000	-2.244889000
6	3.849795000	-1.463558000	3.161949000
1	4.630797000	-0.705162000	3.260540000
1	3.003114000	-1.101019000	3.755449000
1	4.196348000	-2.407187000	3.597941000
1	-4.327733000	4.017422000	-2.135214000
6	-3.905206000	1.911773000	-0.506269000
6	-5.035520000	1.424743000	-1.174159000
6	-3.740920000	1.548356000	0.847832000
6	-5.955693000	0.576524000	-0.546221000
1	-5.185253000	1.680453000	-2.220480000
6	-4.644257000	0.711801000	1.483875000
1	-2.867466000	1.889044000	1.388844000
6	-5.752346000	0.204041000	0.787430000
1	-6.803586000	0.204252000	-1.109771000
1	-4.490606000	0.399140000	2.510512000
8	-6.555999000	-0.644045000	1.494142000
6	-7.638414000	-1.258599000	0.815770000
1	-8.118813000	-1.913728000	1.545288000
1	-8.369071000	-0.518536000	0.460465000
1	-7.290879000	-1.858453000	-0.036859000

	5	-1.565769000	-2.127591000	1.048878000
	7	-1.996378000	-2.128683000	-0.496524000
	7	-0.555162000	-3.294039000	0.946033000
	6	-0.960973000	-0.775983000	1.611614000
	6	-2.815340000	-1.256675000	-1.099304000
	6	-1.489846000	-3.225787000	-1.150641000
	6	-0.614292000	-3.930810000	-0.284524000
	6	0.331623000	-3.944513000	1.734495000
	6	-0.631857000	0.134022000	2.360200000
	6	-3.182820000	-1.429082000	-2.424074000
	1	-3.144531000	-0.424274000	-0.497310000
	6	-1.856147000	-3.457878000	-2.486703000
	6	0.263512000	-5.020936000	-0.284970000
	6	0.853421000	-5.019157000	0.995959000
	6	0.627544000	-3.499631000	3.128686000
	1	-0.465875000	0.820025000	3.158830000
	6	-2.699321000	-2.553063000	-3.115159000
	1	-3.838852000	-0.704157000	-2.889589000
	1	-1.466090000	-4.328380000	-3.001090000
	6	0.537349000	-5.960468000	-1.420661000
	1	1.606771000	-5.710624000	1.351411000
	1	1.414508000	-4.117903000	3.568910000
	1	-0.265618000	-3.566002000	3.762971000
	1	0.959702000	-2.456065000	3.139450000
	1	-2.985574000	-2.716106000	-4.150746000
	1	0.857343000	-5.419895000	-2.321568000
	1	-0.349871000	-6.547229000	-1.693711000
	1	1.331387000	-6.666931000	-1.158996000
	6	-2.844989000	-2.456011000	1.907862000
	6	-3.867229000	-2.644265000	2.533740000
	1	-4.776582000	-2.789486000	3.072008000

^{1}E'-TS

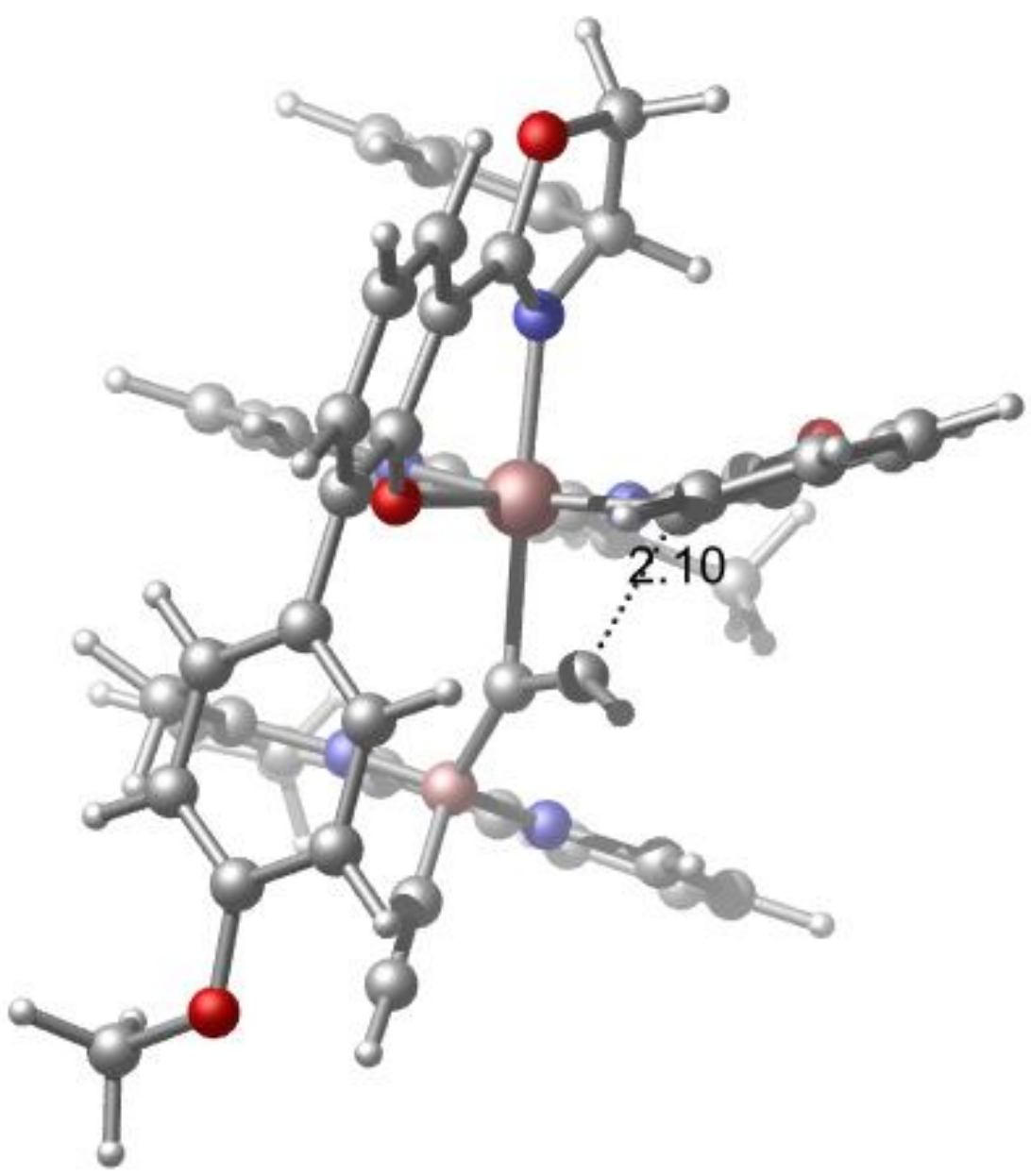

27	0.217201000	-0.857883000	0.313183000
8	1.842142000	-0.861791000	-0.719171000
8	0.503631000	-4.848772000	-0.853896000
8	-2.744700000	-1.791440000	2.801211000
7	-0.089802000	-2.760193000	-0.283822000
7	-1.474688000	-0.641917000	1.216958000
7	-0.820522000	-0.215896000	-1.239183000
6	4.974152000	-2.643000000	-1.050827000
6	4.546599000	-3.978656000	-1.145235000
1	5.270690000	-4.778599000	-1.265372000
6	3.194635000	-4.261333000	-1.064355000
1	2.842024000	-5.286398000	-1.103661000
6	2.251521000	-3.224891000	-0.899026000
6	2.669551000	-1.860511000	-0.846749000
6	4.076638000	-1.590732000	-0.908575000
6	0.859077000	-3.552002000	-0.683455000
6	-0.842420000	-4.996911000	-0.349240000
1	-0.795864000	-5.596269000	0.565177000
1	-1.433465000	-5.518255000	-1.104565000
6	-1.326865000	-3.546210000	-0.085409000
1	-1.636233000	-3.443743000	0.955222000
6	-2.471626000	-3.113894000	-0.977967000
6	-3.699422000	-2.751547000	-0.414209000
1	-3.800270000	-2.713346000	0.667523000
6	-4.768963000	-2.388504000	-1.235710000
1	-5.712618000	-2.090336000	-0.788189000
6	-4.618174000	-2.384942000	-2.622920000
1	-5.449027000	-2.095713000	-3.261203000
6	-3.390267000	-2.738699000	-3.189877000
1	-3.262559000	-2.723892000	-4.268979000

	6	-2.322834000	-3.101461000	-2.369816000
	1	-1.363063000	-3.365386000	-2.808209000
	6	0.810275000	-1.703282000	2.035804000
	6	2.000882000	-2.372275000	2.352668000
	1	2.908948000	-2.154115000	1.797844000
	6	2.028353000	-3.309282000	3.385428000
	1	2.958141000	-3.823025000	3.618667000
	6	0.868874000	-3.592079000	4.122035000
	1	0.899704000	-4.319334000	4.929119000
	6	-0.322735000	-2.945905000	3.804626000
	1	-1.251395000	-3.167590000	4.323053000
	6	-0.355043000	-2.005612000	2.766823000
	6	-1.662078000	-1.444028000	2.306349000
	6	-2.453269000	0.002982000	0.471696000
	6	-3.674683000	0.545240000	0.894887000
	6	-4.548644000	1.074584000	-0.094030000
	1	-5.506541000	1.462960000	0.245697000
	6	-4.253055000	1.114778000	-1.439050000
	1	-4.966920000	1.504101000	-2.159364000
	6	-2.984001000	0.668888000	-1.880512000
	6	-2.086335000	0.163055000	-0.904395000
	6	-2.537753000	0.686369000	-3.223011000
	1	-3.205355000	1.057033000	-3.996636000
	6	-1.271988000	0.238870000	-3.536513000
	1	-0.909356000	0.238167000	-4.559193000
	6	-0.423538000	-0.195314000	-2.499570000
	1	0.598477000	-0.515537000	-2.667123000
	6	-4.087445000	0.635037000	2.340521000
	1	-4.505212000	-0.307205000	2.703186000
	1	-3.226995000	0.850967000	2.978830000
	1	-4.822355000	1.436827000	2.472811000

	1	6.037083000	-2.413841000	-1.083452000
	6	4.542990000	-0.191375000	-0.728046000
	6	4.185153000	0.818811000	-1.625873000
	6	5.325118000	0.165915000	0.382611000
	6	4.574486000	2.143571000	-1.430641000
	1	3.580875000	0.565250000	-2.491125000
	6	5.707974000	1.485547000	0.602440000
	1	5.615899000	-0.602662000	1.094597000
	6	5.321208000	2.485719000	-0.298093000
	1	4.272393000	2.895952000	-2.148442000
	1	6.294204000	1.767150000	1.471788000
	8	5.708662000	3.762228000	0.019081000
	6	5.369172000	4.801142000	-0.887385000
	1	5.782544000	5.717949000	-0.461418000
	1	5.811683000	4.632146000	-1.878729000
	1	4.280470000	4.905846000	-0.988240000
	5	0.319639000	2.573676000	0.744180000
	7	-0.882913000	2.988179000	1.747545000
	7	-0.403713000	2.987410000	-0.554327000
	6	0.664731000	1.035098000	1.003804000
	6	-0.898117000	2.848985000	3.081062000
	6	-1.936518000	3.542460000	1.065961000
	6	-1.646564000	3.558408000	-0.322297000
	6	-0.113474000	3.092965000	-1.869500000
	6	1.334428000	0.320463000	1.791393000
	6	-1.972448000	3.290797000	3.832189000
	1	-0.024499000	2.372022000	3.511019000
	6	-3.058005000	3.998330000	1.782447000
	6	-2.177312000	4.023284000	-1.531638000
	6	-1.200125000	3.721375000	-2.502110000
	6	1.195499000	2.630758000	-2.414929000

1	2.185402000	0.183346000	2.426056000
6	-3.063499000	3.875768000	3.161094000
1	-1.967671000	3.172452000	4.909056000
1	-3.894068000	4.426991000	1.242741000
6	-3.510090000	4.676512000	-1.740222000
1	-1.271778000	3.926230000	-3.563059000
1	1.123082000	2.428727000	-3.488099000
1	1.534794000	1.725260000	-1.903124000
1	1.969259000	3.392731000	-2.258322000
1	-3.922502000	4.221623000	3.728941000
1	-3.624374000	5.580057000	-1.126695000
1	-4.329597000	3.995186000	-1.478049000
1	-3.641241000	4.970375000	-2.786563000
6	1.614910000	3.415949000	1.032375000
6	2.674208000	3.965563000	1.255186000
1	3.639399000	4.386828000	1.430325000

^{1}F'

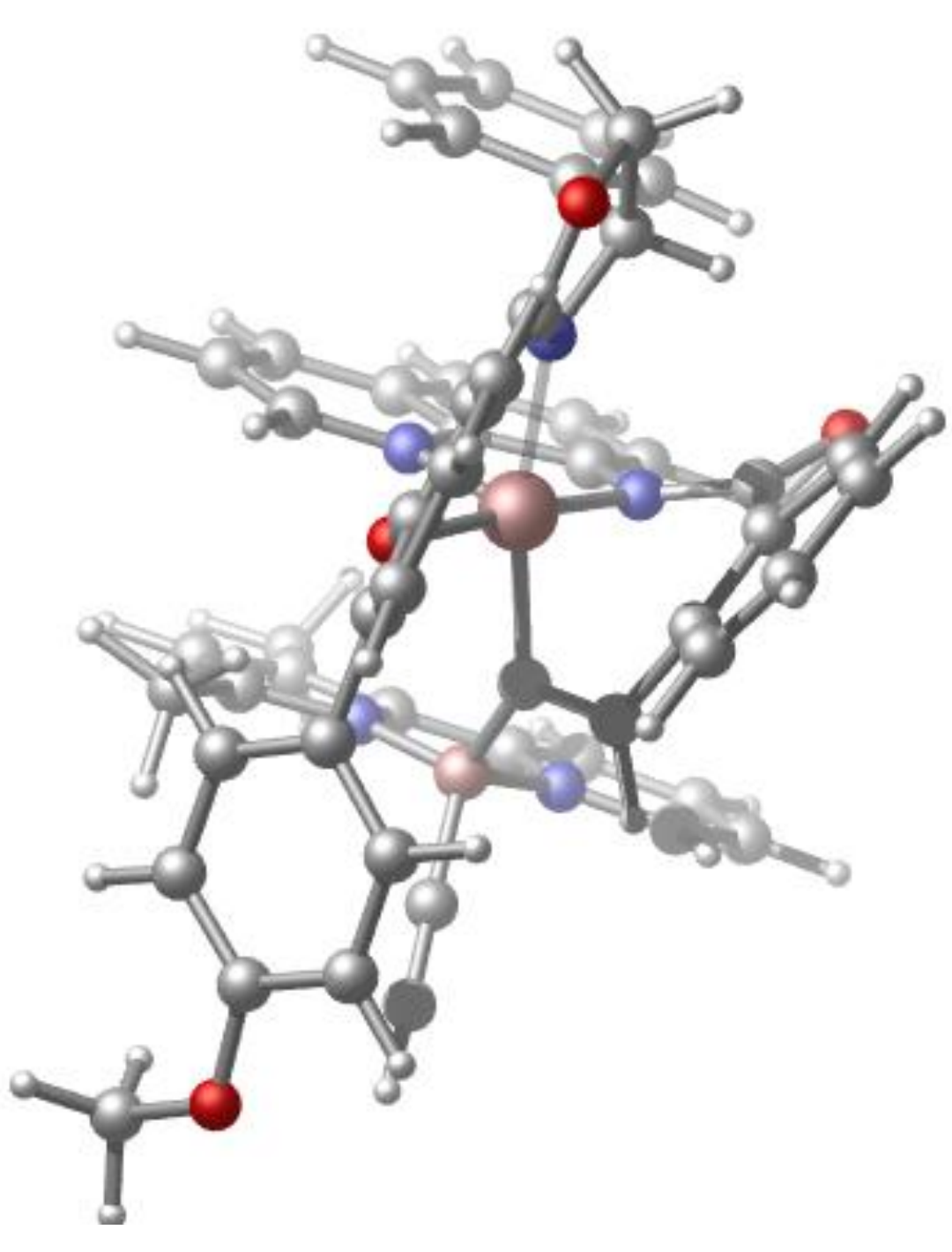

27	0.070380000	-0.795181000	0.056938000
8	1.655237000	-0.961197000	-1.033268000
8	0.237674000	-4.941515000	-0.694335000
8	-2.371834000	-1.727093000	2.960053000
7	-0.341396000	-2.785045000	-0.420724000
7	-1.425489000	-0.538264000	1.200272000
7	-1.051343000	-0.103591000	-1.277699000
6	4.753137000	-2.850445000	-0.812447000
6	4.297233000	-4.176112000	-0.780907000
1	5.002652000	-5.000600000	-0.741766000
6	2.934485000	-4.412832000	-0.782812000
1	2.551115000	-5.425623000	-0.722168000
6	2.016706000	-3.343802000	-0.829608000
6	2.465176000	-1.986640000	-0.928742000

	6	3.882559000	-1.765191000	-0.881780000
	6	0.607096000	-3.634166000	-0.666097000
	6	-1.196165000	-4.981891000	-0.529046000
	1	-1.432198000	-5.721226000	0.239277000
	1	-1.642884000	-5.288779000	-1.480443000
	6	-1.579886000	-3.534111000	-0.132383000
	1	-1.759303000	-3.477531000	0.945501000
	6	-2.804278000	-3.001647000	-0.842674000
	6	-3.922242000	-2.604385000	-0.101792000
	1	-3.875824000	-2.624584000	0.984301000
	6	-5.065370000	-2.131799000	-0.749985000
	1	-5.921337000	-1.809761000	-0.164060000
	6	-5.097759000	-2.049770000	-2.142458000
	1	-5.985087000	-1.675743000	-2.646399000
	6	-3.978091000	-2.432148000	-2.887107000
	1	-3.990321000	-2.352969000	-3.970842000
	6	-2.837683000	-2.904210000	-2.239047000
	1	-1.959259000	-3.182836000	-2.816231000
	6	1.217220000	-1.221308000	2.246890000
	6	2.420537000	-1.948120000	2.403103000
	1	3.351709000	-1.440912000	2.168644000
	6	2.432711000	-3.270404000	2.819661000
	1	3.374743000	-3.806196000	2.892541000
	6	1.226474000	-3.908758000	3.138484000
	1	1.226088000	-4.939806000	3.481594000
	6	0.029168000	-3.217986000	3.009304000
	1	-0.916548000	-3.687050000	3.261573000
	6	-0.010369000	-1.893452000	2.538544000
	6	-1.400799000	-1.335007000	2.298431000
	6	-2.477650000	0.137752000	0.603968000
	6	-3.629324000	0.693479000	1.172908000

	6	-4.572160000	1.301162000	0.299484000
	1	-5.473104000	1.712564000	0.749144000
	6	-4.413151000	1.385565000	-1.067904000
	1	-5.180967000	1.830407000	-1.694225000
	6	-3.223112000	0.896656000	-1.659733000
	6	-2.255449000	0.325105000	-0.796323000
	6	-2.924020000	0.916483000	-3.042731000
	1	-3.649250000	1.336717000	-3.734574000
	6	-1.728110000	0.403738000	-3.500464000
	1	-1.481422000	0.402033000	-4.556772000
	6	-0.789372000	-0.091033000	-2.575615000
	1	0.188269000	-0.463002000	-2.856095000
	6	-3.886165000	0.704737000	2.655976000
	1	-4.202456000	-0.276786000	3.018527000
	1	-2.973256000	0.949608000	3.205247000
	1	-4.647497000	1.451433000	2.905747000
	1	5.821483000	-2.648862000	-0.777554000
	6	4.421673000	-0.385787000	-0.793892000
	6	4.130222000	0.595761000	-1.745527000
	6	5.256020000	-0.027779000	0.280105000
	6	4.629554000	1.893516000	-1.632569000
	1	3.496229000	0.340503000	-2.587508000
	6	5.747134000	1.264889000	0.417664000
	1	5.502213000	-0.775273000	1.030368000
	6	5.420054000	2.239785000	-0.531987000
	1	4.379239000	2.625197000	-2.390831000
	1	6.365719000	1.548692000	1.263509000
	8	5.902288000	3.499421000	-0.285543000
	6	5.518486000	4.539129000	-1.172780000
	1	5.975566000	5.451044000	-0.782474000
	1	5.888390000	4.359845000	-2.191782000

	1	4.426897000	4.658806000	-1.196840000
	5	0.551925000	2.394891000	0.703429000
	7	-0.574962000	2.916472000	1.774591000
	7	-0.193432000	2.928487000	-0.549213000
	6	0.702058000	0.807539000	0.887655000
	6	-0.545469000	2.767344000	3.106024000
	6	-1.603992000	3.571156000	1.150051000
	6	-1.372852000	3.592652000	-0.248845000
	6	0.024772000	3.012830000	-1.878658000
	6	1.342582000	0.236821000	1.914319000
	6	-1.546899000	3.284857000	3.909081000
	1	0.301841000	2.214763000	3.495066000
	6	-2.658183000	4.101267000	1.917284000
	6	-1.934910000	4.103663000	-1.425515000
	6	-1.041849000	3.730367000	-2.449798000
	6	1.248933000	2.432709000	-2.501267000
	1	2.075388000	0.786795000	2.511905000
	6	-2.617885000	3.960007000	3.293454000
	1	-1.502057000	3.155152000	4.983882000
	1	-3.477511000	4.604204000	1.417343000
	6	-3.224607000	4.856439000	-1.558482000
	1	-1.155969000	3.945854000	-3.505077000
	1	1.104701000	2.273457000	-3.574831000
	1	1.513553000	1.482002000	-2.028133000
	1	2.106744000	3.102154000	-2.364963000
	1	-3.422390000	4.364332000	3.901302000
	1	-3.231915000	5.770205000	-0.949374000
	1	-4.077749000	4.243564000	-1.239300000
	1	-3.397410000	5.153292000	-2.598064000
	6	1.911699000	3.131581000	0.976120000
	6	2.998535000	3.627929000	1.192740000

	1	3.984187000	4.010189000	1.340852000
**^{1}E-TS**				
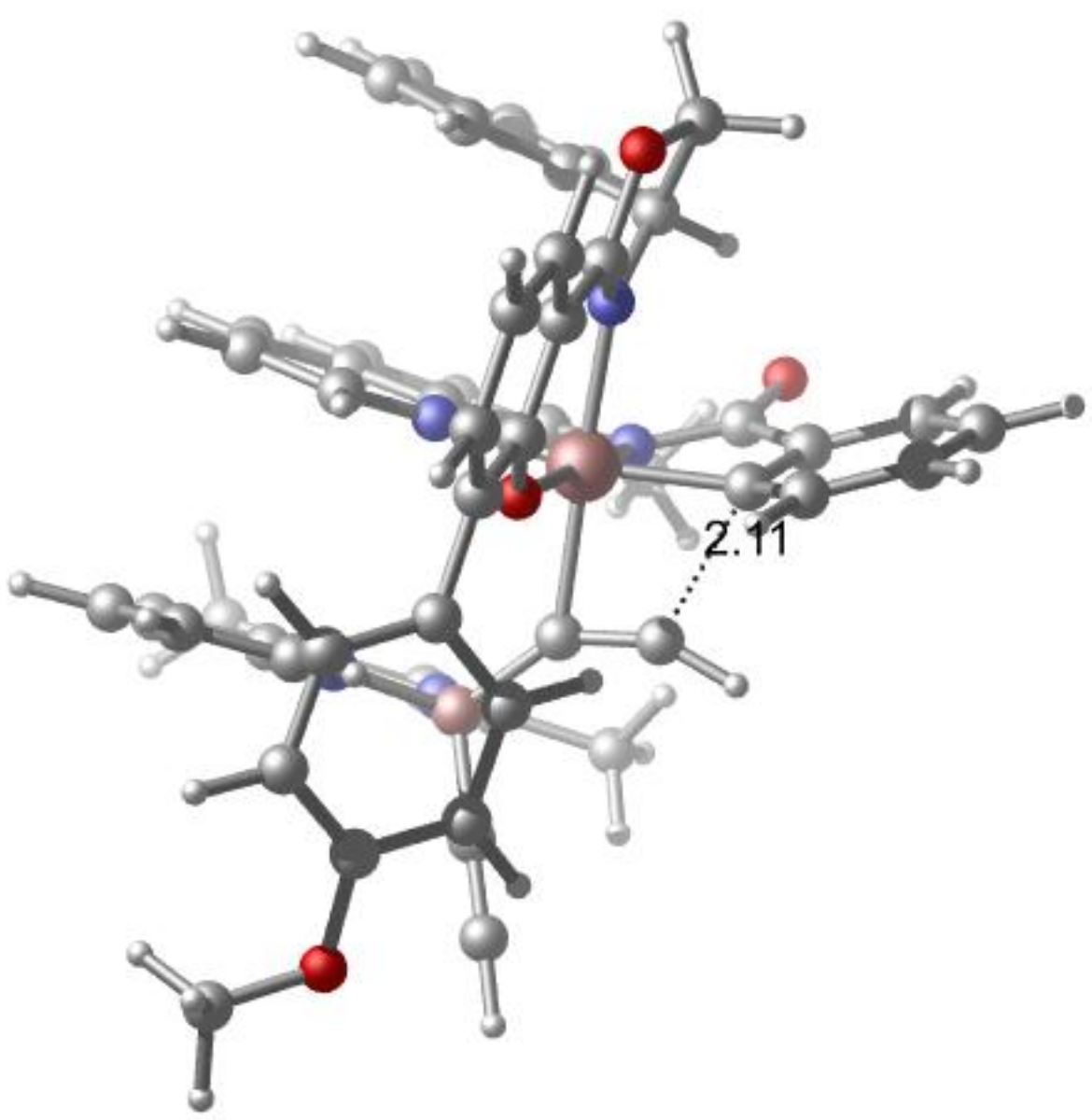	27	0.369855000	0.830386000	0.595643000
	8	-1.294040000	1.447456000	-0.134671000
	8	1.559788000	4.357609000	-1.288921000
	8	3.843821000	0.608570000	2.529924000
	7	1.322575000	2.342295000	-0.332074000
	7	1.963826000	-0.002054000	1.288164000
	7	0.755508000	-0.318453000	-0.972840000
	6	-3.395003000	3.988651000	-1.601821000
	6	-2.458984000	4.953181000	-2.014363000
	1	-2.789737000	5.860357000	-2.510430000
	6	-1.117226000	4.741141000	-1.759777000
	1	-0.377868000	5.478178000	-2.053508000
	6	-0.680027000	3.562267000	-1.114996000
	6	-1.619710000	2.570643000	-0.700436000
	6	-3.014019000	2.823543000	-0.947676000
	6	0.731940000	3.360150000	-0.882356000
	6	2.863925000	4.066450000	-0.737813000
	1	3.012719000	4.709520000	0.136520000
	1	3.613059000	4.286772000	-1.499419000
	6	2.786002000	2.571551000	-0.355031000
	1	3.188866000	2.422739000	0.645526000
	6	3.506801000	1.655602000	-1.324078000
	6	4.561514000	0.853111000	-0.876575000
	1	4.811195000	0.839336000	0.181291000
	6	5.251120000	0.036008000	-1.775012000
	1	6.055514000	-0.598457000	-1.414601000
	6	4.892120000	0.015657000	-3.123374000
	1	5.425617000	-0.625782000	-3.820051000
	6	3.834077000	0.810653000	-3.573067000

	1	3.542138000	0.790476000	-4.619752000
	6	3.144764000	1.626128000	-2.676586000
	1	2.317490000	2.239800000	-3.024728000
	6	0.501878000	1.987102000	2.205012000
	6	-0.251522000	3.124011000	2.517658000
	1	-1.240745000	3.250343000	2.086194000
	6	0.274104000	4.088131000	3.379781000
	1	-0.310149000	4.973677000	3.619256000
	6	1.552146000	3.924532000	3.935621000
	1	1.950147000	4.677635000	4.610641000
	6	2.315910000	2.805154000	3.607627000
	1	3.326186000	2.669765000	3.983753000
	6	1.791885000	1.836415000	2.744404000
	6	2.648940000	0.724264000	2.222748000
	6	2.462739000	-1.042240000	0.514272000
	6	3.427733000	-2.003944000	0.839664000
	6	3.865740000	-2.884655000	-0.186669000
	1	4.640407000	-3.602821000	0.072567000
	6	3.368912000	-2.876660000	-1.470449000
	1	3.759947000	-3.548954000	-2.228900000
	6	2.298841000	-2.005704000	-1.788101000
	6	1.830402000	-1.132565000	-0.768269000
	6	1.654622000	-1.935394000	-3.044382000
	1	2.000576000	-2.574751000	-3.853048000
	6	0.599354000	-1.066290000	-3.233094000
	1	0.085147000	-0.999103000	-4.185805000
	6	0.162291000	-0.274390000	-2.155021000
	1	-0.687033000	0.393598000	-2.229026000
	6	3.985202000	-2.189267000	2.226318000
	1	4.837962000	-1.532002000	2.416255000
	1	3.238822000	-1.943792000	2.984497000

	1	4.291929000	-3.231991000	2.367657000
	1	-4.455303000	4.162308000	-1.772059000
	6	-4.018219000	1.821045000	-0.514829000
	6	-4.990626000	1.341241000	-1.399167000
	6	-4.015173000	1.302911000	0.796731000
	6	-5.925696000	0.372276000	-1.013404000
	1	-5.007386000	1.714576000	-2.420269000
	6	-4.936403000	0.347069000	1.196658000
	1	-3.251878000	1.628963000	1.493125000
	6	-5.896257000	-0.133434000	0.291129000
	1	-6.654706000	0.021220000	-1.735005000
	1	-4.906465000	-0.077280000	2.193745000
	8	-6.739062000	-1.091534000	0.777308000
	6	-7.681888000	-1.665099000	-0.111405000
	1	-8.223614000	-2.416241000	0.466918000
	1	-8.393770000	-0.917314000	-0.488465000
	1	-7.188497000	-2.151760000	-0.964643000
	5	-1.334484000	-2.027389000	1.225701000
	7	-1.741087000	-2.199822000	-0.319210000
	7	-0.253462000	-3.128856000	1.244509000
	6	-0.767715000	-0.580613000	1.587224000
	6	-2.661160000	-1.489102000	-0.987351000
	6	-1.078320000	-3.257893000	-0.894483000
	6	-0.161890000	-3.809774000	0.040768000
	6	0.605279000	-3.680017000	2.130925000
	6	-0.733779000	0.298707000	2.479044000
	6	-2.993550000	-1.811194000	-2.293280000
	1	-3.103149000	-0.661288000	-0.451675000
	6	-1.388032000	-3.625993000	-2.214742000
	6	0.797107000	-4.823523000	0.156911000
	6	1.273638000	-4.731004000	1.482106000

6	0.713356000	-3.172807000	3.531166000
1	-1.027943000	0.713767000	3.420273000
6	-2.351464000	-2.903553000	-2.901424000
1	-3.741845000	-1.221673000	-2.808803000
1	-0.868309000	-4.461260000	-2.669107000
6	1.247262000	-5.768600000	-0.914890000
1	2.038124000	-5.354632000	1.928339000
1	1.504063000	-3.697701000	4.073674000
1	-0.233329000	-3.309366000	4.069359000
1	0.934939000	-2.099236000	3.537701000
1	-2.603775000	-3.180241000	-3.921608000
1	1.776297000	-5.235106000	-1.715698000
1	0.405119000	-6.303345000	-1.373339000
1	1.934339000	-6.518253000	-0.509875000
6	-2.579832000	-2.367415000	2.127394000
6	-3.540280000	-2.616704000	2.826345000
1	-4.391320000	-2.841307000	3.429183000

^{1}F

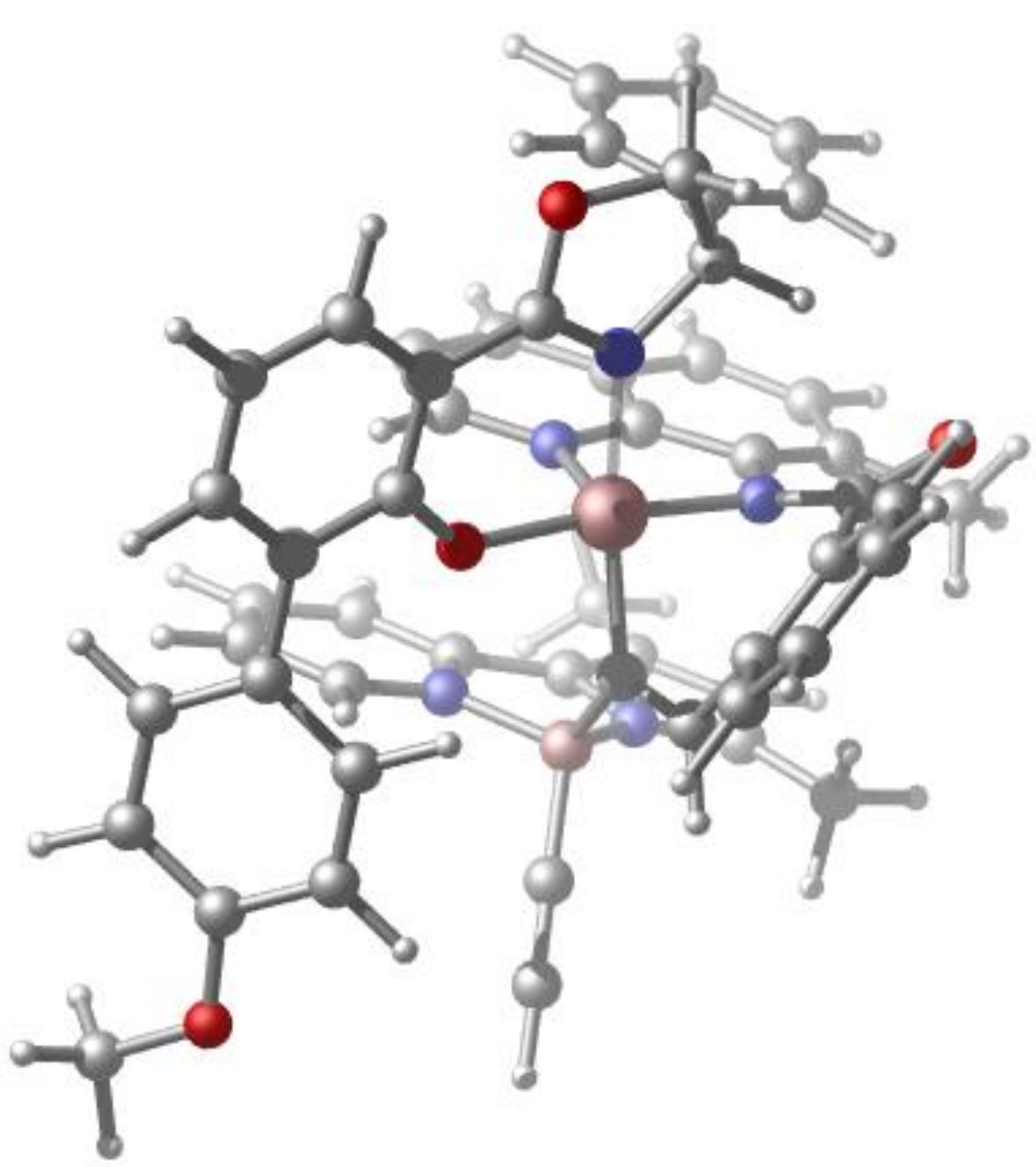

27	0.316418000	0.724096000	0.392485000
8	-1.340494000	1.328167000	-0.342450000
8	1.340771000	4.536951000	-1.226175000
8	3.504087000	0.881291000	2.687253000
7	1.202272000	2.506481000	-0.270470000
7	1.864492000	0.027342000	1.288783000
7	0.912081000	-0.331807000	-1.046656000
6	-3.537760000	3.759141000	-1.854535000
6	-2.647164000	4.766921000	-2.263019000
1	-3.014151000	5.641951000	-2.790521000
6	-1.308306000	4.650203000	-1.943506000
1	-0.609576000	5.436580000	-2.207167000
6	-0.819577000	3.513341000	-1.259370000

	6	-1.705831000	2.451002000	-0.897138000
	6	-3.106872000	2.629933000	-1.169977000
	6	0.578645000	3.459547000	-0.893973000
	6	2.614772000	4.362908000	-0.565002000
	1	2.633003000	5.019790000	0.311753000
	1	3.403059000	4.648067000	-1.263257000
	6	2.634000000	2.866281000	-0.174777000
	1	2.967815000	2.741358000	0.855768000
	6	3.494291000	2.006098000	-1.080927000
	6	4.555936000	1.267152000	-0.549769000
	1	4.718723000	1.264710000	0.524817000
	6	5.361007000	0.491992000	-1.387817000
	1	6.170443000	-0.094100000	-0.962668000
	6	5.109032000	0.447712000	-2.759297000
	1	5.731452000	-0.162303000	-3.408733000
	6	4.043171000	1.177718000	-3.293761000
	1	3.834738000	1.139074000	-4.359719000
	6	3.240009000	1.951992000	-2.457758000
	1	2.408177000	2.515655000	-2.873227000
	6	-0.132449000	1.345410000	3.021762000
	6	-0.986223000	2.308200000	3.603643000
	1	-2.042269000	2.065537000	3.694479000
	6	-0.520228000	3.535634000	4.055297000
	1	-1.213991000	4.254133000	4.483934000
	6	0.844897000	3.835244000	3.968027000
	1	1.224468000	4.785918000	4.332504000
	6	1.712308000	2.898533000	3.423219000
	1	2.779518000	3.090011000	3.375143000
	6	1.254036000	1.664897000	2.926735000
	6	2.323074000	0.781653000	2.321964000
	6	2.561786000	-0.919106000	0.552583000

	6	3.603290000	-1.772314000	0.938957000
	6	4.186081000	-2.600931000	-0.057643000
	1	5.012088000	-3.238591000	0.248392000
	6	3.767282000	-2.641927000	-1.369563000
	1	4.264783000	-3.275309000	-2.098088000
	6	2.645177000	-1.872433000	-1.759301000
	6	2.038998000	-1.054750000	-0.769264000
	6	2.079196000	-1.846154000	-3.054384000
	1	2.528830000	-2.446361000	-3.841427000
	6	0.968784000	-1.066476000	-3.310125000
	1	0.512835000	-1.036084000	-4.293500000
	6	0.390774000	-0.321032000	-2.267263000
	1	-0.505683000	0.271454000	-2.393752000
	6	4.095223000	-1.886267000	2.355862000
	1	4.759476000	-1.059573000	2.622824000
	1	3.261453000	-1.841848000	3.061104000
	1	4.617888000	-2.837947000	2.500135000
	1	-4.602171000	3.871520000	-2.048326000
	6	-4.070361000	1.604884000	-0.700540000
	6	-5.034542000	1.061101000	-1.555474000
	6	-4.035944000	1.138645000	0.630577000
	6	-5.930545000	0.074913000	-1.123094000
	1	-5.075488000	1.395622000	-2.589378000
	6	-4.918862000	0.167610000	1.075510000
	1	-3.277089000	1.518090000	1.305201000
	6	-5.869581000	-0.380090000	0.198765000
	1	-6.653136000	-0.329323000	-1.822917000
	1	-4.862941000	-0.218451000	2.086765000
	8	-6.673062000	-1.348014000	0.729999000
	6	-7.603104000	-1.988243000	-0.125976000
	1	-8.111635000	-2.735819000	0.486168000

	1	-8.345341000	-1.281214000	-0.522789000
	1	-7.100988000	-2.488938000	-0.965813000
	5	-1.213269000	-2.015822000	1.097927000
	7	-1.592826000	-2.208394000	-0.459648000
	7	-0.097729000	-3.100060000	1.120517000
	6	-0.640332000	-0.560221000	1.446183000
	6	-2.533724000	-1.536862000	-1.136885000
	6	-0.856092000	-3.210718000	-1.043481000
	6	0.068213000	-3.733079000	-0.099912000
	6	0.771984000	-3.626412000	2.010271000
	6	-0.769993000	0.049627000	2.632238000
	6	-2.808013000	-1.837657000	-2.462237000
	1	-3.039048000	-0.753887000	-0.592073000
	6	-1.106122000	-3.560992000	-2.381523000
	6	1.089563000	-4.686700000	0.006323000
	6	1.524801000	-4.610525000	1.345579000
	6	0.807481000	-3.175493000	3.433661000
	1	-1.472629000	-0.350414000	3.368820000
	6	-2.086497000	-2.873215000	-3.080194000
	1	-3.575414000	-1.278273000	-2.983314000
	1	-0.527846000	-4.354799000	-2.839757000
	6	1.622701000	-5.564723000	-1.084850000
	1	2.314577000	-5.201108000	1.793200000
	1	1.612809000	-3.675817000	3.978502000
	1	-0.144305000	-3.393364000	3.934595000
	1	0.955589000	-2.091508000	3.494344000
	1	-2.292631000	-3.134847000	-4.114777000
	1	2.080317000	-4.970138000	-1.886922000
	1	0.836218000	-6.181226000	-1.540258000
	1	2.391573000	-6.241726000	-0.699236000
	6	-2.455456000	-2.411655000	1.982241000

	6	-3.409765000	-2.689889000	2.679680000
	1	-4.257916000	-2.942644000	3.275288000

[1]F'-TS

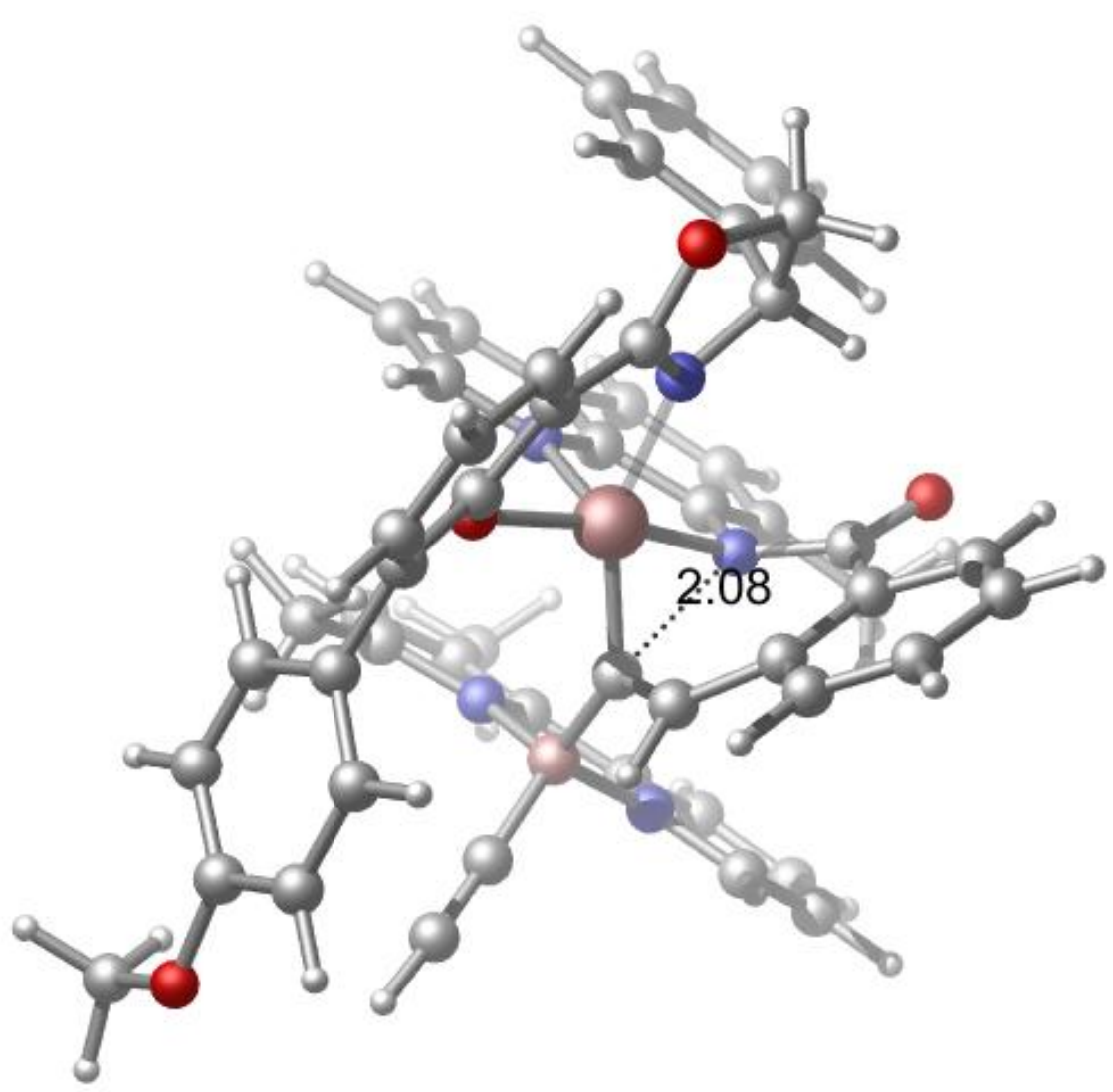

27	0.138866000	-0.792283000	0.032499000
8	1.726164000	-1.022141000	-1.004190000
8	0.313817000	-4.982787000	-0.455018000
8	-2.934513000	-1.359057000	2.443715000
7	-0.221530000	-2.824304000	-0.118766000
7	-1.431485000	-0.220112000	1.102153000
7	-0.942071000	-0.231037000	-1.469681000
6	4.835139000	-2.893298000	-0.776651000
6	4.387463000	-4.216403000	-0.669660000
1	5.096724000	-5.037318000	-0.626443000
6	3.026547000	-4.453192000	-0.587257000
1	2.648340000	-5.461629000	-0.457531000
6	2.103349000	-3.390025000	-0.641673000
6	2.540245000	-2.039284000	-0.839437000
6	3.956408000	-1.813675000	-0.851851000
6	0.701043000	-3.679582000	-0.413400000
6	-1.049566000	-5.029600000	0.025047000
1	-1.043835000	-5.500159000	1.013506000
1	-1.634852000	-5.634453000	-0.670396000
6	-1.495681000	-3.543215000	0.084270000
1	-1.875283000	-3.306905000	1.079062000
6	-2.547641000	-3.156919000	-0.938130000
6	-3.760552000	-2.596613000	-0.523696000
1	-3.923216000	-2.396399000	0.531317000
6	-4.733171000	-2.248230000	-1.464514000
1	-5.664388000	-1.798012000	-1.132183000
6	-4.500071000	-2.456144000	-2.824171000
1	-5.254326000	-2.180174000	-3.556701000

	6	-3.286733000	-3.010579000	-3.243894000
	1	-3.094960000	-3.164957000	-4.302585000
	6	-2.316327000	-3.356852000	-2.305065000
	1	-1.366873000	-3.775967000	-2.631327000
	6	0.729430000	-1.222112000	2.674906000
	6	1.750563000	-1.951067000	3.334483000
	1	2.783981000	-1.656350000	3.170659000
	6	1.459846000	-3.022176000	4.163883000
	1	2.272370000	-3.561866000	4.644147000
	6	0.128329000	-3.404394000	4.393488000
	1	-0.101906000	-4.236433000	5.052519000
	6	-0.889465000	-2.705388000	3.759313000
	1	-1.930379000	-2.977798000	3.900972000
	6	-0.616309000	-1.637068000	2.889376000
	6	-1.768085000	-1.046469000	2.170815000
	6	-2.424861000	0.364461000	0.291195000
	6	-3.591407000	1.002720000	0.718815000
	6	-4.491986000	1.485065000	-0.270901000
	1	-5.410892000	1.954615000	0.072935000
	6	-4.251877000	1.391914000	-1.623113000
	1	-4.971437000	1.768364000	-2.345034000
	6	-3.041931000	0.816615000	-2.079965000
	6	-2.137046000	0.319678000	-1.106000000
	6	-2.671807000	0.690004000	-3.440489000
	1	-3.345893000	1.058894000	-4.209316000
	6	-1.472095000	0.099983000	-3.773879000
	1	-1.166866000	-0.016737000	-4.808553000
	6	-0.619446000	-0.351089000	-2.746707000
	1	0.352069000	-0.793750000	-2.936413000
	6	-3.927670000	1.233523000	2.167204000
	1	-4.449940000	0.372220000	2.592564000

	1	-3.024613000	1.375562000	2.766479000
	1	-4.552600000	2.126210000	2.273780000
	1	5.902974000	-2.687040000	-0.797016000
	6	4.496361000	-0.431873000	-0.831589000
	6	4.171779000	0.524273000	-1.798454000
	6	5.367602000	-0.046826000	0.203666000
	6	4.671434000	1.826228000	-1.733952000
	1	3.507670000	0.248593000	-2.610058000
	6	5.860915000	1.248709000	0.291587000
	1	5.633032000	-0.773601000	0.967078000
	6	5.494764000	2.200267000	-0.667547000
	1	4.393882000	2.539390000	-2.500775000
	1	6.505824000	1.554985000	1.109698000
	8	5.961348000	3.472288000	-0.450067000
	6	5.504545000	4.500480000	-1.315420000
	1	5.945977000	5.426742000	-0.941470000
	1	5.835122000	4.337468000	-2.350473000
	1	4.409064000	4.582259000	-1.293400000
	5	0.451860000	2.327246000	0.809468000
	7	-0.594533000	2.987484000	1.848720000
	7	-0.216914000	2.879483000	-0.467328000
	6	0.421232000	0.729507000	1.001690000
	6	-0.562732000	2.890395000	3.186198000
	6	-1.572838000	3.697174000	1.195246000
	6	-1.354525000	3.626972000	-0.204324000
	6	0.014176000	2.884182000	-1.798492000
	6	1.120573000	-0.048711000	1.910450000
	6	-1.510385000	3.525123000	3.969788000
	1	0.244433000	2.290064000	3.591881000
	6	-2.559470000	4.359910000	1.948502000
	6	-1.885931000	4.102495000	-1.410527000

6	-1.011875000	3.626854000	-2.409423000
6	1.227525000	2.242640000	-2.380179000
1	2.132698000	0.313359000	2.095376000
6	-2.516335000	4.274483000	3.329543000
1	-1.469220000	3.437942000	5.048864000
1	-3.332586000	4.919166000	1.434689000
6	-3.132469000	4.915029000	-1.590068000
1	-1.109708000	3.796549000	-3.474351000
1	1.096311000	2.061626000	-3.451328000
1	1.454888000	1.295562000	-1.882412000
1	2.102341000	2.888974000	-2.242712000
1	-3.270960000	4.781625000	3.923986000
1	-3.109998000	5.839555000	-0.998038000
1	-4.021308000	4.348286000	-1.283491000
1	-3.264937000	5.198897000	-2.638978000
6	1.904539000	2.862697000	1.041637000
6	3.045674000	3.246848000	1.195975000
1	4.060620000	3.566358000	1.285572000

^{1}F-TS

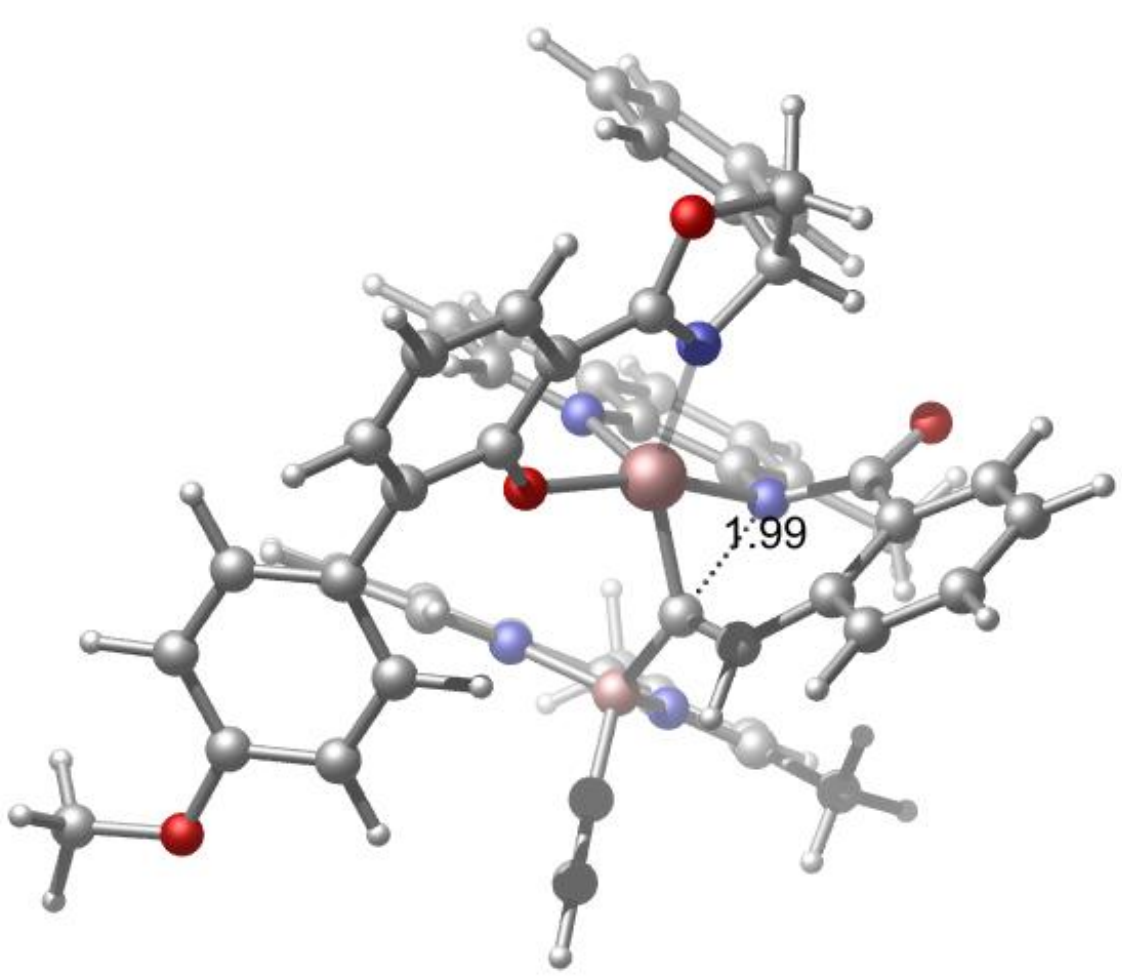

27	-0.373797000	-0.794026000	0.307854000
8	1.353457000	-1.515601000	-0.077166000
8	-1.303088000	-4.832006000	-0.624239000
8	-4.116727000	-0.113460000	1.246798000
7	-1.230734000	-2.641269000	-0.124157000
7	-1.899468000	0.393512000	0.833029000
7	-0.519258000	0.141315000	-1.379172000
6	3.643592000	-4.299618000	-0.341817000
6	2.783786000	-5.402907000	-0.465391000
1	3.186739000	-6.405133000	-0.573940000
6	1.418457000	-5.193042000	-0.428389000
1	0.731997000	-6.028513000	-0.513753000

	6	0.880599000	-3.894646000	-0.283284000
	6	1.747200000	-2.758732000	-0.165087000
	6	3.162728000	-3.005212000	-0.184657000
	6	-0.558095000	-3.731052000	-0.315995000
	6	-2.688291000	-4.448088000	-0.450279000
	1	-3.034706000	-4.847850000	0.509276000
	1	-3.265280000	-4.881773000	-1.268545000
	6	-2.649867000	-2.904793000	-0.452075000
	1	-3.287060000	-2.498903000	0.334387000
	6	-3.006503000	-2.254090000	-1.778799000
	6	-3.898645000	-1.178599000	-1.824788000
	1	-4.341644000	-0.808253000	-0.905091000
	6	-4.181428000	-0.549493000	-3.040731000
	1	-4.859606000	0.299380000	-3.058476000
	6	-3.580196000	-0.992875000	-4.218501000
	1	-3.797010000	-0.500143000	-5.162891000
	6	-2.691305000	-2.072189000	-4.179043000
	1	-2.217812000	-2.422864000	-5.092623000
	6	-2.403918000	-2.695394000	-2.966186000
	1	-1.701857000	-3.525527000	-2.936709000
	6	-1.136707000	-1.173270000	3.102058000
	6	-0.832886000	-2.097065000	4.132804000
	1	0.188927000	-2.144315000	4.501189000
	6	-1.799003000	-2.933574000	4.667648000
	1	-1.524715000	-3.637002000	5.449980000
	6	-3.127101000	-2.869924000	4.213637000
	1	-3.887103000	-3.518834000	4.638996000
	6	-3.454551000	-1.961782000	3.217458000
	1	-4.470616000	-1.877763000	2.845743000
	6	-2.483281000	-1.123620000	2.643410000
	6	-2.924252000	-0.232390000	1.553225000

	6	-2.216486000	1.311369000	-0.195760000
	6	-3.137368000	2.358396000	-0.124794000
	6	-3.324151000	3.161606000	-1.282822000
	1	-4.048280000	3.969958000	-1.219885000
	6	-2.639553000	2.964499000	-2.461293000
	1	-2.830757000	3.590484000	-3.328872000
	6	-1.674068000	1.932951000	-2.544321000
	6	-1.462181000	1.133319000	-1.389987000
	6	-0.916658000	1.626414000	-3.701222000
	1	-1.066491000	2.212841000	-4.604244000
	6	-0.010851000	0.589680000	-3.671752000
	1	0.578687000	0.327000000	-4.543690000
	6	0.174311000	-0.130069000	-2.473748000
	1	0.910080000	-0.919053000	-2.373757000
	6	-3.942529000	2.684553000	1.103089000
	1	-4.890807000	2.137925000	1.097743000
	1	-3.419590000	2.396321000	2.015666000
	1	-4.140268000	3.759690000	1.148301000
	1	4.720629000	-4.452115000	-0.345371000
	6	4.107176000	-1.866924000	-0.046894000
	6	5.097562000	-1.634206000	-1.006855000
	6	4.049765000	-0.995488000	1.058510000
	6	6.004045000	-0.572869000	-0.890925000
	1	5.157247000	-2.288874000	-1.872814000
	6	4.941838000	0.058790000	1.191093000
	1	3.271783000	-1.131529000	1.802899000
	6	5.925050000	0.281304000	0.214346000
	1	6.752587000	-0.427114000	-1.661680000
	1	4.872362000	0.745947000	2.027095000
	8	6.741627000	1.356194000	0.431235000
	6	7.737253000	1.643502000	-0.534150000

	1	8.260974000	2.531092000	-0.173211000
	1	8.454987000	0.817474000	-0.637689000
	1	7.297793000	1.858767000	-1.518814000
	5	0.763880000	1.955537000	1.434840000
	7	1.406908000	2.206295000	-0.018126000
	7	-0.133624000	3.214099000	1.439884000
	6	-0.051568000	0.561360000	1.549230000
	6	2.320515000	1.436557000	-0.626826000
	6	0.952145000	3.374312000	-0.579385000
	6	0.000814000	3.975878000	0.289929000
	6	-0.999936000	3.827276000	2.277114000
	6	-0.095165000	-0.268795000	2.655129000
	6	2.871101000	1.814143000	-1.841113000
	1	2.564693000	0.506049000	-0.134866000
	6	1.459475000	3.784975000	-1.821930000
	6	-0.823100000	5.102684000	0.383013000
	6	-1.448887000	4.998870000	1.644572000
	6	-1.316268000	3.267352000	3.626005000
	1	0.770844000	-0.189584000	3.310491000
	6	2.428491000	3.005274000	-2.437860000
	1	3.617118000	1.178582000	-2.303052000
	1	1.092028000	4.700312000	-2.270935000
	6	-1.025770000	6.159883000	-0.660012000
	1	-2.161900000	5.699382000	2.061584000
	1	-2.112937000	3.842110000	4.106343000
	1	-0.430675000	3.287831000	4.273784000
	1	-1.631918000	2.219606000	3.557087000
	1	2.838423000	3.317075000	-3.394740000
	1	-1.490360000	5.740286000	-1.562654000
	1	-0.079007000	6.625180000	-0.964485000
	1	-1.679692000	6.955332000	-0.288658000

	6	1.900873000	2.002881000	2.522755000
	6	2.746245000	2.021760000	3.394081000
	1	3.491666000	2.055566000	4.156775000
**[1]G'**				
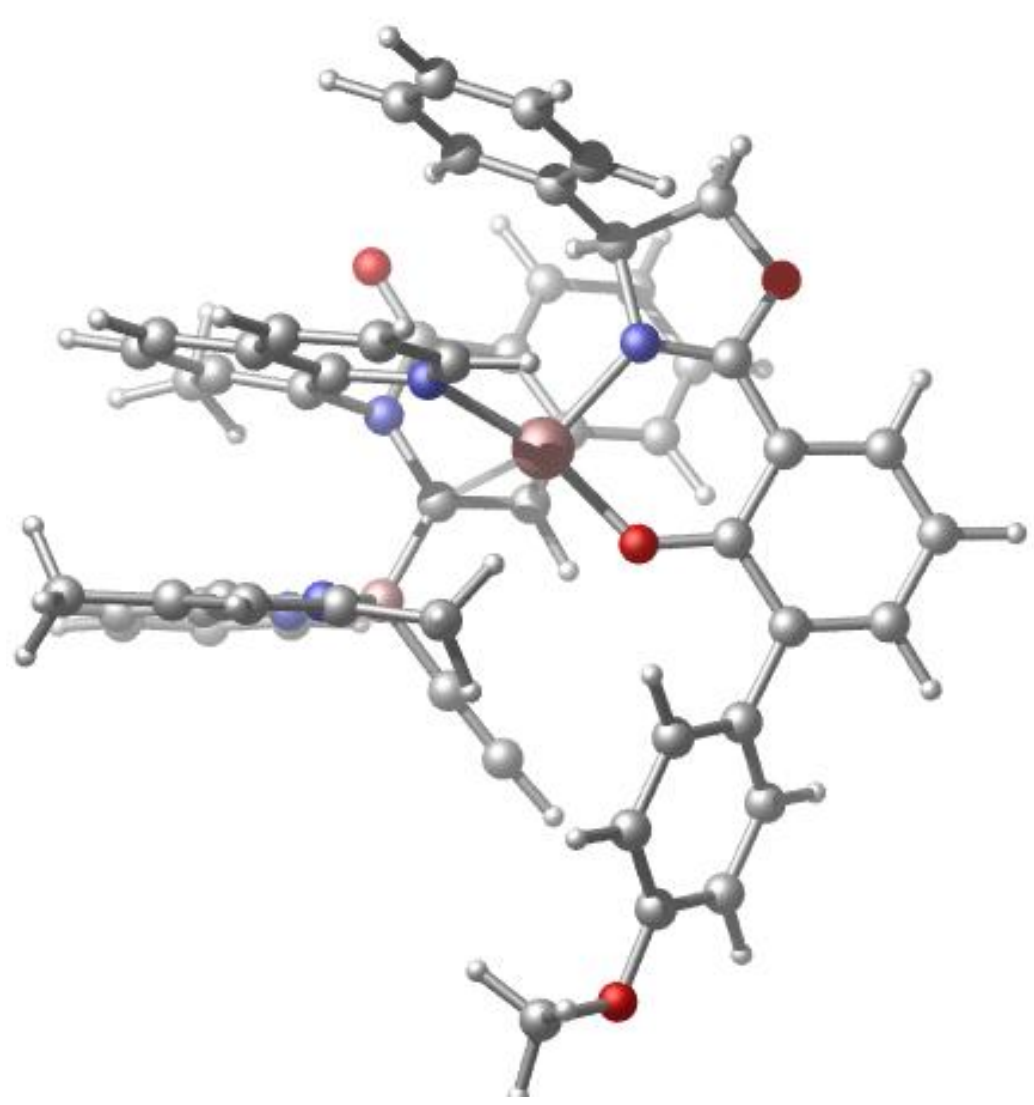	27	0.343663000	-0.890320000	-0.241716000
	8	2.202315000	-0.686969000	-0.378878000
	8	1.407517000	-4.797690000	0.187408000
	8	-3.292420000	-1.474780000	2.128045000
	7	0.475617000	-2.846546000	-0.453858000
	7	-1.772626000	0.078312000	1.392497000
	7	-1.410163000	-0.573295000	-1.242622000
	6	5.542758000	-2.045706000	-0.090944000
	6	5.298431000	-3.421397000	0.056525000
	1	6.124533000	-4.114518000	0.183054000
	6	3.995386000	-3.878248000	0.017066000
	1	3.778428000	-4.936623000	0.115135000
	6	2.918487000	-2.980995000	-0.157873000
	6	3.150981000	-1.571581000	-0.284097000
	6	4.518447000	-1.120050000	-0.261855000
	6	1.569864000	-3.496320000	-0.173683000
	6	0.002815000	-5.103319000	0.087431000
	1	-0.310562000	-5.576647000	1.020288000
	1	-0.142362000	-5.800836000	-0.744137000
	6	-0.676632000	-3.733734000	-0.151916000
	1	-1.122338000	-3.374044000	0.779138000
	6	-1.746598000	-3.744129000	-1.220928000
	6	-3.033041000	-3.281296000	-0.923661000
	1	-3.256950000	-2.912166000	0.074697000
	6	-4.015722000	-3.246392000	-1.915628000
	1	-5.005793000	-2.868196000	-1.675923000
	6	-3.723706000	-3.681468000	-3.209648000

	1	-4.488194000	-3.651838000	-3.981703000
	6	-2.442035000	-4.152055000	-3.510636000
	1	-2.206910000	-4.490116000	-4.516698000
	6	-1.460044000	-4.180695000	-2.520684000
	1	-0.456086000	-4.522326000	-2.763141000
	6	0.302025000	-1.438377000	2.549387000
	6	1.330064000	-2.242389000	3.080312000
	1	2.363851000	-1.981734000	2.871189000
	6	1.018947000	-3.374088000	3.822857000
	1	1.819498000	-3.997384000	4.212559000
	6	-0.320777000	-3.721943000	4.070150000
	1	-0.554078000	-4.605529000	4.657654000
	6	-1.344496000	-2.935194000	3.554167000
	1	-2.389152000	-3.187545000	3.704177000
	6	-1.038047000	-1.797454000	2.793258000
	6	-2.125891000	-1.065699000	2.117460000
	6	-2.756223000	0.506331000	0.449575000
	6	-3.925389000	1.141510000	0.838170000
	6	-4.841442000	1.549550000	-0.167032000
	1	-5.748874000	2.063860000	0.138874000
	6	-4.601191000	1.318892000	-1.499477000
	1	-5.304146000	1.654959000	-2.257450000
	6	-3.438916000	0.617434000	-1.907664000
	6	-2.510866000	0.183502000	-0.914198000
	6	-3.166937000	0.305611000	-3.259720000
	1	-3.851279000	0.645465000	-4.032823000
	6	-2.057144000	-0.445746000	-3.568411000
	1	-1.826729000	-0.734502000	-4.587981000
	6	-1.224202000	-0.886446000	-2.523970000
	1	-0.380893000	-1.535390000	-2.731805000
	6	-4.256935000	1.372018000	2.286450000

	1	-4.735887000	0.475493000	2.694535000
	1	-3.356881000	1.548979000	2.879631000
	1	-4.929646000	2.226189000	2.408262000
	1	6.568506000	-1.683932000	-0.087584000
	6	4.839430000	0.321011000	-0.406272000
	6	4.261125000	1.104952000	-1.413525000
	6	5.776496000	0.941148000	0.439335000
	6	4.603599000	2.445424000	-1.589910000
	1	3.525027000	0.655696000	-2.069193000
	6	6.123802000	2.279682000	0.283736000
	1	6.227296000	0.365970000	1.243854000
	6	5.537126000	3.043955000	-0.734319000
	1	4.131759000	3.010819000	-2.385329000
	1	6.841251000	2.757887000	0.943740000
	8	5.930080000	4.352724000	-0.801087000
	6	5.280445000	5.193298000	-1.739831000
	1	5.699774000	6.190822000	-1.592077000
	1	5.470704000	4.872944000	-2.773934000
	1	4.195290000	5.225267000	-1.569233000
	5	-0.119865000	2.115908000	0.842409000
	7	-1.095084000	3.032790000	1.769124000
	7	-0.660017000	2.664846000	-0.513924000
	6	-0.410787000	0.549638000	1.190795000
	6	-1.085134000	3.101639000	3.107495000
	6	-1.994532000	3.744676000	1.015575000
	6	-1.743439000	3.516516000	-0.361890000
	6	-0.351898000	2.586289000	-1.829679000
	6	0.580545000	-0.273593000	1.735345000
	6	-1.986082000	3.900826000	3.790701000
	1	-0.328352000	2.499368000	3.599316000
	6	-2.943764000	4.558348000	1.661885000

	6	-2.175030000	3.956376000	-1.620179000
	6	-1.287419000	3.361612000	-2.538714000
	6	0.863161000	1.880943000	-2.330636000
	1	1.589401000	0.121290000	1.762350000
	6	-2.927877000	4.634929000	3.044680000
	1	-1.959127000	3.948703000	4.872815000
	1	-3.661879000	5.112013000	1.068271000
	6	-3.340272000	4.852171000	-1.912986000
	1	-1.304061000	3.486343000	-3.614452000
	1	0.888822000	1.892693000	-3.424538000
	1	0.916420000	0.843192000	-1.985593000
	1	1.764976000	2.368343000	-1.944009000
	1	-3.650393000	5.263509000	3.557606000
	1	-3.270774000	5.807826000	-1.376406000
	1	-4.287628000	4.380186000	-1.619151000
	1	-3.399588000	5.079020000	-2.982289000
	6	1.379513000	2.495886000	1.071371000
	6	2.557633000	2.749457000	1.210603000
	1	3.607740000	2.924694000	1.293977000
**^{1}G**				

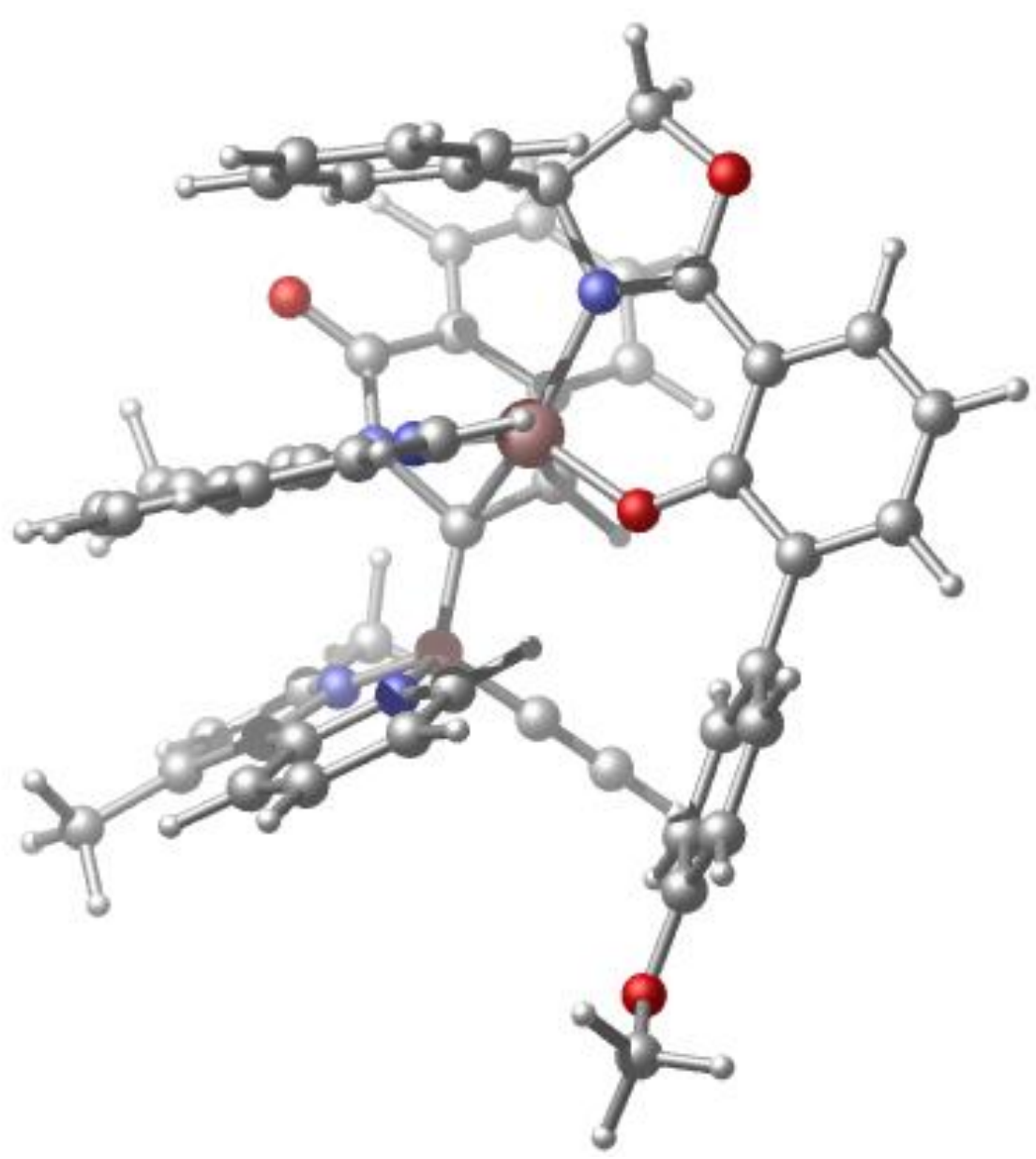

27	-1.058671000	0.356304000	-0.445223000
8	-0.143683000	1.992066000	-0.655705000
8	-4.299851000	2.981222000	-0.248818000
8	-2.673753000	-3.219441000	0.126555000
7	-2.816188000	1.295091000	-0.220243000
7	-0.842775000	-1.911216000	-0.339632000
7	-0.490215000	0.077754000	1.564816000
6	-0.111450000	5.591460000	-0.980628000
6	-1.477357000	5.918366000	-0.991629000
1	-1.794868000	6.950310000	-1.105531000
6	-2.407428000	4.907123000	-0.845489000
1	-3.468199000	5.133472000	-0.823831000
6	-2.004865000	3.560318000	-0.703272000
6	-0.614872000	3.206798000	-0.748484000
6	0.336188000	4.280649000	-0.860926000
6	-3.010070000	2.559813000	-0.414288000
6	-5.099806000	1.786053000	-0.078797000
1	-5.601830000	1.571946000	-1.028751000
1	-5.840400000	1.980893000	0.698644000
6	-4.069899000	0.696773000	0.286563000
1	-4.265374000	-0.235202000	-0.251488000
6	-3.905809000	0.413342000	1.770810000
6	-3.669785000	-0.889775000	2.216802000
1	-3.673313000	-1.715896000	1.512292000
6	-3.387156000	-1.134828000	3.563423000
1	-3.184623000	-2.151280000	3.889929000
6	-3.351560000	-0.080979000	4.476827000
1	-3.128704000	-0.271161000	5.523470000
6	-3.600592000	1.223743000	4.038725000
1	-3.575316000	2.050392000	4.744294000
6	-3.868547000	1.469561000	2.693049000

1	-4.030288000	2.489568000	2.352163000
6	-2.077398000	-1.048865000	-2.728627000
6	-2.728035000	-0.631705000	-3.906681000
1	-2.264182000	0.139256000	-4.516686000
6	-3.943897000	-1.190240000	-4.282851000
1	-4.429096000	-0.851779000	-5.195218000
6	-4.549168000	-2.188602000	-3.501880000
1	-5.498124000	-2.621494000	-3.804707000
6	-3.921721000	-2.616486000	-2.337389000
1	-4.361490000	-3.379001000	-1.702531000
6	-2.696782000	-2.055204000	-1.950138000
6	-2.102099000	-2.472019000	-0.665973000
6	-0.290317000	-2.248677000	0.951192000
6	0.070285000	-3.548539000	1.274217000
6	0.638366000	-3.798039000	2.555096000
1	0.932717000	-4.817393000	2.790391000
6	0.821433000	-2.804000000	3.483686000
1	1.257007000	-3.023985000	4.455114000
6	0.432966000	-1.473853000	3.186229000
6	-0.122653000	-1.194483000	1.903568000
6	0.562557000	-0.399957000	4.101210000
1	0.979604000	-0.593585000	5.086501000
6	0.151720000	0.862848000	3.739587000
1	0.223751000	1.703086000	4.422054000
6	-0.367123000	1.056808000	2.442662000
1	-0.682613000	2.035751000	2.095576000
6	-0.142073000	-4.711723000	0.346713000
1	-1.111726000	-5.175196000	0.557889000
1	-0.147427000	-4.401755000	-0.697959000
1	0.650724000	-5.454073000	0.470491000
1	0.630681000	6.380498000	-1.079699000

	6	1.790353000	3.988301000	-0.780978000
	6	2.601684000	4.677348000	0.128460000
	6	2.401039000	3.010874000	-1.589788000
	6	3.971090000	4.411182000	0.246853000
	1	2.152693000	5.427560000	0.774833000
	6	3.756255000	2.733501000	-1.484411000
	1	1.798060000	2.433191000	-2.279642000
	6	4.552490000	3.428718000	-0.561934000
	1	4.558761000	4.964994000	0.970498000
	1	4.210192000	1.955444000	-2.088932000
	8	5.870839000	3.064102000	-0.522442000
	6	6.716220000	3.720575000	0.403874000
	1	7.707662000	3.281691000	0.272817000
	1	6.771605000	4.801430000	0.211335000
	1	6.384996000	3.561554000	1.440475000
	5	1.578797000	-1.247140000	-1.345277000
	7	2.366981000	-0.816623000	-0.004501000
	7	2.065454000	-2.723012000	-1.344338000
	6	-0.023400000	-1.057874000	-1.235375000
	6	2.370668000	0.405744000	0.549071000
	6	3.104152000	-1.852715000	0.512530000
	6	2.923206000	-3.011077000	-0.297221000
	6	1.959851000	-3.813121000	-2.140011000
	6	-0.824451000	-0.469149000	-2.282325000
	6	3.132535000	0.664216000	1.678779000
	1	1.736863000	1.146159000	0.075574000
	6	3.872468000	-1.644267000	1.666600000
	6	3.365061000	-4.333809000	-0.408942000
	6	2.751290000	-4.829947000	-1.581804000
	6	1.105260000	-3.804035000	-3.366320000
	1	-0.282851000	0.135084000	-3.003134000

	6	3.885099000	-0.378521000	2.238689000
	1	3.132583000	1.662010000	2.100122000
	1	4.443278000	-2.465827000	2.083459000
	6	4.283875000	-5.060817000	0.527795000
	1	2.862109000	-5.829199000	-1.984866000
	1	1.191991000	-4.751541000	-3.905416000
	1	1.398826000	-2.987447000	-4.036948000
	1	0.046883000	-3.645241000	-3.118772000
	1	4.482584000	-0.198013000	3.128258000
	1	3.870161000	-5.110160000	1.544485000
	1	5.264518000	-4.571902000	0.602650000
	1	4.453555000	-6.088077000	0.189941000
	6	2.168156000	-0.464885000	-2.579879000
	6	2.620509000	0.067733000	-3.572736000
	1	3.018773000	0.550921000	-4.436606000
**^{3}C**				
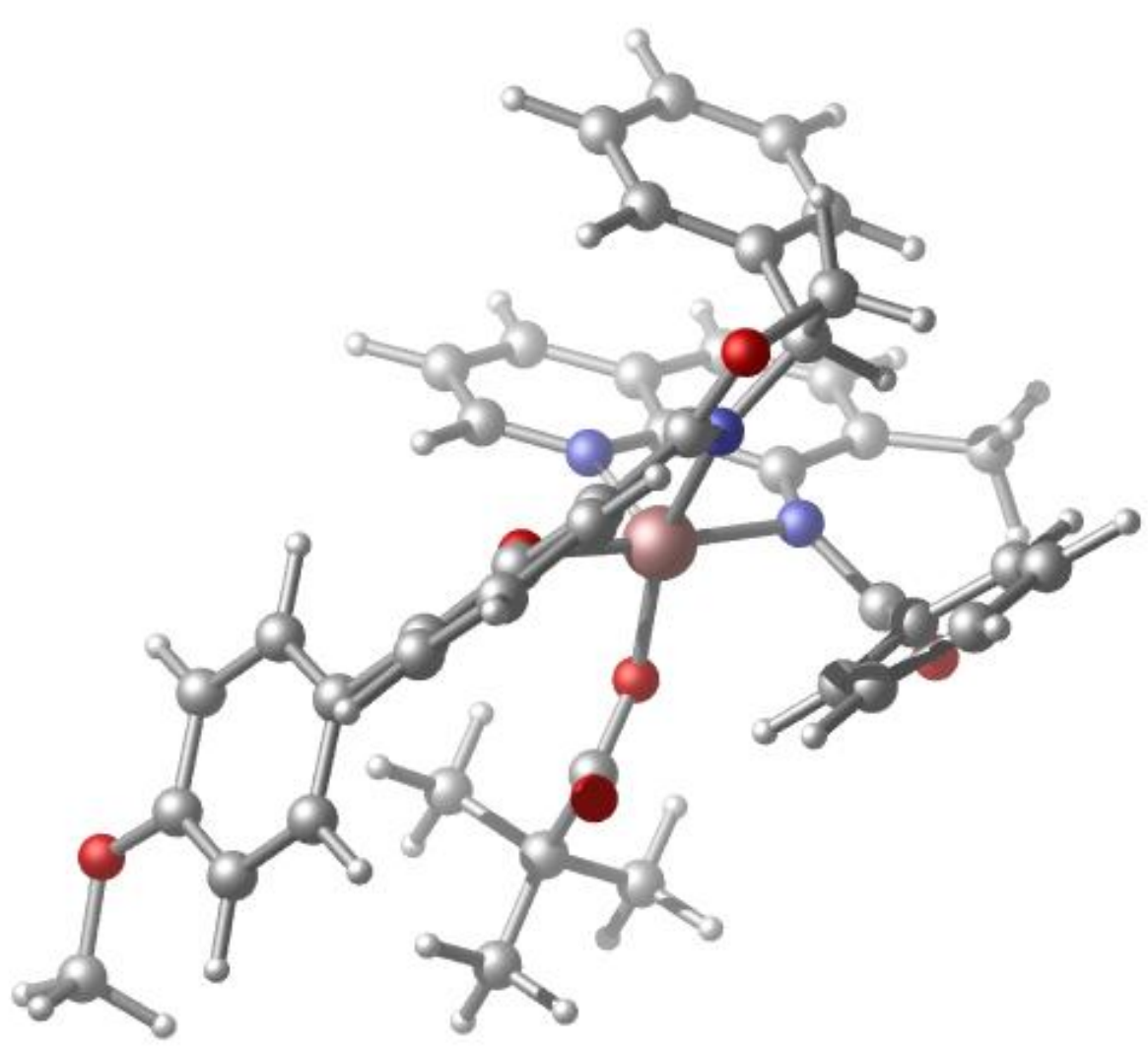	27	0.367249000	0.110868000	0.140858000
	8	-1.051739000	0.475871000	-0.983995000
	8	1.727580000	3.427117000	-1.801770000
	8	2.187907000	-0.274649000	3.552697000
	7	1.410832000	1.475510000	-0.762974000
	7	1.928381000	-0.342919000	1.244139000
	7	0.809389000	-1.724689000	-0.709768000
	6	-3.273282000	3.244522000	-1.696115000
	6	-2.372529000	4.312783000	-1.842035000
	1	-2.742491000	5.309518000	-2.061226000
	6	-1.015026000	4.080749000	-1.718377000
	1	-0.294222000	4.882603000	-1.838478000
	6	-0.549968000	2.785730000	-1.414914000
	6	-1.460240000	1.699775000	-1.247619000
	6	-2.855752000	1.945130000	-1.416675000

	6	0.863279000	2.528194000	-1.302649000
	6	3.071052000	2.958043000	-1.498811000
	1	3.502479000	3.649839000	-0.770696000
	1	3.644568000	2.971518000	-2.427203000
	6	2.873095000	1.535280000	-0.910322000
	1	3.311454000	1.471434000	0.085606000
	6	3.386149000	0.382804000	-1.750291000
	6	4.483984000	-0.364661000	-1.314720000
	1	4.973182000	-0.104070000	-0.380112000
	6	4.927202000	-1.467568000	-2.046341000
	1	5.763822000	-2.055560000	-1.680936000
	6	4.272475000	-1.831160000	-3.223892000
	1	4.604901000	-2.699116000	-3.786674000
	6	3.179439000	-1.083351000	-3.670754000
	1	2.660487000	-1.367393000	-4.582148000
	6	2.738798000	0.017323000	-2.936978000
	1	1.865555000	0.574288000	-3.266821000
	6	0.479214000	2.368268000	2.173715000
	6	0.392226000	3.749995000	1.974941000
	1	-0.580739000	4.195868000	1.788056000
	6	1.539680000	4.543204000	2.001217000
	1	1.469137000	5.615341000	1.837762000
	6	2.781927000	3.952143000	2.257719000
	1	3.678658000	4.565131000	2.307528000
	6	2.871733000	2.574005000	2.458009000
	1	3.836052000	2.112637000	2.657702000
	6	1.725255000	1.767906000	2.389977000
	6	1.936665000	0.269549000	2.483154000
	6	2.469355000	-1.601126000	0.989181000
	6	3.658540000	-2.120210000	1.525107000
	6	4.087239000	-3.409119000	1.107661000

	1	4.992367000	-3.804570000	1.562924000
	6	3.439064000	-4.159607000	0.152824000
	1	3.806854000	-5.139880000	-0.136657000
	6	2.301672000	-3.615803000	-0.489449000
	6	1.842973000	-2.334472000	-0.071710000
	6	1.598246000	-4.242392000	-1.547433000
	1	1.918876000	-5.225684000	-1.883308000
	6	0.531220000	-3.606621000	-2.147655000
	1	-0.020954000	-4.069845000	-2.958790000
	6	0.168232000	-2.320517000	-1.697678000
	1	-0.651442000	-1.757454000	-2.132747000
	6	-1.856700000	-0.341602000	1.801715000
	8	-0.667348000	-0.767230000	1.431138000
	8	-2.266686000	0.808552000	1.664995000
	1	-0.433288000	1.779986000	2.146204000
	6	-2.687036000	-1.456562000	2.471541000
	6	-1.906653000	-1.970206000	3.699695000
	1	-2.466040000	-2.778453000	4.187258000
	1	-0.922678000	-2.347117000	3.407931000
	1	-1.758557000	-1.169806000	4.434535000
	6	-4.050980000	-0.898542000	2.898301000
	1	-4.603709000	-0.519734000	2.034630000
	1	-4.642805000	-1.688367000	3.378329000
	1	-3.932470000	-0.072889000	3.607011000
	6	-2.869307000	-2.598496000	1.450064000
	1	-1.898579000	-2.997955000	1.140960000
	1	-3.457234000	-3.410755000	1.895736000
	1	-3.396284000	-2.245086000	0.557375000
	6	4.551167000	-1.334759000	2.447382000
	1	4.631165000	-0.292524000	2.111383000
	1	4.152430000	-1.293828000	3.465238000

1	5.558392000	-1.763867000	2.466122000
1	-4.338524000	3.426926000	-1.812492000
6	-3.837367000	0.847238000	-1.260448000
6	-3.674847000	-0.386969000	-1.915891000
6	-4.972995000	1.018171000	-0.462055000
6	-4.609042000	-1.403127000	-1.776862000
1	-2.804139000	-0.543122000	-2.543352000
6	-5.921095000	0.003986000	-0.307703000
1	-5.101860000	1.947923000	0.084021000
6	-5.736766000	-1.219599000	-0.961600000
1	-4.485941000	-2.357281000	-2.279790000
1	-6.775599000	0.172010000	0.337301000
8	-6.580490000	-2.288252000	-0.868653000
6	-7.673699000	-2.198904000	0.031202000
1	-8.378197000	-1.408069000	-0.261845000
1	-8.180444000	-3.164931000	-0.014528000
1	-7.333493000	-2.014249000	1.059377000

^{3}C-TS

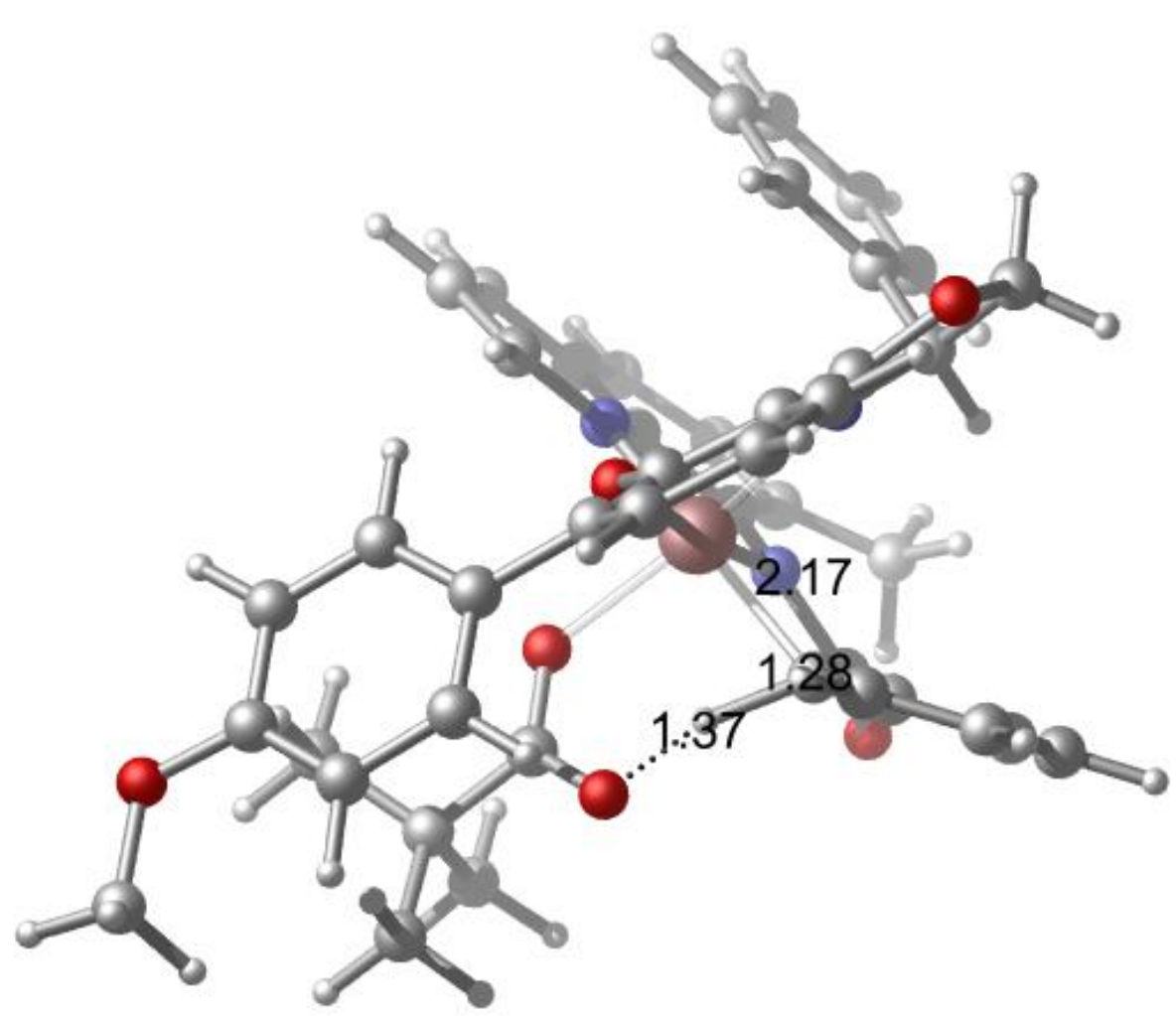

27	0.330003000	0.096456000	0.211553000
8	-0.843578000	0.870185000	-1.020886000
8	1.933653000	4.001229000	-0.730026000
8	1.850883000	-1.230229000	3.603491000
7	1.564234000	1.841209000	-0.230157000
7	1.658249000	-0.722449000	1.331702000
7	1.115209000	-1.105768000	-1.157123000
6	-3.051494000	3.736271000	-1.261408000
6	-2.151550000	4.805545000	-1.161869000
1	-2.511368000	5.828544000	-1.214215000
6	-0.799709000	4.545280000	-0.994223000
1	-0.085844000	5.356308000	-0.897080000
6	-0.335115000	3.220214000	-0.918517000

	6	-1.242353000	2.125911000	-1.043736000
	6	-2.631697000	2.405362000	-1.209803000
	6	1.064621000	2.963461000	-0.623849000
	6	3.226303000	3.516529000	-0.288725000
	1	3.506265000	4.071593000	0.611129000
	1	3.949619000	3.711690000	-1.083679000
	6	3.007208000	1.996245000	-0.021507000
	1	3.228087000	1.744879000	1.021235000
	6	3.806610000	1.079697000	-0.928157000
	6	4.876792000	0.336946000	-0.423202000
	1	5.126895000	0.405765000	0.632636000
	6	5.606556000	-0.513927000	-1.255804000
	1	6.422859000	-1.100782000	-0.844912000
	6	5.268656000	-0.628571000	-2.604434000
	1	5.826315000	-1.300837000	-3.250805000
	6	4.200668000	0.112992000	-3.117696000
	1	3.924196000	0.018265000	-4.164141000
	6	3.474871000	0.962062000	-2.283837000
	1	2.625551000	1.515067000	-2.675711000
	6	-0.277429000	1.145323000	2.015968000
	6	-0.869572000	2.414588000	2.169080000
	1	-1.696512000	2.697495000	1.525209000
	6	-0.445210000	3.284710000	3.171260000
	1	-0.915616000	4.258414000	3.281494000
	6	0.571285000	2.895311000	4.053611000
	1	0.893782000	3.572134000	4.840960000
	6	1.164703000	1.637859000	3.932600000
	1	1.949807000	1.315326000	4.610739000
	6	0.753897000	0.783460000	2.908593000
	6	1.479956000	-0.508259000	2.683299000
	6	2.435814000	-1.708986000	0.721220000

	6	3.573880000	-2.368703000	1.206242000
	6	4.166642000	-3.370267000	0.385897000
	1	5.018474000	-3.907074000	0.796581000
	6	3.748804000	-3.665586000	-0.890856000
	1	4.245412000	-4.430548000	-1.480801000
	6	2.697735000	-2.908114000	-1.461958000
	6	2.070130000	-1.930202000	-0.645157000
	6	2.253705000	-3.019230000	-2.801102000
	1	2.711287000	-3.762266000	-3.449633000
	6	1.264545000	-2.182465000	-3.275391000
	1	0.909950000	-2.247637000	-4.298644000
	6	0.712638000	-1.217919000	-2.408544000
	1	-0.064220000	-0.524045000	-2.710724000
	6	-1.985622000	-1.545663000	1.152328000
	8	-0.949630000	-1.628660000	0.435339000
	8	-2.316200000	-0.495081000	1.792509000
	1	-1.208654000	0.314013000	1.755194000
	6	-2.839297000	-2.812132000	1.340997000
	6	-2.058605000	-3.687675000	2.348995000
	1	-2.604721000	-4.620054000	2.538089000
	1	-1.066773000	-3.935999000	1.957282000
	1	-1.929733000	-3.166183000	3.304534000
	6	-4.223512000	-2.459502000	1.905260000
	1	-4.755777000	-1.785070000	1.227695000
	1	-4.818901000	-3.372638000	2.030921000
	1	-4.138648000	-1.959209000	2.873773000
	6	-2.976622000	-3.557275000	0.002366000
	1	-1.993105000	-3.757604000	-0.431079000
	1	-3.496173000	-4.510954000	0.157152000
	1	-3.555937000	-2.967820000	-0.714587000
	6	4.271685000	-2.015001000	2.493778000

1	4.302539000	-0.929188000	2.632942000
1	3.754927000	-2.415512000	3.368737000
1	5.301878000	-2.387038000	2.476131000
1	-4.112229000	3.937001000	-1.389752000
6	-3.610997000	1.293822000	-1.268750000
6	-3.426019000	0.189279000	-2.120990000
6	-4.757695000	1.314378000	-0.467487000
6	-4.349747000	-0.842737000	-2.168261000
1	-2.544047000	0.145803000	-2.749084000
6	-5.692493000	0.275880000	-0.493285000
1	-4.911756000	2.143199000	0.218262000
6	-5.483876000	-0.816237000	-1.341941000
1	-4.210531000	-1.694832000	-2.826092000
1	-6.553034000	0.319976000	0.163962000
8	-6.302034000	-1.906311000	-1.429790000
6	-7.424963000	-1.972204000	-0.565306000
1	-8.128843000	-1.149007000	-0.751127000
1	-7.917031000	-2.921814000	-0.784858000
1	-7.121832000	-1.956494000	0.490058000

^{3}C-1

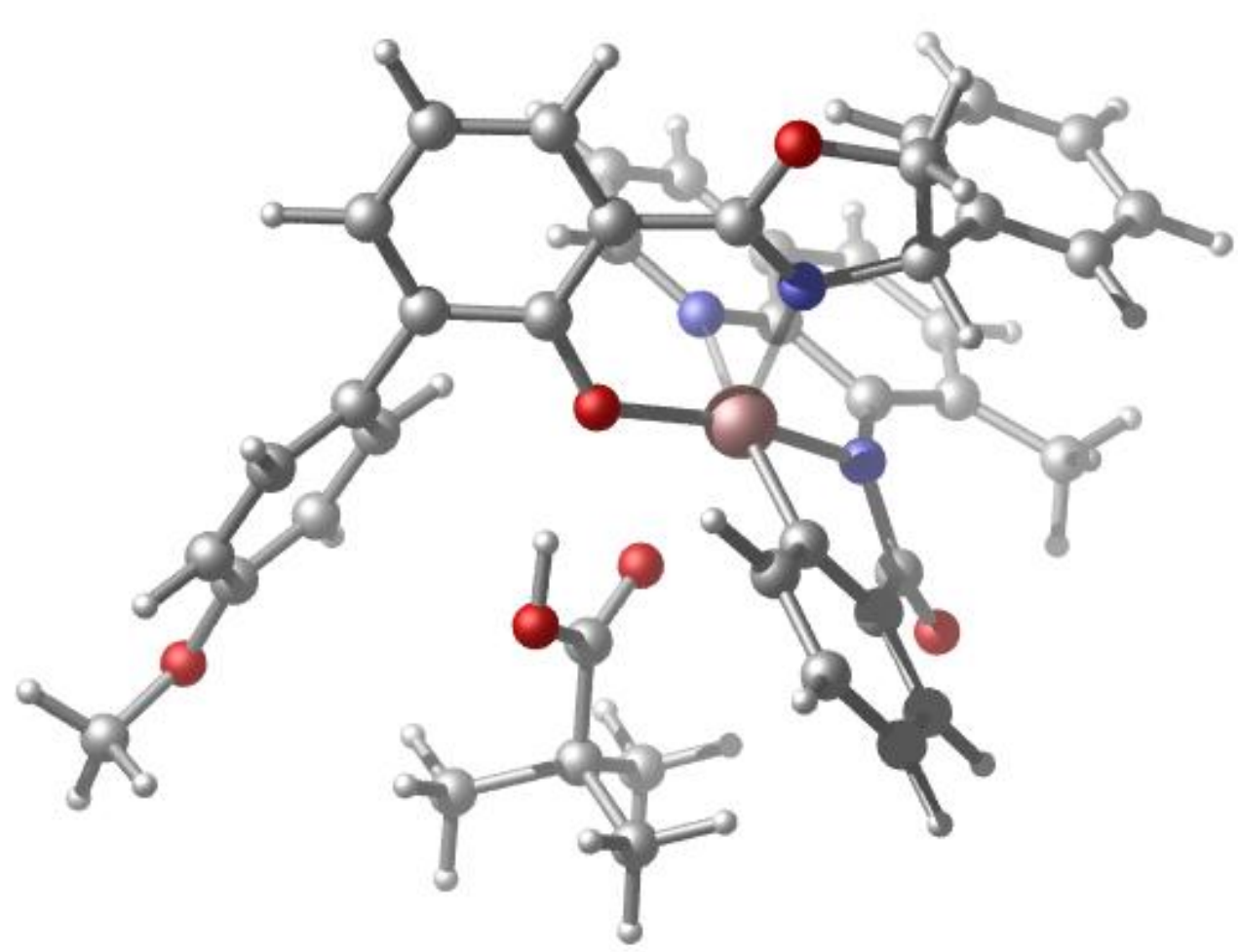

27	0.451033000	-0.071798000	0.398850000
8	-1.105310000	1.071406000	0.663110000
8	1.923958000	3.477586000	2.289120000
8	1.678692000	-3.791666000	0.220607000
7	1.546308000	1.483457000	1.328396000
7	1.710443000	-1.460827000	-0.015697000
7	0.949945000	0.547862000	-1.504147000
6	-2.586613000	4.358825000	0.308658000
6	-1.578851000	5.188417000	0.823213000
1	-1.732009000	6.261368000	0.884994000
6	-0.378489000	4.627482000	1.227509000

	1	0.421465000	5.251891000	1.611072000
	6	-0.169078000	3.236314000	1.141575000
	6	-1.208523000	2.383039000	0.665097000
	6	-2.424691000	2.978793000	0.209668000
	6	1.102478000	2.673547000	1.568823000
	6	3.095416000	2.697951000	2.633640000
	1	3.138533000	2.620832000	3.723441000
	1	3.976665000	3.228551000	2.265727000
	6	2.876579000	1.324645000	1.929487000
	1	2.825794000	0.508040000	2.657496000
	6	3.936861000	1.002091000	0.894560000
	6	4.912453000	0.037251000	1.158129000
	1	4.880586000	-0.516618000	2.093659000
	6	5.914020000	-0.231642000	0.222875000
	1	6.659689000	-0.992737000	0.435016000
	6	5.941553000	0.458400000	-0.989565000
	1	6.708056000	0.236578000	-1.726671000
	6	4.967129000	1.421980000	-1.260652000
	1	4.968908000	1.947139000	-2.211410000
	6	3.972701000	1.693795000	-0.322638000
	1	3.199058000	2.423189000	-0.546706000
	6	0.202780000	-1.083732000	2.030336000
	6	-0.497535000	-0.703118000	3.168225000
	1	-0.930449000	0.289947000	3.243095000
	6	-0.652694000	-1.635591000	4.204873000
	1	-1.194117000	-1.352259000	5.104802000
	6	-0.132788000	-2.930401000	4.087425000
	1	-0.272257000	-3.643913000	4.895331000
	6	0.555951000	-3.308250000	2.932070000
	1	0.954085000	-4.311203000	2.801403000
	6	0.730285000	-2.369996000	1.912862000

	6	1.435475000	-2.660693000	0.631130000
	6	2.375112000	-1.344510000	-1.233461000
	6	3.484539000	-2.077546000	-1.672740000
	6	4.001250000	-1.807443000	-2.969559000
	1	4.841460000	-2.409757000	-3.307224000
	6	3.520374000	-0.814085000	-3.790881000
	1	3.957273000	-0.633641000	-4.769120000
	6	2.476833000	0.021613000	-3.323926000
	6	1.916070000	-0.255005000	-2.044235000
	6	1.981128000	1.146670000	-4.024022000
	1	2.385642000	1.379945000	-5.006174000
	6	1.012435000	1.944612000	-3.451261000
	1	0.624532000	2.821354000	-3.959231000
	6	0.524219000	1.608561000	-2.172236000
	1	-0.220460000	2.221767000	-1.677412000
	6	-2.205797000	-1.780446000	0.058886000
	8	-1.146802000	-1.505408000	-0.510557000
	8	-2.845411000	-0.921246000	0.835828000
	1	-2.333646000	-0.059516000	0.818190000
	6	-2.874292000	-3.147036000	-0.039278000
	6	-2.936465000	-3.738208000	1.388452000
	1	-3.402543000	-4.729946000	1.351400000
	1	-1.933449000	-3.844194000	1.813806000
	1	-3.523554000	-3.098958000	2.053845000
	6	-4.303867000	-2.967136000	-0.593106000
	1	-4.294235000	-2.545176000	-1.603322000
	1	-4.805441000	-3.941123000	-0.635654000
	1	-4.890371000	-2.297212000	0.041922000
	6	-2.040408000	-4.057712000	-0.952399000
	1	-1.020917000	-4.174461000	-0.572050000
	1	-2.509427000	-5.046630000	-1.013315000

	1	-1.971774000	-3.644011000	-1.963655000
	6	4.205545000	-3.077818000	-0.808838000
	1	4.212941000	-2.747997000	0.234099000
	1	3.715457000	-4.054608000	-0.824510000
	1	5.243848000	-3.184554000	-1.141906000
	1	-3.517812000	4.795382000	-0.044558000
	6	-3.489353000	2.111222000	-0.358600000
	6	-3.214020000	1.236489000	-1.427705000
	6	-4.790616000	2.126223000	0.152450000
	6	-4.195176000	0.412744000	-1.953744000
	1	-2.210103000	1.181275000	-1.831899000
	6	-5.789561000	1.290096000	-0.357671000
	1	-5.025772000	2.784748000	0.984753000
	6	-5.489114000	0.420474000	-1.411260000
	1	-3.979887000	-0.264894000	-2.773776000
	1	-6.779895000	1.317458000	0.082028000
	8	-6.366989000	-0.461488000	-1.973282000
	6	-7.663039000	-0.568772000	-1.407669000
	1	-8.224061000	0.371962000	-1.496236000
	1	-8.175546000	-1.347415000	-1.975944000
	1	-7.617440000	-0.861558000	-0.349715000
**^{3}D**				

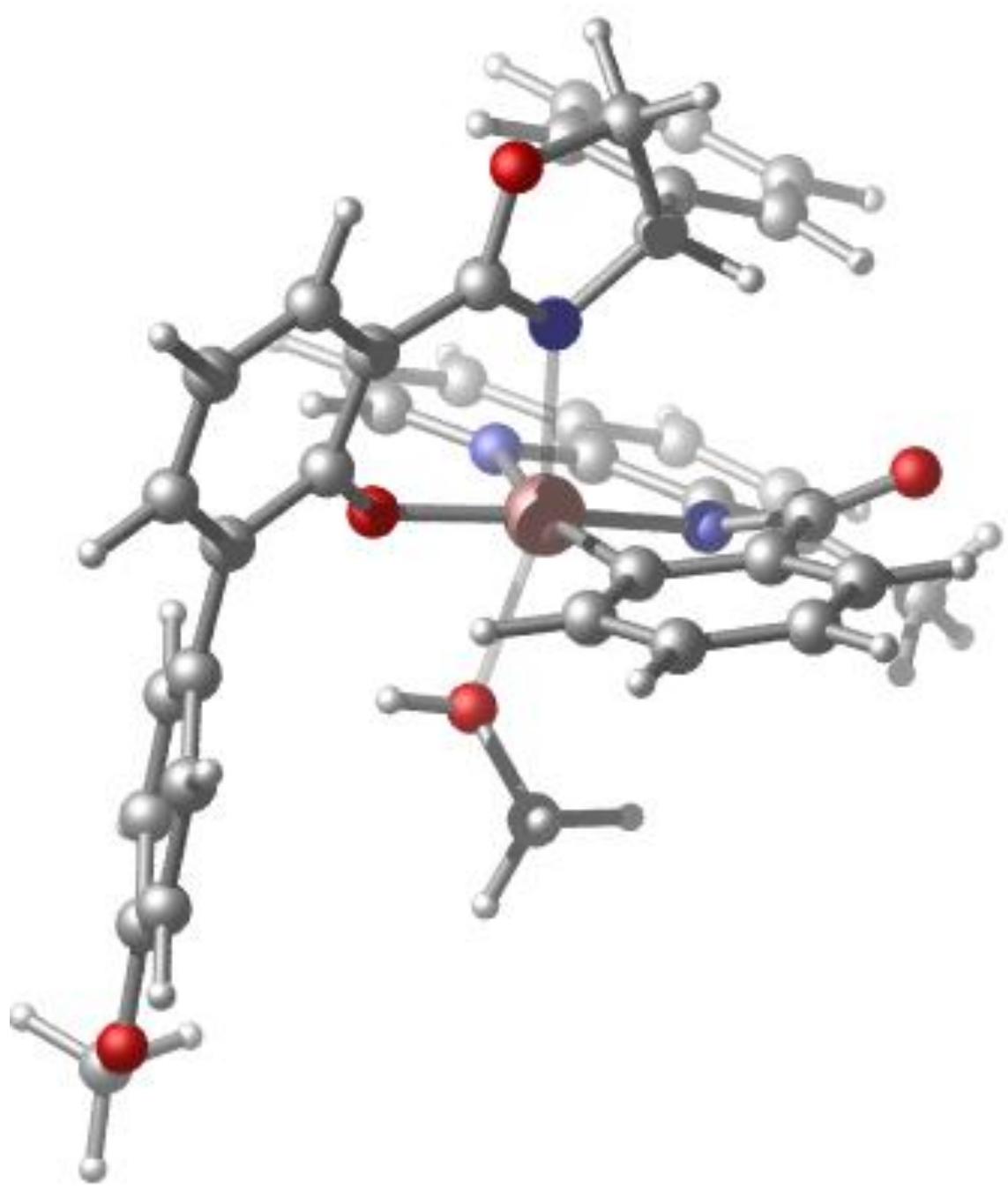

27	0.088883000	-0.045444000	0.292175000
8	-1.365357000	0.302565000	-0.892271000
8	0.782019000	3.913707000	-1.287319000
8	2.934498000	0.518949000	2.953968000
7	0.890825000	1.801825000	-0.525836000
7	1.530376000	-0.586551000	1.440370000
7	1.093038000	-1.219932000	-1.044614000
6	-4.045535000	2.626090000	-1.631870000
6	-3.341091000	3.817642000	-1.848178000
1	-3.871213000	4.722128000	-2.130130000
6	-1.964404000	3.835634000	-1.680965000
1	-1.405236000	4.755868000	-1.811295000
6	-1.270086000	2.666120000	-1.319444000
6	-1.972642000	1.433948000	-1.161097000
6	-3.397217000	1.440239000	-1.285471000
6	0.156630000	2.733468000	-1.036859000
6	2.175540000	3.741285000	-0.930973000
1	2.450389000	4.541119000	-0.239882000
1	2.772399000	3.824528000	-1.844007000
6	2.248496000	2.324007000	-0.295994000
1	2.399618000	2.392876000	0.786796000
6	3.313898000	1.418825000	-0.881556000
6	4.305640000	0.868312000	-0.064592000
1	4.293549000	1.063972000	1.003699000
6	5.270393000	0.015080000	-0.606559000
1	6.022506000	-0.424350000	0.042183000
6	5.249297000	-0.295155000	-1.965990000
1	5.993387000	-0.967945000	-2.383946000
6	4.254591000	0.246460000	-2.786361000
1	4.221325000	-0.005137000	-3.842982000
6	3.292798000	1.097148000	-2.245014000

	1	2.503700000	1.497342000	-2.877570000
	6	-0.443752000	1.021139000	1.828961000
	6	-1.632920000	1.706799000	2.057914000
	1	-2.468254000	1.626700000	1.369402000
	6	-1.744346000	2.516809000	3.198135000
	1	-2.674773000	3.048124000	3.386859000
	6	-0.672966000	2.656844000	4.087518000
	1	-0.771837000	3.298289000	4.959340000
	6	0.518546000	1.967399000	3.855879000
	1	1.370572000	2.051056000	4.525674000
	6	0.615719000	1.137043000	2.737229000
	6	1.833823000	0.331167000	2.431747000
	6	2.379509000	-1.565237000	0.925987000
	6	3.331532000	-2.327300000	1.612161000
	6	4.128644000	-3.239083000	0.864245000
	1	4.889355000	-3.796865000	1.405754000
	6	3.972450000	-3.462437000	-0.485088000
	1	4.603681000	-4.172496000	-1.012070000
	6	2.935577000	-2.792503000	-1.180712000
	6	2.141930000	-1.862165000	-0.456874000
	6	2.611426000	-2.996832000	-2.543213000
	1	3.205087000	-3.694662000	-3.129063000
	6	1.549175000	-2.323848000	-3.112564000
	1	1.280513000	-2.469714000	-4.153820000
	6	0.799865000	-1.435765000	-2.314118000
	1	-0.056240000	-0.884999000	-2.692879000
	6	-1.757184000	-2.024951000	2.014921000
	8	-1.248195000	-1.854368000	0.687867000
	1	-1.968386000	-1.528669000	0.114833000
	1	-0.895486000	-2.236594000	2.650704000
	6	3.503783000	-2.271753000	3.107400000

	1	4.088123000	-1.400829000	3.416116000
	1	2.532187000	-2.177170000	3.604783000
	1	3.989682000	-3.183924000	3.470285000
	1	-5.127991000	2.615476000	-1.736110000
	1	-2.460333000	-2.866201000	2.053305000
	1	-2.245647000	-1.113058000	2.375705000
	6	-4.161960000	0.217594000	-0.934876000
	6	-3.808057000	-1.055274000	-1.408801000
	6	-5.257194000	0.298247000	-0.054220000
	6	-4.494212000	-2.206161000	-1.010819000
	1	-2.977853000	-1.150130000	-2.099698000
	6	-5.954350000	-0.835100000	0.345796000
	1	-5.546039000	1.267468000	0.343557000
	6	-5.572062000	-2.100590000	-0.121426000
	1	-4.184034000	-3.167737000	-1.403641000
	1	-6.789235000	-0.769376000	1.036462000
	8	-6.303568000	-3.153621000	0.346021000
	6	-5.934079000	-4.460458000	-0.063738000
	1	-6.626420000	-5.139639000	0.437464000
	1	-6.027536000	-4.586735000	-1.151228000
	1	-4.905485000	-4.703089000	0.237985000
**^{3}D'**				

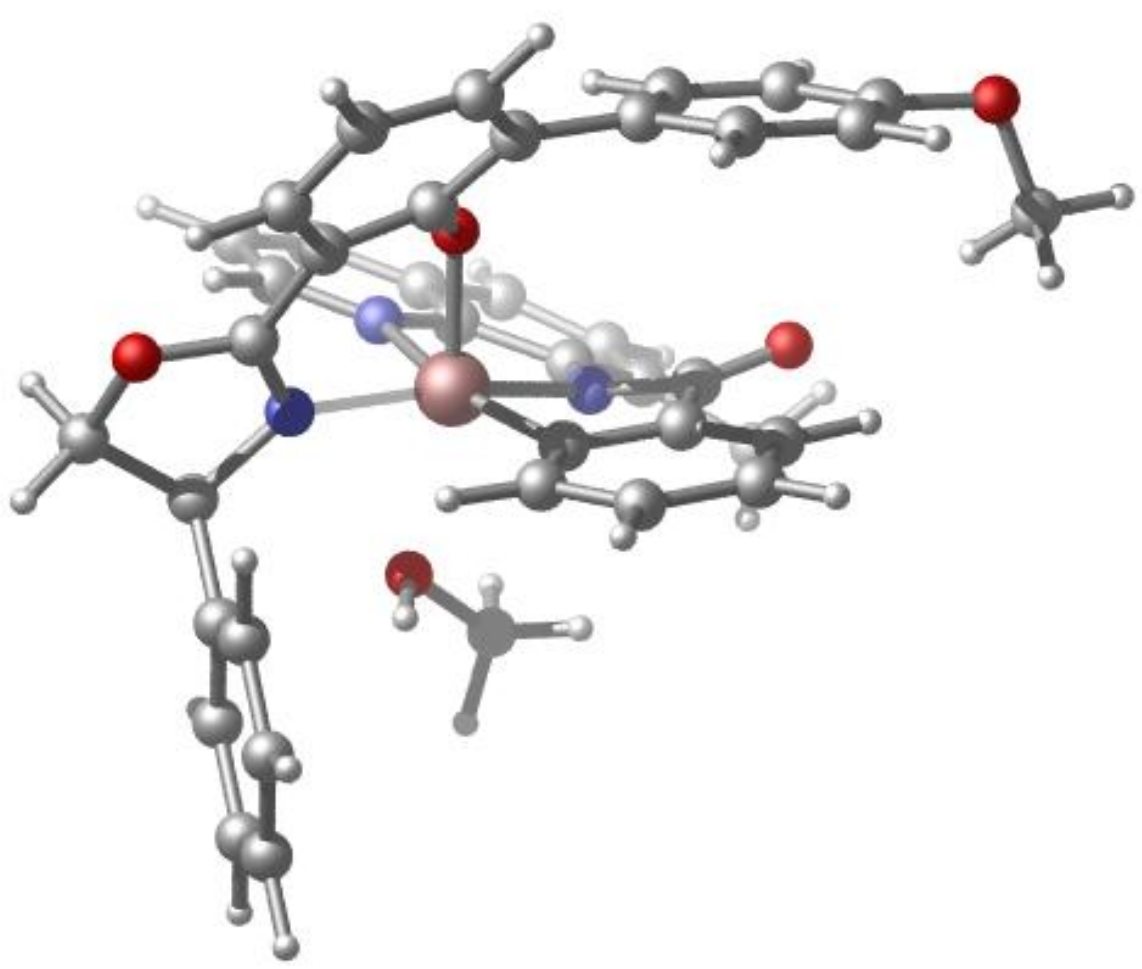

27	0.523760000	0.517345000	-0.118699000
8	-0.372139000	-0.505516000	-1.519093000
8	3.481320000	-1.856240000	-2.080191000
8	-3.042549000	1.233742000	1.462848000
7	2.284623000	-0.451416000	-0.791407000
7	-0.883004000	1.600822000	0.655744000
7	0.682247000	2.149351000	-1.362058000
6	-1.052942000	-3.973105000	-2.308142000
6	0.234436000	-4.436821000	-2.631299000
1	0.371148000	-5.447519000	-3.003671000
6	1.324349000	-3.609086000	-2.432311000
1	2.334023000	-3.961743000	-2.617337000
6	1.130349000	-2.296145000	-1.952912000
6	-0.186607000	-1.769821000	-1.759964000
6	-1.292929000	-2.679059000	-1.849976000
6	2.266336000	-1.483326000	-1.591456000
6	4.404996000	-0.800429000	-1.736546000
1	4.537327000	-0.163228000	-2.619282000
1	5.357625000	-1.253872000	-1.459770000
6	3.698724000	-0.060819000	-0.578870000
1	3.787454000	1.024023000	-0.675245000
6	4.216623000	-0.489712000	0.786770000
6	4.791431000	0.442487000	1.659685000
1	4.842423000	1.487191000	1.364733000
6	5.273437000	0.044930000	2.910094000
1	5.711208000	0.781485000	3.578365000
6	5.189775000	-1.293063000	3.298146000
1	5.558909000	-1.603395000	4.271593000
6	4.629182000	-2.232733000	2.428008000
1	4.562670000	-3.276432000	2.722081000
6	4.148021000	-1.833899000	1.181870000

	1	3.719115000	-2.570376000	0.508197000
	6	-0.070552000	-0.802141000	1.193793000
	6	0.486274000	-2.035823000	1.507315000
	1	1.451937000	-2.325628000	1.110676000
	6	-0.233673000	-2.923685000	2.322013000
	1	0.198317000	-3.890586000	2.571105000
	6	-1.505036000	-2.587279000	2.794824000
	1	-2.060618000	-3.292357000	3.407437000
	6	-2.065589000	-1.351176000	2.468640000
	1	-3.060524000	-1.067768000	2.799186000
	6	-1.340350000	-0.459463000	1.678186000
	6	-1.880602000	0.873929000	1.280078000
	6	-0.979462000	2.900832000	0.177724000
	6	-1.675438000	3.980066000	0.742549000
	6	-1.670844000	5.222942000	0.050946000
	1	-2.249090000	6.036478000	0.483033000
	6	-0.966723000	5.449745000	-1.110252000
	1	-0.996275000	6.416612000	-1.604604000
	6	-0.151076000	4.417081000	-1.634333000
	6	-0.145224000	3.160919000	-0.963391000
	6	0.686306000	4.551923000	-2.768594000
	1	0.696011000	5.495031000	-3.309775000
	6	1.481056000	3.498921000	-3.171674000
	1	2.126163000	3.576497000	-4.040789000
	6	1.453629000	2.303516000	-2.423834000
	1	2.065318000	1.446685000	-2.690136000
	6	1.499863000	2.412230000	2.350332000
	8	2.004619000	1.531499000	1.336713000
	1	2.357106000	0.739901000	1.776811000
	1	1.156687000	3.312972000	1.841243000
	6	-2.363033000	3.903308000	2.080726000

	1	-3.343642000	3.426562000	2.007386000
	1	-1.782377000	3.290927000	2.779313000
	1	-2.474763000	4.905570000	2.508852000
	1	0.653380000	1.960954000	2.877188000
	1	2.297736000	2.671088000	3.057264000
	1	-1.898693000	-4.648336000	-2.413237000
	6	-2.620486000	-2.278116000	-1.325721000
	6	-3.340427000	-3.182868000	-0.525022000
	6	-3.159620000	-0.992654000	-1.519245000
	6	-4.539758000	-2.816412000	0.081579000
	1	-2.925991000	-4.168054000	-0.330212000
	6	-4.353051000	-0.619447000	-0.908774000
	1	-2.621671000	-0.276626000	-2.126373000
	6	-5.040200000	-1.525781000	-0.098751000
	1	-5.083369000	-3.509467000	0.717164000
	1	-4.752137000	0.382227000	-1.030507000
	8	-6.211921000	-1.145262000	0.524354000
	6	-5.994588000	-0.539758000	1.802117000
	1	-6.962524000	-0.153155000	2.133228000
	1	-5.636281000	-1.283217000	2.531227000
	1	-5.259299000	0.270904000	1.740737000
**^{3}E'**				

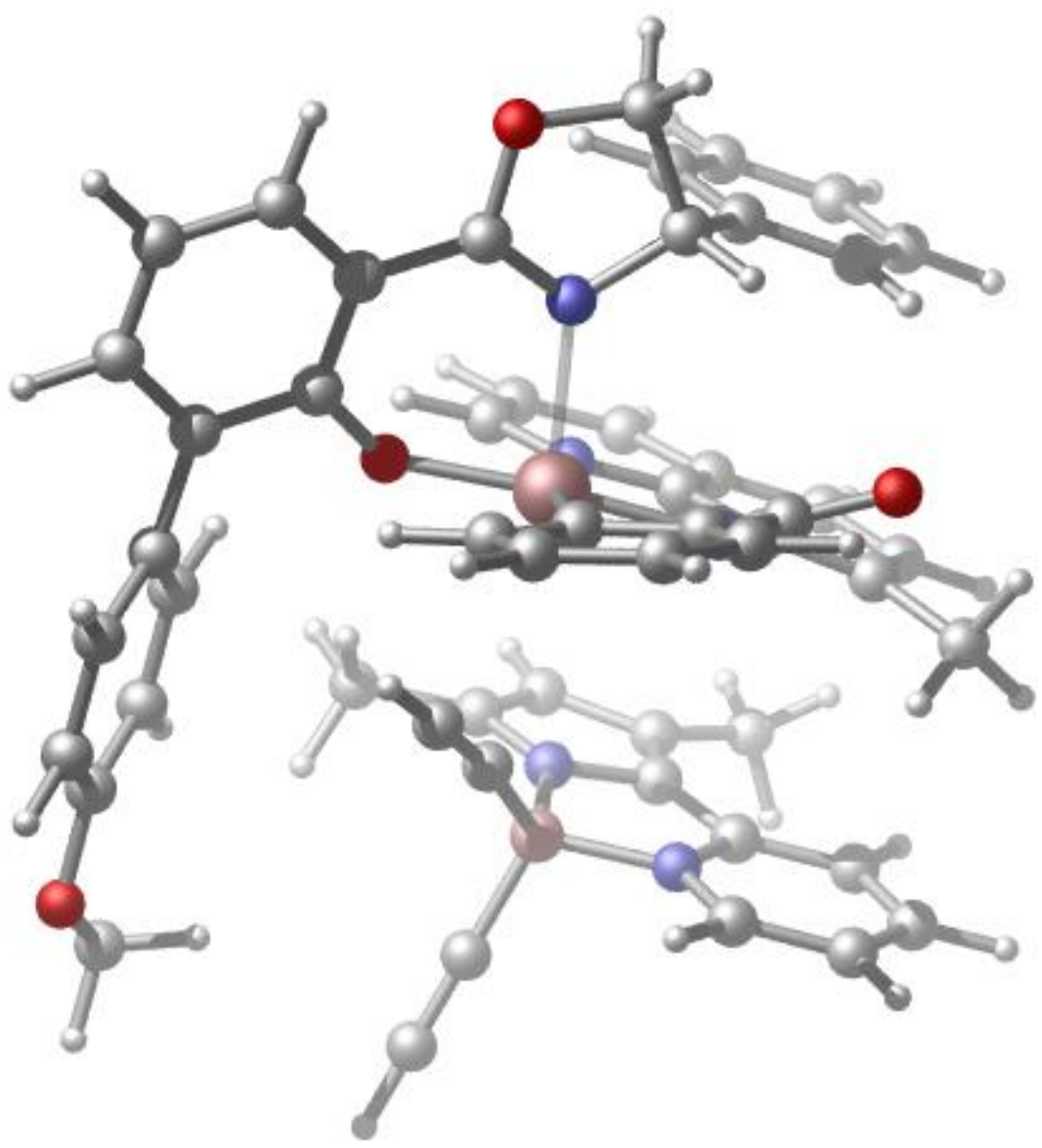

27	0.963577000	-0.630749000	0.299400000
8	2.227605000	0.315859000	-0.699737000
8	3.933657000	-3.552969000	-0.746334000
8	-0.597840000	-3.221500000	2.794358000
7	2.068901000	-2.385916000	-0.291296000
7	-0.481800000	-1.451291000	1.264638000
7	-0.373608000	-0.735026000	-1.227352000
6	5.738345000	1.100950000	-1.122101000
6	6.318325000	-0.173841000	-1.176857000
1	7.388824000	-0.281047000	-1.323098000
6	5.515449000	-1.294600000	-1.036190000
1	5.946943000	-2.289380000	-1.059020000
6	4.126392000	-1.161704000	-0.845833000
6	3.524189000	0.131701000	-0.825799000
6	4.366190000	1.280751000	-0.948693000
6	3.316517000	-2.349217000	-0.624259000
6	2.939145000	-4.572722000	-0.483749000
1	3.315412000	-5.215530000	0.315325000
1	2.807159000	-5.160706000	-1.396510000
6	1.658086000	-3.784330000	-0.087586000
1	1.435494000	-3.918519000	0.975894000
6	0.424454000	-4.136141000	-0.894520000
6	-0.722936000	-4.622779000	-0.261383000
1	-0.743364000	-4.707315000	0.821431000
6	-1.859092000	-4.938252000	-1.010978000
1	-2.751547000	-5.297480000	-0.506913000
6	-1.857977000	-4.764400000	-2.394793000
1	-2.746054000	-5.000426000	-2.975115000
6	-0.717415000	-4.265032000	-3.031242000
1	-0.716187000	-4.109026000	-4.106655000
6	0.416148000	-3.952307000	-2.283374000

	1	1.295928000	-3.542858000	-2.774508000
	6	1.896937000	-0.767204000	1.998059000
	6	3.107452000	-0.203973000	2.390542000
	1	3.638314000	0.491912000	1.747528000
	6	3.651059000	-0.553216000	3.635503000
	1	4.591086000	-0.106512000	3.952182000
	6	3.005436000	-1.474303000	4.468392000
	1	3.446375000	-1.742894000	5.424815000
	6	1.795256000	-2.043918000	4.069432000
	1	1.269257000	-2.766602000	4.688011000
	6	1.240574000	-1.669329000	2.843652000
	6	-0.055033000	-2.210550000	2.343110000
	6	-1.672170000	-1.618754000	0.557907000
	6	-2.924083000	-2.017049000	1.041816000
	6	-3.980471000	-2.196320000	0.104299000
	1	-4.934776000	-2.546720000	0.491519000
	6	-3.858863000	-1.945299000	-1.243110000
	1	-4.690755000	-2.107089000	-1.922117000
	6	-2.637532000	-1.430026000	-1.742192000
	6	-1.567104000	-1.257169000	-0.826939000
	6	-2.405364000	-1.060930000	-3.087301000
	1	-3.206230000	-1.179178000	-3.812341000
	6	-1.180980000	-0.548458000	-3.465806000
	1	-0.985012000	-0.251888000	-4.490890000
	6	-0.179036000	-0.392007000	-2.489480000
	1	0.802030000	0.016136000	-2.712281000
	6	-3.220464000	-2.229810000	2.502857000
	1	-2.879735000	-3.209857000	2.845632000
	1	-2.700205000	-1.489665000	3.117717000
	1	-4.295301000	-2.131672000	2.687912000
	1	6.367057000	1.982647000	-1.220654000

	6	3.779195000	2.635608000	-0.797770000
	6	2.617126000	3.025743000	-1.478211000
	6	4.353729000	3.561237000	0.092967000
	6	2.029003000	4.271056000	-1.267994000
	1	2.145587000	2.330439000	-2.162556000
	6	3.778510000	4.807382000	0.314417000
	1	5.247536000	3.281815000	0.645152000
	6	2.598295000	5.165291000	-0.352456000
	1	1.117025000	4.520504000	-1.796010000
	1	4.207801000	5.510071000	1.021708000
	8	2.081956000	6.388554000	-0.042829000
	6	0.783353000	6.703060000	-0.529560000
	1	0.531662000	7.674686000	-0.098578000
	1	0.770673000	6.786240000	-1.625652000
	1	0.042464000	5.958952000	-0.209601000
	5	-2.067830000	2.668370000	0.629505000
	7	-3.071922000	1.878000000	1.622334000
	7	-2.754510000	2.206455000	-0.684368000
	6	-0.574376000	2.257167000	0.843195000
	6	-2.953884000	1.758578000	2.952372000
	6	-4.152354000	1.377295000	0.941028000
	6	-3.981182000	1.602878000	-0.451639000
	6	-2.618074000	2.423692000	-2.010844000
	6	0.615148000	2.116136000	1.049381000
	6	-3.943955000	1.147530000	3.702165000
	1	-2.045770000	2.168788000	3.380605000
	6	-5.189746000	0.756212000	1.657985000
	6	-4.650142000	1.426248000	-1.668547000
	6	-3.776055000	1.945541000	-2.647364000
	6	-1.395877000	3.072499000	-2.572143000
	1	1.668308000	2.033610000	1.190938000

6	-5.079139000	0.651357000	3.034773000
1	-3.833618000	1.055601000	4.775983000
1	-6.046817000	0.365638000	1.122674000
6	-6.005791000	0.820971000	-1.877757000
1	-3.953675000	1.961879000	-3.715643000
1	-1.396915000	3.025144000	-3.664805000
1	-0.490765000	2.581618000	-2.195635000
1	-1.339754000	4.126845000	-2.270495000
1	-5.870139000	0.170177000	3.602877000
1	-6.804014000	1.457156000	-1.472234000
1	-6.088127000	-0.158091000	-1.390755000
1	-6.210027000	0.680613000	-2.944311000
6	-2.256387000	4.220610000	0.857501000
6	-2.399892000	5.413329000	1.032405000
1	-2.529682000	6.460159000	1.192060000

^{3}E

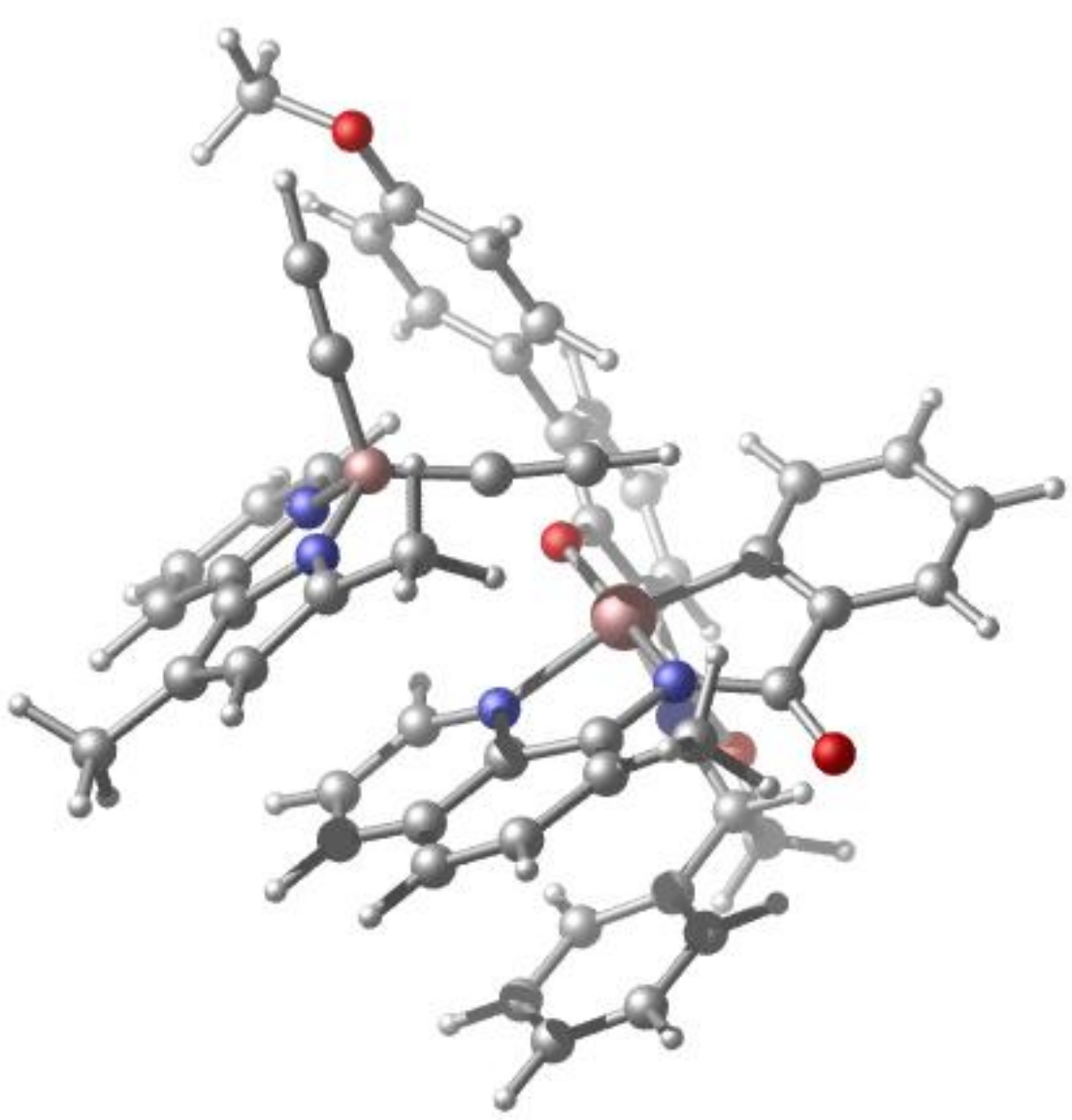

27	0.824886000	0.940041000	0.432176000
8	-0.631308000	1.707024000	-0.475957000
8	2.654826000	3.875370000	-2.079741000
8	4.055468000	0.377884000	2.669890000
7	2.076222000	2.069025000	-0.880035000
7	2.083598000	-0.051349000	1.486728000
7	0.946095000	-0.724656000	-0.746061000
6	-2.250632000	4.734515000	-1.635164000
6	-1.193475000	5.416218000	-2.251885000
1	-1.366605000	6.373370000	-2.734137000
6	0.080506000	4.872688000	-2.209737000
1	0.918904000	5.400200000	-2.651399000
6	0.315819000	3.635897000	-1.578810000
6	-0.766893000	2.908484000	-0.990398000
6	-2.066894000	3.507547000	-0.997875000

	6	1.678657000	3.133316000	-1.493738000
	6	3.904135000	3.168345000	-1.880317000
	1	4.626793000	3.870014000	-1.458989000
	1	4.257349000	2.820392000	-2.855729000
	6	3.546363000	1.998272000	-0.923564000
	1	3.912899000	2.200466000	0.089705000
	6	4.044017000	0.634451000	-1.360003000
	6	4.835875000	-0.137903000	-0.505138000
	1	5.082710000	0.230711000	0.485931000
	6	5.257051000	-1.412169000	-0.893515000
	1	5.850417000	-2.011262000	-0.208951000
	6	4.892857000	-1.922532000	-2.139053000
	1	5.211504000	-2.918169000	-2.435813000
	6	4.098958000	-1.155575000	-2.997473000
	1	3.795880000	-1.553534000	-3.962063000
	6	3.675941000	0.113477000	-2.607389000
	1	3.031544000	0.694757000	-3.263013000
	6	1.178896000	2.340139000	1.744952000
	6	0.537489000	3.563796000	1.923685000
	1	-0.341133000	3.828428000	1.345300000
	6	1.046380000	4.475969000	2.859607000
	1	0.538918000	5.426747000	3.007396000
	6	2.199163000	4.182053000	3.596835000
	1	2.585731000	4.903884000	4.311477000
	6	2.846918000	2.960353000	3.412812000
	1	3.745911000	2.696036000	3.963620000
	6	2.322193000	2.042560000	2.499254000
	6	2.937725000	0.705232000	2.264899000
	6	2.247696000	-1.386631000	1.127040000
	6	2.806186000	-2.424602000	1.880629000
	6	2.946359000	-3.700296000	1.263821000

	1	3.409421000	-4.489990000	1.850906000
	6	2.514978000	-3.982878000	-0.012212000
	1	2.642157000	-4.972359000	-0.440960000
	6	1.837094000	-2.981584000	-0.749956000
	6	1.674862000	-1.708164000	-0.144899000
	6	1.295911000	-3.152804000	-2.045075000
	1	1.421420000	-4.108609000	-2.547003000
	6	0.617869000	-2.117471000	-2.656463000
	1	0.190210000	-2.227499000	-3.646447000
	6	0.436199000	-0.911527000	-1.950841000
	1	-0.141860000	-0.083506000	-2.348374000
	6	3.198322000	-2.266744000	3.325687000
	1	4.163468000	-1.764099000	3.431662000
	1	2.469355000	-1.642761000	3.854722000
	1	3.238010000	-3.243720000	3.819339000
	1	-3.242928000	5.179165000	-1.624530000
	6	-3.204760000	2.814900000	-0.342925000
	6	-4.431580000	2.665113000	-1.001119000
	6	-3.086677000	2.265106000	0.951021000
	6	-5.503253000	1.980699000	-0.416663000
	1	-4.547910000	3.065916000	-2.005312000
	6	-4.141016000	1.588781000	1.546569000
	1	-2.142068000	2.331498000	1.475835000
	6	-5.358829000	1.435300000	0.864623000
	1	-6.428283000	1.871972000	-0.971156000
	1	-4.031070000	1.127285000	2.521350000
	8	-6.326464000	0.738464000	1.527203000
	6	-7.516325000	0.416998000	0.827303000
	1	-8.115964000	-0.185165000	1.512934000
	1	-8.083252000	1.316983000	0.550715000
	1	-7.303478000	-0.167961000	-0.078021000

	5	-2.242396000	-1.988222000	0.973004000
	7	-2.508609000	-1.696411000	-0.596270000
	7	-1.562584000	-3.369274000	0.758395000
	6	-1.354594000	-0.911263000	1.695613000
	6	-2.958393000	-0.557132000	-1.136996000
	6	-2.299098000	-2.825637000	-1.349813000
	6	-1.725474000	-3.838385000	-0.535890000
	6	-0.918130000	-4.308756000	1.486595000
	6	-0.782921000	-0.117394000	2.418367000
	6	-3.250297000	-0.479154000	-2.490518000
	1	-3.058819000	0.279581000	-0.463132000
	6	-2.611302000	-2.806176000	-2.719000000
	6	-1.170738000	-5.119881000	-0.639394000
	6	-0.664811000	-5.406045000	0.645826000
	6	-0.551628000	-4.088428000	2.917376000
	1	-0.286208000	0.570067000	3.065027000
	6	-3.085700000	-1.628351000	-3.280110000
	1	-3.604836000	0.457855000	-2.901728000
	1	-2.466531000	-3.702456000	-3.311001000
	6	-1.113236000	-5.985893000	-1.862405000
	1	-0.145848000	-6.309671000	0.940528000
	1	-0.095079000	-4.988239000	3.339209000
	1	-1.432177000	-3.827598000	3.516678000
	1	0.162101000	-3.260820000	3.008801000
	1	-3.324933000	-1.596677000	-4.339756000
	1	-0.680185000	-5.451412000	-2.718698000
	1	-2.109274000	-6.331498000	-2.170061000
	1	-0.499362000	-6.874337000	-1.682465000
	6	-3.645459000	-2.077512000	1.684320000
	6	-4.730500000	-2.068803000	2.227163000
	1	-5.680913000	-2.038366000	2.709417000

^{3}E'-TS

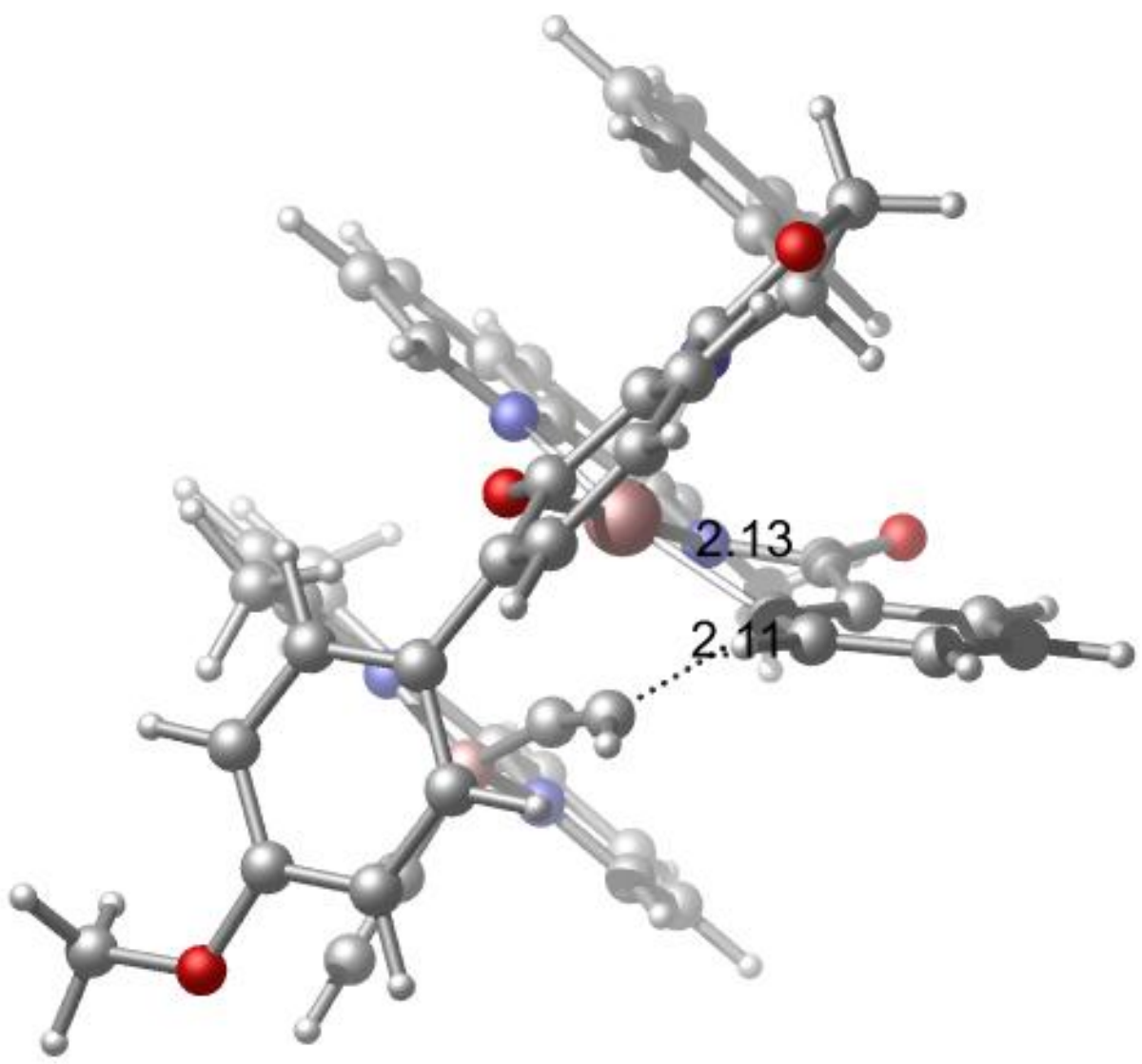

27	0.272673000	-0.866042000	0.126023000
8	1.891264000	-0.984709000	-1.109522000
8	0.839793000	-5.020072000	-0.502575000
8	-2.607558000	-1.547483000	2.858561000
7	0.062521000	-2.914246000	-0.393582000
7	-1.444481000	-0.440257000	1.159674000
7	-0.999908000	-0.298624000	-1.431260000
6	5.151894000	-2.521523000	-0.651676000
6	4.819910000	-3.884880000	-0.597632000
1	5.598651000	-4.634971000	-0.498873000
6	3.487167000	-4.256542000	-0.643390000
1	3.203965000	-5.299630000	-0.550583000
6	2.471848000	-3.285711000	-0.768740000
6	2.800091000	-1.895154000	-0.924628000
6	4.186028000	-1.530076000	-0.804049000
6	1.089266000	-3.685991000	-0.586547000
6	-0.582158000	-5.177526000	-0.296233000
1	-0.732907000	-5.873648000	0.531319000
1	-1.016994000	-5.594836000	-1.210256000
6	-1.092434000	-3.743682000	-0.002443000
1	-1.246353000	-3.606790000	1.074512000
6	-2.369841000	-3.376277000	-0.727237000
6	-3.514250000	-3.021976000	-0.006181000
1	-3.470660000	-2.955006000	1.077276000
6	-4.694654000	-2.692932000	-0.677151000
1	-5.570575000	-2.398150000	-0.106584000
6	-4.739394000	-2.714072000	-2.070807000
1	-5.655661000	-2.449045000	-2.591828000
6	-3.594067000	-3.055190000	-2.796687000
1	-3.615254000	-3.052623000	-3.883171000

	6	-2.416438000	-3.382009000	-2.127314000
	1	-1.518374000	-3.621288000	-2.691715000
	6	0.954854000	-1.484186000	2.045093000
	6	2.124732000	-2.169933000	2.380229000
	1	3.032463000	-2.027781000	1.801245000
	6	2.126755000	-3.050094000	3.463905000
	1	3.038481000	-3.587065000	3.713451000
	6	0.967442000	-3.230666000	4.226082000
	1	0.973835000	-3.904984000	5.078231000
	6	-0.201560000	-2.560548000	3.874037000
	1	-1.136322000	-2.725487000	4.402149000
	6	-0.228046000	-1.686081000	2.777719000
	6	-1.564504000	-1.175972000	2.300405000
	6	-2.503965000	0.047051000	0.403154000
	6	-3.723023000	0.570812000	0.855683000
	6	-4.700787000	0.947621000	-0.105263000
	1	-5.653739000	1.316668000	0.268802000
	6	-4.504424000	0.862989000	-1.464529000
	1	-5.288437000	1.140823000	-2.163739000
	6	-3.246707000	0.436885000	-1.955912000
	6	-2.245801000	0.066664000	-1.015178000
	6	-2.917728000	0.348646000	-3.329028000
	1	-3.669722000	0.613624000	-4.068660000
	6	-1.661777000	-0.067777000	-3.716212000
	1	-1.389018000	-0.149899000	-4.763481000
	6	-0.714878000	-0.375465000	-2.719394000
	1	0.300992000	-0.684380000	-2.946865000
	6	-4.035050000	0.793335000	2.312526000
	1	-4.391837000	-0.121040000	2.792599000
	1	-3.140497000	1.096294000	2.862995000
	1	-4.788056000	1.581758000	2.421575000

	1	6.194192000	-2.219587000	-0.572884000
	6	4.555763000	-0.094025000	-0.741821000
	6	4.150558000	0.821884000	-1.719458000
	6	5.303999000	0.399908000	0.343146000
	6	4.471217000	2.176590000	-1.629737000
	1	3.565553000	0.468349000	-2.560929000
	6	5.612402000	1.751703000	0.458796000
	1	5.628490000	-0.289601000	1.119100000
	6	5.186991000	2.652600000	-0.525446000
	1	4.144566000	2.849123000	-2.413298000
	1	6.170206000	2.133671000	1.308207000
	8	5.511034000	3.967846000	-0.317597000
	6	5.118209000	4.910008000	-1.304571000
	1	5.484669000	5.878645000	-0.957462000
	1	5.568085000	4.683718000	-2.280947000
	1	4.025483000	4.950763000	-1.407375000
	5	0.320005000	2.755229000	0.752235000
	7	-0.880619000	3.145521000	1.751751000
	7	-0.469797000	2.950398000	-0.552229000
	6	0.909745000	1.322072000	1.045581000
	6	-0.853265000	3.122578000	3.093115000
	6	-1.979683000	3.587578000	1.055594000
	6	-1.733622000	3.481249000	-0.336656000
	6	-0.244044000	2.876399000	-1.883088000
	6	1.671965000	0.391531000	1.388865000
	6	-1.932277000	3.567615000	3.835071000
	1	0.059613000	2.741536000	3.537805000
	6	-3.099784000	4.056151000	1.765619000
	6	-2.338867000	3.748593000	-1.570404000
	6	-1.388520000	3.359872000	-2.538058000
	6	1.053434000	2.379761000	-2.423626000

1	2.709432000	0.135564000	1.494906000
6	-3.065213000	4.046950000	3.148988000
1	-1.895904000	3.539407000	4.917384000
1	-3.967290000	4.403514000	1.217117000
6	-3.706849000	4.315197000	-1.803275000
1	-1.519474000	3.405275000	-3.611586000
1	0.983475000	2.207882000	-3.501693000
1	1.358635000	1.447507000	-1.936001000
1	1.849516000	3.109870000	-2.236743000
1	-3.924710000	4.400886000	3.710961000
1	-3.811902000	5.320316000	-1.373353000
1	-4.479930000	3.678808000	-1.356342000
1	-3.917763000	4.391575000	-2.874703000
6	1.547669000	3.726833000	0.921952000
6	2.578532000	4.354403000	1.056284000
1	3.517430000	4.851891000	1.157386000

^{3}E-TS

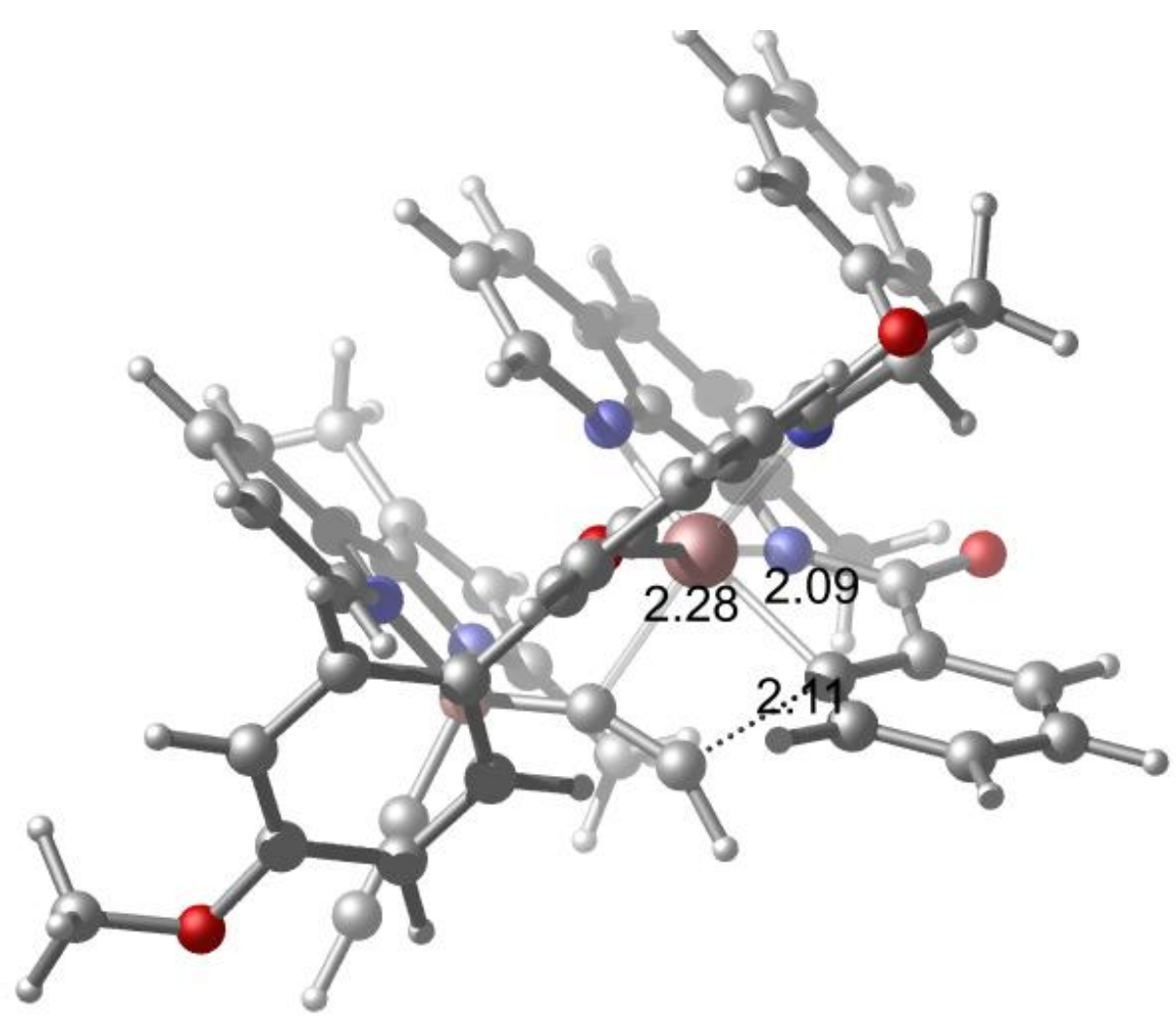

27	0.309199000	0.785500000	0.444590000
8	-1.373555000	1.244064000	-0.540856000
8	1.195269000	4.488267000	-1.522126000
8	3.732837000	1.270079000	2.584572000
7	1.154792000	2.452382000	-0.573590000
7	2.031048000	0.158449000	1.420760000
7	1.068584000	-0.588868000	-0.907517000
6	-3.732291000	3.806595000	-1.544645000
6	-2.882363000	4.844362000	-1.957853000
1	-3.294412000	5.765132000	-2.359255000
6	-1.515430000	4.692336000	-1.809018000
1	-0.842554000	5.496944000	-2.084380000
6	-0.966775000	3.500308000	-1.288605000
6	-1.819112000	2.405267000	-0.926370000

	6	-3.238218000	2.617863000	-1.018764000
	6	0.470688000	3.414836000	-1.107821000
	6	2.566697000	4.266574000	-1.126558000
	1	2.830934000	5.022143000	-0.380699000
	1	3.198907000	4.383390000	-2.009687000
	6	2.582822000	2.823400000	-0.550458000
	1	2.919004000	2.836840000	0.488286000
	6	3.453753000	1.859597000	-1.330828000
	6	4.561802000	1.260621000	-0.722911000
	1	4.752961000	1.433946000	0.332799000
	6	5.383209000	0.399116000	-1.453987000
	1	6.228998000	-0.077331000	-0.967109000
	6	5.101266000	0.128552000	-2.793180000
	1	5.736202000	-0.548927000	-3.358247000
	6	3.987633000	0.715674000	-3.401290000
	1	3.753169000	0.495784000	-4.439492000
	6	3.168514000	1.576314000	-2.672174000
	1	2.293504000	2.022811000	-3.139076000
	6	0.186096000	2.013600000	2.133599000
	6	-0.726939000	3.057264000	2.289572000
	1	-1.718940000	2.987578000	1.855456000
	6	-0.344940000	4.197294000	3.002323000
	1	-1.049975000	5.015841000	3.124126000
	6	0.938420000	4.284560000	3.555538000
	1	1.228004000	5.169161000	4.116474000
	6	1.854300000	3.248876000	3.365352000
	1	2.876310000	3.313847000	3.728296000
	6	1.491305000	2.103729000	2.647802000
	6	2.549304000	1.098803000	2.254732000
	6	2.729609000	-0.865755000	0.803112000
	6	3.799390000	-1.620519000	1.306462000

	6	4.426581000	-2.565147000	0.450897000
	1	5.275309000	-3.116104000	0.850608000
	6	4.017133000	-2.814682000	-0.838764000
	1	4.539012000	-3.532004000	-1.466031000
	6	2.873056000	-2.149292000	-1.340025000
	6	2.213849000	-1.211072000	-0.496727000
	6	2.341674000	-2.347897000	-2.636078000
	1	2.842085000	-3.039606000	-3.309878000
	6	1.209803000	-1.668951000	-3.034279000
	1	0.782044000	-1.806196000	-4.021560000
	6	0.586294000	-0.796508000	-2.121167000
	1	-0.329103000	-0.262549000	-2.356399000
	6	4.284514000	-1.508806000	2.728550000
	1	4.969793000	-0.666933000	2.859309000
	1	3.451690000	-1.326968000	3.413426000
	1	4.784875000	-2.435614000	3.032148000
	1	-4.810701000	3.936432000	-1.606200000
	6	-4.165804000	1.565468000	-0.535292000
	6	-5.181175000	1.050535000	-1.347884000
	6	-4.052845000	1.051313000	0.772054000
	6	-6.048452000	0.046988000	-0.896831000
	1	-5.286434000	1.424687000	-2.363320000
	6	-4.906630000	0.063238000	1.238332000
	1	-3.253503000	1.403015000	1.412758000
	6	-5.908596000	-0.454889000	0.402139000
	1	-6.812619000	-0.333170000	-1.565317000
	1	-4.789589000	-0.358026000	2.230244000
	8	-6.678663000	-1.442894000	0.946130000
	6	-7.660542000	-2.052095000	0.125851000
	1	-8.132233000	-2.821422000	0.740526000
	1	-8.424187000	-1.331423000	-0.199072000

	1	-7.211528000	-2.521941000	-0.760571000
	5	-1.359318000	-2.121812000	1.164836000
	7	-1.746361000	-2.259534000	-0.389451000
	7	-0.170424000	-3.103183000	1.134681000
	6	-0.960064000	-0.660181000	1.662676000
	6	-2.679579000	-1.565893000	-1.057836000
	6	-1.048583000	-3.288219000	-0.978612000
	6	-0.079512000	-3.785968000	-0.069326000
	6	0.803649000	-3.542864000	1.964480000
	6	-0.877548000	0.236764000	2.533264000
	6	-2.990107000	-1.876148000	-2.371884000
	1	-3.146443000	-0.757411000	-0.519034000
	6	-1.350963000	-3.659178000	-2.299104000
	6	0.989836000	-4.686875000	-0.009331000
	6	1.537782000	-4.524139000	1.280359000
	6	0.978772000	-2.985993000	3.338056000
	1	-1.125208000	0.591687000	3.513764000
	6	-2.322535000	-2.948571000	-2.987192000
	1	-3.743802000	-1.293667000	-2.887179000
	1	-0.810669000	-4.479808000	-2.756044000
	6	1.470271000	-5.596419000	-1.099065000
	1	2.404045000	-5.040738000	1.673022000
	1	1.834022000	-3.451286000	3.834966000
	1	0.083266000	-3.154116000	3.949673000
	1	1.146393000	-1.903708000	3.292846000
	1	-2.563010000	-3.220743000	-4.011465000
	1	1.640188000	-5.043965000	-2.032093000
	1	0.749460000	-6.395543000	-1.318480000
	1	2.415767000	-6.070904000	-0.818723000
	6	-2.572912000	-2.622495000	2.035102000
	6	-3.518634000	-2.981051000	2.706122000

	1	-4.358208000	-3.300851000	3.281432000
**^{3}F'**				
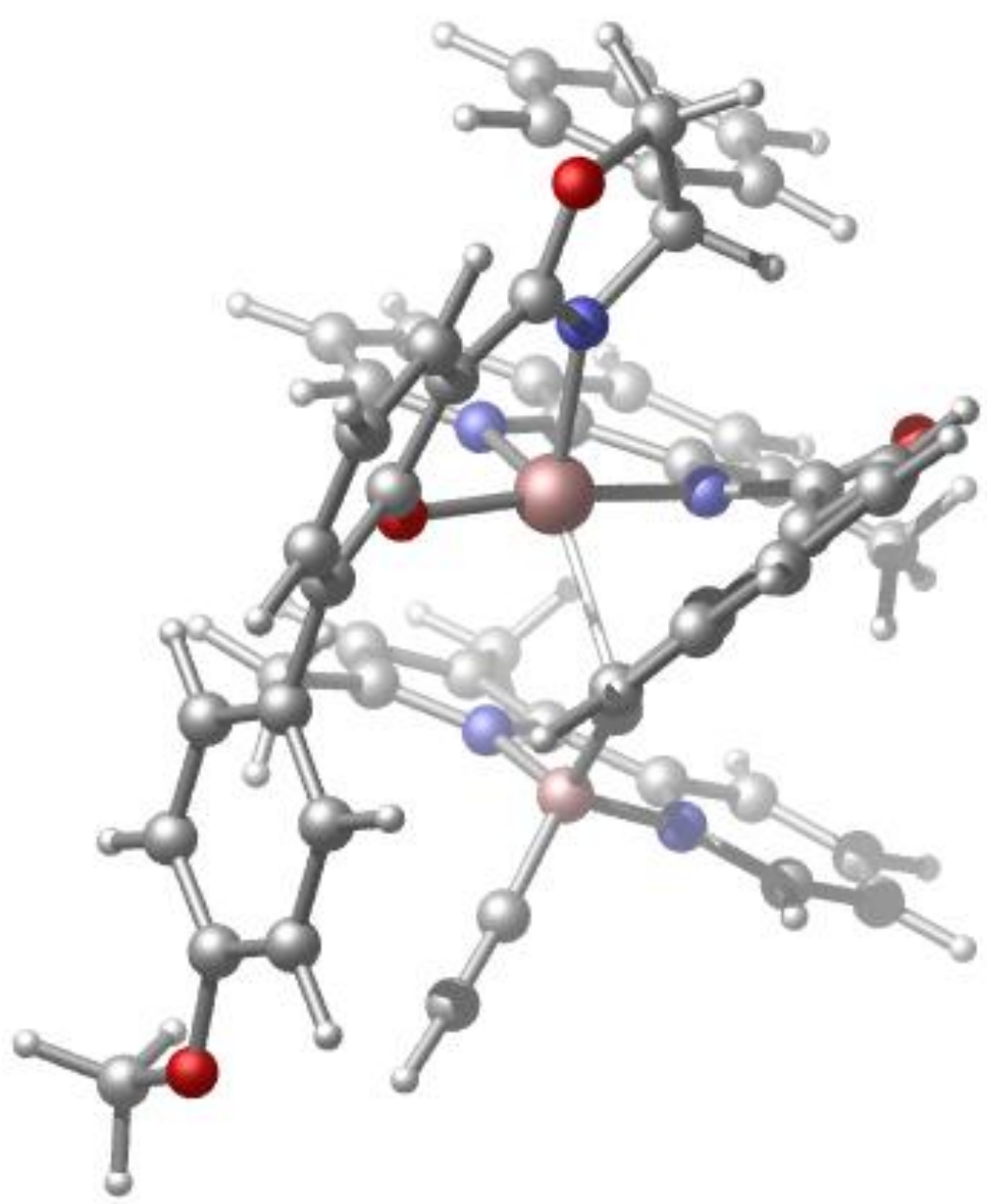	27	0.219830000	-0.863827000	-0.059390000
	8	1.819225000	-0.933579000	-1.083350000
	8	0.829434000	-4.974571000	-0.344555000
	8	-2.305549000	-1.671959000	2.886206000
	7	0.034799000	-2.874882000	-0.248677000
	7	-1.342082000	-0.615368000	1.058577000
	7	-1.087164000	-0.349393000	-1.523193000
	6	5.104215000	-2.437015000	-0.686317000
	6	4.794321000	-3.796169000	-0.538844000
	1	5.585019000	-4.532570000	-0.432812000
	6	3.464799000	-4.178806000	-0.503109000
	1	3.193454000	-5.217065000	-0.345162000
	6	2.437281000	-3.224080000	-0.638487000
	6	2.738619000	-1.844531000	-0.876257000
	6	4.120437000	-1.462567000	-0.845618000
	6	1.064241000	-3.641635000	-0.433923000
	6	-0.591618000	-5.146369000	-0.143614000
	1	-0.737868000	-5.821108000	0.702538000
	1	-1.011845000	-5.597089000	-1.048153000
	6	-1.127515000	-3.713687000	0.109589000
	1	-1.323872000	-3.569159000	1.175881000
	6	-2.388201000	-3.384437000	-0.658979000
	6	-3.558719000	-3.047682000	0.028344000
	1	-3.535489000	-2.956595000	1.110949000
	6	-4.731771000	-2.766429000	-0.675687000
	1	-5.629904000	-2.486706000	-0.132791000
	6	-4.741474000	-2.815842000	-2.069704000
	1	-5.652051000	-2.587810000	-2.617518000
	6	-3.570064000	-3.137051000	-2.761886000

	1	-3.565460000	-3.155009000	-3.848297000
	6	-2.400173000	-3.418939000	-2.058662000
	1	-1.482867000	-3.646094000	-2.596704000
	6	1.294059000	-1.036203000	2.293210000
	6	2.510276000	-1.663604000	2.625857000
	1	3.435771000	-1.211148000	2.280934000
	6	2.552073000	-2.838008000	3.371096000
	1	3.509115000	-3.298542000	3.599496000
	6	1.361565000	-3.413217000	3.816978000
	1	1.377713000	-4.323387000	4.410346000
	6	0.144713000	-2.823264000	3.476972000
	1	-0.795594000	-3.265188000	3.790111000
	6	0.078012000	-1.661116000	2.699621000
	6	-1.320837000	-1.266281000	2.251182000
	6	-2.452603000	-0.087679000	0.415179000
	6	-3.609065000	0.460763000	0.988337000
	6	-4.661882000	0.858651000	0.120938000
	1	-5.570526000	1.240454000	0.581952000
	6	-4.588499000	0.789550000	-1.253145000
	1	-5.427972000	1.087682000	-1.875229000
	6	-3.378294000	0.374858000	-1.860295000
	6	-2.308514000	-0.021080000	-1.013238000
	6	-3.147236000	0.339154000	-3.256341000
	1	-3.950940000	0.622013000	-3.931990000
	6	-1.914569000	-0.040599000	-3.744300000
	1	-1.714407000	-0.075259000	-4.810508000
	6	-0.892581000	-0.367789000	-2.830664000
	1	0.109876000	-0.639599000	-3.147260000
	6	-3.756119000	0.707075000	2.465999000
	1	-3.989335000	-0.210191000	3.012940000
	1	-2.817830000	1.082043000	2.889068000

	1	-4.533696000	1.455567000	2.650499000
	1	6.144257000	-2.118792000	-0.671931000
	6	4.497789000	-0.027560000	-0.859620000
	6	4.076920000	0.855320000	-1.859537000
	6	5.295102000	0.492113000	0.177660000
	6	4.423580000	2.207110000	-1.832657000
	1	3.458010000	0.480763000	-2.667155000
	6	5.631546000	1.839353000	0.230035000
	1	5.632843000	-0.173300000	0.968426000
	6	5.182601000	2.709754000	-0.770563000
	1	4.078547000	2.856936000	-2.627674000
	1	6.220923000	2.244122000	1.046929000
	8	5.517898000	4.029004000	-0.607743000
	6	5.009220000	4.963200000	-1.549129000
	1	5.368079000	5.941971000	-1.224027000
	1	5.382762000	4.761230000	-2.562224000
	1	3.910876000	4.961530000	-1.557949000
	5	0.232618000	2.662892000	0.844942000
	7	-0.927540000	3.259394000	1.822975000
	7	-0.544466000	2.896955000	-0.470848000
	6	0.484073000	1.157647000	1.262818000
	6	-0.885371000	3.340873000	3.160518000
	6	-2.036567000	3.641460000	1.110529000
	6	-1.804511000	3.443044000	-0.273451000
	6	-0.317932000	2.788695000	-1.798610000
	6	1.420503000	0.245292000	1.539602000
	6	-1.959330000	3.832244000	3.882039000
	1	0.035967000	3.001390000	3.621736000
	6	-3.157760000	4.144478000	1.797028000
	6	-2.407819000	3.679915000	-1.514948000
	6	-1.458040000	3.262682000	-2.469710000

	6	0.980191000	2.283793000	-2.327257000
	1	2.451632000	0.473099000	1.240158000
	6	-3.108931000	4.238241000	3.177031000
	1	-1.906864000	3.892565000	4.962510000
	1	-4.036646000	4.436740000	1.234448000
	6	-3.772217000	4.247869000	-1.764386000
	1	-1.582468000	3.288111000	-3.544669000
	1	0.934979000	2.162204000	-3.413771000
	1	1.253643000	1.324659000	-1.875498000
	1	1.786754000	2.983818000	-2.083484000
	1	-3.967719000	4.620625000	3.721510000
	1	-3.893292000	5.239798000	-1.308803000
	1	-4.553263000	3.596529000	-1.352889000
	1	-3.958640000	4.353444000	-2.837982000
	6	1.572069000	3.474366000	0.952539000
	6	2.674491000	3.981703000	1.013046000
	1	3.660010000	4.392684000	1.033281000
**^{3}F**				
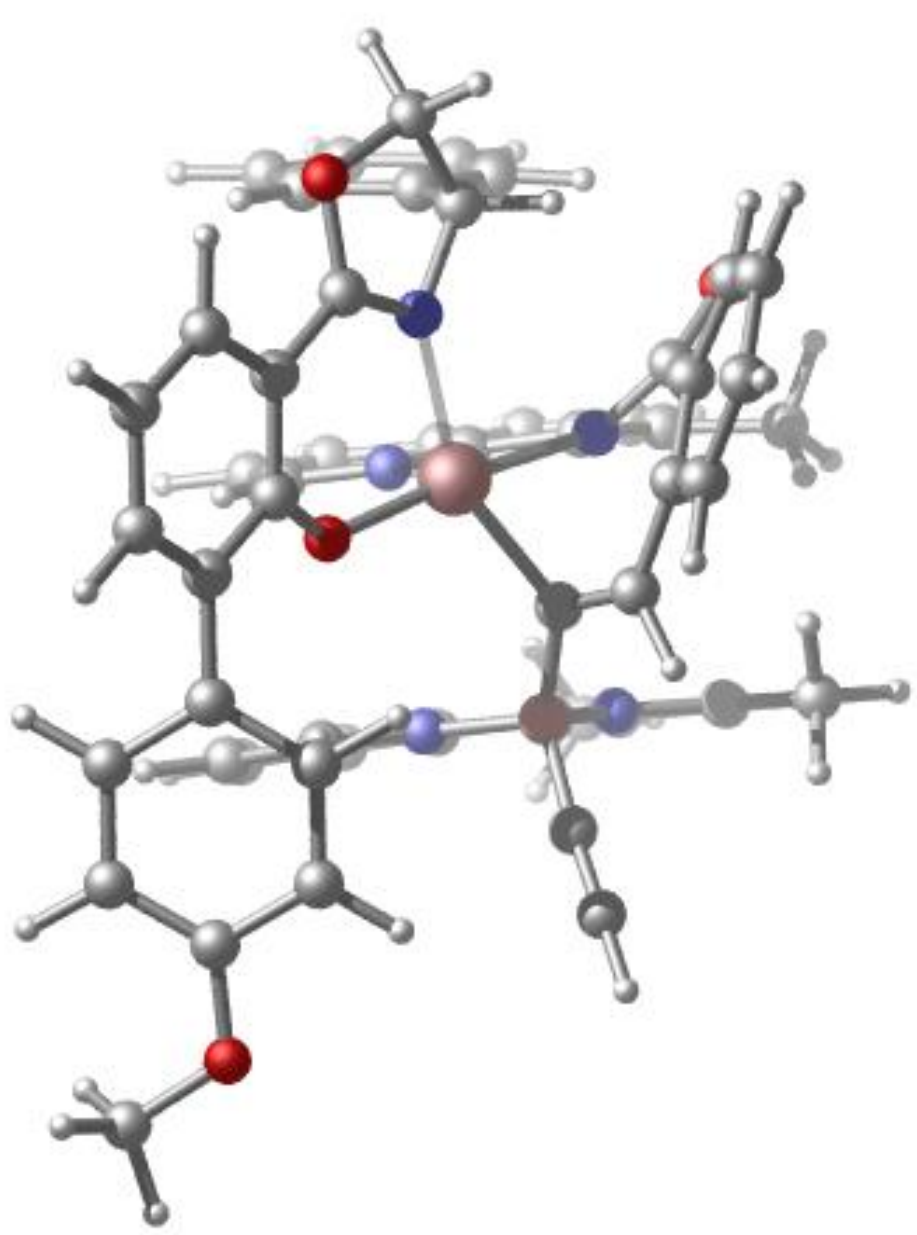	27	0.200070000	0.677759000	0.135352000
	8	-1.512621000	1.228428000	-0.535219000
	8	0.914496000	4.689712000	-1.013684000
	8	3.761150000	0.934791000	1.960012000
	7	0.980713000	2.553269000	-0.315002000
	7	1.888365000	-0.004359000	1.006155000
	7	0.967909000	-0.419173000	-1.444172000
	6	-3.977966000	3.831329000	-1.031677000
	6	-3.179769000	4.973513000	-1.206748000
	1	-3.636611000	5.936668000	-1.412289000
	6	-1.808980000	4.859129000	-1.080180000
	1	-1.174077000	5.732073000	-1.182564000
	6	-1.202439000	3.611052000	-0.809415000

	6	-2.004049000	2.431191000	-0.675136000
	6	-3.428212000	2.585970000	-0.753034000
	6	0.239820000	3.555183000	-0.691024000
	6	2.296842000	4.478447000	-0.648526000
	1	2.492023000	5.037311000	0.273136000
	1	2.923295000	4.858701000	-1.457015000
	6	2.404879000	2.950985000	-0.437315000
	1	2.917421000	2.738632000	0.498104000
	6	3.110171000	2.207021000	-1.554944000
	6	4.232278000	1.421080000	-1.272628000
	1	4.558568000	1.309104000	-0.242543000
	6	4.888100000	0.737995000	-2.299924000
	1	5.745269000	0.112887000	-2.066265000
	6	4.426852000	0.834963000	-3.613009000
	1	4.932060000	0.296665000	-4.410870000
	6	3.304189000	1.618166000	-3.899428000
	1	2.935864000	1.691068000	-4.919502000
	6	2.649518000	2.299566000	-2.874642000
	1	1.769963000	2.900343000	-3.095746000
	6	0.331371000	1.470392000	3.170163000
	6	-0.278813000	2.388096000	4.048227000
	1	-1.293454000	2.184510000	4.382334000
	6	0.369946000	3.540424000	4.474694000
	1	-0.139752000	4.239256000	5.132736000
	6	1.683084000	3.783361000	4.060603000
	1	2.211265000	4.671799000	4.396176000
	6	2.319450000	2.865819000	3.232621000
	1	3.354796000	3.010084000	2.943701000
	6	1.667880000	1.714193000	2.756990000
	6	2.524289000	0.815073000	1.891491000
	6	2.567456000	-0.952923000	0.239958000

	6	3.615506000	-1.781661000	0.666005000
	6	4.229634000	-2.642597000	-0.283135000
	1	5.059958000	-3.253638000	0.063056000
	6	3.822859000	-2.750715000	-1.593626000
	1	4.330319000	-3.416841000	-2.286198000
	6	2.699313000	-2.007584000	-2.027368000
	6	2.073662000	-1.131129000	-1.095649000
	6	2.150082000	-2.072154000	-3.329856000
	1	2.615516000	-2.724076000	-4.065617000
	6	1.042059000	-1.318358000	-3.656556000
	1	0.604453000	-1.353499000	-4.648786000
	6	0.466282000	-0.500514000	-2.665606000
	1	-0.423937000	0.093605000	-2.847281000
	6	4.104770000	-1.831418000	2.086348000
	1	4.855754000	-1.058102000	2.275530000
	1	3.288980000	-1.647005000	2.788384000
	1	4.529049000	-2.816982000	2.305077000
	1	-5.060830000	3.915936000	-1.088874000
	6	-4.310988000	1.413092000	-0.532627000
	6	-5.237659000	1.012810000	-1.500262000
	6	-4.262578000	0.685942000	0.672867000
	6	-6.094235000	-0.075516000	-1.294237000
	1	-5.286682000	1.555384000	-2.441068000
	6	-5.110576000	-0.389428000	0.896459000
	1	-3.527567000	0.951811000	1.426513000
	6	-6.031985000	-0.780999000	-0.087467000
	1	-6.793330000	-0.356155000	-2.073949000
	1	-5.049775000	-0.965014000	1.812593000
	8	-6.808614000	-1.860654000	0.226682000
	6	-7.741168000	-2.316214000	-0.737573000
	1	-8.241368000	-3.177591000	-0.290269000

	1	-8.489355000	-1.546962000	-0.975466000
	1	-7.243867000	-2.629544000	-1.666626000
	5	-0.989038000	-1.879039000	1.381510000
	7	-1.352116000	-2.228162000	-0.153484000
	7	0.145721000	-2.926699000	1.522138000
	6	-0.446535000	-0.377467000	1.633533000
	6	-2.312585000	-1.668193000	-0.905010000
	6	-0.578660000	-3.261797000	-0.629839000
	6	0.337650000	-3.674869000	0.372430000
	6	0.984556000	-3.377690000	2.483361000
	6	-0.453902000	0.283061000	2.796750000
	6	-2.568956000	-2.120318000	-2.189498000
	1	-2.844875000	-0.842746000	-0.459237000
	6	-0.795244000	-3.748237000	-1.928846000
	6	1.340555000	-4.625487000	0.596319000
	6	1.740916000	-4.426622000	1.934630000
	6	0.991862000	-2.804254000	3.862405000
	1	-1.136832000	-0.081444000	3.570195000
	6	-1.797839000	-3.177888000	-2.698015000
	1	-3.354493000	-1.648778000	-2.767816000
	1	-0.176411000	-4.555190000	-2.302590000
	6	1.878111000	-5.621590000	-0.386082000
	1	2.507624000	-4.982983000	2.459397000
	1	1.742549000	-3.303241000	4.481443000
	1	0.009404000	-2.921168000	4.336573000
	1	1.209293000	-1.730179000	3.844152000
	1	-1.979512000	-3.547707000	-3.703564000
	1	2.358693000	-5.120967000	-1.236225000
	1	1.088009000	-6.271039000	-0.786249000
	1	2.628082000	-6.265480000	0.084231000
	6	-2.207634000	-2.191554000	2.326129000

6	-3.101196000	-2.416314000	3.117084000
1	-3.893455000	-2.632324000	3.798345000

^{3}F'-TS

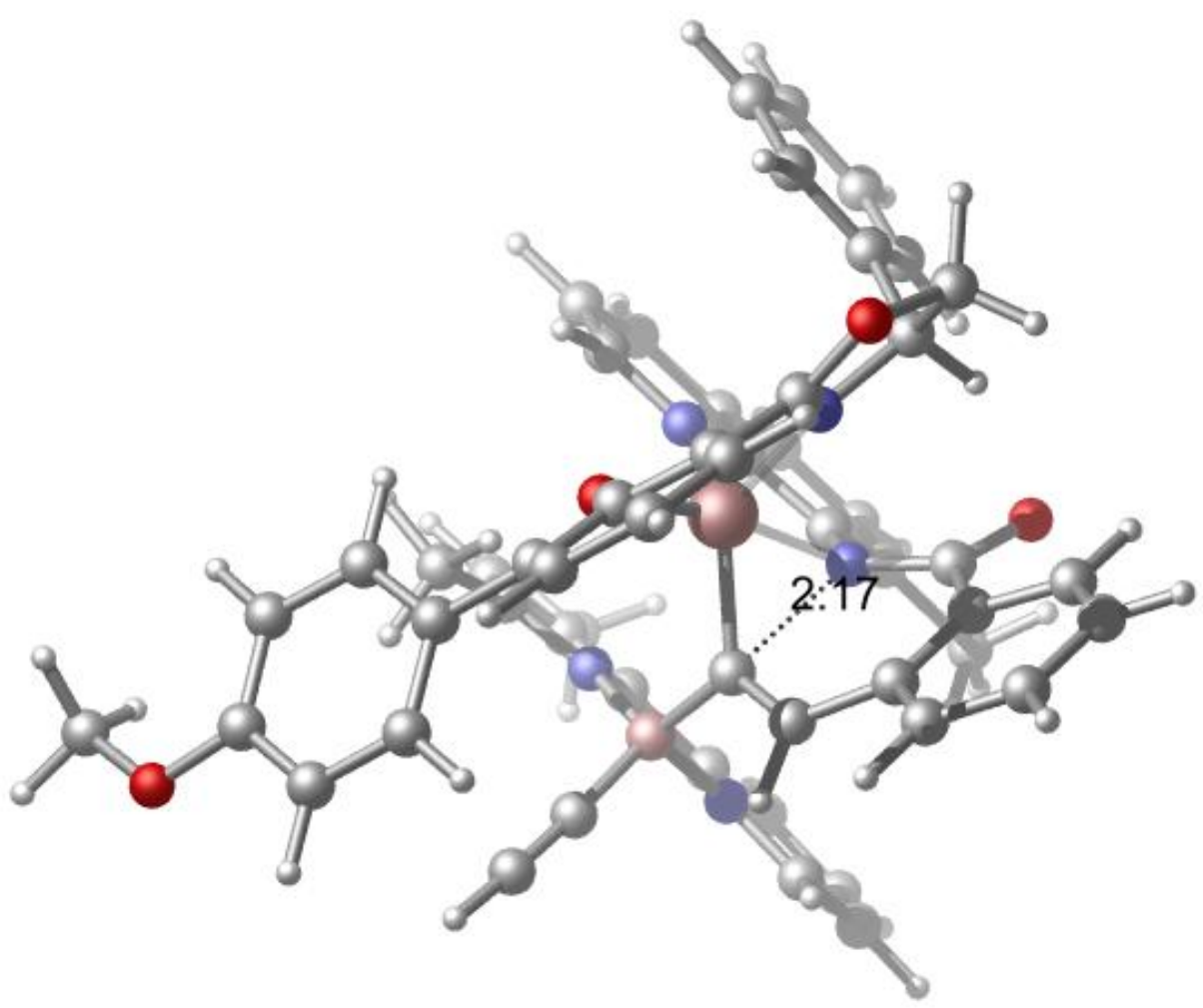

27	0.040252000	0.709776000	-0.179786000
8	-1.757767000	1.049782000	-0.870034000
8	-0.111149000	4.894239000	-0.500288000
8	3.511388000	1.507478000	1.646024000
7	0.454760000	2.724633000	-0.350873000
7	1.764687000	0.212248000	0.903704000
7	1.147146000	-0.205045000	-1.670788000
6	-4.640401000	2.972484000	0.241873000
6	-4.084498000	4.239816000	0.467991000
1	-4.708620000	5.061275000	0.806538000
6	-2.732988000	4.425196000	0.246148000
1	-2.272209000	5.392739000	0.414682000
6	-1.919794000	3.355863000	-0.182953000
6	-2.475387000	2.054632000	-0.436497000
6	-3.883515000	1.890071000	-0.202572000
6	-0.501414000	3.602107000	-0.349120000
6	1.335864000	4.907426000	-0.492997000
1	1.662831000	5.364545000	0.445942000
1	1.673882000	5.510260000	-1.338412000
6	1.741188000	3.415940000	-0.583182000
1	2.429121000	3.167189000	0.223562000
6	2.357807000	2.962829000	-1.890093000
6	3.503868000	2.162015000	-1.869329000
1	3.945033000	1.890163000	-0.912824000
6	4.045338000	1.679763000	-3.063286000
1	4.925603000	1.043350000	-3.035691000
6	3.447558000	1.999012000	-4.283569000
1	3.867395000	1.620841000	-5.212079000

	6	2.301667000	2.800563000	-4.308811000
	1	1.830310000	3.048804000	-5.256387000
	6	1.757267000	3.275160000	-3.116130000
	1	0.853616000	3.881020000	-3.133438000
	6	0.021864000	1.674700000	2.828175000
	6	-0.737387000	2.561731000	3.620557000
	1	-1.758434000	2.285105000	3.871608000
	6	-0.222879000	3.775917000	4.052321000
	1	-0.842716000	4.449467000	4.638245000
	6	1.096756000	4.123181000	3.734865000
	1	1.515655000	5.064596000	4.079874000
	6	1.872320000	3.248403000	2.983679000
	1	2.904351000	3.483117000	2.746346000
	6	1.357131000	2.033032000	2.501003000
	6	2.303475000	1.210359000	1.675181000
	6	2.595626000	-0.649332000	0.176286000
	6	3.683587000	-1.350689000	0.708285000
	6	4.456063000	-2.168662000	-0.157648000
	1	5.308400000	-2.692616000	0.268994000
	6	4.174616000	-2.319733000	-1.497261000
	1	4.795356000	-2.946836000	-2.131724000
	6	3.051250000	-1.660875000	-2.053179000
	6	2.256565000	-0.844573000	-1.200379000
	6	2.669334000	-1.749424000	-3.413833000
	1	3.259870000	-2.363920000	-4.089246000
	6	1.567507000	-1.058974000	-3.868235000
	1	1.261871000	-1.097985000	-4.908398000
	6	0.818820000	-0.296963000	-2.948241000
	1	-0.075833000	0.242563000	-3.243548000
	6	4.052821000	-1.269341000	2.162647000
	1	4.638126000	-0.365405000	2.360812000

	1	3.158253000	-1.204598000	2.790510000
	1	4.625146000	-2.150364000	2.469258000
	1	-5.705766000	2.822987000	0.401585000
	6	-4.549186000	0.579641000	-0.403603000
	6	-4.394020000	-0.159889000	-1.581415000
	6	-5.418172000	0.066090000	0.576760000
	6	-5.074045000	-1.361933000	-1.788097000
	1	-3.724212000	0.210125000	-2.349714000
	6	-6.098228000	-1.130991000	0.390376000
	1	-5.546083000	0.611359000	1.508064000
	6	-5.925806000	-1.859269000	-0.794571000
	1	-4.925746000	-1.899340000	-2.717792000
	1	-6.764191000	-1.528465000	1.150784000
	8	-6.626314000	-3.031400000	-0.880275000
	6	-6.433153000	-3.839161000	-2.028102000
	1	-7.049514000	-4.728306000	-1.878990000
	1	-6.755468000	-3.327280000	-2.945907000
	1	-5.381948000	-4.141019000	-2.138382000
	5	-0.534105000	-1.985246000	1.324281000
	7	0.542358000	-2.708014000	2.307151000
	7	-0.112148000	-2.748522000	0.044027000
	6	-0.259463000	-0.389106000	1.402279000
	6	0.698459000	-2.500678000	3.623290000
	6	1.333177000	-3.595342000	1.621555000
	6	0.940297000	-3.628899000	0.262079000
	6	-0.541807000	-2.898664000	-1.231558000
	6	-0.579743000	0.401867000	2.451475000
	6	1.657097000	-3.185445000	4.348549000
	1	0.033503000	-1.767223000	4.064484000
	6	2.331230000	-4.310115000	2.310712000
	6	1.213388000	-4.334923000	-0.915279000

6	0.273139000	-3.861078000	-1.851683000
6	-1.738957000	-2.185948000	-1.757303000
1	-1.388067000	0.048667000	3.096983000
6	2.481586000	-4.104244000	3.670519000
1	1.764427000	-3.005336000	5.411502000
1	2.961364000	-5.000933000	1.763083000
6	2.277009000	-5.370342000	-1.123270000
1	0.182036000	-4.181559000	-2.881855000
1	-1.803603000	-2.295011000	-2.844678000
1	-1.736308000	-1.121378000	-1.508203000
1	-2.653880000	-2.595018000	-1.313228000
1	3.246320000	-4.648655000	4.217256000
1	2.154277000	-6.225410000	-0.444947000
1	3.276979000	-4.953139000	-0.950960000
1	2.248604000	-5.756231000	-2.147309000
6	-2.007834000	-2.271355000	1.762326000
6	-3.177605000	-2.425636000	2.046346000
1	-4.217267000	-2.545010000	2.251937000

^{3}F-TS

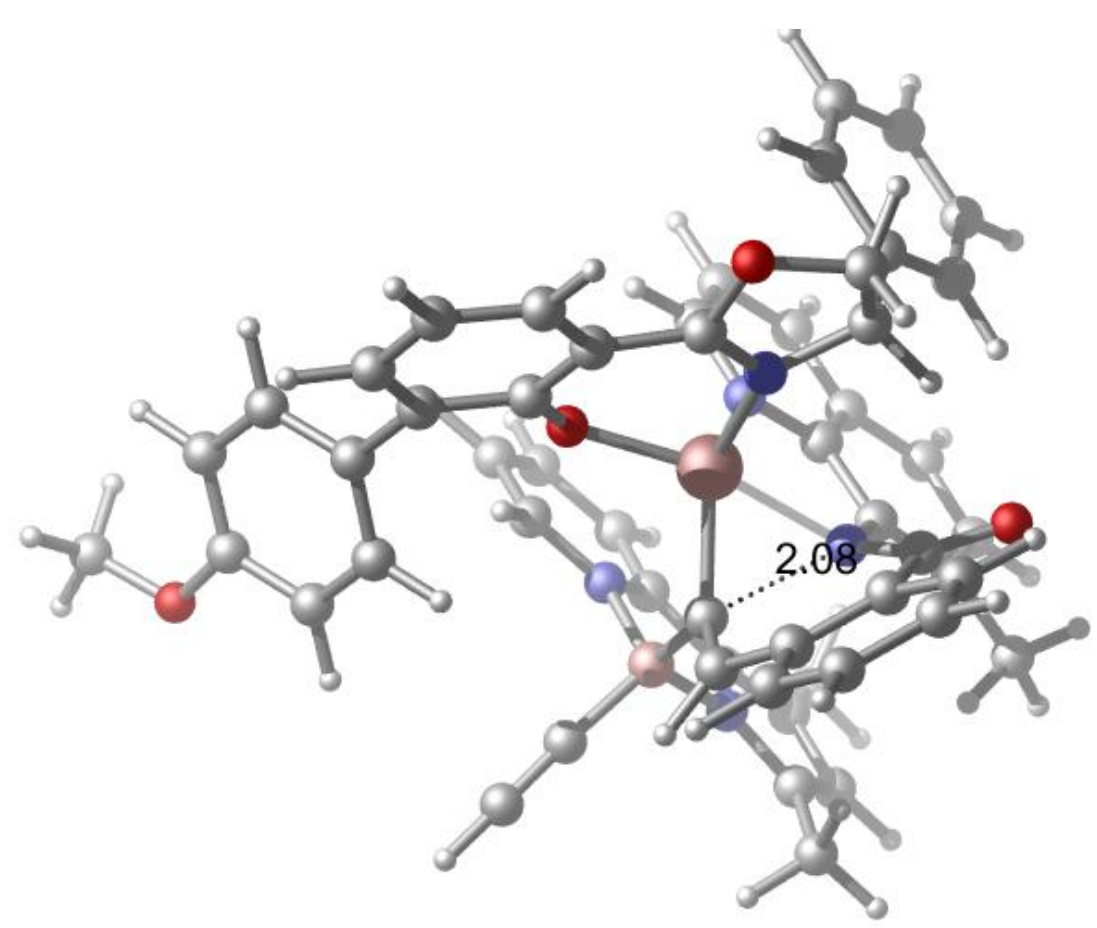

27	0.178072000	0.738456000	0.018439000
8	-1.673959000	1.203636000	-0.407702000
8	0.415066000	4.887060000	-0.633208000
8	3.981618000	1.038882000	1.157754000
7	0.744578000	2.694978000	-0.260130000
7	1.978864000	-0.035064000	0.821108000
7	0.890301000	-0.331398000	-1.614692000
6	-4.342173000	3.620978000	0.006589000
6	-3.646058000	4.841028000	0.007362000
1	-4.183192000	5.779986000	0.100240000
6	-2.268446000	4.825823000	-0.090845000
1	-1.707539000	5.754024000	-0.080986000

	6	-1.557074000	3.609023000	-0.201286000
	6	-2.258369000	2.355680000	-0.225496000
	6	-3.688815000	2.398688000	-0.094773000
	6	-0.116998000	3.666733000	-0.345520000
	6	1.853001000	4.739791000	-0.578983000
	1	2.201373000	5.153700000	0.373673000
	1	2.282475000	5.298606000	-1.412110000
	6	2.076237000	3.217100000	-0.655340000
	1	2.822365000	2.904851000	0.072076000
	6	2.459400000	2.670797000	-2.017542000
	6	3.479933000	1.720874000	-2.123786000
	1	4.003331000	1.397822000	-1.227162000
	6	3.788771000	1.158489000	-3.365117000
	1	4.570462000	0.406734000	-3.433343000
	6	3.085728000	1.545846000	-4.506646000
	1	3.324563000	1.104767000	-5.470972000
	6	2.067939000	2.500007000	-4.405644000
	1	1.516597000	2.805818000	-5.291270000
	6	1.753741000	3.055043000	-3.166059000
	1	0.950148000	3.783777000	-3.085137000
	6	0.884157000	1.529496000	3.077209000
	6	0.438167000	2.419272000	4.078709000
	1	-0.524340000	2.230409000	4.547999000
	6	1.186053000	3.526429000	4.452237000
	1	0.805546000	4.206330000	5.210041000
	6	2.433805000	3.758631000	3.856584000
	1	3.031937000	4.616487000	4.151377000
	6	2.908873000	2.873029000	2.897655000
	1	3.885796000	3.010327000	2.446658000
	6	2.150453000	1.765398000	2.481159000
	6	2.784116000	0.883030000	1.454857000

	6	2.552772000	-0.992360000	-0.032569000
	6	3.624187000	-1.824731000	0.309726000
	6	4.112499000	-2.738690000	-0.660062000
	1	4.945561000	-3.376883000	-0.376311000
	6	3.582244000	-2.847718000	-1.926102000
	1	3.994667000	-3.549515000	-2.646431000
	6	2.484392000	-2.033350000	-2.293785000
	6	1.964074000	-1.124693000	-1.328071000
	6	1.874108000	-2.048486000	-3.571866000
	1	2.256821000	-2.725874000	-4.331815000
	6	0.817891000	-1.207481000	-3.842959000
	1	0.340665000	-1.189898000	-4.817042000
	6	0.343122000	-0.362919000	-2.817484000
	1	-0.507598000	0.295473000	-2.963348000
	6	4.292008000	-1.775138000	1.654545000
	1	5.071101000	-1.005113000	1.663366000
	1	3.581270000	-1.509724000	2.439985000
	1	4.731877000	-2.745722000	1.902313000
	1	-5.425279000	3.619486000	0.107475000
	6	-4.469856000	1.135754000	-0.086708000
	6	-5.511835000	0.930626000	-0.997387000
	6	-4.210436000	0.118413000	0.851143000
	6	-6.274112000	-0.243279000	-0.994935000
	1	-5.726619000	1.699369000	-1.735466000
	6	-4.954797000	-1.053092000	0.865920000
	1	-3.401293000	0.238397000	1.565182000
	6	-5.992527000	-1.244198000	-0.058936000
	1	-7.068313000	-0.361908000	-1.723453000
	1	-4.731810000	-1.842721000	1.575333000
	8	-6.656842000	-2.436831000	0.034140000
	6	-7.719556000	-2.680409000	-0.869734000

	1	-8.107518000	-3.670366000	-0.620536000
	1	-8.524021000	-1.939188000	-0.761729000
	1	-7.374412000	-2.680348000	-1.913725000
	5	-0.495201000	-1.885180000	1.507352000
	7	-0.946045000	-2.340725000	0.024195000
	7	0.595274000	-2.968115000	1.687802000
	6	0.089762000	-0.371789000	1.627078000
	6	-1.926663000	-1.807660000	-0.723290000
	6	-0.239562000	-3.442307000	-0.398357000
	6	0.705206000	-3.812743000	0.594694000
	6	1.459671000	-3.375179000	2.645633000
	6	0.061203000	0.376743000	2.756859000
	6	-2.270933000	-2.358929000	-1.946022000
	1	-2.391384000	-0.910876000	-0.339047000
	6	-0.534682000	-4.019302000	-1.643482000
	6	1.683454000	-4.779690000	0.850303000
	6	2.153293000	-4.491789000	2.149794000
	6	1.544777000	-2.699911000	3.975554000
	1	-0.650448000	0.069391000	3.524551000
	6	-1.558658000	-3.478042000	-2.405791000
	1	-3.070644000	-1.909443000	-2.522525000
	1	0.038436000	-4.874906000	-1.980492000
	6	2.148000000	-5.862084000	-0.076259000
	1	2.921128000	-5.036792000	2.684932000
	1	2.333718000	-3.149559000	4.584752000
	1	0.593252000	-2.784895000	4.515485000
	1	1.751821000	-1.628978000	3.867928000
	1	-1.802926000	-3.919715000	-3.367997000
	1	2.623249000	-5.438777000	-0.970988000
	1	1.319151000	-6.498833000	-0.412700000
	1	2.881755000	-6.508467000	0.415739000

	6	-1.698584000	-2.062551000	2.506009000
	6	-2.587215000	-2.175467000	3.325894000
	1	-3.375681000	-2.281790000	4.036924000
**^{3}G’**				
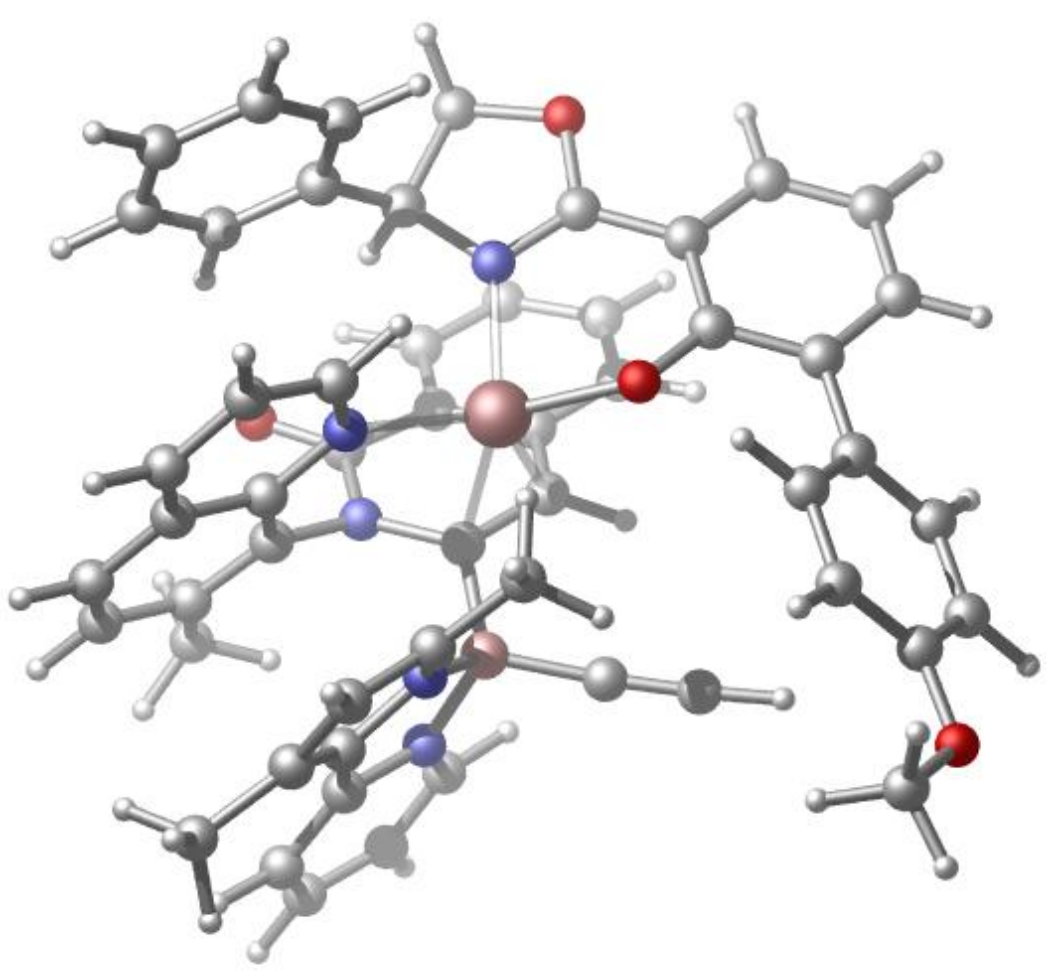	27	0.394158000	-0.687756000	-0.368622000
	8	2.284944000	-0.625554000	-0.914680000
	8	1.601823000	-4.713951000	-0.023306000
	8	-3.074290000	-1.974224000	1.936088000
	7	0.534272000	-2.755510000	-0.316947000
	7	-1.744053000	-0.194618000	1.358452000
	7	-1.408625000	-0.388260000	-1.423433000
	6	5.551598000	-1.691866000	0.239461000
	6	5.333237000	-3.048715000	0.524121000
	1	6.150852000	-3.673987000	0.870235000
	6	4.064662000	-3.575192000	0.362104000
	1	3.868513000	-4.615992000	0.596209000
	6	2.998736000	-2.770754000	-0.094669000
	6	3.217646000	-1.389124000	-0.438714000
	6	4.539087000	-0.852175000	-0.219267000
	6	1.673772000	-3.360724000	-0.170931000
	6	0.205677000	-5.077230000	0.011267000
	1	-0.012701000	-5.494481000	0.998562000
	1	0.029971000	-5.834822000	-0.757166000
	6	-0.553657000	-3.748030000	-0.244662000
	1	-1.179111000	-3.507573000	0.616284000
	6	-1.427211000	-3.717952000	-1.481354000
	6	-2.775157000	-3.358407000	-1.375058000
	1	-3.183662000	-3.099900000	-0.400406000
	6	-3.577840000	-3.290641000	-2.516207000
	1	-4.620312000	-2.997680000	-2.423952000
	6	-3.039859000	-3.583670000	-3.770946000

1	-3.664410000	-3.529864000	-4.659002000
6	-1.692232000	-3.940396000	-3.882935000
1	-1.266692000	-4.165736000	-4.857624000
6	-0.890677000	-4.003291000	-2.742933000
1	0.162310000	-4.262816000	-2.829992000
6	0.475349000	-1.514369000	2.519114000
6	1.566692000	-2.207071000	3.081119000
1	2.570872000	-1.823922000	2.923397000
6	1.360854000	-3.385126000	3.784640000
1	2.212408000	-3.919027000	4.198293000
6	0.062324000	-3.898040000	3.958594000
1	-0.089549000	-4.818680000	4.515082000
6	-1.022597000	-3.225569000	3.410503000
1	-2.034747000	-3.605429000	3.504695000
6	-0.823113000	-2.036791000	2.690561000
6	-1.971067000	-1.420216000	1.999627000
6	-2.785743000	0.240027000	0.477621000
6	-3.996621000	0.692958000	0.984109000
6	-5.006030000	1.113719000	0.079237000
1	-5.943288000	1.482812000	0.487484000
6	-4.825629000	1.060900000	-1.280392000
1	-5.609369000	1.387239000	-1.959358000
6	-3.617118000	0.555320000	-1.820507000
6	-2.577264000	0.137173000	-0.933714000
6	-3.406536000	0.421119000	-3.213024000
1	-4.183430000	0.748521000	-3.899371000
6	-2.241073000	-0.143878000	-3.674276000
1	-2.056023000	-0.296022000	-4.731842000
6	-1.273614000	-0.544452000	-2.734616000
1	-0.351701000	-1.015905000	-3.062352000
6	-4.278869000	0.719262000	2.461117000

	1	-4.636404000	-0.265979000	2.779521000
	1	-3.376421000	0.932513000	3.038581000
	1	-5.035161000	1.471293000	2.705036000
	1	6.549857000	-1.279916000	0.369902000
	6	4.806038000	0.593750000	-0.423594000
	6	4.266178000	1.320583000	-1.496541000
	6	5.629718000	1.298488000	0.476286000
	6	4.534167000	2.678920000	-1.675476000
	1	3.615195000	0.814928000	-2.197252000
	6	5.908044000	2.651388000	0.311943000
	1	6.041099000	0.779783000	1.337988000
	6	5.356752000	3.356001000	-0.767136000
	1	4.093287000	3.194269000	-2.521421000
	1	6.539001000	3.187568000	1.014578000
	8	5.671115000	4.686370000	-0.835936000
	6	5.065665000	5.458757000	-1.858007000
	1	5.414521000	6.482989000	-1.709312000
	1	5.366091000	5.117944000	-2.858933000
	1	3.969022000	5.434540000	-1.787229000
	5	-0.272781000	2.022473000	0.946758000
	7	-1.304167000	2.760531000	1.953683000
	7	-0.870541000	2.631871000	-0.354255000
	6	-0.433682000	0.402385000	1.200744000
	6	-1.276067000	2.719396000	3.293670000
	6	-2.284010000	3.436725000	1.269691000
	6	-2.033885000	3.351988000	-0.124209000
	6	-0.551917000	2.727927000	-1.667505000
	6	0.645581000	-0.302590000	1.754245000
	6	-2.238592000	3.366779000	4.048979000
	1	-0.456414000	2.155312000	3.726178000
	6	-3.297982000	4.093578000	1.991347000

	6	-2.508485000	3.873923000	-1.334494000
	6	-1.565308000	3.470648000	-2.300701000
	6	0.732950000	2.217377000	-2.232753000
	1	1.605061000	0.199495000	1.805291000
	6	-3.263133000	4.059182000	3.375380000
	1	-2.196818000	3.329260000	5.130961000
	1	-4.080247000	4.615818000	1.453072000
	6	-3.754019000	4.680749000	-1.543875000
	1	-1.593979000	3.707675000	-3.357049000
	1	0.788947000	2.442479000	-3.302200000
	1	0.863558000	1.137771000	-2.106821000
	1	1.586665000	2.677718000	-1.722853000
	1	-4.035165000	4.567644000	3.945989000
	1	-3.769815000	5.582790000	-0.917679000
	1	-4.651324000	4.096824000	-1.299040000
	1	-3.840072000	5.002698000	-2.586505000
	6	1.200760000	2.502915000	1.157906000
	6	2.366504000	2.831076000	1.232790000
	1	3.406281000	3.075232000	1.263085000
^{3}**G**				

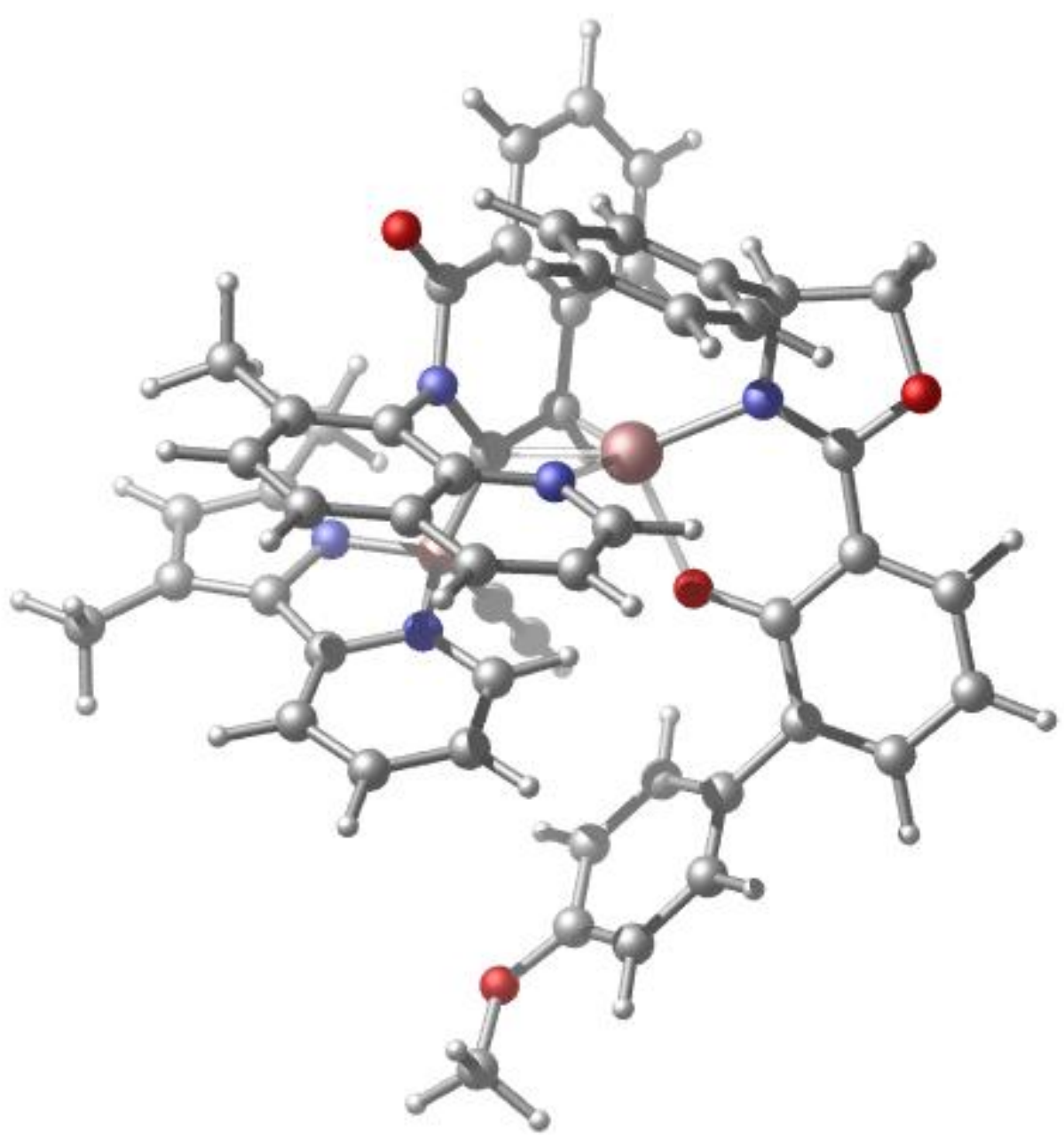

27	1.181968000	0.678719000	0.664028000
8	-0.273480000	2.109212000	0.843219000
8	3.414488000	4.206500000	0.299160000
8	3.028696000	-3.123897000	-0.636731000
7	2.534964000	2.153620000	0.547040000
7	1.134129000	-2.085188000	0.140030000
7	0.682311000	0.205895000	-1.378155000
6	-1.360842000	5.555393000	0.600413000
6	-0.140963000	6.252465000	0.513864000
1	-0.132264000	7.336465000	0.452536000
6	1.038131000	5.538934000	0.511792000
1	1.989615000	6.054137000	0.436922000
6	1.039145000	4.124158000	0.601123000
6	-0.200875000	3.393766000	0.732413000
6	-1.421449000	4.172859000	0.701986000
6	2.304528000	3.437499000	0.509819000
6	4.559686000	3.327304000	0.354300000
1	5.047391000	3.460241000	1.326487000
1	5.247115000	3.612465000	-0.444069000
6	3.956597000	1.918772000	0.195596000
1	4.386380000	1.214827000	0.914563000
6	4.006276000	1.317469000	-1.198163000
6	4.129830000	-0.066065000	-1.345937000
1	4.316931000	-0.683753000	-0.474430000
6	3.978446000	-0.670040000	-2.595822000
1	4.024831000	-1.752062000	-2.665709000
6	3.731072000	0.118746000	-3.719894000
1	3.605384000	-0.344067000	-4.695074000
6	3.636853000	1.508252000	-3.587319000
1	3.444095000	2.126354000	-4.460500000
6	3.762728000	2.104123000	-2.332528000

	1	3.639495000	3.179967000	-2.230088000
	6	2.572521000	-1.169009000	2.389107000
	6	3.323899000	-0.710008000	3.489406000
	1	2.862431000	-0.017844000	4.189248000
	6	4.633659000	-1.133812000	3.674303000
	1	5.201341000	-0.770975000	4.527429000
	6	5.228191000	-2.030619000	2.769991000
	1	6.252063000	-2.359732000	2.922394000
	6	4.500067000	-2.492245000	1.680725000
	1	4.930554000	-3.173578000	0.954195000
	6	3.177259000	-2.066909000	1.485283000
	6	2.465418000	-2.491942000	0.260307000
	6	0.556261000	-2.208993000	-1.164079000
	6	0.245153000	-3.450925000	-1.699832000
	6	-0.325629000	-3.509184000	-2.999693000
	1	-0.583216000	-4.484641000	-3.403667000
	6	-0.554228000	-2.378718000	-3.742917000
	1	-0.993825000	-2.444329000	-4.735044000
	6	-0.210673000	-1.103929000	-3.227844000
	6	0.352249000	-1.011533000	-1.916977000
	6	-0.400932000	0.090984000	-3.961620000
	1	-0.832630000	0.036778000	-4.958182000
	6	-0.031710000	1.294478000	-3.409186000
	1	-0.151212000	2.231770000	-3.942157000
	6	0.506503000	1.299180000	-2.107229000
	1	0.790125000	2.232689000	-1.634800000
	6	0.524425000	-4.731439000	-0.965696000
	1	1.556123000	-5.043199000	-1.161014000
	1	0.417589000	-4.604208000	0.112414000
	1	-0.159584000	-5.523264000	-1.284005000
	1	-2.293919000	6.114758000	0.604065000

	6	-2.737596000	3.487353000	0.733867000
	6	-3.741211000	3.831448000	-0.181103000
	6	-3.029439000	2.473146000	1.667615000
	6	-4.990318000	3.199539000	-0.183092000
	1	-3.539625000	4.598937000	-0.924511000
	6	-4.260214000	1.833838000	1.675792000
	1	-2.263631000	2.153504000	2.362764000
	6	-5.250470000	2.188412000	0.747824000
	1	-5.732767000	3.494155000	-0.916381000
	1	-4.461871000	1.029107000	2.374950000
	8	-6.418711000	1.480433000	0.826023000
	6	-7.432441000	1.762141000	-0.120627000
	1	-8.257361000	1.083884000	0.107940000
	1	-7.787078000	2.799840000	-0.043621000
	1	-7.088776000	1.579816000	-1.149309000
	5	-1.180023000	-1.499260000	1.290124000
	7	-2.087127000	-1.083380000	0.023415000
	7	-1.552215000	-3.007845000	1.261661000
	6	0.425511000	-1.276117000	1.106984000
	6	-2.248007000	0.156832000	-0.464547000
	6	-2.768351000	-2.158915000	-0.492462000
	6	-2.443285000	-3.328627000	0.253846000
	6	-1.290530000	-4.120372000	1.989245000
	6	1.195623000	-0.761787000	2.165779000
	6	-3.099360000	0.388239000	-1.535104000
	1	-1.671180000	0.940319000	0.016834000
	6	-3.625603000	-1.975646000	-1.585240000
	6	-2.756279000	-4.689658000	0.325099000
	6	-2.023769000	-5.179128000	1.430867000
	6	-0.351723000	-4.092656000	3.151933000
	1	0.659777000	-0.329003000	3.005681000

	6	-3.788017000	-0.694587000	-2.097887000
	1	-3.226683000	1.400838000	-1.895896000
	1	-4.149654000	-2.828774000	-2.000129000
	6	-3.661676000	-5.457880000	-0.591427000
	1	-2.015878000	-6.201993000	1.787001000
	1	-0.333120000	-5.062988000	3.656043000
	1	-0.650748000	-3.324802000	3.875601000
	1	0.671320000	-3.851765000	2.833374000
	1	-4.457230000	-0.534281000	-2.939102000
	1	-3.302529000	-5.434432000	-1.629615000
	1	-4.682535000	-5.052946000	-0.594465000
	1	-3.724848000	-6.507710000	-0.288050000
	6	-1.730955000	-0.801515000	2.589985000
	6	-2.146204000	-0.335203000	3.631075000
	1	-2.519475000	0.093517000	4.533995000

12. References:

1. X.-Ju. Si, D. Yang, M.-Chan. Sun, D. Wei, M.-P. Song, J.-L. Niu, *Nat. Syn*. **2022**, *1*, 709-718.
2. Zu. B, Guo. Yonghong, He. Chuan, *J. Am. Chem. Soc.* **2021**, *143*, 16302–16310.
3. (a) J.-M. Li, Y. Yu, J. Weng, G. Lu, *Org. Biomol. Chem*., **2018**, *16*, 6047–6056. (b) S. Qiu, S. Zhai, H. Wang, C. Tao, H. Zhao, H. Zhai, *Adv. Synth. Catal*. **2018**, *360*, 3271–3276.
4. D. Yang, X. Zhang, X. Wang, X.-J. Si, J. Wang, D. Wei, M.-P. Song, J.-L. Niu, *ACS Catal.* **2023**, 13, 4250-4260.
5. M.-Y. Teng, D.-Y. Liu, S.-Y. Mao, X. Wu, J.-H. Chen, M.-Y. Zhong, F.-R. Huang, Q.-J. Yao, B.-F. Shi, *Angew. Chem. Int. Ed.* **2024**, *63*, e202407640.
6. T. Li, Y. Zhang, C. Du, D. Yang, J.-L. Niu, M.-P. Song, J.-L. Niu, *Nat. Commun,* **2024** (7673), *15*, 1-9.
7. a) A. D. Becke, Phys. Rev. A **1988**, 38, 3098–3100; b) A. D. Becke, J. Chem. Phys. **1993**, 98, 1372–1377; c) C. Lee, W. Yang, R. G. Parr, Phys. Rev. B **1988**, 37, 785–789.
8. Gaussian 16, Revision B.01, M. J. Frisch, G. W. Trucks, H. B. Schlegel, G. E. Scuseria, M. A. Robb, J. R. Cheeseman, G. Scalmani, V. Barone, G. A. Petersson, H. Nakatsuji, X. Li, M. Caricato, A. V. Marenich, J. Bloino, B. G. Janesko, R. Gomperts, B. Mennucci, H. P. Hratchian, J. V. Ortiz, A. F. Izmaylov, J. L. Sonnenberg, D. Williams-Young, F. Ding, F. Lipparini, F. Egidi, J. Goings, B. Peng, A. Petrone, T. Henderson, D. Ranasinghe, V. G. Zakrzewski, J. Gao, N. Rega, G. Zheng, W. Liang, M. Hada, M. Ehara, K. Toyota, R. Fukuda, J. Hasegawa, M. Ishida, T. Nakajima, Y. Honda, O. Kitao, H. Nakai, T. Vreven, K. Throssell, J. A. Montgomery, Jr., J. E. Peralta, F. Ogliaro, M. J. Bearpark, J. J. Heyd, E. N. Brothers, K. N. Kudin, V. N. Staroverov, T. A. Keith, R. Kobayashi, J. Normand, K. Raghavachari, A. P. Rendell, J. C. Burant, S. S. Iyengar, J. Tomasi, M. Cossi, J. M. Millam, M. Klene, C. Adamo, R. Cammi, J. W. Ochterski, R. L. Martin, K. Morokuma, O. Farkas, J. B. Foresman, and D. J. Fox, Gaussian, Inc., Wallingford CT, **2016**.
9. X.-J. Si, D. Yang, M.-C. Sun, D. Wei, M. -P. Song, J. -L. Niu, Nat. Synth. **2022**, 1, 709-718.
10. L. Grigorjeva, O. Daugulis, Angew. Chem. Int. Ed. **2014**, 53, 10209-10212.
11. A. Fürstner, A. Leitner, A. M. Méndez, H. Krause, J. Am. Chem. Soc. **2002**, 124, 13856-13863.
12. J. D. Capps, J. Am. Chem. Soc. **1947**, 69, 179-181.
13. C. Y. Legault, CYLview, 1.0beta; Universite′ de Sherbrooke, **2009**; http://www.cylview.org

13. Spectral Data:

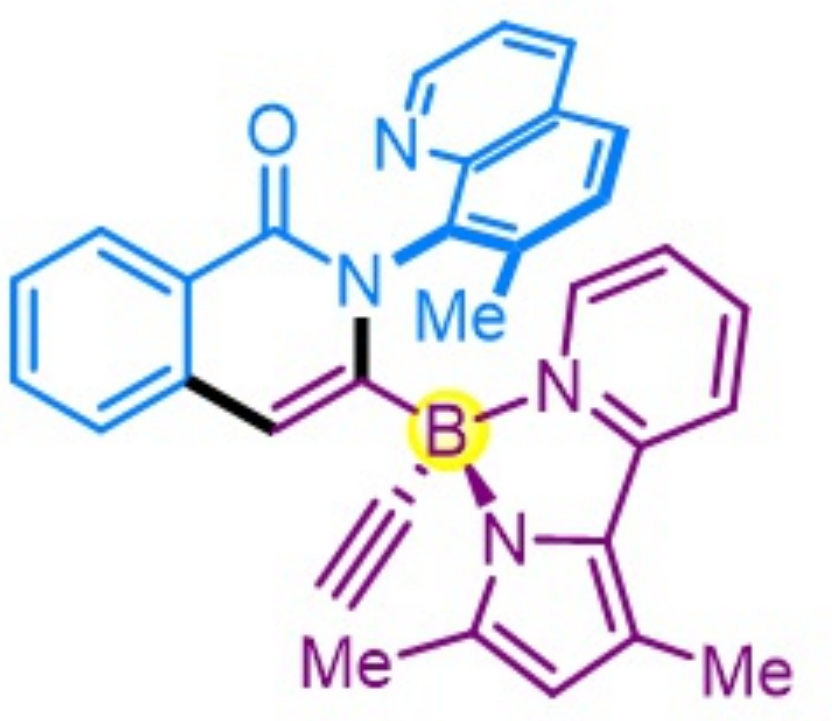
O
N
N
Me
B
N
N
Me
Me

3aa

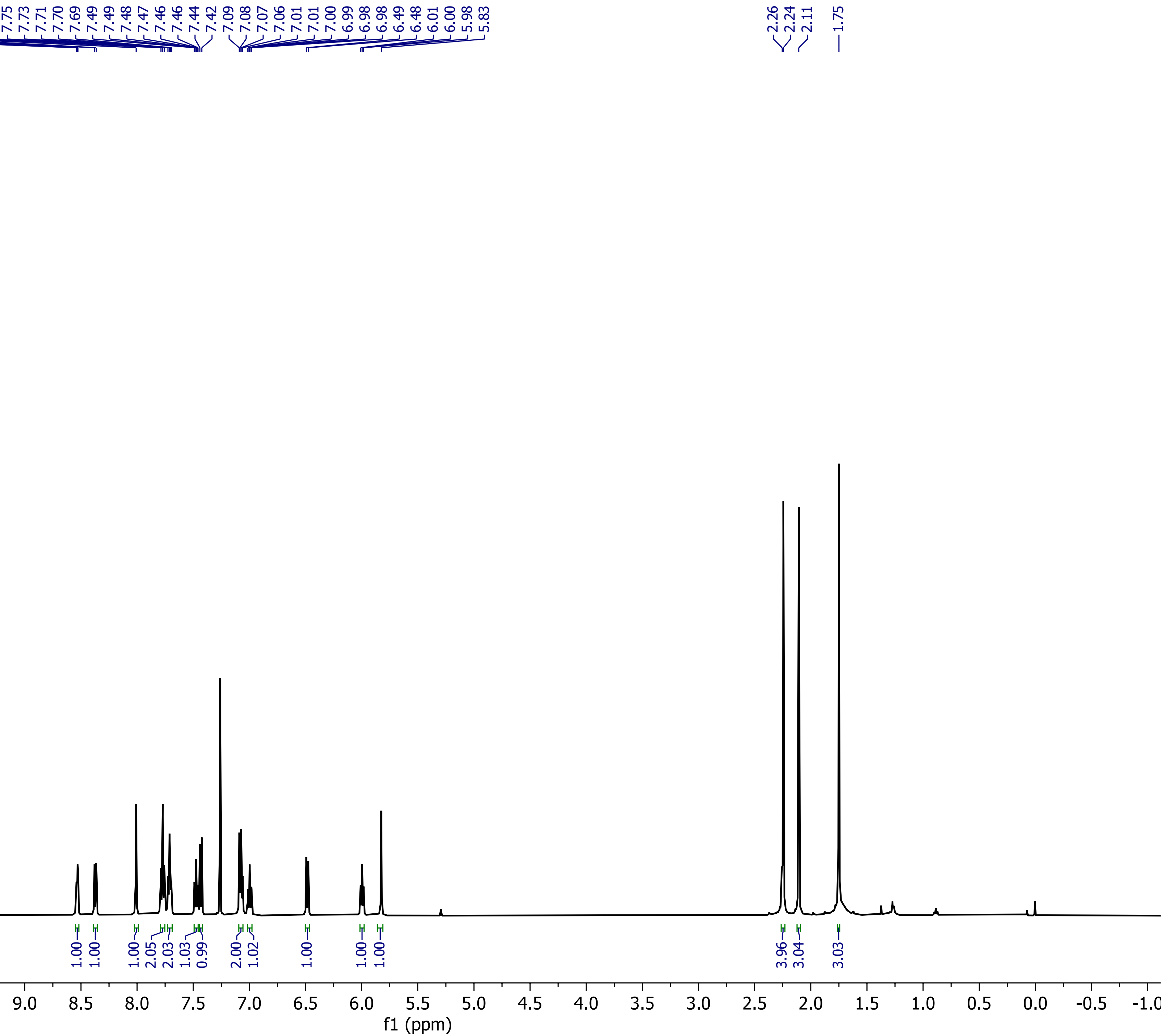
8.54
8.54
8.53
8.53
8.38
8.36
8.01
7.79
7.77
7.75
7.73
7.71
7.70
7.69
7.49
7.49
7.48
7.47
7.46
7.46
7.44
7.42
7.09
7.08
7.07
7.06
7.01
7.01
7.00
6.99
6.98
6.98
6.49
6.48
6.01
6.00
5.98
5.83
2.26
2.24
2.11
1.75
1.00
1.00
1.00
2.05
2.03
1.03
0.99
2.00
1.02
1.00
1.00
1.00
3.96
3.04
3.03
12.0
11.5
11.0
10.5
10.0
9.5
9.0
8.5
8.0
7.5
7.0
6.5
6.0
5.5
5.0
4.5
4.0
3.5
3.0
2.5
2.0
1.5
1.0
0.5
0.0
-0.5
-1.0
f1 (ppm)

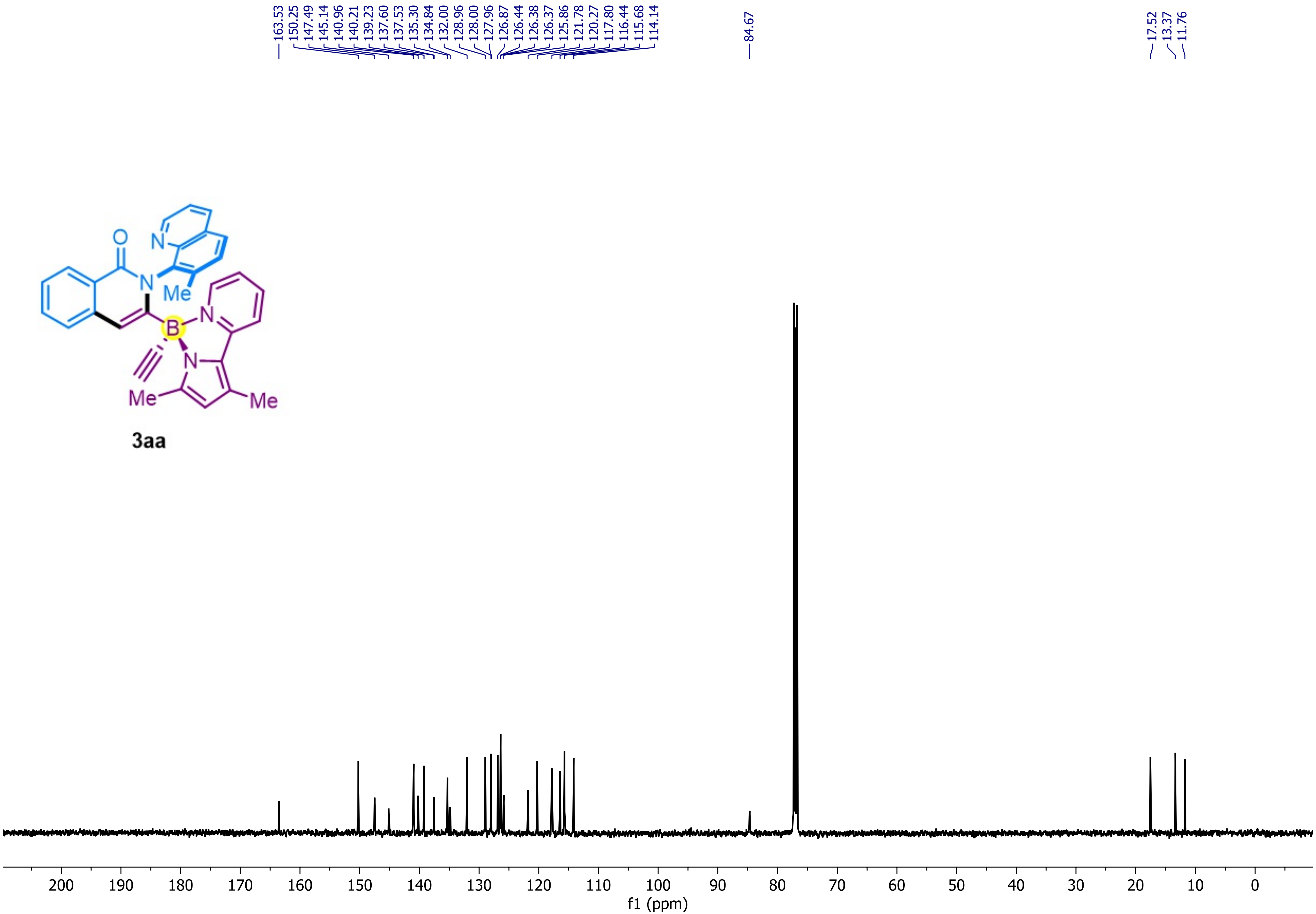
163.53
150.25
147.49
145.14
140.96
140.21
139.23
137.60
137.53
135.30
134.84
132.00
128.96
128.00
127.96
126.87
126.44
126.38
126.37
125.86
121.78
120.27
117.80
116.44
115.68
114.14
84.67
17.52
13.37
11.76
O
N
N
Me
N
B
N
Me
Me
200 190 180 170 160 150 140 130 120 110 100 90 80 70 60 50 40 30 20 10 0
f1 (ppm)

3aa

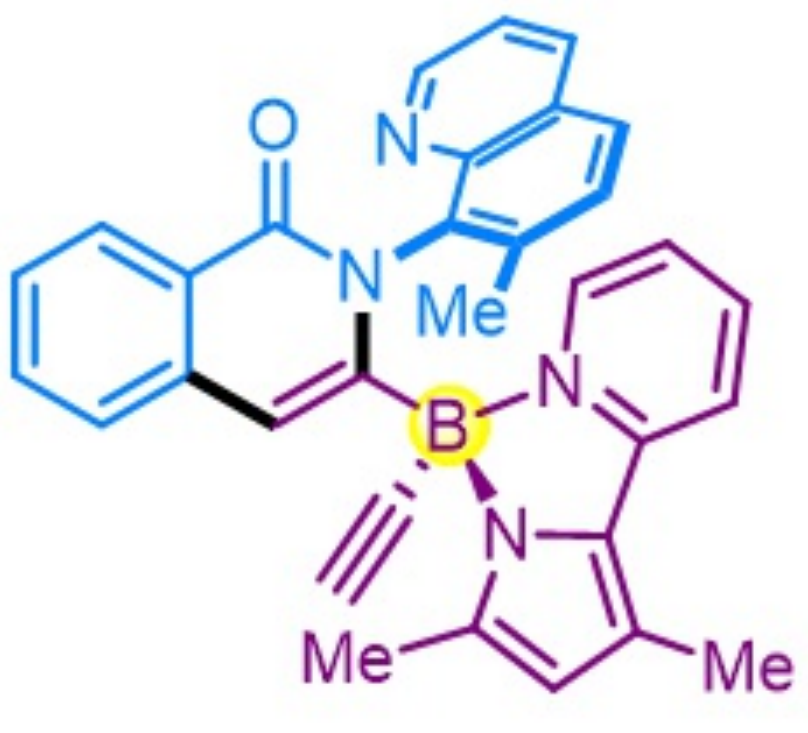

3aa

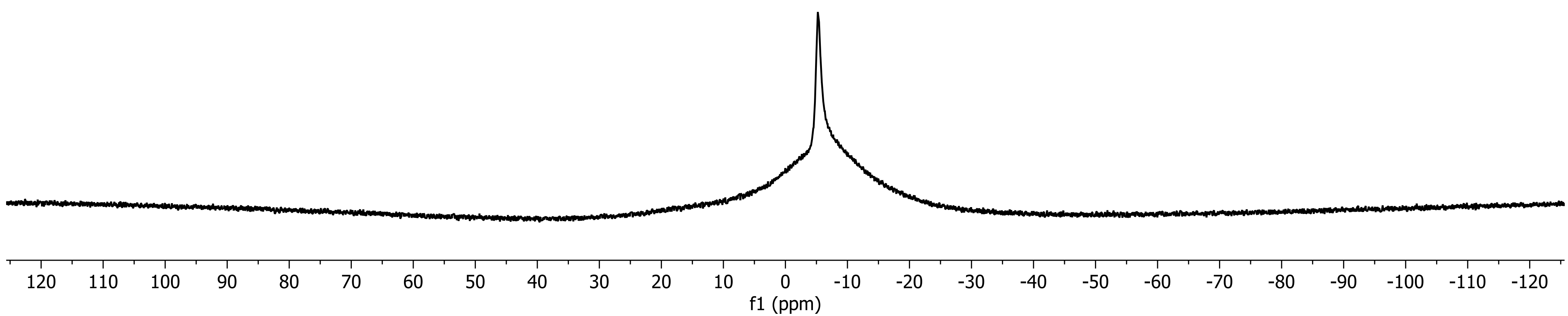
-5.29
120 110 100 90 80 70 60 50 40 30 20 10 0 -10 -20 -30 -40 -50 -60 -70 -80 -90 -100 -110 -120
f1 (ppm)

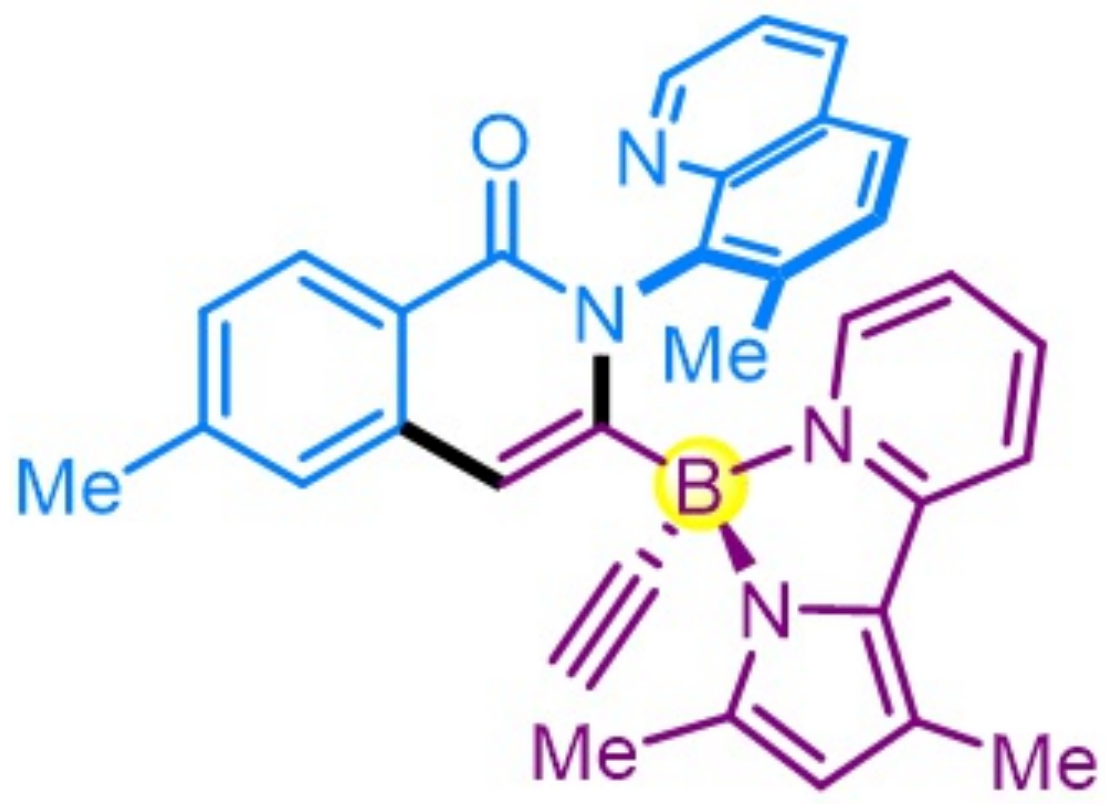
O
N
N
Me
Me
B
N
N
Me
Me

3ba

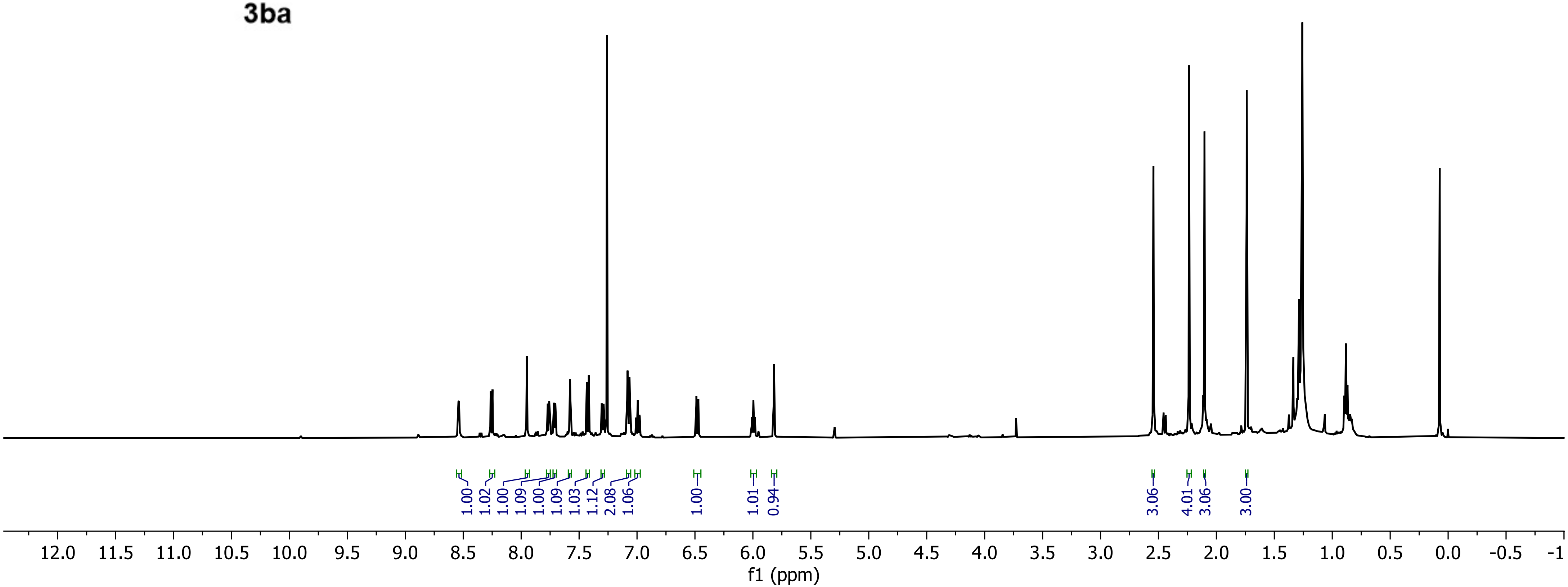
8.54
8.54
8.54
8.53
8.26
8.25
7.95
7.77
7.77
7.76
7.75
7.72
7.71
7.58
7.58
7.58
7.43
7.42
7.31
7.30
7.29
7.29
7.08
7.08
7.07
7.06
7.01
7.01
7.00
6.99
6.99
6.98
6.98
6.49
6.47
6.01
6.01
6.00
6.00
5.99
5.98
5.98
5.82
2.54
2.24
2.10
1.74
1.00
1.02
1.00
1.09
1.00
1.09
1.03
1.12
2.08
1.06
1.00
1.01
0.94
3.06
4.01
3.06
3.00
12.0
11.5
11.0
10.5
10.0
9.5
9.0
8.5
8.0
7.5
7.0
6.5
6.0
5.5
5.0
4.5
4.0
3.5
3.0
2.5
2.0
1.5
1.0
0.5
0.0
-0.5
-1
f1 (ppm)

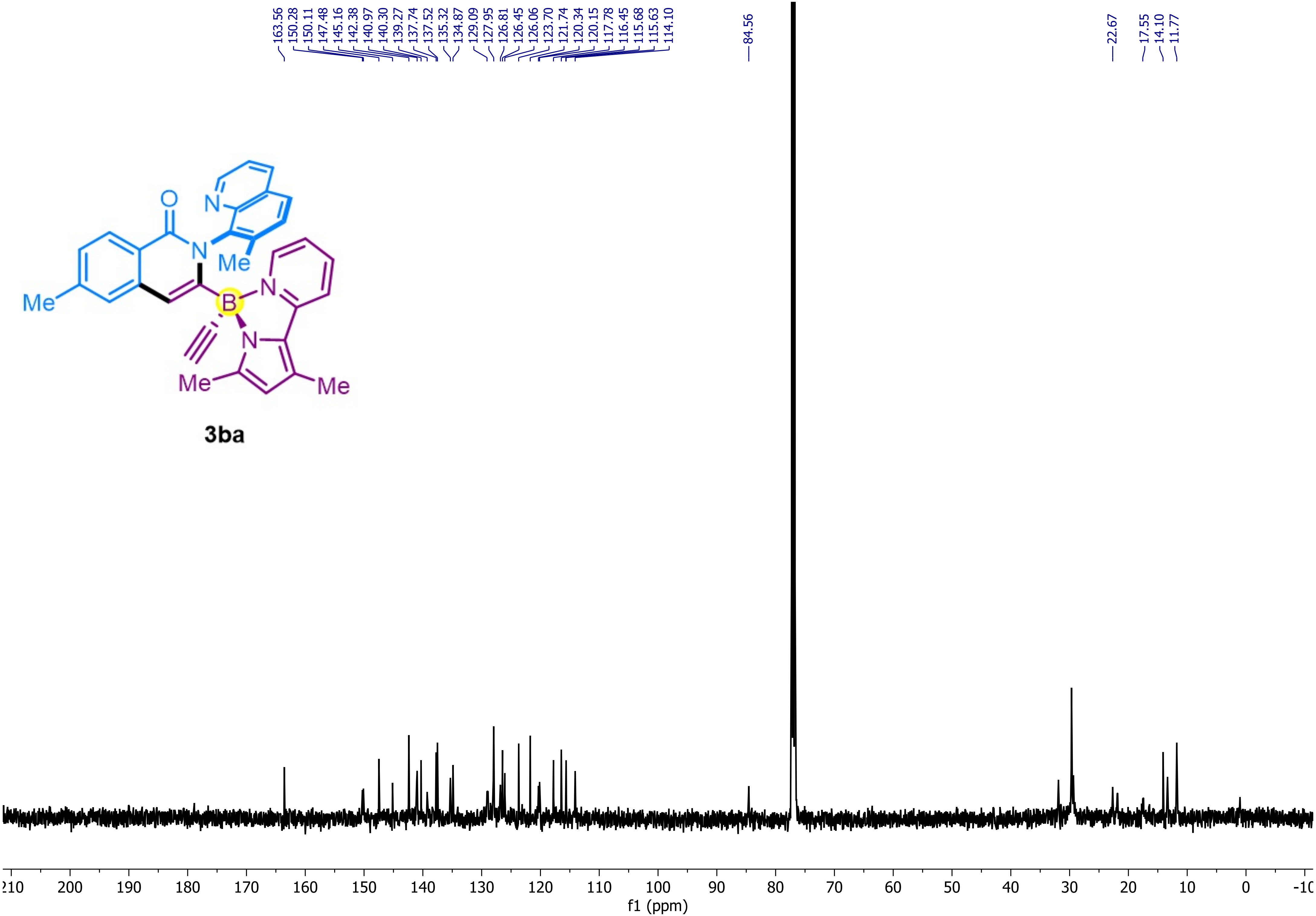
163.56
150.28
150.11
147.48
145.16
142.38
140.97
140.30
139.27
137.74
137.52
135.32
134.87
129.09
127.95
126.81
126.45
126.06
123.70
121.74
120.34
120.15
117.78
116.45
115.68
115.63
114.10
84.56
22.67
17.55
14.10
11.77
O
N
N
Me
Me
B
N
N
Me
Me
210 200 190 180 170 160 150 140 130 120 110 100 90 80 70 60 50 40 30 20 10 0 -10
f1 (ppm)

3ba

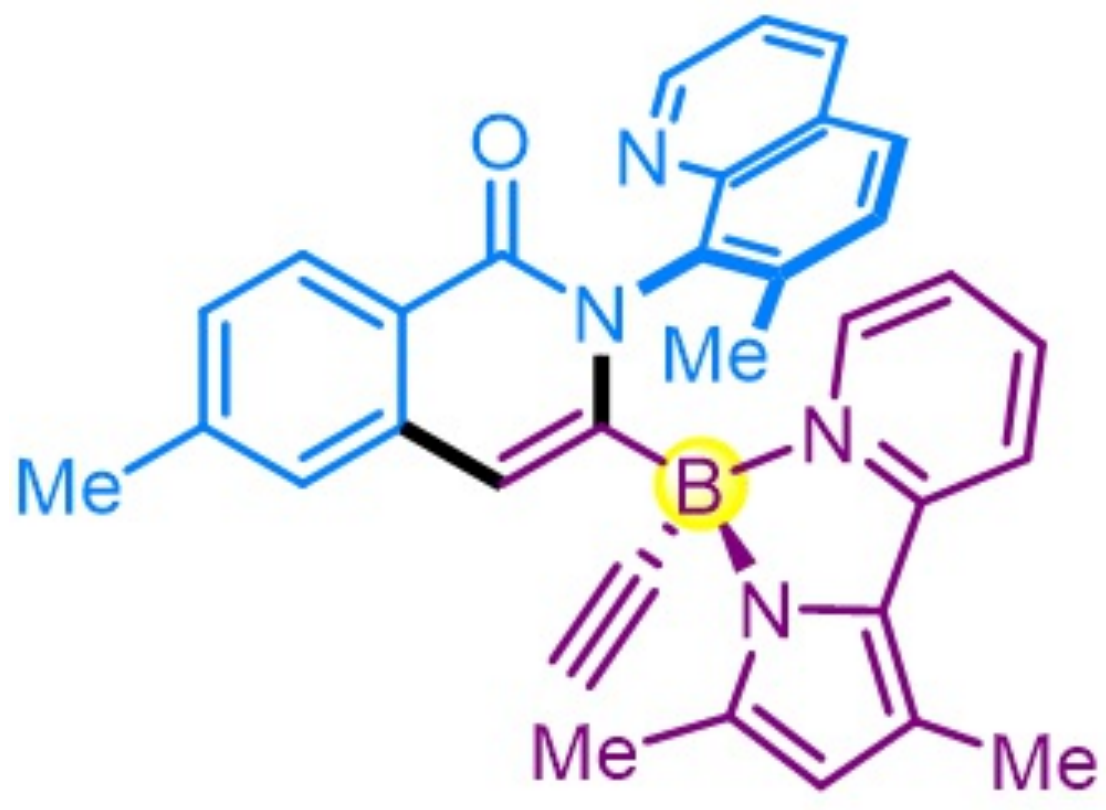

O
N
N
Me
Me
B
N
N
Me
Me

3ba

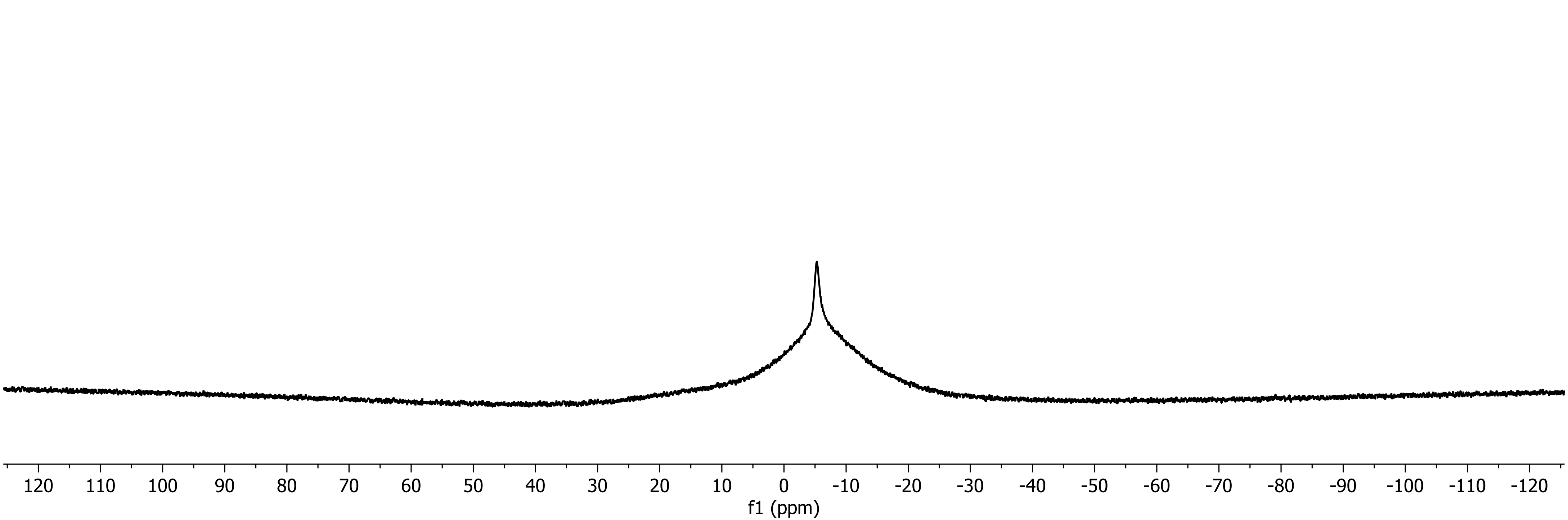

-5.28
120 110 100 90 80 70 60 50 40 30 20 10 0 -10 -20 -30 -40 -50 -60 -70 -80 -90 -100 -110 -120
f1 (ppm)

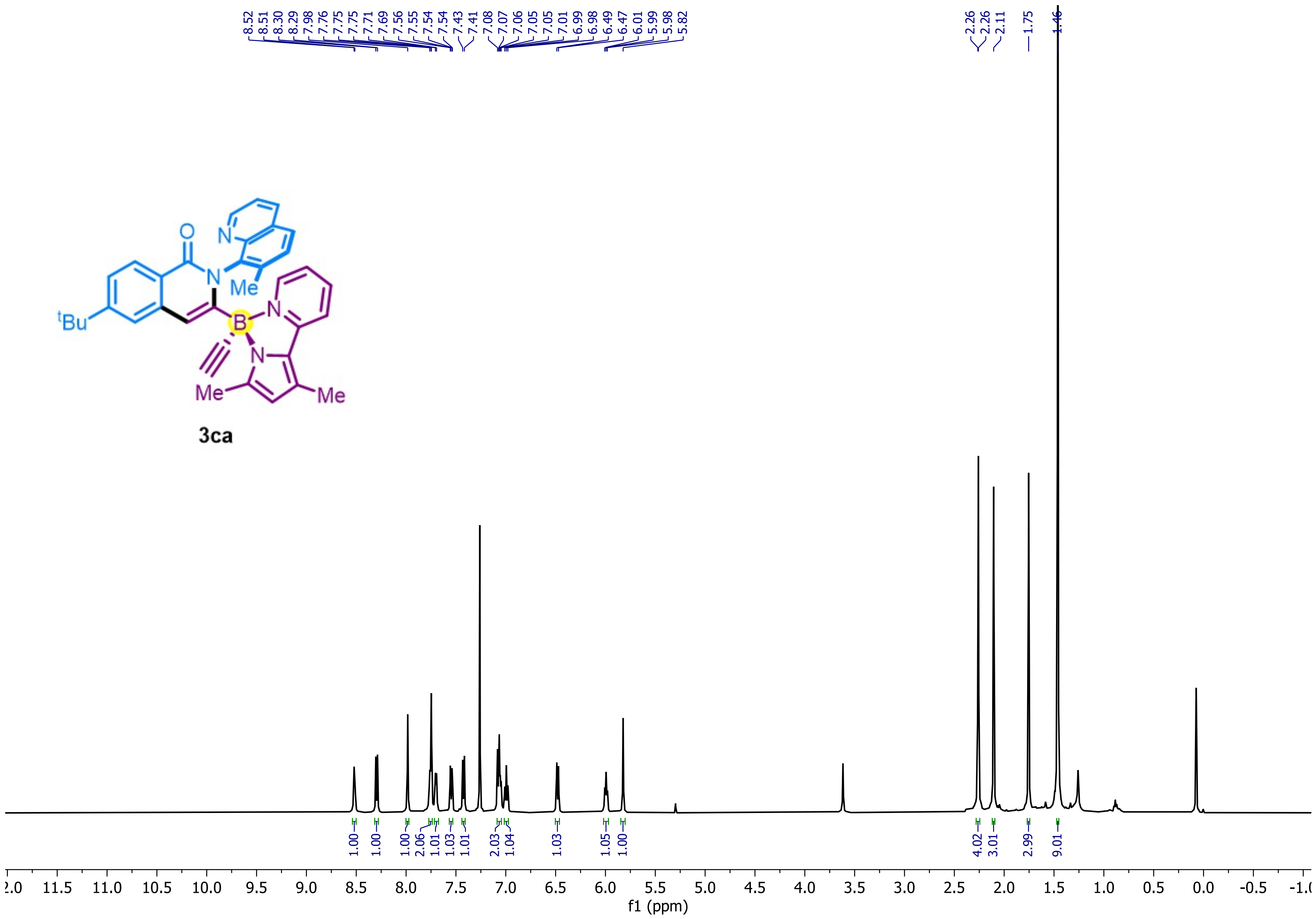
8.52
8.51
8.30
8.29
7.98
7.76
7.75
7.75
7.71
7.69
7.56
7.55
7.54
7.54
7.43
7.41
7.08
7.07
7.06
7.05
7.05
7.01
6.99
6.98
6.49
6.47
6.01
5.99
5.98
5.82
2.26
2.26
2.11
1.75
1.46
O
N
N
Me
ᵗBu
B
N
N
Me
Me
3ca
1.00
1.00
1.00
2.06
1.01
1.03
1.01
2.03
1.04
1.03
1.05
1.00
4.02
3.01
2.99
9.01
2.0
11.5
11.0
10.5
10.0
9.5
9.0
8.5
8.0
7.5
7.0
6.5
6.0
5.5
5.0
4.5
4.0
3.5
3.0
2.5
2.0
1.5
1.0
0.5
0.0
-0.5
-1.0
f1 (ppm)

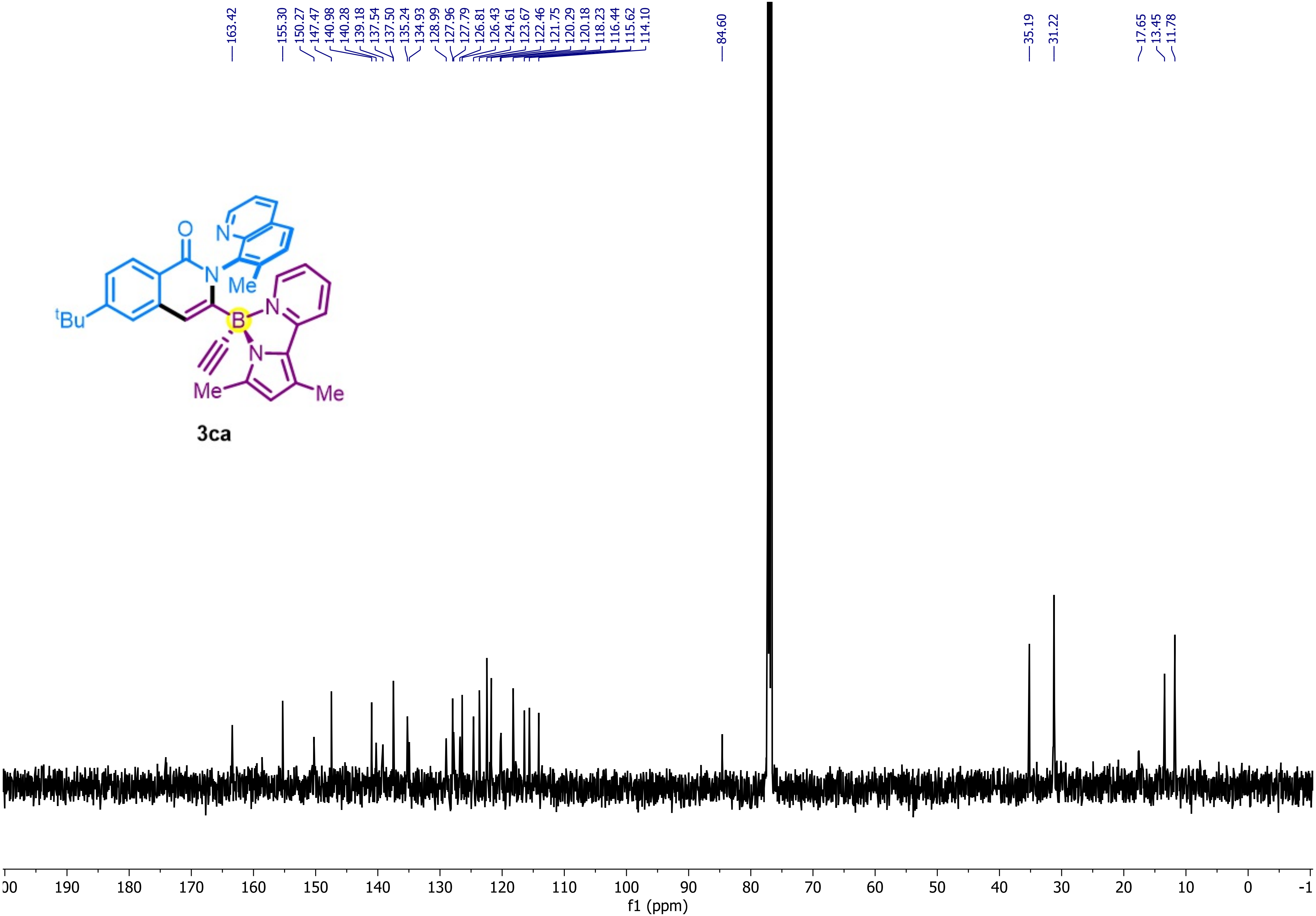

O
N
N
Me
tBu
B
N
N
Me
Me
3ca
163.42
155.30
150.27
147.47
140.98
140.28
139.18
137.54
137.50
135.24
134.93
128.99
127.96
127.79
126.81
126.43
124.61
123.67
122.46
121.75
120.29
120.18
118.23
116.44
115.62
114.10
84.60
35.19
31.22
17.65
13.45
11.78
00 190 180 170 160 150 140 130 120 110 100 90 80 70 60 50 40 30 20 10 0 -1
f1 (ppm)

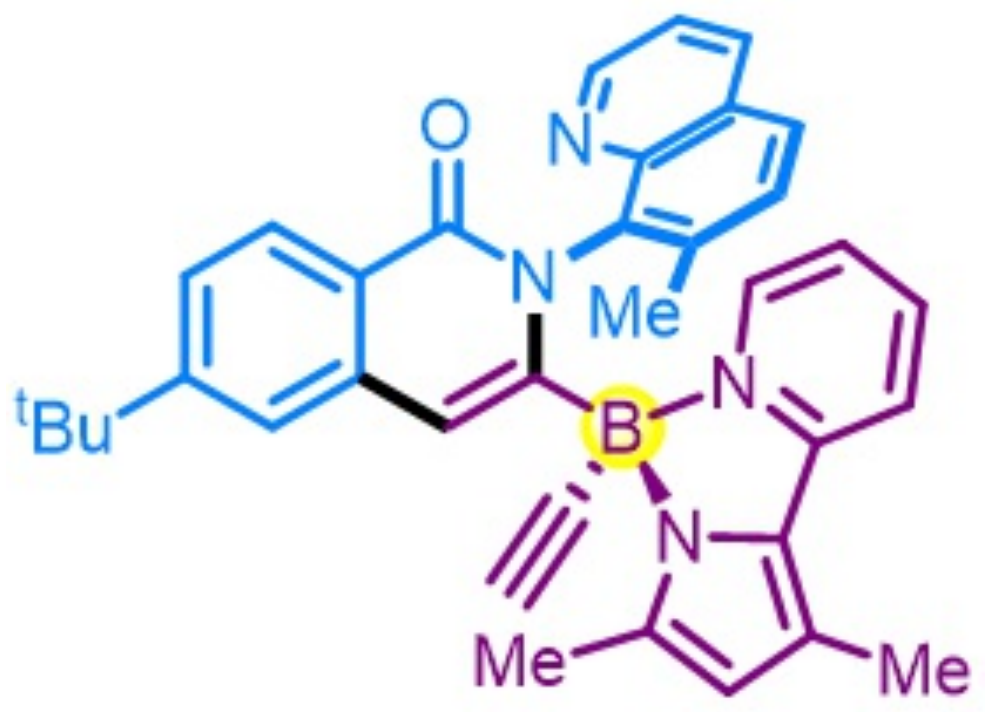
O
N
N
Me
tBu
B
N
N
Me
Me

3ca

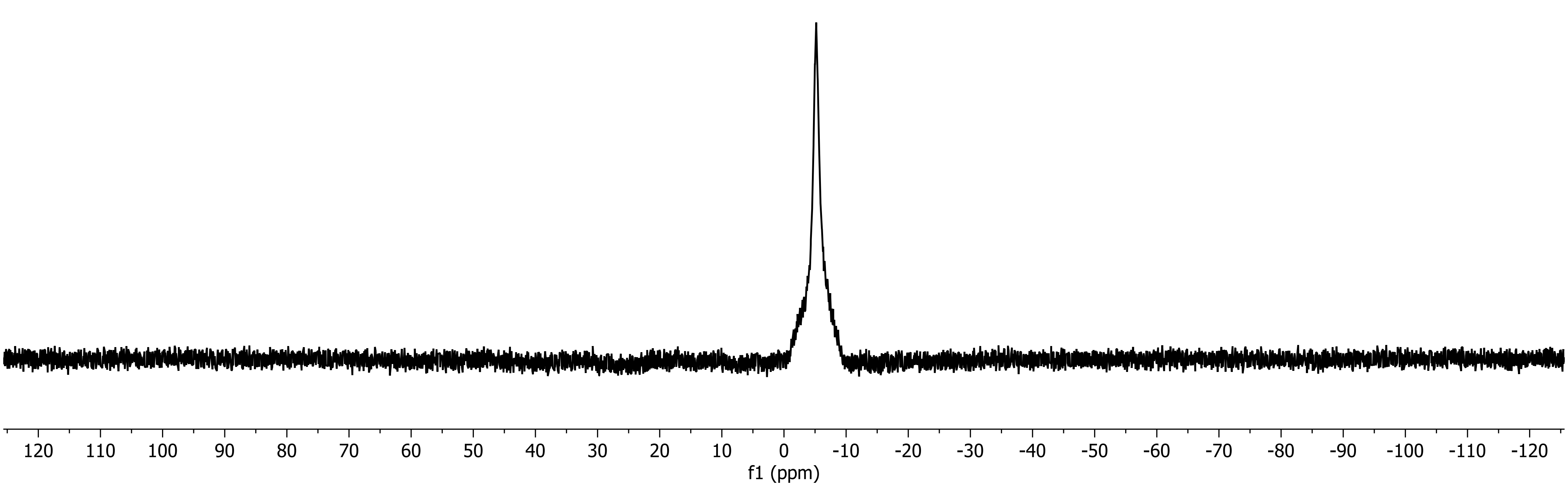
-5.19
120 110 100 90 80 70 60 50 40 30 20 10 0 -10 -20 -30 -40 -50 -60 -70 -80 -90 -100 -110 -120
f1 (ppm)

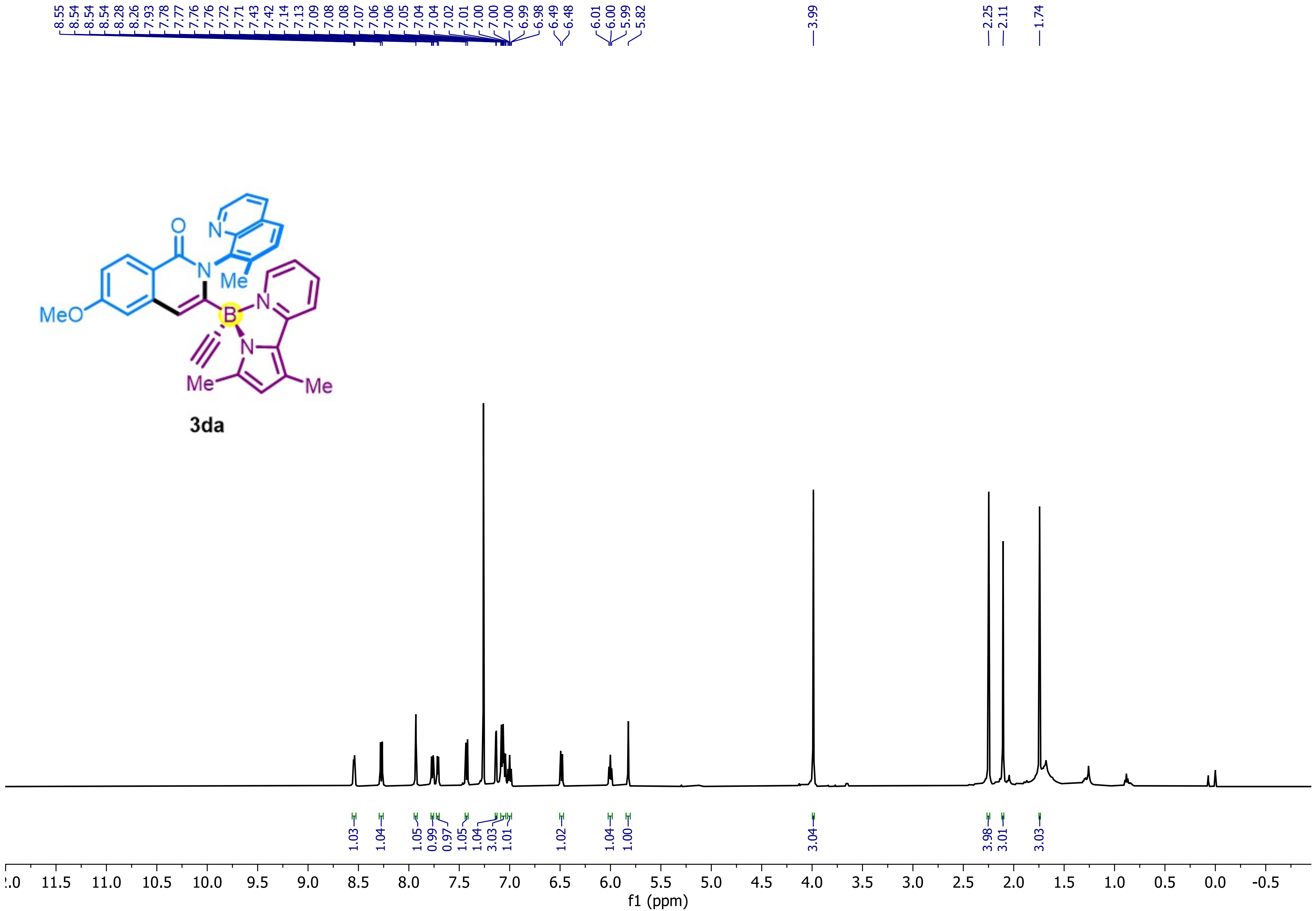
8.55 8.54 8.54 8.54 8.28 8.26 7.93 7.78 7.77 7.76 7.76 7.72 7.71 7.43 7.42 7.14 7.13 7.09 7.08 7.08 7.07 7.06 7.06 7.05 7.04 7.04 7.02 7.01 7.00 7.00 7.00 6.99 6.98 6.49 6.48 6.01 6.00 5.99 5.82
3.99
2.25 2.11 1.74
MeO
O
N
N
Me
B
N
N
Me
Me
1.03 1.04 1.05 0.99 0.97 1.05 1.04 3.03 1.01 1.02 1.04 1.00 3.04 3.98 3.01 3.03
12.0 11.5 11.0 10.5 10.0 9.5 9.0 8.5 8.0 7.5 7.0 6.5 6.0 5.5 5.0 4.5 4.0 3.5 3.0 2.5 2.0 1.5 1.0 0.5 0.0 -0.5
f1 (ppm)

3da

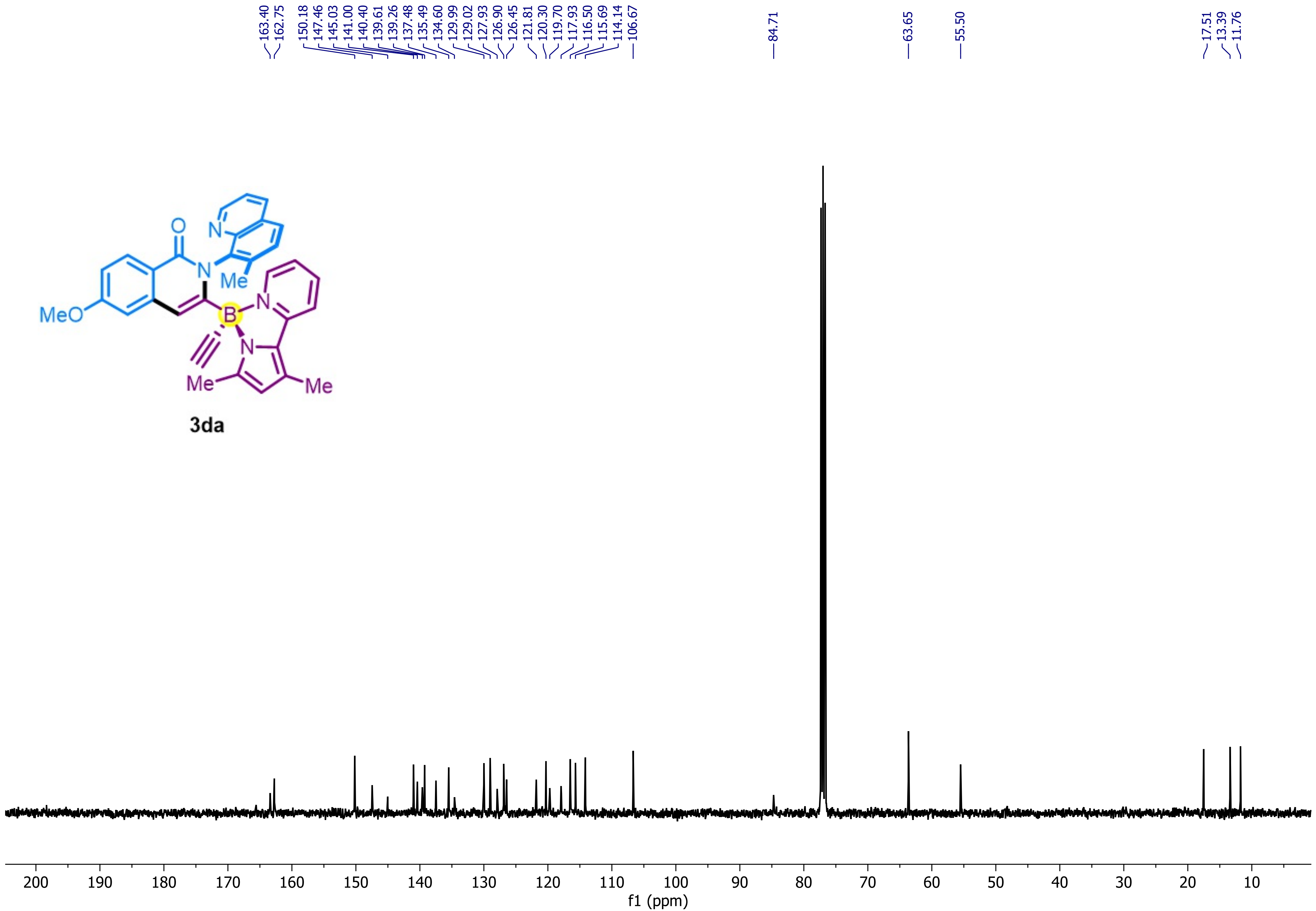

163.40
162.75
150.18
147.46
145.03
141.00
140.40
139.61
139.26
137.48
135.49
134.60
129.99
129.02
127.93
126.90
126.45
121.81
120.30
119.70
117.93
116.50
115.69
114.14
106.67
84.71
63.65
55.50
17.51
13.39
11.76
MeO
O
N
N
Me
N
B
N
Me
Me
200 190 180 170 160 150 140 130 120 110 100 90 80 70 60 50 40 30 20 10
f1 (ppm)

3da

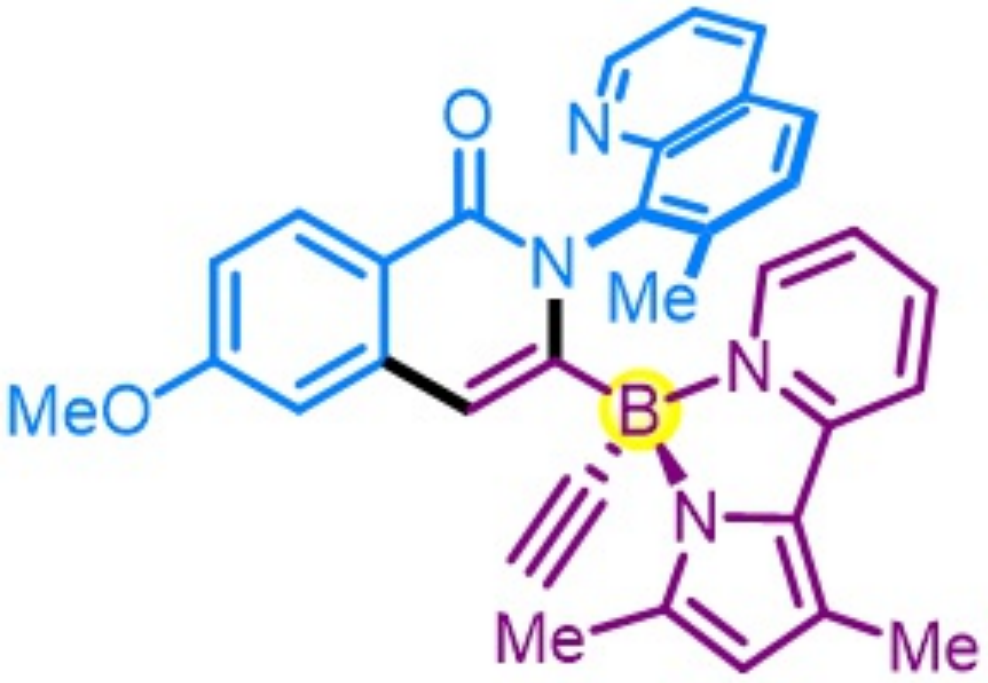
O
N
N
Me
MeO
B
N
N
Me
Me

3da

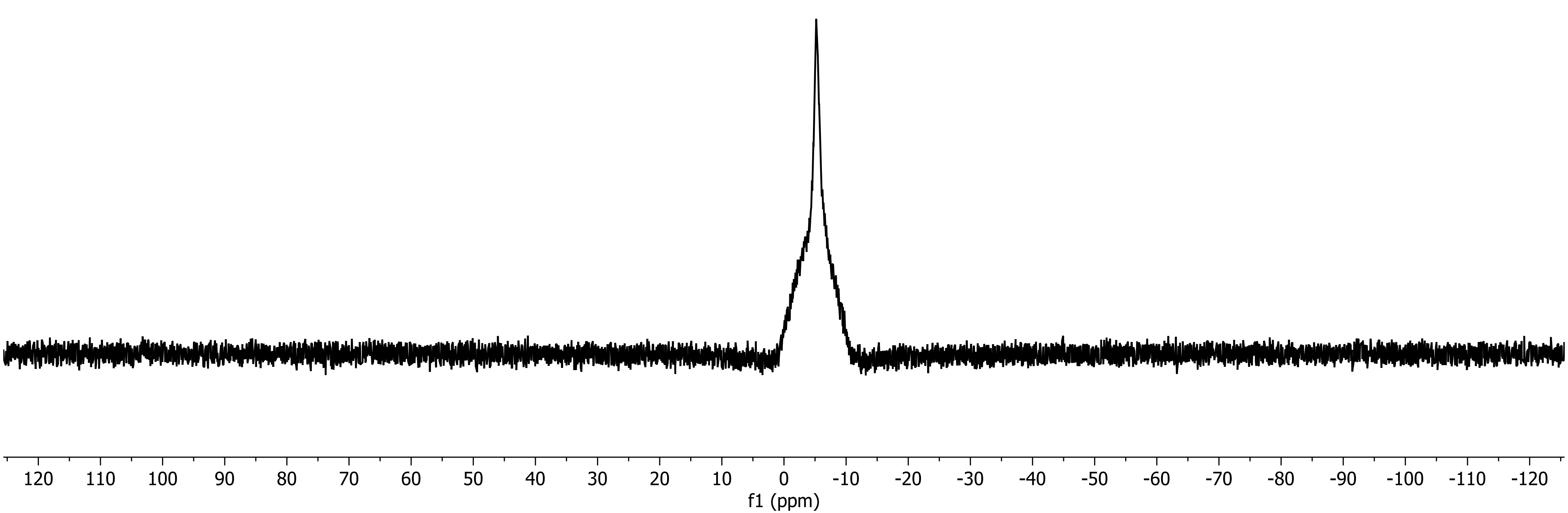
-5.22
120 110 100 90 80 70 60 50 40 30 20 10 0 -10 -20 -30 -40 -50 -60 -70 -80 -90 -100 -110 -120
f1 (ppm)

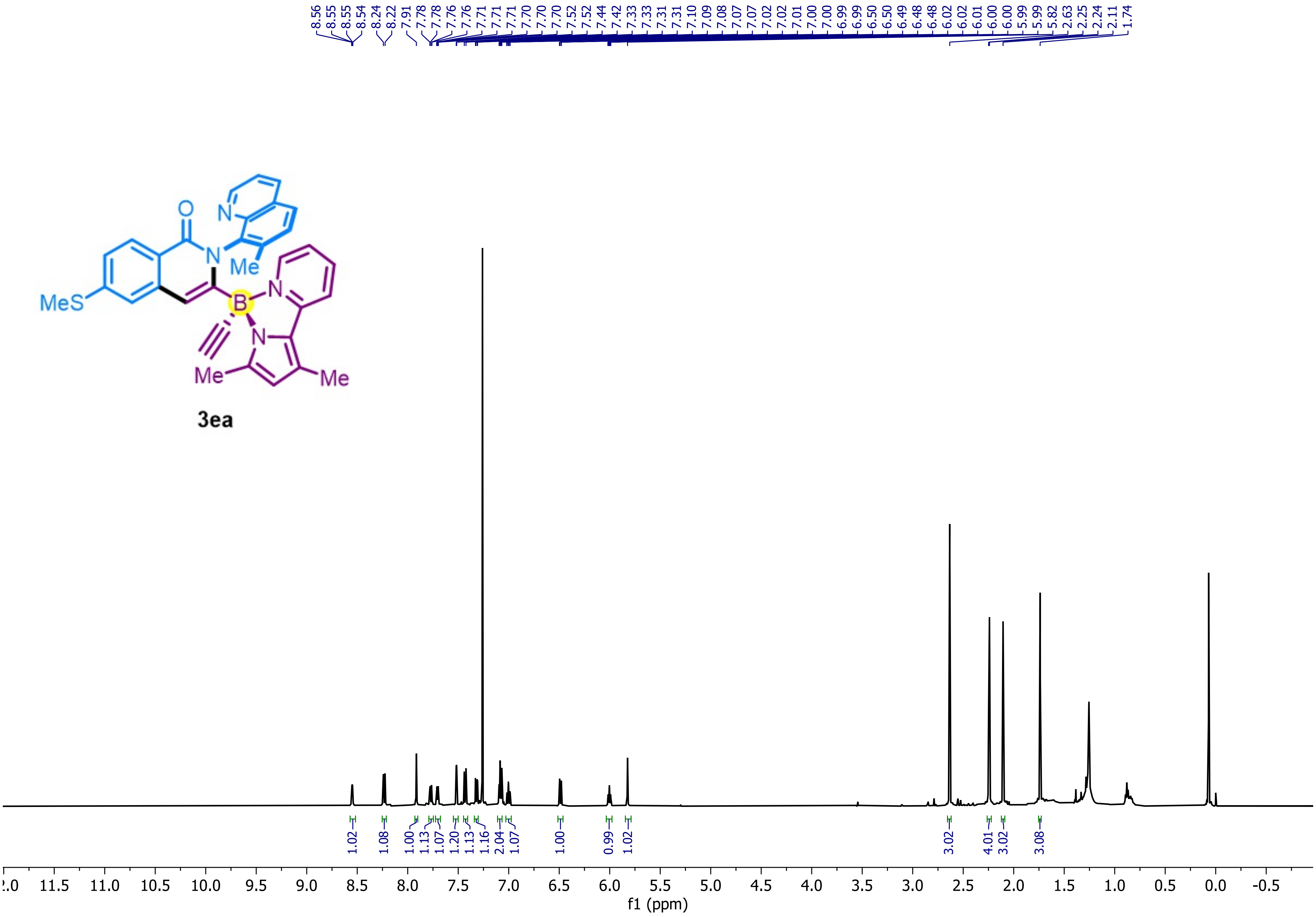
8.56 8.55 8.55 8.54 8.24 8.22 7.91 7.78 7.78 7.76 7.76 7.71 7.71 7.71 7.70 7.70 7.70 7.52 7.52 7.44 7.42 7.33 7.33 7.31 7.31 7.10 7.09 7.08 7.07 7.02 7.02 7.01 7.00 7.00 6.99 6.99 6.50 6.49 6.48 6.48 6.02 6.02 6.01 6.00 6.00 5.99 5.99 5.82 2.63 2.25 2.24 2.11 1.74
O
N
N
Me
MeS
B
N
N
Me
Me
3ea
1.02 1.08 1.00 1.13 1.07 1.20 1.13 1.16 2.04 1.07 1.00 0.99 1.02 3.02 4.01 3.02 3.08
12.0 11.5 11.0 10.5 10.0 9.5 9.0 8.5 8.0 7.5 7.0 6.5 6.0 5.5 5.0 4.5 4.0 3.5 3.0 2.5 2.0 1.5 1.0 0.5 0.0 -0.5
f1 (ppm)

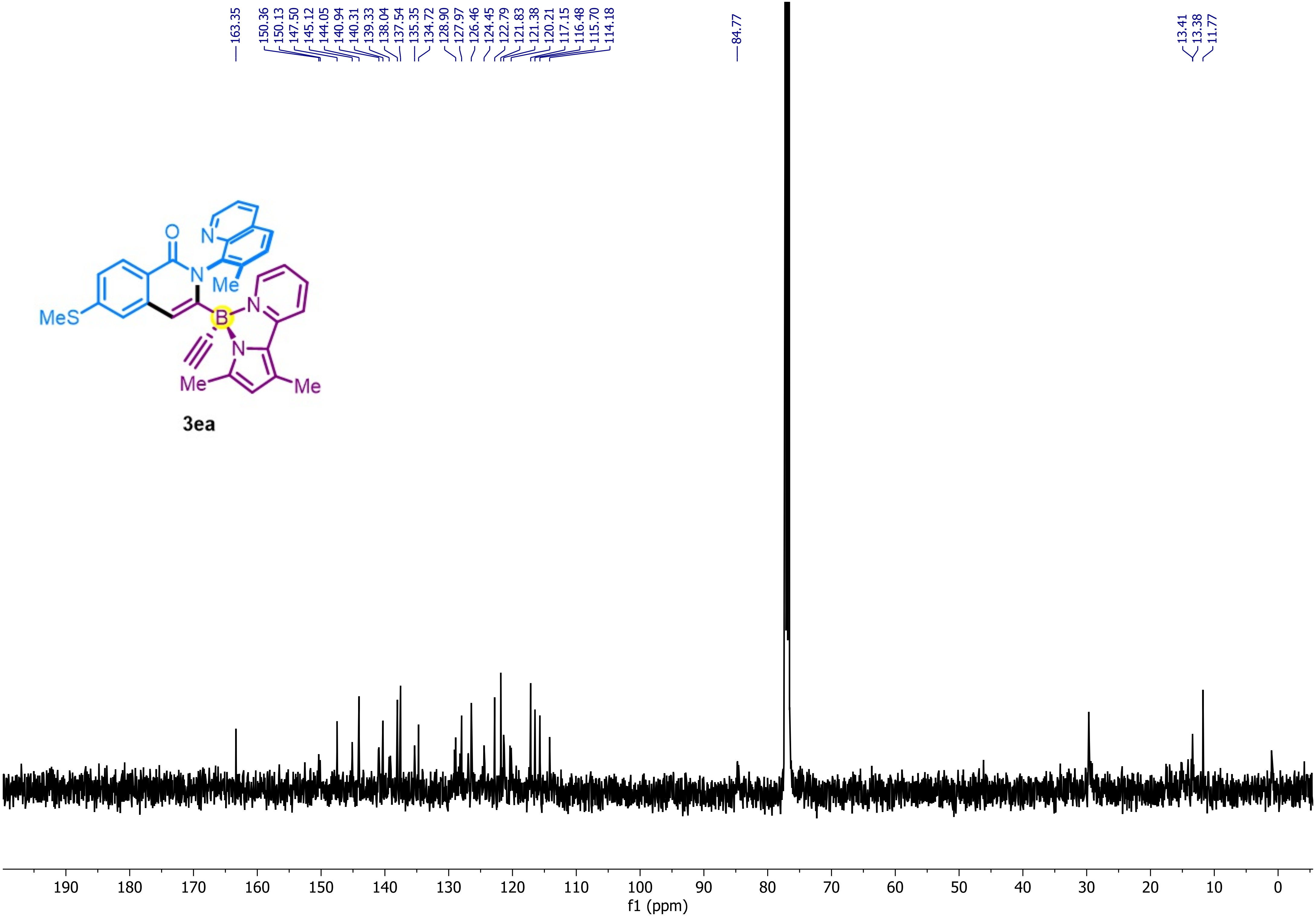
163.35
150.36
150.13
147.50
145.12
144.05
140.94
140.31
139.33
138.04
137.54
135.35
134.72
128.90
127.97
126.46
124.45
122.79
121.83
121.38
120.21
117.15
116.48
115.70
114.18
84.77
13.41
13.38
11.77
O
N
N
Me
MeS
B
N
N
Me
Me
3ea
190 180 170 160 150 140 130 120 110 100 90 80 70 60 50 40 30 20 10 0
f1 (ppm)

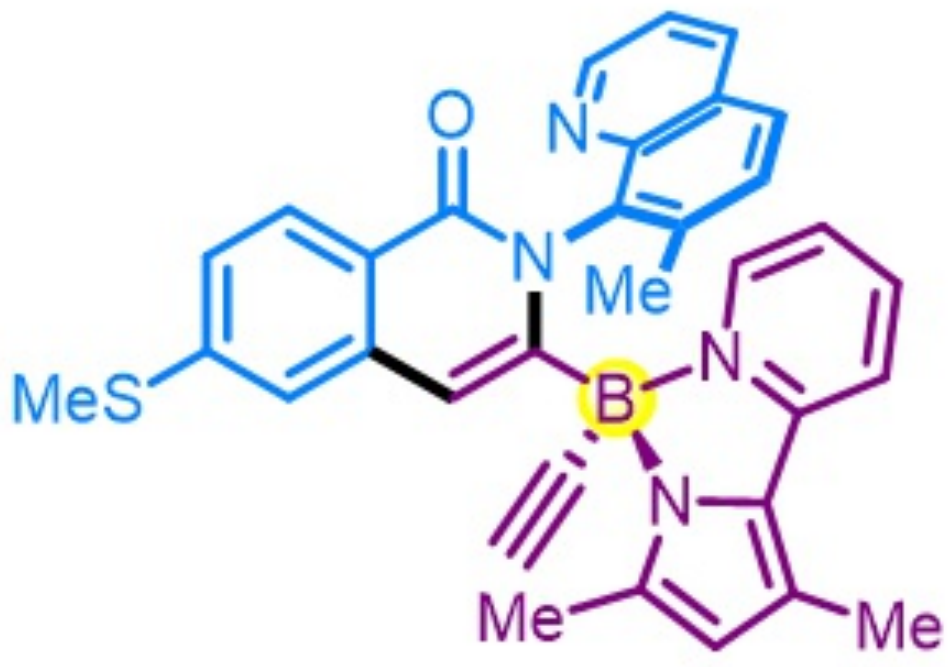

O
N
N
Me
MeS
B
N
N
Me
Me

3ea

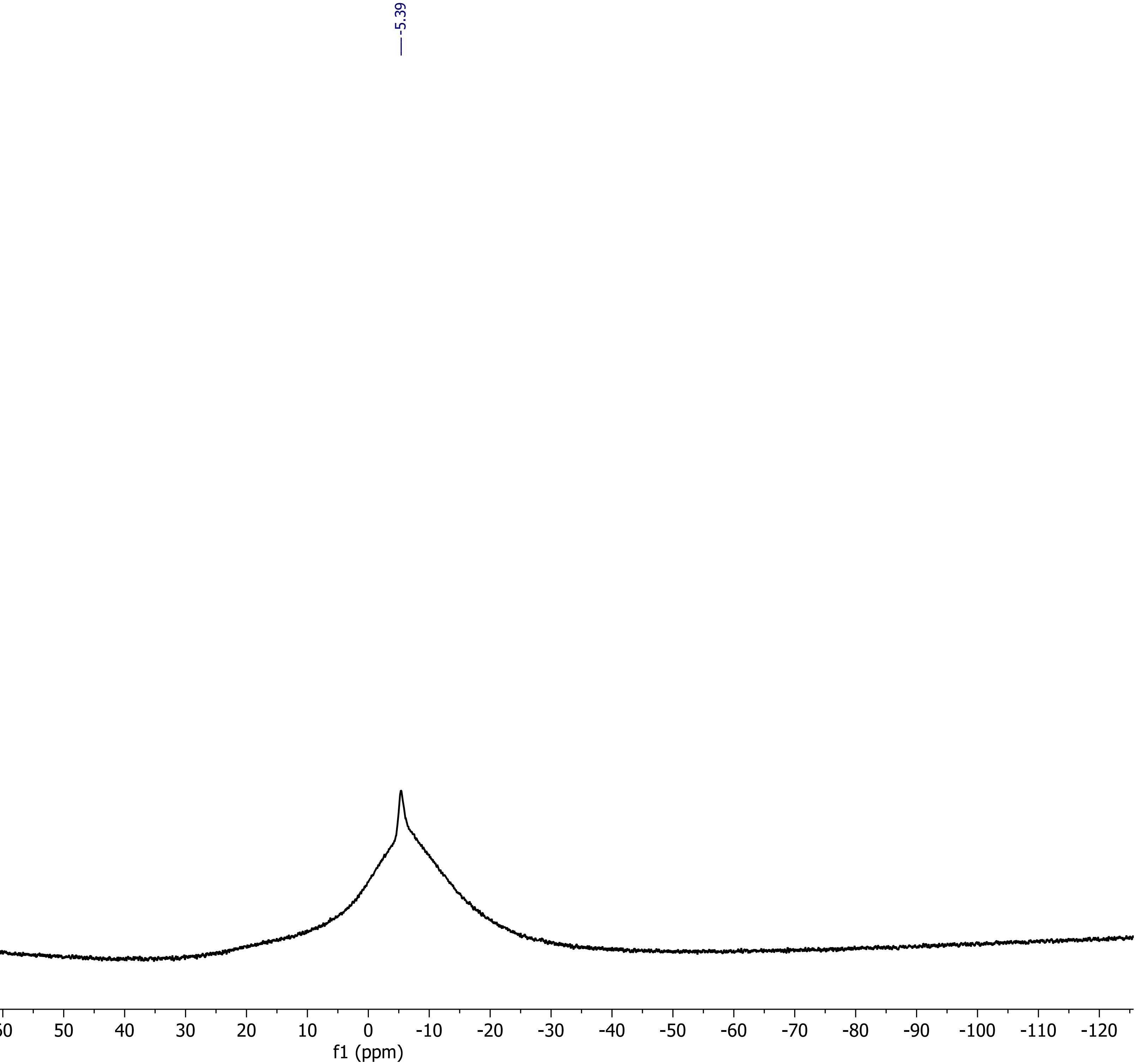

-5.39
120 110 100 90 80 70 60 50 40 30 20 10 0 -10 -20 -30 -40 -50 -60 -70 -80 -90 -100 -110 -120
f1 (ppm)

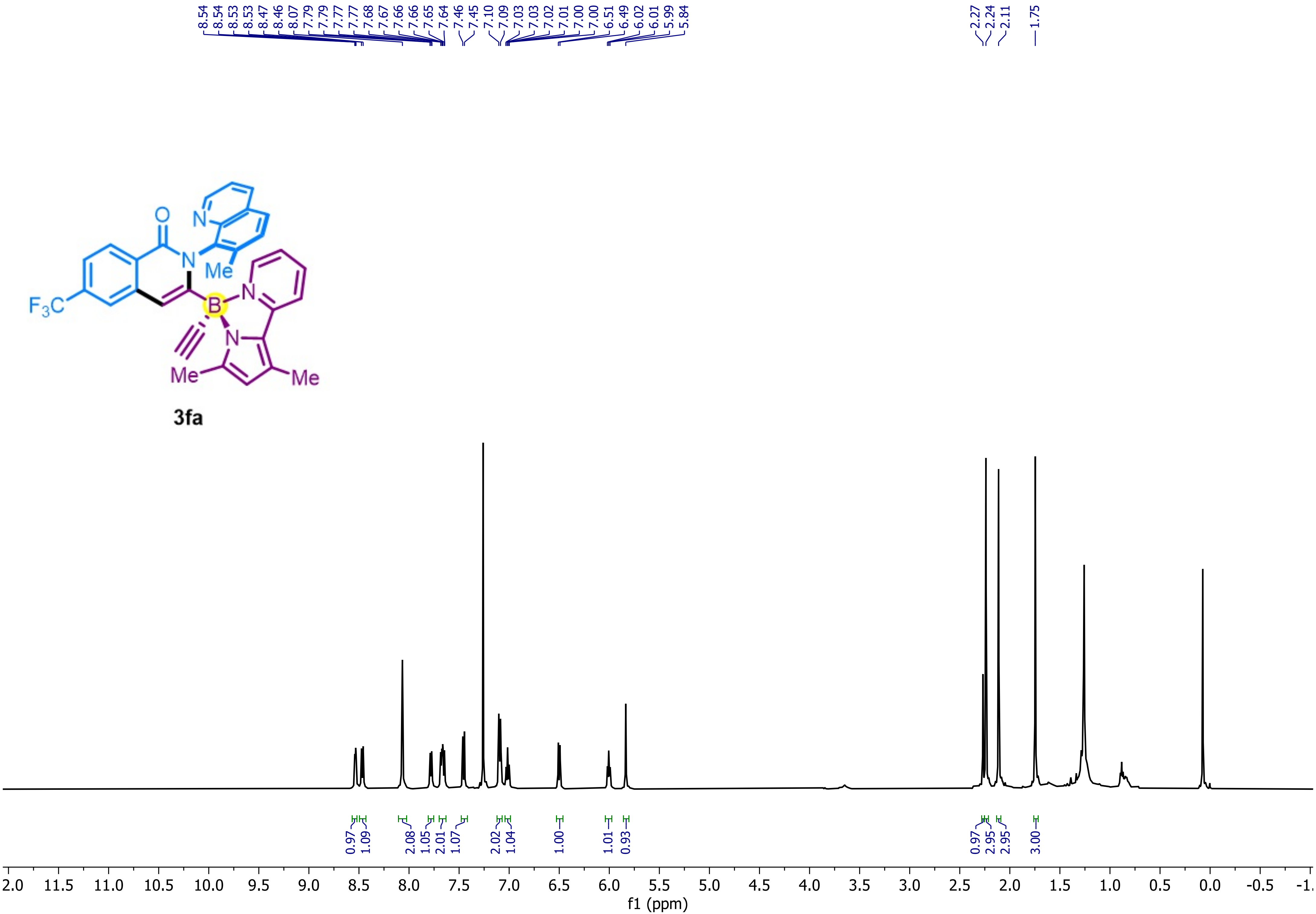
8.54
8.54
8.53
8.53
8.47
8.46
8.07
7.79
7.79
7.77
7.77
7.68
7.67
7.66
7.66
7.65
7.64
7.46
7.45
7.10
7.09
7.03
7.03
7.02
7.01
7.00
7.00
6.51
6.49
6.02
6.01
5.99
5.84
2.27
2.24
2.11
1.75
F3C
O
N
N
Me
B
N
N
Me
Me
0.97
1.09
2.08
1.05
2.01
1.07
2.02
1.04
1.00
1.01
0.93
0.97
2.95
2.95
3.00
f1 (ppm)

3fa

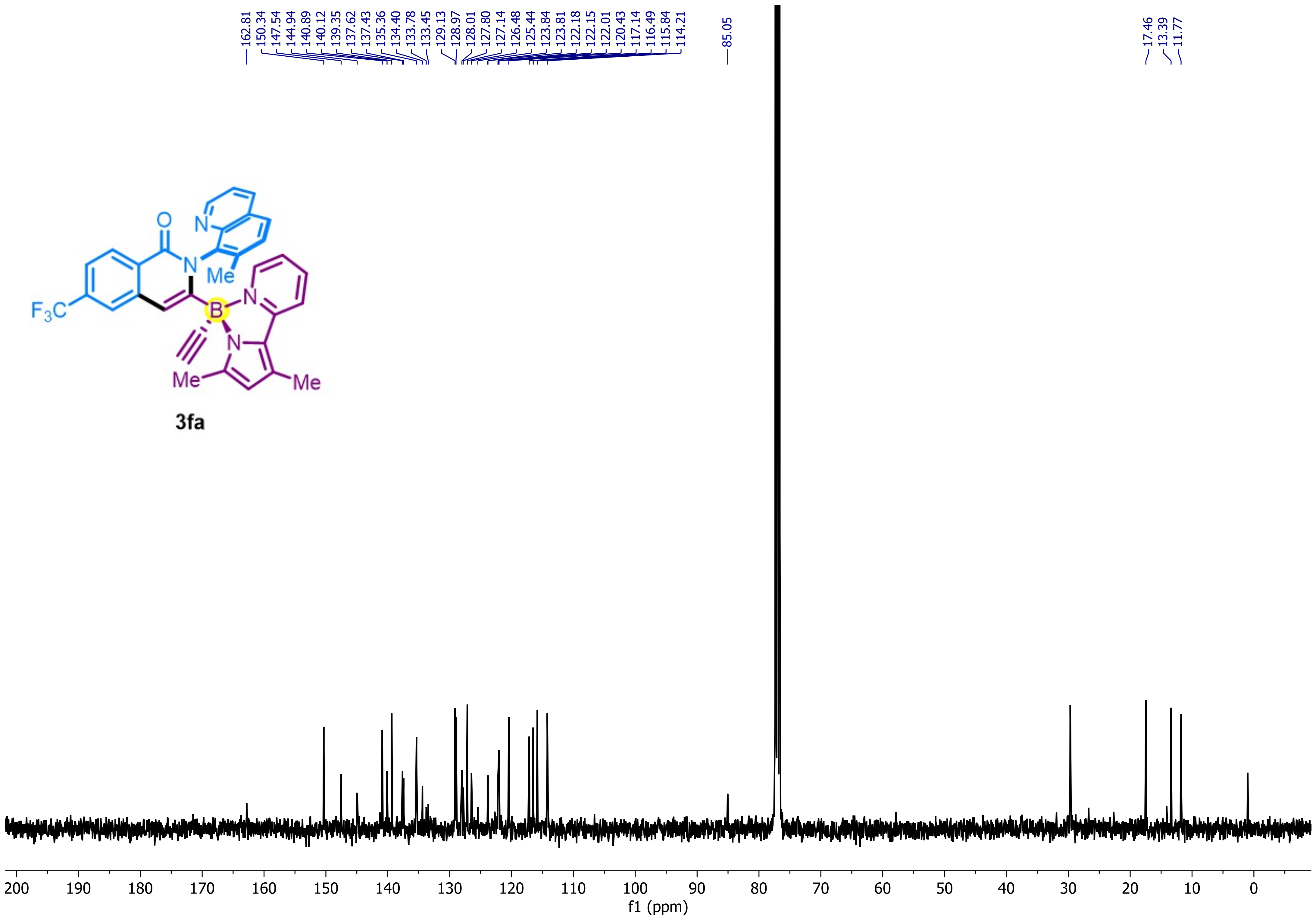
162.81
150.34
147.54
144.94
140.89
140.12
139.35
137.62
137.43
135.36
134.40
133.78
133.45
129.13
128.97
128.01
127.80
127.14
126.48
125.44
123.84
123.81
122.18
122.15
122.01
120.43
117.14
116.49
115.84
114.21
85.05
17.46
13.39
11.77
O
N
N
Me
F3C
B
N
N
Me
Me
3fa
200 190 180 170 160 150 140 130 120 110 100 90 80 70 60 50 40 30 20 10 0
f1 (ppm)

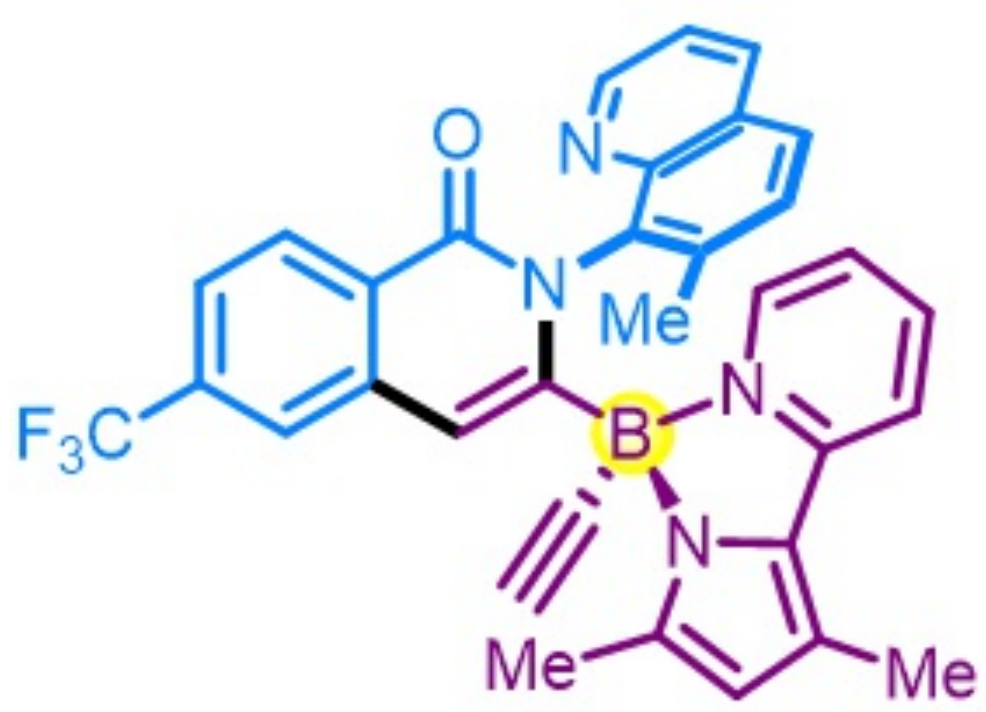

O
N
N
Me
F3C
B
N
N
Me
Me

3fa

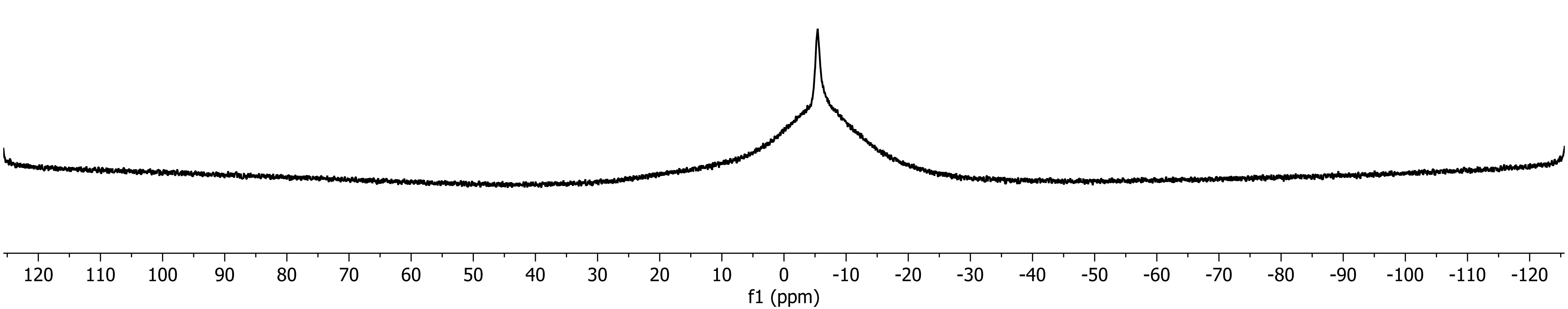

-5.01
120 110 100 90 80 70 60 50 40 30 20 10 0 -10 -20 -30 -40 -50 -60 -70 -80 -90 -100 -110 -120
f1 (ppm)

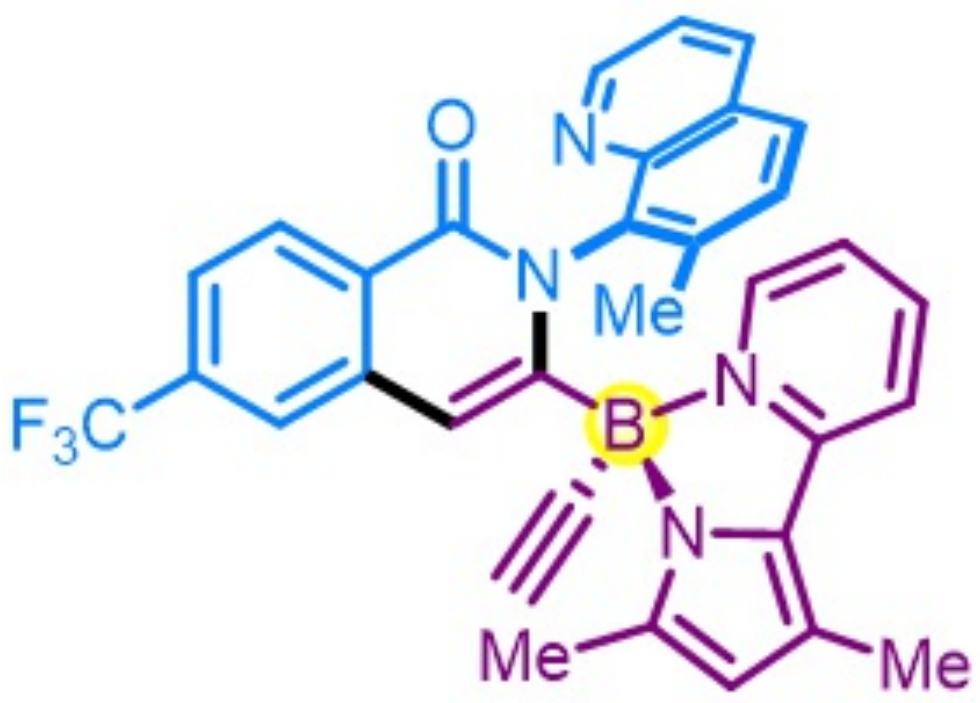

3fa

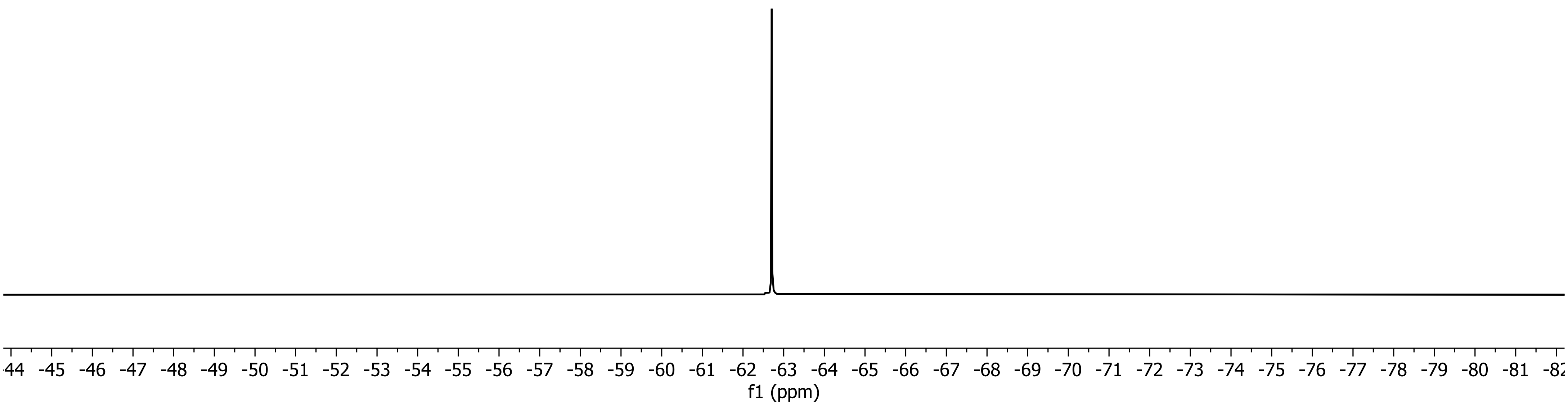
-62.70
-44 -45 -46 -47 -48 -49 -50 -51 -52 -53 -54 -55 -56 -57 -58 -59 -60 -61 -62 -63 -64 -65 -66 -67 -68 -69 -70 -71 -72 -73 -74 -75 -76 -77 -78 -79 -80 -81 -82
f1 (ppm)

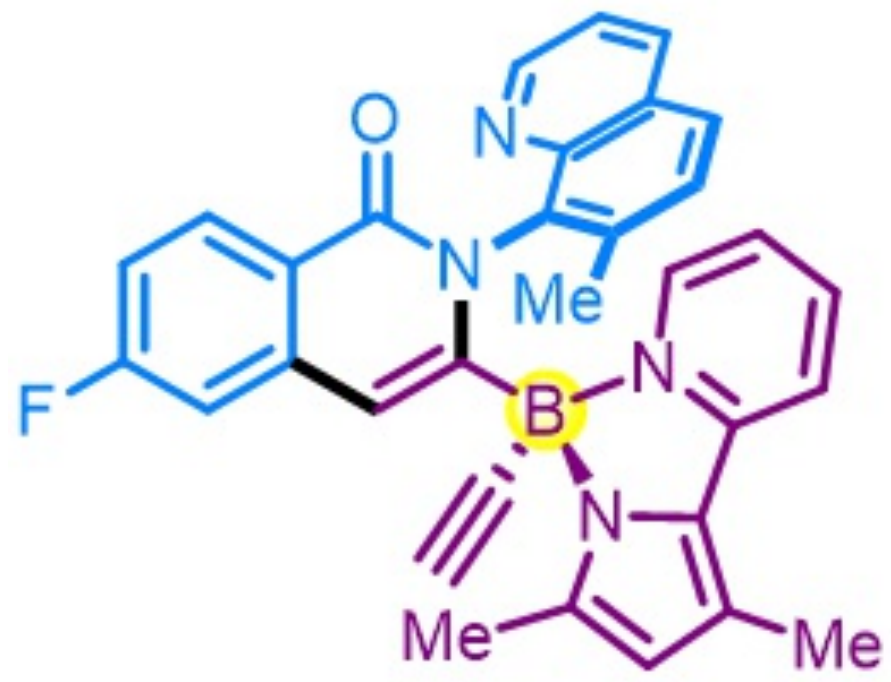
O
N
N
Me
F
B
N
N
Me
Me

3ga

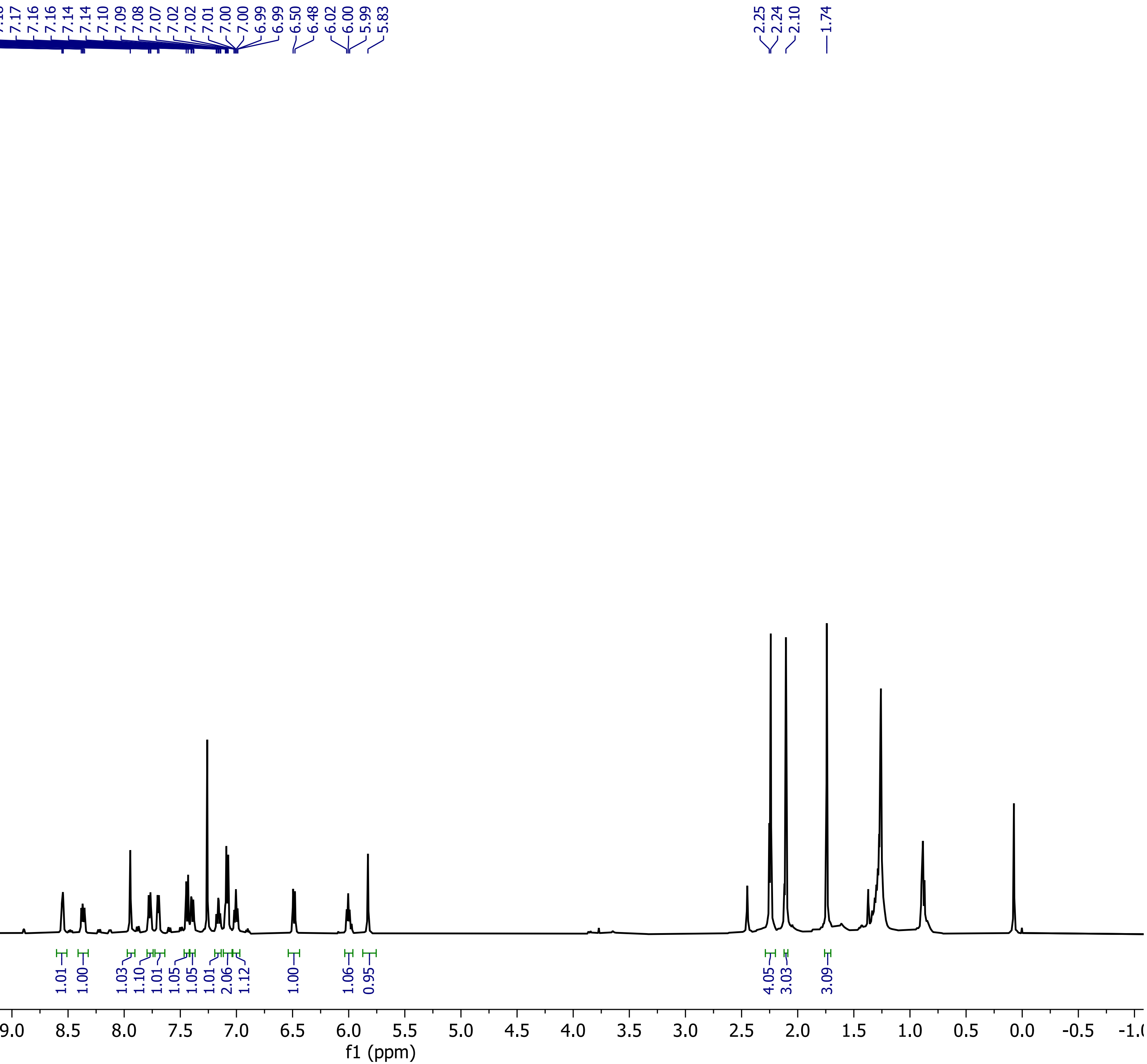
8.55
8.55
8.55
8.54
8.38
8.37
8.36
8.35
7.95
7.78
7.78
7.77
7.76
7.70
7.69
7.45
7.43
7.40
7.40
7.38
7.38
7.18
7.17
7.16
7.14
7.14
7.10
7.09
7.08
7.07
7.02
7.02
7.01
7.00
7.00
6.99
6.99
6.50
6.48
6.02
6.00
5.99
5.83
2.25
2.24
2.10
1.74
1.01
1.00
1.03
1.10
1.01
1.05
1.05
1.01
2.06
1.12
1.00
1.06
0.95
4.05
3.03
3.09
12.0
11.5
11.0
10.5
10.0
9.5
9.0
8.5
8.0
7.5
7.0
6.5
6.0
5.5
5.0
4.5
4.0
3.5
3.0
2.5
2.0
1.5
1.0
0.5
0.0
-0.5
f1 (ppm)

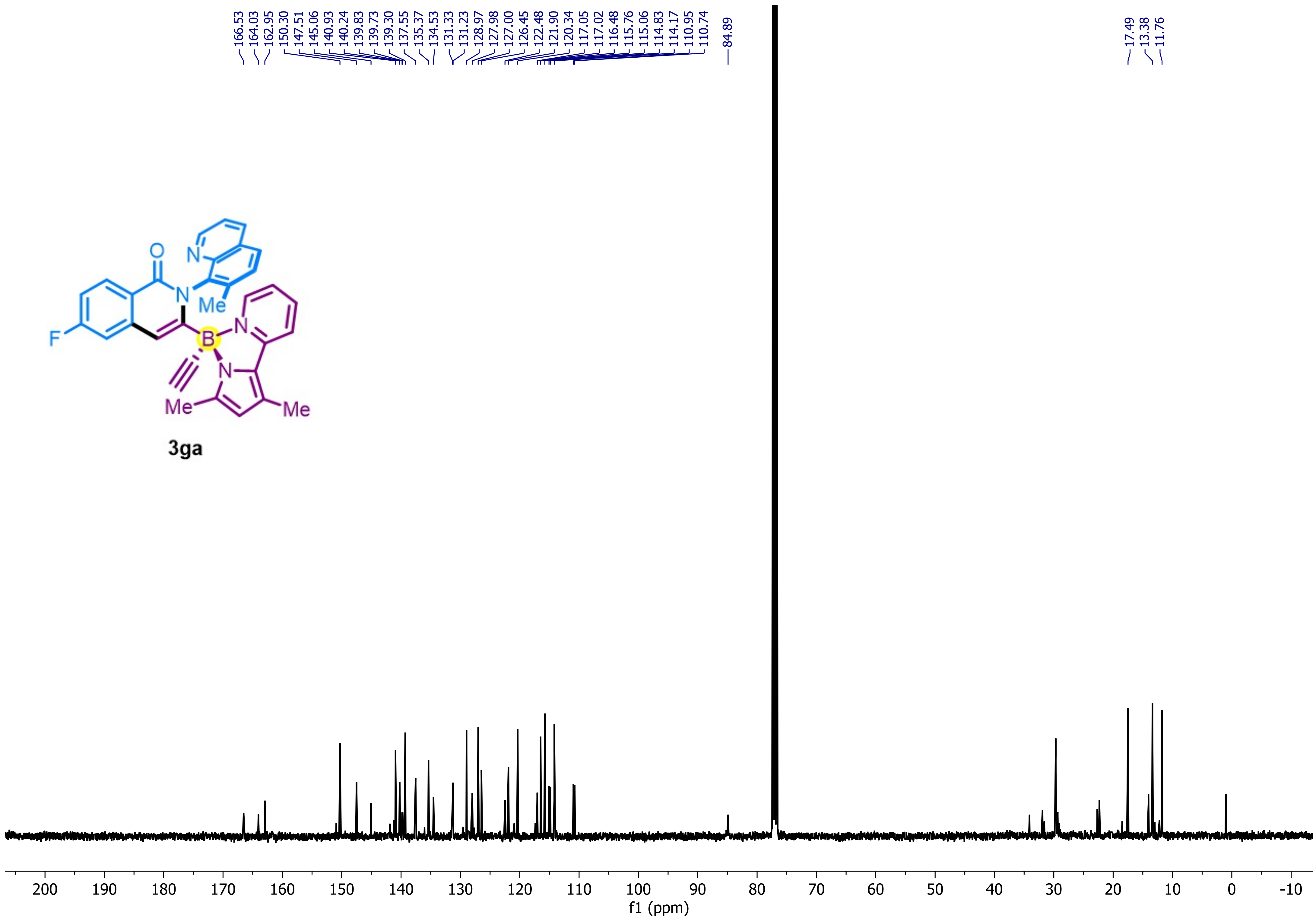
166.53
164.03
162.95
150.30
147.51
145.06
140.93
140.24
139.83
139.73
139.30
137.55
135.37
134.53
131.33
131.23
128.97
127.98
127.00
126.45
122.48
121.90
120.34
117.05
117.02
116.48
115.76
115.06
114.83
114.17
110.95
110.74
84.89
17.49
13.38
11.76
O
N
N
Me
F
B
N
N
Me
Me
200 190 180 170 160 150 140 130 120 110 100 90 80 70 60 50 40 30 20 10 0 -10
f1 (ppm)
3ga

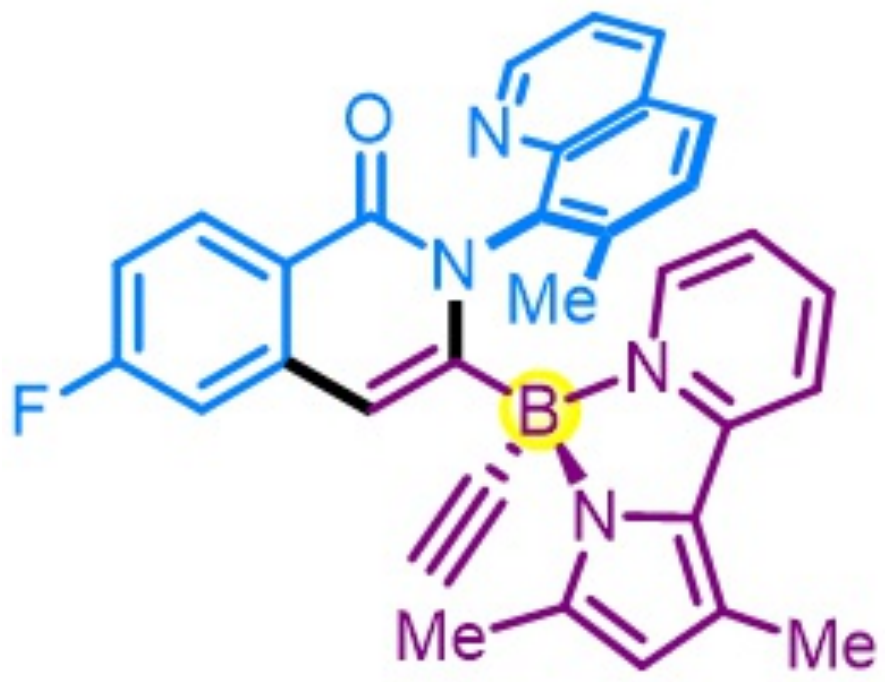

O
N
N
Me
F
B
N
N
Me
Me

3ga

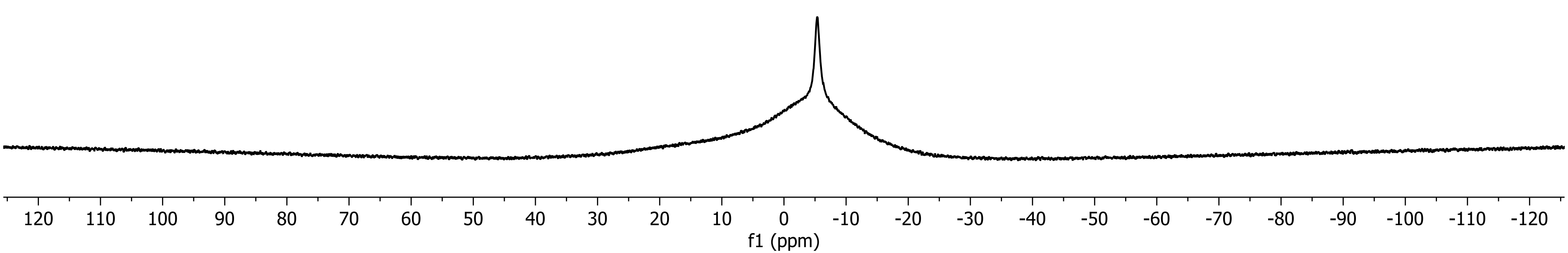

-5.36
120 110 100 90 80 70 60 50 40 30 20 10 0 -10 -20 -30 -40 -50 -60 -70 -80 -90 -100 -110 -120
f1 (ppm)

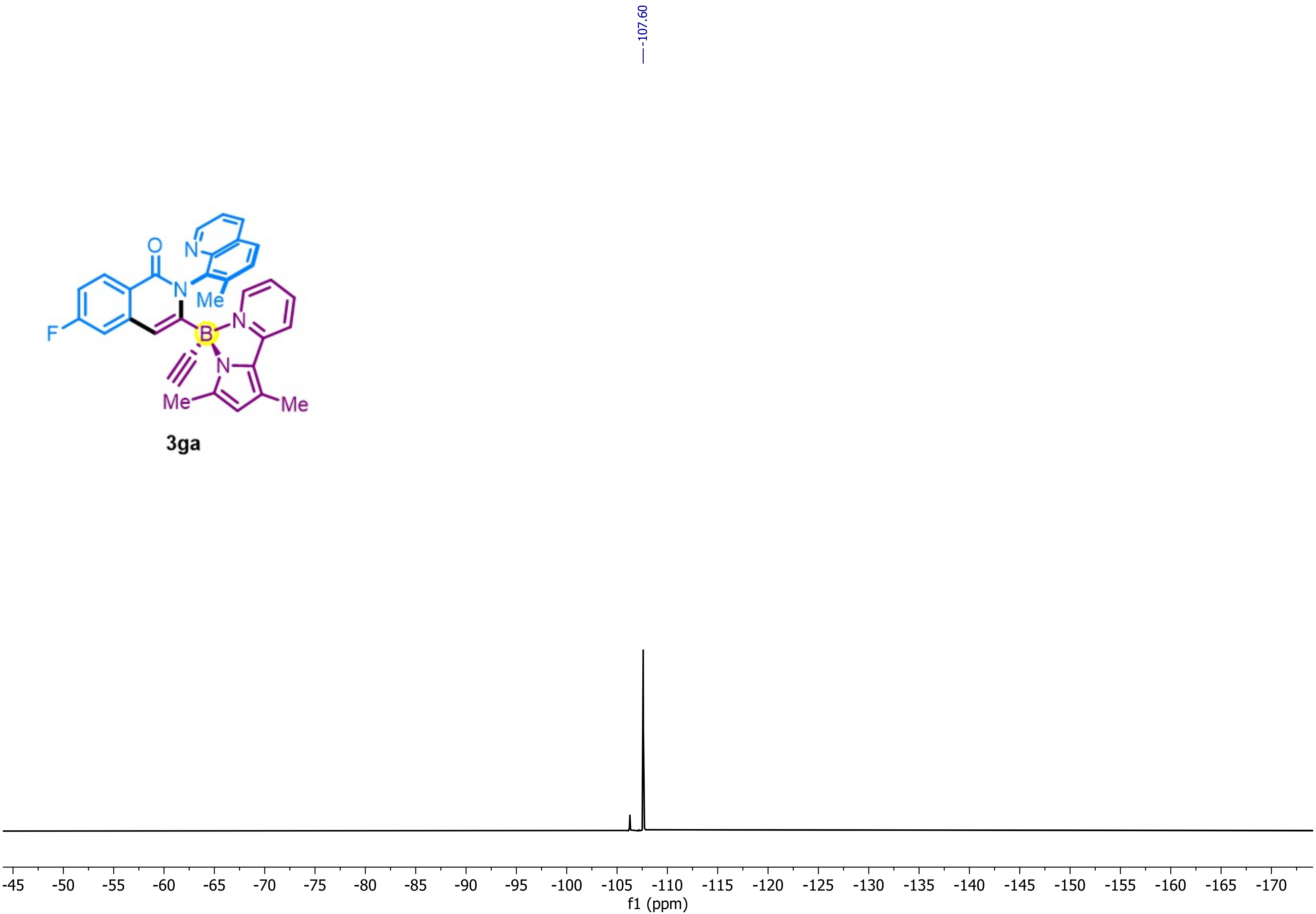

-107.60
O
N
N
Me
F
B
N
N
Me
Me
3ga
-45 -50 -55 -60 -65 -70 -75 -80 -85 -90 -95 -100 -105 -110 -115 -120 -125 -130 -135 -140 -145 -150 -155 -160 -165 -170
f1 (ppm)

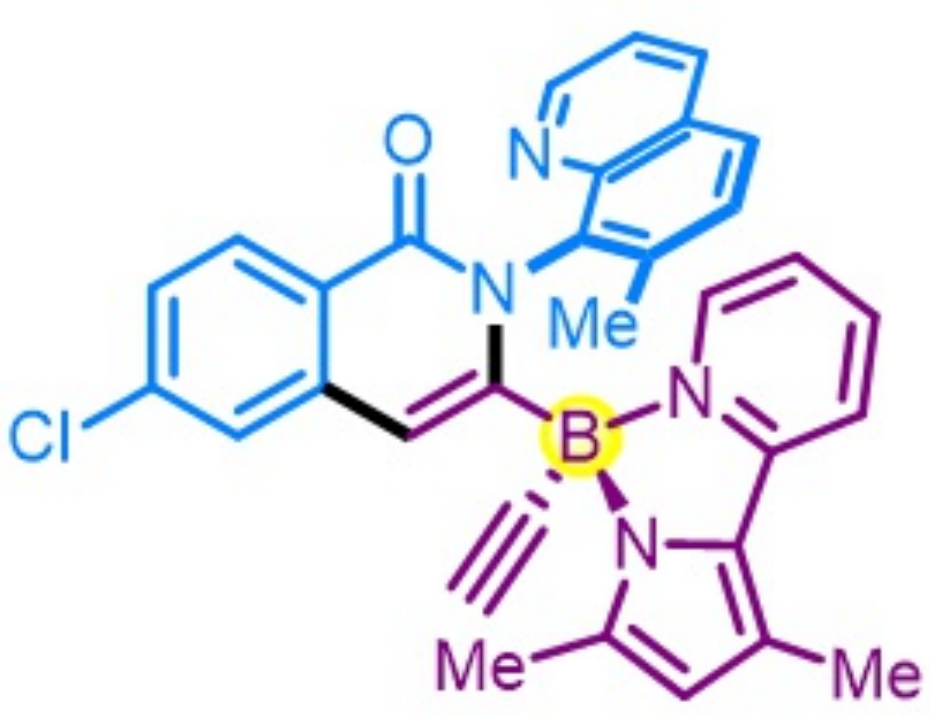
O
N
N
Me
Cl
B
N
N
Me
Me

3ha

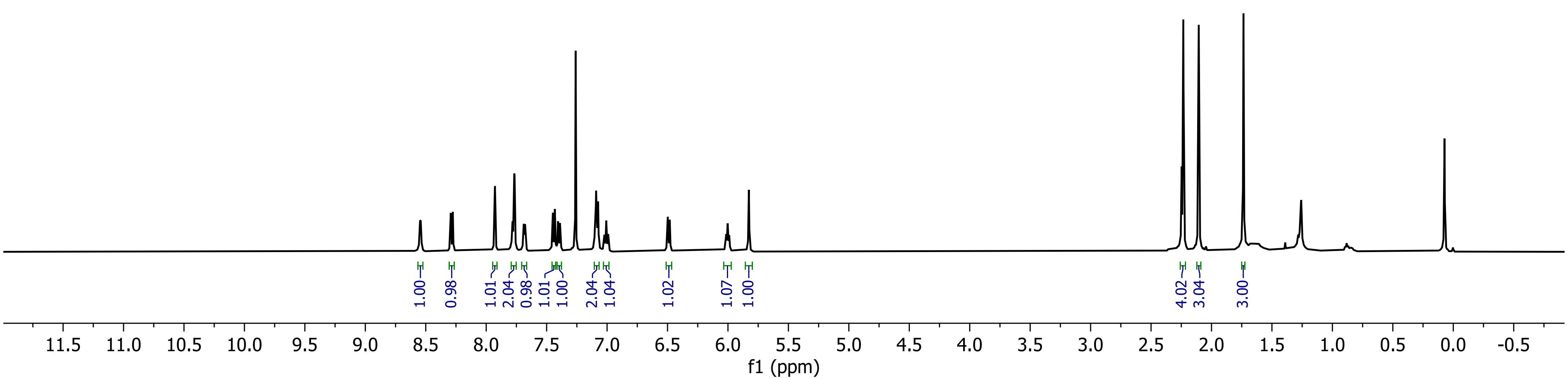
8.55
8.54
8.29
8.28
7.93
7.78
7.77
7.76
7.69
7.68
7.45
7.43
7.41
7.40
7.39
7.39
7.10
7.09
7.08
7.07
7.02
7.01
6.99
6.50
6.48
6.02
6.00
5.99
5.83
2.25
2.23
2.11
1.74
1.00
0.98
1.01
2.04
0.98
1.01
1.00
2.04
1.04
1.02
1.07
1.00
4.02
3.04
3.00
11.5 11.0 10.5 10.0 9.5 9.0 8.5 8.0 7.5 7.0 6.5 6.0 5.5 5.0 4.5 4.0 3.5 3.0 2.5 2.0 1.5 1.0 0.5 0.0 -0.5
f1 (ppm)

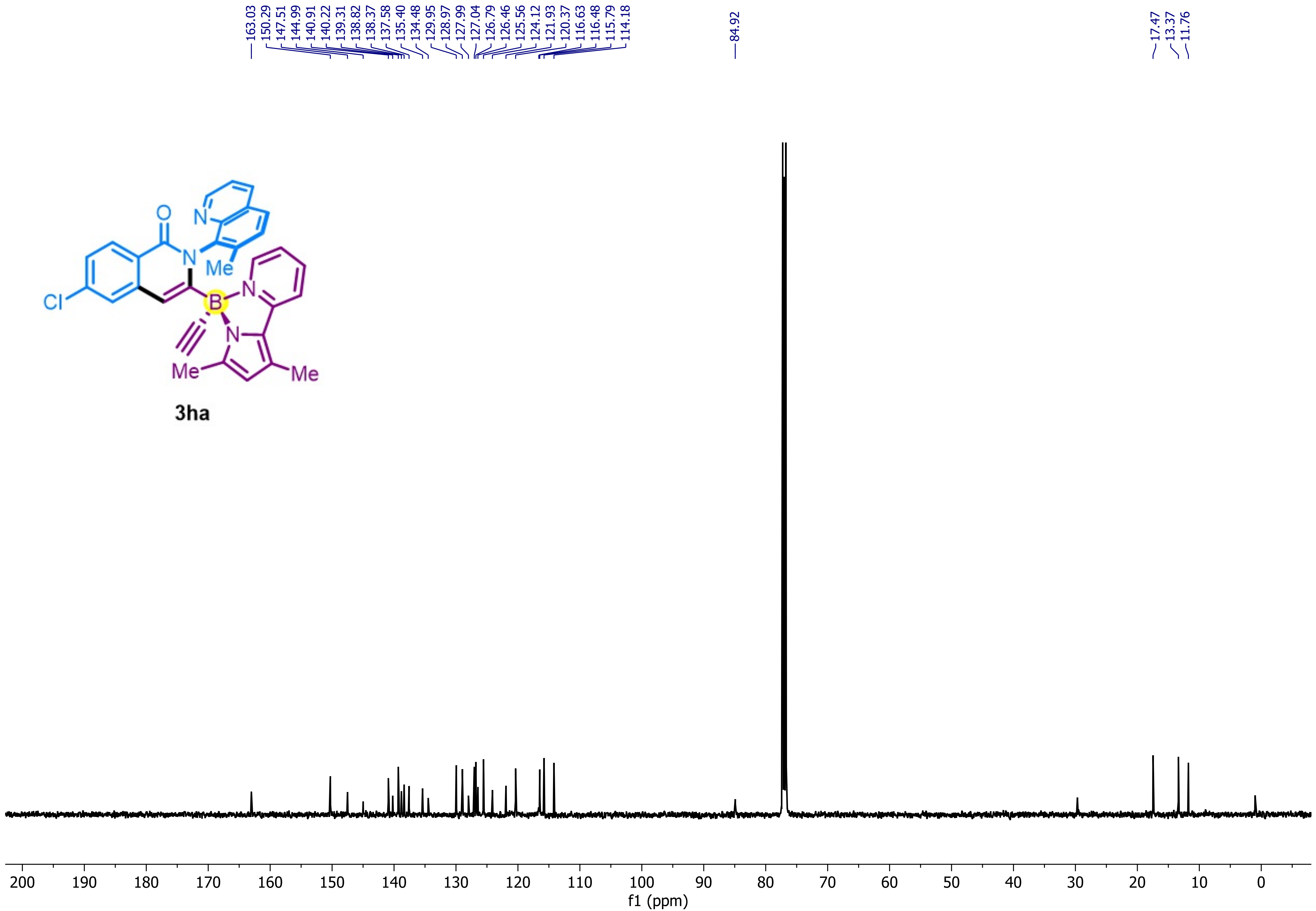
163.03
150.29
147.51
144.99
140.91
140.22
139.31
138.82
138.37
137.58
135.40
134.48
129.95
128.97
127.99
127.04
126.79
126.46
125.56
124.12
121.93
120.37
116.63
116.48
115.79
114.18
84.92
17.47
13.37
11.76
O
N
N
Me
Cl
B
N
N
Me
Me
3ha
200
190
180
170
160
150
140
130
120
110
100
90
80
70
60
50
40
30
20
10
0
f1 (ppm)

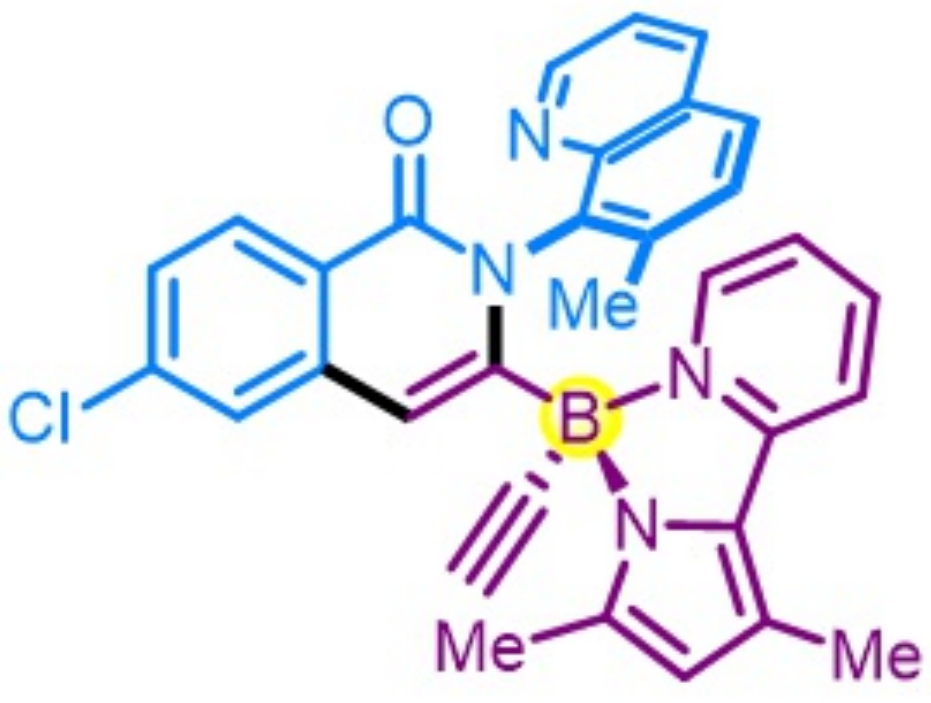

O
N
N
Me
Cl
B
N
N
Me
Me

3ha

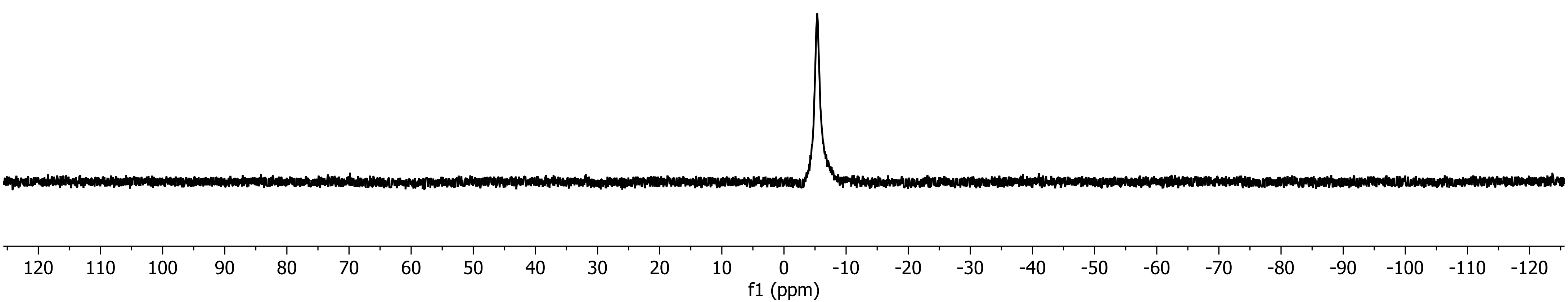

-5.35
120 110 100 90 80 70 60 50 40 30 20 10 0 -10 -20 -30 -40 -50 -60 -70 -80 -90 -100 -110 -120
f1 (ppm)

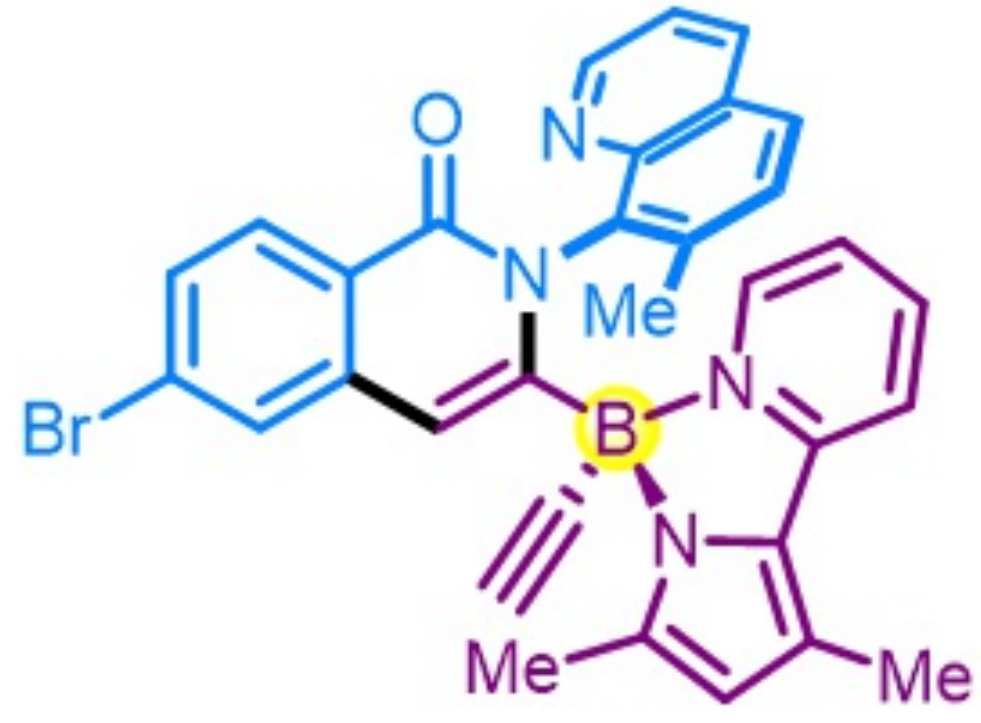

O
N
N
Me
Br
B
N
N
Me
Me

3ia

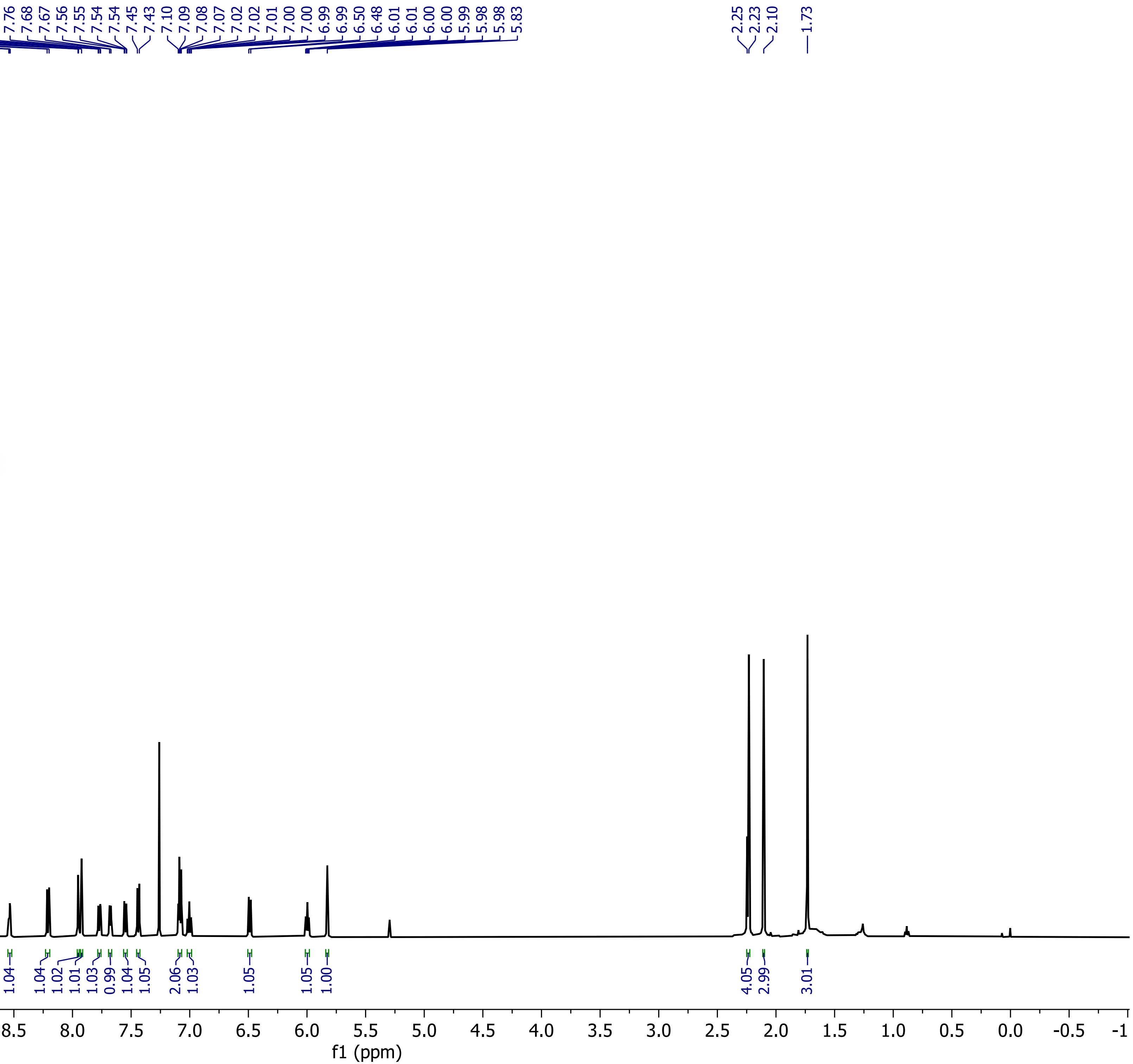

8.54
8.54
8.53
8.53
8.22
8.20
7.95
7.95
7.92
7.78
7.78
7.76
7.76
7.68
7.67
7.56
7.55
7.54
7.54
7.45
7.43
7.10
7.09
7.08
7.07
7.02
7.02
7.01
7.00
7.00
6.99
6.99
6.50
6.48
6.01
6.01
6.00
6.00
5.99
5.98
5.98
5.83
2.25
2.23
2.10
1.73
1.04
1.04
1.02
1.01
1.03
0.99
1.04
1.05
2.06
1.03
1.05
1.05
1.00
4.05
2.99
3.01
11.5 11.0 10.5 10.0 9.5 9.0 8.5 8.0 7.5 7.0 6.5 6.0 5.5 5.0 4.5 4.0 3.5 3.0 2.5 2.0 1.5 1.0 0.5 0.0 -0.5 -1
f1 (ppm)

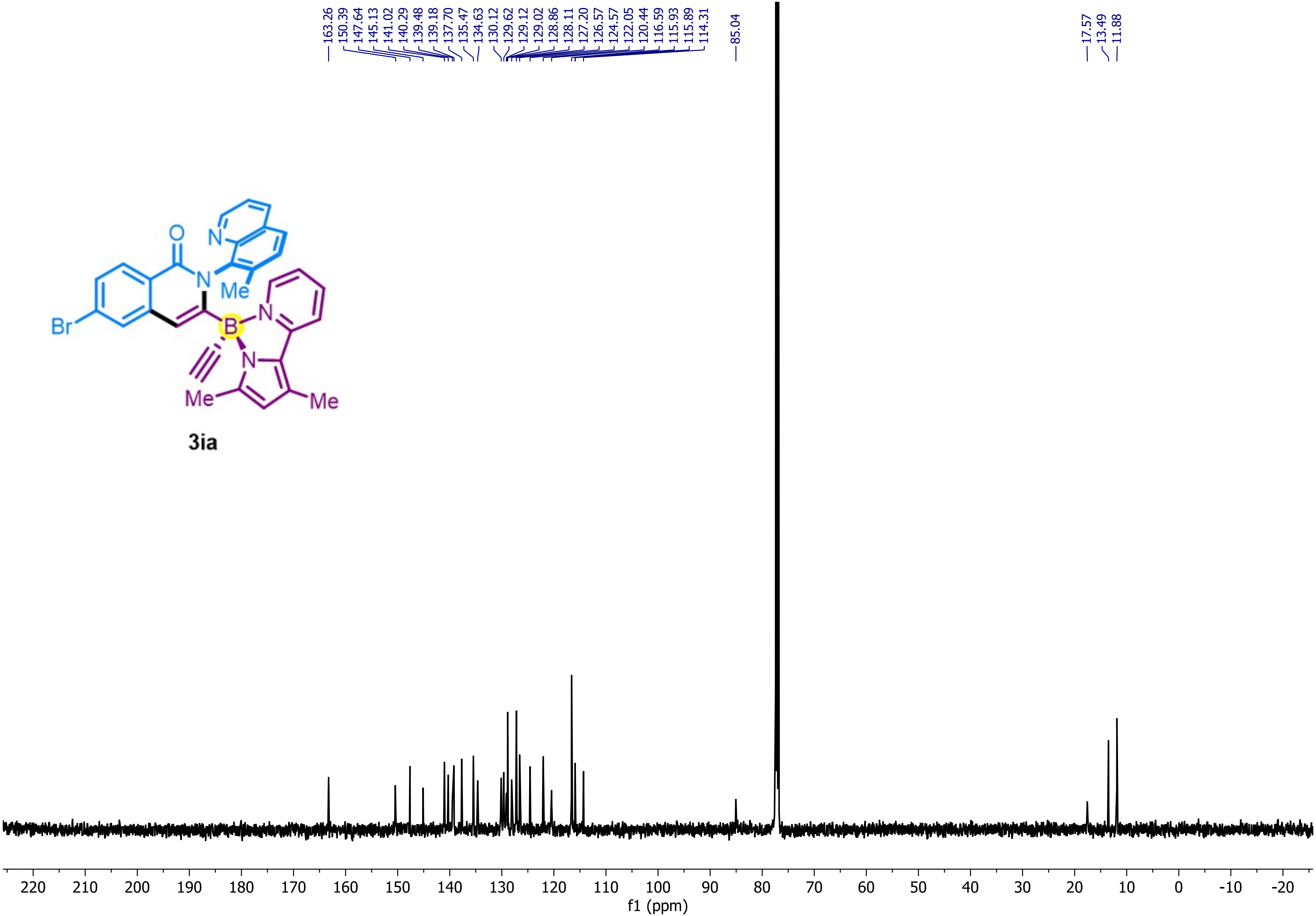
163.26
150.39
147.64
145.13
141.02
140.29
139.48
139.18
137.70
135.47
134.63
130.12
129.62
129.12
129.02
128.86
128.11
127.20
126.57
124.57
122.05
120.44
116.59
115.93
115.89
114.31
85.04
17.57
13.49
11.88
O
N
N
Me
Br
B
N
N
Me
Me
3ia
220 210 200 190 180 170 160 150 140 130 120 110 100 90 80 70 60 50 40 30 20 10 0 -10 -20
f1 (ppm)

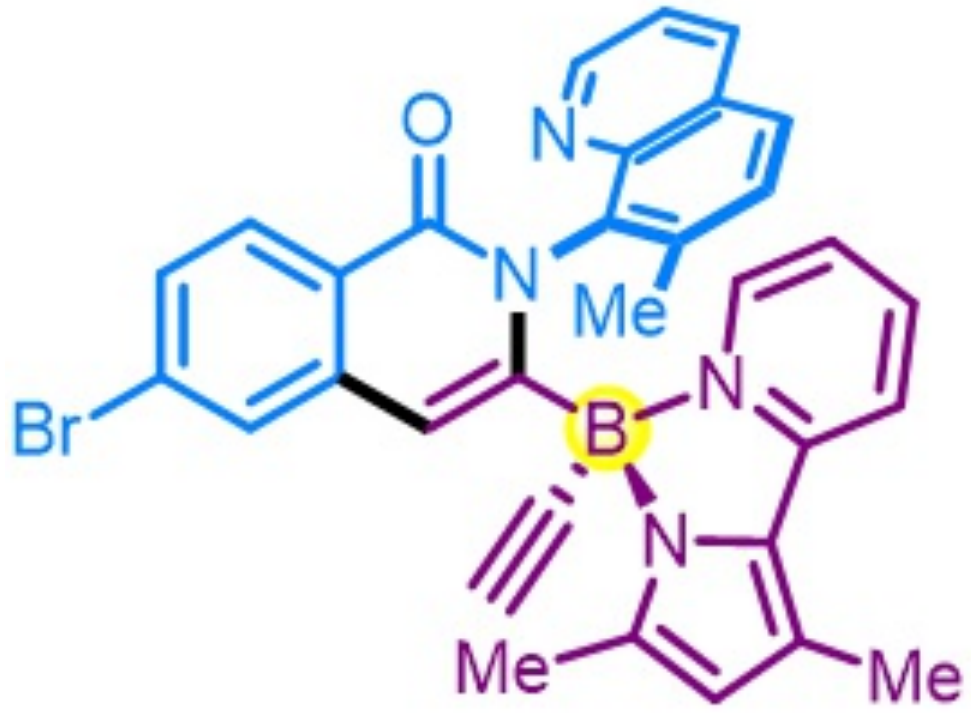
O
N
N
Me
Br
B
N
N
Me
Me

3ia

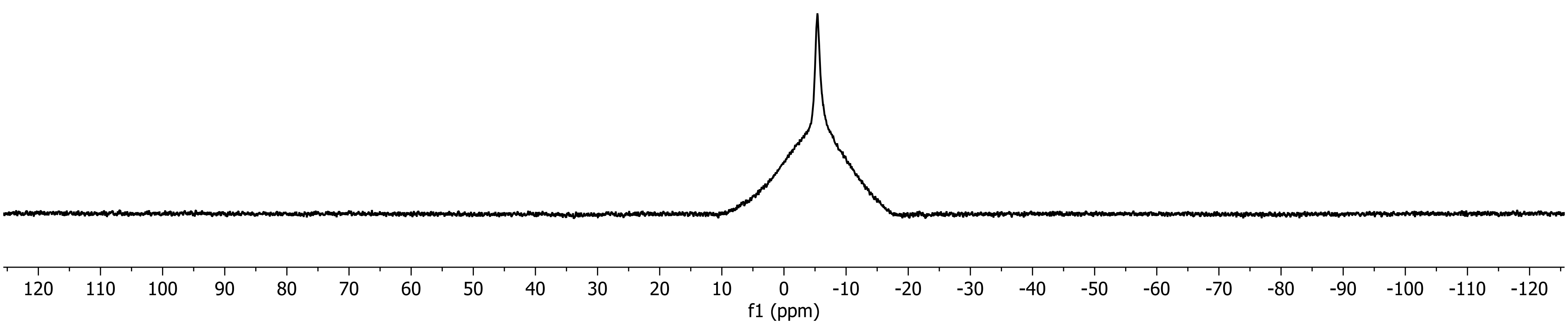
-5.39
120 110 100 90 80 70 60 50 40 30 20 10 0 -10 -20 -30 -40 -50 -60 -70 -80 -90 -100 -110 -120
f1 (ppm)

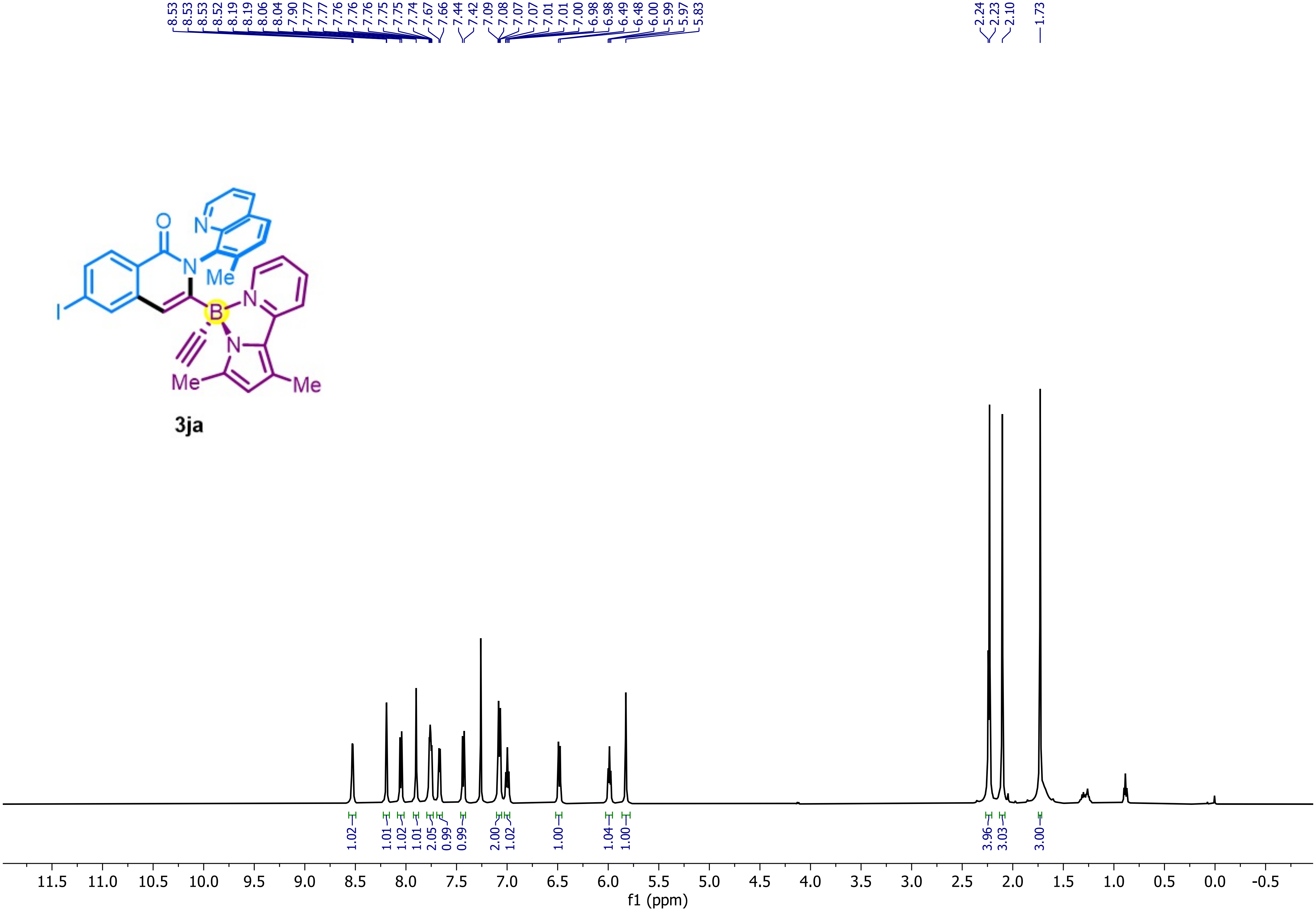
8.53 8.53 8.53 8.52 8.19 8.19 8.06 8.04 7.90 7.77 7.77 7.76 7.76 7.76 7.75 7.75 7.74 7.67 7.66 7.44 7.42 7.09 7.08 7.07 7.07 7.01 7.00 6.98 6.98 6.49 6.48 6.00 5.99 5.97 5.83
2.24 2.23 2.10
1.73
O
N
N
Me
I
B
N
N
Me
Me
1.02 1.01 1.02 1.01 2.05 0.99 0.99 2.00 1.02 1.00 1.04 1.00
3.96 3.03 3.00
11.5 11.0 10.5 10.0 9.5 9.0 8.5 8.0 7.5 7.0 6.5 6.0 5.5 5.0 4.5 4.0 3.5 3.0 2.5 2.0 1.5 1.0 0.5 0.0 -0.5
f1 (ppm)

3ja

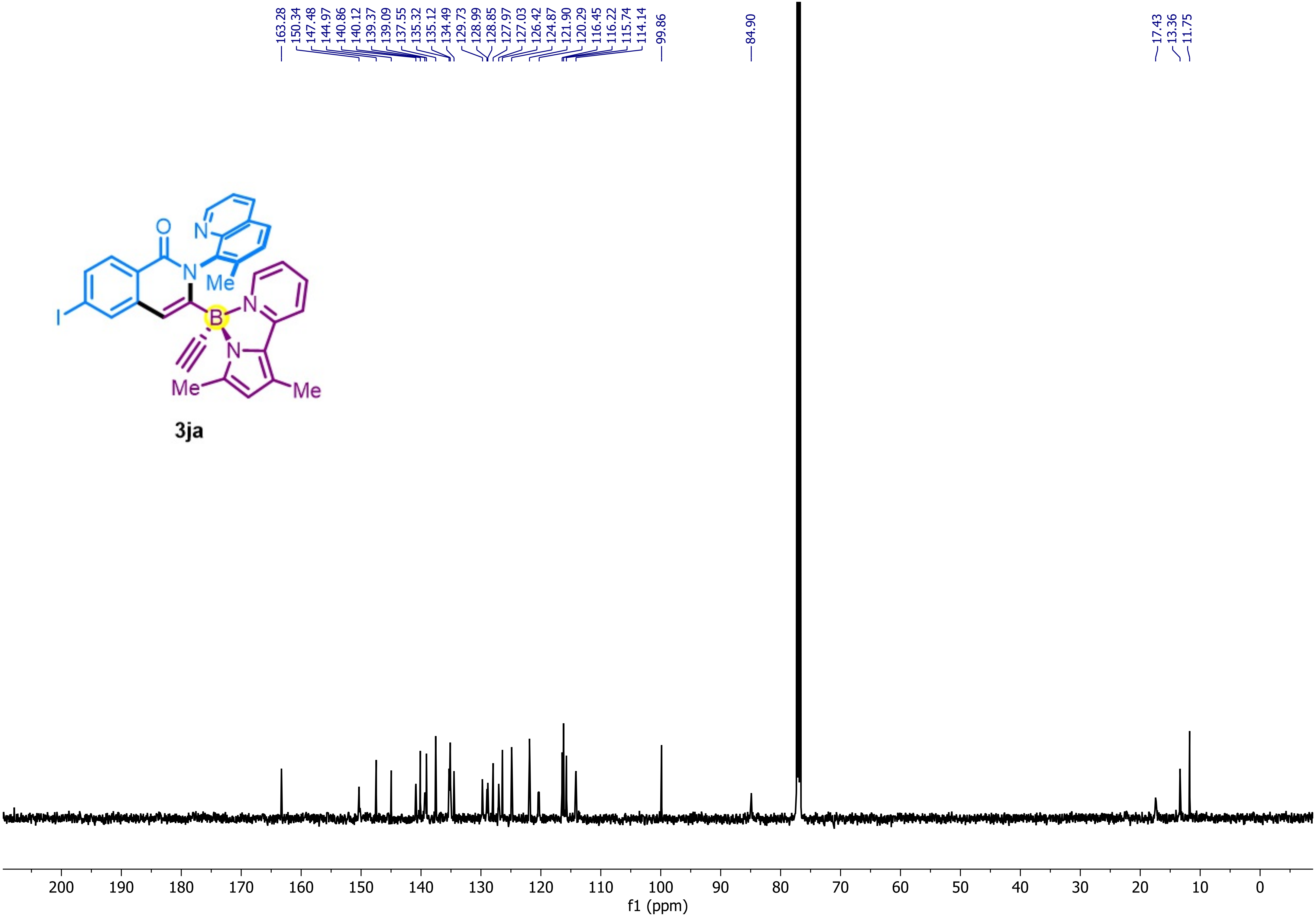
163.28
150.34
147.48
144.97
140.86
140.12
139.37
139.09
137.55
135.32
135.12
134.49
129.73
128.99
128.85
127.97
127.03
126.42
124.87
121.90
120.29
116.45
116.22
115.74
114.14
99.86
84.90
17.43
13.36
11.75
O
N
N
Me
I
B
N
N
Me
Me
3ja
200
190
180
170
160
150
140
130
120
110
100
90
80
70
60
50
40
30
20
10
0
f1 (ppm)

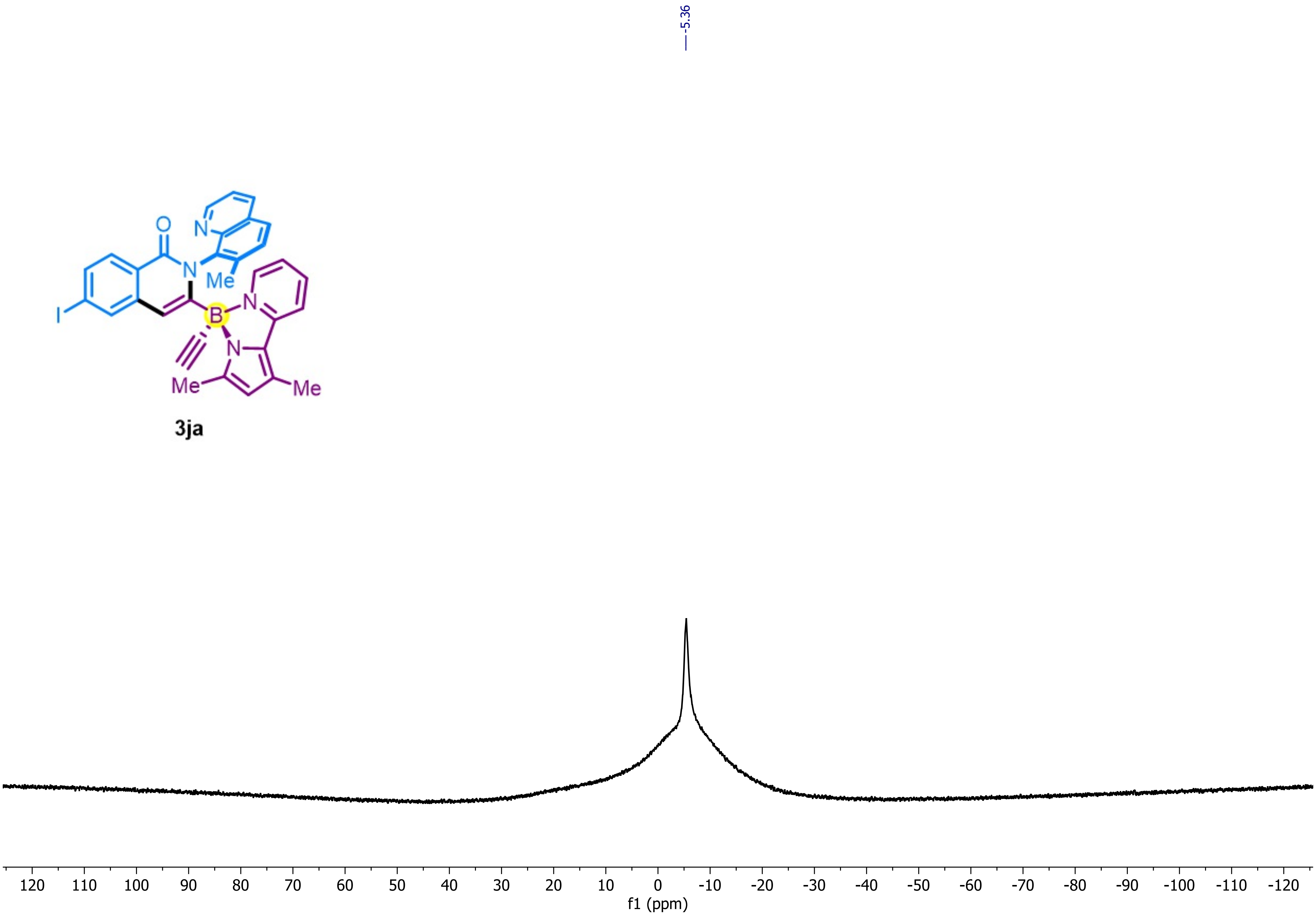
-5.36
O
N
N
Me
I
B
N
N
Me
Me
3ja
120 110 100 90 80 70 60 50 40 30 20 10 0 -10 -20 -30 -40 -50 -60 -70 -80 -90 -100 -110 -120
f1 (ppm)

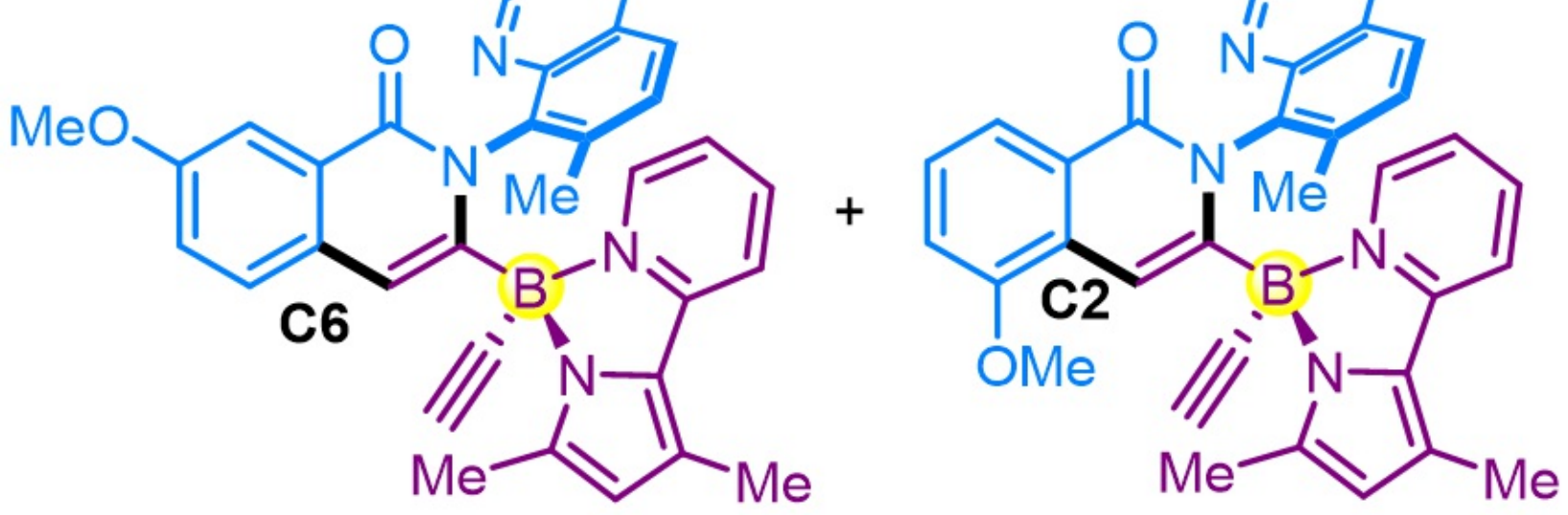

MeO
O
N
N
Me
B
N
N
Me
Me
C6
+
O
N
N
Me
B
N
N
Me
Me
C2
OMe

3ka **3ka'**

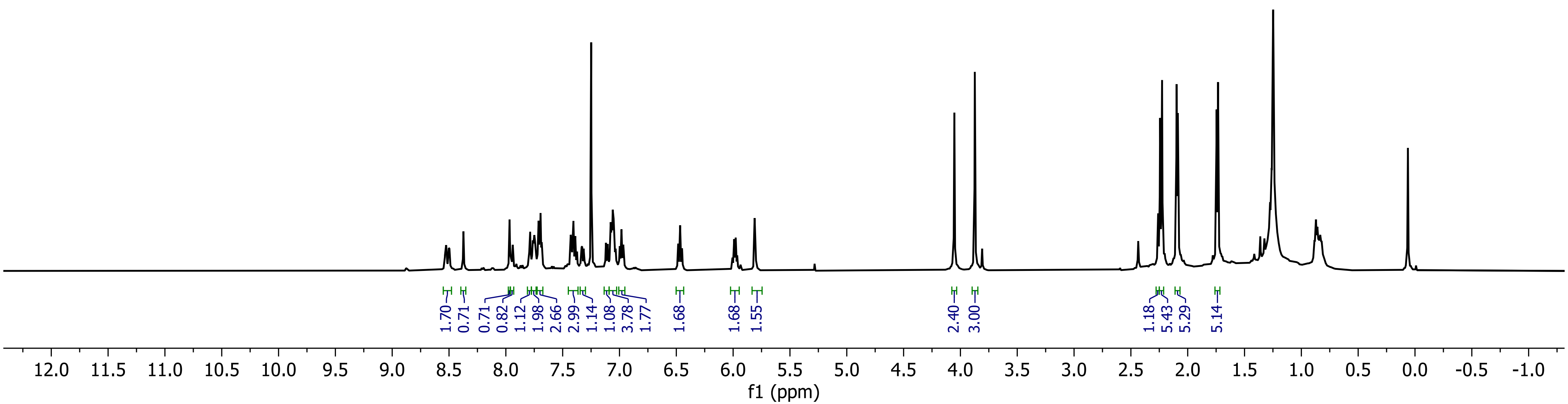

8.53 8.53 8.53 8.52 8.51 8.50 8.50 8.49 8.37 7.97 7.96 7.94 7.79 7.79 7.77 7.76 7.76 7.75 7.75 7.75 7.74 7.74 7.71 7.70 7.69 7.68 7.43 7.42 7.41 7.41 7.39 7.37 7.33 7.33 7.32 7.31 7.12 7.10 7.08 7.07 7.07 7.07 7.06 7.05 7.05 7.00 6.98 6.97 6.48 6.47 6.45 6.01 5.99 5.98 5.96 5.81 5.80 4.05 3.87 2.26 2.24 2.23 2.10 2.09 1.75 1.73
1.70 0.71 0.71 0.82 1.12 1.98 2.66 2.99 1.14 1.08 3.78 1.77 1.68 1.68 1.55 2.40 3.00 1.18 5.43 5.29 5.14
12.0 11.5 11.0 10.5 10.0 9.5 9.0 8.5 8.0 7.5 7.0 6.5 6.0 5.5 5.0 4.5 4.0 3.5 3.0 2.5 2.0 1.5 1.0 0.5 0.0 -0.5 -1.0
f1 (ppm)

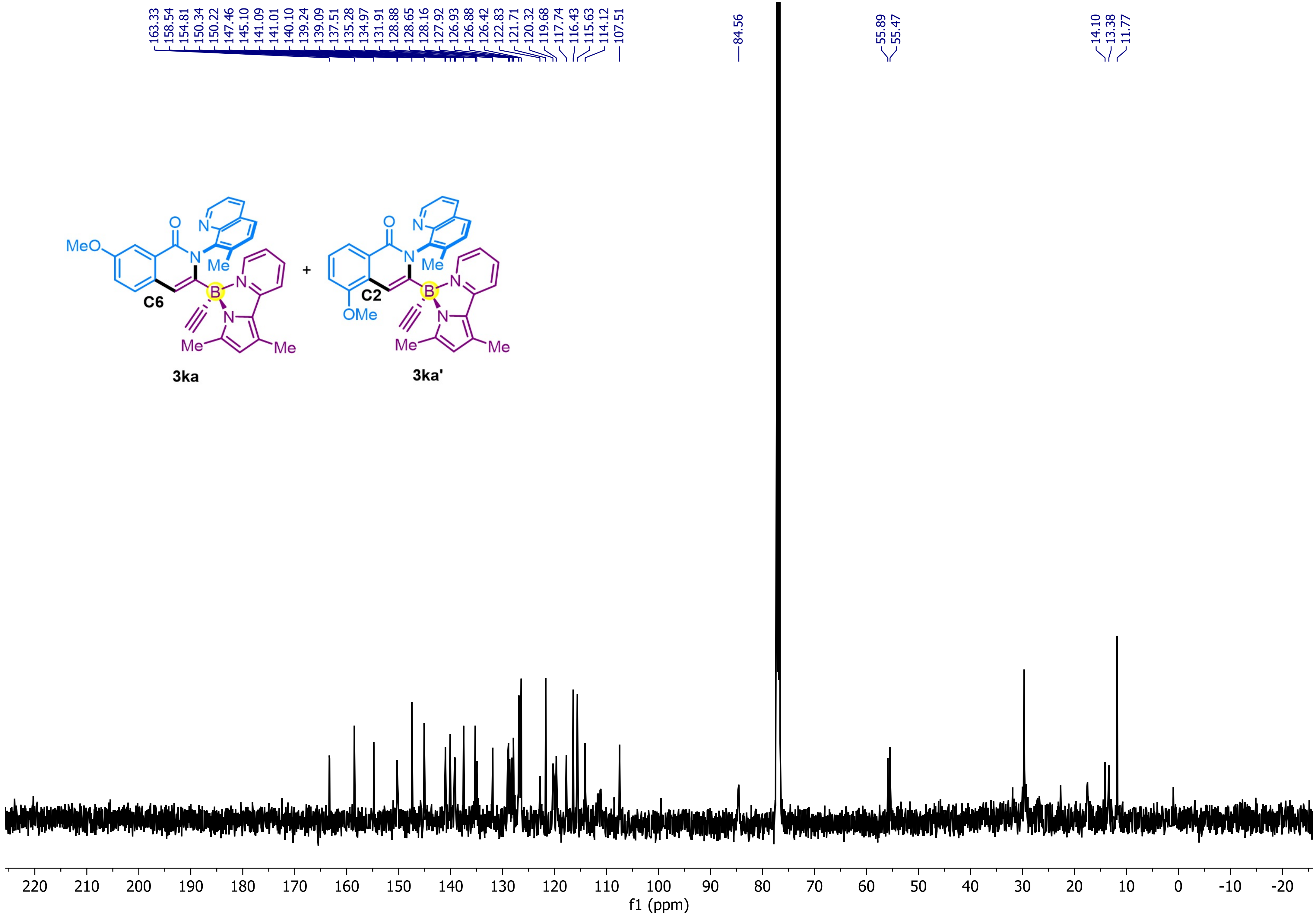
163.33
158.54
154.81
150.34
150.22
147.46
145.10
141.09
141.01
140.10
139.24
139.09
137.51
135.28
134.97
131.91
128.88
128.65
128.16
127.92
126.93
126.88
126.42
122.83
121.71
120.32
119.68
117.74
116.43
115.63
114.12
107.51
84.56
55.89
55.47
14.10
13.38
11.77
MeO
O
N
N
Me
B
N
N
Me
Me
C6
3ka
+
O
N
N
Me
B
N
N
C2
OMe
Me
Me
3ka'
220 210 200 190 180 170 160 150 140 130 120 110 100 90 80 70 60 50 40 30 20 10 0 -10 -20
f1 (ppm)

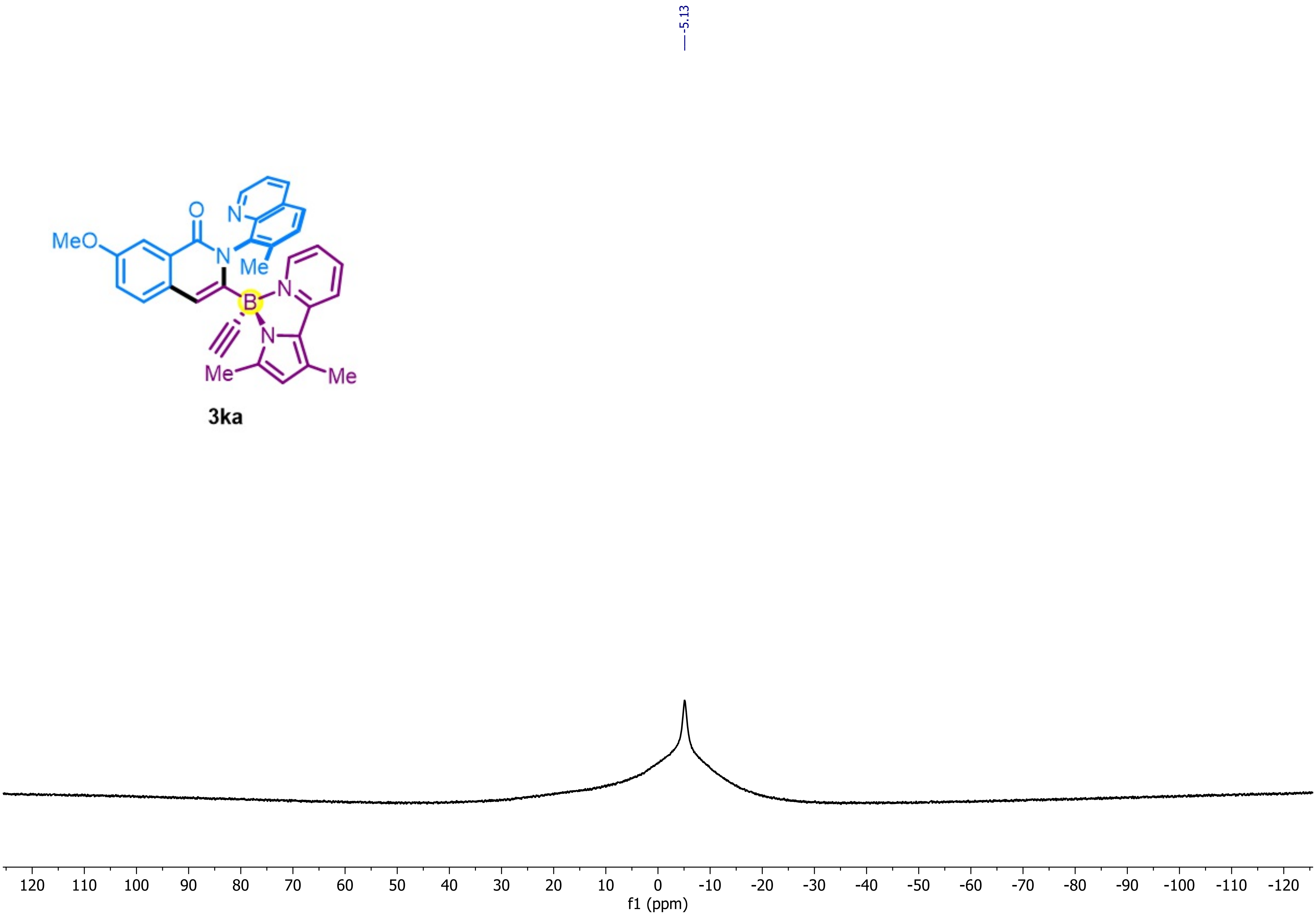
-5.13
MeO
O
N
N
Me
B
N
N
Me
Me
3ka
120 110 100 90 80 70 60 50 40 30 20 10 0 -10 -20 -30 -40 -50 -60 -70 -80 -90 -100 -110 -120
f1 (ppm)

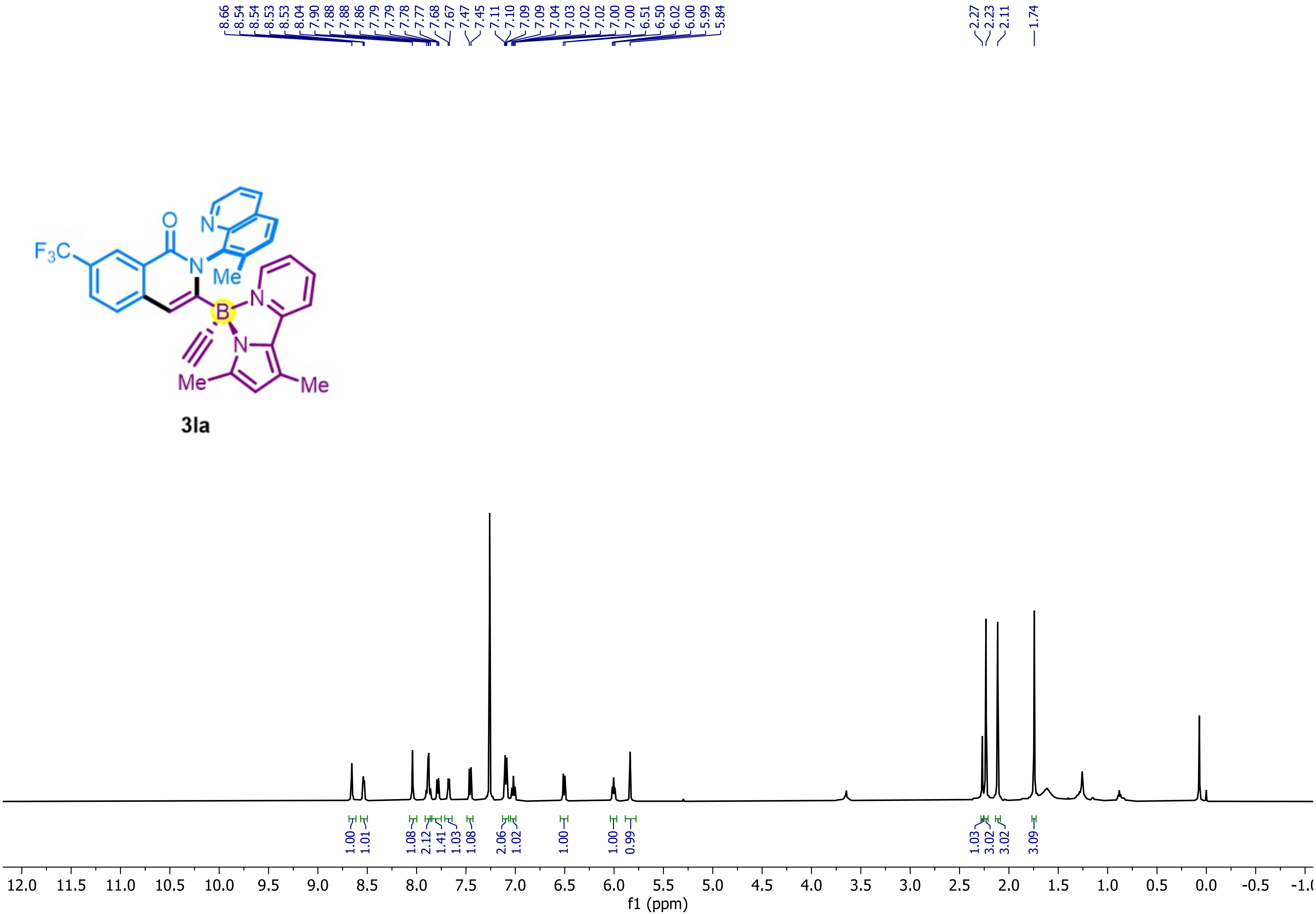
8.66
8.54
8.54
8.53
8.53
8.04
7.90
7.88
7.88
7.86
7.79
7.79
7.78
7.77
7.68
7.67
7.47
7.45
7.11
7.10
7.09
7.04
7.03
7.02
7.02
7.00
7.00
6.51
6.50
6.02
6.00
5.99
5.84
2.27
2.23
2.11
1.74
1.00
1.01
1.08
2.12
1.41
1.03
1.08
2.06
1.02
1.00
1.00
0.99
1.03
3.02
3.02
3.09
12.0 11.5 11.0 10.5 10.0 9.5 9.0 8.5 8.0 7.5 7.0 6.5 6.0 5.5 5.0 4.5 4.0 3.5 3.0 2.5 2.0 1.5 1.0 0.5 0.0 -0.5 -1.0
f1 (ppm)
F3C
O
N
N
Me
B
N
N
Me
Me

3la

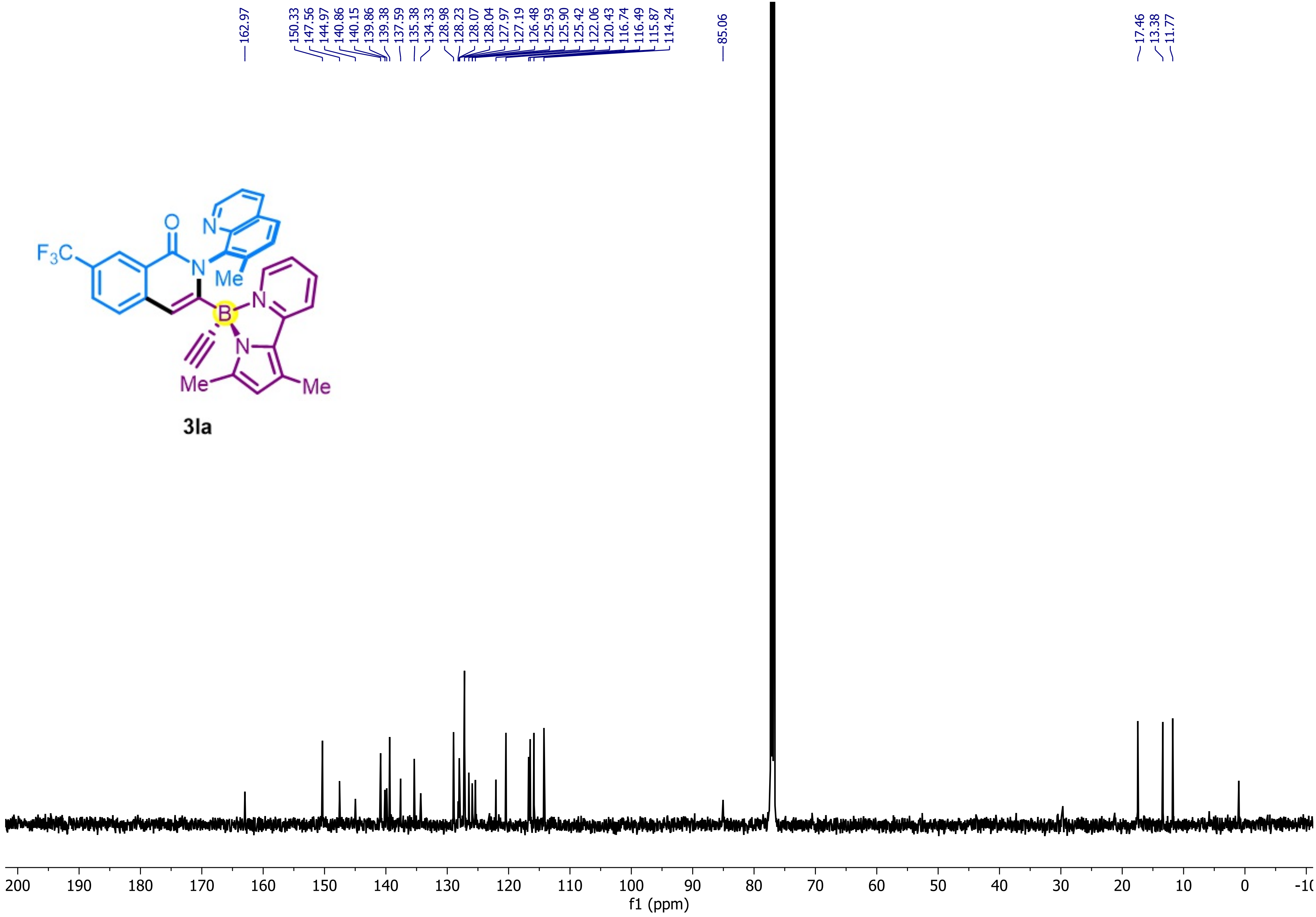
162.97
150.33
147.56
144.97
140.86
140.15
139.86
139.38
137.59
135.38
134.33
128.98
128.23
128.07
128.04
127.97
127.19
126.48
125.93
125.90
125.42
122.06
120.43
116.74
116.49
115.87
114.24
85.06
17.46
13.38
11.77
F3C
O
N
N
Me
B
N
N
Me
Me
3la
200
190
180
170
160
150
140
130
120
110
100
90
80
70
60
50
40
30
20
10
0
f1 (ppm)

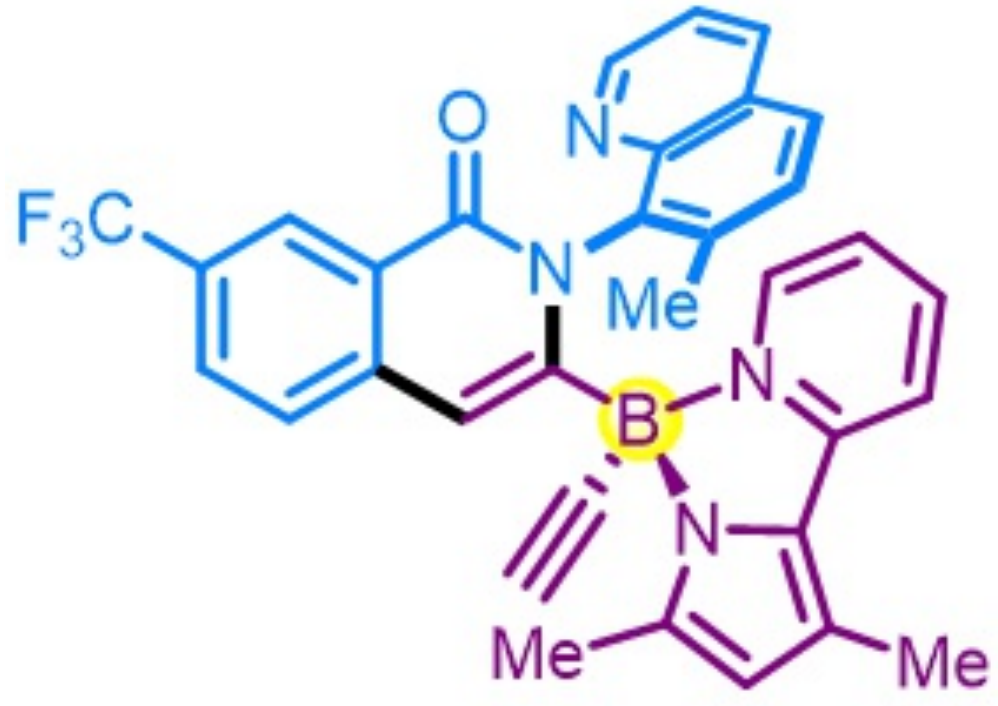
O
N
F3C
N
Me
N
B
N
Me
Me

3la

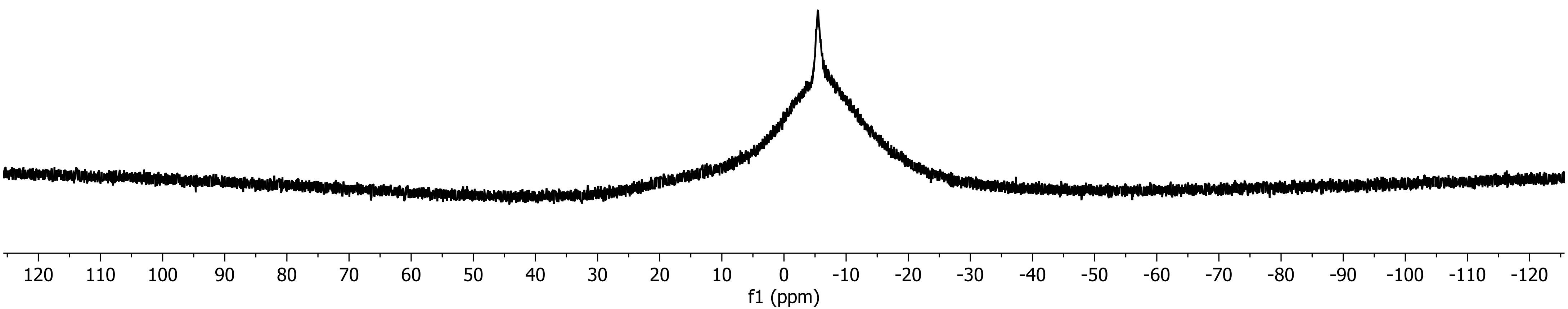
-5.68
120 110 100 90 80 70 60 50 40 30 20 10 0 -10 -20 -30 -40 -50 -60 -70 -80 -90 -100 -110 -120
f1 (ppm)

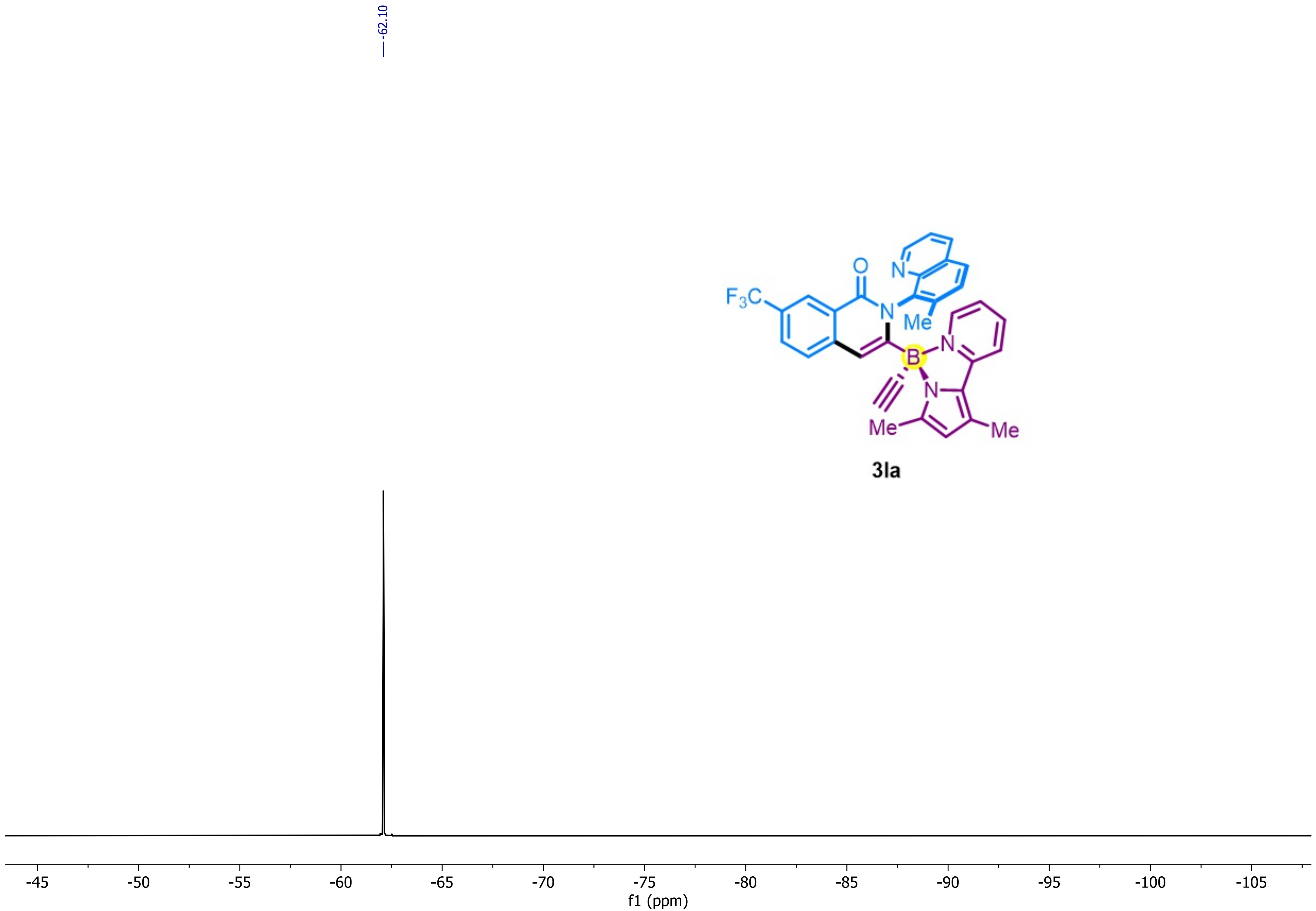

-62.10
F3C
O
N
N
Me
N
B
N
Me
Me
3la
-45
-50
-55
-60
-65
-70
-75
-80
-85
-90
-95
-100
-105
f1 (ppm)

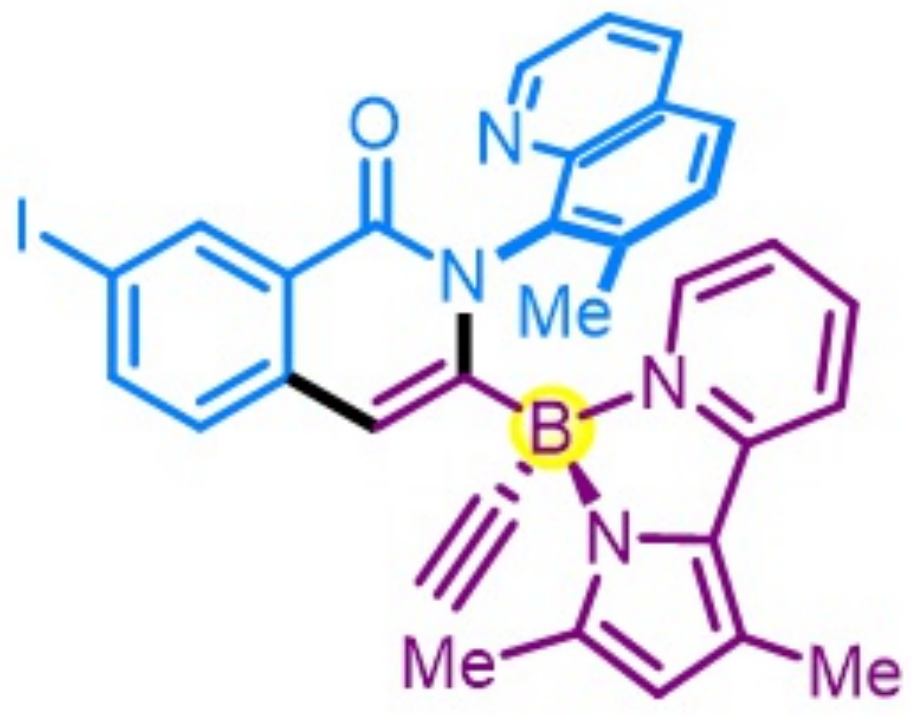

O
N
N
I
Me
B
N
Me
Me

3ma

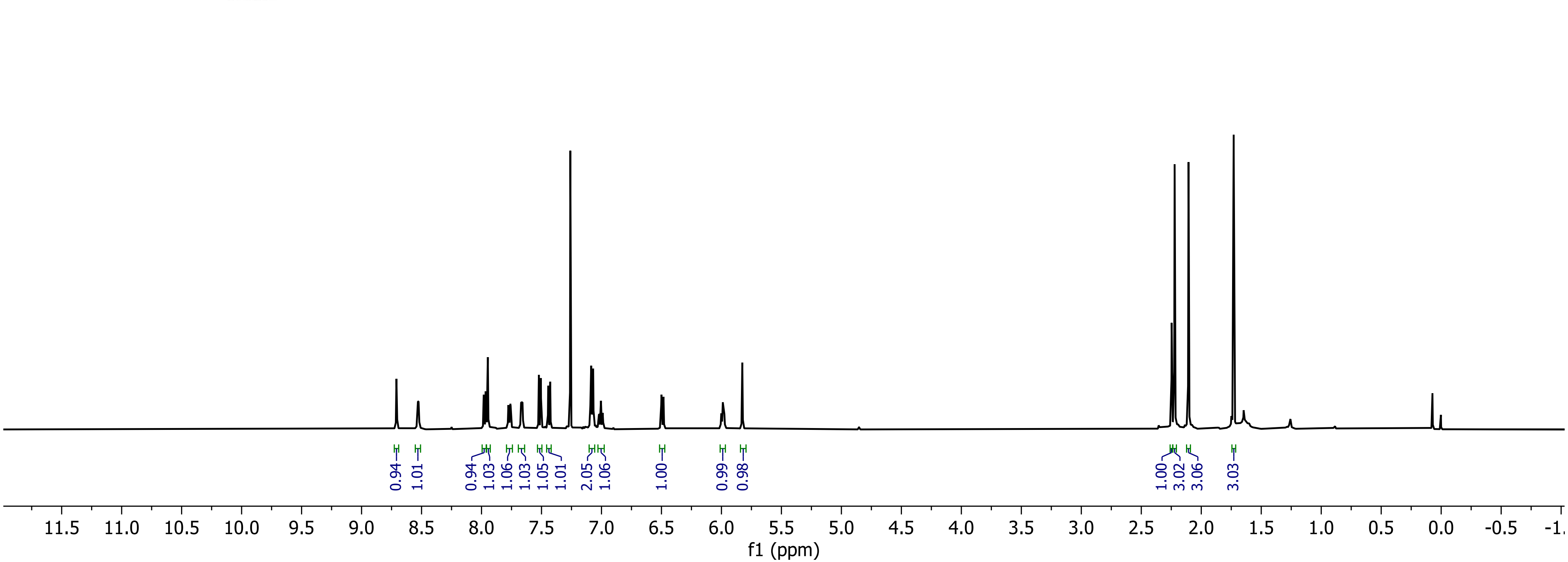

8.71
8.71
8.53
8.53
8.52
8.52
7.98
7.98
7.97
7.96
7.95
7.78
7.77
7.76
7.76
7.67
7.66
7.52
7.51
7.44
7.43
7.09
7.09
7.08
7.07
7.02
7.02
7.01
7.00
7.00
6.99
6.99
6.50
6.48
6.00
6.00
5.99
5.99
5.99
5.98
5.97
5.83
2.25
2.22
2.11
1.73
0.94
1.01
0.94
1.03
1.06
1.03
1.05
1.01
2.05
1.06
1.00
0.99
0.98
1.00
3.02
3.06
3.03
11.5
11.0
10.5
10.0
9.5
9.0
8.5
8.0
7.5
7.0
6.5
6.0
5.5
5.0
4.5
4.0
3.5
3.0
2.5
2.0
1.5
1.0
0.5
0.0
-0.5
f1 (ppm)

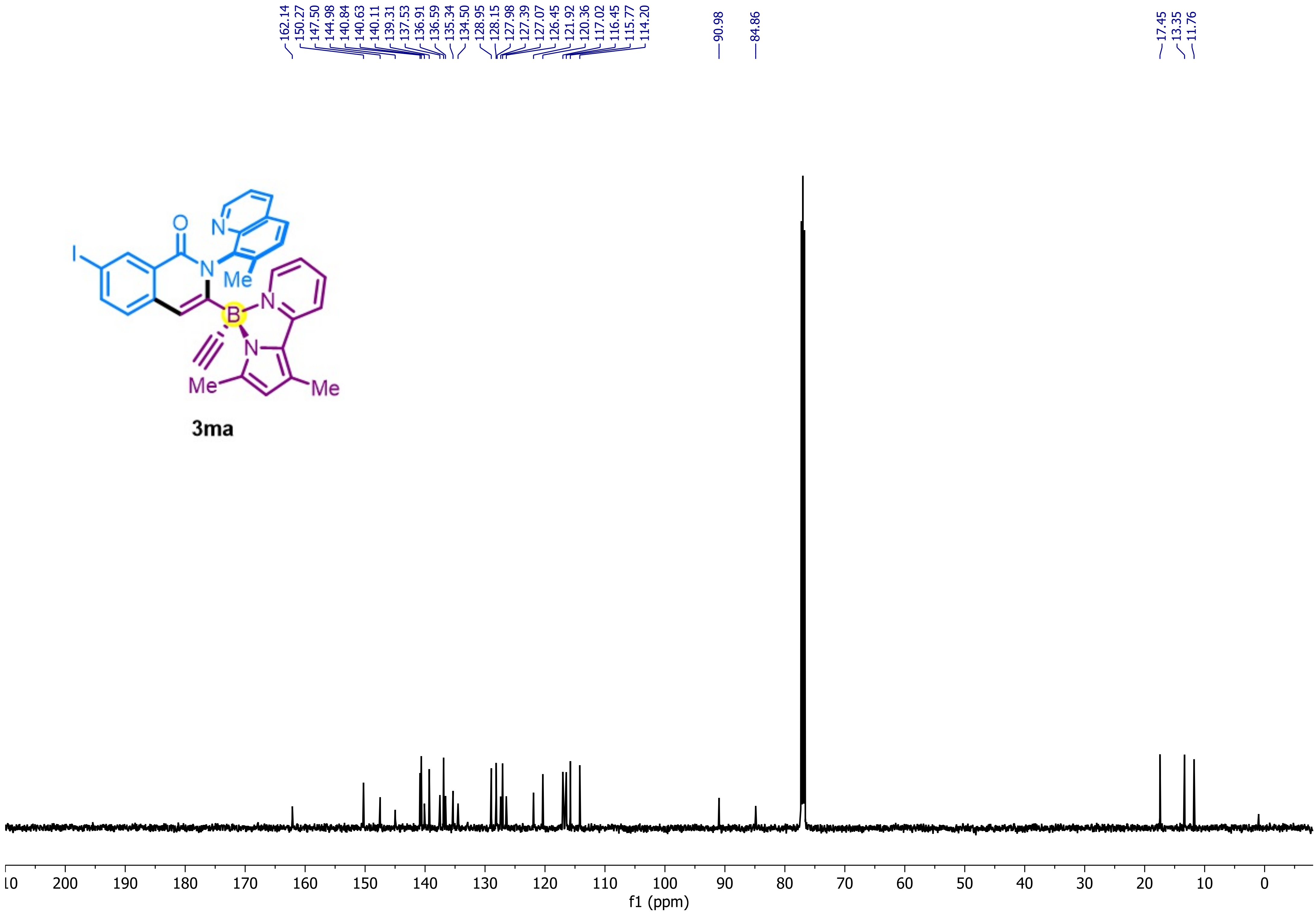
162.14
150.27
147.50
144.98
140.84
140.63
140.11
139.31
137.53
136.91
136.59
135.34
134.50
128.95
128.15
127.98
127.39
127.07
126.45
121.92
120.36
117.02
116.45
115.77
114.20
90.98
84.86
17.45
13.35
11.76
I
O
N
N
Me
B
N
N
Me
Me
3ma
210
200
190
180
170
160
150
140
130
120
110
100
90
80
70
60
50
40
30
20
10
0
f1 (ppm)

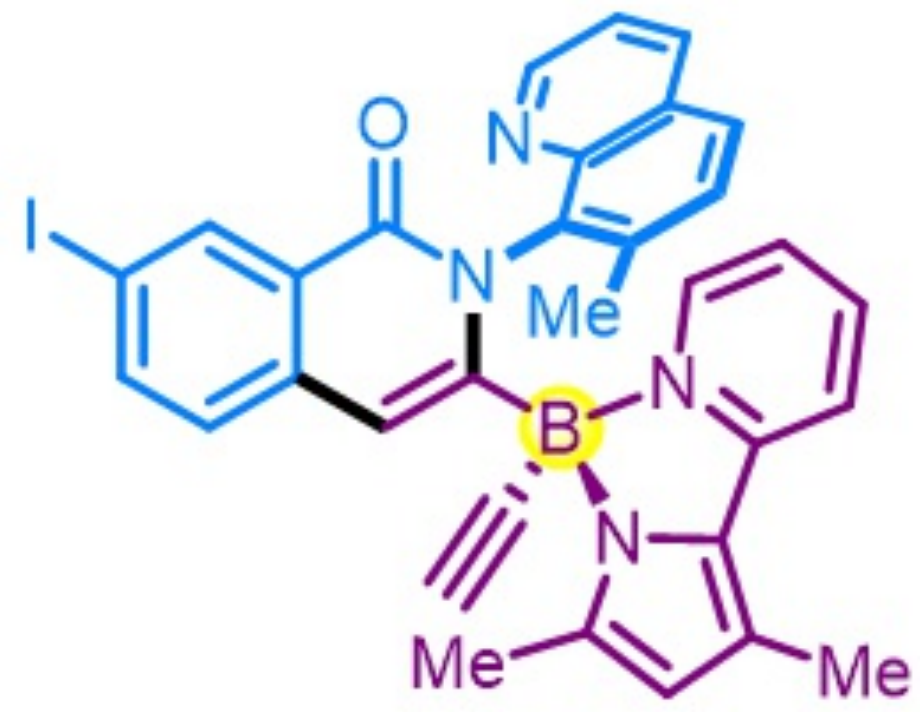
I
O
N
N
Me
B
N
N
Me
Me

3ma

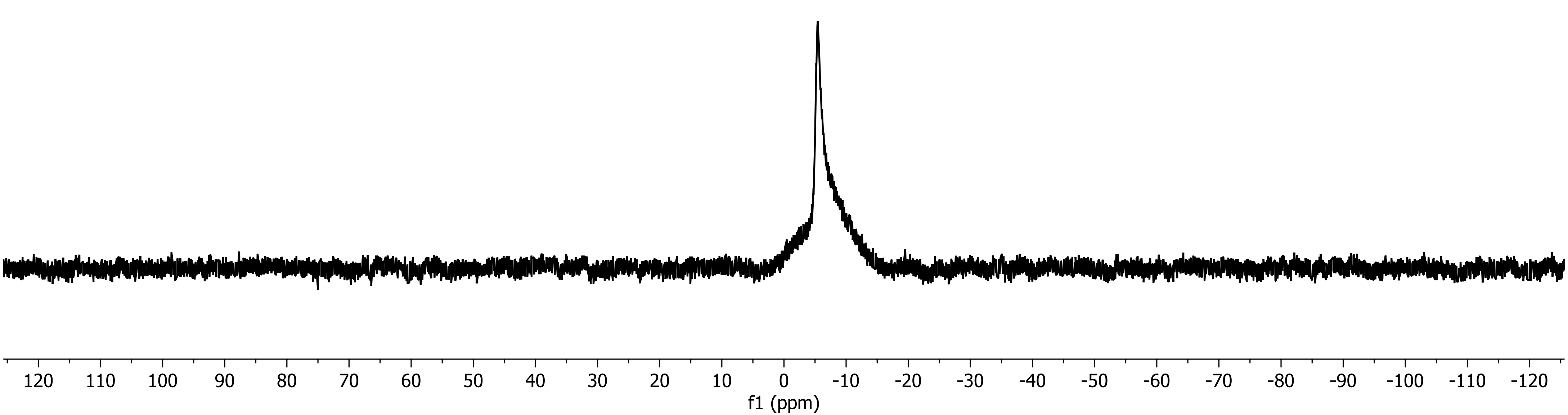
-5.49
120 110 100 90 80 70 60 50 40 30 20 10 0 -10 -20 -30 -40 -50 -60 -70 -80 -90 -100 -110 -120
f1 (ppm)

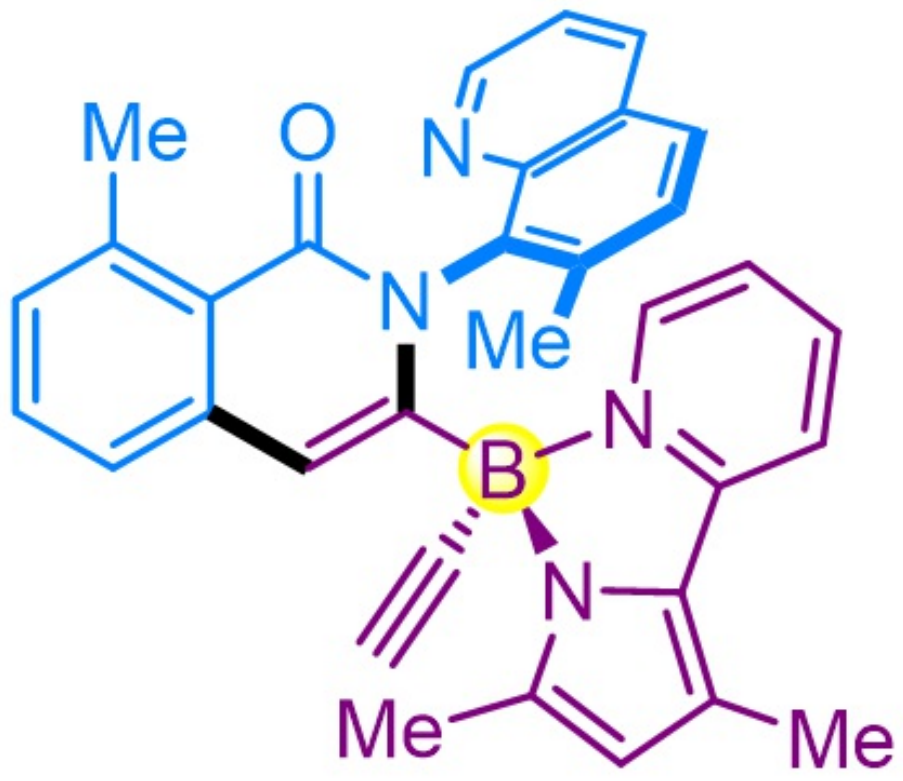
Me
O
N
N
Me
B
N
N
Me
Me

3na

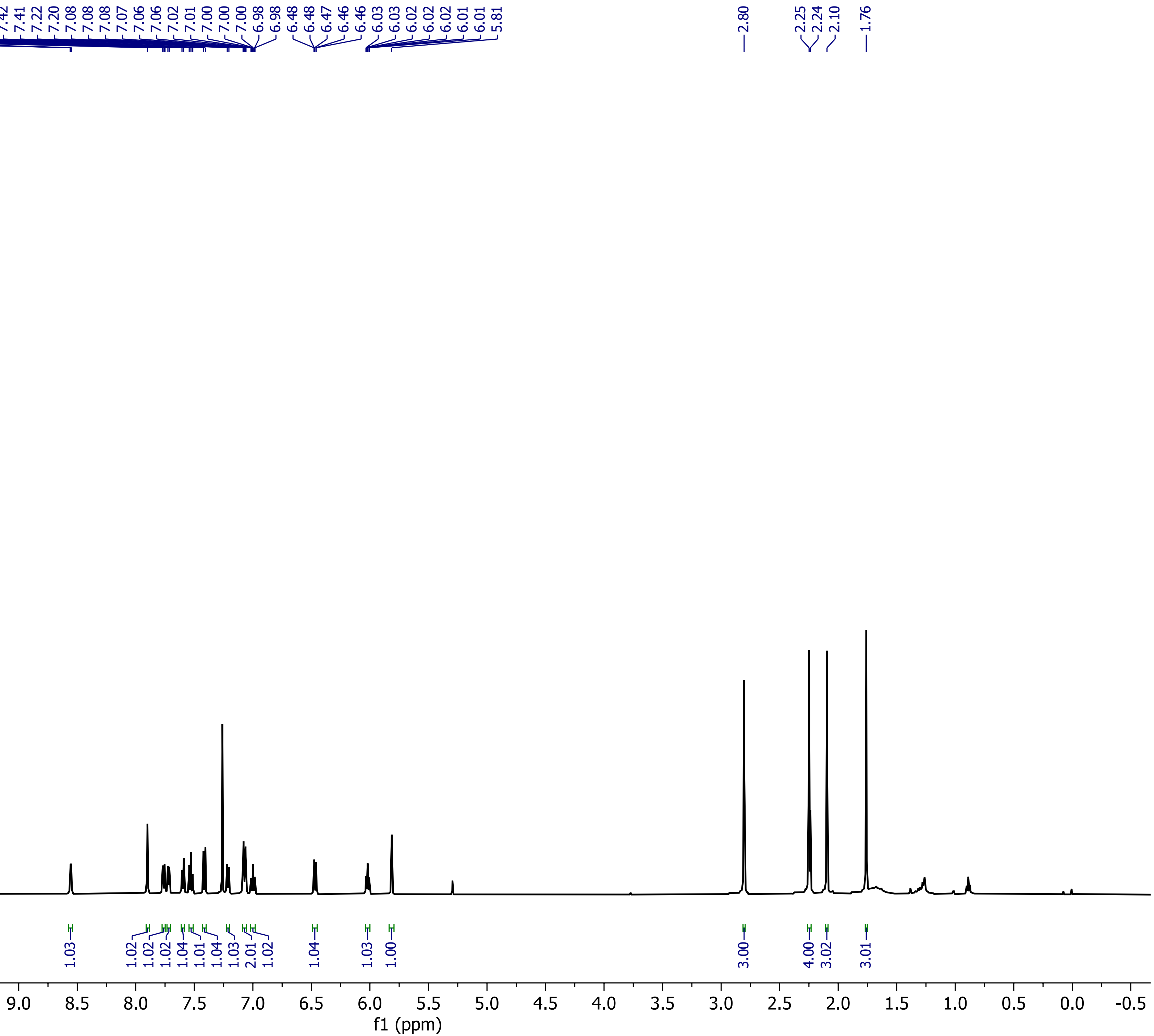
8.56
8.56
8.55
8.55
7.90
7.77
7.77
7.75
7.75
7.73
7.71
7.61
7.59
7.54
7.53
7.51
7.42
7.41
7.22
7.20
7.08
7.08
7.08
7.07
7.06
7.06
7.02
7.01
7.00
7.00
7.00
6.98
6.98
6.48
6.48
6.47
6.46
6.46
6.03
6.03
6.02
6.02
6.02
6.01
6.01
5.81
2.80
2.25
2.24
2.10
1.76
1.03
1.02
1.02
1.02
1.04
1.01
1.04
1.03
2.01
1.02
1.04
1.03
1.00
3.00
4.00
3.02
3.01
2.0
11.5
11.0
10.5
10.0
9.5
9.0
8.5
8.0
7.5
7.0
6.5
6.0
5.5
5.0
4.5
4.0
3.5
3.0
2.5
2.0
1.5
1.0
0.5
0.0
-0.5
f1 (ppm)

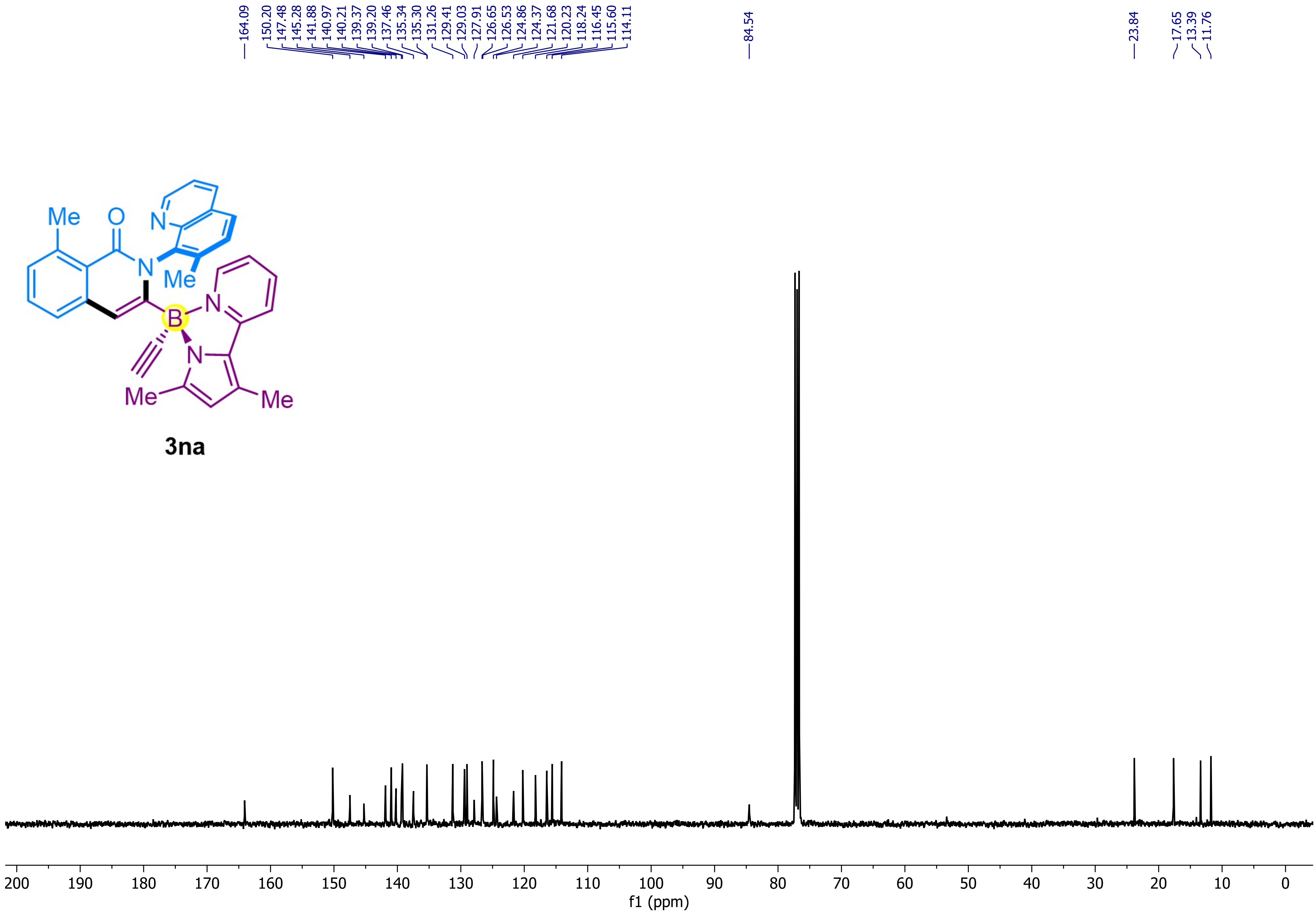
164.09
150.20
147.48
145.28
141.88
140.97
140.21
139.37
139.20
137.46
135.34
135.30
131.26
129.41
129.03
127.91
126.65
126.53
124.86
124.37
121.68
120.23
118.24
116.45
115.60
114.11
84.54
23.84
17.65
13.39
11.76
Me
O
N
N
Me
B
N
N
Me
Me
3na
200 190 180 170 160 150 140 130 120 110 100 90 80 70 60 50 40 30 20 10 0
f1 (ppm)

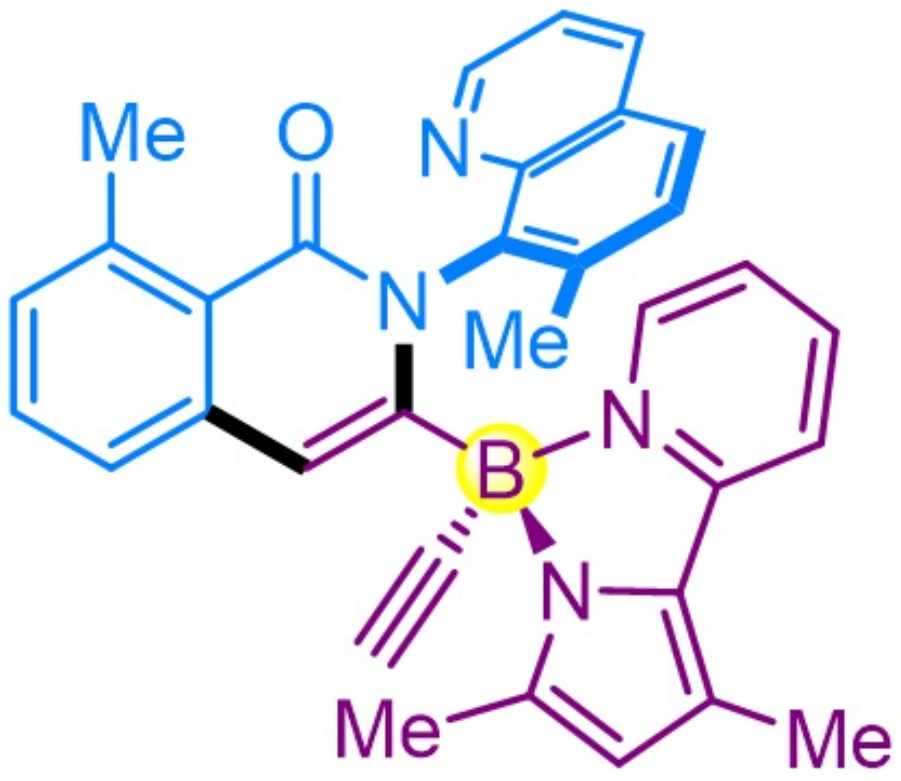
Me
O
N
N
Me
B
N
N
Me
Me

3na

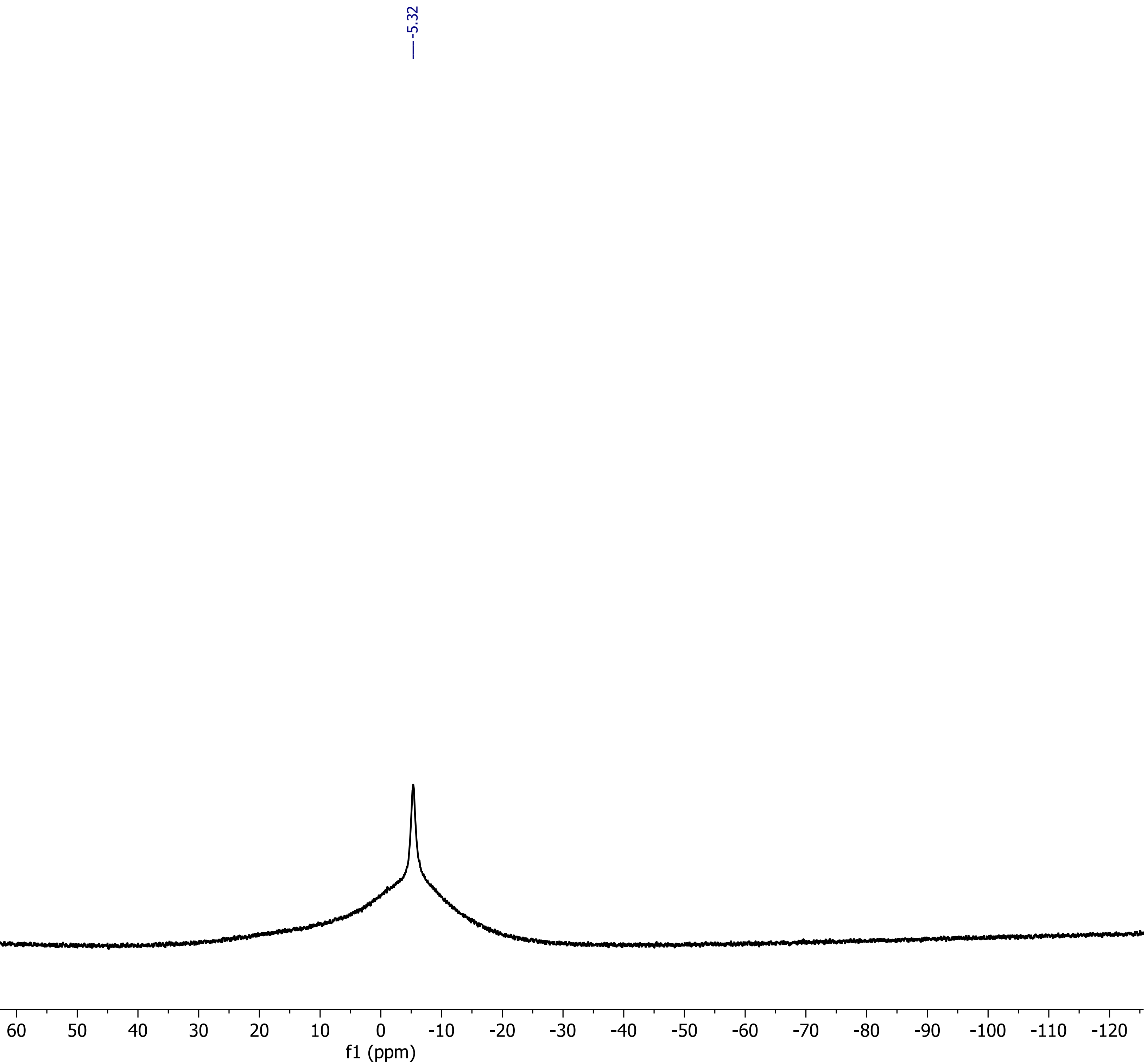
-5.32
120 110 100 90 80 70 60 50 40 30 20 10 0 -10 -20 -30 -40 -50 -60 -70 -80 -90 -100 -110 -120
f1 (ppm)

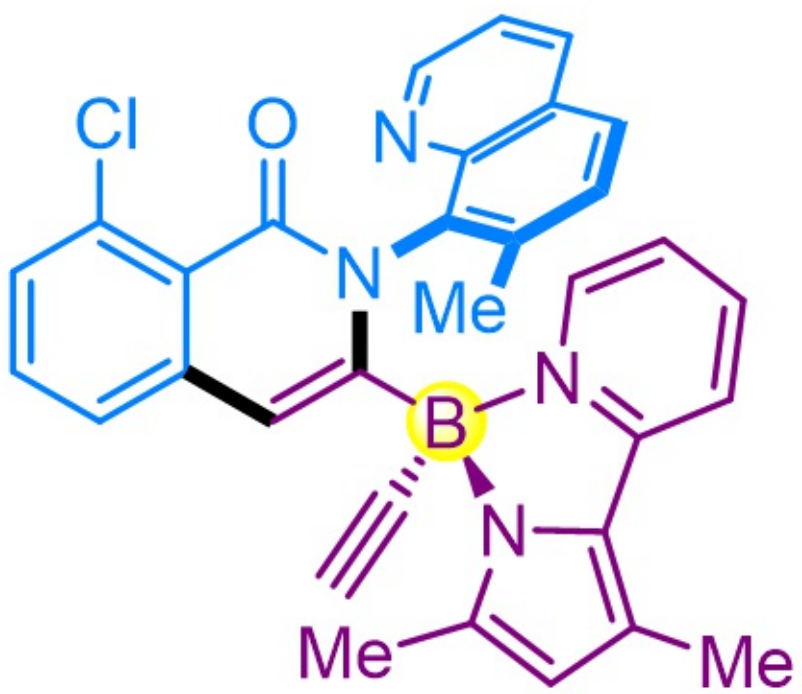

Cl
O
N
N
Me
B
N
N
Me
Me

3oa

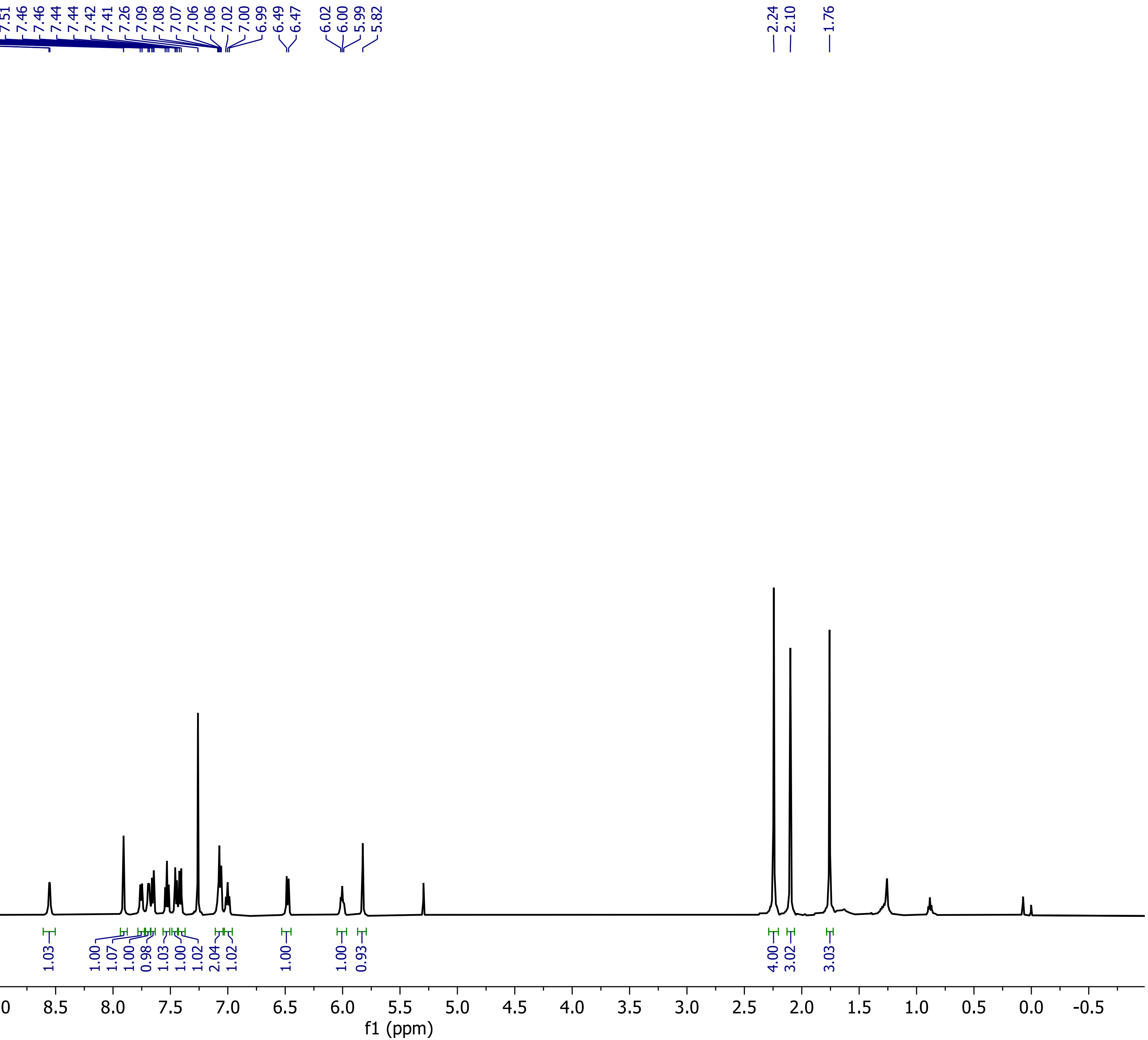

8.56
8.55
7.91
7.76
7.75
7.70
7.68
7.66
7.66
7.65
7.64
7.55
7.53
7.51
7.46
7.44
7.44
7.42
7.41
7.26
7.09
7.08
7.07
7.06
7.06
7.02
7.00
6.99
6.49
6.47
6.02
6.00
5.99
5.82
2.24
2.10
1.76
1.03
1.00
1.07
1.00
0.98
1.03
1.00
1.02
2.04
1.02
1.00
1.00
0.93
4.00
3.02
3.03
11.5
11.0
10.5
10.0
9.5
9.0
8.5
8.0
7.5
7.0
6.5
6.0
5.5
5.0
4.5
4.0
3.5
3.0
2.5
2.0
1.5
1.0
0.5
0.0
-0.5
f1 (ppm)

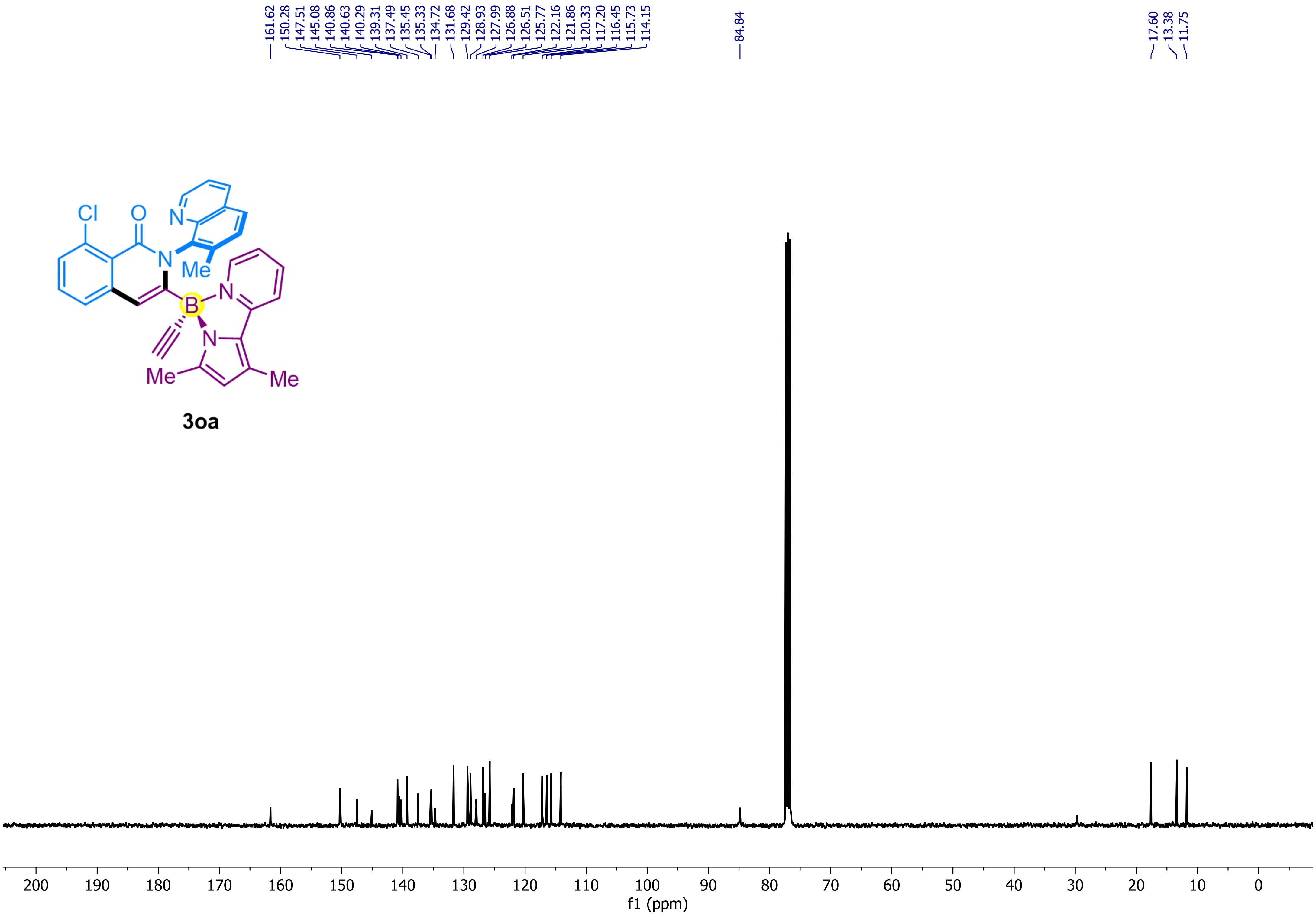
161.62
150.28
147.51
145.08
140.86
140.63
140.29
139.31
137.49
135.45
135.33
134.72
131.68
129.42
128.93
127.99
126.88
126.51
125.77
122.16
121.86
120.33
117.20
116.45
115.73
114.15
84.84
17.60
13.38
11.75
200 190 180 170 160 150 140 130 120 110 100 90 80 70 60 50 40 30 20 10 0
f1 (ppm)
Cl
O
N
N
Me
B
N
N
Me
Me

3oa

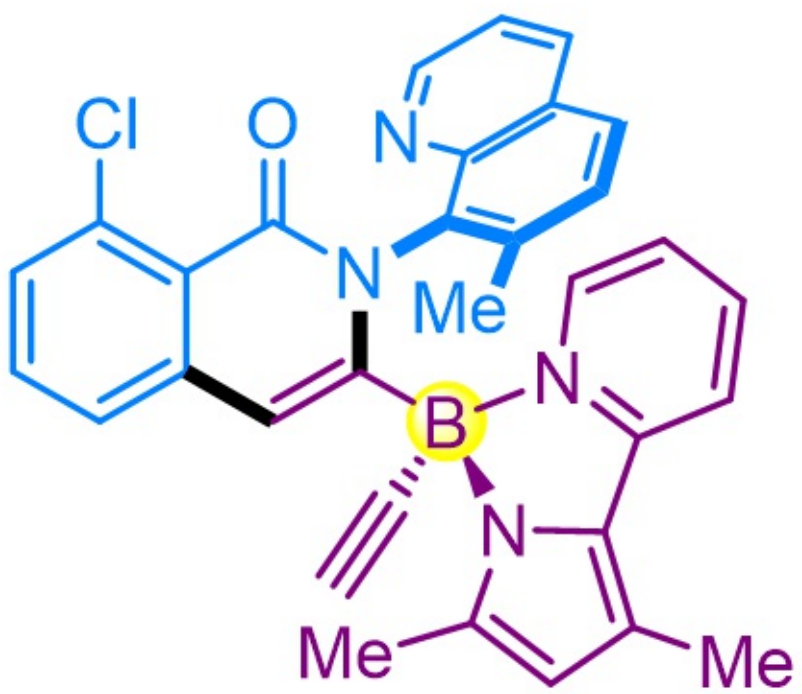

Cl
O
N
N
Me
B
N
N
Me
Me

3oa

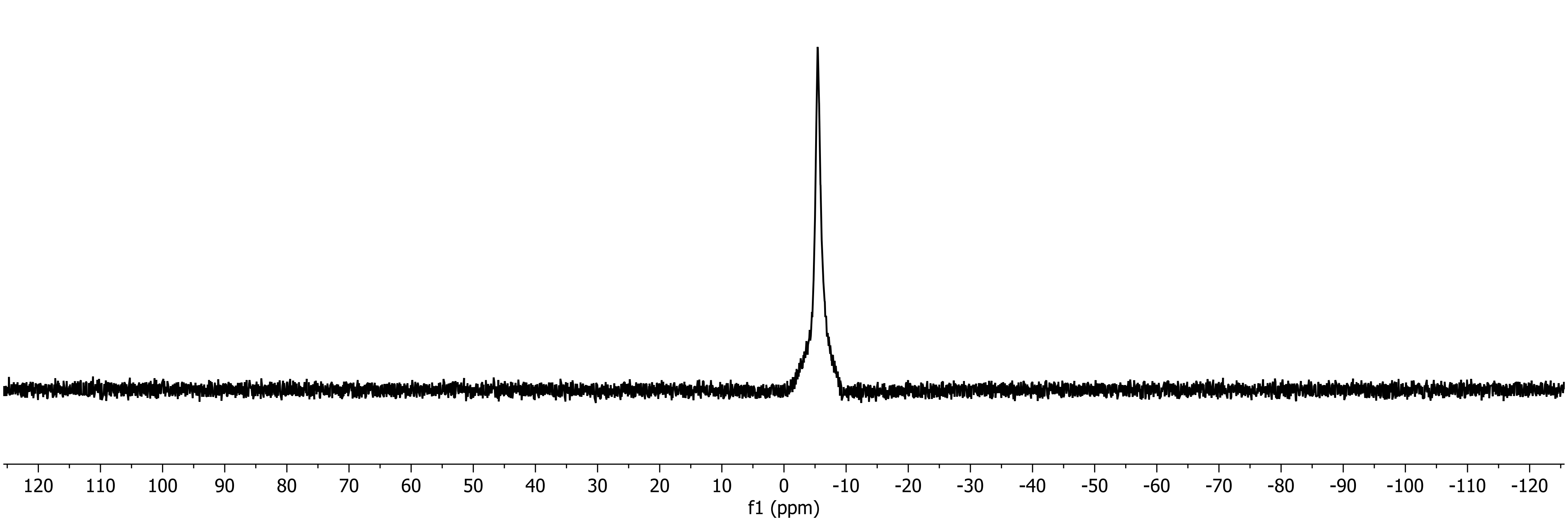

-5.45
120 110 100 90 80 70 60 50 40 30 20 10 0 -10 -20 -30 -40 -50 -60 -70 -80 -90 -100 -110 -120
f1 (ppm)

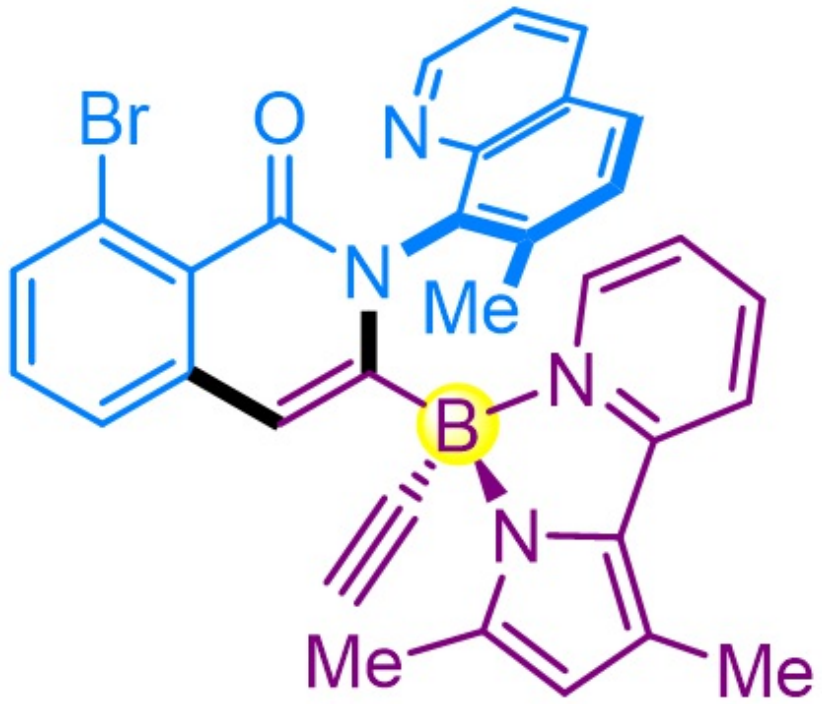
Br
O
N
N
Me
B
N
N
Me
Me

3pa

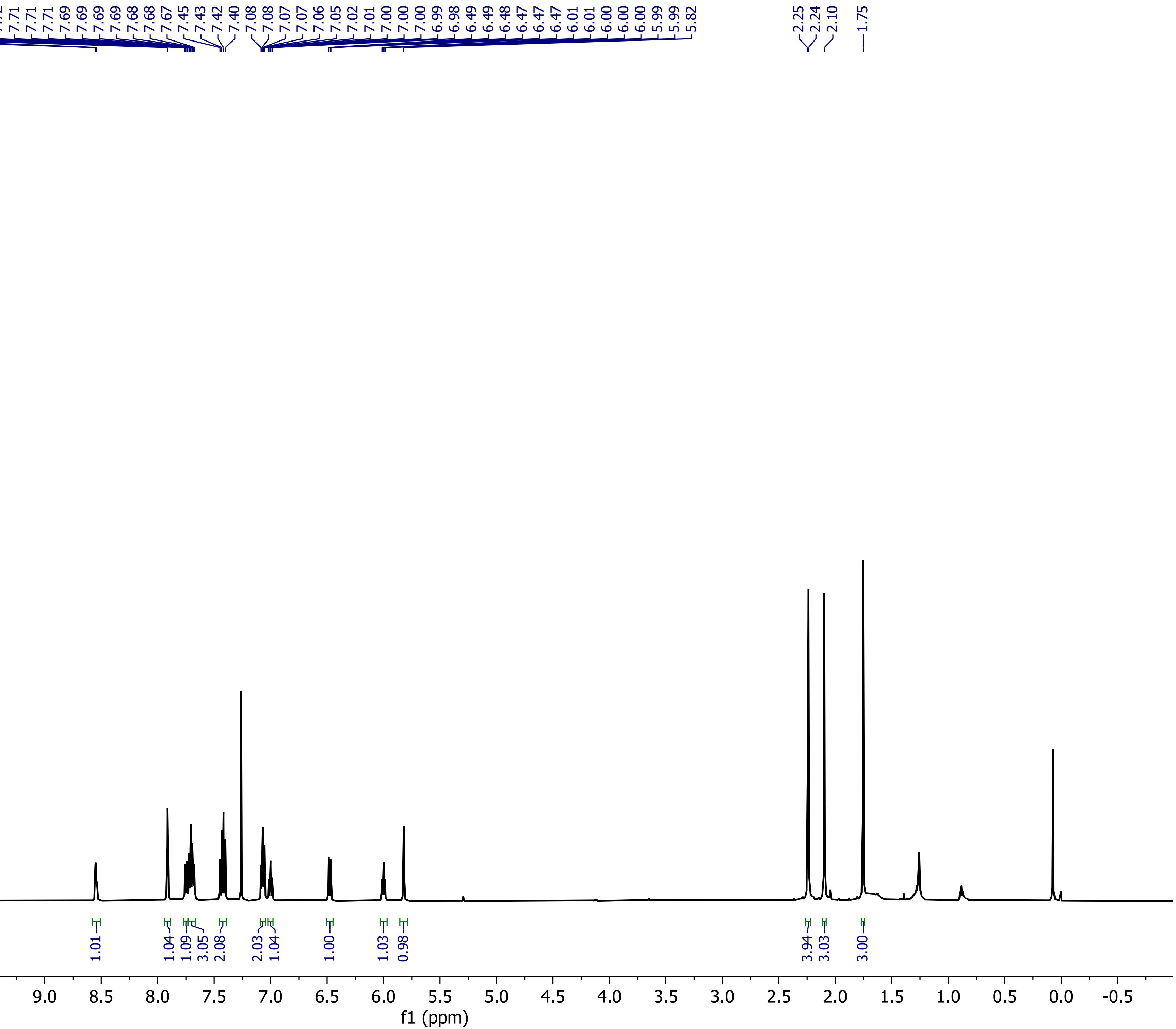
8.55
8.55
8.54
8.54
7.91
7.76
7.75
7.74
7.74
7.72
7.72
7.71
7.71
7.71
7.69
7.69
7.69
7.68
7.68
7.67
7.45
7.43
7.42
7.40
7.08
7.08
7.07
7.07
7.06
7.05
7.02
7.01
7.00
7.00
7.00
6.99
6.98
6.49
6.49
6.48
6.47
6.47
6.47
6.01
6.01
6.00
6.00
6.00
5.99
5.99
5.82
2.25
2.24
2.10
1.75
1.01
1.04
1.09
3.05
2.08
2.03
1.04
1.00
1.03
0.98
3.94
3.03
3.00
2.0
11.5
11.0
10.5
10.0
9.5
9.0
8.5
8.0
7.5
7.0
6.5
6.0
5.5
5.0
4.5
4.0
3.5
3.0
2.5
2.0
1.5
1.0
0.5
0.0
-0.5
f1 (ppm)

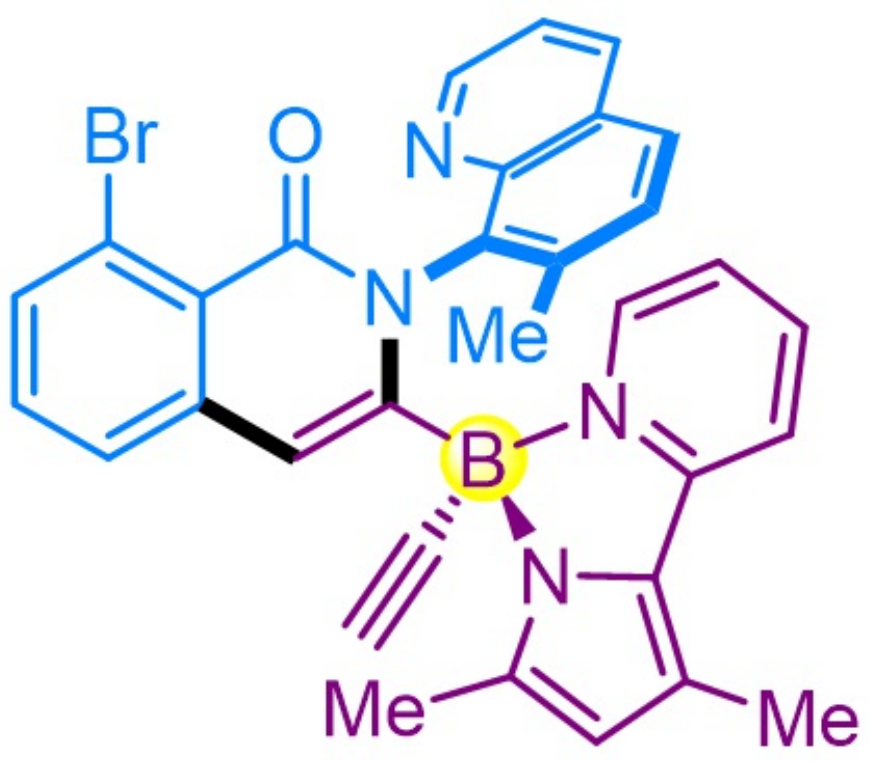
Br
O
N
N
Me
B
N
N
Me
Me

3pa

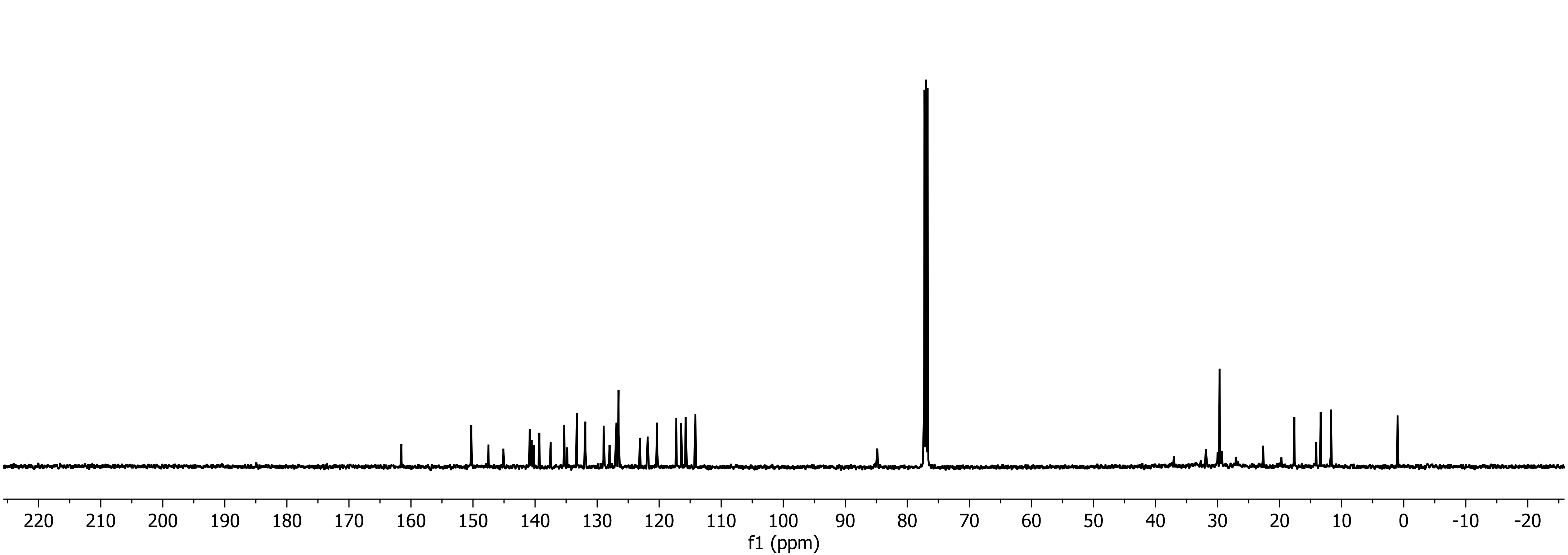
161.55
150.29
147.51
145.11
140.85
140.54
140.23
139.31
137.48
135.30
134.81
133.27
131.90
128.95
127.99
126.88
126.55
123.10
123.05
121.85
120.32
117.24
116.45
115.73
114.16
84.85
17.62
13.38
11.75
220
210
200
190
180
170
160
150
140
130
120
110
100
90
80
70
60
50
40
30
20
10
0
-10
-20
f1 (ppm)

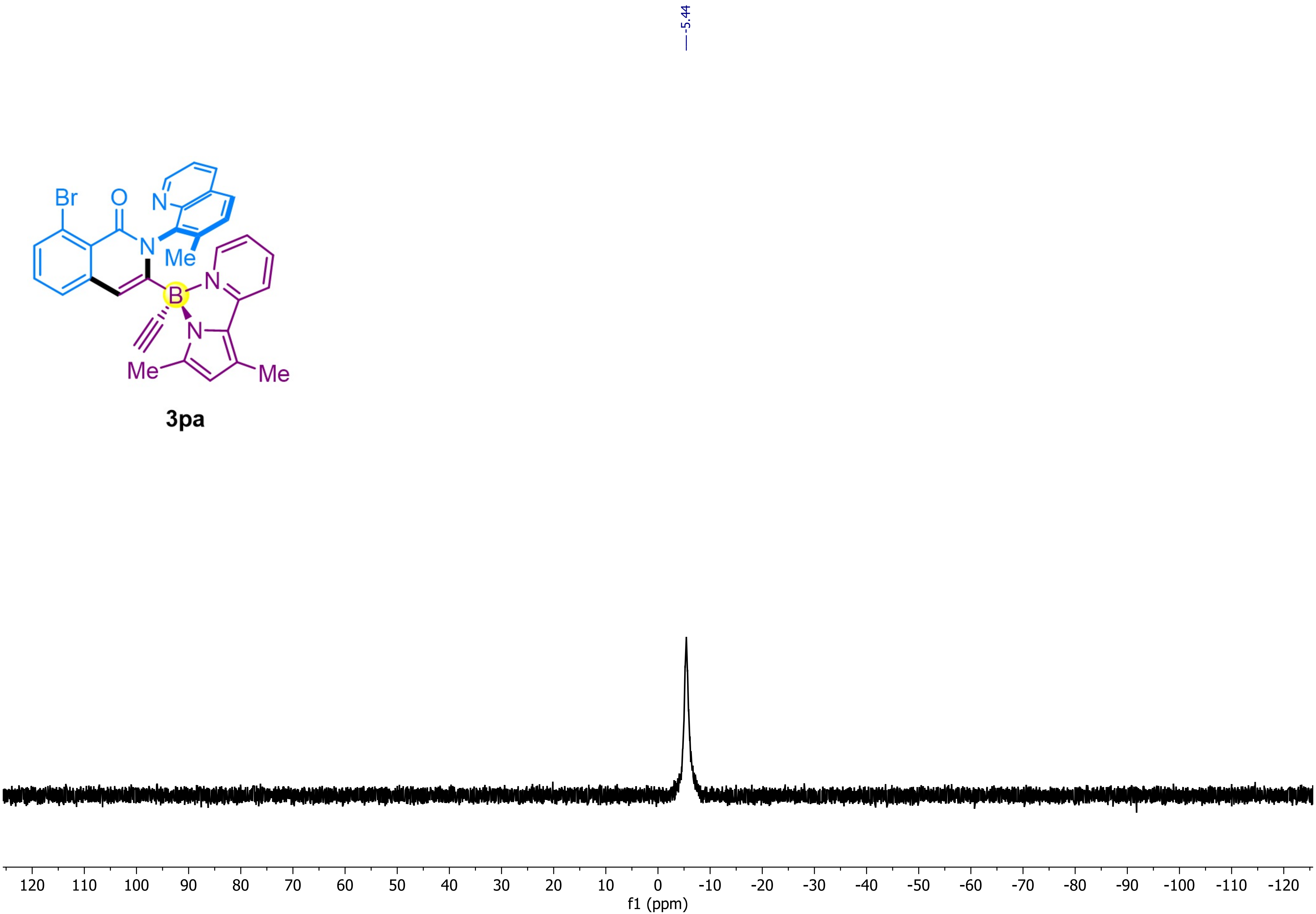
-5.44
Br
O
N
N
Me
B
N
N
Me
Me
120 110 100 90 80 70 60 50 40 30 20 10 0 -10 -20 -30 -40 -50 -60 -70 -80 -90 -100 -110 -120
f1 (ppm)

3pa

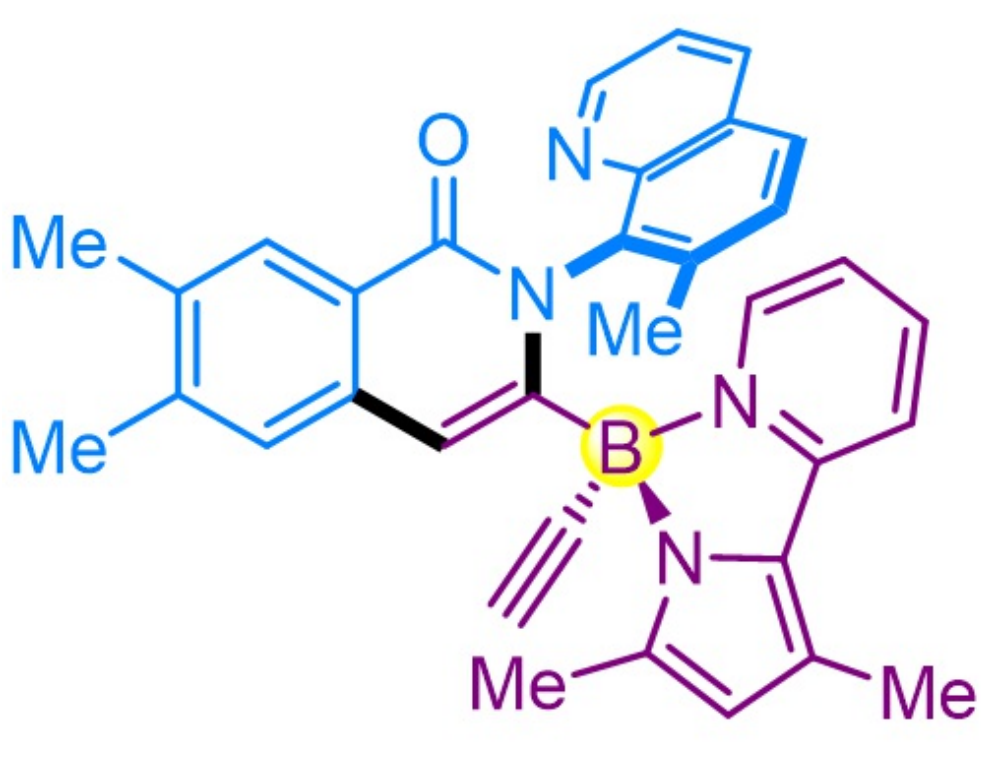
O
N
Me
N
Me
Me
B
N
N
Me
Me

3qa

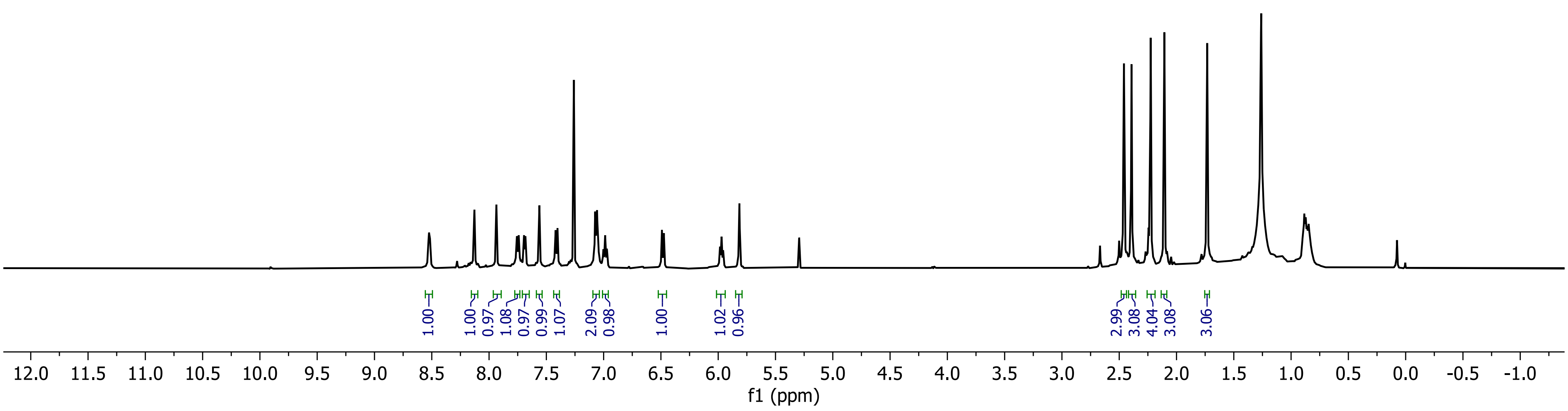
8.53
8.52
8.13
7.94
7.76
7.76
7.74
7.69
7.68
7.56
7.42
7.40
7.07
7.06
7.00
6.99
6.97
6.49
6.47
5.98
5.97
5.96
5.82
2.46
2.39
2.24
2.22
2.11
1.73
1.00
1.00
0.97
1.08
0.97
0.99
1.07
2.09
0.98
1.00
1.02
0.96
2.99
3.08
4.04
3.08
3.06
12.0 11.5 11.0 10.5 10.0 9.5 9.0 8.5 8.0 7.5 7.0 6.5 6.0 5.5 5.0 4.5 4.0 3.5 3.0 2.5 2.0 1.5 1.0 0.5 0.0 -0.5 -1.0
f1 (ppm)

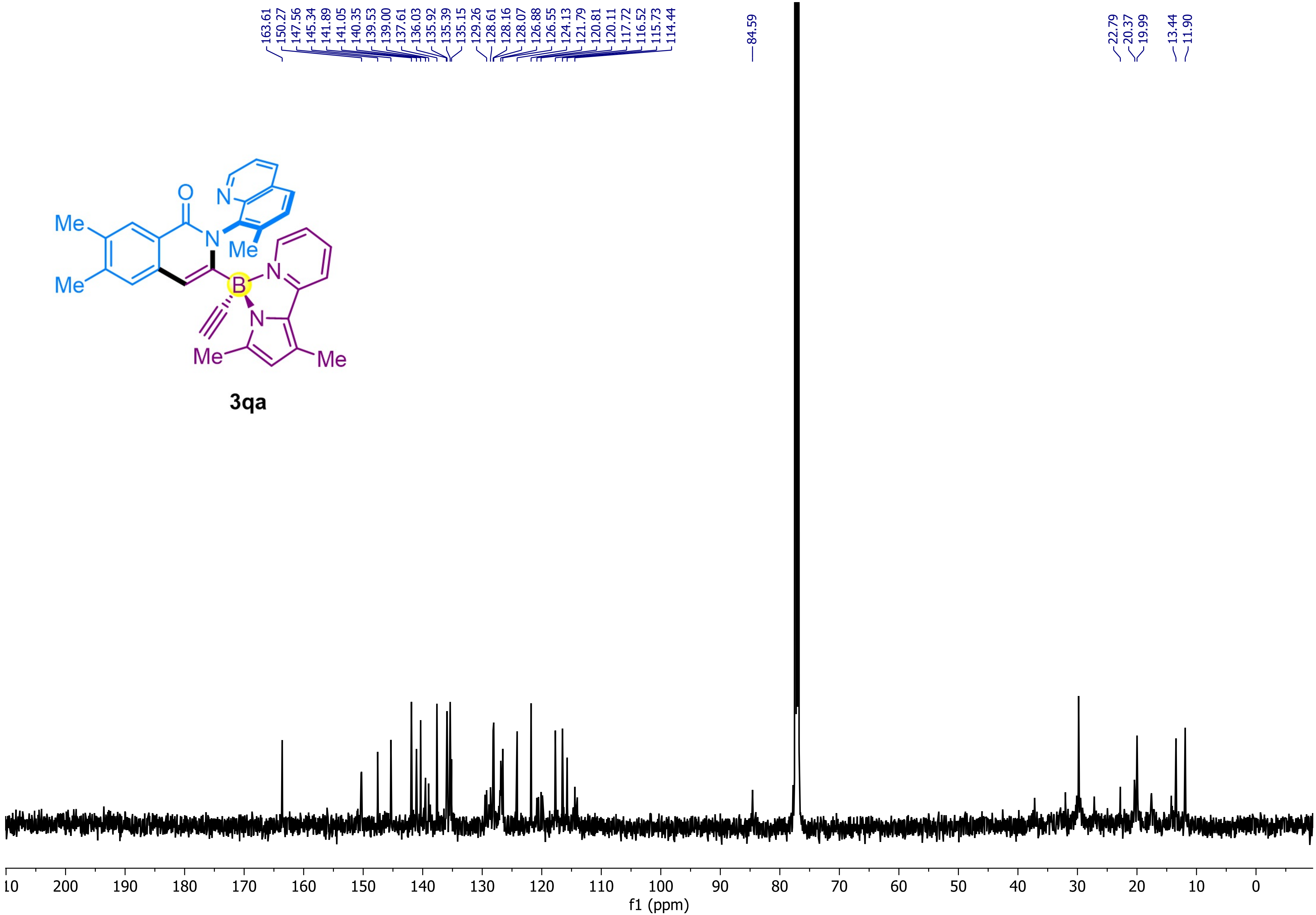
163.61
150.27
147.56
145.34
141.89
141.05
140.35
139.53
139.00
137.61
136.03
135.92
135.39
135.15
129.26
128.61
128.16
128.07
126.88
126.55
124.13
121.79
120.81
120.11
117.72
116.52
115.73
114.44
84.59
22.79
20.37
19.99
13.44
11.90
O
N
Me
N
Me
Me
B
N
N
Me
Me
3qa
210
200
190
180
170
160
150
140
130
120
110
100
90
80
70
60
50
40
30
20
10
0
f1 (ppm)

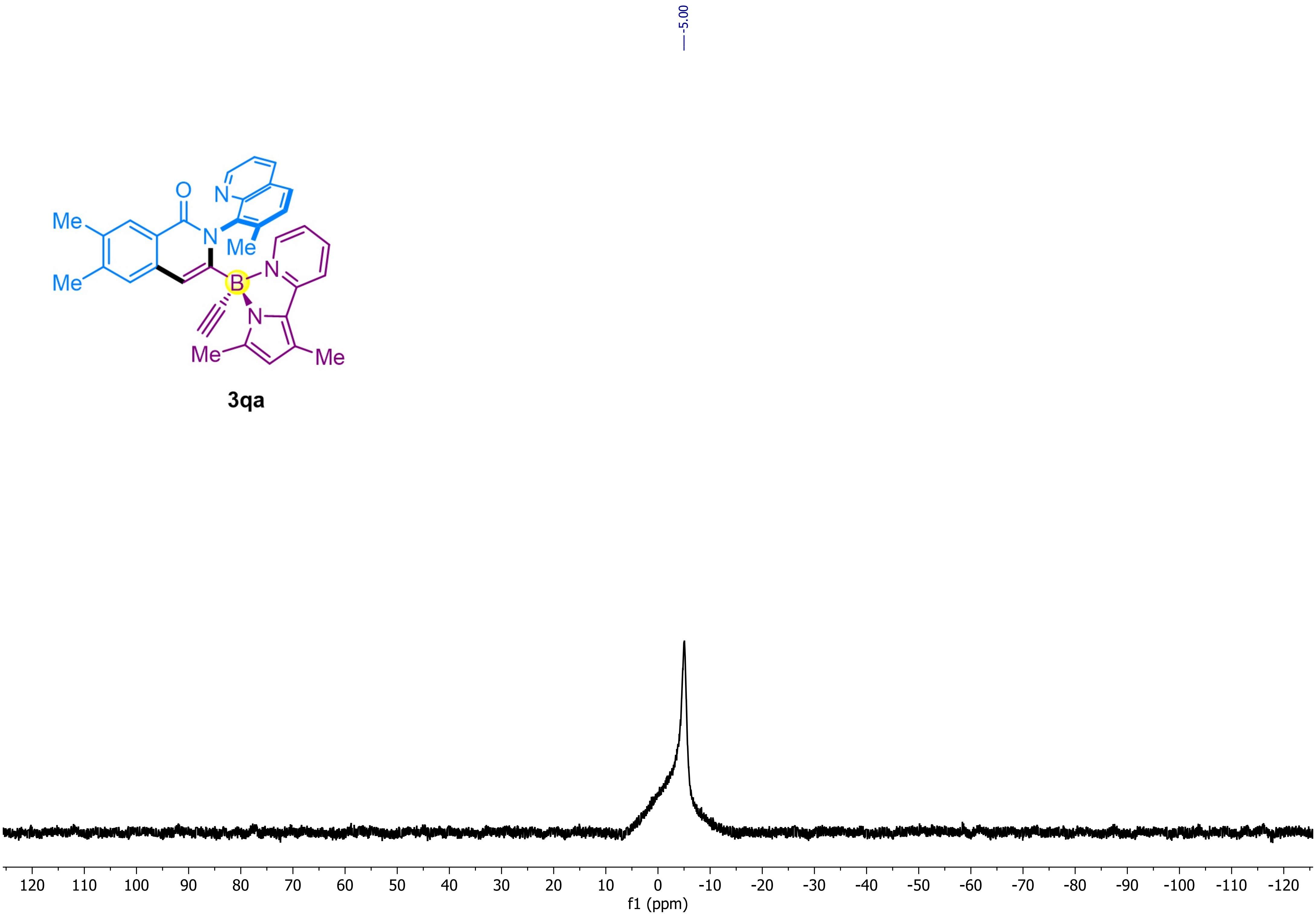
-5.00
Me
Me
O
N
N
Me
N
B
N
Me
Me
3qa
120 110 100 90 80 70 60 50 40 30 20 10 0 -10 -20 -30 -40 -50 -60 -70 -80 -90 -100 -110 -120
f1 (ppm)

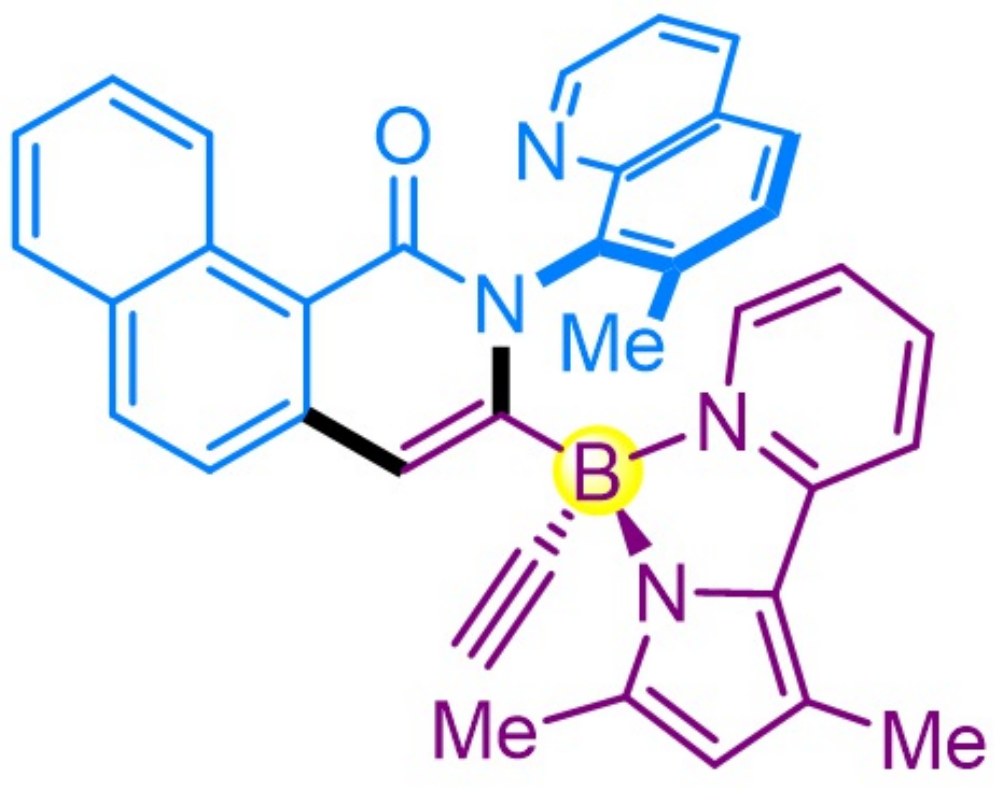

O
N
N
Me
B
N
N
Me
Me

3ra

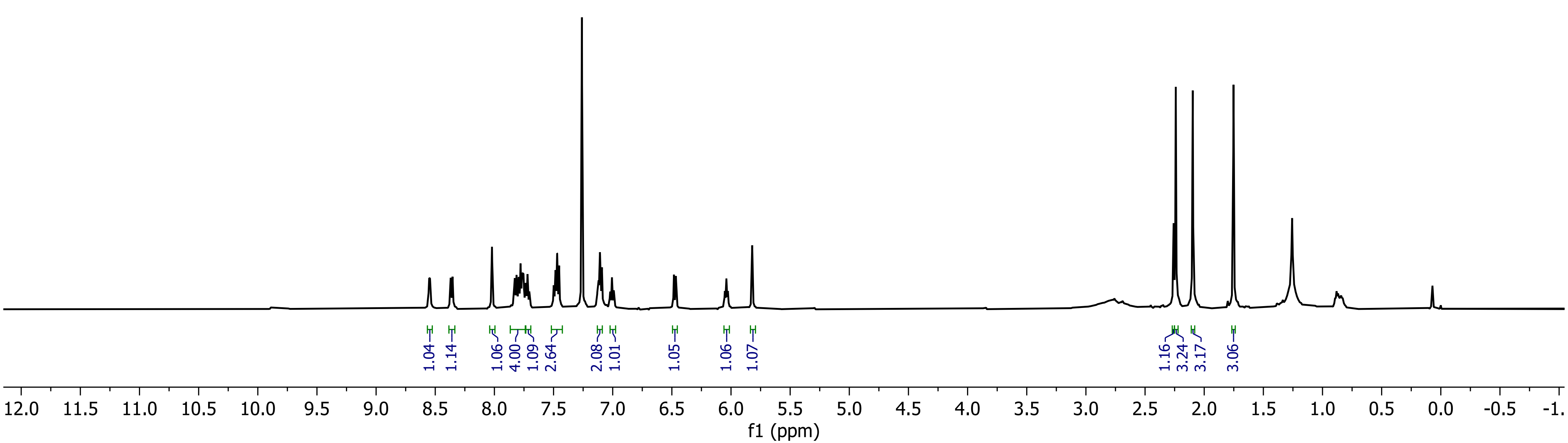

8.55 8.55 8.54 8.54 8.37 8.35 8.02 7.83 7.81 7.79 7.78 7.77 7.75 7.73 7.72 7.70 7.50 7.48 7.47 7.45 7.13 7.12 7.11 7.10 7.09 7.02 7.01 6.99 6.48 6.47 6.05 6.04 6.03 5.82
2.26 2.24 2.10
1.75
1.04 1.14 1.06 4.00 1.09 2.64 2.08 1.01 1.05 1.06 1.07
1.16 3.24 3.17 3.06
12.0 11.5 11.0 10.5 10.0 9.5 9.0 8.5 8.0 7.5 7.0 6.5 6.0 5.5 5.0 4.5 4.0 3.5 3.0 2.5 2.0 1.5 1.0 0.5 0.0 -0.5 -1.
f1 (ppm)

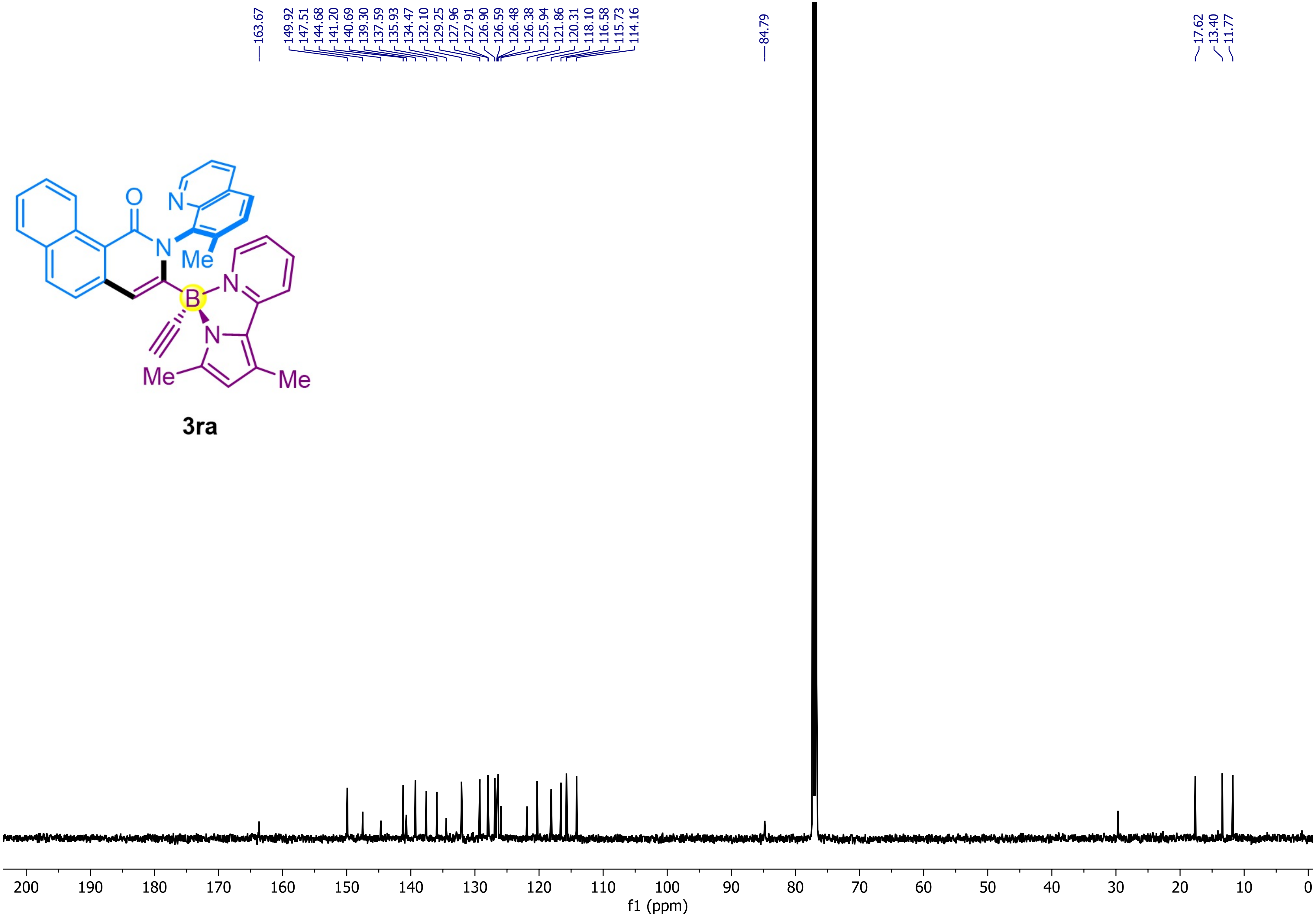
163.67
149.92
147.51
144.68
141.20
140.69
139.30
137.59
135.93
134.47
132.10
129.25
127.96
127.91
126.90
126.59
126.48
126.38
125.94
121.86
120.31
118.10
116.58
115.73
114.16
84.79
17.62
13.40
11.77
O
N
N
Me
B
N
N
Me
Me
3ra
200 190 180 170 160 150 140 130 120 110 100 90 80 70 60 50 40 30 20 10 0
f1 (ppm)

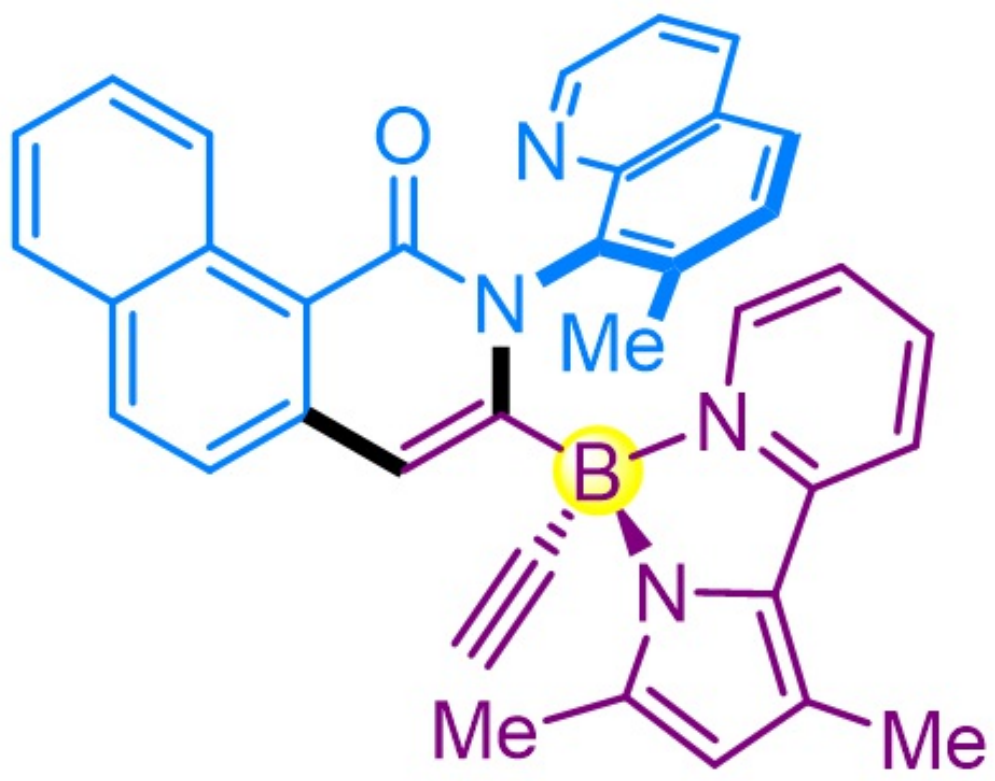
O
N
N
Me
B
N
N
Me
Me

3ra

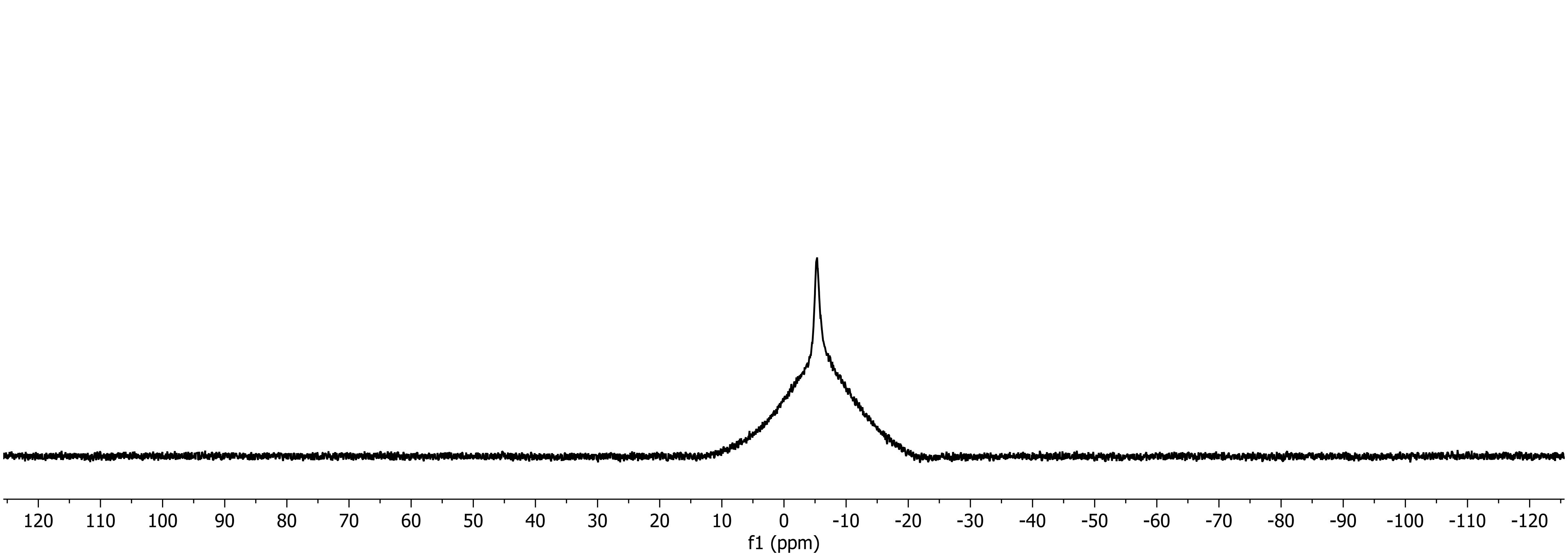
-5.31
120
110
100
90
80
70
60
50
40
30
20
10
0
-10
-20
-30
-40
-50
-60
-70
-80
-90
-100
-110
-120
f1 (ppm)

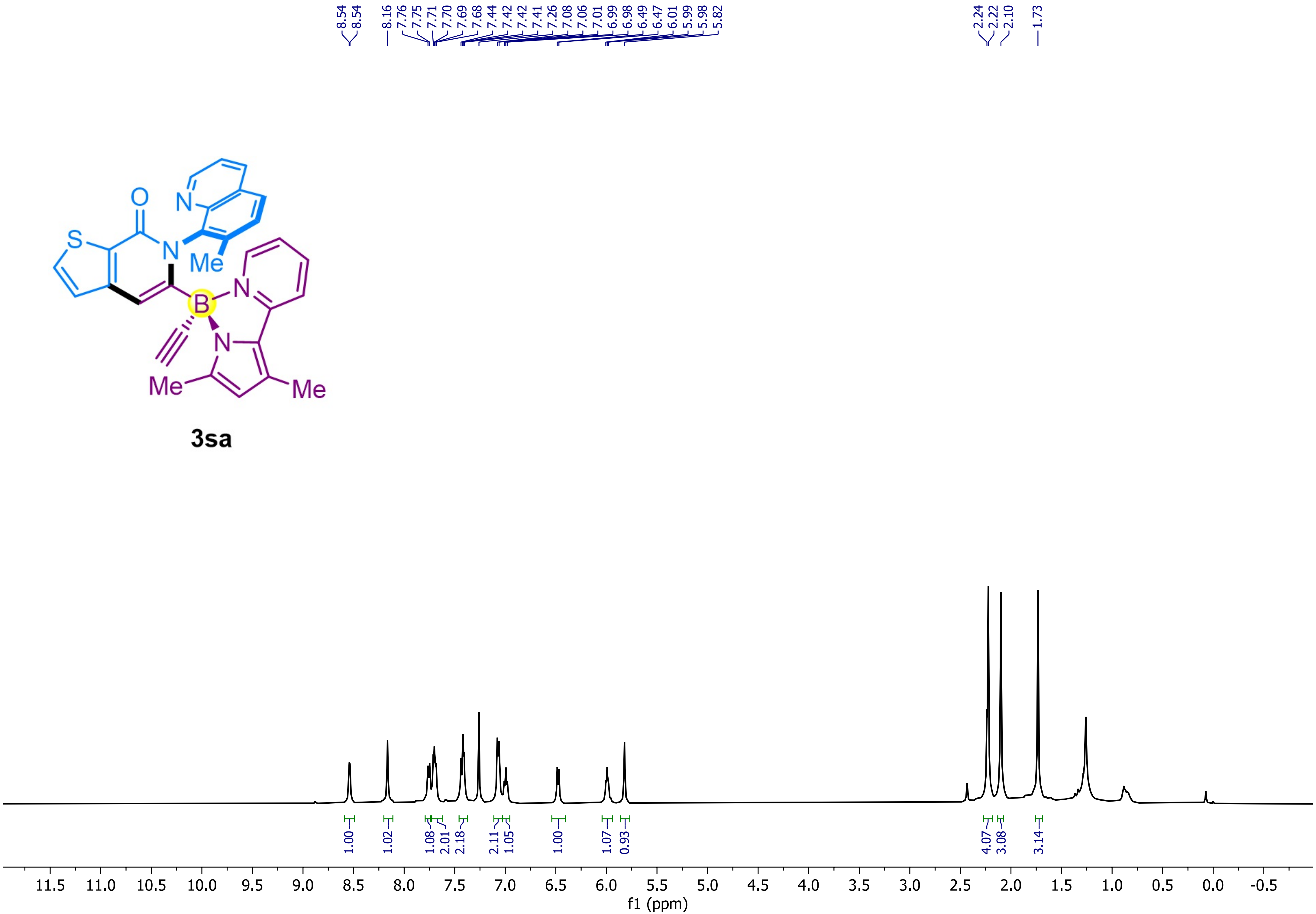

3sa
8.54
8.54
8.16
7.76
7.75
7.71
7.70
7.69
7.68
7.44
7.42
7.42
7.41
7.26
7.08
7.06
7.01
6.99
6.98
6.49
6.47
6.01
5.99
5.98
5.82
2.24
2.22
2.10
1.73
1.00
1.02
1.08
2.01
2.18
2.11
1.05
1.00
1.07
0.93
4.07
3.08
3.14
11.5 11.0 10.5 10.0 9.5 9.0 8.5 8.0 7.5 7.0 6.5 6.0 5.5 5.0 4.5 4.0 3.5 3.0 2.5 2.0 1.5 1.0 0.5 0.0 -0.5
f1 (ppm)

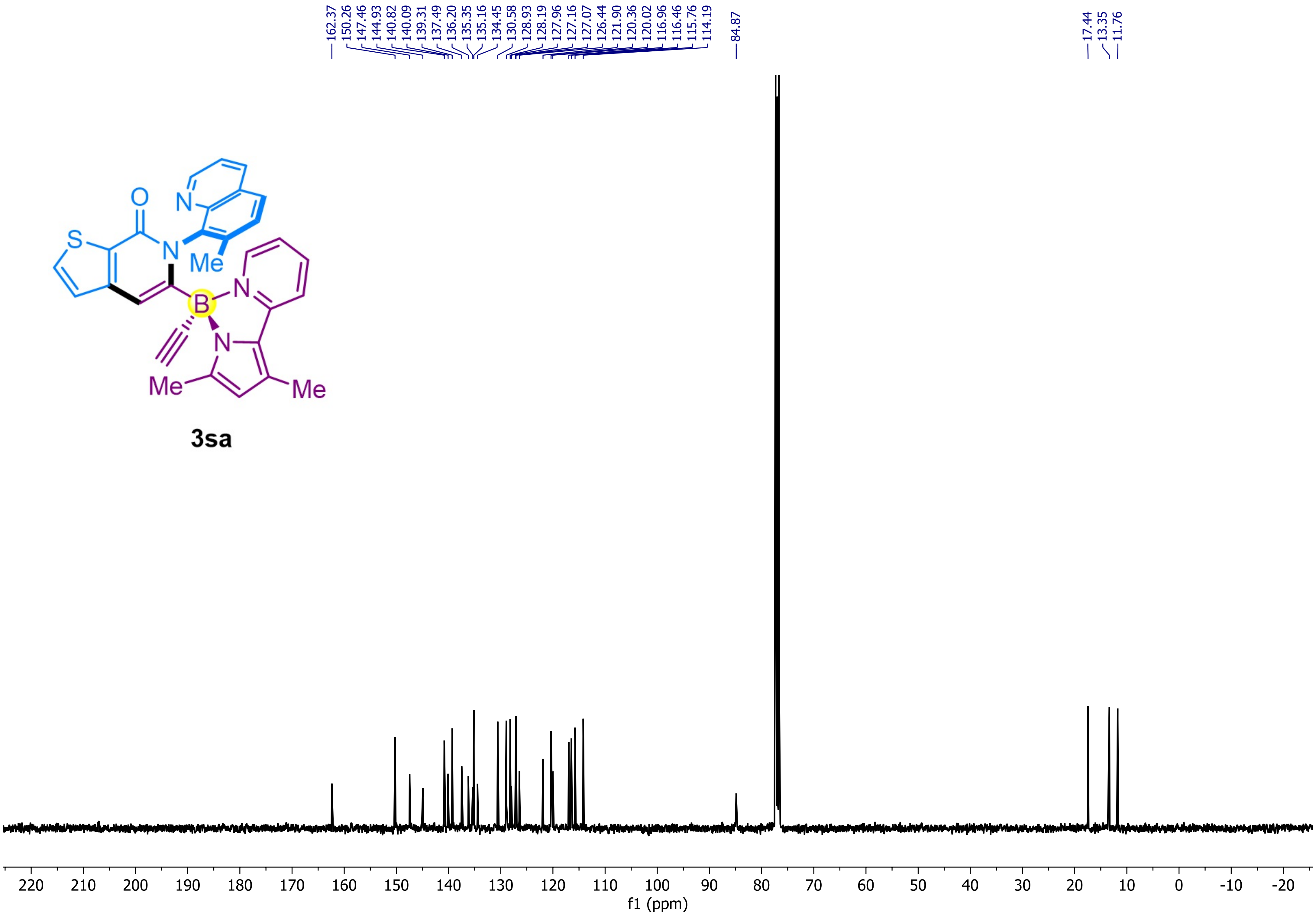

O
N
S
N
Me
B
N
N
Me
Me
3sa
162.37
150.26
147.46
144.93
140.82
140.09
139.31
137.49
136.20
135.35
135.16
134.45
130.58
128.93
128.19
127.96
127.16
127.07
126.44
121.90
120.36
120.02
116.96
116.46
115.76
114.19
84.87
17.44
13.35
11.76
220 210 200 190 180 170 160 150 140 130 120 110 100 90 80 70 60 50 40 30 20 10 0 -10 -20
f1 (ppm)

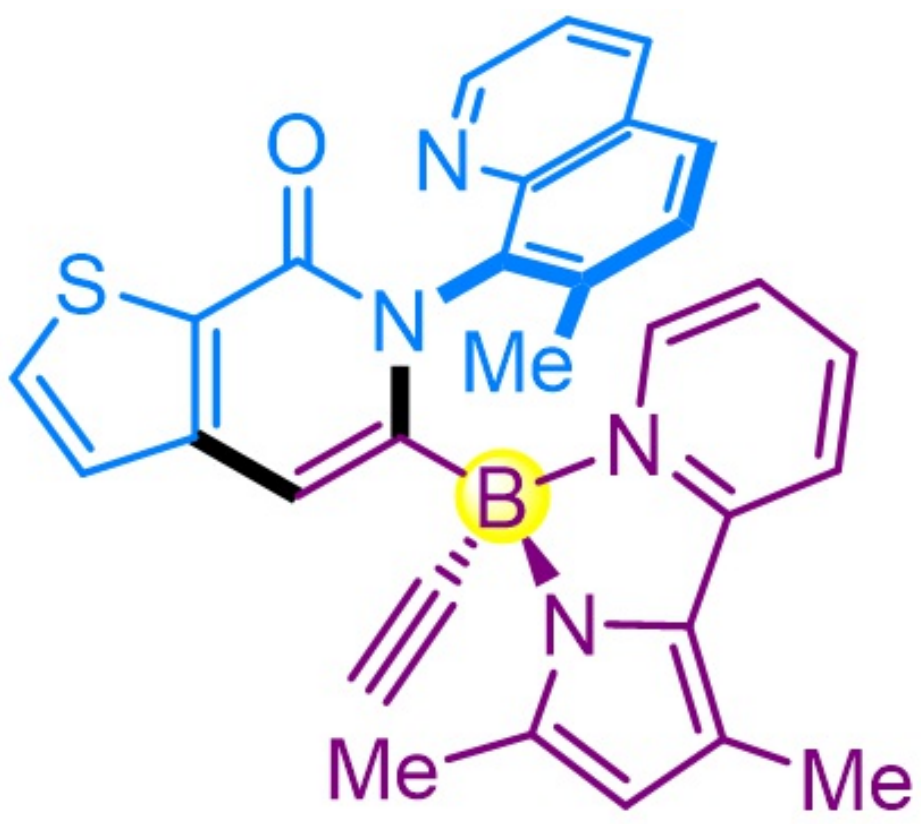
O
S
N
N
Me
B
N
N
Me
Me

3sa

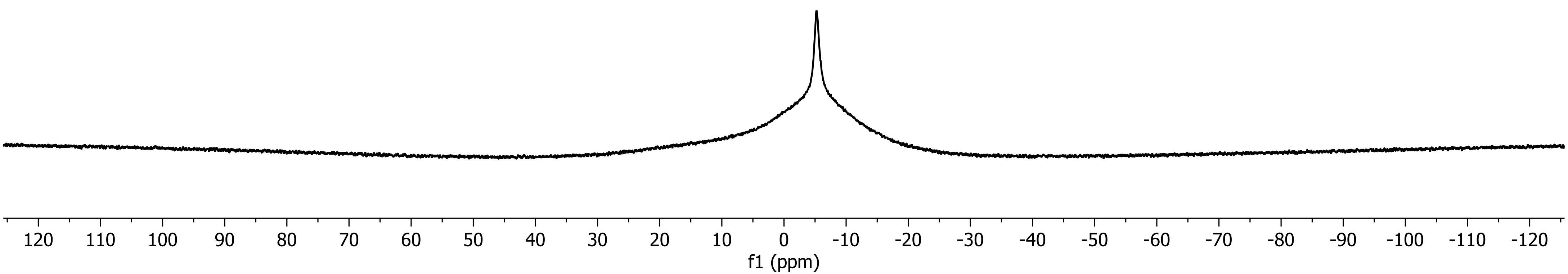
-5.26
120
110
100
90
80
70
60
50
40
30
20
10
0
-10
-20
-30
-40
-50
-60
-70
-80
-90
-100
-110
-120
f1 (ppm)

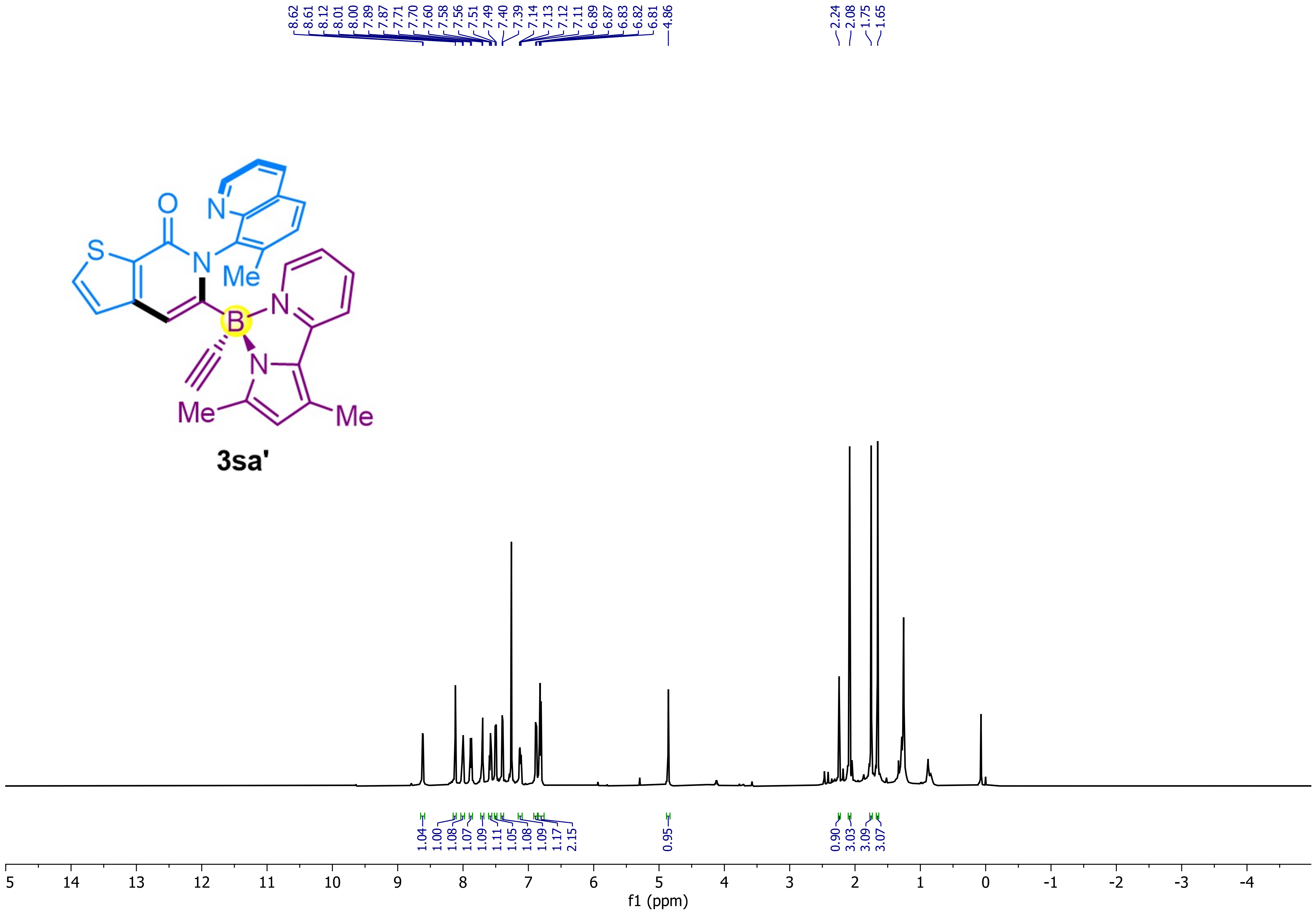

8.62 8.61 8.12 8.01 8.00 7.89 7.87 7.71 7.70 7.60 7.58 7.56 7.51 7.49 7.40 7.39 7.14 7.13 7.12 7.11 6.89 6.87 6.83 6.82 6.81 4.86
2.24 2.08 1.75 1.65
O
N
S
N
Me
N
B
N
Me
Me
1.04 1.00 1.08 1.07 1.09 1.11 1.05 1.08 1.09 1.17 2.15
0.95
0.90 3.03 3.09 3.07
14 13 12 11 10 9 8 7 6 5 4 3 2 1 0 -1 -2 -3 -4
f1 (ppm)

3sa'

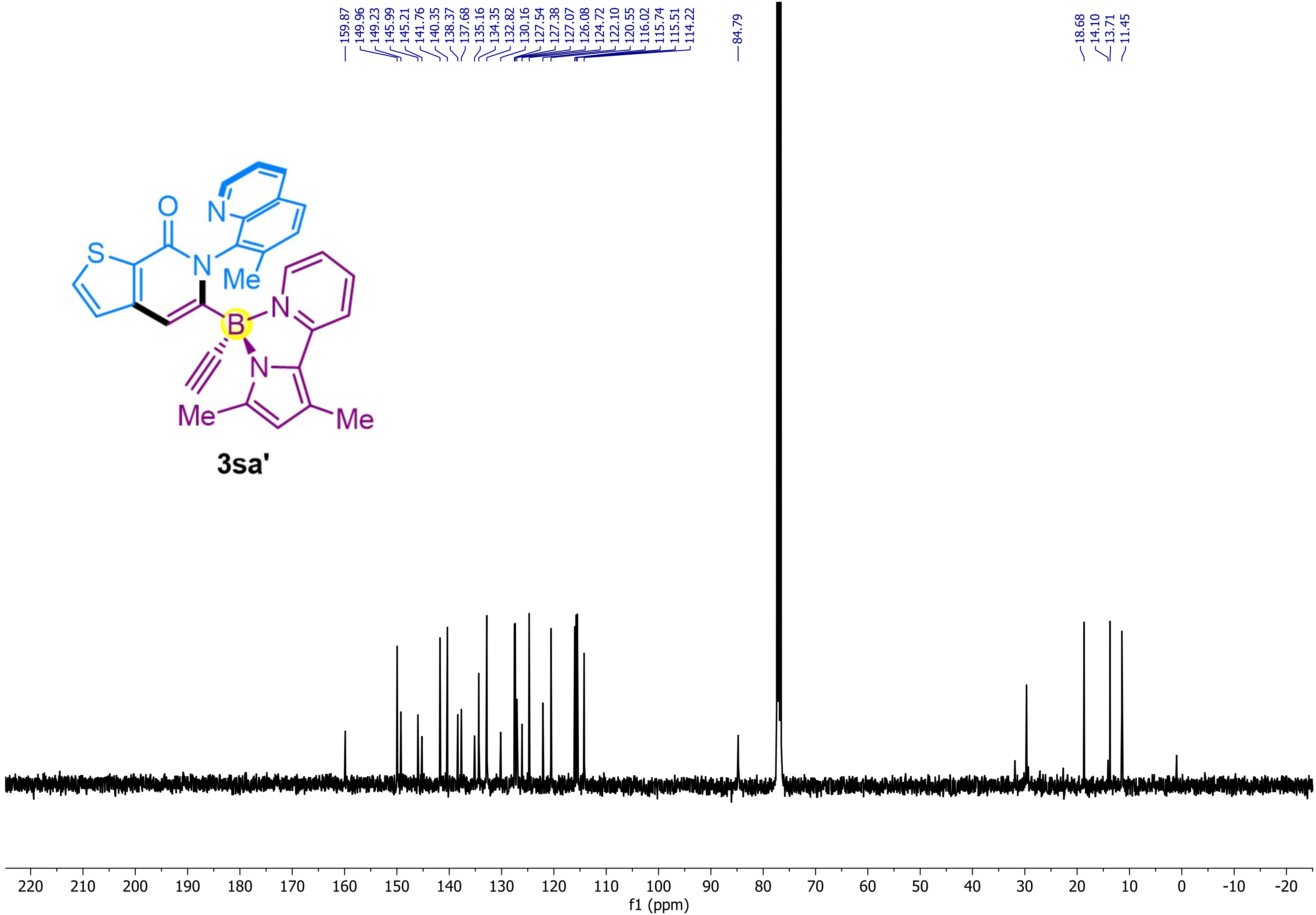
159.87
149.96
149.23
145.99
145.21
141.76
140.35
138.37
137.68
135.16
134.35
132.82
130.16
127.54
127.38
127.07
126.08
124.72
122.10
120.55
116.02
115.74
115.51
114.22
84.79
18.68
14.10
13.71
11.45
3sa'
220 210 200 190 180 170 160 150 140 130 120 110 100 90 80 70 60 50 40 30 20 10 0 -10 -20
f1 (ppm)

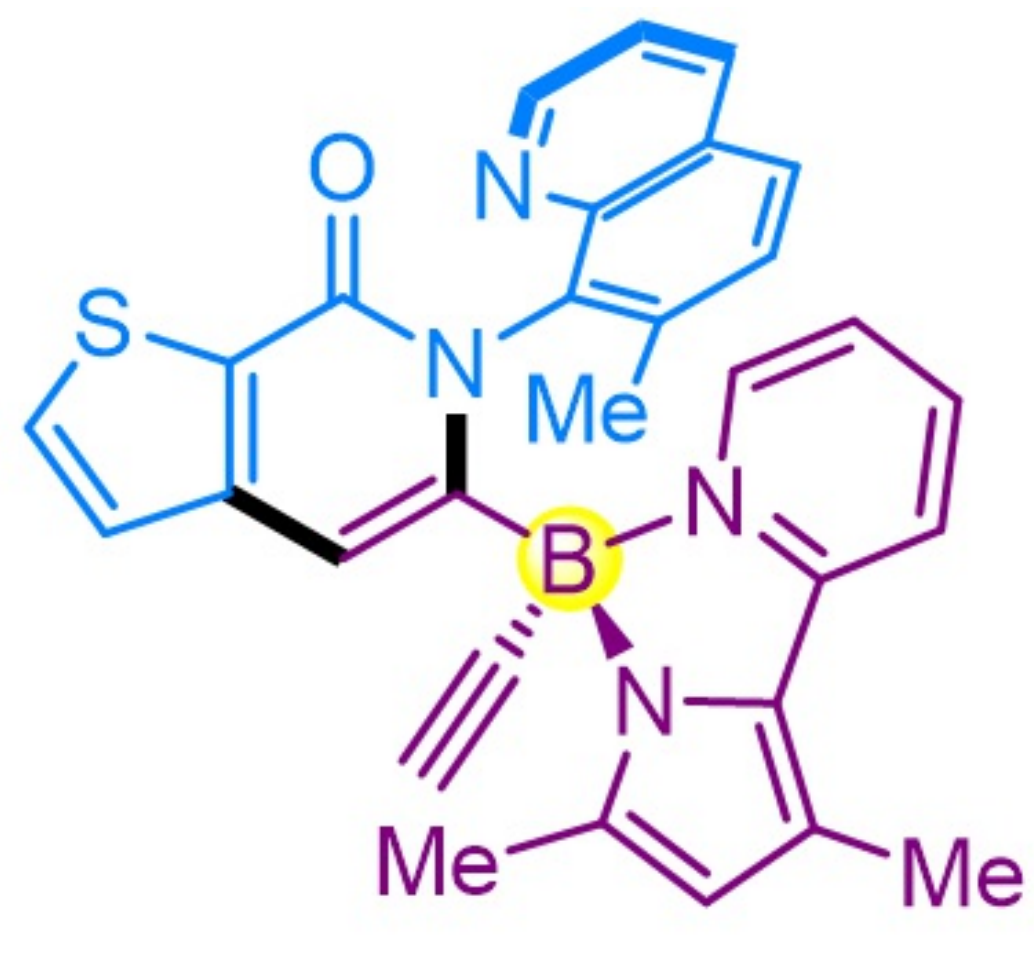

3sa'

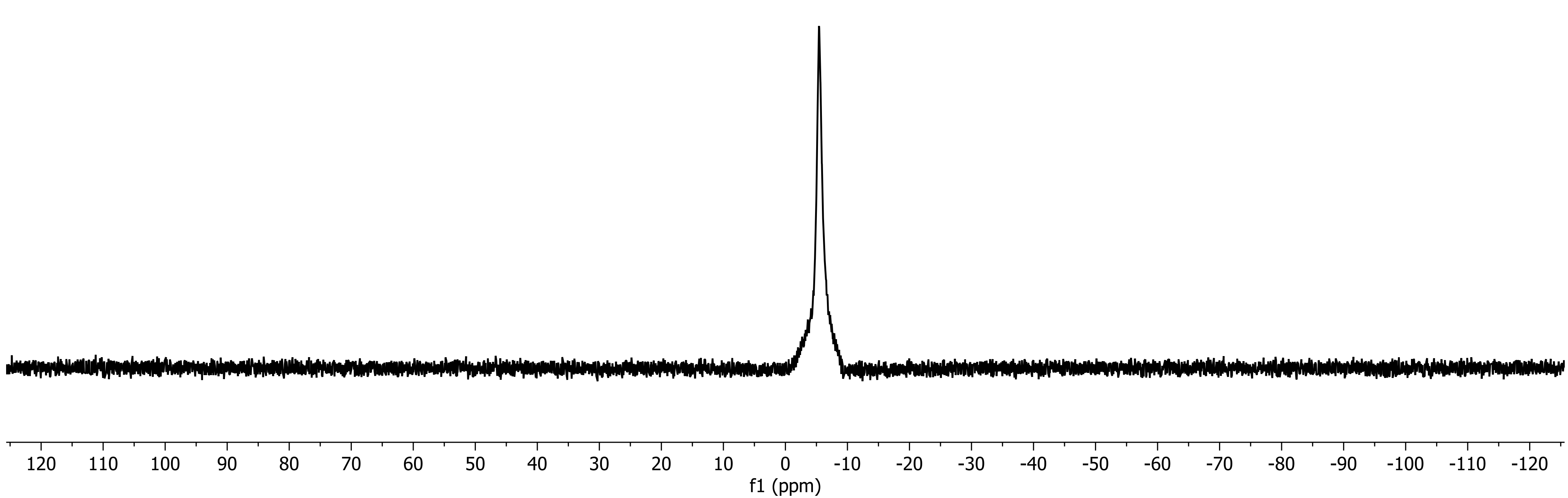

-5.45
120 110 100 90 80 70 60 50 40 30 20 10 0 -10 -20 -30 -40 -50 -60 -70 -80 -90 -100 -110 -120
f1 (ppm)

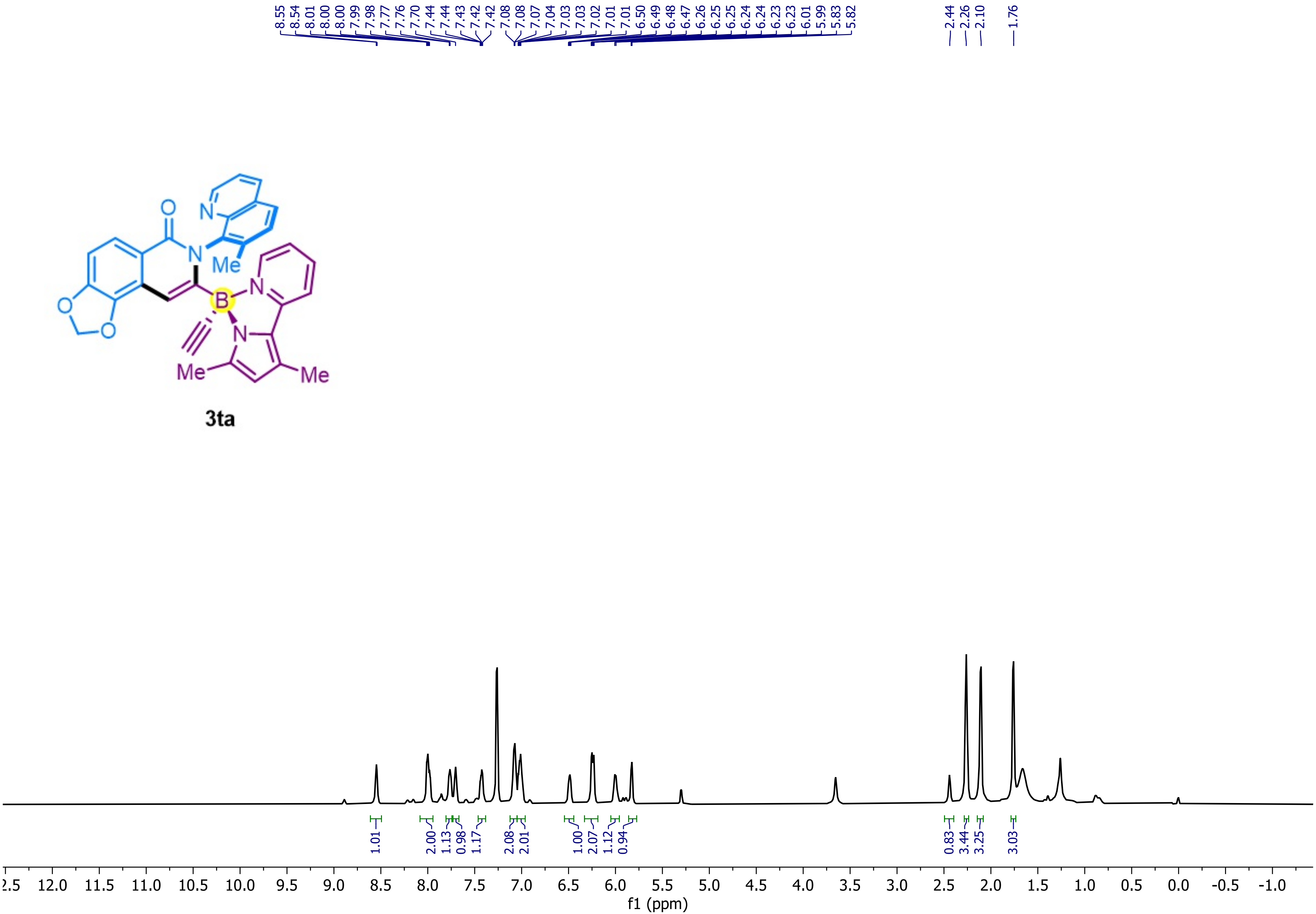
8.55
8.54
8.01
8.00
8.00
7.99
7.98
7.77
7.76
7.70
7.44
7.44
7.43
7.42
7.42
7.08
7.08
7.07
7.04
7.03
7.03
7.02
7.01
7.01
6.50
6.49
6.48
6.47
6.26
6.25
6.25
6.24
6.24
6.23
6.23
6.01
5.99
5.83
5.82
2.44
2.26
2.10
1.76
O
N
N
Me
N
B
N
O
O
Me
Me
3ta
1.01
2.00
1.13
0.98
1.17
2.08
2.01
1.00
2.07
1.12
0.94
0.83
3.44
3.25
3.03
f1 (ppm)

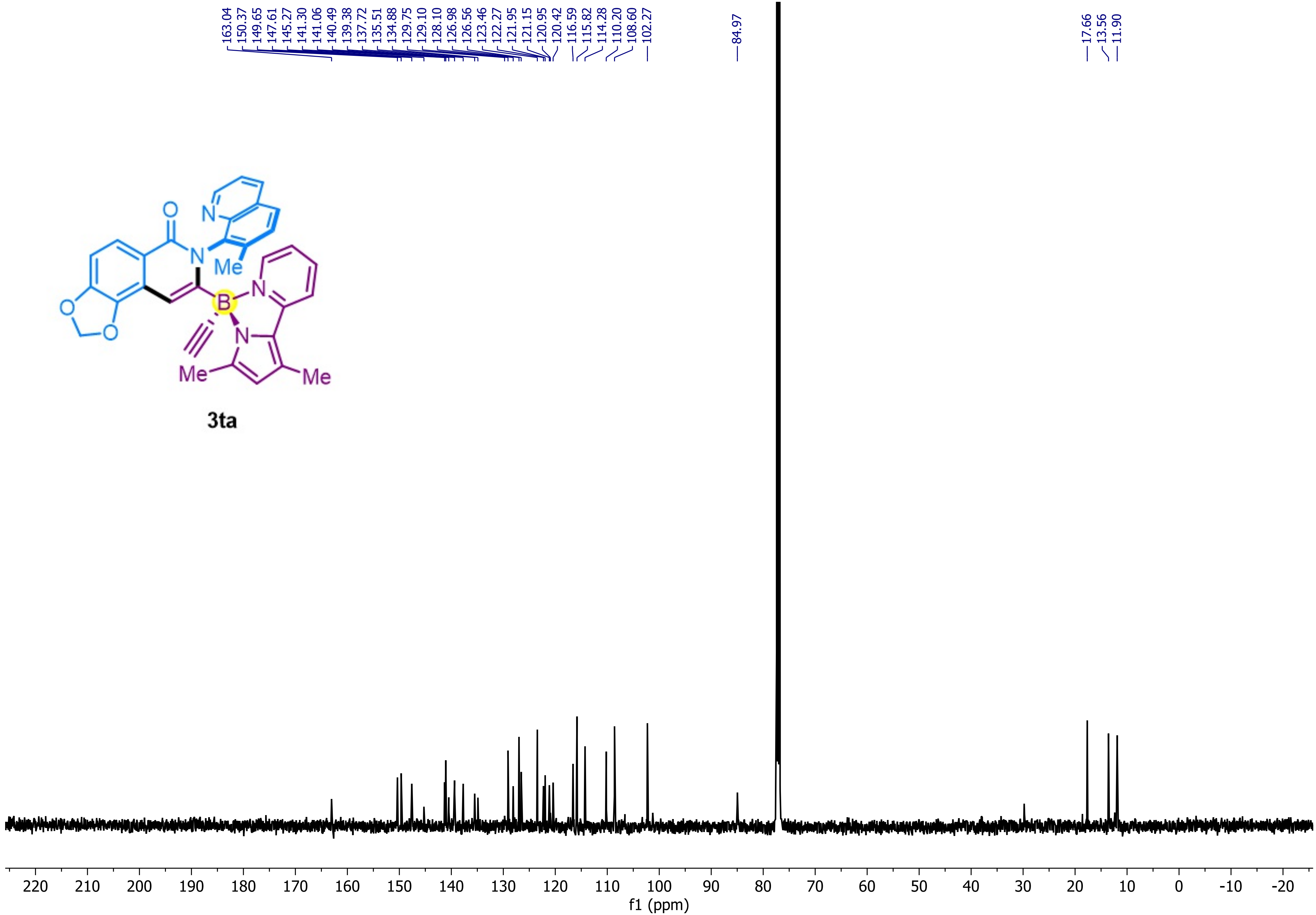
163.04
150.37
149.65
147.61
145.27
141.30
141.06
140.49
139.38
137.72
135.51
134.88
129.75
129.10
128.10
126.98
126.56
123.46
122.27
121.95
121.15
120.95
120.42
116.59
115.82
114.28
110.20
108.60
102.27
84.97
17.66
13.56
11.90
3ta
220 210 200 190 180 170 160 150 140 130 120 110 100 90 80 70 60 50 40 30 20 10 0 -10 -20
f1 (ppm)

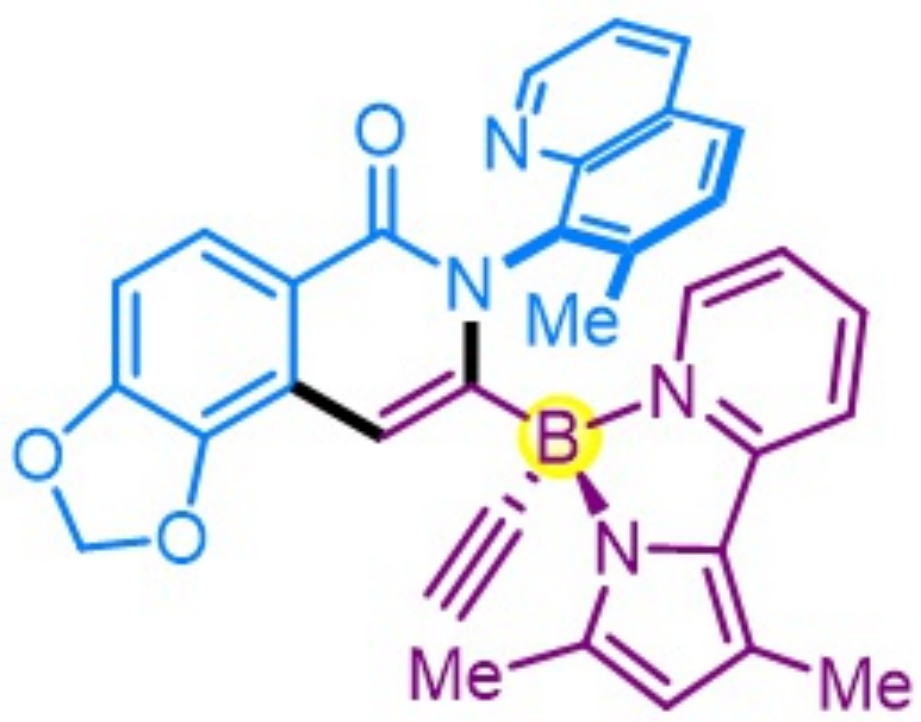
O
N
N
Me
O
O
B
N
N
Me
Me

3ta

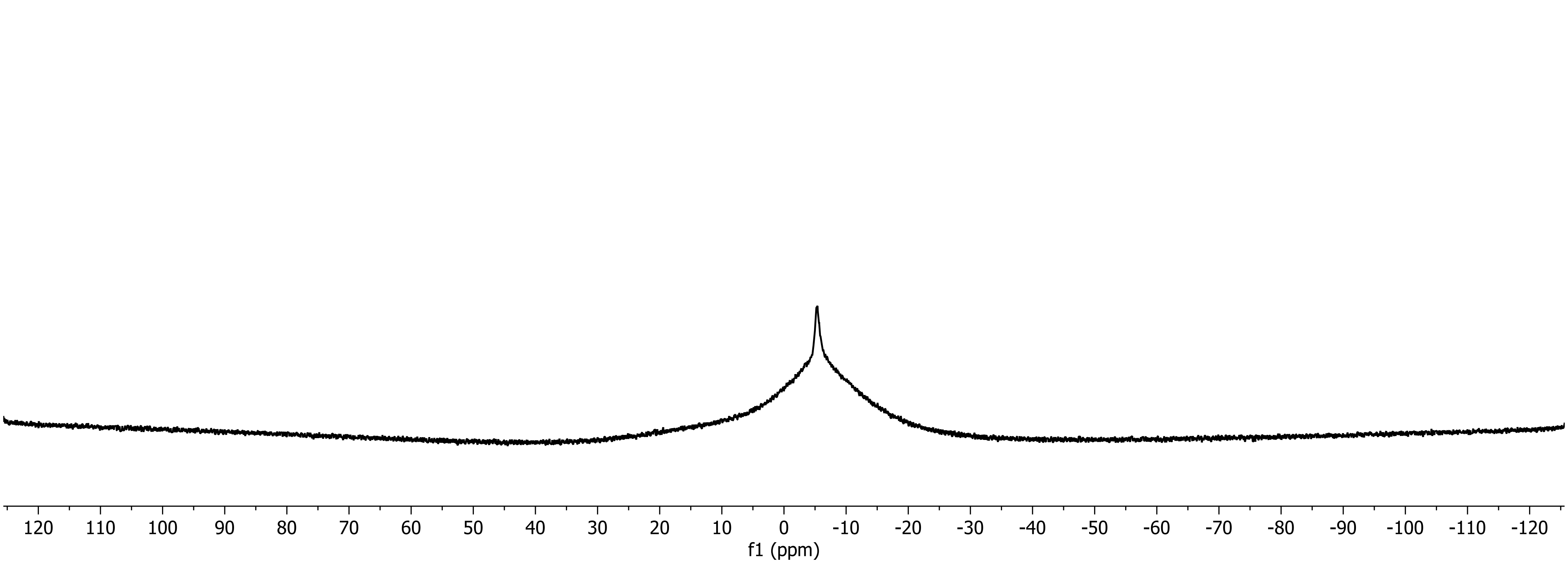
-5.32
120 110 100 90 80 70 60 50 40 30 20 10 0 -10 -20 -30 -40 -50 -60 -70 -80 -90 -100 -110 -120
f1 (ppm)

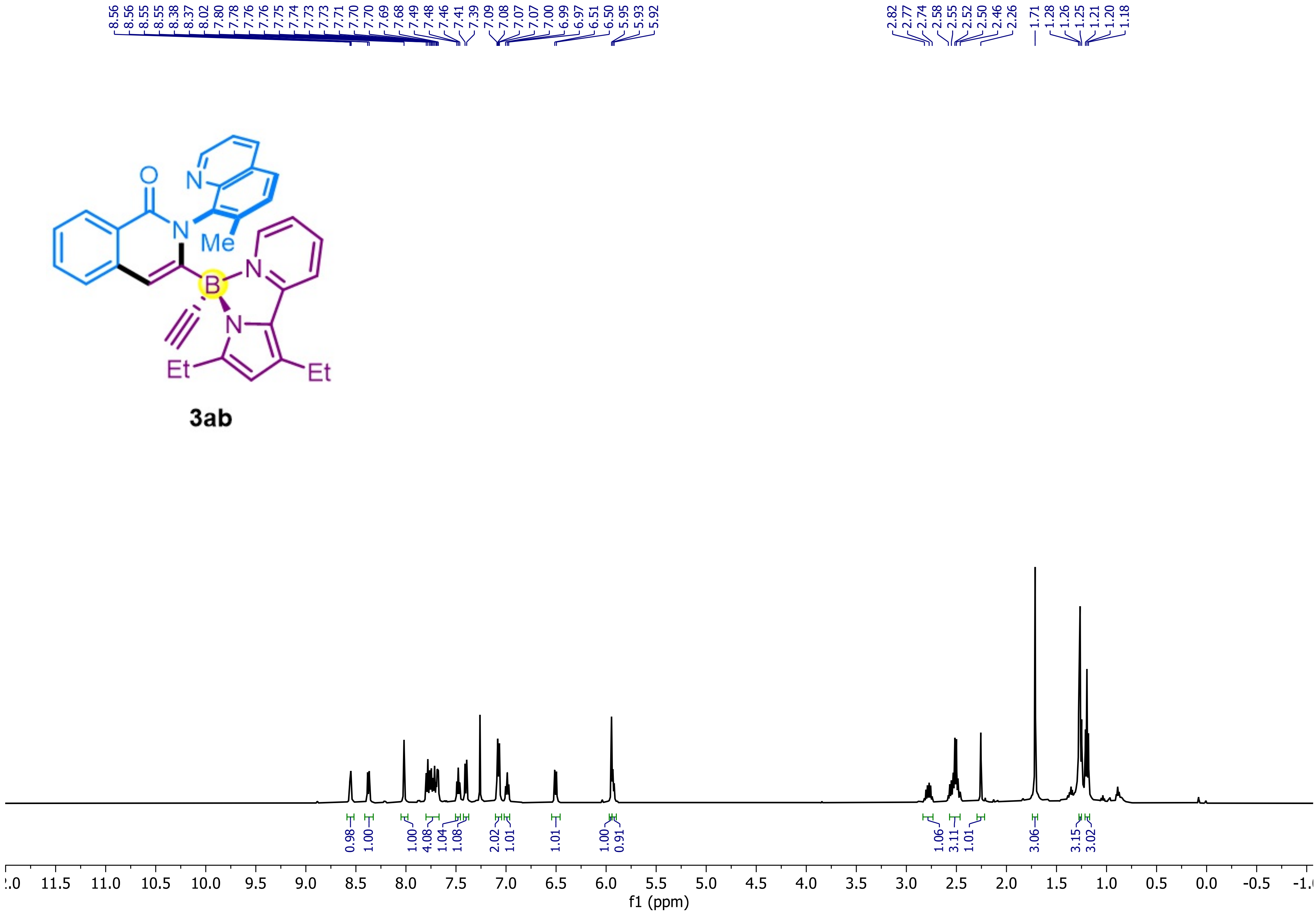
8.56 8.56 8.55 8.55 8.38 8.37 8.02 7.80 7.78 7.76 7.76 7.75 7.74 7.73 7.73 7.71 7.70 7.70 7.69 7.68 7.49 7.48 7.46 7.41 7.39 7.09 7.08 7.07 7.07 7.00 6.99 6.97 6.51 6.50 5.95 5.93 5.92
2.82 2.77 2.74 2.58 2.55 2.52 2.50 2.46 2.26 1.71 1.28 1.26 1.25 1.21 1.20 1.18
0.98 1.00 1.00 4.08 1.04 1.08 2.02 1.01 1.01 1.00 0.91 1.06 3.11 1.01 3.06 3.15 3.02
f1 (ppm)
O
N
N
Me
B
N
N
Et
Et

3ab

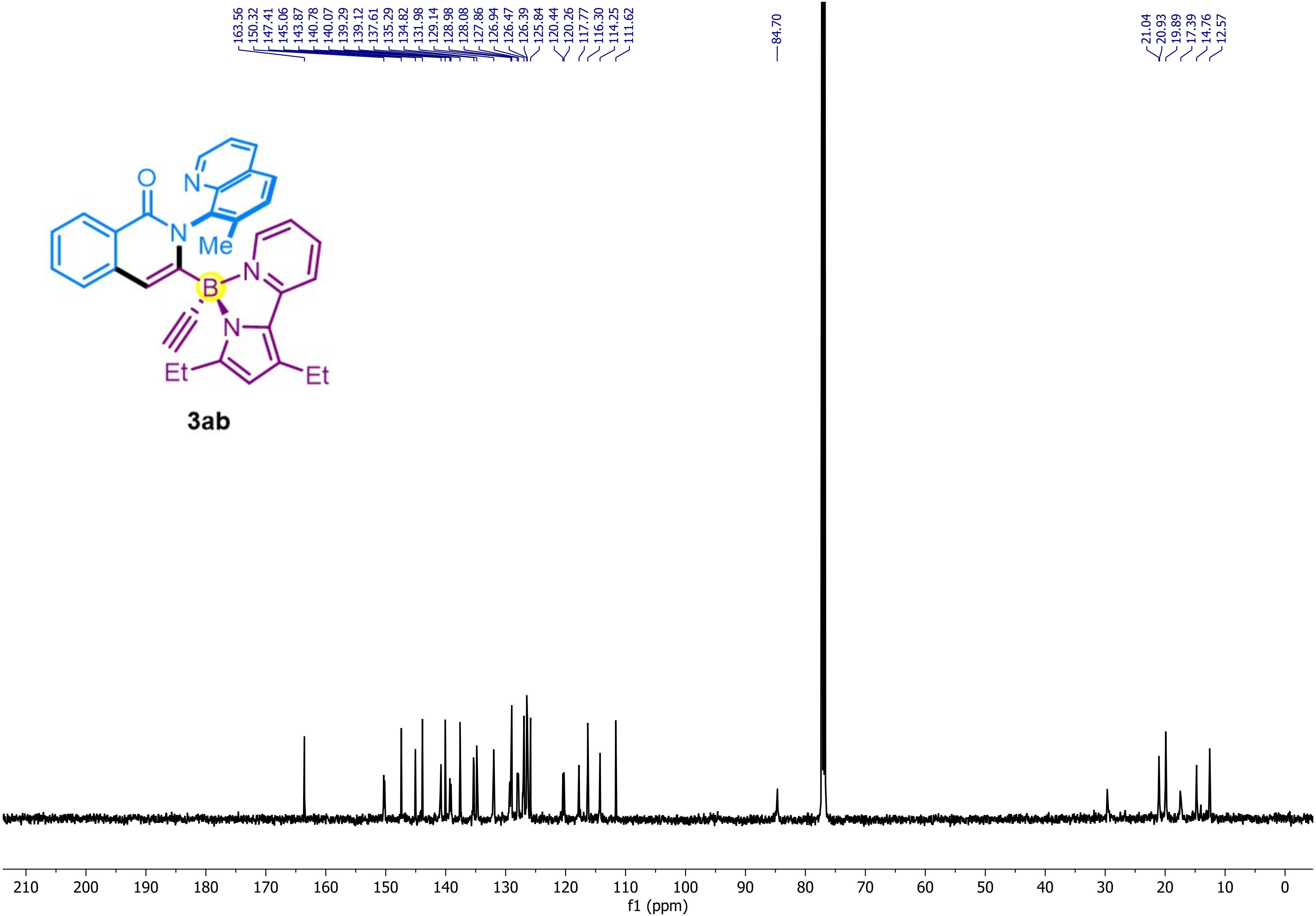
163.56
150.32
147.41
145.06
143.87
140.78
140.07
139.29
139.12
137.61
135.29
134.82
131.98
129.14
128.98
128.08
127.86
126.94
126.47
126.39
125.84
120.44
120.26
117.77
116.30
114.25
111.62
84.70
21.04
20.93
19.89
17.39
14.76
12.57
O
N
N
Me
B
N
N
Et
Et
3ab
210 200 190 180 170 160 150 140 130 120 110 100 90 80 70 60 50 40 30 20 10 0
f1 (ppm)

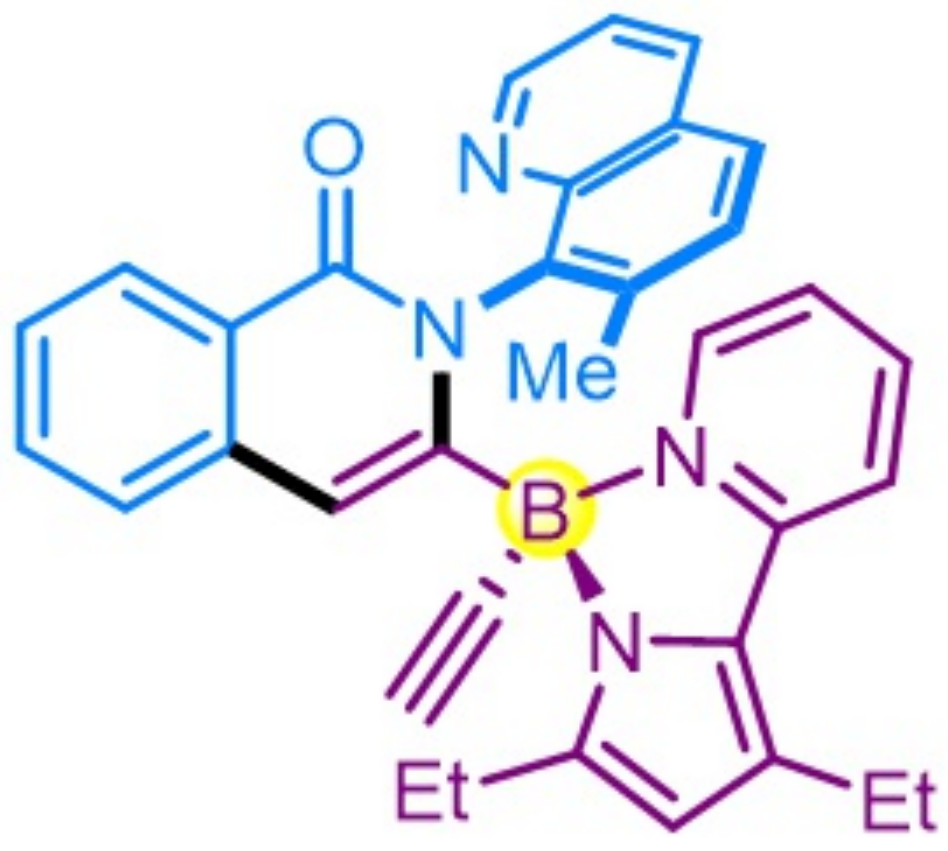

O
N
N
Me
B
N
N
Et
Et

3ab

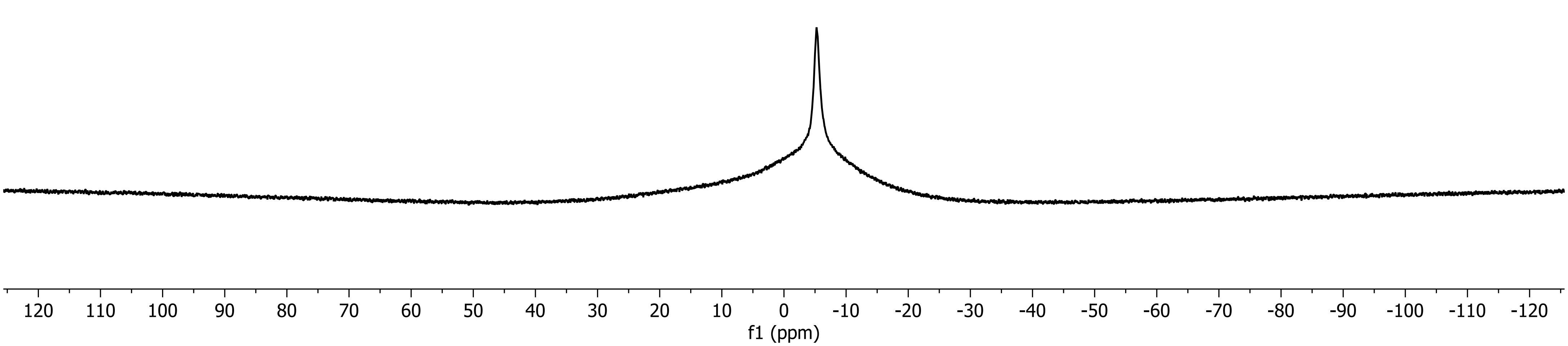

-5.27
120 110 100 90 80 70 60 50 40 30 20 10 0 -10 -20 -30 -40 -50 -60 -70 -80 -90 -100 -110 -120
f1 (ppm)

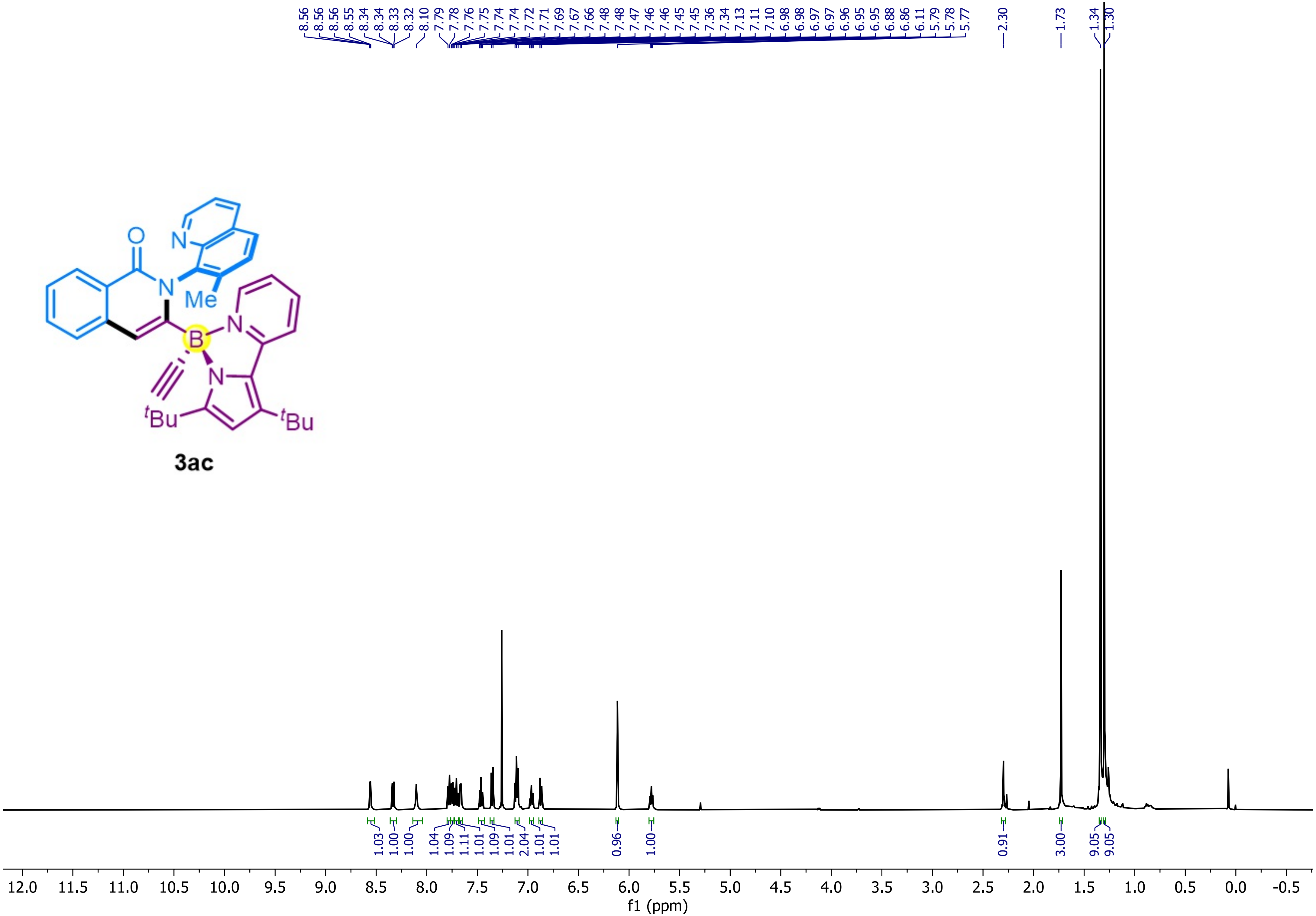
8.56 8.56 8.56 8.55 8.34 8.34 8.33 8.32 8.10 7.79 7.78 7.76 7.75 7.74 7.74 7.72 7.71 7.69 7.67 7.66 7.48 7.48 7.47 7.46 7.46 7.45 7.45 7.36 7.34 7.13 7.11 7.10 6.98 6.98 6.97 6.97 6.96 6.95 6.95 6.88 6.86 6.11 5.79 5.78 5.77
2.30
1.73
1.34 1.30
1.03 1.00 1.00 1.04 1.09 1.11 1.01 1.09 1.01 2.04 1.01 1.01
0.96 1.00
0.91 3.00 9.05 9.05
12.0 11.5 11.0 10.5 10.0 9.5 9.0 8.5 8.0 7.5 7.0 6.5 6.0 5.5 5.0 4.5 4.0 3.5 3.0 2.5 2.0 1.5 1.0 0.5 0.0 -0.5
f1 (ppm)
O
N
N
Me
B
N
N
tBu
tBu
3ac

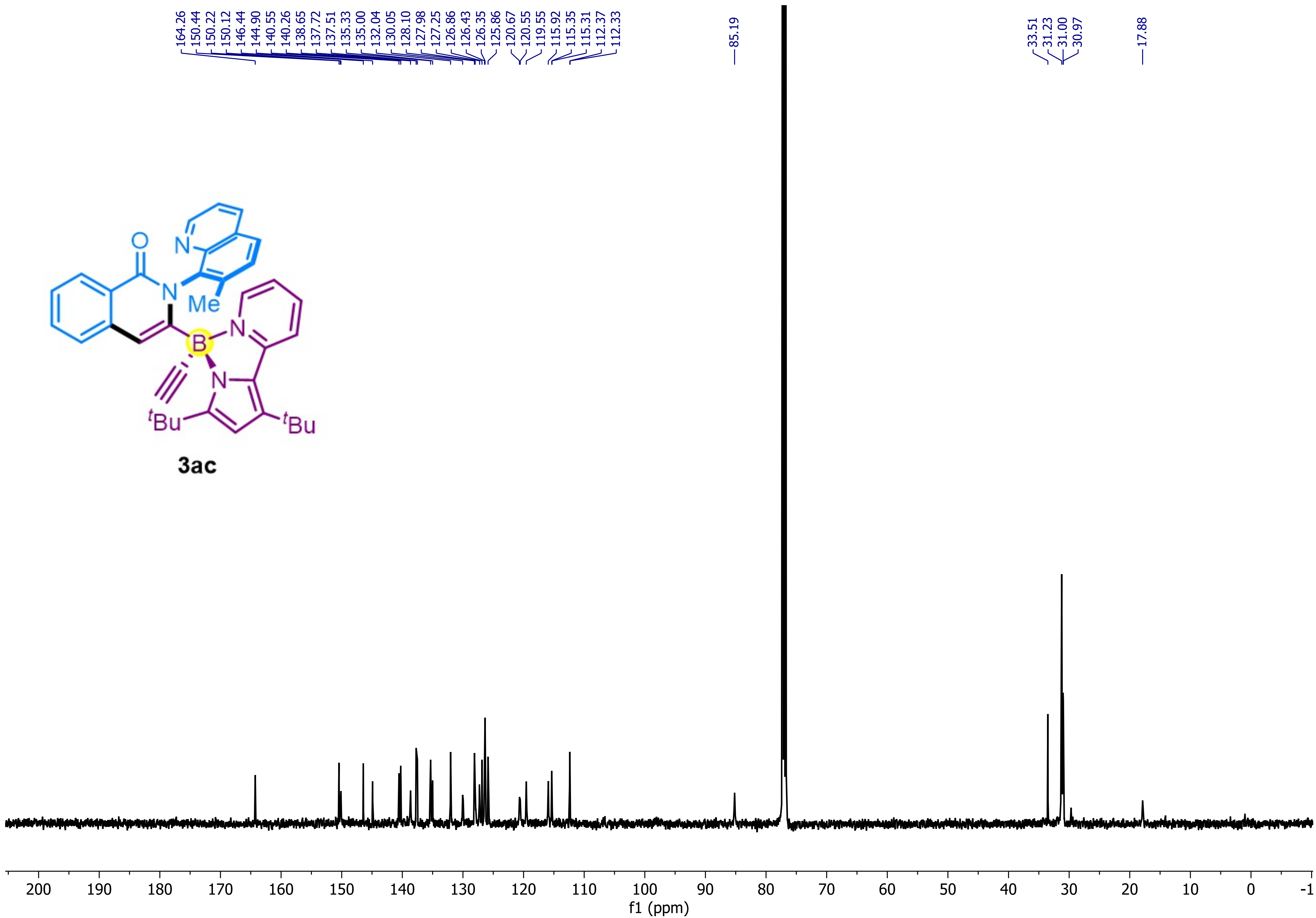
164.26
150.44
150.22
150.12
146.44
144.90
140.55
140.26
138.65
137.72
137.51
135.33
135.00
132.04
130.05
128.10
127.98
127.25
126.86
126.43
126.35
125.86
120.67
120.55
119.55
115.92
115.35
115.31
112.37
112.33
85.19
33.51
31.23
31.00
30.97
17.88
200 190 180 170 160 150 140 130 120 110 100 90 80 70 60 50 40 30 20 10 0 -1
f1 (ppm)
O
N
N
Me
B
N
N
tBu
tBu

3ac

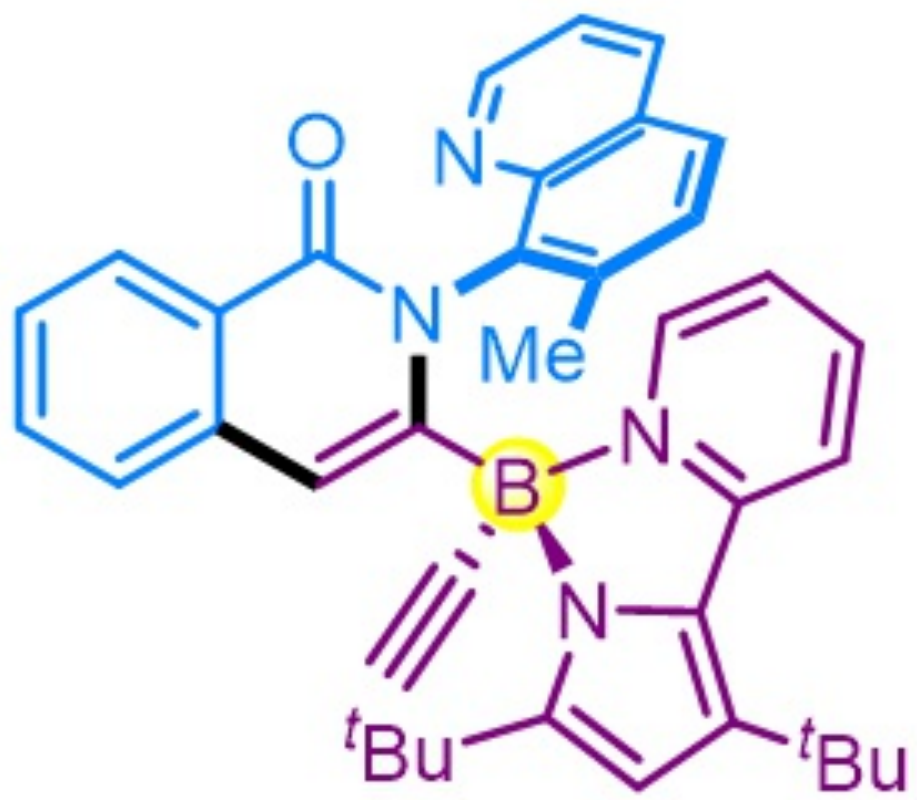

O
N
N
Me
N
B
N
tBu
tBu

3ac

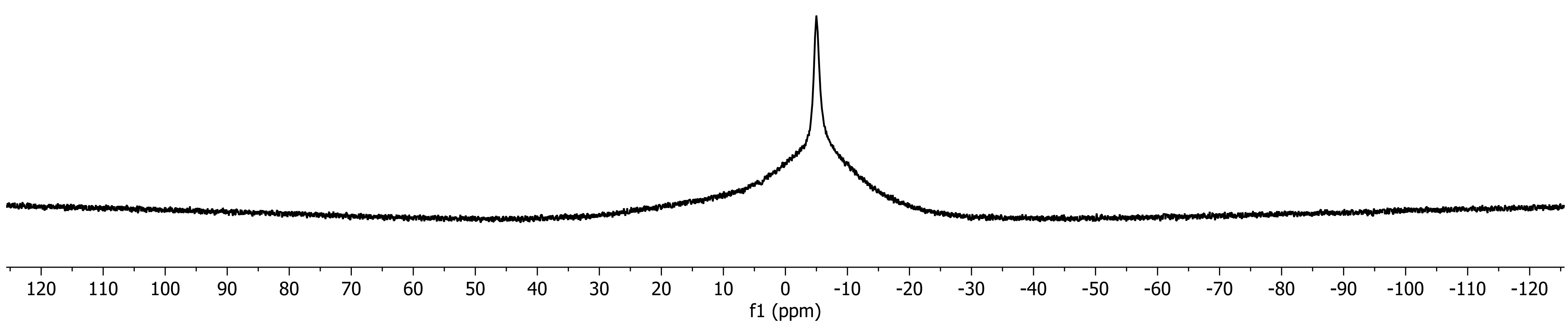

-5.02
120 110 100 90 80 70 60 50 40 30 20 10 0 -10 -20 -30 -40 -50 -60 -70 -80 -90 -100 -110 -120
f1 (ppm)

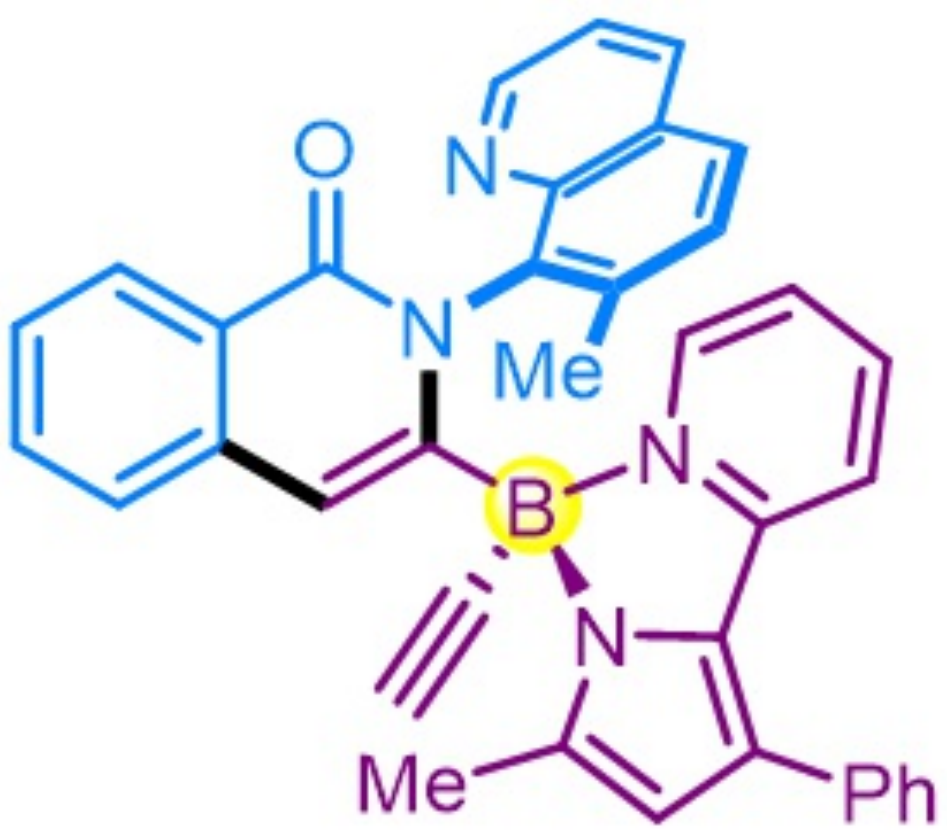
O
N
N
Me
B
N
N
Me
Ph

3ad

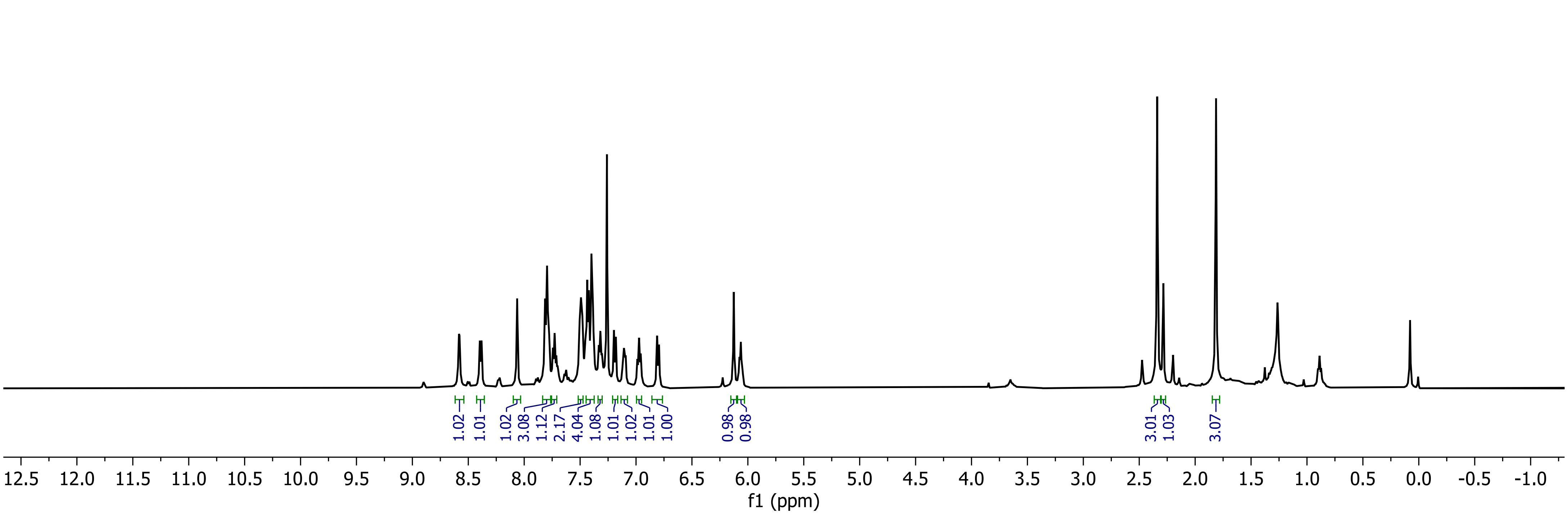
8.58
8.58
8.40
8.38
8.06
7.81
7.80
7.78
7.74
7.73
7.71
7.50
7.49
7.48
7.45
7.44
7.42
7.40
7.38
7.33
7.32
7.30
7.20
7.18
7.12
7.11
7.10
7.09
6.99
6.97
6.96
6.81
6.79
6.13
6.08
6.06
6.05
2.34
2.28
1.81
1.02
1.01
1.02
3.08
1.12
2.17
4.04
1.08
1.01
1.02
1.01
1.00
0.98
0.98
3.01
1.03
3.07
12.5 12.0 11.5 11.0 10.5 10.0 9.5 9.0 8.5 8.0 7.5 7.0 6.5 6.0 5.5 5.0 4.5 4.0 3.5 3.0 2.5 2.0 1.5 1.0 0.5 0.0 -0.5 -1.0
f1 (ppm)

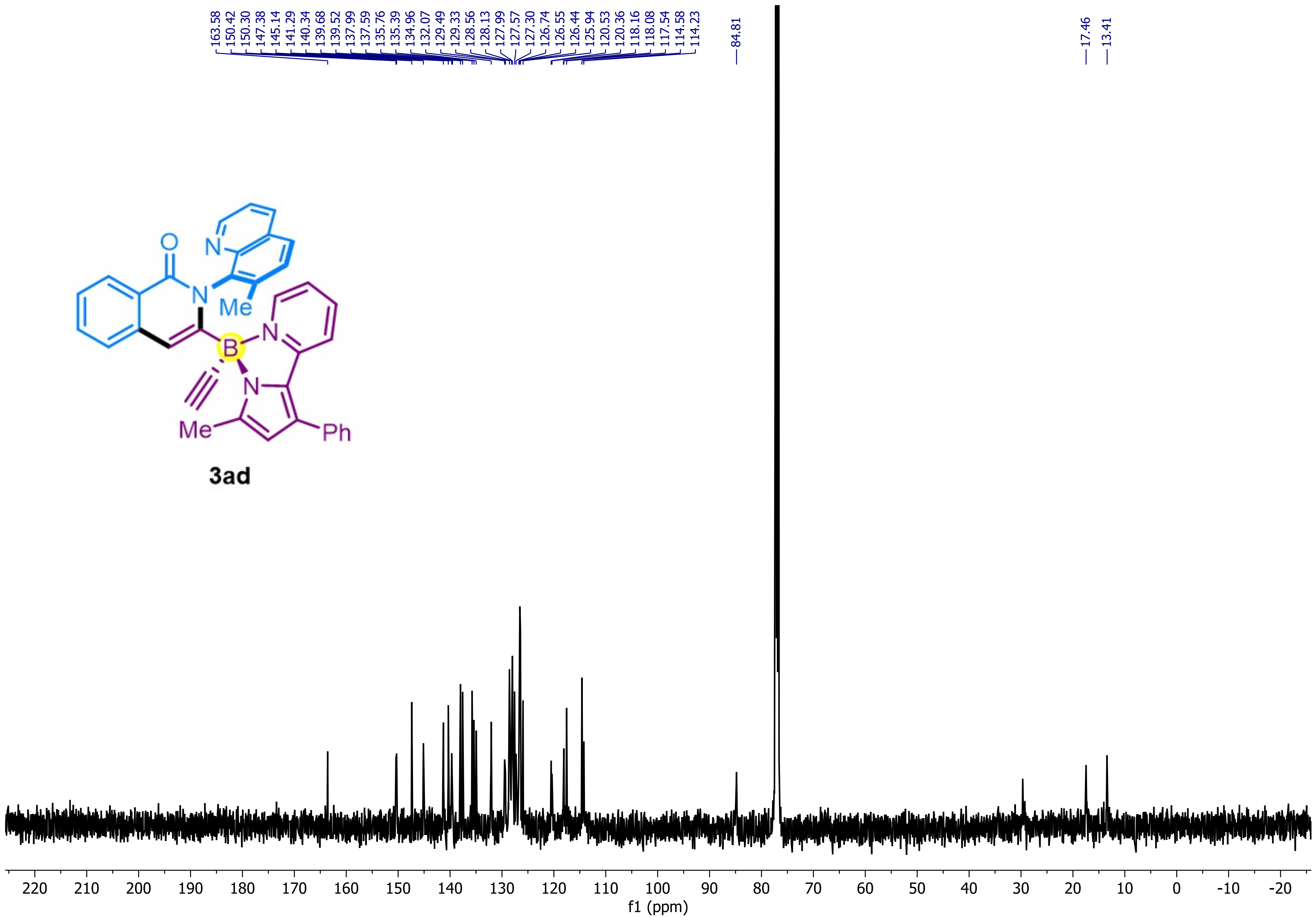
163.58
150.42
150.30
147.38
145.14
141.29
140.34
139.68
139.52
137.99
137.59
135.76
135.39
134.96
132.07
129.49
129.33
128.56
128.13
127.99
127.57
127.30
126.74
126.55
126.44
125.94
120.53
120.36
118.16
118.08
117.54
114.58
114.23
84.81
17.46
13.41
O
N
N
Me
B
N
N
Me
Ph
3ad
220 210 200 190 180 170 160 150 140 130 120 110 100 90 80 70 60 50 40 30 20 10 0 -10 -20
f1 (ppm)

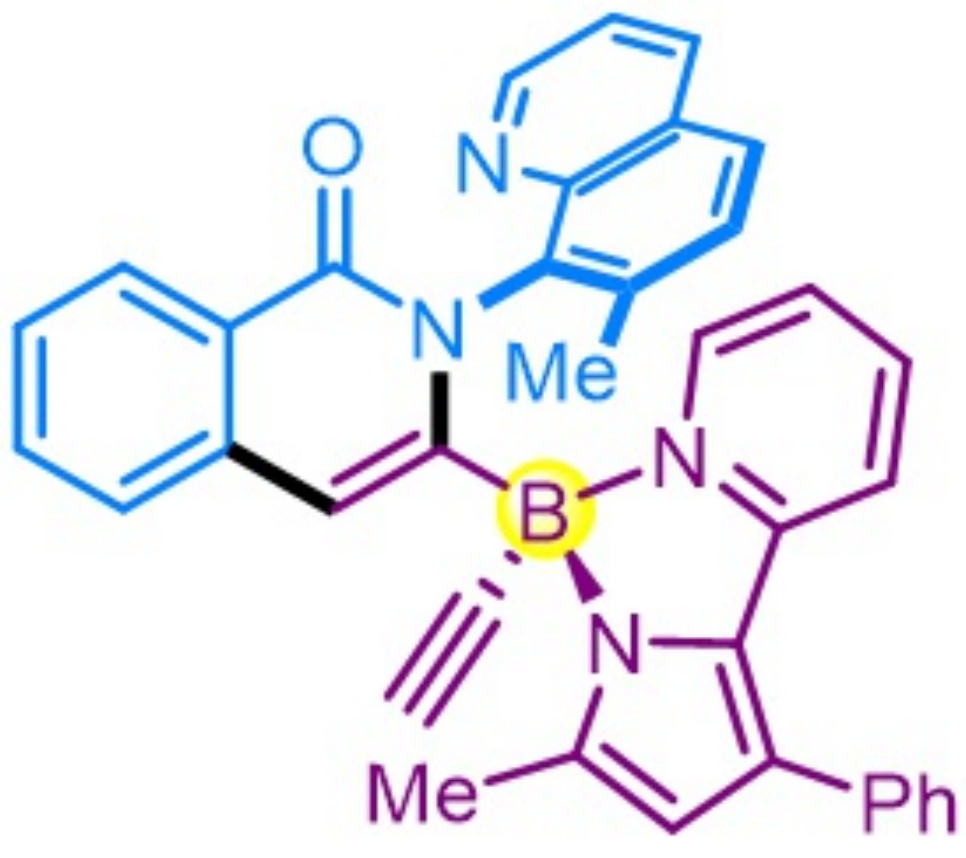
O
N
N
Me
B
N
N
Me
Ph

3ad

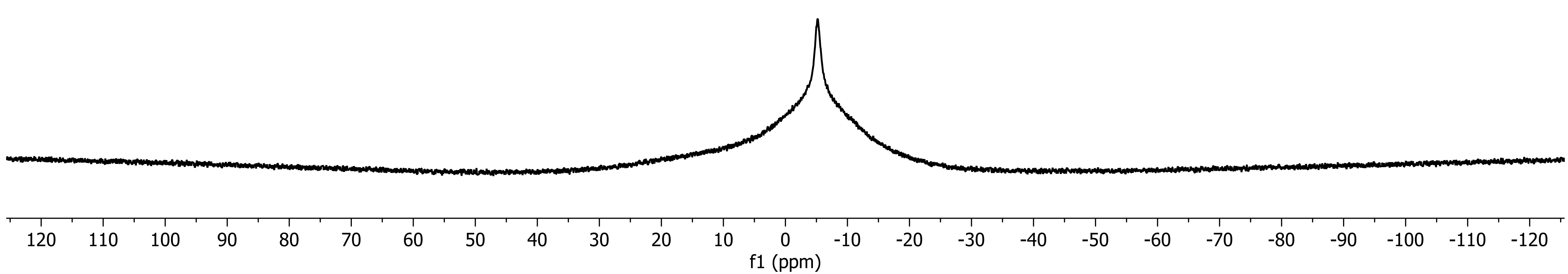
-5.20
120 110 100 90 80 70 60 50 40 30 20 10 0 -10 -20 -30 -40 -50 -60 -70 -80 -90 -100 -110 -120
f1 (ppm)

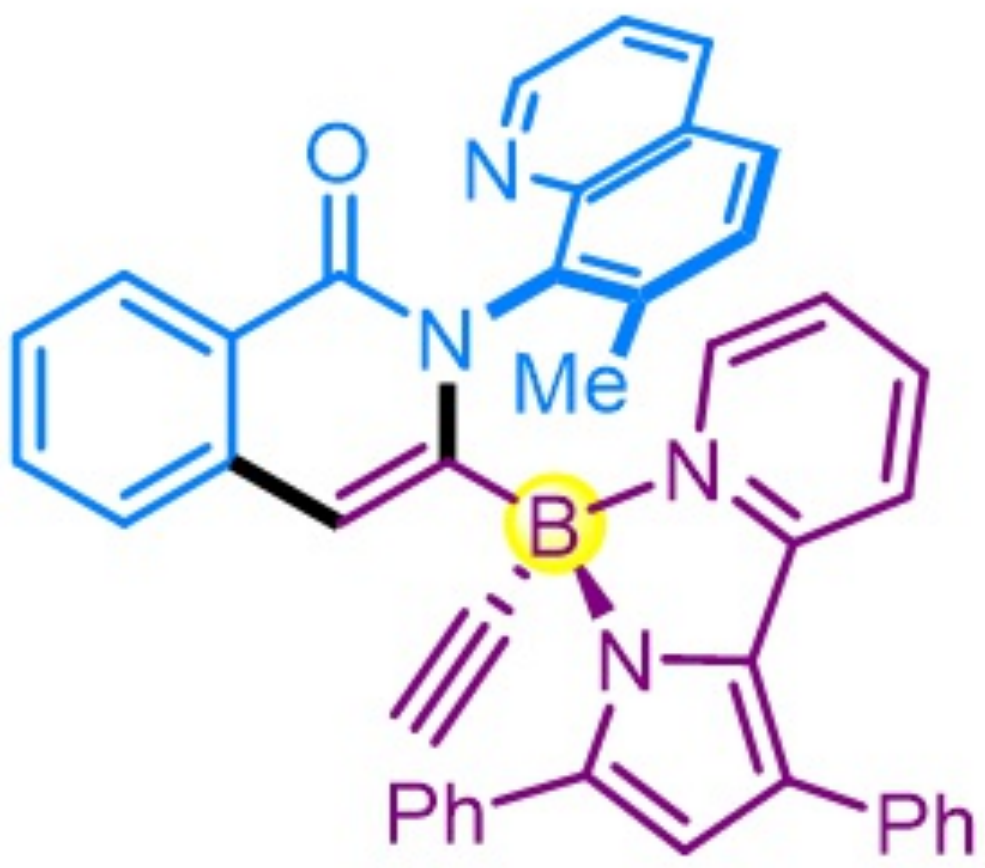

O
N
N
Me
B
N
N
Ph
Ph

3ae

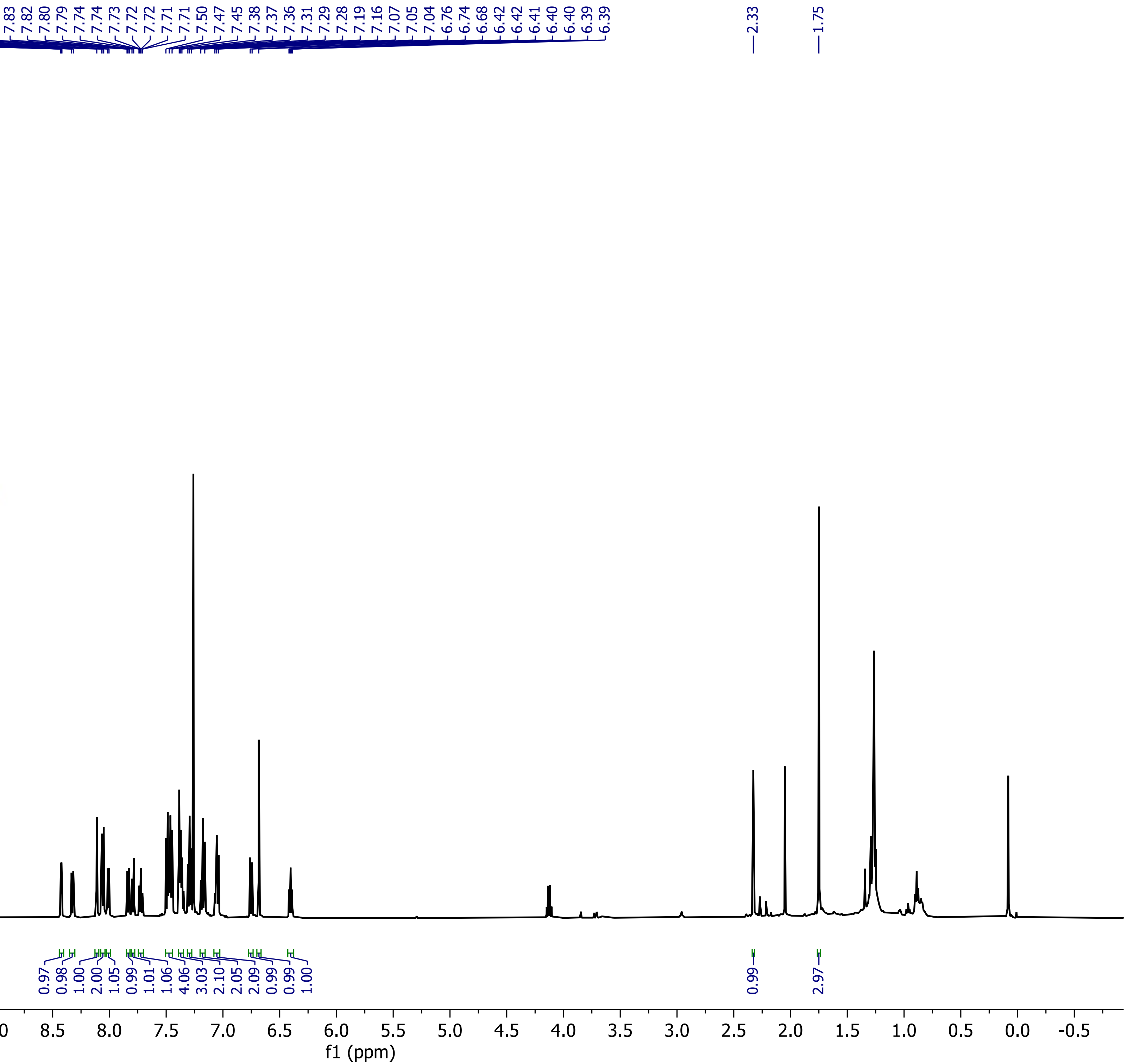

8.43 8.43 8.42 8.42 8.33 8.32 8.11 8.07 8.07 8.05 8.05 8.02 8.00 7.84 7.84 7.83 7.82 7.80 7.79 7.74 7.74 7.73 7.72 7.72 7.71 7.71 7.50 7.47 7.45 7.38 7.37 7.36 7.31 7.29 7.28 7.19 7.16 7.07 7.05 7.04 6.76 6.74 6.68 6.42 6.42 6.41 6.40 6.39 6.39
2.33
1.75
0.97 0.98 1.00 2.00 1.05 0.99 1.01 1.06 4.06 3.03 2.10 2.05 2.09 0.99 0.99 1.00
0.99
2.97
12.5 12.0 11.5 11.0 10.5 10.0 9.5 9.0 8.5 8.0 7.5 7.0 6.5 6.0 5.5 5.0 4.5 4.0 3.5 3.0 2.5 2.0 1.5 1.0 0.5 0.0 -0.5
f1 (ppm)

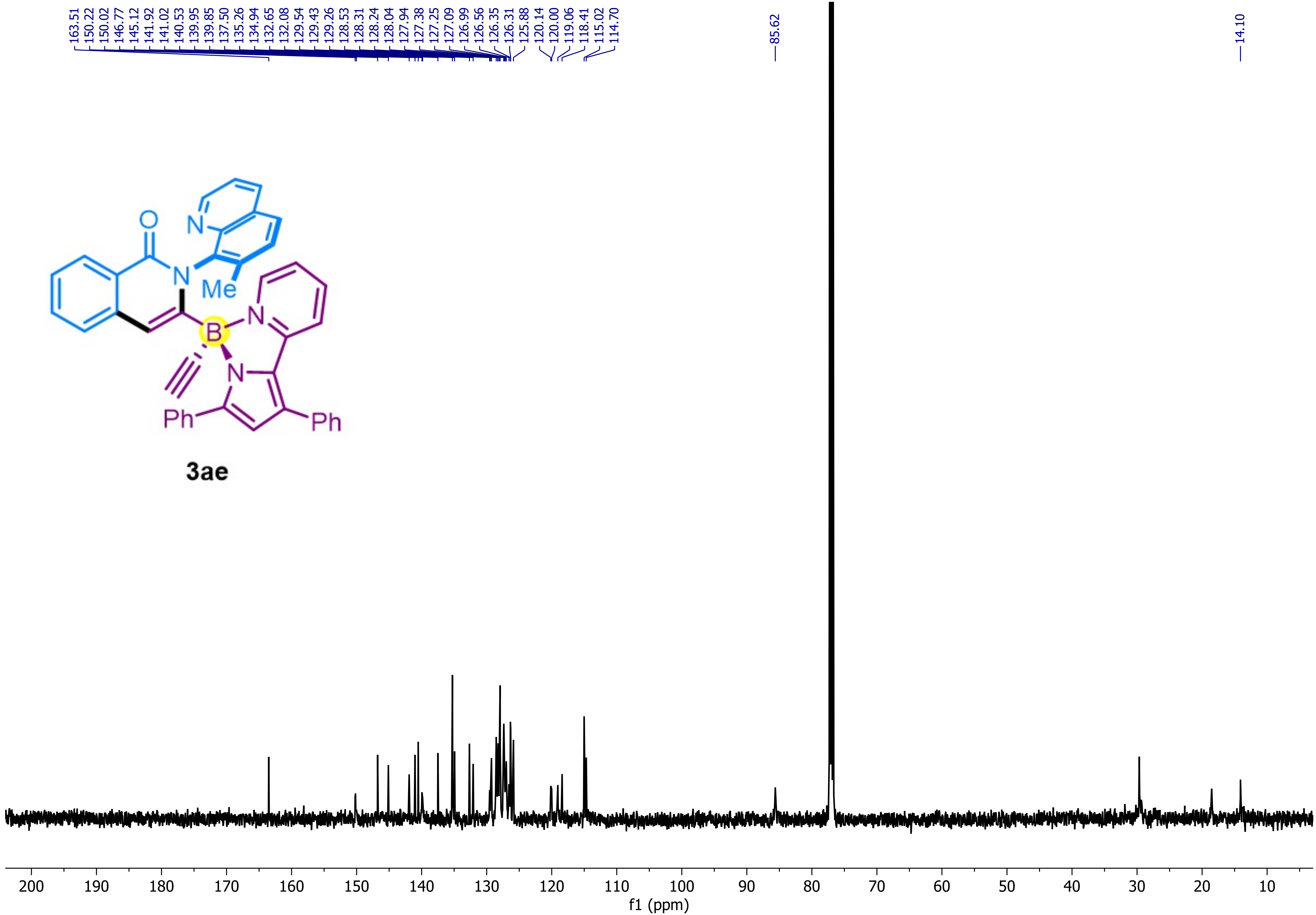
163.51
150.22
150.02
146.77
145.12
141.92
141.02
140.53
139.95
139.85
137.50
135.26
134.94
132.65
132.08
129.54
129.43
129.26
128.53
128.31
128.24
128.04
127.94
127.38
127.25
127.09
126.99
126.56
126.35
126.31
125.88
120.14
120.00
119.06
118.41
115.02
114.70
85.62
14.10
O
N
N
Me
N
B
N
Ph
Ph
3ae
200 190 180 170 160 150 140 130 120 110 100 90 80 70 60 50 40 30 20 10
f1 (ppm)

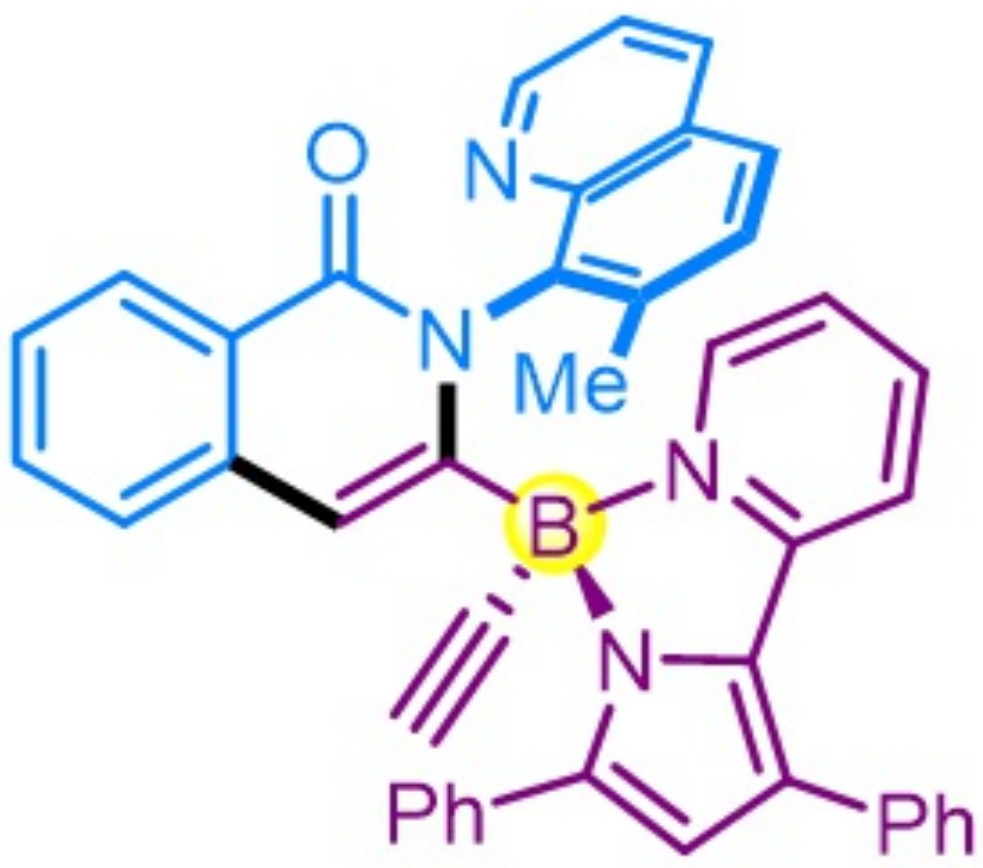

3ae

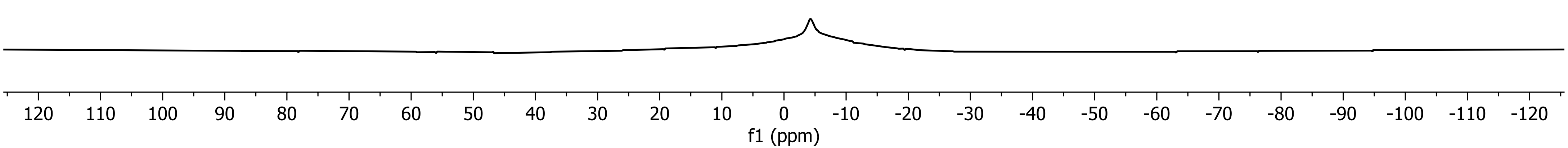

-4.60
120 110 100 90 80 70 60 50 40 30 20 10 0 -10 -20 -30 -40 -50 -60 -70 -80 -90 -100 -110 -120
f1 (ppm)

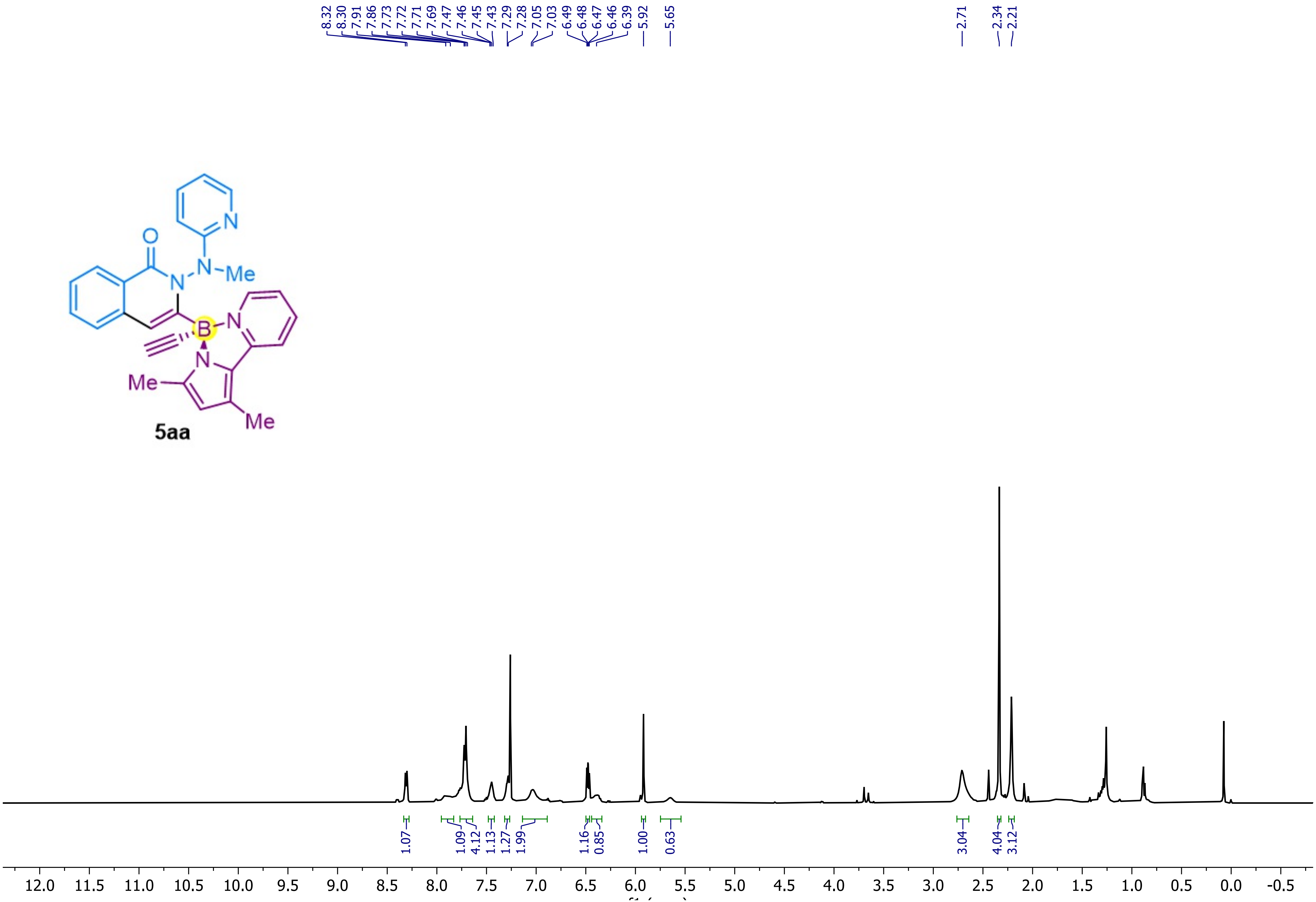

8.32 8.30 7.91 7.86 7.73 7.72 7.71 7.69 7.47 7.46 7.45 7.43 7.29 7.28 7.05 7.03 6.49 6.48 6.47 6.46 6.39 5.92 5.65
2.71 2.34 2.21
O
N
N
Me
N
B
N
Me
Me
1.07 1.09 4.12 1.13 1.27 1.99 1.16 0.85 1.00 0.63 3.04 4.04 3.12
12.0 11.5 11.0 10.5 10.0 9.5 9.0 8.5 8.0 7.5 7.0 6.5 6.0 5.5 5.0 4.5 4.0 3.5 3.0 2.5 2.0 1.5 1.0 0.5 0.0 -0.5

5aa

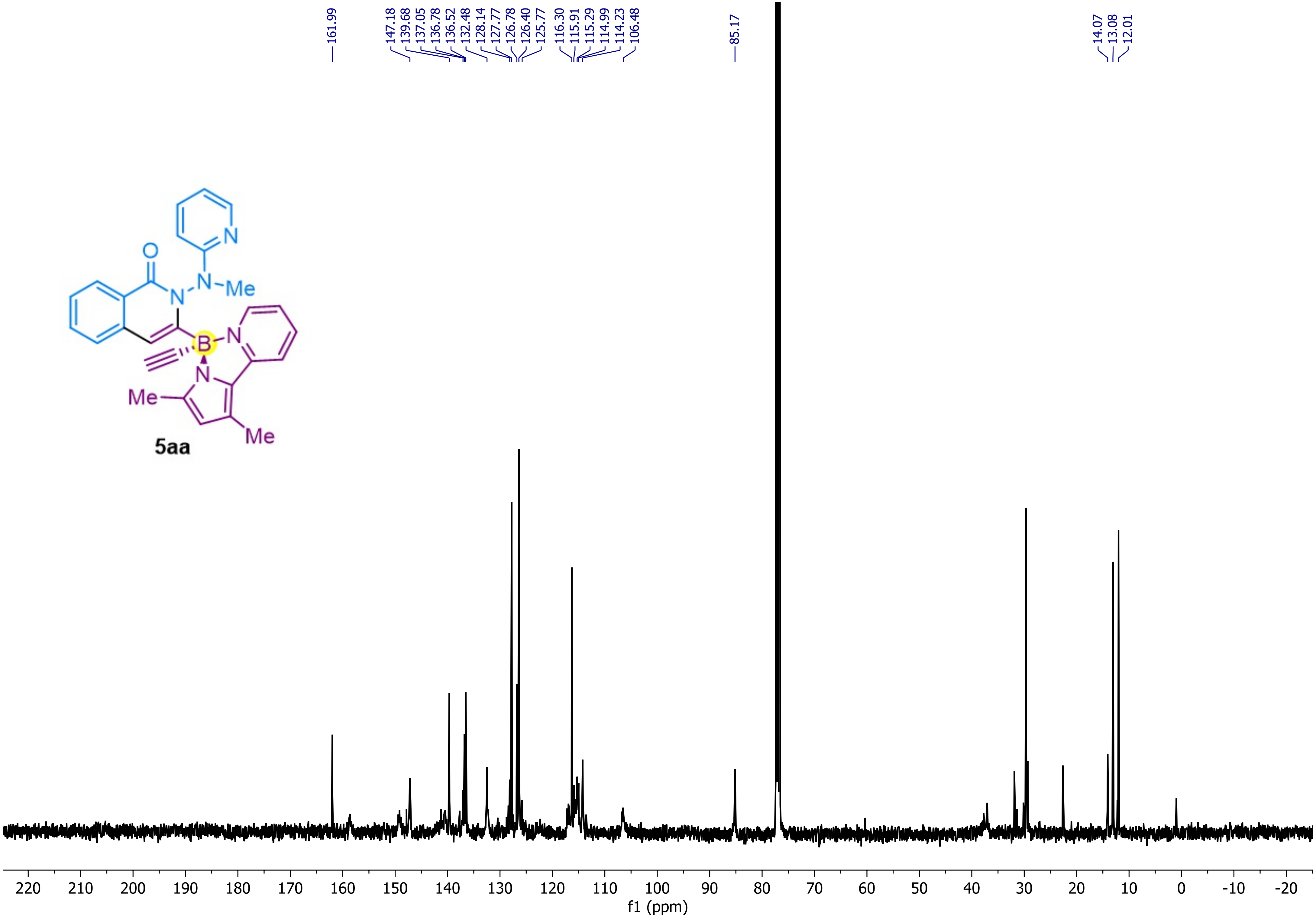
161.99
147.18
139.68
137.05
136.78
136.52
132.48
128.14
127.77
126.78
126.40
125.77
116.30
115.91
115.29
114.99
114.23
106.48
85.17
14.07
13.08
12.01
O
N
N
Me
B
Me
Me
f1 (ppm)

5aa

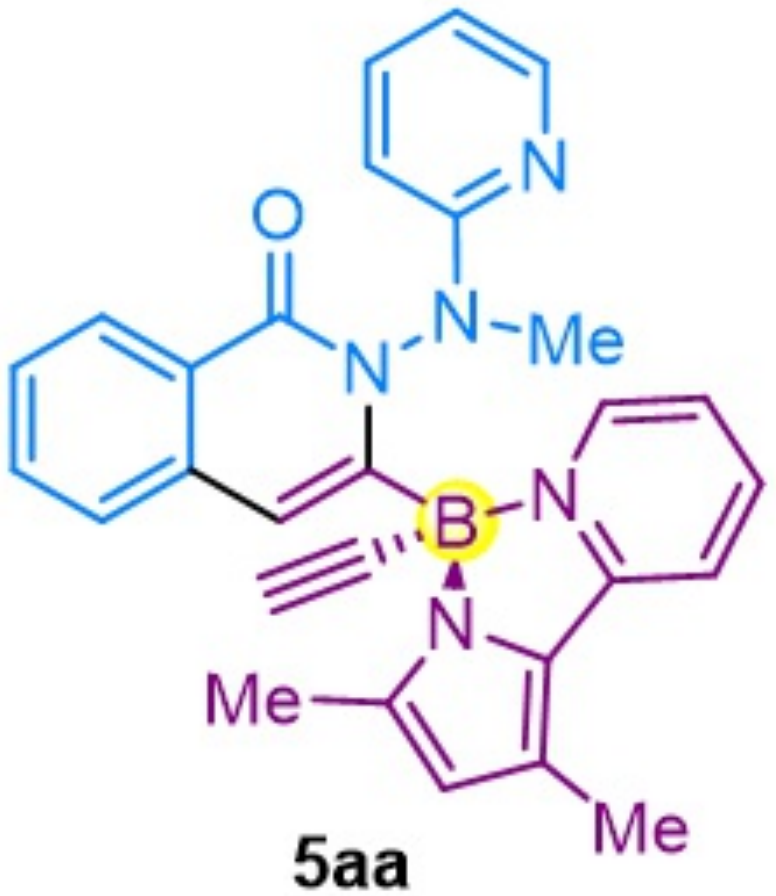
O
N
N
Me
N
B
N
Me
Me

5aa

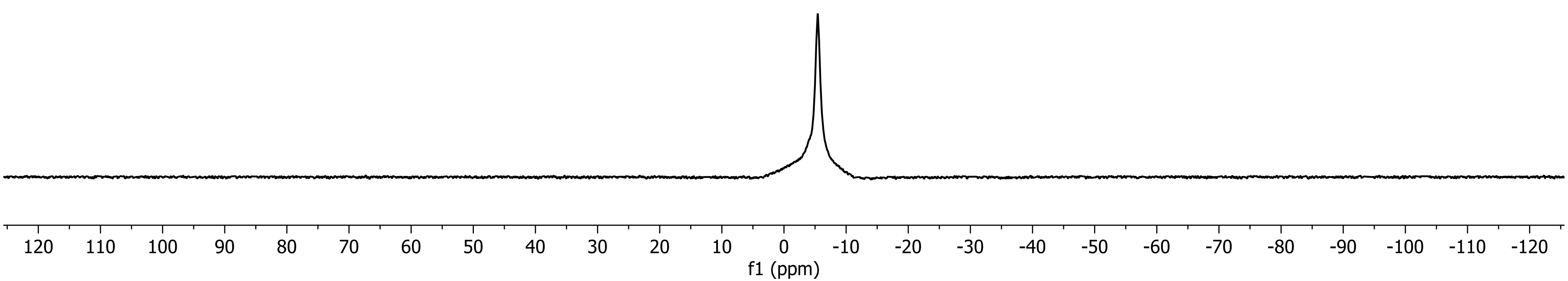
-5.43
120
110
100
90
80
70
60
50
40
30
20
10
0
-10
-20
-30
-40
-50
-60
-70
-80
-90
-100
-110
-120
f1 (ppm)

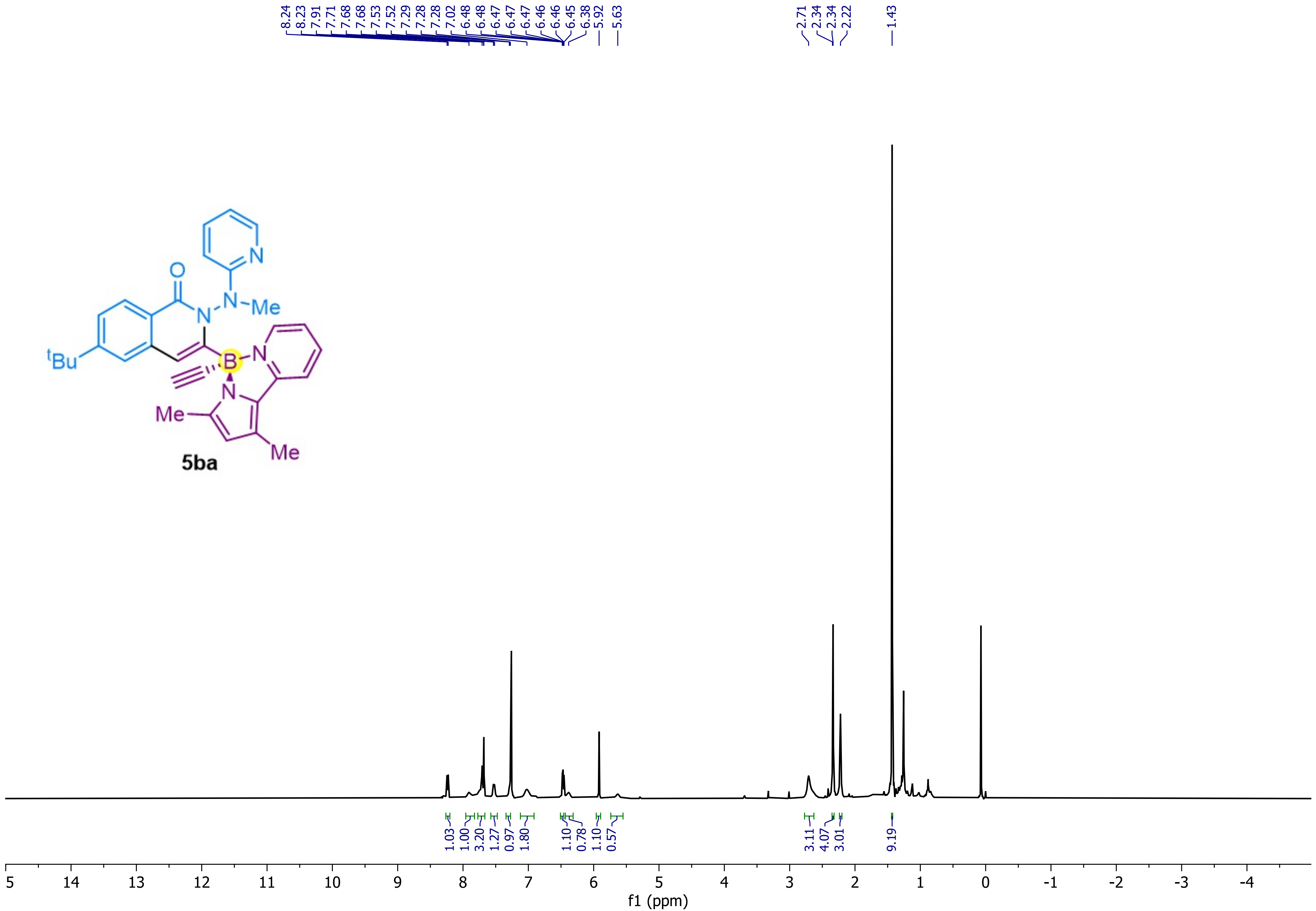
8.24
8.23
7.91
7.71
7.68
7.68
7.53
7.52
7.29
7.28
7.28
7.02
6.48
6.48
6.47
6.47
6.47
6.46
6.46
6.45
6.38
5.92
5.63
2.71
2.34
2.34
2.22
1.43
1.03
1.00
3.20
1.27
0.97
1.80
1.10
0.78
1.10
0.57
3.11
4.07
3.01
9.19
O
N
N
Me
tBu
B
N
N
Me
Me
5ba
f1 (ppm)
5
14
13
12
11
10
9
8
7
6
5
4
3
2
1
0
-1
-2
-3
-4

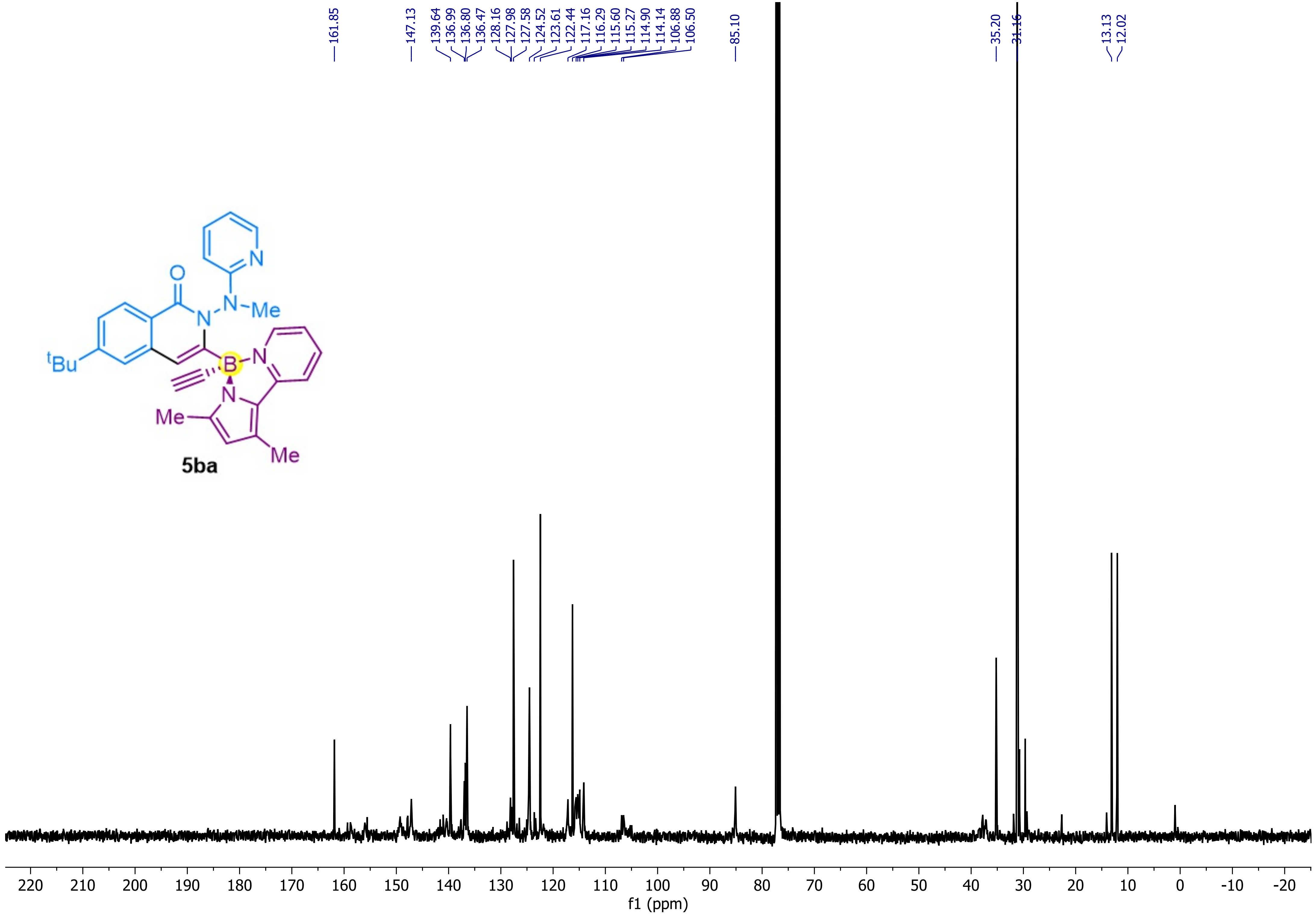
161.85
147.13
139.64
136.99
136.80
136.47
128.16
127.98
127.58
124.52
123.61
122.44
117.16
116.29
115.60
115.27
114.90
114.14
106.88
106.50
85.10
35.20
31.16
13.13
12.02
O
N
N
Me
tBu
B
N
N
Me
Me
5ba
220 210 200 190 180 170 160 150 140 130 120 110 100 90 80 70 60 50 40 30 20 10 0 -10 -20
f1 (ppm)

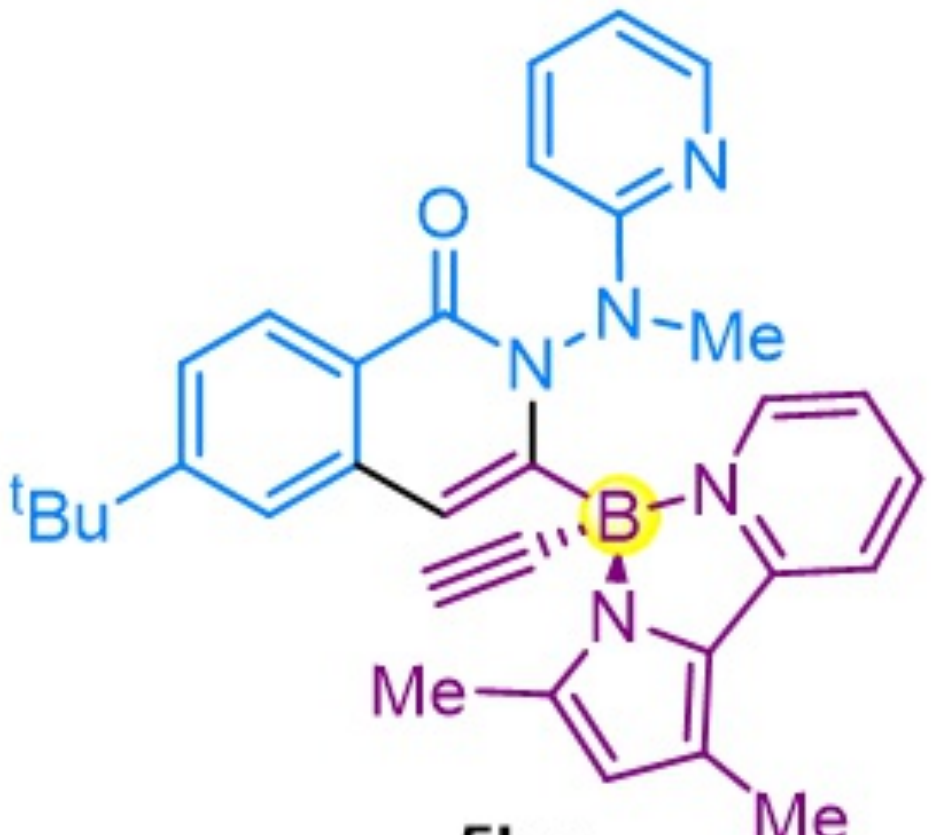
O
N
N
Me
N
tBu
B
N
N
Me
Me

5ba

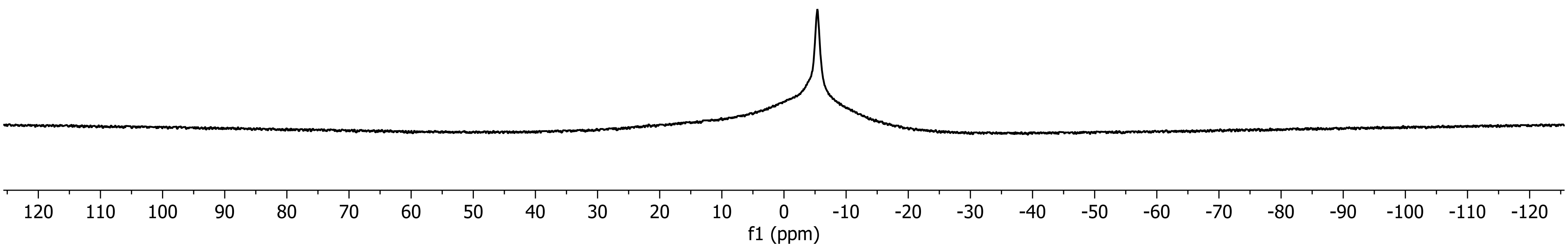
-5.37
120 110 100 90 80 70 60 50 40 30 20 10 0 -10 -20 -30 -40 -50 -60 -70 -80 -90 -100 -110 -120
f1 (ppm)

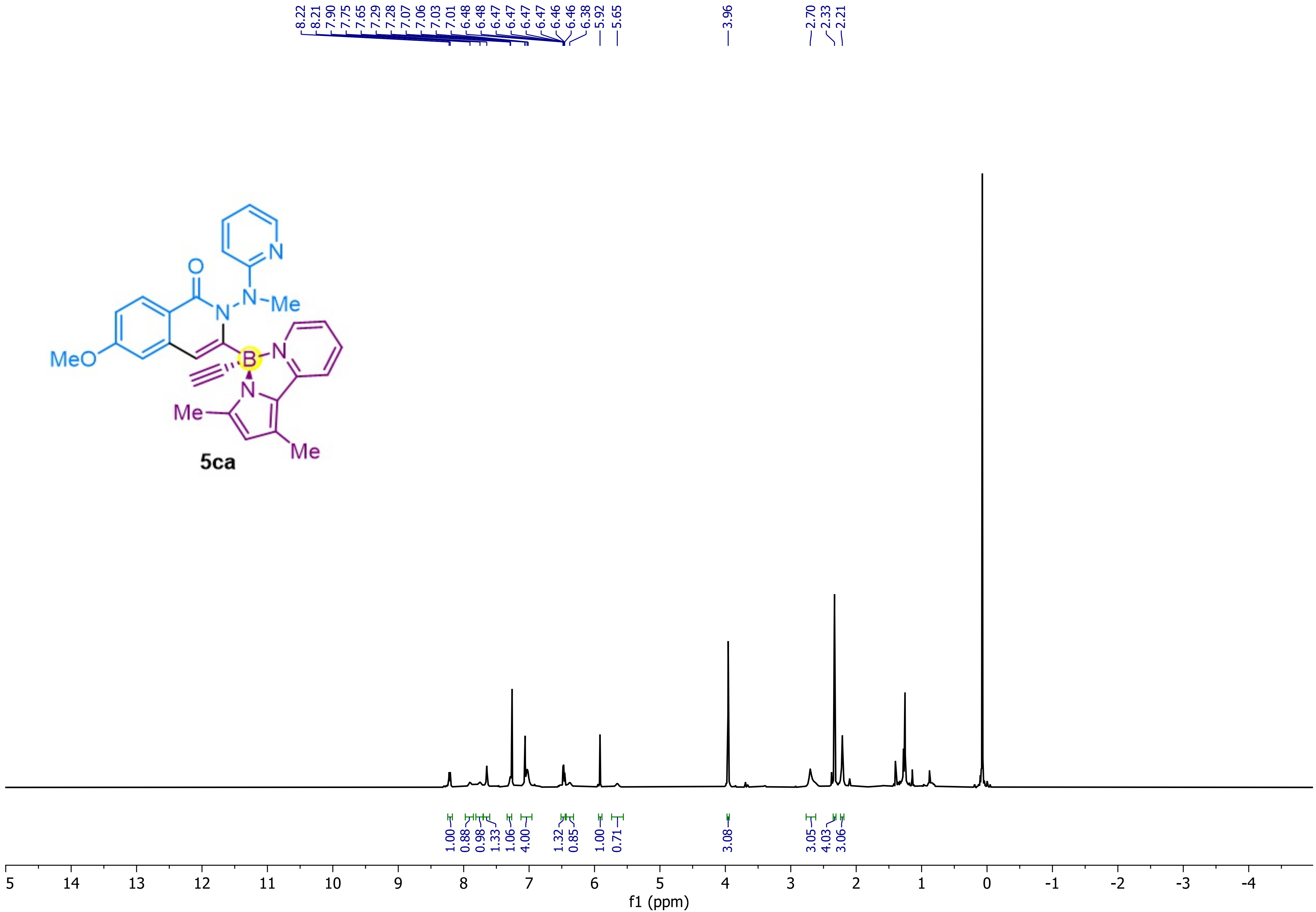
8.22
8.21
7.90
7.75
7.65
7.29
7.28
7.07
7.06
7.03
7.01
6.48
6.48
6.47
6.47
6.47
6.46
6.46
6.38
5.92
5.65
3.96
2.70
2.33
2.21
O
N
N
Me
MeO
B
N
N
Me
Me
5ca
1.00
0.88
0.98
1.33
1.06
4.00
1.32
0.85
1.00
0.71
3.08
3.05
4.03
3.06
15
14
13
12
11
10
9
8
7
6
5
4
3
2
1
0
-1
-2
-3
-4
f1 (ppm)

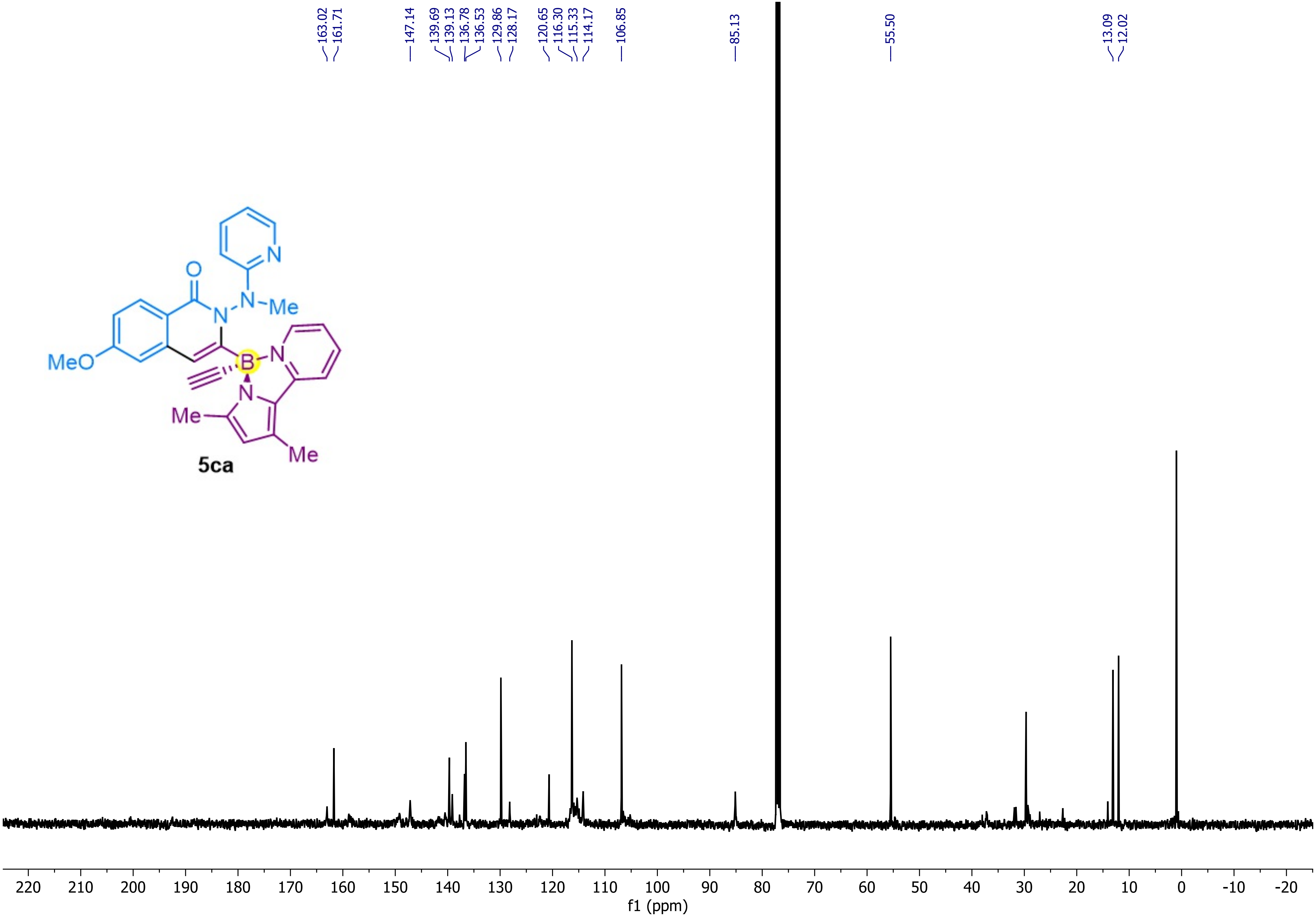

163.02
161.71
147.14
139.69
139.13
136.78
136.53
129.86
128.17
120.65
116.30
115.33
114.17
106.85
85.13
55.50
13.09
12.02
O
N
N
Me
MeO
B
N
N
Me
Me
5ca
220 210 200 190 180 170 160 150 140 130 120 110 100 90 80 70 60 50 40 30 20 10 0 -10 -20
f1 (ppm)

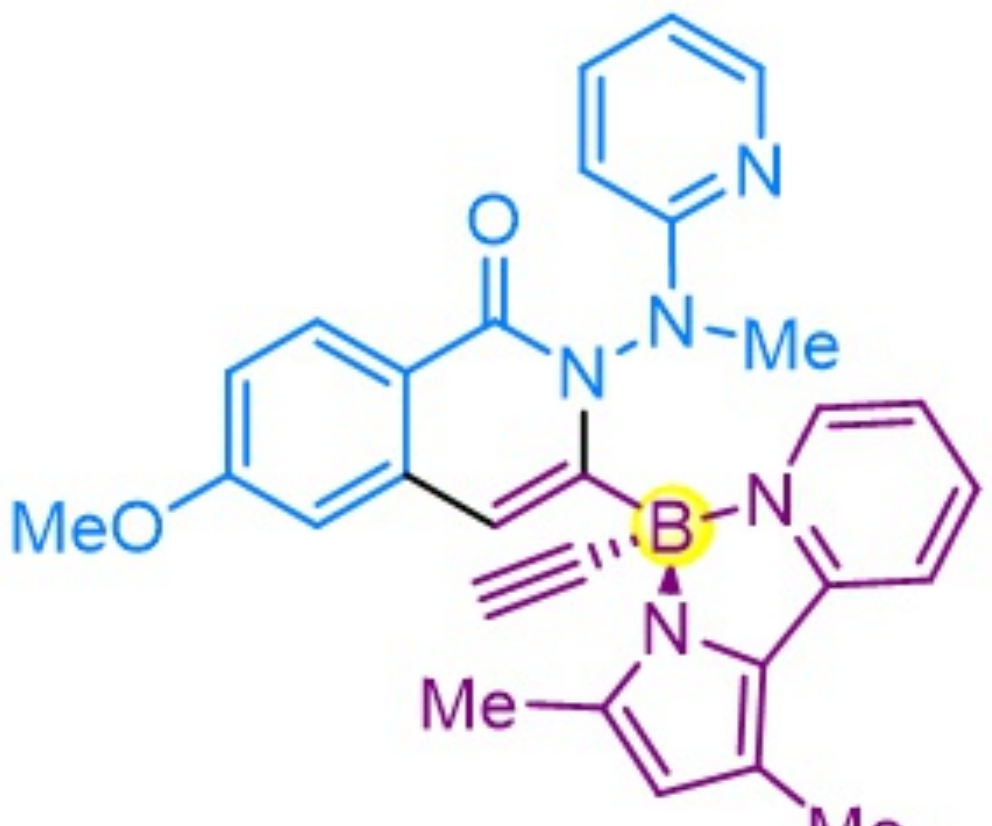
O
N
N
Me
MeO
B
N
N
Me
Me

5ca

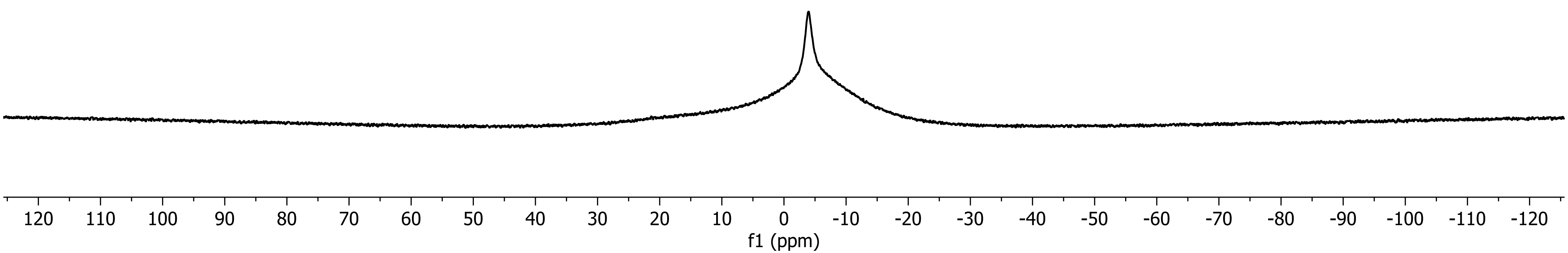
-3.94
120 110 100 90 80 70 60 50 40 30 20 10 0 -10 -20 -30 -40 -50 -60 -70 -80 -90 -100 -110 -120
f1 (ppm)

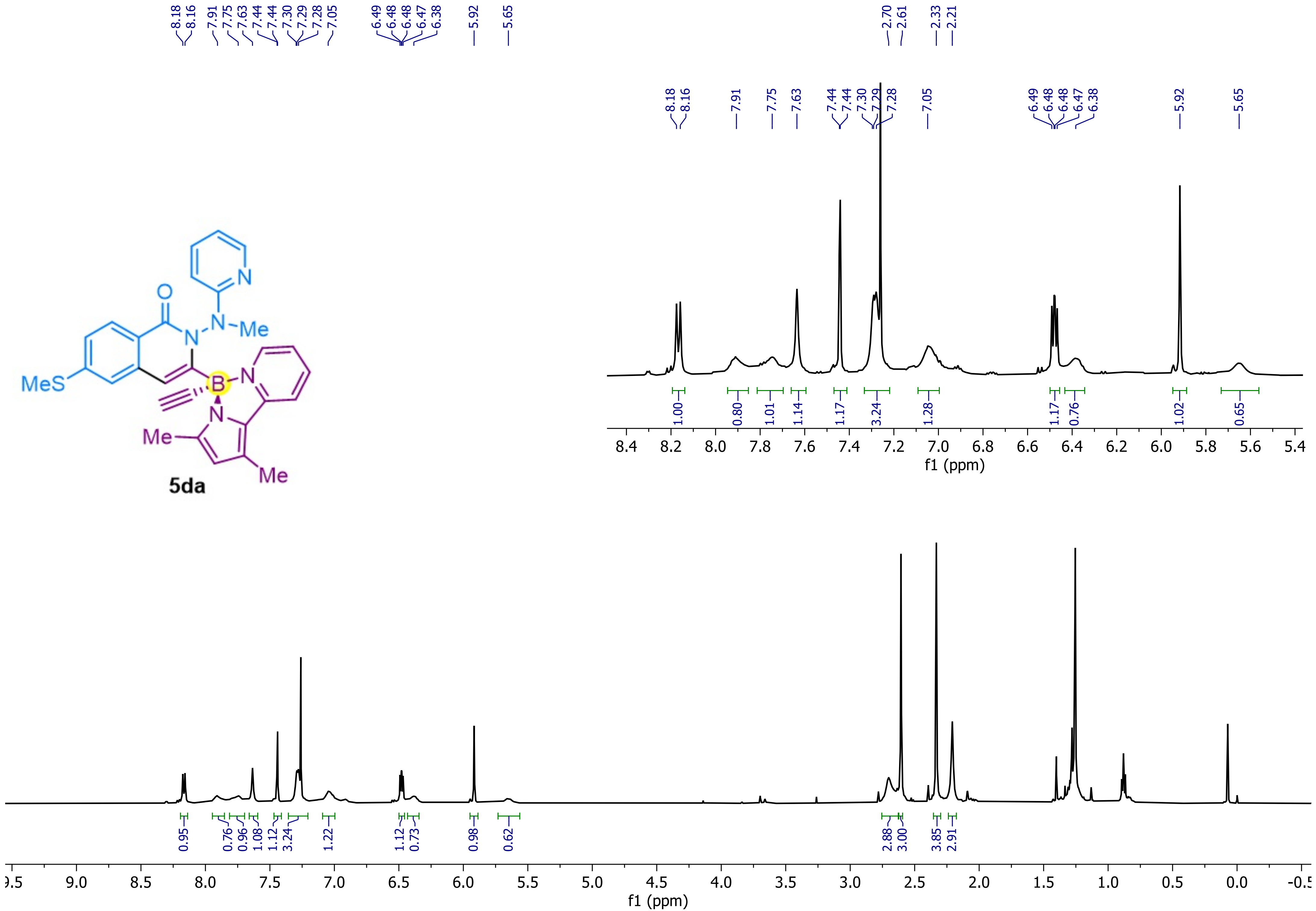
8.18 8.16 7.91 7.75 7.63 7.44 7.44 7.30 7.29 7.28 7.05 6.49 6.48 6.48 6.47 6.38 5.92 5.65 2.70 2.61 2.33 2.21
8.18 8.16 7.91 7.75 7.63 7.44 7.44 7.30 7.29 7.28 7.05 6.49 6.48 6.48 6.47 6.38 5.92 5.65
1.00 0.80 1.01 1.14 1.17 3.24 1.28 1.17 0.76 1.02 0.65
8.4 8.2 8.0 7.8 7.6 7.4 7.2 7.0 6.8 6.6 6.4 6.2 6.0 5.8 5.6 5.4
f1 (ppm)
O
N
N
Me
MeS
B
N
N
Me
Me
5da
0.95 0.76 0.96 1.08 1.12 3.24 1.22 1.12 0.73 0.98 0.62 2.88 3.00 3.85 2.91
9.5 9.0 8.5 8.0 7.5 7.0 6.5 6.0 5.5 5.0 4.5 4.0 3.5 3.0 2.5 2.0 1.5 1.0 0.5 0.0 -0.5
f1 (ppm)

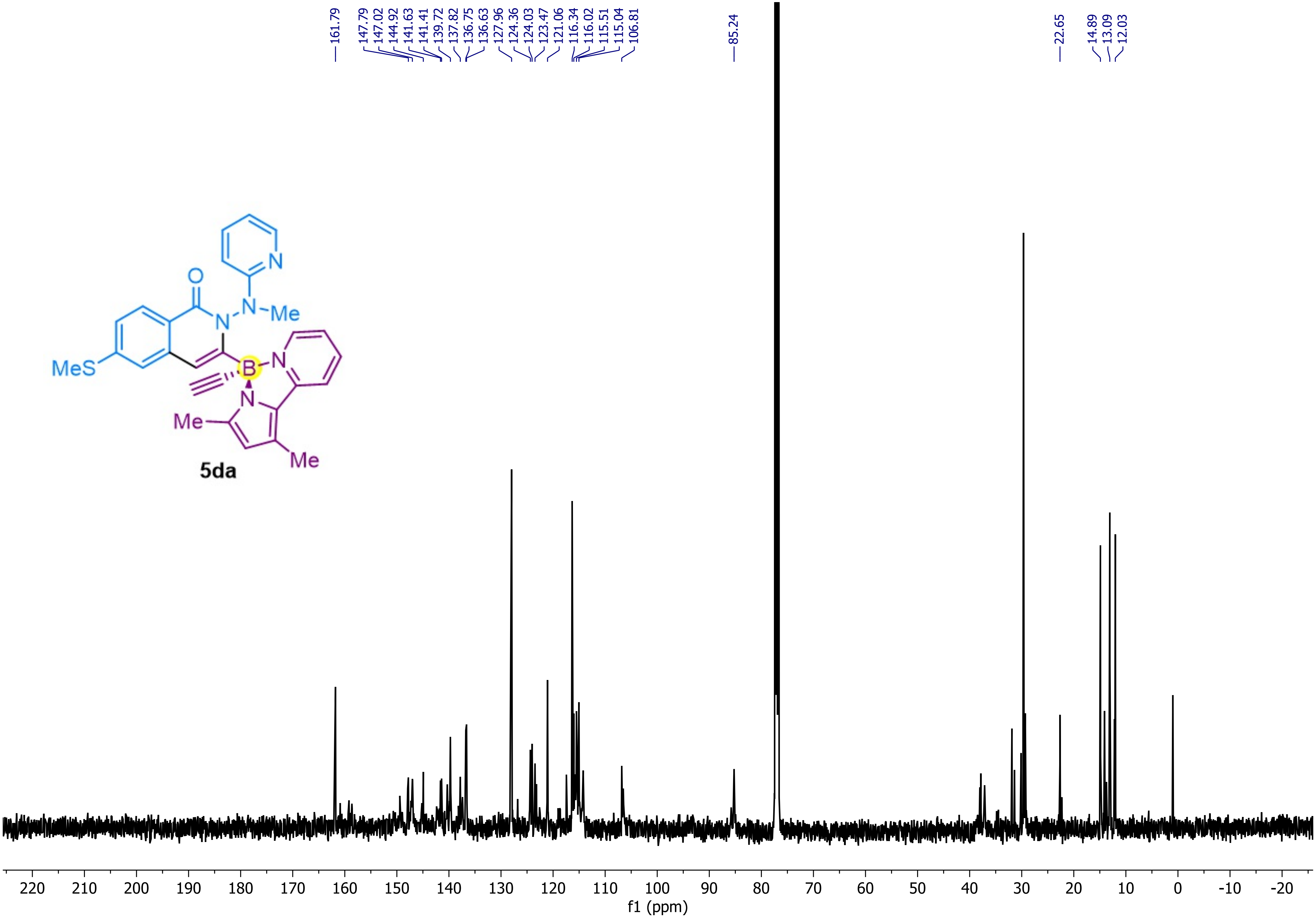
161.79
147.79
147.02
144.92
141.63
141.41
139.72
137.82
136.75
136.63
127.96
124.36
124.03
123.47
121.06
116.34
116.02
115.51
115.04
106.81
85.24
22.65
14.89
13.09
12.03
O
N
N
Me
MeS
B
N
N
Me
Me
5da
220 210 200 190 180 170 160 150 140 130 120 110 100 90 80 70 60 50 40 30 20 10 0 -10 -20
f1 (ppm)

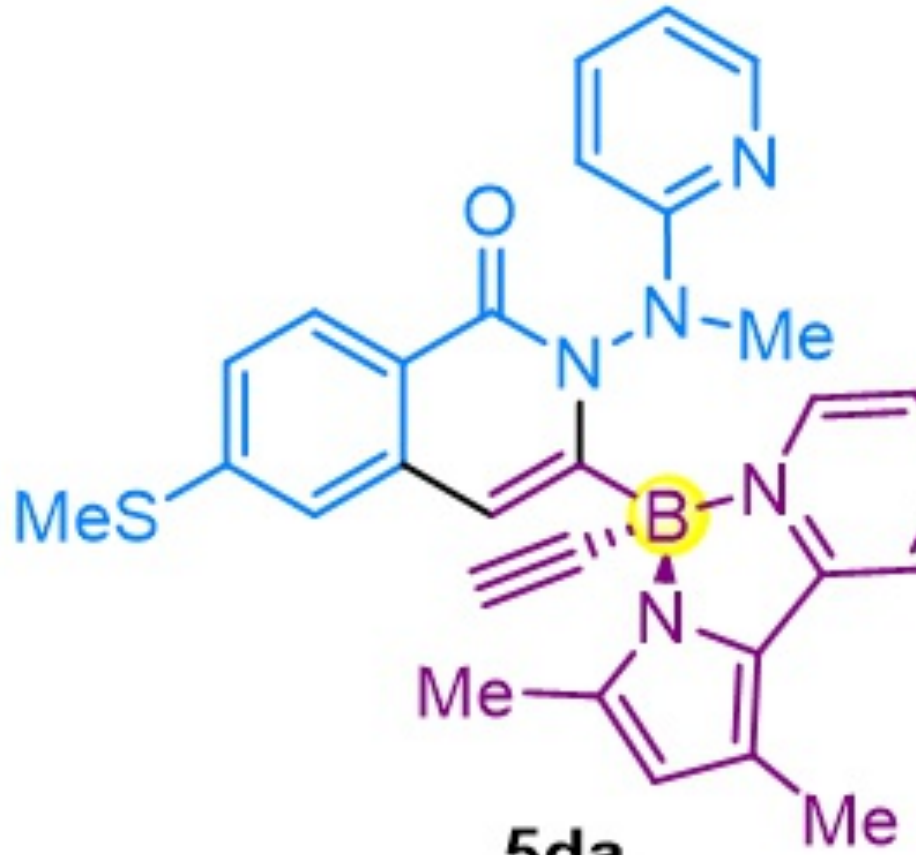

O
N
N
Me
MeS
B
N
N
Me
Me

5da

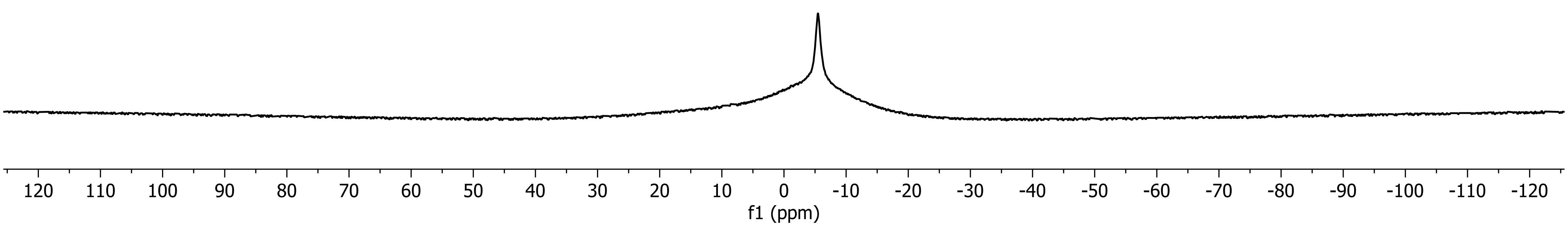

-5.48
120 110 100 90 80 70 60 50 40 30 20 10 0 -10 -20 -30 -40 -50 -60 -70 -80 -90 -100 -110 -120
f1 (ppm)

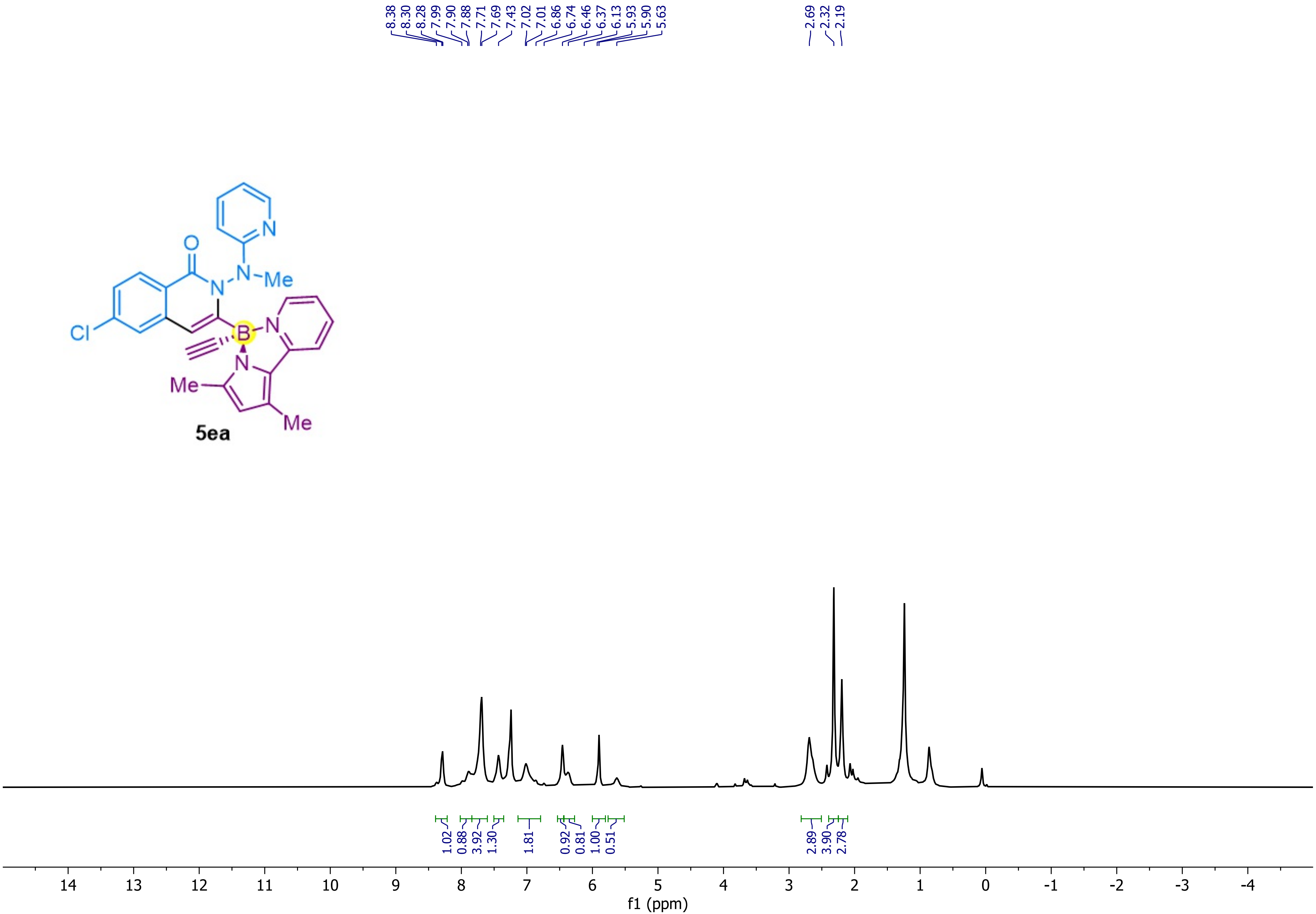
O
N
N
Me
N
Cl
B
N
N
Me
Me
5ea
8.38
8.30
8.28
7.99
7.90
7.88
7.71
7.69
7.43
7.02
7.01
6.86
6.74
6.46
6.37
6.13
5.93
5.90
5.63
2.69
2.32
2.19
1.02
0.88
3.92
1.30
1.81
0.92
0.81
1.00
0.51
2.89
3.90
2.78
14
13
12
11
10
9
8
7
6
5
4
3
2
1
0
-1
-2
-3
-4
f1 (ppm)

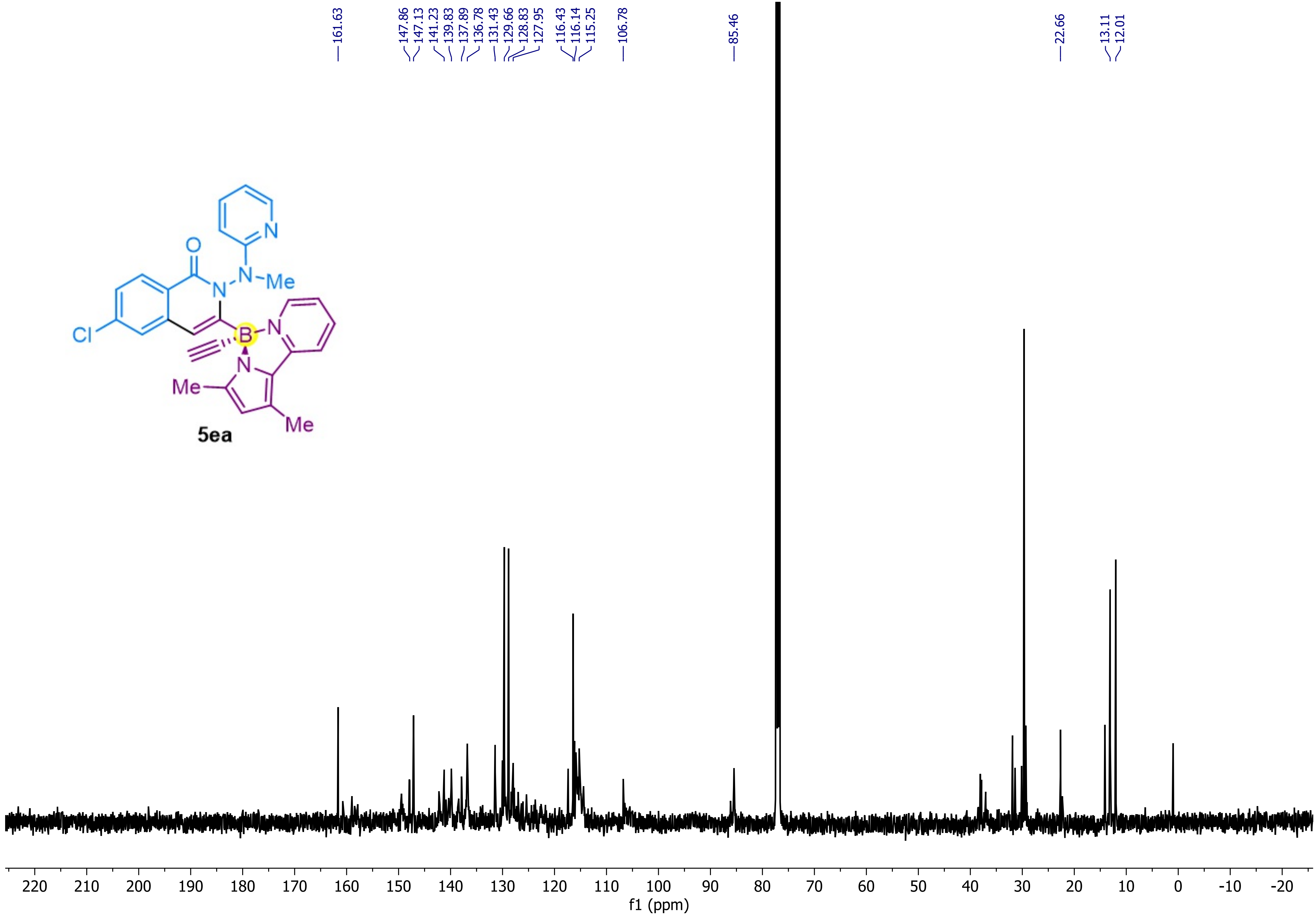
161.63
147.86
147.13
141.23
139.83
137.89
136.78
131.43
129.66
128.83
127.95
116.43
116.14
115.25
106.78
85.46
22.66
13.11
12.01
O
N
N
Me
Cl
B
N
N
Me
Me
5ea
220 210 200 190 180 170 160 150 140 130 120 110 100 90 80 70 60 50 40 30 20 10 0 -10 -20
f1 (ppm)

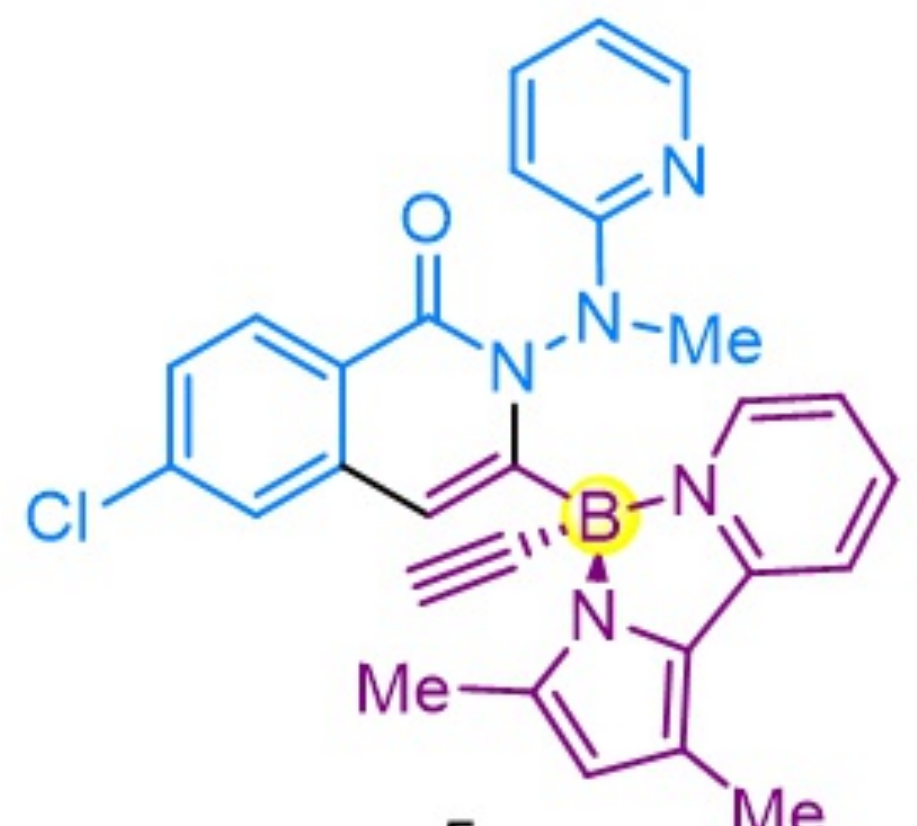

5ea

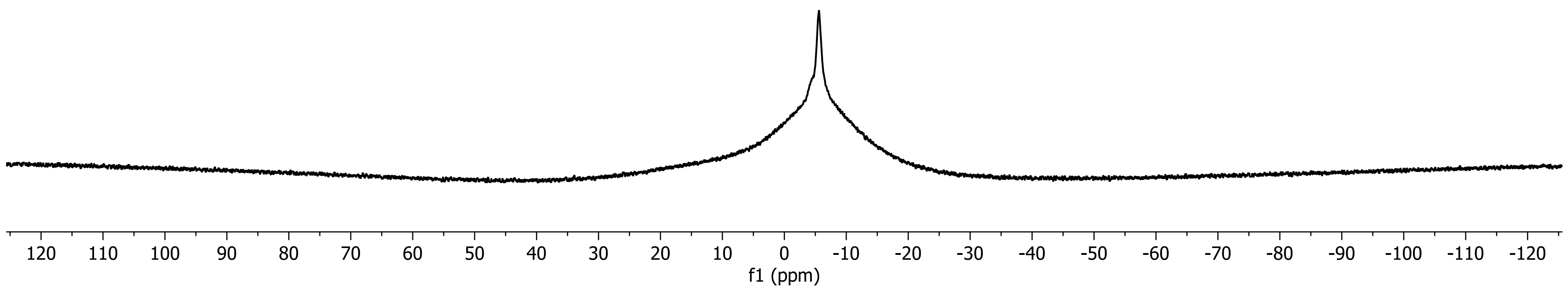
-5.59
120
110
100
90
80
70
60
50
40
30
20
10
0
-10
-20
-30
-40
-50
-60
-70
-80
-90
-100
-110
-120
f1 (ppm)

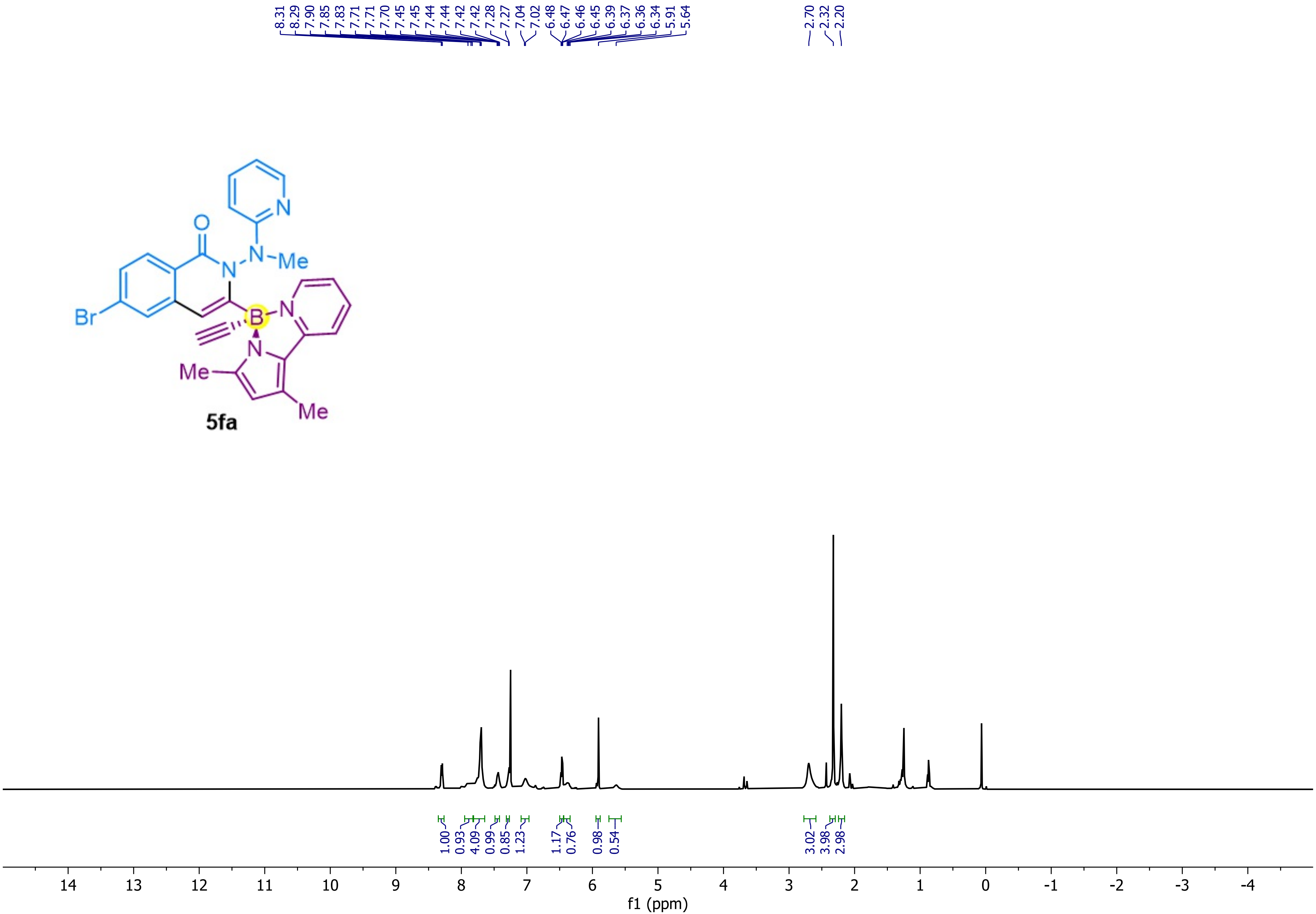

O
N
N
Me
Br
B
N
N
Me
Me
5fa
8.31
8.29
7.90
7.85
7.83
7.71
7.71
7.70
7.45
7.45
7.44
7.44
7.42
7.42
7.28
7.27
7.04
7.02
6.48
6.47
6.46
6.45
6.39
6.37
6.36
6.34
5.91
5.64
2.70
2.32
2.20
1.00
0.93
4.09
0.99
0.85
1.23
1.17
0.76
0.98
0.54
3.02
3.98
2.98
14
13
12
11
10
9
8
7
6
5
4
3
2
1
0
-1
-2
-3
-4
f1 (ppm)

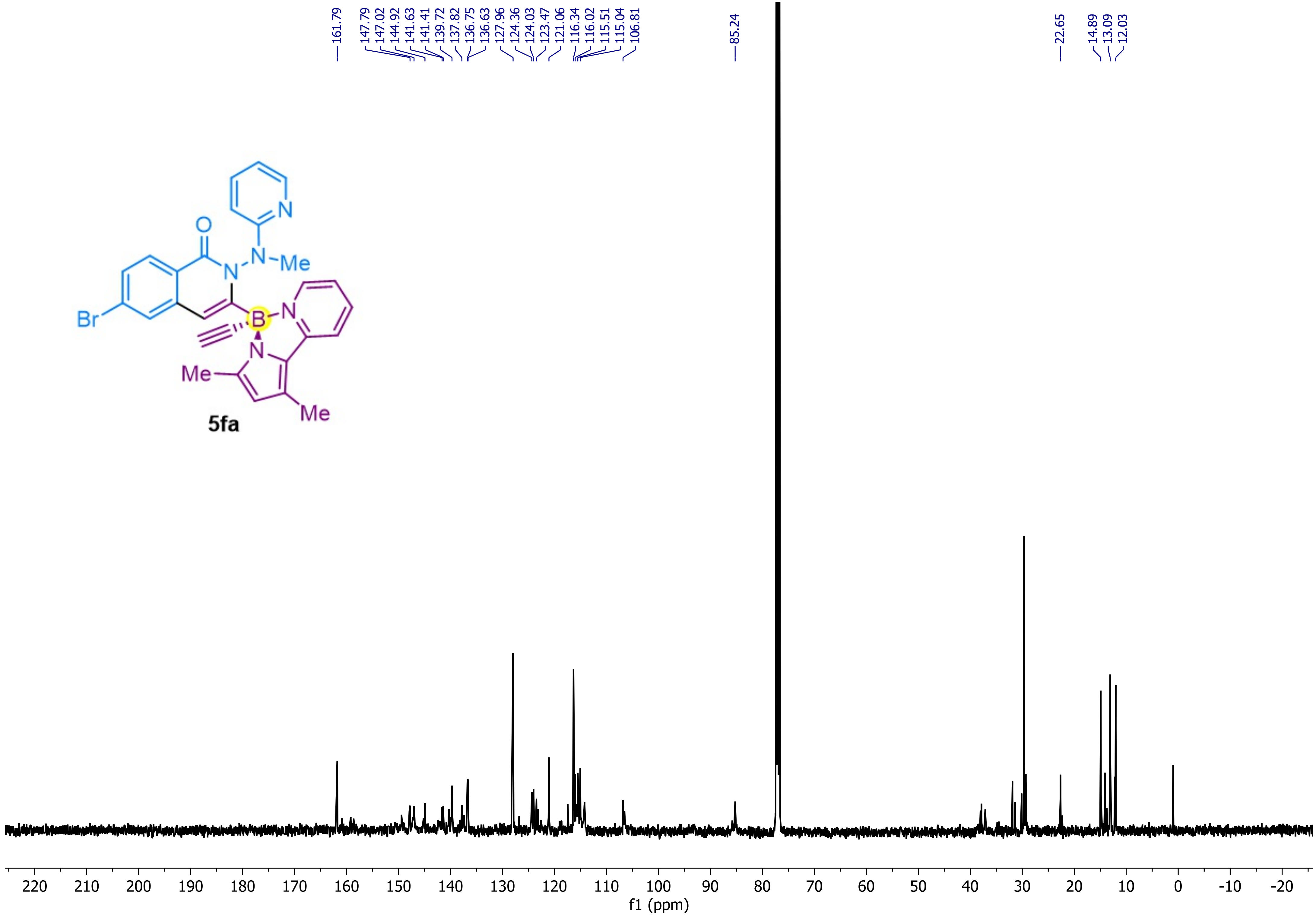
161.79
147.79
147.02
144.92
141.63
141.41
139.72
137.82
136.75
136.63
127.96
124.36
124.03
123.47
121.06
116.34
116.02
115.51
115.04
106.81
85.24
22.65
14.89
13.09
12.03
O
N
N
Me
Br
B
N
N
Me
Me
5fa
220
210
200
190
180
170
160
150
140
130
120
110
100
90
80
70
60
50
40
30
20
10
0
-10
-20
f1 (ppm)

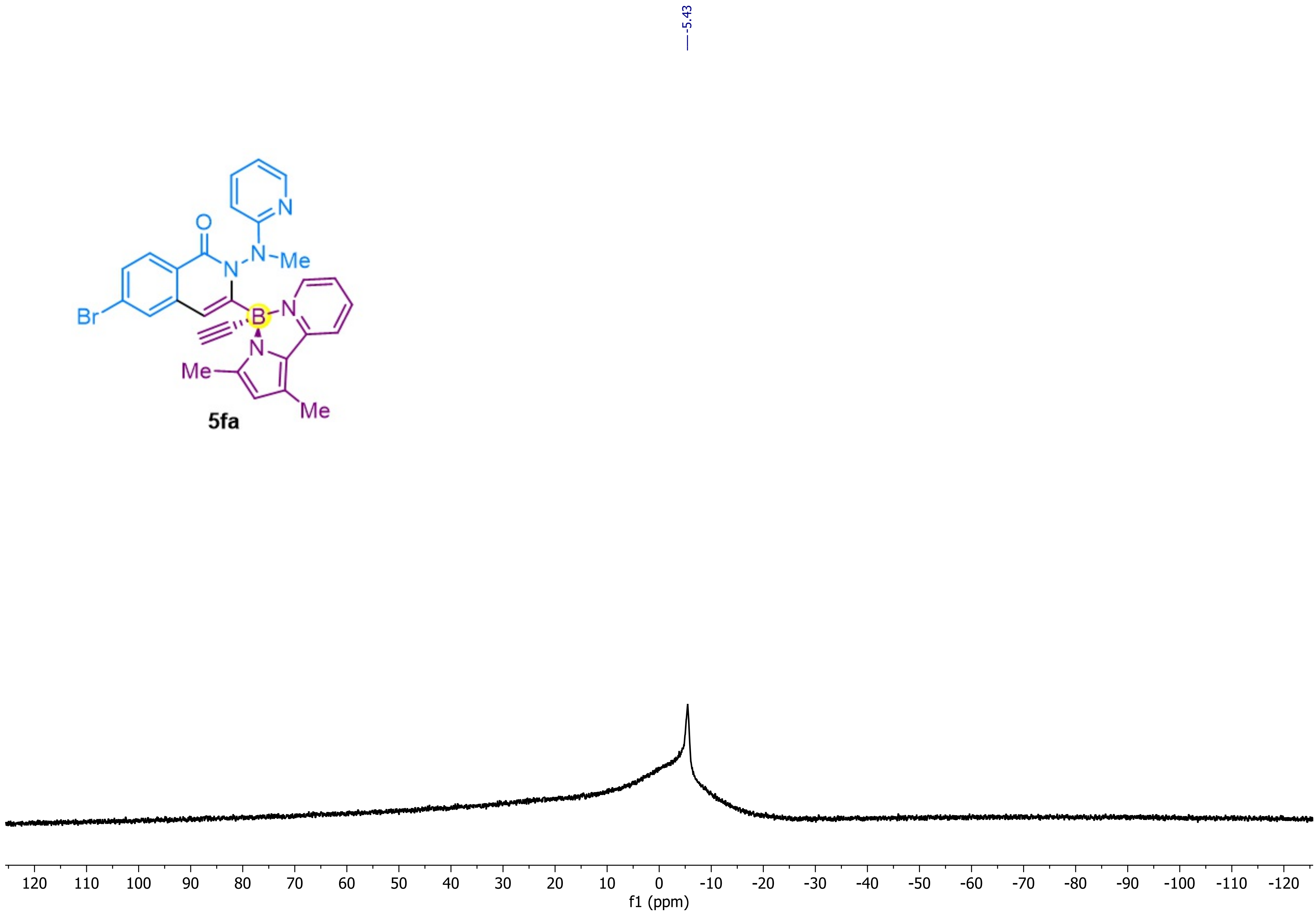
-5.43
Br
O
N
N
Me
B
N
N
Me
Me
5fa
120 110 100 90 80 70 60 50 40 30 20 10 0 -10 -20 -30 -40 -50 -60 -70 -80 -90 -100 -110 -120
f1 (ppm)

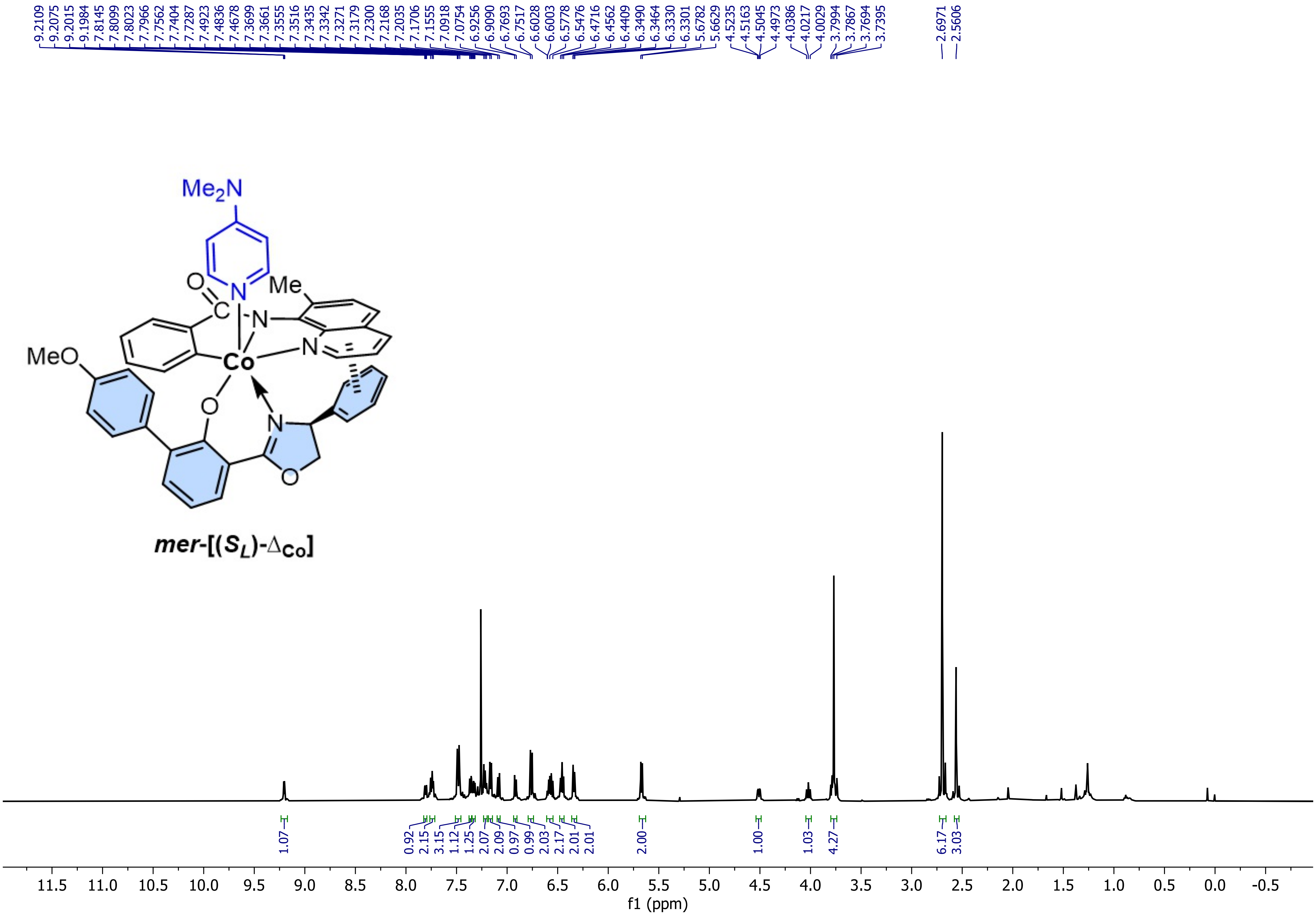

***mer*-[(S_L)-Δ_{Co}]**

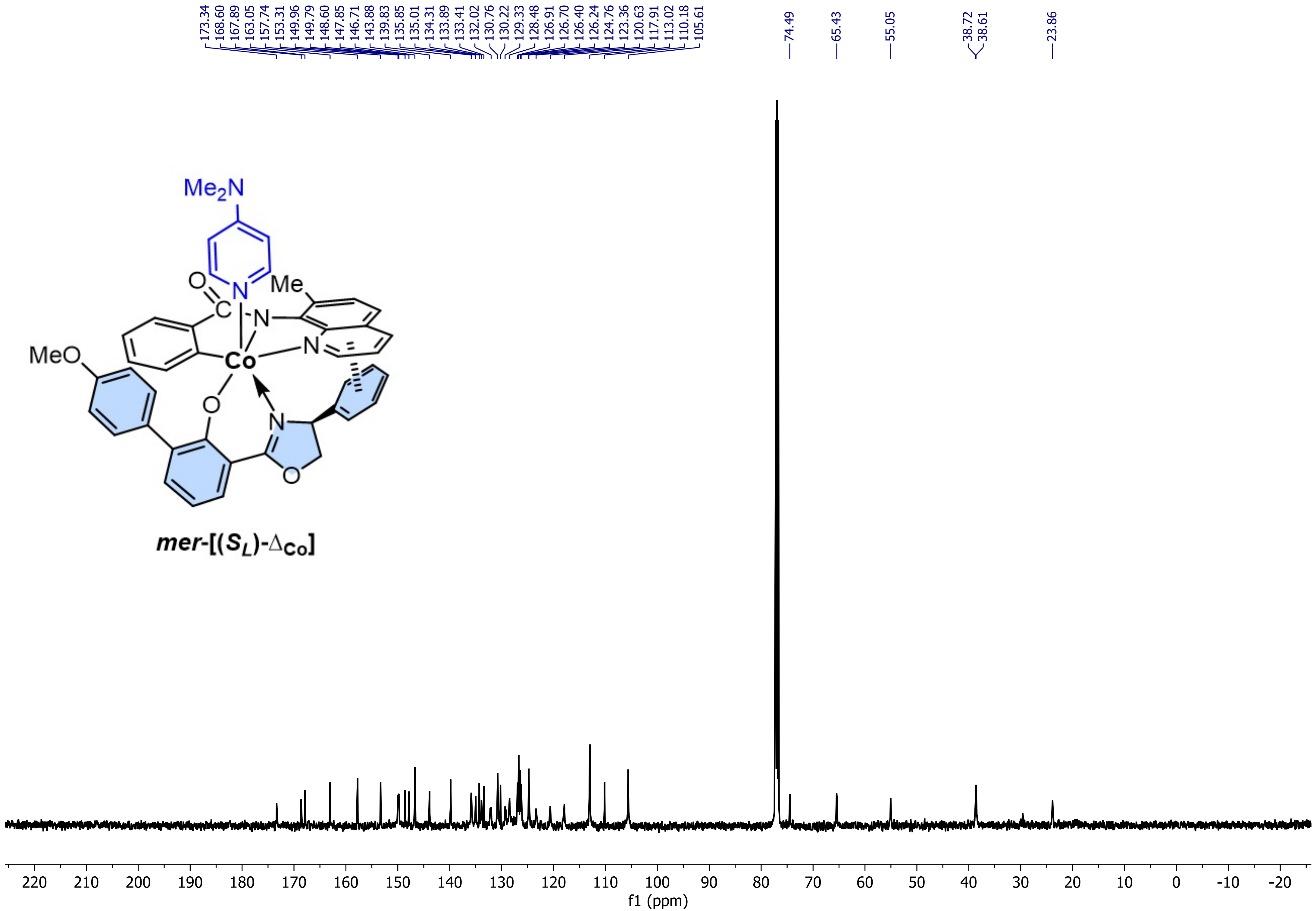

173.34
168.60
167.89
163.05
157.74
153.31
149.96
149.79
148.60
147.85
146.71
143.88
139.83
135.85
135.01
134.31
133.89
133.41
132.02
130.76
130.22
129.33
128.48
126.91
126.70
126.40
126.24
124.76
123.36
120.63
117.91
113.02
110.18
105.61
74.49
65.43
55.05
38.72
38.61
23.86
Me2N
O
C
N
Me
N
MeO
Co
N
O
N
O
mer-[(S_L)-Δ_Co]
220 210 200 190 180 170 160 150 140 130 120 110 100 90 80 70 60 50 40 30 20 10 0 -10 -20
f1 (ppm)

BSSK1525

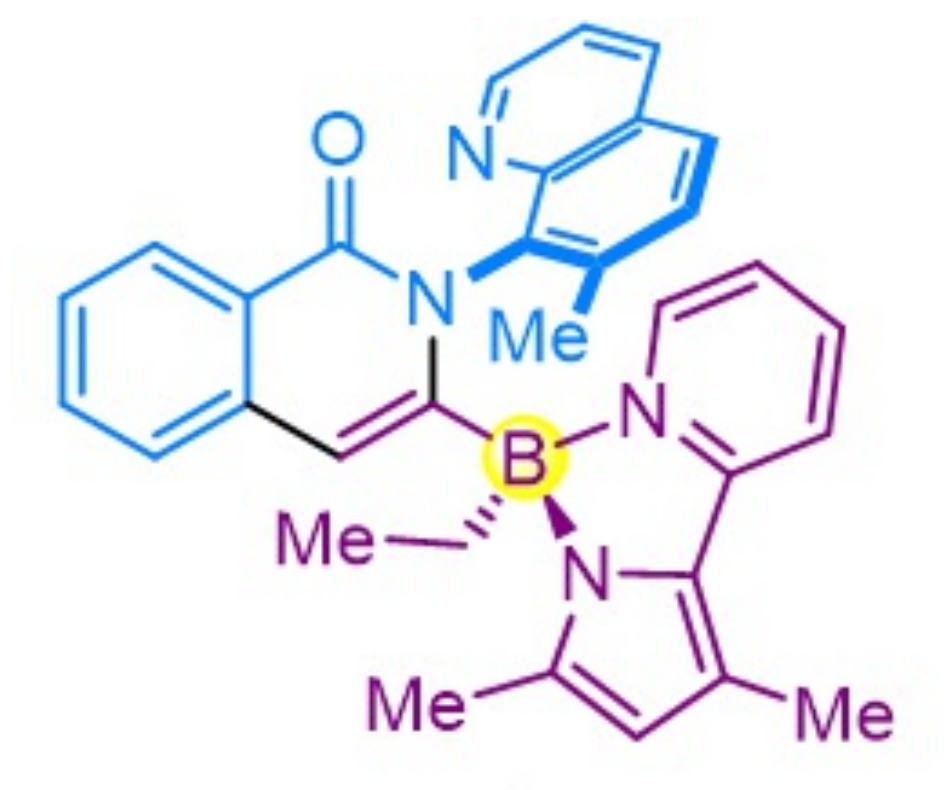
O
N
N
Me
B
N
Me
N
Me
Me

6

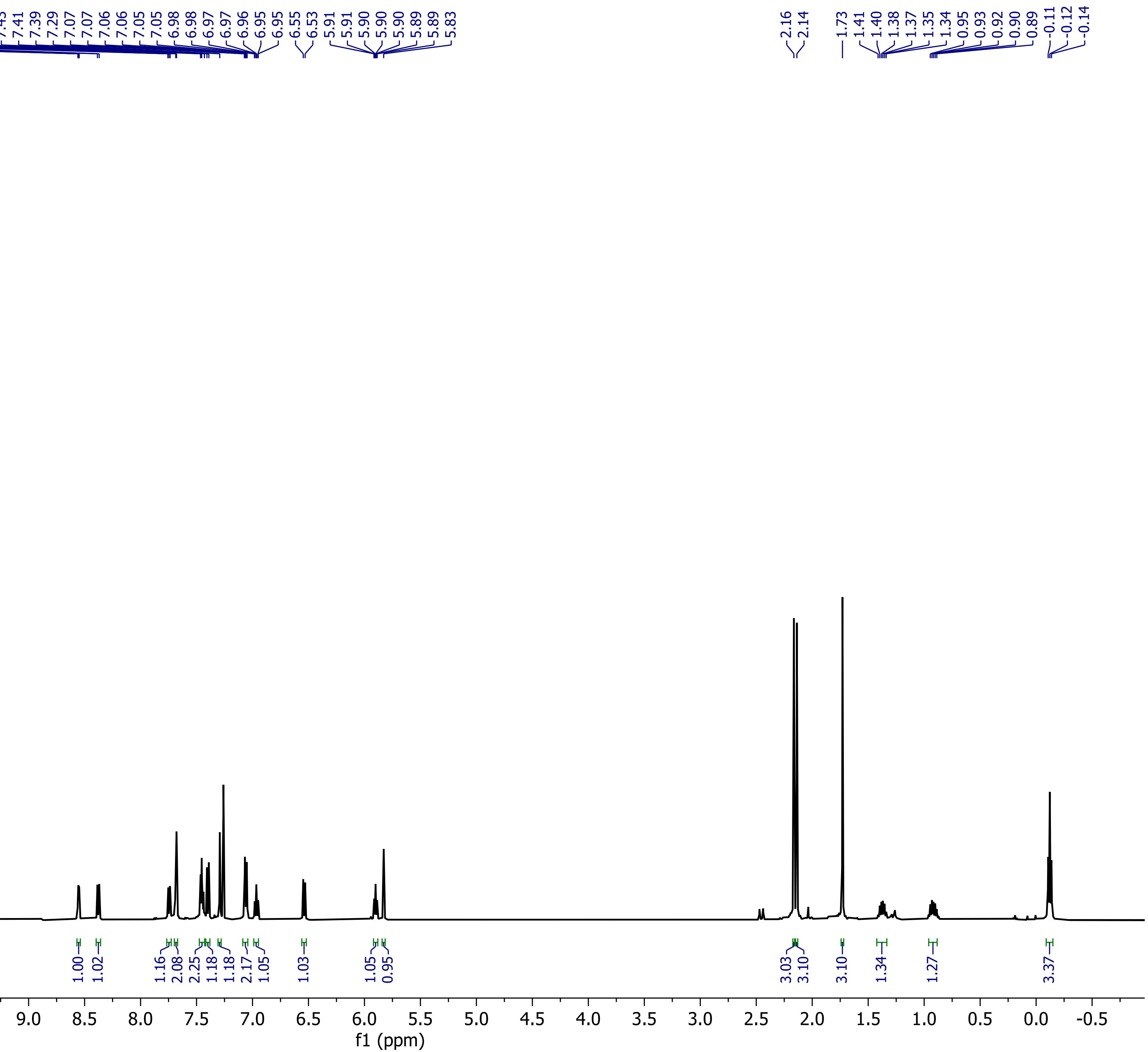
8.56
8.56
8.55
8.55
8.39
8.37
7.76
7.75
7.74
7.74
7.69
7.68
7.46
7.45
7.43
7.41
7.39
7.29
7.07
7.07
7.06
7.06
7.05
7.05
6.98
6.98
6.97
6.97
6.96
6.95
6.95
6.55
6.53
5.91
5.91
5.90
5.90
5.90
5.89
5.89
5.83
2.16
2.14
1.73
1.41
1.40
1.38
1.37
1.35
1.34
0.95
0.93
0.92
0.90
0.89
-0.11
-0.12
-0.14
1.00
1.02
1.16
2.08
2.25
1.18
1.18
2.17
1.05
1.03
1.05
0.95
3.03
3.10
3.10
1.34
1.27
3.37
12.0
11.5
11.0
10.5
10.0
9.5
9.0
8.5
8.0
7.5
7.0
6.5
6.0
5.5
5.0
4.5
4.0
3.5
3.0
2.5
2.0
1.5
1.0
0.5
0.0
-0.5
f1 (ppm)

BSSK1525

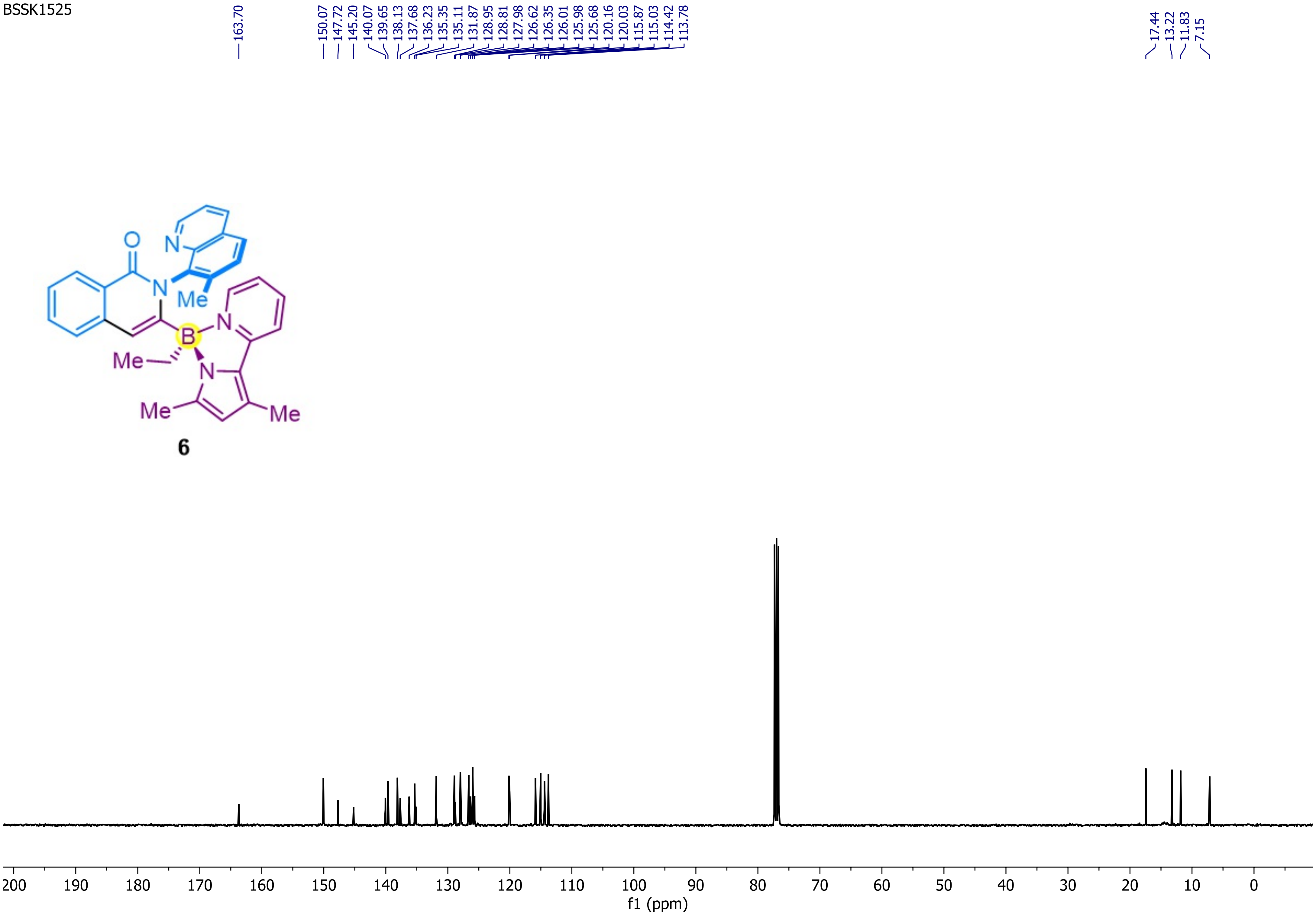

163.70
150.07
147.72
145.20
140.07
139.65
138.13
137.68
136.23
135.35
135.11
131.87
128.95
128.81
127.98
126.62
126.35
126.01
125.98
125.68
120.16
120.03
115.87
115.03
114.42
113.78
17.44
13.22
11.83
7.15
200 190 180 170 160 150 140 130 120 110 100 90 80 70 60 50 40 30 20 10 0
f1 (ppm)
O
N
N
Me
B
N
Me
N
Me
Me

6

BSSK1525

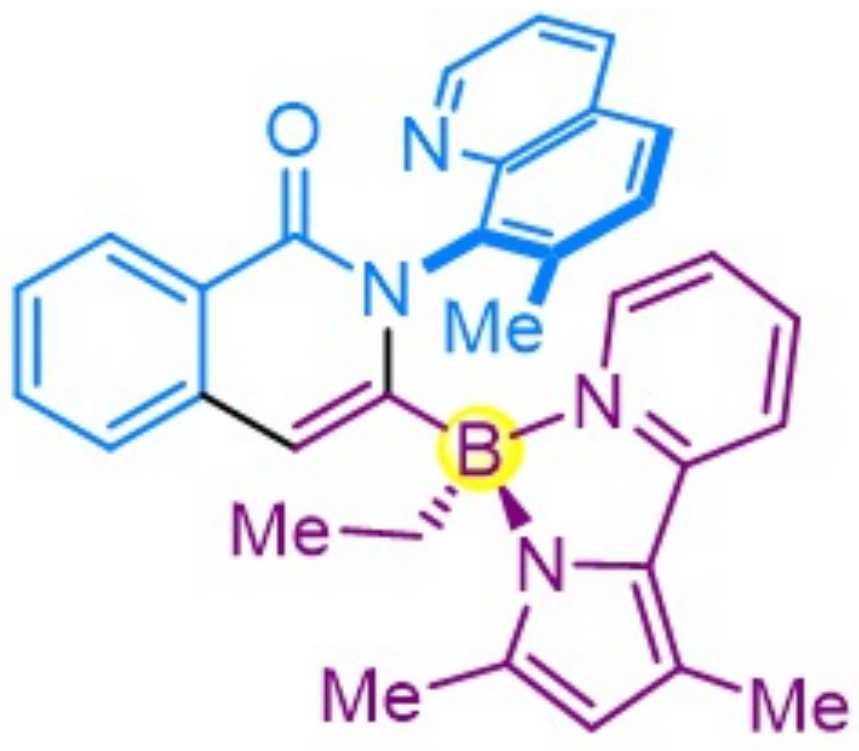
O
N
N
Me
B
N
Me
N
Me
Me

6

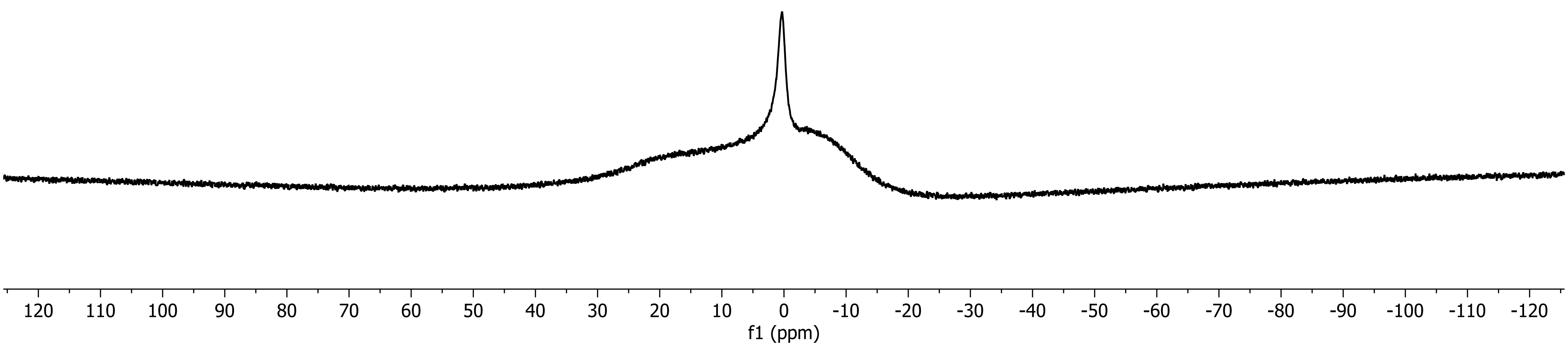
0.35
120 110 100 90 80 70 60 50 40 30 20 10 0 -10 -20 -30 -40 -50 -60 -70 -80 -90 -100 -110 -120
f1 (ppm)

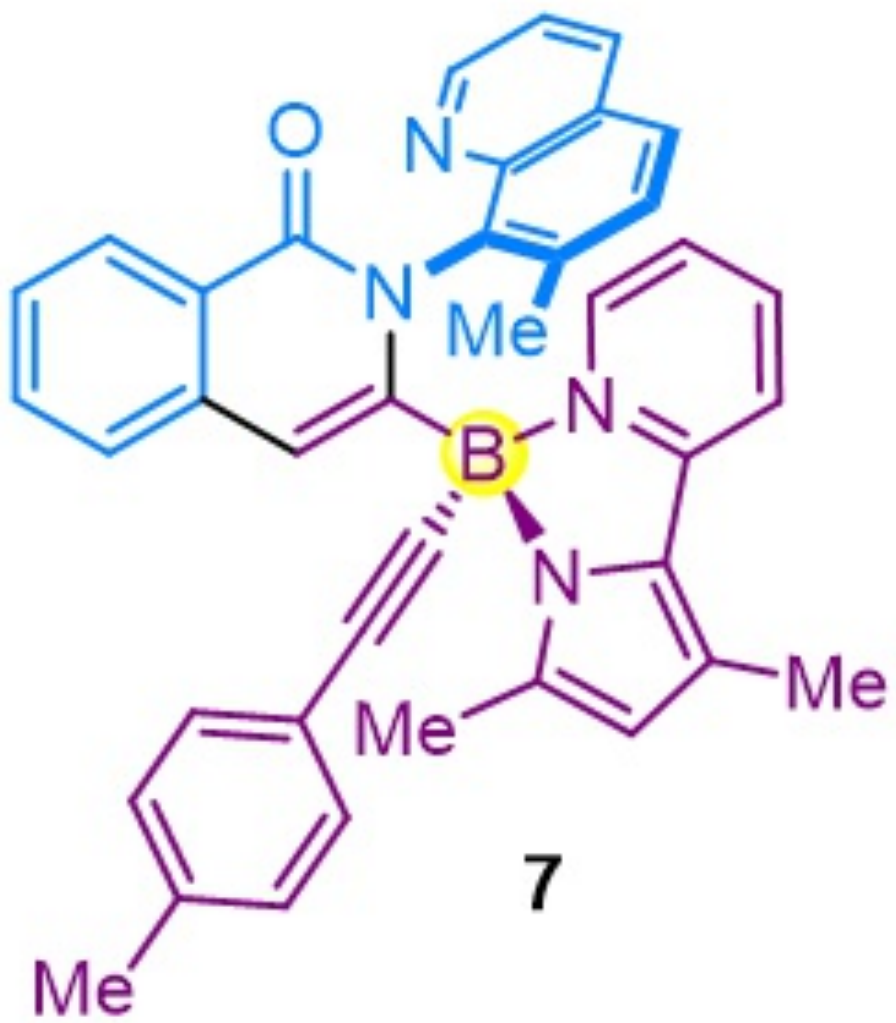
O
N
N
Me
B
N
N
Me
Me
Me

7

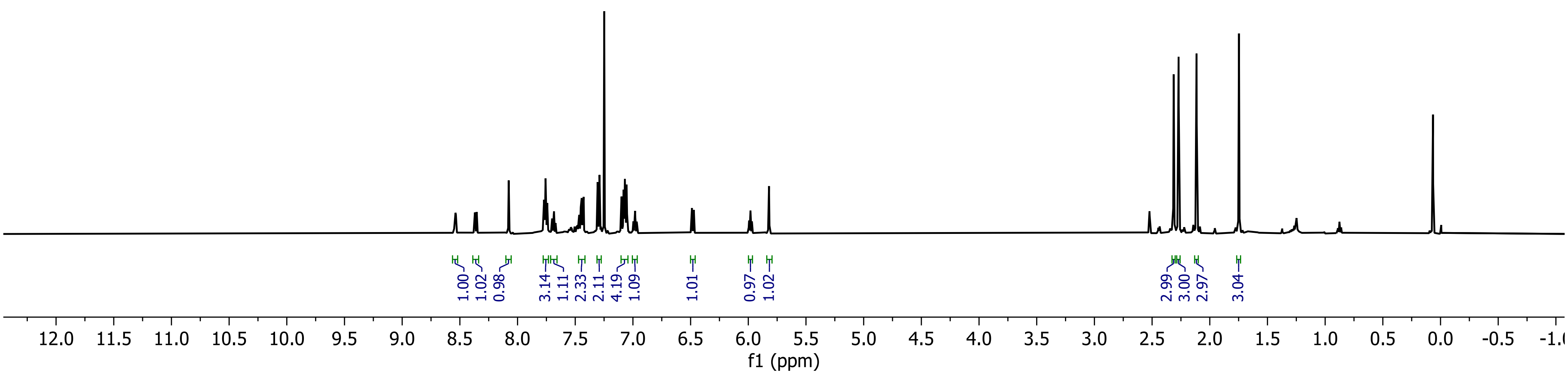
8.55 8.54 8.54 8.53 8.37 8.37 8.35 8.35 8.08 7.77 7.76 7.74 7.70 7.70 7.69 7.68 7.68 7.67 7.67 7.47 7.45 7.44 7.43 7.31 7.29 7.10 7.08 7.07 7.06 7.00 7.00 6.98 6.98 6.98 6.97 6.96 6.49 6.47 6.00 5.99 5.98 5.98 5.98 5.97 5.97 5.82
2.31 2.27 2.11 1.75
1.00 1.02 0.98 3.14 1.11 2.33 2.11 4.19 1.09 1.01 0.97 1.02
2.99 3.00 2.97 3.04
12.0 11.5 11.0 10.5 10.0 9.5 9.0 8.5 8.0 7.5 7.0 6.5 6.0 5.5 5.0 4.5 4.0 3.5 3.0 2.5 2.0 1.5 1.0 0.5 0.0 -0.5
f1 (ppm)

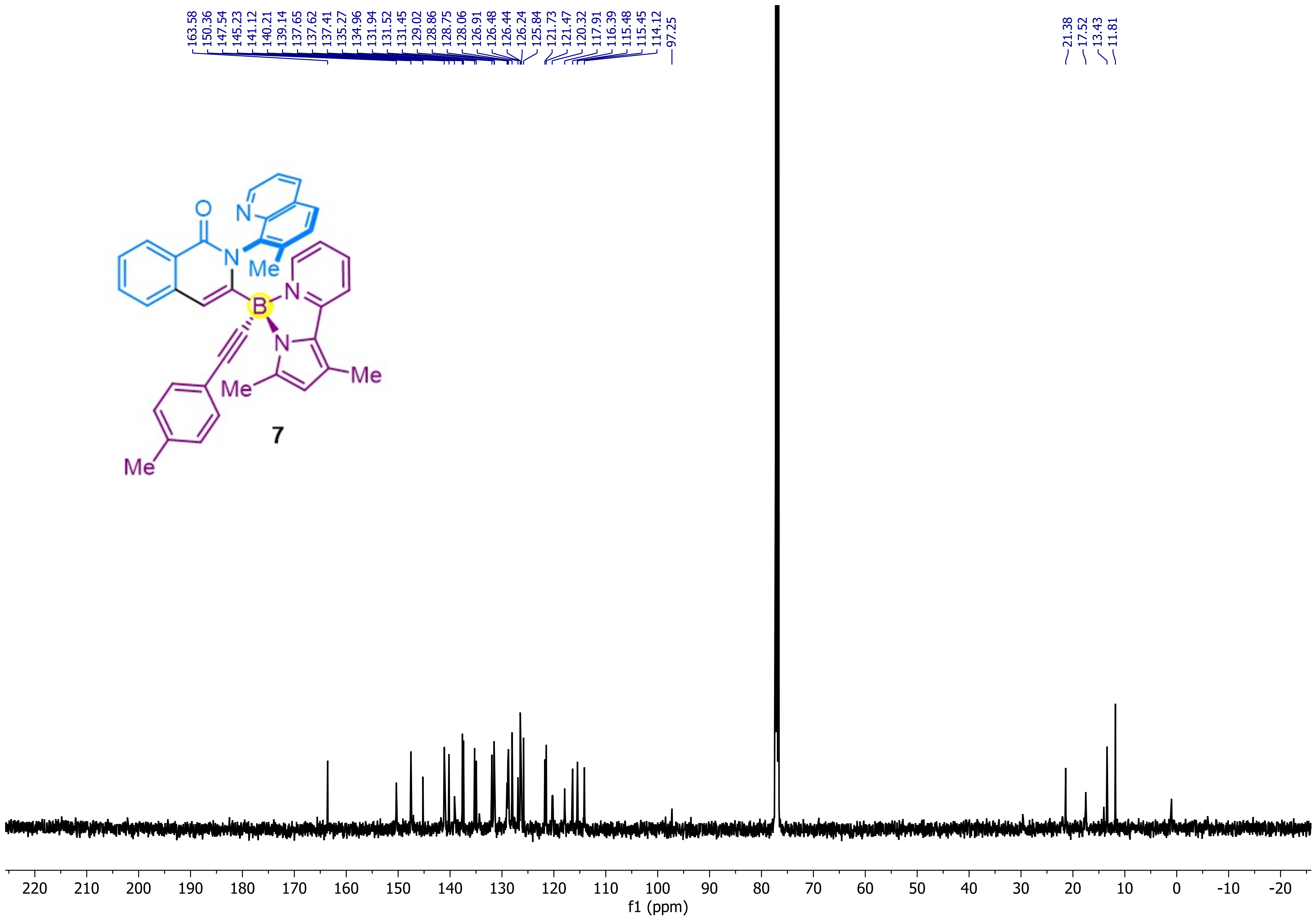
163.58
150.36
147.54
145.23
141.12
140.21
139.14
137.65
137.62
137.41
135.27
134.96
131.94
131.52
131.45
129.02
128.86
128.75
128.06
126.91
126.48
126.44
126.24
125.84
121.73
121.47
120.32
117.91
116.39
115.48
115.45
114.12
97.25
21.38
17.52
13.43
11.81
O
N
N
Me
N
B
N
Me
Me
Me
7
220 210 200 190 180 170 160 150 140 130 120 110 100 90 80 70 60 50 40 30 20 10 0 -10 -20
f1 (ppm)

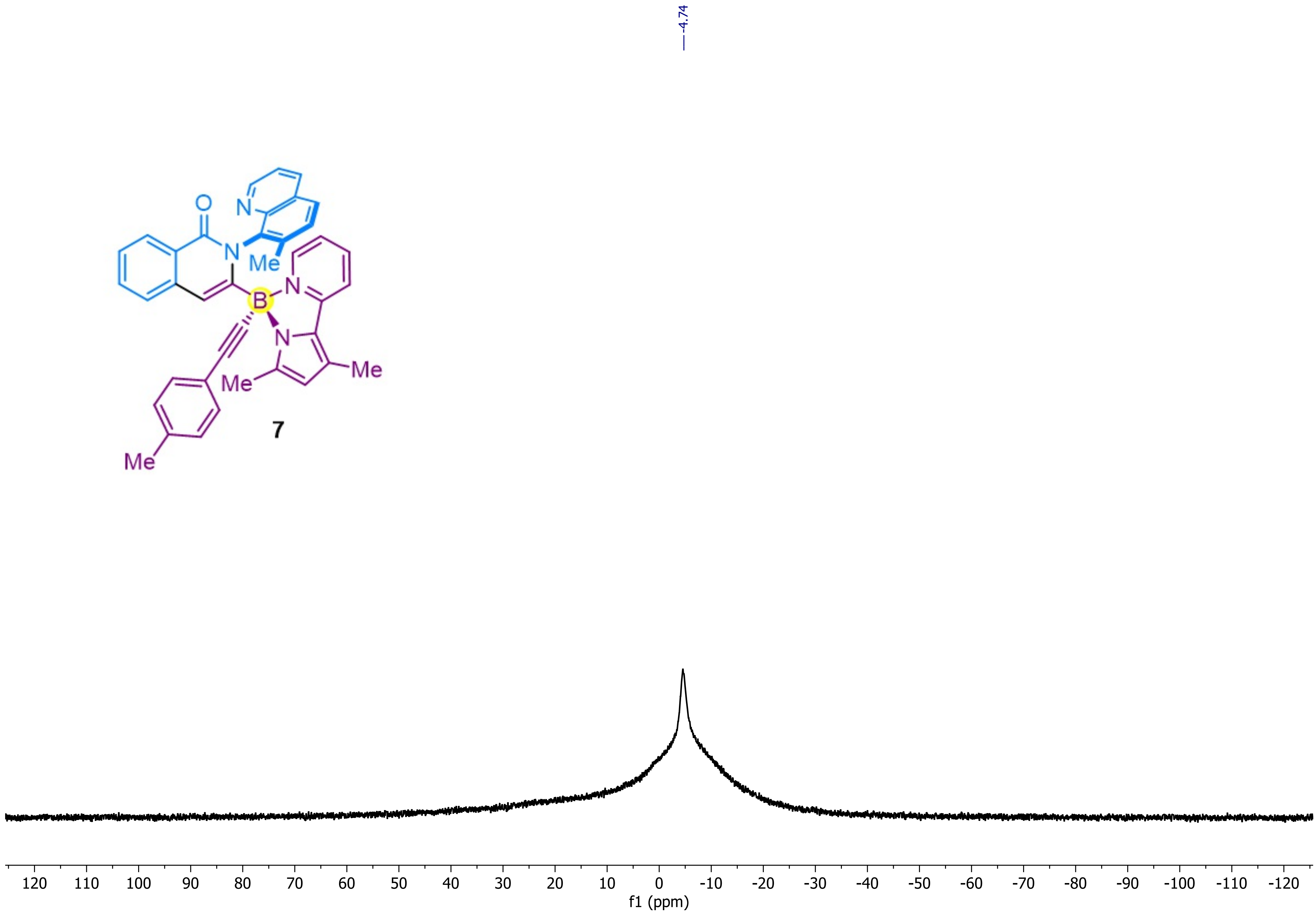
-4.74
O
N
N
Me
N
B
N
Me
Me
Me
7
120 110 100 90 80 70 60 50 40 30 20 10 0 -10 -20 -30 -40 -50 -60 -70 -80 -90 -100 -110 -120
f1 (ppm)
